# Rice_Phospho 1.0: a new rice-specific SVM predictor for protein phosphorylation sites

**DOI:** 10.1038/srep11940

**Published:** 2015-07-07

**Authors:** Shoukai Lin, Qi Song, Huan Tao, Wei Wang, Weifeng Wan, Jian Huang, Chaoqun Xu, Vivien Chebii, Justine Kitony, Shufu Que, Andrew Harrison, Huaqin He

**Affiliations:** 1 College of Life Sciences, Fujian Agriculture and Forestry University, Fuzhou 350002, China; 2 Department of Mathematical Sciences, University of Essex, Wivenhoe Park, Colchester, CO4 3SQ, UK

## Abstract

Experimentally-determined or computationally-predicted protein phosphorylation sites for distinctive species are becoming increasingly common. In this paper, we compare the predictive performance of a novel classification algorithm with different encoding schemes to develop a rice-specific protein phosphorylation site predictor. Our results imply that the combination of Amino acid occurrence Frequency with Composition of K-Spaced Amino Acid Pairs (AF-CKSAAP) provides the best description of relevant sequence features that surround a phosphorylation site. A support vector machine (SVM) using AF-CKSAAP achieves the best performance in classifying rice protein phophorylation sites when compared to the other algorithms. We have used SVM with AF-CKSAAP to construct a rice-specific protein phosphorylation sites predictor, Rice_Phospho 1.0 (http://bioinformatics.fafu.edu.cn/rice_phospho1.0). We measure the Accuracy (ACC) and Matthews Correlation Coefficient (MCC) of Rice_Phospho 1.0 to be 82.0% and 0.64, significantly higher than those measures for other predictors such as Scansite, Musite, PlantPhos and PhosphoRice. Rice_Phospho 1.0 also successfully predicted the experimentally identified phosphorylation sites in LOC_Os03g51600.1, a protein sequence which did not appear in the training dataset. In summary, Rice_phospho 1.0 outputs reliable predictions of protein phosphorylation sites in rice, and will serve as a useful tool to the community.

Phosphorylation is one of the most important protein post-translation modification (PTMs) in eukaryotes. It plays essential roles in the majority of biological pathways, regulating cellular processes like metabolism, proliferation, differentiation and apoptosis^1^. More than 30% of all eukaryotic proteins are estimated to undergo reversible phosphorylation^2^. Biochemically, phosphorylation results in a transfer of a phosphate moiety from adenosine triphosphate (ATP) to the acceptor residue, thereby generating adenosine diphosphate (ADP) whilst inducing the residue to be phosphorylated. The process of protein phosphorylation usually involves distinct short peptide motifs, or patterns including phosphorylation of substrate sites, being recognized by different protein kinases which then leads to a phosphate moiety being typically attached to either Serine (Ser), Threonine (Thr) or Tyrosine (Tyr) residues.

Conventional experimental identifications and recent advances in high throughput Mass Spectrometry (MS) techniques have generated a large number of phosphorylated substrates with confirmed phosphorylation sites. In parallel, a series of algorithms have also been developed to predict phosphorylation sites from amino acid sequence. These range from simple motif or pattern searches to more complex machine learning methods like Artificial Neural Networks (ANN) and Support Vector Machines (SVM). Examples of such predictive algorithms include NetPhos^3^, NetPhosK^4^, KinasePhos^5^, DISPHOS^6^, Scansite^7^, PPSP^8^, GPS^9^, PredPhospho^10^ and Musite^11^.

Most computational phosphorylation site predictors are not organism-specific predictors. However, with the increases in experimentally verified protein phosphorylation sites for different organisms, an increasing goal is to develop organism-specific phosphorylation predictors, such has occurred for yeast^12^, *Arabidopsis*^13^ and rice^1^. The yeast-specific predictor, NetPhosYeast, outperforms existing generic predictors in the identification of phosphorylation sites in yeast^12^. PhosPhAt predicting phosphorylated-Serine sites for *Arabidopsis* is found to perform better with Arabidopsis sequences than other generic predictors^13^. Furthermore, a protein family specific phosphorylation site predictor, PhosTryp, was developed specifically for the trypanosomatidae family in parasitic protozoa^14^.

We have focused our efforts on Rice (*Oryza sativa* L.). Rice is considered a model plant species of the monocots group, it has a sequenced genome^15^ and serves as a cornerstone for the study of functional genomics in cereal plants^16^. Phosphorylation proteins have been identified in rice treated with various hormones^17^ and under different environmental conditions, including high salinity^18^, drought^19^ and high temperature^20^. Many phosphorylation sites in rice were identified by Nakagami *et al.*^21^ However, current predictors perform poorly when individually used to predict phosphorylation sites in rice phosphoproteins^1^. We have therefore established a meta-predictor for rice-specific phosphorylation sites^1^. However, this rice-specific predictor was not trained directly by the rice phosphorylation sites data, but was developed by integrating six newly predicting programs, including NetPhosK, NetPhos2.0, KinasePhos, PrePhospho 1.0, Scansite and DISPHOS. This paper augments this earlier work by building a Support Vector Machine (SVM) prediction model using experimentally identified rice phosphorylation sites directly.

## Results

### Performance of the 6 encoding schemes

**Figure 1:**
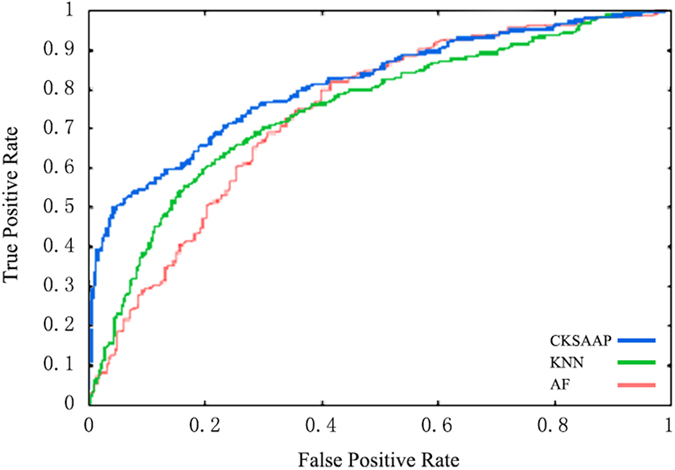
ROC curves of predicting performance of SVM with 3 different sole encoding schemes. *In the diagrams, the increased area under the ROC indicates the improved classification performance. The same below.

The performance of the three sole encoding schemes was measured by using different sizes of datasets and with SVM used as the classifier. CKSAAP performed best among the three sole encoding schemes (Fig. 1). However, with the size of dataset increasing, the performance of SVM with CKSAAP decreased, SVM with AF kept fluctuating, while that of SVM with KNN increased (Table 1). The same changing trends (CKSSAP decreasing, AF fluctuating and KNN increasing) in performance of SVM with AF, KNN or CKSAAP was also true when the ratio of (+) sites to (−) sites increased (Table 1).

**Figure 2:**
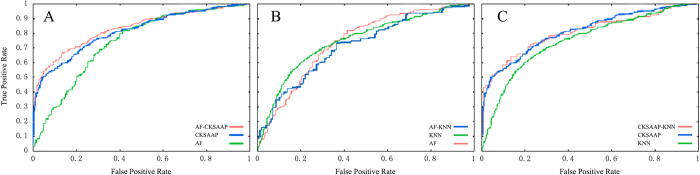
ROC curves of predicting performance of SVM with the combining encoding schemes. *A. ROC curves of SVM with AF-CKSAAP, AF and CKSAAP. **B**. ROC curves of SVM with AF-KNN, AF and KNN. **C**. ROC curves of SVM with CKSAAP-KNN, KNN and CKSAAP.

The performance of AF combined with CKAAP (AF-CKSAAP) was better than the sole encoding scheme, AF or CKSAAP (Fig. 2A). The same was true for AF combined with KNN (AF-KNN) (Fig. 2B). However, CKSAAP combined with KNN (CKSAAP-KNN) outperformed KNN, but did not outperform CKSAAP (Fig. 2C). In the preliminary experiment, we found that the combination of all the three encoding schemes did not significantly outperform CKSAAP (Data not shown) but increased feature dimensions. This result implies that AF, KNN and CKSAAP might be complementary to each other to some extent, especially AF and CKSAAP.

### Performance of 4 different classifiers

**Figure 3:**
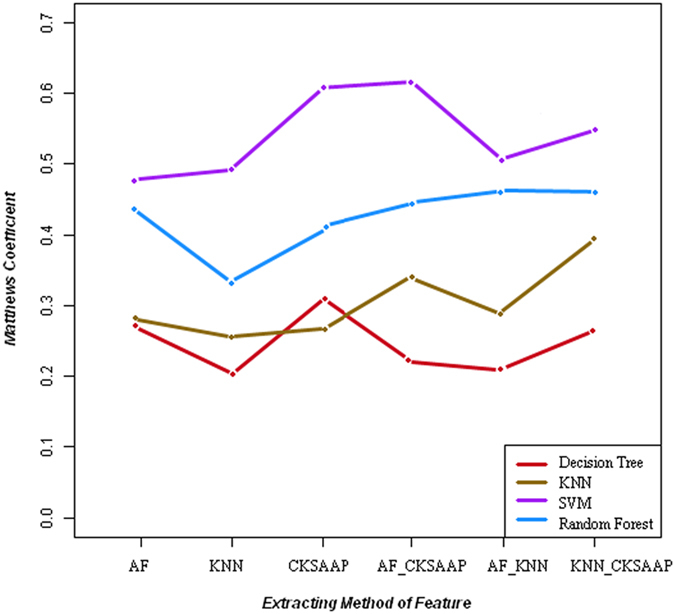
MCC of predicting performance of different classification algorithms with different encoding schemes.

The performance of the classifiers with the six different encoding schemes were firstly compared. The best results of a DT classifier were for CKSAAP, with an ACC of 71.14% and MCC of 0.314 (Fig. 3). The best results of a KNN classifier were for CKSAAP-KNN, with an ACC of 73.71% and MCC of 0.402 (Fig. 3). The best results of a RF classifier were for AF-KNN, with an ACC of 75.1% and MCC of 0.458 (Fig. 3). The best results of a SVM classifier were for AF-CKSAAP, with an ACC of 80.90% and MCC of 0.617 (Fig. 3).

We then compared classifying ability of the 4 different classifiers on phosphorylation sites of proteins in rice. As shown in Fig. 3, SVM performed best on the phosphorylation sites among the 4 classifiers with any of the 6 encoding schemes. The SVM with AF-CKSAAP, CKSAAP and CKSAAP-KNN lay in the top 3 predicting models.

### Performance of the top three predicting models on different phospho-amio acids

We used different phospho-amino acids datasets with different ratio of (+) sites to (−) sites to detect the performance of the top three predicting models (SVM with AF-CKSAAP, SVM with CKSAAP and SVM with CKSAAP-KNN). For phospho-serine, phospho-threonine or phospho-tyrosine sites, SVM with AF-CKSAAP out-performed SVM with CKSAAP or CKSAAP-KNN, even though the ratio of (+) sites to (−) sites in the dataset was unbalanced (Table 2).

Blom *et al.* (2004) suggested that a real non-phosphorylation site had to be solvent-inaccessible^4^. After discarding the predicting solvent-accessible non-phosphorylation sites from Table S2 and composing a new negative dataset (Table S3), we used the new training dataset which was extracted from Table S3 and its balancing positive dataset to re-train the top three predicting models. Table 3 also indicated that the overall performance of SVM with AF-CKSAAP was better than that of SVM with CKSAAP or CKSAAP-KNN.

### Assessment of the predictor, Rice_Phospho 1.0, with the newly existing predictors

**Figure 4:**
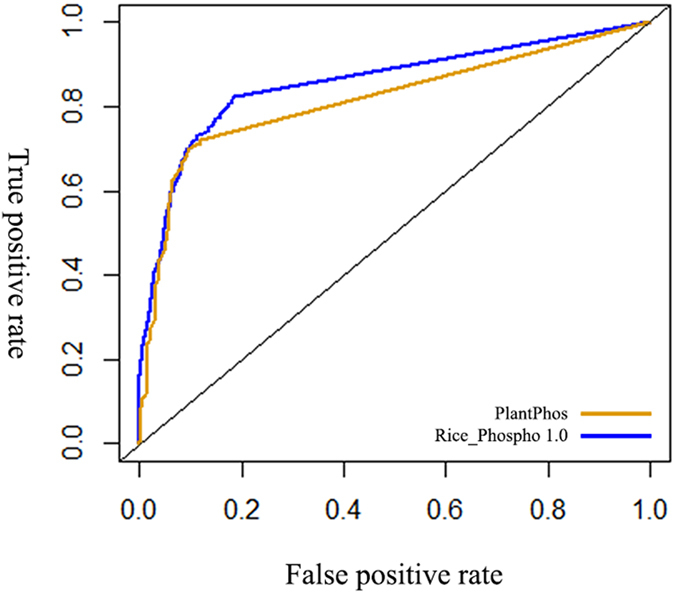
ROC curves of predicting performance of Rice_Phospho 1.0 and PlantPhos.

We used SVM with AF-CKSAAP to develop a new rice-specific predictor, Rice_Phospho 10. We applied the independent test dataset to compare the predicting performance of Rice_phospho 1.0 with the newly existing predictors, including Scansite, Musite, PlantPhos and PhosphoRice. The MCC of the prediction performance of Rice_phosphos 1.0 in comparison to Scansite, Musite and PhosphoRice were shown in Table 4. Rice_Phospho 1.0 had higher MCC value than the existing predictors, indicating that the performance of Rice_Phospho 1.0 was significantly better than that of Scansite, Musite and PhosphoRice. The Area Under ROC Curve (AUC) of Rice_Phospho1.0 was higher than that of PlantPhos (Fig. 4), implying that Rice_Phospho 1.0 also outperformed PlantPhos.

### Construction online predictor, Rice_Phospho 1.0

**Figure 5:**
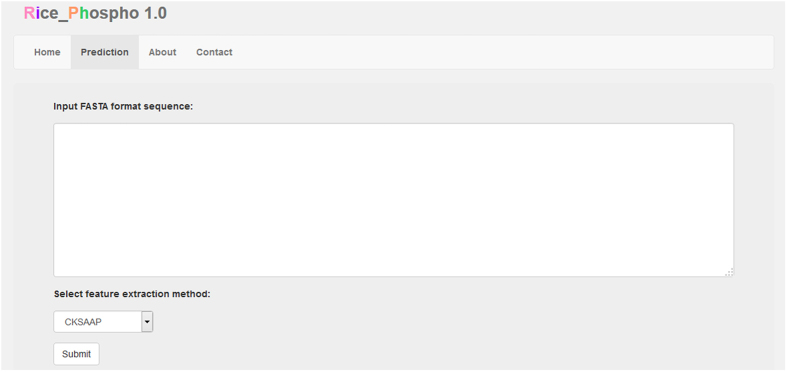
Interface of the online predictor, Rice_Phospho 1.0, on rice protein phosphorylation sites.

We constructed the online tool, Rice_Phospho 1.0, which was a specific SVM predictor on the protein phosphorylation sites in rice (*Oryza sativa* L.). The potential phosphorylation sites are retrieved after the user uploads a protein sequence in FASTA format into the text area and selects one of the encoding schemes (Fig. 5). Rice_Phospho 1.0 is accessible via http://bioinformatics.fafu.edu.cn/rice_phospho1.0.

## Discussion

Our analysis indicates that CKSAAP encoding can extract the character around the phoshorylation sites more concisely than AF and KNN. Generally, AF and KNN methods select the position-specific feature of a sequence fragment, while the CKSAAP encoding pays attention to the co-location of amino acid pairs at different positions surrounding phosphorylation sites^22^. The sequence character extracted by CKSAAP can also reflect the composition of short linear motifs, which have been widely reported to be involved in many biological processes such as the communication of protein-protein interactions^23^. The results in this paper imply that short linear motifs maybe more important than position-specific patterns in recognizing protein phosphorylated substrates. CKSAAP encoding has been reported to predict the structural property of a sequence fragment^24^ and PTM sites, including ubiquitylated sites^22^ and mucin-type O-glycosylated sites^25^. Therefore, we might also expect a better performance of the CKSAAP encoding in the prediction of protein phosphorylated sites.

More importantly, the reasonably good performance of SVM with AF-CKSAAP reflected that the combining encoding schemes, AF and CKSAAP, can effectively capture the information of enriched/depleted residue pairs around phosphorylation sites in rice. The AF encoding scheme clearly characterizes amino acids in different positions surrounding a potential phosphorylated site, but it is weak in reflecting the coupling effect of amino acid pairs at different positions. On the other hand, the CKSAAP has the ability to detect the relationship between amino acid pairs at different positions, but it cannot capture the position specific amino acid information^22^. Therefore, the complementary capability of AF to CKSAAP results in a better performance for AF-CKSAAP in extracting the sequence character surrounding a potential phosphorylated site when compared with the individual encoding scheme. Meanwhile, in terms of different phospho-amino acid sites, SVM with AF-CKSAAP performed better than others, even though the ratio of (+) sites to (−) sites in the test dataset was unbalanced. This is because the accuracy of the predictors may be overestimated when the ratio of (+) sites to (−) sites in the training dataset was optimized^1^.

We used the model of SVM with AF-CKSAAP to develop an online tool, Rice_Phospho 1.0, which was a specific predictor on the phosphorylation sites in rice. To verify the performance of Rice_Phospho 1.0, one experimentally identified phosphorylated protein in rice which did not appear in the training dataset was used as a query sequence. A α-tubulin isoform (LOC_Os03g51600.1) was experimentally identified to be phosphorylated at Thr349 site by a comprehensive mutagenesis method^26^. Rice_Phospho 1.0 successfully predicted the experimentally identified pThr at the position 349. Moreover, three more sites, including Ser216, Tyr432 and Ser439, were predicted as novel phophorylated sites.

In summary, we have benchmarked the combination of several encoding schemes and classification routines to establish their relative effectiveness. This led to the choice of a SVM with AF-CKSAAP encoding scheme which we have incorporated into the development of an effective rice specific protein phosphorylation site predictor, Rice_phospho 1.0 (http://bioinformatics.fafu.edu.cn/rice_phospho1.0). Rice_Phospho 1.0 provides state of the art levels of reliability in predicting protein phosphorylation sites in rice and will be a useful tool to the community.

## Methods

### Preprocessing of dataset

We collected rice phosphorylation sites from the recent literature of Nakagami *et al.*^21^. We also used the feature table of Swiss-Prot database, from which records annotated as ‘predicted’ or ‘similarity’ were excluded. After removing the redundant phosphorylation sites, the number of serine (S), threonine (T) and tyrosine (Y) substrates were 4220, 605 and 141 respectively, and these phosphorylation sites were involved in 2162 proteins^1^.

The 25-mer sequences (−12 to +12) surrounding the phosphorylation sites were extracted from the protein sequences^1^. Because all of these phosphorylation sites were experimentally verified, they were regarded as (+) sites and compiled within a positive dataset (Table S1). The Ser, Thr and Tyr residues that were not annotated as phosphorylation sites within the dataset were regarded as (−) sites (*i.e.*, non-phosphorylation sites), and the 25-mer sequences surrounding them were extracted and compiled within a negative dataset (Table S2). We extracted one-third of the data from each of these two datasets to compose an independent test dataset. We used the remainder of the data in the two datasets to construct a training dataset. The phosphorylation and non-phosphorylation sites were randomly chosen from the training dataset to compile a different ratio of (+) sites to (−) sites dataset during the cross-validation processes.

Because the residues buried in the core of a protein would not be accessible to any kinases^4^, the NetSurfP program^27^ was used to predict the surface accessibility of each non-phosphorylated site in Table S2. The solvent-inaccessible non-phosphorylation sites were compiled in Table S3. We randomly selected one third of the data to compose another independent test dataset, and re-trained a Support Vector Machine (SVM) by using the Composition of K-Spaced Amino Acid Pairs (CKSAAP), Amino acid occurrence frequency combined with CKSAAP (AF-CKSAAP), CKSAAP combined with K-Nearest Neighbor (CKSAAP-KNN) as feature selection methods.

A standard 10-fold cross validation was used to train the classifiers. We calculated the Sensitivity (Sn), Specificity (Sp), Accuracy (ACC) and the Matthew’s Correlation Coefficient (MCC) of each predictor^1^. The dataset was randomly partitioned into 10 subsets, including one testing subset and nine training subsets. Each predictor was trained by shifting the test subset stepwise so that all data is used for training and testing.

### Encoding schemes and feature selection

#### K-Nearest Neighbor (KNN)

The KNN method is used to classify the samples based on their distances. For each sequence, the distances between all the sequences of positive datasets or negative datasets were measured using the formula introduced by Gao *et al.* (2009)^28^.


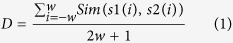


where two protein sequences s1 = {s1(−w), s1(−w + 1),…, s1(w)} and s2 = {s2(−w), s2(−w + 1),…, s2(w)}, *w* = 12, *sim*—amino acid similarity matrix—is derived from the normalized BLOSUM62.

The K nearest distances were selected, and the average distance among them was calculated. This process was repeated for different values of K (0.1%, 0.2%, 0.5%, 1%, 2%, 5% and 10% of the positive datasets or negative datasets). The ratios of the average distances between positive datasets and negative datasets were extracted as the feature values^28^.

#### Composition of K-Spaced Amino Acid Pairs (CKSAAP)

CKSAAP has been successfully used to represent sequence fragments^22^. A sequence fragment may contain 400 types (AxA, AxC, AxD, …, OxO) of K-spaced amino acid pairs (i.e. the pairs separated by K other amino acids). The flowchart and the calculation used for the CKSAAP feature selection approach are shown in Fig. S1. The value of N_AA_ is the composition of the corresponding amino acid pairs in the sequence fragment, while N_total_ represent the total composition of amino acid pairs in the sequence fragment. For instance, if there are n AxA pairs in the sequence fragment, the value of corresponding component of N_AA_ is n (Fig. S1).

When the value of K is increased, the prediction accuracy and sensitivity increases, but so does the computational complexity and the time required for training the models^22^,^29^. In this paper, we considered the CKSAAP encoding scheme with K = 0, 1, 2, 3, 4 and 5, meaning the total dimension of the 6-spaced feature vector is 2400.

#### Amino acid occurrence frequency (AF)

The frequency of one amino acid in each sequence fragment was calculated by the following equation:


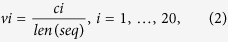


where *c*_*i*_ and *len* (*seq*) denote the number of instances of amino acid i in the sequence fragment and the length of the sequence fragment, respectively. *v*_i_ illustrates the frequency of the amino acids in the sequence.

### Combined encoding schemes

We combined the three sole encoding schemes (KNN, CKSAAP and AF) to construct three bi-encoding schemes. Because of the high dimensionality of the CKSAAP, relief-F was used to decrease the total dimension for the combined methods. Relief-F is the extension to the original Relief algorithm, which is able to deal with noisy and multi-class problems rather than two-class problem^30^. Relif-F was run in Waikato Environment for Knowledge Analysis (WEKA) to decrease the dimension of the combining encoding schemes^31^, leading to total dimensions for AF-KNN, AF-CKSAAP and CKSAAP-KNN of 27, 952 and 939, respectively.

### Classification Algorithms

#### Support Vector Machine (SVM)

A SVM is a supervised learning algorithm for two-group classification problems, whose goal is to find a rule that best maps each member of the training set to the correct classification^25^. Briefly, SVM constructs a hyperplane that separates two different groups of feature vectors in the training set with a maximum margin. The orientation of a test sample relative to the hyperplane gives the predicted score, and hence the predicted class can be derived^32^. Because of its solid mathematical foundation in statistics theory, and the ability of overcoming over-fitting, SVMs are popular and have been used to predict protein PTMs sites^28^, protein localization^33^ and protein-protein interaction^34^. In this paper, LibSVM in WEKA with Radial Basis Kernels (RBF) used with K (x_i_, y_i_) = exp(−γ||x_i_ − y_i_||^2^)^29^.

#### Random Forest (RF)

The RF is an ensemble of unpruned decision trees^35^ which have already been used to predict protein-protein interactions^36^,^37^ and long disordered regions in proteins^38^. In RF, the number of trees in the forest is adjustable, and each tree is grown to full length using a subset of the training dataset. To classify an instance with an unknown class label, each tree casts a unit classification vote. The forest selects the classification having the most votes over all the trees in the forest. Therefore, there are two key parameters in RF. One is the number of the trees, M, the other is the number of features selected randomly, m. In this paper, we selected the optimal value of M = 100, and determined m based on the result of a preliminary evaluation.

#### Decision Tree (DT)

Decision trees are an attractive predictive modeling procedure because of their easy interpretation by non-statisticians. In DT algorithms, the classification tree analysis generates groups of individuals on the basis of a selected criterion, the Gini index, for splitting a group into two to maximize the probability of a single outcome, namely substantial renal deterioration^39^. The recursive process of partitioning data continues until the Gini index indicates that the tree fits, without overfitting, the information contained in the dataset. It can provide a practical model for dichotomous outcomes if the validity of the obtained model is proved sufficient. The missing values were replaced with values minimizing the impurity of the nodes, median values for continuous variables and most frequent categories for categorical variables, or distribution-based estimates. The decision trees and random forests were implemented using R platform with package e1071.

### Performance Assessment

#### Predictors comparison

The current rice-specific phosphorylation sites predictor, PhosphoRice, two non-organism specific predictors, Musite and Scansite, and one plant-specific predictor, PlantPhos, were compared with the new predictor in this paper. In Musite online prediction, General phospho-serine/threonine and General phospho-tyrosine (Green plants) were selected and 25mer was input to the predictor with default settings. In Scansite, the setting of medium stringency level was selected and resulted in the production of Scansite_medium predictor. In PlantPhos, phosphorylation sites will be predicted with the score over −3.0 (HMMER bit score).

### Evaluation

Sn, Sp, ACC and MCC were employed to evaluate the performance of the different predictors.


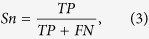



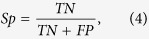



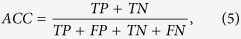



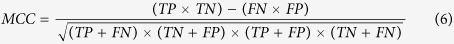


where *TP*, *FP*, *FN*, and *TN* denote true positives, false positives, false negatives, and true negatives. *Sn* and *Sp* illustrate the correct prediction ratios of positive and negative datasets, respectively. Because MCC is much less susceptible to the ratio of positive samples and negative samples in the dataset, it is the most widely used prediction measure for two-class prediction programs^1^.

### Statistics

We used SPSS 16.0 to create receiver operating characteristic (ROC) curves to measure the performance of different predictors. For each possible threshold, the sensitivity and specificity were evaluated, and the ROC curves [sensitivity versus (1-specificity) curve] were used to compare the predictive performance of different classifiers with different encoding schemes.

## Additional Information

**How to cite this article**: Lin, S. *et al.* Rice_Phospho 1.0: a new rice-specific SVM predictor for protein phosphorylation sites. *Sci. Rep.* **5**, 11940; doi: 10.1038/srep11940 (2015).

## Supplementary Material

Supplementary Information

## Figures and Tables

**Table 1:** Performance of 3 sole encoding schemes on different size of dataset.

Method	(+) sites	(−) sites	Ratio	Sn (%)	Sp (%)	ACC (%)	MCC
AF	112	127	0.88:1	67.20	66.13	69.51	0.403
	365	370	0.99:1	72.00	73.07	75.11	0.461
	853	937	0.91:1	68.31	69.43	72.30	0.408
	1530	1630	0.94:1	69.37	68.21	70.15	0.391
	2107	2018	1.04:1	75.33	76.86	74.94	0.477
KNN	112	127	0.88:1	57.00	52.3	58.29	0.237
	365	370	0.99:1	60.14	58.12	59.50	0.281
	853	937	0.91:1	68.22	63.10	67.20	0.306
	1530	1630	0.94:1	68.73	65.16	69.75	0.362
	2107	2018	1.04:1	75.35	71.03	72.39	0.407
CKSAAP	112	127	0.88:1	82.77	80.30	83.37	0.633
	365	370	0.99:1	82.27	80.63	82.84	0.617
	853	937	0.91:1	81.02	80.12	81.27	0.623
	1530	1630	0.94:1	77.84	79.70	80.56	0.612
	2107	2018	1.04:1	80.02	79.33	80.41	0.605

**Table 2:** Predicting performance of SVM with 3 different encoding schemes on different phospho-amino acid sites.

Methods	Phospho-amino acids	Ratio	Sn (%)	Sp (%)	ACC (%)	MCC
CKSAAP	Serine	1:0.7	79.84	80.41	80.36	0.617
		1:1	80.32	80.55	80.51	0.619
		0.7:1	79.91	80.31	80.16	0.613
	Threonine	1:0.34	79.89	80.11	79.95	0.597
		1:1	79.34	79.62	78.79	0.583
		0.34:1	78.42	78.86	78.37	0.589
	Tyrosine	1:0.17	84.17	83.36	84.00	0.638
		1:1	77.25	78.71	78.03	0.573
		0.17:1	74.61	73.83	73.02	0.532
AF- CKSAAP	Serine	1:0.7	81.22	79.20	81.15	0.623
		1:1	81.87	81.23	82.14	0.635
		0.7:1	83.22	83.14	84.71	0.642
	Threonine	1:0.34	78.57	78.27	79.52	0.601
		1:1	76.28	77.31	77.20	0.593
		0.34:1	78.19	77.22	78.05	0.591
	Tyrosine	1:0.17	80.19	79.75	80.72	0.623
		1:1	78.35	77.55	79.12	0.597
		0.17:1	77.75	75.38	78.04	0.593
CKSAAP -KNN	Serine	1:0.7	77.14	75.43	76.31	0.542
		1:1	76.18	74.83	75.12	0.526
		0.7:1	80.86	80.17	81.14	0.624
	Threonine	1:0.34	71.77	71.19	72.37	0.497
		1:1	72.46	72.81	73.34	0.509
		0.34:1	75.53	73.11	74.27	0.520
	Tyrosine	1:0.17	68.91	69.71	69.53	0.418
		1:1	69.92	70.24	70.11	0.433
		0.17:1	72.86	73.42	70.34	0.465

**Table 3:** Predicting performance of the three top SVM models trained by the negative dataset in Table S3 and its balancing positive dataset.

Models	Sn (%)	Sp (%)	ACC (%)	MCC	AUC
CKSAAP	79.47	82.83	81.15	0.623	0.839
AF-CKSAAP	82.84	81.59	82.04	0.641	0.858
CKSAAP-KNN	77.46	79.86	78.63	0.579	0.820

AUC is the area under ROC curve.

**Table 4:** Predicting performance of SVM models and newly developed predictors.

Tools	Sn (%)	Sp (%)	ACC (%)	MCC
Musite	55.18	81.91	73.32	0.368
Scansite	75.18	53.23	59.90	0.285
PhosphoRice	70.25	74.40	75.32	0.462
Rice_Phospho 1.0	79.93	81.21	80.33	0.616
