# Supplementary material for: Rice_Phospho 1.0: a new rice-specific SVM predictor for protein phosphorylation sites
Source: Sci Rep. 2015 Jul 7;5:11940. doi: 10.1038/srep11940 (PMC4493637; doi:10.1038/srep11940)
Supplement: Supplementary Information [file srep11940-s1.pdf]

Rice_Phospho 1.0: a new rice-specific SVM predictor on protein phosphorylation sites

Shoukai Lin[1#], Qi Song[1#], Huan Tao[1#], Wei Wang[1], Weifeng Wan[1], Jian Huang[1], Chaoqun Xu[1], Vivien Chebii[1], Justine Kitony[1], Shufu Que[1], Andrew Harrison[2] & Huaqin He[1*]

[1]College of Life Sciences, Fujian Agriculture and Forestry University, Fuzhou 350002, China.

[2]Department of Mathematical Sciences, University of Essex, Wivenhoe Park, Colchester, CO4 3SQ, UK

*To whom correspondence may be addressed. E-mail: hehq3@fafu.edu.cn

# These authors contributed equally to this work.

Subject Category: Computational biology and bioinformatics

Type: research article

Table S1. The 25-mer sequences (-12 to +12) surrounding phosphorylation sites were extracted from phospho-protein sequences and compiled into the positive dataset.

No.	Protein Name	Sequence	Phospho-amino acid	Position			No.	Protein Name	Sequence	Phospho-amino Acid	Position
1	LOC Os01g01150.1	QHKSKKSEEGSKSRKDDCLLDDTLS	S	863			2	LOC Os01g01150.1	DPDQDDDLHRPSSPAQASRDAYSDA	S	57
4	LOC Os01g01150.1	PSSPAQASRDAYSDADDDDDDRPHA	S	67			5	LOC Os01g01150.1	VSSVAHKRSSSASEDEMLNSNSKRS	S	733
7	LOC Os01g01150.1	KLDVSSVAHKRSSSASEDEMLNSNS	S	730			8	LOC Os01g01150.1	SDSRSPKRHREESLSPSKSRRSARA	S	629
10	LOC Os01g01150.1	SRKDDCLLDDTLSSDRRKVQSEDSP	S	875			11	LOC Os01g01150.1	SRSPIKYRRSRRSRSYSPPVRHTRG	S	541
13	LOC Os01g01510.1	RGKNAYYDAGEQSGDDEDDYEEARR	S	140			14	LOC Os01g01510.1	LSMQDFGLEDGESDEEDRAIKASNH	S	173
16	LOC Os01g01510.1	KEQKYSPNPVIESLEEETEGDGKRK	S	563			17	LOC Os01g01510.1	ARRIQKEEESKLSMQDFGLEDGESD	S	162
19	LOC Os01g01510.1	PTVKPPVMAASASDDDEIDAFHKHR	S	26			20	LOC Os01g01689.1	KESEDIDSCLDDSQEACEGNKDAAR	S	2777
22	LOC Os01g01800.1	QTPYPHIQSPMSSPVQVRGDWGVAG	S	401			23	LOC Os01g01800.1	TQTPYPHIQSPMSSPVQVRGDWGVA	S	400
25	LOC Os01g01960.1	NTPKVEREEGELSPNGDFEEDNFAP	S	873			26	LOC Os01g01960.1	DMDHDDQDAKAESEGEAEGTTETHD	S	987
28	LOC Os01g03500.1	AKENANLIDADDSDELRSICSESDE	S	131			29	LOC Os01g03520.1	LSVAFGFVTRLASELFARESGDDID	S	727
31	LOC Os01g03520.1	NVSNGVDVDSQSSLPDNKVRVLWID	S	105			32	LOC Os01g03520.1	DNVSNGVDVDSQSSLPDNKVRVLWI	S	104
34	LOC Os01g03760.1	AARIAAATASAYSDDDDDSDEAQAT	S	102			35	LOC Os01g03760.1	ATASAYSDDDDDSDEAQATRPEGEV	S	108
37	LOC Os01g04100.1	NRAGDAIAAARRSPEQLDGVKERLL	S	70			38	LOC Os01g04160.1	SSMPDDFLSRSDSLPLERSSRLQPV	S	229
40	LOC Os01g04330.1	FTRFDADGDGRISPSELAAVTRAIA	S	44			41	LOC Os01g04650.1	ARHPHDVQSVPDSPMIDKNSSFGST	S	268
43	LOC Os01g04650.1	ISGSLDGIPRGISTDSASVNCLLGL	S	207			44	LOC Os01g04650.1	DGIPRGISTDSASVNCLLGLEDDSS	S	212
46	LOC Os01g04650.1	WFVDALNSAISGSLDGIPRGISTDS	S	198			47	LOC Os01g04650.1	SVPDSPMIDKNSSFGSTSSAPSLSN	S	276
49	LOC Os01g04650.1	PMIDKNSSFGSTSSAPSLSNLPPIR	S	281			50	LOC Os01g04650.1	RGGDSWDEPFPSSAAAAAAAAGGGR	S	43
52	LOC Os01g04650.1	SISPSDASSRVFSDDDKSDHGGGGG	S	360			53	LOC Os01g04650.1	DASSRVFSDDDKSDHGGGGGRKPQP	S	365
55	LOC Os01g04720.1	SFDVSAAATLVLSFRSPWPEPASRY	S	137			56	LOC Os01g05010.1	RIIDAEIKFAEESDDHDRVEEIPDN	S	81
58	LOC Os01g05420.1	IQELWDTIAGGDSEDDQEGVRTLDD	S	194			59	LOC Os01g05420.1	EDDLGDDWNRGRSPTPVHGDDGAGS	S	47
61	LOC Os01g05420.1	PADRYGSDNDGHSPRHYPQAEEGEE	S	229			62	LOC Os01g05820.1	LSSGGDDRSRVVSGGGGGVRLSPHE	S	61
64	LOC Os01g06270.1	QVPSTPKAETVVSPKSPAKPDQPLD	S	485			65	LOC Os01g06290.1	ERRDMSTAANGRSPSPRDYEDNGNH	S	302
67	LOC Os01g07110.1	SPMFQTGSGKMVSLSKGSIQKARAV	S	178			68	LOC Os01g07260.1	ESSPGPDLRRIASCFTSEQREKRKR	S	155
70	LOC Os01g07520.1	DRLRRRHSSSNGSADDVLPSYDFQP	S	59			71	LOC Os01g07880.1	APNNLKEGGGVESDEEIRRVPEMGG	S	44
73	LOC Os01g07890.1	QRSSQTFRRSGSSGLVWDERLMLDG	S	17			74	LOC Os01g08260.1	GITNATSVQPVISIERFVSYRERAA	S	413
76	LOC Os01g08410.1	SQEPADGTEETASAEPDEDTTSVDV	S	265			77	LOC Os01g08420.1	NGAVATRLSSNKSISVPAGFDQRTV	S	711
79	LOC Os01g08430.1	ALDESRVIYDMDSDDEVWVSRWRKL	S	1186			80	LOC Os01g08560.1	KMETDDAPNEAASGTDVNMQEAKAP	S	527
82	LOC Os01g09280.1	VKQKEPEFARHLSDLQLRKHEESEF	S	221			83	LOC Os01g09550.1	QQRRRDSGSGSCSSTRDHEVSATSY	S	301
85	LOC Os01g09570.1	HHLHNTQLLEGESSPVKDSSKCNGT	S	515			86	LOC Os01g09570.1	HLHNTQLLEGESSPVKDSSKCNGTA	S	516
88	LOC Os01g09620.1	GWSYPSSSAVYGSPKAATGLYSLPT	S	294			89	LOC Os01g09620.1	SFRQLRLNKVKSSPSGGWSYPSSSA	S	278
91	LOC Os01g09790.1	GKGRKWKLWRSSSGDHGSLWRGSRG	S	49			92	LOC Os01g09850.1	RCDCGTLFSRRDSFITHRAFCDALA	S	191
94	LOC Os01g10610.1	VRAWEGERIHEDSGSDDLELTLGSS	S	347			95	LOC Os01g10610.1	AWEGERIHEDSGSDDLELTLGSSRT	S	349
97	LOC Os01g10690.1	QICAKEEANADSSDDEQAVHYKRGS	S	296			98	LOC Os01g10690.1	AKGQQQGLPGSSSPKKGTSNKAEKD	S	199
100	LOC Os01g10820.1	RASKVLEQLSGQSPVFSKARYTVRS	S	46			101	LOC Os01g10820.1	SPVFSKARYTVRSFGIRRNEKIACY	S	58
103	LOC Os01g11040.1	MDKAEHVQSKCISPEQQEIDHREME	S	388			104	LOC Os01g11040.1	SNTPLHTRLNGTSADDLGDDLKQSS	S	330
106	LOC Os01g11330.1	AGVTIKRAKQNCSAPKKRTMGAVLG	S	556			107	LOC Os01g11580.1	NTADDNERYVGSSSPVHLEDQKGEN	S	301
109	LOC Os01g11580.1	EIDNSKVLERSDSTVHAAGMEATPK	S	126			110	LOC Os01g11920.1	ANYGHGSIVRSRSGGVVVAEEDAVV	S	179
112	LOC Os01g11960.1	RGLFELETDEGCSLEKSVNDQSIPD	S	271			113	LOC Os01g12280.1	PRRIRDLPSQPTSPERATSPAITST	S	101
115	LOC Os01g12480.1	LSDMYRKPAAEKSDDTSDDDEEPDI	S	64			116	LOC Os01g12480.1	YRKPAAEKSDDTSDDDEEPDIDIGK	S	68
118	LOC Os01g12650.1	KLHIGGDGSSSDSDADERKQPKPSA	S	36			119	LOC Os01g12650.1	KIADKLHIGGDGSSSDSDADERKQP	S	32
121	LOC Os01g12660.1	STSSFPKLKRNASTSSDMSSLASQG	S	216			122	LOC Os01g12660.1	SSFPKLKRNASTSSDMSSLASQGPP	S	218
124	LOC Os01g12660.1	PPNNPASLRRASSWTFDEKILVQAV	S	241			125	LOC Os01g12660.1	GSSTDSSFKRSISETTLEKVSGLLG	S	161
127	LOC Os01g12780.1	QMAAVDDGKLRASSRSSTQPPPRRR	S	111			128	LOC Os01g12780.1	MAAVDDGKLRASSRSSTQPPPRRRR	S	112
130	LOC Os01g13160.1	INKNELSDIFGSSPEPFFQERVEDK	S	511			131	LOC Os01g13270.1	LVMEYCSGGNLHSLRQRQLNKHFNE	S	226
133	LOC Os01g13530.1	IISTQTKETRAGSPIPNSNPLARSA	S	242			134	LOC Os01g13530.1	RQHMSRKMHKSQSISTPVGRQSTMR	S	184
136	LOC Os01g14050.1	AGEPGRSLTSILSRKISRKKPEDKL	S	606			137	LOC Os01g14440.1	SSTGNKDQERGDSPDAPSTAAAWLP	S	245
139	LOC Os01g14514.1	PSPRGGSVHARCSPFRNLVEMSGSM	S	119			140	LOC Os01g14690.1	LISKDHERAYFDSADWALGKSGQQG	S	65
142	LOC Os01g14950.1	QLEATTQFRKLLSIERSPPIEEVIQ	S	101			143	LOC Os01g14950.1	QAVWALGNVAGDSPKCRDLVLANGA	S	189
145	LOC Os01g15020.1	ENGVPNGRTSTSSATSNPAADQIQR	S	1117			146	LOC Os01g15020.1	DHTCTPPNGARASPVSVPLAAVPKA	S	213
148	LOC Os01g15039.1	VAQDGSGRWRTVSEAVARAPSHSRR	S	319			149	LOC Os01g15260.1	SSDVDRKPAAAGSPPPGAAAAAGHK	S	34
151	LOC Os01g15600.1	DDVGAGFIAGHGSQSASGPEVDVER	S	203			152	LOC Os01g15600.1	AGFIAGHGSQSASGPEVDVERLVDL	S	207
154	LOC Os01g16020.1	TVAAKPEIKRLQSGIRARAAGKVAG	S	269			155	LOC Os01g16030.1	NAAEITDKLGLHSLRQRHWYIQSTC	S	147
157	LOC Os01g16110.1	HYGGVSESPPSNSIGYFYGSTPENH	S	700			158	LOC Os01g16110.1	SPPSNSIGYFYGSTPENHSYRSSRL	S	707
160	LOC Os01g16110.1	LSSSVTSLNKGLSNISIDNKPKSIS	S	476			161	LOC Os01g16110.1	KYWAFHHFRNQDSSPIIKHPELERL	S	875
163	LOC Os01g16110.1	PHGIPTGSSPVGSVPKSFPPFQHPS	S	735			164	LOC Os01g16110.1	TPENHSYRSSRLSSSPHGIPTGSSP	S	720
166	LOC Os01g16110.1	PENHSYRSSRLSSSPHGIPTGSSPV	S	721			167	LOC Os01g16110.1	ENHSYRSSRLSSSPHGIPTGSSPVG	S	722
169	LOC Os01g16330.1	SSRPSRSLVGNMSSRTSRAVQNNQP	S	229			170	LOC Os01g16330.1	SRPSRSLVGNMSSRTSRAVQNNQPS	S	230
172	LOC Os01g16390.1	LRYKLEPPAAASSSGEYAPRCYDDL	S	154			173	LOC Os01g16390.1	RYKLEPPAAASSSGEYAPRCYDDLD	S	155
175	LOC Os01g16870.1	AQVSQFIKFDEMSETSSSHGGHTSA	S	870			176	LOC Os01g16870.1	SQFIKFDEMSETSSSHGGHTSAGSA	S	873
178	LOC Os01g16870.1	FIKFDEMSETSSSHGGHTSAGSAPV	S	875			179	LOC Os01g16870.1	MSETSSSHGGHTSAGSAPVPELPRL	S	881
181	LOC Os01g17320.1	PPEADKKDLDLTSDESSDEDTIDAI	S	112			182	LOC Os01g17320.1	SGDKQDELEEGFSDLEVPPEADKKD	S	95
184	LOC Os01g18890.1	AHSFGRSHCSAFSFRLYPQVAPDMD	S	115			185	LOC Os01g19480.1	NGRYNISMCKVLSNHWSQERRKFLR	S	328
187	LOC Os01g19760.1	SKDLPRVLINTISGRWNPVNDPSAP	S	847			188	LOC Os01g19894.1	AFVQKREFRVKVSNRTTYDVKCIQG	S	845
190	LOC Os01g20940.1	SSAGSRGSSDSVSPLSRESWSNKYF	S	590			191	LOC Os01g20950.1	QVYVRDRAPDLSSLPAPRLLATHIP	S	141
193	LOC Os01g21590.1	GHHKRRRGDDVSSAGAGDDDDEDGV	S	380			194	LOC Os01g21590.1	DDEGPSAVQQLQSELAAAVAGGGDP	S	418
196	LOC Os01g21960.1	SFRYMDKDLGFQSADEGGSGTFRHN	S	143			197	LOC Os01g21960.1	KDLGFQSADEGGSGTFRHNSAHAIT	S	149
199	LOC Os01g23540.1	GNAKLAVQCDKRSLDMVEHTPSPPA	S	763			200	LOC Os01g23540.1	DKRSLDMVEHTPSPPATAELPKKVS	S	772
202	LOC Os01g23590.1	LEKEEEDYFNEDSDEEDSVRRTKHA	S	700			203	LOC Os01g23590.1	EDYFNEDSDEEDSVRRTKHAQKQDG	S	705
205	LOC Os01g23640.1	EEELSQLSIKQPSPPPVLAELERRP	S	307			206	LOC Os01g23930.1	GRRPSPPLPRRSSVDRSGPSSSRRG	S	1368
208	LOC Os01g25040.1	PTGFSANVRKLVSLKDLTISGYNAH	S	829			209	LOC Os01g25330.1	IGKWDVHDMMLSSPHLSGPEKTSSM	S	777
211	LOC Os01g25370.1	RSNPSCFTPSPSSPQHPRSLGLEQY	S	139			212	LOC Os01g25610.1	DEDAKFKLCKVRSVQFGQKGIPYLN	S	128
214	LOC Os01g26940.1	ETNRRRASSRELSPHGRQNSPSPPE	S	348			215	LOC Os01g26940.1	RSPRTHSPKELDSPRPENEVGKLAT	S	307

217	LOC Os01g26940.1	SSHRHHSPRRPWSPPANRKTGLGKP	S	603			218	LOC Os01g27730.1	DCPGVVMLKSSNSGVSVALRNCKRV	S	480
220	LOC Os01g27790.1	DDEGHVLAVEGASRAPRKGRPASDK	S	456			221	LOC Os01g27990.1	QPPPPKALSAAGSFGKKMTMDDLIA	S	103
223	LOC Os01g31580.1	LDFSHATLQRSSSTPNIPMPDPKAA	S	115			224	LOC Os01g31629.1	SSKSITVEGQRKSLEETQQLLDQRK	S	120
226	LOC Os01g32130.1	LLGEGRLKLMLNSDGELEEEQETHA	S	195			227	LOC Os01g32720.1	FHTNIDDISRKFSPGTDFEVNRQHF	S	848
229	LOC Os01g32870.1	TMVNSVGAGSSSSRYPTESPENGNI	S	368			230	LOC Os01g32964.1	LRCWPGGLCLSRSIGDIDVGEFIVP	S	121
232	LOC Os01g33000.1	GRVKHLTQEGFSSDDGESGDLHGRL	S	202			233	LOC Os01g33090.1	FGLGRLANIAGGSPFTQSDHAGIEK	S	562
235	LOC Os01g33370.1	SSDANNYRNEIPSSTRVIRHRRKGI	S	198			236	LOC Os01g33490.1	PPAASPPVVSSPSHLVTAASLLRLP	S	90
238	LOC Os01g34030.1	RQTQLAEIQALDSVMRETEEEQHAA	S	424			239	LOC Os01g34330.1	GRRRRFAQLATTSDDDDDDAPAADA	S	14
241	LOC Os01g34330.1	LVKKTIDRIGQLSDIEPADAPGDNN	S	310			242	LOC Os01g34410.1	LHPGAQQPVNIDSGDEEAPNVRTEK	S	139
244	LOC Os01g34780.1	KDEMDIDAVDADSHGSKDKKREKDK	S	350			245	LOC Os01g34780.1	MDIDAVDADSHGSKDKKREKDKEKK	S	353
247	LOC Os01g35050.1	NTPVPSKYNGSESPSLAEIVNDQRL	S	756			248	LOC Os01g35050.1	PVFKGDEDDEKFSISDEPEAEQVLV	S	723
250	LOC Os01g35050.1	RRNTPVPSKYNGSESPSLAEIVNDQ	S	754			251	LOC Os01g36790.1	KKKIFRNKKRSKSFIDIYGDGVPVR	S	308
253	LOC Os01g36860.1	SRSPGASRRHERSATGSGSALPDSG	S	656			254	LOC Os01g36860.1	GASRRHERSATGSGSALPDSGHGER	S	660
256	LOC Os01g36860.1	RSPTPQDKSGPYSPAYNGKTSRSVS	S	720			257	LOC Os01g36860.1	SGPYSPAYNGKTSRSVSPGNQVEGN	S	728
259	LOC Os01g36930.1	KASSSKENEGSSSNAGESSSMDIDK	S	381			260	LOC Os01g36940.1	KKHQRQRQQRPLSPGCCGCARCGCR	S	47
262	LOC Os01g37790.1	VIPPEQKSGLEPSSPHVVKPIKNAR	S	586			263	LOC Os01g37790.1	IPPEQKSGLEPSSPHVVKPIKNART	S	587
265	LOC Os01g37790.1	SATSPRKMRRLSSFASSRRDGASRK	S	725			266	LOC Os01g37825.1	DFPVPSHVEPRFSCFVESEAAGSAV	S	294
268	LOC Os01g37825.1	RFSCFVESEAAGSAVVVSCKMPADR	S	304			269	LOC Os01g37832.1	KSISVVGGADGTSPSRMRAFINREG	S	92
271	LOC Os01g40050.1	SKKKAVKQLNVLSFGDEVEEEENEA	S	207			272	LOC Os01g40050.1	SKKSDFRELEHDSETDDYPDDENEE	S	282
274	LOC Os01g40050.1	AHRKDKELPVHRSDDDNDDDNEDHQ	S	338			275	LOC Os01g40070.1	AKMEGSFDAAAESDATARSKRSRLA	S	149
277	LOC Os01g40110.1	NLSVNSGEVVQSSNEEAETKAKSKG	S	203			278	LOC Os01g40340.1	GRPTSLHDRGRDSDDDDFRDRDYDV	S	404
280	LOC Os01g40590.1	FARNGQQPMRSLSYGPHASPYRQSP	S	474			281	LOC Os01g40590.1	QERAASLGNPIGSQSMKAQGTFARN	S	453
283	LOC Os01g40750.1	APPMGKGDIDEDSDDDGYADEETTC	S	63			284	LOC Os01g41190.1	VPSRESIANKGASPPRNDIVNEEVD	S	662
286	LOC Os01g41430.1	SKDFAARGAAELSPDADGCLRWLDA	S	271			287	LOC Os01g41550.1	PSQISRRFGRSFSYCLVDRTSSSAS	S	295
289	LOC Os01g41630.1	QLQNPTRPARSLSTLTRVVRRGAES	S	454			290	LOC Os01g41640.1	EVKNGYQSRMTHSDADLFFIDKQSG	S	418
292	LOC Os01g41880.1	NVERDERTRKSLSINEFLKPAEGER	S	315			293	LOC Os01g41880.1	QSDEVEKHKEGESNEEEEKEPEDKE	S	229
295	LOC Os01g42260.1	GLSGSLQQVQSRSPQIPGPAQSIKT	S	251			296	LOC Os01g42380.1	ASMRLGGSMRGDSGSMWRRGDDVFS	S	23
298	LOC Os01g42380.1	SMWRRGDDVFSRSSREEDDEEALRW	S	37			299	LOC Os01g42700.1	SGLSAREDGIGVSEGEEDISERALH	S	223
301	LOC Os01g42820.1	KRKPDENGAAVASSPAAGAAALGLT	S	19			302	LOC Os01g42820.1	RKPDENGAAVASSPAAGAAALGLTR	S	20
304	LOC Os01g42830.1	KSAFFEQGLNKLSFNEKANSKEAPV	S	199			305	LOC Os01g42830.1	PPPPPSPVSPTDSGIAASPFKAEFP	S	228
307	LOC Os01g43050.1	EDKHADLSKDDESDGSPESLLCKQS	S	352			308	LOC Os01g43050.1	HADLSKDDESDGSPESLLCKQSPVR	S	355
310	LOC Os01g43050.1	PSQEGKGPLRVKSFVPEQFSDLLAE	S	821			311	LOC Os01g43050.1	KRNKKQDLNRRKSLADAGLTWQAGV	S	758
313	LOC Os01g43330.1	RSIQLKTDGLLLSPSRSGDIESPKD	S	360			314	LOC Os01g43330.1	IQLKTDGLLLSPSRSGDIESPKDIL	S	362
316	LOC Os01g43330.1	PLEDTRNASDAASLSEISEEKKTEE	S	575			317	LOC Os01g43330.1	EDTRNASDAASLSEISEEKKTEEEN	S	577
319	LOC Os01g43330.1	QVEPSDARVSPCSPVLEDKLVDPLC	S	394			320	LOC Os01g43330.1	LKTDGLLLSPSRSGDIESPKDILCE	S	364
322	LOC Os01g43330.1	VDPLCSQEKDNNSEDLGMANISDVN	S	414			323	LOC Os01g43420.1	VGMKDQPGVRHLSSFSSFDNPRGRQ	S	264
325	LOC Os01g43440.1	SGPIPAALGRLSSLRSLILGDNRMQ	S	305			326	LOC Os01g43750.1	EKPKFPTLTKLFSDSLANHEGEKWV	S	149
328	LOC Os01g44310.1	ETTSGDFVGCGKSWDGGDIAELRME	S	620			329	LOC Os01g45550.1	EAALPDGLTKMGSSSTAELHPKVVD	S	421
331	LOC Os01g46070.1	NGVEEVLGLGQLSDFEKEGLENLKG	S	313			332	LOC Os01g46710.1	IVEPDQGMEEVASPTIKEPDEDIEV	S	1157
334	LOC Os01g46710.1	REQRGSADKSSGSGRLDALVERMRS	S	466			335	LOC Os01g46710.1	RSSSHRSHRRGGSVERSESEGDEAG	S	20
337	LOC Os01g46710.1	SHRRGGSVERSESEGDEAGGGAGAR	S	26			338	LOC Os01g46710.1	DAHPSPKHLSERSPTEQRFSGRLSG	S	600
340	LOC Os01g46750.1	EALESWAAANGISGDLEALCENPKA	S	577			341	LOC Os01g46840.1	IATVHPLSIRNLSGPELRNLNKGGG	S	527
343	LOC Os01g48180.1	NAKAATVEESPRSSGADEWGLENRR	S	1467			344	LOC Os01g48180.1	AKAATVEESPRSSGADEWGLENRRP	S	1468
346	LOC Os01g48180.1	AREKIRAFQNVISDSEAEKEANDAE	S	651			347	LOC Os01g48180.1	EKIRAFQNVISDSEAEKEANDAERD	S	653
349	LOC Os01g48210.1	AVEDEPATPRALSPQPAPEATLSGS	S	153			350	LOC Os01g48210.1	TEPWEPLPRQFDSDNDAVYNWHVID	S	523
352	LOC Os01g48320.1	PRGLPMMRRRLESPSRSPSPPSPTP	S	141			353	LOC Os01g48320.1	PMMRRRLESPSRSPSPPSPTPAVAL	S	145
355	LOC Os01g48320.1	PNPNLPYREDCWSDGETAALVDAWG	S	20			356	LOC Os01g48320.1	RLESPSRSPSPPSPTPAVALPLPNY	S	150
358	LOC Os01g48330.1	TSKTRRIRGRSISITPEIGDDIVRA	S	410			359	LOC Os01g48330.1	PRNRNGSTQKAMSLPSSPHEYRAQI	S	487
361	LOC Os01g48610.1	GGYGYASPVTLGSAMVDGGTLTADD	S	464			362	LOC Os01g48720.1	PYIMEDASNANDSPRSSISGAKRKH	S	226
364	LOC Os01g49190.1	NSEVSALLGRIPSAVGYQPTLATDL	S	355			365	LOC Os01g49200.1	GLDIAGLPIKKLSFNASTLRETETP	S	576
367	LOC Os01g49250.1	ERSVAADSWSVRSEYGSTLDDDQRY	S	64			368	LOC Os01g49250.1	AADSWSVRSEYGSTLDDDQRYADAA	S	68
370	LOC Os01g49350.1	VEDTGKYQAAPQSPSSPAGSAVMHA	S	247			371	LOC Os01g49350.1	TGKYQAAPQSPSSPAGSAVMHAATK	S	250
373	LOC Os01g49470.1	AGEGTLAAVMPRSPSPTASAAAGSA	S	20			374	LOC Os01g49470.1	EGTLAAVMPRSPSPTASAAAGSAAE	S	22
376	LOC Os01g49529.1	GGKDKKGPDPPLSPDTVHAQWDSRQ	S	682			377	LOC Os01g49640.1	YAKNPDLRKYINSQNSRKVAAACSV	S	86
379	LOC Os01g49890.1	EVTAELPIYLANSLNSLRLEGQKTA	S	304			380	LOC Os01g50310.1	AAVVAEKTSPNGSVAEDEVTVTAAE	S	87
382	LOC Os01g50310.1	ATVAAEKTVPDGSVAEDEVTVTAPV	S	149			383	LOC Os01g50310.1	AKSYAAVVAEKTSPNGSVAEDEVTV	S	83
385	LOC Os01g50320.1	KHHPESDEENYDSEESYKHSRKKHR	S	732			386	LOC Os01g50320.1	EAGRASPGDLNLSESDPQPSAKERE	S	675
388	LOC Os01g50470.1	FNSAGYCGTNSFSFRKSDVGFDRLN	S	170			389	LOC Os01g51010.1	GTTTKTAKSYFDSDDSEDELAQRKE	S	947
391	LOC Os01g51010.1	SSADWGQQHRNESPVNHSSSTLFSG	S	622			392	LOC Os01g51010.1	REEFNHSRERKSSVSGSNWNIEFKD	S	338
394	LOC Os01g51020.1	FSKEPNNLYNVHSYKHSGLANKKTV	S	45			395	LOC Os01g51154.1	SNIDLKDKWRNLSFSASGLGSRDKL	S	57
397	LOC Os01g51230.1	YGSHKPPLQRQRSGDLLHHNNRMAF	S	384			398	LOC Os01g51300.1	AEHISQFNEEARSPFVKKYKTIIHP	S	98
400	LOC Os01g51390.1	LPELSWFRSHTYSDDEFSSRTFLQD	S	489			401	LOC Os01g51620.1	NDEMSCAIIKIGSIIRNKERFVKRR	S	177
403	LOC Os01g51710.1	SPSPEVALKISRSLRSLNAKTGLFT	S	248			404	LOC Os01g51890.1	PFISEKALRSCLSFKSAHGDSNAFP	S	379
406	LOC Os01g52390.1	NAERDERAKKSVSINEFLKPAEGER	S	318			407	LOC Os01g53000.1	EERAGSVTSDVPSSLASDRLIVVAN	S	72
409	LOC Os01g53000.1	GTLAELDEERAGSVTSDVPSSLASD	S	65			410	LOC Os01g53000.1	GSVTSDVPSSLASDRLIVVANTLPV	S	76
412	LOC Os01g53520.1	EHKEEQSVMEKLSEKLHGDSSSSSS	S	15			413	LOC Os01g53560.1	RDHTAVGTTEESSPKKVKASPGVAE	S	110
415	LOC Os01g53680.1	DSYPNLRALRNASAMSLPDDDAAYA	S	67			416	LOC Os01g53680.1	PFSATCVRISRDSYPNLRALRNASA	S	56
418	LOC Os01g54560.1	PGGGGGGRRRSGSFGLKRMSRVMTV	S	34			419	LOC Os01g54920.1	EEKEEEEDDIEESEEEESEDESEGK	S	52
421	LOC Os01g55040.1	SQRPRRRLERGTSTVSQLFKGQEDG	S	1064			422	LOC Os01g55050.1	SGSGVPAGRGSRSFDFGADDVLCSY	S	14
424	LOC Os01g55280.1	TEPPAEEESSINSPATDVFEVVRTE	S	154			425	LOC Os01g55280.1	SSPKLADRHSPRSPLHEKKRAGTRV	S	53
427	LOC Os01g55280.1	PATDVFEVVRTESGDKENQSAAGAE	S	167			428	LOC Os01g55350.1	TLKRIRDPGFHVSPRAHLSKDIMDS	S	873
430	LOC Os01g55420.1	LGAPPRPAAAAASPSRAALPDKPWV	S	70			431	LOC Os01g55490.1	AIVDYAHDETAMSPEQEDGEINGID	S	139
433	LOC Os01g56000.1	THDSAGGAATLTSPGQNFGKAKHSY	S	522			434	LOC Os01g56100.1	PSDSEKANLHITSDDDDKEVEDWLK	S	353
436	LOC Os01g56140.1	AKVGQKRLNHLVSCDQTEVLEPKRK	S	844			437	LOC Os01g56140.1	KVNGGEITSNCLSALEEKCSKNEHD	S	876
439	LOC Os01g56140.1	QVIFKRSPEKSASHDQFVQNGVPKK	S	805			440	LOC Os01g56190.1	SDKPLNAPHFQKSFNHPDGASGHIR	S	1173

442	LOC Os01g56490.1	EHSDVTPKRSCLSNQNRNSFDKAVK	S	1095			443	LOC Os01g56800.1	ISMKVDTSEEGASDVSDNLDGSIEI	S	546
445	LOC Os01g56800.1	RAALEPLPCQPVSPKDDKCSQSRTK	S	390			446	LOC Os01g56800.1	GNLVDSEVTSISSSGRLNLADHYYD	S	1179
448	LOC Os01g56910.1	SFRRKQSQLQADSATIEPINKLINK	S	877			449	LOC Os01g57280.1	ADSDSDIRRTLLSEHSSMKKLTELM	S	945
451	LOC Os01g58550.1	ARPLKKSLQKSLSMPASLDNAAAAT	S	45			452	LOC Os01g58550.1	LDNAAAATTCAASPEKSRAADFARA	S	62
454	LOC Os01g58870.1	QKKAAQADEEAGSLLQERDSHGERK	S	344			455	LOC Os01g59670.1	LKEETAAGYPLDSDDDADKIYPEPM	S	938
457	LOC Os01g59670.1	ESSEQLDVIDAKSVDDIYAALKEHT	S	1172			458	LOC Os01g59670.1	GFEDNLLDSSLGSPWARVGSEDGVG	S	331
460	LOC Os01g59850.1	AFFDGKLGSFLYSPGRLRDQMHEDR	S	195			461	LOC Os01g59990.1	KRRRTTKKPYSRSIVGATLEVIQKK	S	84
463	LOC Os01g59990.1	YPGKGIRFIRADSQVFLFANSKCKR	S	28			464	LOC Os01g60040.1	QNIIPPMDERALSGLQARMERLKSG	S	1981
466	LOC Os01g60040.1	SPKFAPSPVHTKSINNKTDCNEDDA	S	1875			467	LOC Os01g60190.1	FGHVTFFWNGNRSGYFDETKEEYVE	S	374
469	LOC Os01g61180.1	LERTVRTLDRQISQFVTMDRLIWAD	S	108			470	LOC Os01g61370.1	FSWESSPGVPKRSSACMHMAQEIMP	S	83
472	LOC Os01g61430.1	PTQYPNTLHSAKSLEVEDPSWLVDK	S	99			473	LOC Os01g61590.1	EVAIMRHLPKSASIVSLREACEDEG	S	137
475	LOC Os01g61590.1	SRHYSRGRFNSLSMKLIKDGSVKLV	S	537			476	LOC Os01g61720.1	HADKLMERGRRHSDPPKVDPASLKD	S	484
478	LOC Os01g61814.1	RNQRWADKAYKKSHLGNEWKKPFAG	S	30			479	LOC Os01g61830.1	ELNELEASLSRTSIQIREPGEGTSS	S	354
481	LOC Os01g61860.1	SANNEEIKPSEDSDDEFYDVEKVDP	S	155			482	LOC Os01g61860.1	KNFLDSQAEYDESSGEDQDAKVSPS	S	61
484	LOC Os01g61930.1	TKYYANAITIANSPEEKKRREHRSK	S	657			485	LOC Os01g62310.1	RAGGFSDPPSPLSPPLSPASAAAAA	S	27
487	LOC Os01g62310.1	PPSPLSPPLSPASAAAAALANARWT	S	34			488	LOC Os01g62500.1	ALLRPGRFDRQISIDKPDIKGRDQI	S	487
490	LOC Os01g62650.1	ERERELNMFRSGSAPPTIEGSLNAI	S	61			491	LOC Os01g62650.1	VRINSNEKKLNCSSSFNTASSKAVE	S	333
493	LOC Os01g62650.1	INSNEKKLNCSSSFNTASSKAVEPD	S	335			494	LOC Os01g62650.1	ATPDSHYVPRVPSPGLPPVGVRINS	S	313
496	LOC Os01g63250.1	SRSQDAFAIESTSGEMSKDGNLEEQ	S	285			497	LOC Os01g63930.1	KLGGAATGSDLLSRFMGSIDDDKYL	S	294
499	LOC Os01g64000.1	MEEAKVVDSGSGSGDAGGSGLCRQG	S	105			500	LOC Os01g64000.1	GDAGGSGLCRQGSFSLPLPLCQKTV	S	118
502	LOC Os01g64280.1	MSQERSQSQSPRSPASAAGVPFLSI	S	13			503	LOC Os01g64450.1	DSAELTRFELGGSDGAKRGQLLRYN	S	97
505	LOC Os01g64670.1	KRPAPRLNERILSSLSRRSVAAHPW	S	25			506	LOC Os01g64670.1	PEYRHYNDLSELSPHRVQEIRRFFE	S	159
508	LOC Os01g64970.1	KSSRTGYWSDAGSDEEEKEEEERPE	S	318			509	LOC Os01g65230.1	SSEVRSHISRLASIDSFDSRWQDFL	S	386
511	LOC Os01g65260.1	MAAAAAAATATASSGLIRCATGGAP	S	13			512	LOC Os01g65330.1	RSPTQRGHTITLSPNKASSNSSPLL	S	554
514	LOC Os01g65900.1	WADPMKNVILPNSPKESESSISCAG	S	162			515	LOC Os01g66130.1	GLKQLRDNGFQVSDEEQYLERNGKS	S	413
517	LOC Os01g66140.1	RLNDRKSVIVIDSDSDEDEDPHPEQ	S	751			518	LOC Os01g66140.1	NDRKSVIVIDSDSDEDEDPHPEQHE	S	753
520	LOC Os01g66520.1	HLSSEKCEEQDGSDIDDDENSRPSF	S	288			521	LOC Os01g67126.1	NLKKLTYEQRKASLVERLNALNSSA	S	277
523	LOC Os01g67126.1	ASLVERLNALNSSAGADDDDEEEDD	S	288			524	LOC Os01g67170.1	KRDRKRKRRRYDSDEDDLDQLLELG	S	448
526	LOC Os01g67250.1	SAQAVDDMKGELSDIVHDNVNAFDN	S	857			527	LOC Os01g67250.1	LMEETVPSRVHESPVLSPQRKASPS	S	316
529	LOC Os01g67250.1	LGQVTAEKGTTESDGSNKVGSLTSR	S	524			530	LOC Os01g67250.1	VTAEKGTTESDGSNKVGSLTSRKRH	S	527
532	LOC Os01g67330.1	RSKESVVVNVSDSIATEAKHRHGTD	S	382			533	LOC Os01g67550.1	NPLGSLRLARLRSILDEGKSLQLQF	S	519
535	LOC Os01g67860.1	GTPGKGILAADESTGTIGKRFASIN	S	32			536	LOC Os01g67860.1	DESTGTIGKRFASINVENVEENRRS	S	42
538	LOC Os01g68370.1	NSSGAVGKHKHGSPEKPGVSSNTKA	S	658			539	LOC Os01g68750.1	PSYYKEDHQMSRSQPSGDSLSGEFG	S	705
541	LOC Os01g68810.1	SIVKDESDPNSASKRRKIKRDQSGL	S	1698			542	LOC Os01g68810.1	DKFRPMDHDKSASEEQILSRPEKSK	S	1493
544	LOC Os01g68970.1	SNSFIKNFEKSFSSTFRTLHLVNEI	S	140			545	LOC Os01g69030.1	DTPMKKKFQRNFSELTVSWSDENKE	S	117
547	LOC Os01g69090.1	KNGAWRGISKRFSVLYNGKWLKNME	S	139			548	LOC Os01g69090.1	SQAHQMAFGRMRSMYRGRSAPLMCK	S	337
550	LOC Os01g69100.1	AAGVGVRSPGSGSKDEMFFDSRAWL	S	65			551	LOC Os01g69130.1	TPKQPRSRKSSHSGEQLPFNPDASG	S	803
553	LOC Os01g69130.1	AHGLSSSFQDGSSPYSTPKQPRSRK	S	787			554	LOC Os01g69130.1	YSTPKQPRSRKSSHSGEQLPFNPDA	S	801
556	LOC Os01g69260.1	DRVSTRESSEKWSASAYVYLQWLGR	S	75			557	LOC Os01g69260.1	VSTRESSEKWSASAYVYLQWLGRTA	S	77
559	LOC Os01g69910.1	NQAALRKLKVQLSLEDREDHDVDAK	S	218			560	LOC Os01g69990.1	IENSGKLGLRSSSGSMLDMQRGDFS	S	1319
562	LOC Os01g69990.1	SVEQHEHLERSLSLHERLHRGGQGI	S	1080			563	LOC Os01g69990.1	VDIPVSTNKEAGSFISPSGTSVDGP	S	1452
565	LOC Os01g69990.1	VHPQQHRLQEQLSGNLARLDRHWSD	S	1156			566	LOC Os01g69990.1	GHFDLLQSLQRSSSVEQHEHLERSL	S	1067
568	LOC Os01g69990.1	GGQGIHSLERSGSLPGGAPLPNLDV	S	1100			569	LOC Os01g70020.1	ASTRPAKGVSKPSQDTEEDDEPEVE	S	331
571	LOC Os01g70020.1	SEVKHIIEEVINSMSDDEEGEEDNA	S	475			572	LOC Os01g70020.1	RKSNVHFLKRNISQFSGFVWTDNQE	S	141
574	LOC Os01g70020.1	KTTNKNKGKGKGSTEAGAAPTTEEL	S	413			575	LOC Os01g70160.1	TQEESLALRRNGSSSGIEHLAAQKK	S	126
577	LOC Os01g70320.1	LRAGSPVYGRQRSGSSTGSSSPGGV	S	16			578	LOC Os01g70320.1	AGSPVYGRQRSGSSTGSSSPGGVSP	S	18
580	LOC Os01g71000.1	LDDLDMTGSQMGSPAQTGRKRETPR	S	444			581	LOC Os01g71000.1	LAGWNEDRRSVRSPNHHSPDLRREA	S	397
583	LOC Os01g71050.1	EAPILREVPVHISPVRETSFLREVP	S	200			584	LOC Os01g71050.1	ETSFLREVPAPVSPVKETPILREVP	S	216
586	LOC Os01g71230.1	EQFKAPDLSNVISKAEPSAAAQDDE	S	144			587	LOC Os01g71540.2	VSNESSSLNHNISFLGRIQQLSGTH	S	493
589	LOC Os01g71990.1	GKHPGQLKDMVTSPAGTTITGIQEL	S	246			590	LOC Os01g72080.1	MKLSIQSFARKLSLPSPKRTWSSGG	S	13
592	LOC Os01g72320.1	GSDHYRLRSPFGSPPKNALDKAFSD	S	181			593	LOC Os01g72320.1	ESRSDIISSGATSPRTYTRRSSPLS	S	144
595	LOC Os01g72890.1	PPPKKASPPRKASPAPESVVLHIDH	S	101			596	LOC Os01g72890.1	ASPPRKASPAPESVVLHIDHLSRNV	S	106
598	LOC Os01g72890.1	RQPDPSPRRRPDSPPIRRRADASPV	S	262			599	LOC Os01g72890.1	PRRRSPGPIRRRSPPPPPRRPRSPP	S	330
601	LOC Os01g72980.1	STLRSAATKADDSSDEVVHASATNA	S	705			602	LOC Os01g73040.1	GKHIPPHVGNSFSFKLQDRKGRMHR	S	405
604	LOC Os01g73300.1	KIILHKTIDSVLSRGERLDSLVEKS	S	178			605	LOC Os01g73620.1	FEASAVKSNFGSSPDFKHSNHSSVH	S	825
607	LOC Os01g73644.1	VLDSVVGMDFLLSDSGGNVGADGKT	S	55			608	LOC Os01g73644.1	DSVVGMDFLLSDSGGNVGADGKTRY	S	57
610	LOC Os01g73950.1	AAADAVRALEDGSDSDASAELDEKL	S	152			611	LOC Os01g73950.1	ADAVRALEDGSDSDASAELDEKLEG	S	154
613	LOC Os01g74020.1	SAATVSRLRRASSSSSSSFPAFASK	S	77			614	LOC Os01g74030.1	IGMPGLEIGRQNSFSGIFQDDSYQH	S	146
616	LOC Os01g74030.1	QYMDQTNGNQRHSYMKRSEQGHVKV	S	324			617	LOC Os01g74030.1	SYASIIGSSLSRSASPDPELVRRVP	S	223
619	LOC Os01g74370.1	RHSRSGDGNGWASPERTMVWRTELK	S	18			620	LOC Os01g74470.1	ASERAPSIKRQDSFEMKLPDLPKID	S	739
622	LOC Os02g01030.1	PEPDELDQDIVESDLELEGDIVESD	S	94			623	LOC Os02g01110.1	DRGSRRELSWSKSNPGNYGENMQNV	S	311
625	LOC Os02g01280.1	TKVVRCCLEHAASVAKTFITSDAVV	S	563			626	LOC Os02g01280.1	VVGGGCTLLRLASKVDAIIETLEND	S	475
628	LOC Os02g01490.1	DSGRKIAHNRSVSEPDFGRTPKQGA	S	1186			629	LOC Os02g01490.1	SPLMPGKQSHSRSNSNSSVQFNSLT	S	1494
631	LOC Os02g01490.1	LMPGKQSHSRSNSNSSVQFNSLTED	S	1496			632	LOC Os02g01490.1	YSEQINAVAGARSPDQQTMPSPLMP	S	1474
634	LOC Os02g01510.1	RSRSRSRDMKKASSLSELGFDAEGA	S	44			635	LOC Os02g01960.1	KEAGRSRNRKQKSLENLFVTNSQRV	S	572
637	LOC Os02g02050.1	DSQTKHQLETVLSQRGETCGHLGDA	S	1861			638	LOC Os02g02290.1	GSPSSSGRTGPLSPTNAGRMGPAPS	S	2122
640	LOC Os02g02290.1	APSSSSKKLRSLSALDSRPGALSKR	S	1709			641	LOC Os02g02290.1	IYREIDDDDFEESDDDSEERNTSSL	S	1615
643	LOC Os02g02360.1	QNLAEWEELDQMSEAGGDLKSRILP	S	200			644	LOC Os02g02360.1	ALKESEQSIETSSAAPSIDDDKILS	S	291
646	LOC Os02g02410.1	IKLIKKKYSKDISKDNRALGKLRRE	S	283			647	LOC Os02g02570.1	RSCEKLESSASSSPQEANVANKGKI	S	320
649	LOC Os02g02870.1	RSYGGSWGGGRRSGGGGPGGGCFKC	S	117			650	LOC Os02g02870.1	GGSGGGGRSYGGSWGGGRRSGGGGP	S	110
652	LOC Os02g03040.1	SPQGGNKGDERGSPPNNYDRERREA	S	249			653	LOC Os02g03040.1	PPNNYDRERREASPGYDRPRSRSPA	S	262
655	LOC Os02g03080.1	DKDDDDETEDEDSDAEEDPEKIAMS	S	200			656	LOC Os02g03080.1	SNLGKWQENFVSSQGRQGGGRSGYS	S	858
658	LOC Os02g03080.1	AEEDPEKIAMSESDEGDDDEEDDQD	S	214			659	LOC Os02g03740.1	DTKAFSALSLSSSSRDTQDGSKNKP	S	236
661	LOC Os02g03740.1	SDTKAFSALSLSSSSRDTQDGSKNK	S	235			662	LOC Os02g03820.1	DTQRDYKLTKSISLGCSFIVKASNL	S	387
664	LOC Os02g04050.1	TEVMNLLESAGFSRSNPYYVVQQGK	S	131			665	LOC Os02g04240.1	FDANKENIPCPNSPEKSVCRSKRYK	S	149

667	LOC Os02g04270.1	AEVTKDGDDSDDSDGMDDDAMFRID	S	995			668	LOC Os02g04450.1	SFGFLSQIPRNFSFSDLTEDFSQSA	S	267
670	LOC Os02g04660.1	RSNHNVSQGGVLSGDENHTEDTAVR	S	275			671	LOC Os02g05310.1	DDYRRRRLARIISPERHDPFAAGEA	S	126
673	LOC Os02g05450.1	QMFGEPIGNDEQSEDEDWGLNKRKK	S	399			674	LOC Os02g05450.1	QRTESSSELISVSKRATRQNTPRKP	S	31
676	LOC Os02g05450.1	QKGLDKKPKSINSPPRPQDNETCLS	S	557			677	LOC Os02g05450.1	SPPRPQDNETCLSPTDKTKEGTPPT	S	569
679	LOC Os02g05620.1	SALFIPRPRRAGSFQIRHTNDKITS	S	639			680	LOC Os02g05840.1	MPGGSGLDQNNGSPKANSGGQSDPS	S	458
682	LOC Os02g06430.1	EVAPQHPPERTVSLPSDSGNLGVKP	S	625			683	LOC Os02g06740.1	SRLSSMCLIPKLSIVKGSKCHSCVQ	S	335
685	LOC Os02g07070.1	DLCESEHERRGLSHERDRSPYMQHS	S	168			686	LOC Os02g07070.1	HERRGLSHERDRSPYMQHSRSRSRG	S	174
688	LOC Os02g07070.1	ERDRDYDRYSYDSDYEKSRRDGSWR	S	141			689	LOC Os02g07070.1	DSYERDRDYDRYSYDSDYEKSRRDG	S	138
691	LOC Os02g07190.1	LKRAGLDDSLSESFRRTEIFKDKSA	S	809			692	LOC Os02g07210.1	AKNSGRSLGKRLSHLDSIPHTPRVN	S	48
694	LOC Os02g07260.1	NPKKPFAAIVGGSKVSTKIGVIESL	S	201			695	LOC Os02g07630.1	STVSQPKLQNTLSGQFRIGGMTCAA	S	132
697	LOC Os02g08190.1	AAKSSAAAATPASIDAIDRHLRSLH	S	56			698	LOC Os02g08190.1	SIWEDATDGFVPSPSRSPMPSRSPI	S	240
700	LOC Os02g08350.1	SAFKANDCNLHISGGRQTQVRDESS	S	718			701	LOC Os02g08350.1	HTSELRNKELLVSELQSTLEDKSKR	S	839
703	LOC Os02g08360.1	LPASKWSREDDVSDDEDRKGGRGLG	S	774			704	LOC Os02g08360.1	RRRLQSEYGLSFSNDGANSRRSSER	S	873
706	LOC Os02g08360.1	RKGGRGLGLSYSSGSDIAGDSGKAD	S	791			707	LOC Os02g08360.1	DEDRKGGRGLGLSYSSGSDIAGDSG	S	788
709	LOC Os02g08364.1	ITQNKKADVGEQSVKGVEEAEINAA	S	345			710	LOC Os02g09740.1	RLMQRASIVRRQSGEELGYSYRAPE	S	696
712	LOC Os02g09920.1	SFQDNLEASSTKSIELDADNNEMDT	S	422			713	LOC Os02g10020.1	ECAEEPVPQVPKSTKPSILKLRQAT	S	361
715	LOC Os02g10220.1	RAQRKDRFDSVLSSIMSKCDPKGSS	S	245			716	LOC Os02g10220.1	LSSIMSKCDPKGSSSSEPTEEEFEQ	S	256
718	LOC Os02g10510.1	HIRDEEKLSKLASLSQGAAGESSTA	S	335			719	LOC Os02g10510.1	RDEEKLSKLASLSQGAAGESSTARE	S	337
721	LOC Os02g10690.1	NPSNAVKKPQRKSLPKLPSEETGPF	S	328			722	LOC Os02g10790.1	CAENLRSANLLQSPGSSNLAMNGNK	S	478
724	LOC Os02g10970.1	SKTERNADIALTSNRSKSRFPIFLH	S	408			725	LOC Os02g10970.1	ERNADIALTSNRSKSRFPIFLHTSI	S	411
727	LOC Os02g10970.1	SKRHHRGRKSKASSQVSGENHTALA	S	345			728	LOC Os02g10970.1	HRGRKSKASSQVSGENHTALAALKD	S	349
730	LOC Os02g10970.1	SATPRSLAQEDKSPKENGDTRTNGV	S	381			731	LOC Os02g11060.1	WQLGPATSDEQESPPNANGKLKVNT	S	309
733	LOC Os02g11750.1	DYDRAAEMHNVDSDNDREKENSKDY	S	178			734	LOC Os02g11750.1	FSRPKDHDSRDLSVSSDDLHSDAKR	S	218
736	LOC Os02g11750.1	RPKDHDSRDLSVSSDDLHSDAKRQL	S	220			737	LOC Os02g11750.1	PKDHDSRDLSVSSDDLHSDAKRQLN	S	221
739	LOC Os02g11820.1	DDRNSRYSYGERSPGYEHNDYKKSP	S	186			740	LOC Os02g11820.1	NRRSDGNWGGSRSPPYNESYSDRRS	S	156
742	LOC Os02g11820.1	PPPQMQRTSTASSIGSSEGTSEQIK	S	327			743	LOC Os02g11820.1	RRPSEPQRPDNGSPNYQKETDGSSP	S	236
745	LOC Os02g11820.1	GSPNYQKETDGSSPVVRPVRDILGD	S	247			746	LOC Os02g12310.1	TSWIMHEYRLMNSPRAAVPSSSSVN	S	146
748	LOC Os02g12360.1	QDISGAGKKWDSSEEDDLDGYKELG	S	641			749	LOC Os02g12850.1	RDGGDGGAYGDASGDDGRAPGGDSS	S	53
751	LOC Os02g13130.1	LINEVLAEADAASSGNLSSRKYNAP	S	220			752	LOC Os02g13130.1	DDDAGAGVGSLGSSGGGGGGRATGL	S	96
754	LOC Os02g13560.1	DIAHHAHRGSALSMRRRSLLEEGGE	S	398			755	LOC Os02g14530.1	GSRRQVSNQKAVSLPSSPHRLRSDG	S	477
757	LOC Os02g14530.1	QVSNQKAVSLPSSPHRLRSDGSGLR	S	481			758	LOC Os02g14530.1	NWWSSHAEHKLGSISLTKQERNSGS	S	31
760	LOC Os02g14530.1	KVVEEHQRTASSSPEHPLSRTRGRS	S	356			761	LOC Os02g14530.1	ASTSNMRRGRRRSISFTPEIGDDIV	S	399
763	LOC Os02g14770.1	AAAGKAAMERHQSIDAQLRLLAPGK	S	15			764	LOC Os02g14780.1	QPKQVIWDGHSGSIGRTATQALSMG	S	515
766	LOC Os02g15060.1	SHRTCPMCRCDLSPPREVAAKEATA	S	151			767	LOC Os02g15220.1	QQPSQEPNIGVKSPKQNLIVDEKLA	S	594
769	LOC Os02g15220.1	YPRKNPKPPPNPSPSSSPLAQTLAS	S	61			770	LOC Os02g15220.1	KNPKPPPNPSPSSSPLAQTLASIRR	S	64
772	LOC Os02g15220.1	SPSSSPLAQTLASIRRSIRRPEDGP	S	73			773	LOC Os02g15310.1	RSRSPRYRGRPRSRSYSPAPRRRDD	S	163
775	LOC Os02g15310.1	SPAPRRRDDYSASPQRKDTHRAKSP	S	179			776	LOC Os02g15910.1	SNSDIKMKQGYMSMKALVDDRTPKD	S	50
778	LOC Os02g16600.1	RDSYSKDPNFLLSPKLPTASTSSCD	S	290			779	LOC Os02g16620.1	AGKAHVELSRQYSVLDAKVLSKIPR	S	209
781	LOC Os02g17980.1	LLDRVERRLTASSMFLPQGGRLTLI	S	222			782	LOC Os02g18660.1	IVMPKEATTTAASDDGTLVGSKKGK	S	439
784	LOC Os02g18660.1	IATDDDDGTLGGSKREKAGKEELDG	S	411			785	LOC Os02g18660.1	GGDEEGEEVEGDSSESEPEPEPVKK	S	98
787	LOC Os02g18660.1	EEGEEVEGDSSESEPEPEPVKKESA	S	101			788	LOC Os02g18660.1	PPPQKPSGRGAASSDEEEEEEEDSD	S	59
790	LOC Os02g18660.1	VSKYNLAPEPSPSSKSGKALSRWTT	S	193			791	LOC Os02g18660.1	SKYNLAPEPSPSSKSGKALSRWTTD	S	194
793	LOC Os02g18910.1	VVDYVLVIYAAKSATARSFEKKVIN	S	56			794	LOC Os02g19150.1	YKRIHCELLSSRSAGDCSETDSCAS	S	191
796	LOC Os02g19170.1	PTLASMLKKKVTSKDRIAERLLNAR	S	685			797	LOC Os02g19220.1	RPLLDVEQTSESSDDVTEKSASKTL	S	511
799	LOC Os02g19420.1	DLLDPELKRHAPSELRKKREMFQNL	S	33			800	LOC Os02g19804.1	KNIRKRPTAPAGSDDDDEDGSGAIA	S	39
802	LOC Os02g19860.1	APPSRRPLKRMSSPERWEAKQLIAS	S	419			803	LOC Os02g19860.1	FLQGQSRFSIDMSPVKIFKNPEGSL	S	483
805	LOC Os02g20970.1	NESESSEEFRKISAEYEPVDIEGLE	S	469			806	LOC Os02g20970.1	PDDQHSSPERGESESPTKRHLRKKA	S	620
808	LOC Os02g22090.1	DGQTACSKNQGLSDGEIGNGKSDFV	S	252			809	LOC Os02g22210.1	KSEIAKLKVQLSSEFEMKDLGAAKK	S	835
811	LOC Os02g22370.1	QAQKRNALGRQPSQEILSEEKTLPL	S	531			812	LOC Os02g23827.1	SAKEITAVPPHESPKALLEDEVPDI	S	382
814	LOC Os02g24080.1	SKKPSRSSNPFDSDSDDGGREQRPA	S	61			815	LOC Os02g24080.1	KPSRSSNPFDSDSDDGGREQRPARA	S	63
817	LOC Os02g24354.1	DDGSIAKIRRGESFDDYMKFFDNLS	S	477			818	LOC Os02g24800.1	AAQDGSRGTSNASPVVVAPRSREAT	S	327
820	LOC Os02g25580.1	RPRGSDDGYSSDSVLKHSSSDFKKL	S	590			821	LOC Os02g25580.1	RRTGGTWARPRGSDDGYSSDSVLKH	S	582
823	LOC Os02g25580.1	WARPRGSDDGYSSDSVLKHSSSDFK	S	588			824	LOC Os02g26140.1	PPGKVQVAAAAASVSKTRRCTESPS	S	316
826	LOC Os02g26349.1	PDAGRHKRRRDGSPAAAATDHKDDK	S	103			827	LOC Os02g28810.1	RTLDAHIEEQFGSGRLLACISSRPG	S	176
829	LOC Os02g28980.1	KRKETKLYGNMISKLSKLEDSETEG	S	602			830	LOC Os02g29400.1	GGGGGEGEGILASFSRSSVAAHGRE	S	20
832	LOC Os02g30050.1	RAIRRRLTKHQLSLKTEREKKREKY	S	101			833	LOC Os02g30140.1	PPPSLSSSLRSLSLSSSPRGRHHRG	S	35
835	LOC Os02g30230.1	DPTGLFFSLRRLSLGSMDDLDTSSE	S	190			836	LOC Os02g30230.1	GLFFSLRRLSLGSMDDLDTSSEFDA	S	193
838	LOC Os02g31150.1	ERMKYWSEYRNFSVSPPLKDSFSPV	S	408			839	LOC Os02g31150.1	MKYWSEYRNFSVSPPLKDSFSPVTS	S	410
841	LOC Os02g32340.1	NAPVNKTIVHSKSNVQETEEVCGEN	S	458			842	LOC Os02g32340.1	NKNSWSDLNISKSFHLPPLNDENIR	S	556
844	LOC Os02g32469.1	AKCVCAPATHAGSFKCRLHRTNSQG	S	66			845	LOC Os02g32490.1	AGASSDKLRHVESMSELPSGAGRIS	S	22
847	LOC Os02g32550.1	RSRSVGCGSRSFSGDFLERISNGFG	S	280			848	LOC Os02g33320.1	QKNHPASHDQGHSPDVAPEQKSSDG	S	119
850	LOC Os02g33320.1	RGYQQDIKDSRLSGESPVIPLRLKI	S	493			851	LOC Os02g33320.1	QQDIKDSRLSGESPVIPLRLKIQRN	S	496
853	LOC Os02g33320.1	SMAVSNVSPLLVSGSPQHNLPSTAE	S	441			854	LOC Os02g33320.1	TAENKNSDFLVNSPLEGNLDSKTTT	S	463
856	LOC Os02g33360.1	VKVVADVPGTGSSMDGEVKQAPDGS	S	91			857	LOC Os02g33610.1	EDSETKSSGKFGSFSAPRHSSRSSG	S	670
859	LOC Os02g33770.1	SEVAVIDMNKMLSEGSGGPTAEKSP	S	318			860	LOC Os02g33770.1	AVIDMNKMLSEGSGGPTAEKSPSTP	S	321
862	LOC Os02g34080.1	KASMVRGRSFGTSSLPRGRSFSNQG	S	1274			863	LOC Os02g34500.1	SHKDSLMQETDLSPSTASHKDSLLE	S	495
865	LOC Os02g34560.1	VERQRSFDDRSLSDVSYSGGGHGGT	S	50			866	LOC Os02g34560.1	QRSFDDRSLSDVSYSGGGHGGTRGG	S	53
868	LOC Os02g34560.1	HGGTRGGFDGMYSPGGGLRSLVGTP	S	71			869	LOC Os02g34560.1	MELAVGAGGMRRSASHTSLSESDDF	S	13
871	LOC Os02g34560.1	GAGGMRRSASHTSLSESDDFDLSRL	S	18			872	LOC Os02g34560.1	GGMRRSASHTSLSESDDFDLSRLLN	S	20
874	LOC Os02g34590.1	DRVMEGQGAVTNSGDEMGMRAYGDE	S	60			875	LOC Os02g34680.1	HQWPRNVLYEDDSEETEEEGDNVGD	S	312
877	LOC Os02g34840.1	AAKRRAAGDDGPSESADDDIVVAQI	S	28			878	LOC Os02g35150.1	GNGERSDTLEVTSDSDTRDMSSDAW	S	639
880	LOC Os02g35150.1	DDRWVATNSDVDSDLETQYQRSSSE	S	578			881	LOC Os02g35190.1	LRYNDSGGGRSGSAGEPLLRKRTMN	S	53
883	LOC Os02g35190.1	GQSQHRAPEREGSHNYDIESTDGSG	S	15			884	LOC Os02g35690.1	GGGGGGGREDAWSDGATSTLIDAWG	S	65
886	LOC Os02g35910.1	LRCWPGGLCLSRSIGDQDVGQFIVP	S	222			887	LOC Os02g36974.1	LRDNLTLWTSDISEDAAEEIKEAPK	S	243
889	LOC Os02g37030.1	QQKHCSGNGGGRSFSSGSSAWSSSR	S	143			890	LOC Os02g37030.1	KHCSGNGGGRSFSSGSSAWSSSRSV	S	145

892	LOC Os02g37420.1	IKLQIWDTAGQESFRSITRSYYRGA	S	67			893	LOC Os02g37870.1	PADDEEAEAEAASEGEEDAAAAAAA	S	49
895	LOC Os02g37920.1	DPGKEMDPAIDGSDEDMCLTEKENV	S	366			896	LOC Os02g37920.1	KNGFVLAEDLHASPCNRYFIKAVPF	S	801
898	LOC Os02g38050.1	PSPHFPYPGRLLSSGHLHGDLGNNI	S	404			899	LOC Os02g38050.1	GLKTLSVEAPATSGVKSSQGQQASL	S	256
901	LOC Os02g38250.1	EEEDDTDEDDYDSDGESRALVAKAT	S	78			902	LOC Os02g38430.1	QSGSVSQMQKLRSGISRMLSQRSMG	S	555
904	LOC Os02g38480.1	TAAHSRLMRKGSSDHLVLNIGRESE	S	1681			905	LOC Os02g38480.1	APDDLGGSRSARSLDESIAVIDFPD	S	94
907	LOC Os02g38920.1	VVDLTVRLEKPASYDQIKAAIKEEA	S	256			908	LOC Os02g38920.1	KGILGYVEEDLVSTDFQGDSRSSIF	S	285
910	LOC Os02g38920.1	GGRAASFNIIPSSTGAAKAVGKVLP	S	212			911	LOC Os02g38920.1	PSMKDWRGGRAASFNIIPSSTGAAK	S	205
913	LOC Os02g38980.1	EQLVEQEKSDVISPVDAIRPADDVE	S	509			914	LOC Os02g38980.1	AETVGESEGAAGSPVRVDSEDSSAP	S	48
916	LOC Os02g38980.1	AAGSPVRVDSEDSSAPKRKCISLNS	S	57			917	LOC Os02g38980.1	PVEEDVDICGNASPILIEKDAHNNP	S	439
919	LOC Os02g39030.1	YHSMIDEDDGTVSPKRTIEESVDKS	S	56			920	LOC Os02g39520.1	EGKEVILVDDNDSEQEDGGSGKVDE	S	65
922	LOC Os02g39700.1	RTTVNSDMEEQKSSDDMTEAKEVNA	S	285			923	LOC Os02g39700.1	EAKEVNAGEATESGDKCTVDALLES	S	303
925	LOC Os02g39750.1	VRRWSSAARPEASSRASSCAATAST	S	136			926	LOC Os02g39820.1	HCEEVKEMTRFASDVGIRGEMLREL	S	178
928	LOC Os02g39890.1	SEKNPRFREKGDSDEEDDDYDKRRR	S	230			929	LOC Os02g39920.1	PSGKDVVDDGKLSERTISDELPQEP	S	275
931	LOC Os02g39920.1	YDNIIATICEDSSDVKEDMDADPSG	S	253			932	LOC Os02g39920.1	EGEAAAEVEQGGSAGGASTGGKKRR	S	740
934	LOC Os02g40690.1	TALMDIDDSPRSSGAGAVFLDDEED	S	76			935	LOC Os02g40880.1	WGKKLIVQKRRASLNDFDRFKVMLA	S	101
937	LOC Os02g41550.1	TFDIPKVVLRETSPCALPIDQRVPH	S	526			938	LOC Os02g41990.1	DSAVARSNDVEISPSEGLRNRKQSN	S	218
940	LOC Os02g42040.1	EKPENEKLSDSESQKVQPHDTAFSA	S	828			941	LOC Os02g42040.1	VLEGSKDPRAVESESGEETVDEEGG	S	972
943	LOC Os02g42540.1	PSQPAENDKPKQSPEDSVNKVSSPQ	S	152			944	LOC Os02g42550.1	KDVHGNAQFNSESFSGNQNTDDLHL	S	143
946	LOC Os02g42580.1	APLPAASLVRSASLSEILDVKTETG	S	337			947	LOC Os02g43020.1	QRPEASESSQPSSSPPSQPQEQPEA	S	208
949	LOC Os02g43020.1	ASESSQPSSSPPSQPQEQPEAKARE	S	212			950	LOC Os02g43270.1	RVKAAQDGSRGTSSVSPAVASTDVV	S	318
952	LOC Os02g43270.1	AAQDGSRGTSSVSPAVASTDVVVVP	S	321			953	LOC Os02g43270.1	AVASTDVVVVPRSREATPSGPASDL	S	335
955	LOC Os02g43740.1	ELVAEPTSARSMSFVGTHEYLAPEI	S	528			956	LOC Os02g43870.1	VSFKNLVLERKISDAFSSDLVRQSK	S	358
958	LOC Os02g44642.1	TSPGPSSRVHSGSVSPLHPRAGGMA	S	337			959	LOC Os02g44642.1	PGPSSRVHSGSVSPLHPRAGGMAPE	S	339
961	LOC Os02g44690.1	SGLLRGAFFGGSSEGEEEDEKTRKK	S	143			962	LOC Os02g44740.1	KTDSDVTSLAPSSPPRSPRRSAYYV	S	18
964	LOC Os02g44770.1	SPRSPPKVRRDGSVEFWKNDGGGDG	S	108			965	LOC Os02g44770.1	ERRSSVLRTPTKSGQFISGLMTGKS	S	316
967	LOC Os02g44810.1	SSSDPDQEPTSPSAAGADGAQPRAP	S	32			968	LOC Os02g44810.1	MSSSSDPDQEPTSPSAAGADGAQPR	S	30
970	LOC Os02g45070.1	RARFYMEPDTSDSGSMASGAHTRGG	S	1036			971	LOC Os02g45070.1	RFYMEPDTSDSGSMASGAHTRGGGP	S	1038
973	LOC Os02g45070.1	AFRARFYMEPDTSDSGSMASGAHTR	S	1034			974	LOC Os02g45110.1	LPDPARSTRRKTSPGSSPASVAAAP	S	103
976	LOC Os02g45110.1	PARSTRRKTSPGSSPASVAAAPGGL	S	106			977	LOC Os02g45110.1	TRRKTSPGSSPASVAAAPGGLDAVR	S	110
979	LOC Os02g46090.1	GSAAAADAPHAASMKRGAPAPAELT	S	65			980	LOC Os02g46090.1	LVAAFSYFDKDGSGYITVDELQQAC	S	482
982	LOC Os02g46090.1	TCGVTLRSKYFASFRGASQRHDEAG	S	16			983	LOC Os02g46650.1	ADITNLDRPSTPSPKRKRFFSDDNE	S	562
985	LOC Os02g46956.1	AEVADELIEKDGSQTEPCEASETPY	S	70			986	LOC Os02g46962.1	ALVRKPAGSRARSGGDDDILDDLDE	S	352
988	LOC Os02g46962.1	VSKKMTAGMSSMSFKMKEIFQGQTP	S	35			989	LOC Os02g46962.1	GSEVSKKMTAGMSSMSFKMKEIFQG	S	32
991	LOC Os02g47140.1	SSLAPKIGPLGLSPKKIGEDIAKET	S	38			992	LOC Os02g47150.1	KKQPAKKASTQLSDDEDDEVLALKD	S	1292
994	LOC Os02g47170.1	SKEKITTAEKIRSDGSDFDESIEKC	S	239			995	LOC Os02g47170.1	KITTAEKIRSDGSDFDESIEKCDRR	S	242
997	LOC Os02g47410.1	FGSACLEGKTIYSYIQSRYYRSPEV	S	290			998	LOC Os02g47810.1	GIVPEEHGMGSSSPSAELIACPRAP	S	31
1000	LOC Os02g47890.1	ISSPQEENETSVSPKNNMDQLVLNN	S	236			1001	LOC Os02g47890.1	IACSIDPVRKISSPQEENETSVSPK	S	226
1003	LOC Os02g47970.1	RASLAHRSNSCLSAVAVQDPETAVV	S	450			1004	LOC Os02g48000.1	VATVANAMSRQNSLSFTSSVDEARH	S	224
1006	LOC Os02g48000.1	ELPGVATVANAMSRQNSLSFTSSVD	S	220			1007	LOC Os02g48010.1	NLEVKKDDGQGDSVTVGGTRKRRFA	S	963
1009	LOC Os02g48010.1	EGTSKAEEPATGSKGASQSADDASQ	S	1068			1010	LOC Os02g48010.1	SLLQKCSRIFKFSPRKKAEQSSEQQ	S	786
1012	LOC Os02g48010.1	SEQDEDSEAHSESVSLGGQRRKRRQ	S	992			1013	LOC Os02g48010.1	KGRAKGGVKRTRSVLAVVEDAKEIL	S	936
1015	LOC Os02g48180.1	LDEIKGGGLRHLSSVDGSDLARTIS	S	632			1016	LOC Os02g48180.1	DEIKGGGLRHLSSVDGSDLARTISS	S	633
1018	LOC Os02g48380.1	QPPMNLLALPDFSKASILARTAKAR	S	48			1019	LOC Os02g48380.1	MNLLALPDFSKASILARTAKARDPL	S	51
1021	LOC Os02g48640.1	YPEFAAGLVRQDSFGGGELPRQDSF	S	465			1022	LOC Os02g48640.1	DSFAIGELRRQGSYGVELVRQDSFG	S	487
1024	LOC Os02g48730.1	EAMSSAVDAISCSKGIAAPPTEEAT	S	134			1025	LOC Os02g48740.1	KVKASGRTSQMESFKDKSLSNGRFF	S	549
1027	LOC Os02g48810.1	HEISGEKCSDNSSIVASEANIKSKA	S	443			1028	LOC Os02g48810.1	GEKCSDNSSIVASEANIKSKADSEL	S	447
1030	LOC Os02g48910.1	ELPAAAPPQPKRSTAAAAVARRRIG	S	16			1031	LOC Os02g49070.1	AVKSVTSSISSASGKHVADDTREFV	S	164
1033	LOC Os02g49070.1	FDATNAVKSVTSSISSASGKHVADD	S	159			1034	LOC Os02g49070.1	ATNAVKSVTSSISSASGKHVADDTR	S	161
1036	LOC Os02g49070.1	TQDFLKPSLRITSKGSFDATNAVKS	S	143			1037	LOC Os02g49080.1	SEGSPAYSWRGGSQGSSSGLIVKKT	S	126
1039	LOC Os02g49090.1	MPTSFTDSYSVGSPSKSSTVDQQSE	S	618			1040	LOC Os02g49150.1	SANIPHVNIDSDSDSEGFVEELPRV	S	50
1042	LOC Os02g49320.1	EVLVKDTEWLPGSPEAIKGSYVFVC	S	190			1043	LOC Os02g49450.1	DFDKIDGTSGLESDDDDDKEKAAGK	S	285
1045	LOC Os02g49620.1	KKQMKLKSSQTDSDDMLELLTSKKD	S	80			1046	LOC Os02g49992.1	ANVSPIKGAEFSSPGQLGLTARGDQ	S	229
1048	LOC Os02g50320.1	KVLGNGPHLLNRSTDAVRDCETTDD	S	456			1049	LOC Os02g50550.1	GLNTPEGGSMRQSHSDGSLDTMARK	S	724
1051	LOC Os02g50550.1	EGGSMRQSHSDGSLDTMARKPADPE	S	729			1052	LOC Os02g50550.1	AKRRREKYQKQSSLLSKLTRQLSIH	S	820
1054	LOC Os02g50570.1	GGSTSAGLGRSNSPEQIFLLEPRKS	S	475			1055	LOC Os02g50620.1	EMDTYRYHGHSMSDPGSTYRTRDEI	S	295
1057	LOC Os02g50620.1	AVAVGMEAAITRSDSIITAYRDHCT	S	110			1058	LOC Os02g50620.1	AVGMEAAITRSDSIITAYRDHCTYL	S	112
1060	LOC Os02g50790.1	RLIPSPQTRDDASQGETTDANPPLK	S	748			1061	LOC Os02g50799.1	PLVAADPCDLAGSPMKEMAFSDPAN	S	377
1063	LOC Os02g50840.1	NNLLSQGDKGTTSAGSSPLPEIDSK	S	924			1064	LOC Os02g50840.1	LSQGDKGTTSAGSSPLPEIDSKAKT	S	927
1066	LOC Os02g50840.1	VRFPNPGRSPARSASGGSFEDPSFT	S	683			1067	LOC Os02g50840.1	PGRSPARSASGGSFEDPSFTGSRAS	S	688
1069	LOC Os02g50840.1	FPNPGRSPARSASGGSFEDPSFTGS	S	685			1070	LOC Os02g50840.1	AGEISKSELISSSPSPRNSSANEQG	S	842
1072	LOC Os02g50850.1	FDDWLQEAHKGKSGEGGSPKETDKP	S	295			1073	LOC Os02g50850.1	SWHSNDVKGASVSEEGDKSPYAMLT	S	662
1075	LOC Os02g50850.1	FEDPSFTGSRASSPVLADKHEQSDR	S	617			1076	LOC Os02g50850.1	SELISSSASPRNSPGNEEGCEGDNI	S	765
1078	LOC Os02g50880.1	EAPADADGSRPPSPPRRGEAKPVAN	S	67			1079	LOC Os02g50880.1	SRRHEAPADADGSRPPSPPRRGEAK	S	63
1081	LOC Os02g50910.1	EEKEGGGVVEVSSSDDVDDAKVQEI	S	401			1082	LOC Os02g50910.1	NRDIPLRLPRAMSSPAVDSIPLTDR	S	500
1084	LOC Os02g50960.1	KDREDYVERDDFSFGNRGVMDRDAE	S	392			1085	LOC Os02g50970.1	ERRQSVAAERRRSQEEEWERRRSQE	S	102
1087	LOC Os02g51400.1	PALMFHNLSRLSSLPSSLKRLVGGR	S	484			1088	LOC Os02g51400.1	DWTSNGEAKTDESDDEDALKSVEEL	S	229
1090	LOC Os02g52150.1	TGAQLRRHERDESDDDSGRGYDTRR	S	58			1091	LOC Os02g52150.1	PFSAPQSLGRLLSLMDDLATPAGRA	S	101
1093	LOC Os02g52250.1	AFDAVVKQGENASKIVYSKHSDLVP	S	124			1094	LOC Os02g52250.1	VKQGENASKIVYSKHSDLVPKIATA	S	129
1096	LOC Os02g52250.1	KFKHKRVPRASGSPPVPVMHSPPRP	S	239			1097	LOC Os02g52280.1	DSKELVMESDIRSRMQALKDQLVEA	S	925
1099	LOC Os02g52780.1	QSTLGGVGKDFGSMNMDELLRSIWT	S	48			1100	LOC Os02g52780.1	AAVGAPPVQRQGSLTLPRTLSQKTV	S	98
1102	LOC Os02g52810.1	WEDAEKWILSPVSCDGIGRMSAPAP	S	249			1103	LOC Os02g52820.1	QQQAKQENAGTLSNGDNRVDDDAID	S	295
1105	LOC Os02g53270.1	YDLLPYSPSPPASDEERRDRRKDRK	S	115			1106	LOC Os02g53520.1	RFSPHPGPRRRHSFGEEELAYLPYK	S	515
1108	LOC Os02g53580.1	ANKRLKVEATVTSDGAVVRQRHEAA	S	56			1109	LOC Os02g53760.1	GAANWRGDGGRDSGDEGWRPARSSV	S	31
1111	LOC Os02g54110.1	DNMSMTSRATRQSRWNHTQMFSDFG	S	1047			1112	LOC Os02g54170.1	RTLQYDEDAQDVSPGTDQNQRTNAE	S	303
1114	LOC Os02g54200.1	GSQLVSDDDNAESDDEVLYRRTVML	S	473			1115	LOC Os02g54280.1	NHYGRKRVRVVISDDEAEEAPEIDQ	S	514

1117	LOC_Os02g54510.1	NTGTDQDSDVHGSGEQQKSSGIEAT	S	779			1118	LOC_Os02g54510.1	SDVHGSGEQQKSSGIEATTAEAEAL	S	786
1120	LOC_Os02g54510.1	HSQRYGEMDSPWSPAYISPGHYGVH	S	210			1121	LOC_Os02g54510.1	GEMDSPWSPAYISPGHYGVHDPRDF	S	215
1123	LOC_Os02g54770.1	ECRLRIGPGGLGSGKRRSRSRSRSR	S	112			1124	LOC_Os02g55020.1	HGLCRVGRYHMASKFLRICLHEGNN	S	424
1126	LOC_Os02g55400.1	LRELHTLKGHVESVVKLKGLDIDTI	S	866			1127	LOC_Os02g55940.1	DQKTKDDIKKTRSMQGLSFLANIGG	S	290
1129	LOC_Os02g56210.1	SARGTVWKAKNSSTGAAPVENPVIT	S	273			1130	LOC_Os02g56210.1	AMAGGGGLNRSSSRGQLPPQELLDD	S	14
1132	LOC_Os02g56270.1	PMERKPSNQQIASPPPNYEDVTRDT	S	301			1133	LOC_Os02g56270.1	YRSAFATSGTHRSPGGYDNDRYEGS	S	172
1135	LOC_Os02g56480.1	PVSDHVVVSPVVSPGEFQRQIQGLE	S	290			1136	LOC_Os02g56480.1	HTTNPVSDHVVVSPVVSPGEFQRQI	S	286
1138	LOC_Os02g56480.1	LLRPAPGSGGGSSRGGSTPRLRVFL	S	142			1139	LOC_Os02g56480.1	QLSYVNGETKILSLERPLRFADFAA	S	71
1141	LOC_Os02g56560.1	VARGSRDEPLHRSLELLSLGGGKRK	S	450			1142	LOC_Os02g56740.1	KNRPTVLDQDDDSDVSAVEDDDYSK	S	554
1144	LOC_Os02g56740.1	QHAASFDDDDDGSEDDFKNRPTVLD	S	537			1145	LOC_Os02g56740.1	PKDKLEELQHAASFDDDDDGSEDDF	S	529
1147	LOC_Os02g57080.1	RIEGARDPRFALSTGGHAQSPALQN	S	697			1148	LOC_Os02g57080.1	PRFALSTGGHAQSPALQNRSGPIRM	S	704
1150	LOC_Os02g57390.1	DSSRAQLQSRSESMNGLRSGSTSPS	S	254			1151	LOC_Os02g57390.1	TTPDPQLIRRTPSPCLPPVGVRMGS	S	305
1153	LOC_Os02g57390.1	SHNFASAVGSSISRSTTPDPQLIRR	S	290			1154	LOC_Os02g57500.1	AAAPAAGGERAPSPSPPPPPRLPAL	S	28
1156	LOC_Os02g57510.1	GLQDRVSILKRLSGDIHEEVETHNR	S	56			1157	LOC_Os02g57540.1	PDLTKPALARLSSVYRSLQVAKSGV	S	125
1159	LOC_Os02g57600.1	EHQKELSGQDAVSAEGDDEKFQDTC	S	95			1160	LOC_Os02g57600.1	HWIPSFDKGKNLSLVDMEFDKAFQG	S	128
1162	LOC_Os02g57640.1	RSSPPPPQQQASSEDDKEKPTHLRF	S	33			1163	LOC_Os02g57640.1	PRSSPPPPQQQASSEDDKEKPTHLR	S	32
1165	LOC_Os02g57660.1	PHSKNVSLERRWSLEVAIEKFIGHD	S	322			1166	LOC_Os02g57660.1	GHDATGSSGLERSESINDSDLPMLE	S	344
1168	LOC_Os02g57690.1	IAAAHKRLESTDSLENELEVLRQQI	S	475			1169	LOC_Os02g57820.1	RPPGSGKMQQLASLGKWFLGSVGTG	S	197
1171	LOC_Os02g57980.1	AKKDMRFARFGASSEEESGYDELEA	S	260			1172	LOC_Os02g57980.1	KKDMRFARFGASSEEESGYDELEAR	S	261
1174	LOC_Os02g57980.1	AGRFGRKLRPDSSDEDDEDISTAKK	S	238			1175	LOC_Os02g57980.1	KSYEEENNDLAGSFRELREEIKNRE	S	310
1177	LOC_Os02g58110.1	TSLRGIVPAGEQSGDESKRKGGRRA	S	525			1178	LOC_Os02g58110.1	VRLFLKKLAKKASTKVPLEGKTSSG	S	25
1180	LOC_Os02g58210.1	AAEEVEAAAIDGSGEEAKRKSGKQR	S	14			1181	LOC_Os02g58220.1	GSPAKTYSAMGSSSSNGFSEMTTPT	S	183
1183	LOC_Os02g58220.1	TKSQIGSPAKTYSAMGSSSSNGFSE	S	178			1184	LOC_Os02g58220.1	IGSPAKTYSAMGSSSSNGFSEMTTP	S	182
1186	LOC_Os02g58410.1	FMELAPRAPFGHSPVDPRTPSYSKH	S	1061			1187	LOC_Os02g58410.1	GLQLTKDASSLGSSSPIQEQNQKAY	S	1900
1189	LOC_Os02g58410.1	SAVTGRSVSGPLSPLAPEVSIPNPT	S	1443			1190	LOC_Os02g58410.1	SRRNDAPLSRSDSLSIINGMRYLKL	S	164
1192	LOC_Os02g58440.1	EKERDRGPRARLSPPSQTDLRGKLH	S	412			1193	LOC_Os02g58440.1	KREEHSGGDEEGSQNDFRNIEDDIV	S	541
1195	LOC_Os02g58490.1	QQQGRGCRARGASPPPPPQQQQQQQ	S	54			1196	LOC_Os02g58570.1	PRPKAAAPRNTTSFIIRAKRAGGIA	S	98
1198	LOC_Os03g01420.1	NTKLKLRMKKNKSLCHVSNGTVIAD	S	383			1199	LOC_Os03g01470.1	LAGRRRGPHGDGSAEPTARAAAASA	S	109
1201	LOC_Os03g01490.1	KECKNPQLVNLGSGRFCITRFFHSR	S	338			1202	LOC_Os03g01710.1	SDSVANRSVRSSSGNPLGGTTQDEL	S	189
1204	LOC_Os03g01710.1	SSNGGMHVGRQDSASSYVSASRGDD	S	246			1205	LOC_Os03g01710.1	DRSPTNDDIDSKSDGSDSVANRSVR	S	174
1207	LOC_Os03g01710.1	NDDIDSKSDGSDSVANRSVRSSSGN	S	179			1208	LOC_Os03g01710.1	RIAMQAKLVRLESDLSAVEASHVHE	S	1196
1210	LOC_Os03g01970.1	RKQSPEEVSGPESGQCEAEADGEDN	S	614			1211	LOC_Os03g01970.1	IDEGRKQSPEEVSGPESGQCEAEAD	S	610
1213	LOC_Os03g02010.1	AGNCDRTPHSDGSGDEDFFQEMSEK	S	170			1214	LOC_Os03g02150.1	PASNHLTLVKMPSQNITEESNVAYE	S	657
1216	LOC_Os03g02160.1	LEADISNTSVNQSPQSKILPESSPD	S	173			1217	LOC_Os03g02270.1	DSGEPSSGRSTLSEGNGIGSAGAER	S	86
1219	LOC_Os03g02320.1	LALPTRASANSLSAPIRSSGGYVGS	S	540			1220	LOC_Os03g02480.1	SDRSSSVLSQRFSDLENRAKSRRES	S	422
1222	LOC_Os03g03390.1	PAIKEFEDDVSNSPDSTPKEAKPTN	S	98			1223	LOC_Os03g03430.1	KINHRDAHADGLSPSRVSIAGSRRS	S	348
1225	LOC_Os03g03460.1	QKTDELSAVESGSNYSSRRDDDTAM	S	229			1226	LOC_Os03g03460.1	SQQKTDELSAVESGSNYSSRRDDDT	S	227
1228	LOC_Os03g03470.1	VGWEIDPKIIELSRDYFGLSDLEKA	S	176			1229	LOC_Os03g03550.1	ATAPLARHARSLSMDSLIGKFNFTA	S	216
1231	LOC_Os03g03830.1	AESDLGYDFGEESVRSPSSAGRSAS	S	849			1232	LOC_Os03g03830.1	DLGYDFGEESVRSPSSAGRSASGSP	S	852
1234	LOC_Os03g03830.1	SAGRSASGSPFVSSNFAMHDSSPSK	S	867			1235	LOC_Os03g03830.1	DDSSSYNLGRFDSFRSQESGFFPQE	S	998
1237	LOC_Os03g03830.1	NNSKGENVTGFDSPKSSTNFGRFDS	S	1032			1238	LOC_Os03g03870.1	YAKKIEKTENDVSPDLSNAKTKSAD	S	96
1240	LOC_Os03g04330.1	SRTATPPDSRLRSPPPTRLHWFAPP	S	49			1241	LOC_Os03g04450.1	EEHDLSLYPINRSFHMPKQTFDFAQ	S	632
1243	LOC_Os03g04710.1	ETIQSFTQRGGASTDEEEEEDDDDG	S	117			1244	LOC_Os03g04710.1	PRSKPTTLETIQSFTQRGGASTDEE	S	109
1246	LOC_Os03g05200.1	HSPISRRIMRAQSMESLHSSVKGVG	S	594			1247	LOC_Os03g05200.1	MESLHSSVKGVGSDEEEELNMKQEI	S	607
1249	LOC_Os03g05330.1	VKTLTPKFDRADSSQNFQHGDTRTK	S	1027			1250	LOC_Os03g05390.1	DIQLAIKSLRASSMSHEMVEVSTVT	S	294
1252	LOC_Os03g05390.1	QQRSVIIEDSPPSPASNGDKEKEDE	S	336			1253	LOC_Os03g05390.1	SVIIEDSPPSPASNGDKEKEDEVAE	S	339
1255	LOC_Os03g05390.1	SANENLRSRSVNSEADIQLAIKSLR	S	279			1256	LOC_Os03g05390.1	HRFTPARMSHVSSLNPDDMDCISEP	S	244
1258	LOC_Os03g05390.1	SHRFTPARMSHVSSLNPDDMDCISE	S	243			1259	LOC_Os03g05390.1	DDMDCISEPIIRSNSVRSTSANENL	S	260
1261	LOC_Os03g05390.1	ISEPIIRSNSVRSTSANENLRSRSV	S	265			1262	LOC_Os03g05460.1	NAQVERKPSFRRSCEWMVCREPVRG	S	189
1264	LOC_Os03g05730.1	VEHPEKFEKFGMSPSKGVLFYGPPG	S	515			1265	LOC_Os03g05812.1	LGKHRAGFRRMNSVLAGSPKESDNG	S	768
1267	LOC_Os03g06120.1	RMEYIERDQCEYSFDDEDKEVQDAL	S	150			1268	LOC_Os03g06200.1	VTIAIVRKDLVGSAQPITPVMLDYK	S	279
1270	LOC_Os03g06340.1	HARANQSVGSDNSPLLVETDNRTGE	S	417			1271	LOC_Os03g06410.1	DTSMPSRNPESVSPSFARSHKLDTM	S	709
1273	LOC_Os03g06950.1	TKDKSDTSDSSTSPHNEECESFGAV	S	482			1274	LOC_Os03g06950.1	KILEEELADNVGSDRDKDSMSSDIW	S	456
1276	LOC_Os03g06950.1	LQEEHSILRSCDSDLLQTKEKNHNE	S	1067			1277	LOC_Os03g07580.1	SPNSSAKGLRLRSLSPLHGSSLKVS	S	247
1279	LOC_Os03g07580.1	PPANGHELELSRSLPAHLGLDPAKM	S	186			1280	LOC_Os03g07840.1	ATSRFEGFEYSKSMLREEVERYKRF	S	244
1282	LOC_Os03g08220.1	SEEVPRLAASPSSGIKGGGAGERRP	S	61			1283	LOC_Os03g08270.1	QNLTDAYFGSRRSFSSTMDIMSQLE	S	216
1285	LOC_Os03g08360.1	DLARKSGKFDEDSLAFQSRLLAKSG	S	157			1286	LOC_Os03g08360.1	NRGVEPSGPDAGSPTFSVRVRRRLP	S	26
1288	LOC_Os03g08550.1	SLKPPPKIERHKSFDDDDLSNKPVL	S	379			1289	LOC_Os03g08550.1	IERHKSFDDDDLSNKPVLKKTNVAP	S	386
1291	LOC_Os03g08820.1	DPDIFERIDIGGSPVSAARDEKVNN	S	389			1292	LOC_Os03g08820.1	IFERIDIGGSPVSAARDEKVNNDSD	S	392
1294	LOC_Os03g09100.1	EERLQKALARVKSMVQYPDARDQYQ	S	968			1295	LOC_Os03g09260.1	NKSKRPRADDCESPSAVFKKIHSTG	S	20
1297	LOC_Os03g10140.1	GMPDPDAEVIALSPKTLLATNRFVC	S	52			1298	LOC_Os03g10180.1	PESSPKSKRDRTSSGKNGSKSGPED	S	383
1300	LOC_Os03g10180.1	FYKDIFAGSESMSPRRGGAAGDLDV	S	84			1301	LOC_Os03g10180.1	QEFYKDIFAGSESMSPRRGGAAGDL	S	82
1303	LOC_Os03g10510.1	VFDVLPSTKLVTSVKLPDYNSGKVE	S	96			1304	LOC_Os03g10590.1	KWKHDLYEQTNRSPTPKTEEEQIAK	S	344
1306	LOC_Os03g10710.1	DDKKKRIQAGIASDDEGDDEVVVFS	S	35			1307	LOC_Os03g10740.1	QKDATRIKAGIPSDDEGDVDREDVP	S	35
1309	LOC_Os03g11050.1	KQKKDRANLRRVSSFEALHSATKAL	S	216			1310	LOC_Os03g11050.1	QKKDRANLRRVSSFEALHSATKALP	S	217
1312	LOC_Os03g11140.1	TLLGRASARRDLSADETDFCSDDET	S	538			1313	LOC_Os03g11200.1	ILPSEKRIEFLDSLVEKFLTAEPTE	S	590
1315	LOC_Os03g11220.1	HRGRPPPQQHRPSSGGGGGGGSGDL	S	23			1316	LOC_Os03g11220.1	RGRPPPQQHRPSSGGGGGGGSGDLA	S	24
1318	LOC_Os03g11240.1	GQNRSRFARTRSSPELTDPSVEGYS	S	506			1319	LOC_Os03g11290.1	GPRKLKFRRRSASEFSSSEGLSARR	S	658
1321	LOC_Os03g11380.1	LGTPVYRTNPFDSDSDSEVPSRPSR	S	20			1322	LOC_Os03g11380.1	TPVYRTNPFDSDSDSEVPSRPSRAQ	S	22
1324	LOC_Os03g11500.1	ALSRTAVLTASPSSSLASRRFGSRW	S	124			1325	LOC_Os03g11550.1	LEVKRKRIERRNSLTSNISKEAVGQ	S	183
1327	LOC_Os03g11580.1	PLAKRANLLESKSPDGVGQTFRAQP	S	417			1328	LOC_Os03g11580.1	QDGLMGTKKFNGSPDDIDCNEKTND	S	484
1330	LOC_Os03g11580.1	KRSRGQSPQGRRSCSPLSQSLSSGR	S	388			1331	LOC_Os03g11580.1	SRGQSPQGRRSCSPLSQSLSSGRQS	S	390
1333	LOC_Os03g11580.1	SSDMPGDIDNDESVDKNKRTKDSRS	S	885			1334	LOC_Os03g11580.1	LSPEPLGAKDWSSDMPGDIDNDESV	S	874
1336	LOC_Os03g11580.1	SDELGHPVKEDLSGNDSDRGQQTRR	S	357			1337	LOC_Os03g11580.1	GHPVKEDLSGNDSDRGQQTRRHGRS	S	361
1339	LOC_Os03g11890.1	GYGGDRAMHRSESFSGFRREFPKGF	S	83			1340	LOC_Os03g11890.1	LPRDGRENIVALSDTEVPEQEAAAE	S	653

1342	LOC Os03g11970.1	LDNEAIYDICKRSLDIERPTYTNLN	S	216			1343	LOC Os03g12010.1	VAAEDQAGQAASSPLNSPTRGIVVD	S	438
1345	LOC Os03g12120.1	KEVGPPPPPCVDSDHEDLPLAERRR	S	17			1346	LOC Os03g12300.1	RVHLKNQLQSQKSASAIVLSQEIQR	S	675
1348	LOC Os03g12360.1	VTTAGLIDDSPLSPSTPSPSPRPRT	S	49			1349	LOC Os03g12360.1	TAGLIDDSPLSPSTPSPSPRPRTIV	S	51
1351	LOC Os03g12450.1	PHLLHRRDKEEASPSPPPAPAHTPA	S	21			1352	LOC Os03g12450.1	LLHRRDKEEASPSPPPAPAHTPAPR	S	23
1354	LOC Os03g12450.1	SHQRSSIHLSPQSKNQTTGALWNLI	S	732			1355	LOC Os03g12450.1	LSFVERRRFLYLSKIAAAAGKDVDY	S	1081
1357	LOC Os03g12550.1	YCNTLTEFARTNSSDLFTSGQSKSS	S	295			1358	LOC Os03g12910.1	PLEKFHSDVAGRSFHNGRFIQRMRQ	S	245
1360	LOC Os03g13560.1	TSTMGEMANGDVSPKSSPAKQSDAV	S	600			1361	LOC Os03g13560.1	MGEMANGDVSPKSSPAKQSDAVEQK	S	603
1363	LOC Os03g13560.1	APPHHQQQGPGASPSPPPPPPRGRP	S	120			1364	LOC Os03g13560.1	PHHQQQGPGASPSPPPPPPRGRPSF	S	122
1366	LOC Os03g13614.1	SAPSSNSRDVVLSDPAIQDERELKR	S	292			1367	LOC Os03g13720.1	LLAMEDSNKGIDSPSREDSSSVSHR	S	707
1369	LOC Os03g13790.1	CPRGDVCLSSSSSAVNRTPGNESFS	S	140			1370	LOC Os03g13970.1	ESSGAENKDHASSSNADSVMAEAEP	S	263
1372	LOC Os03g14250.1	DSISGPCLPRSSSDPEIEGPTDSGC	S	741			1373	LOC Os03g14260.1	IDGAITPLARTTSSAAAGAGGAAAH	S	284
1375	LOC Os03g14450.1	GVYEALELRDGGSDYLGKGVLKAVD	S	57			1376	LOC Os03g14580.1	AGASKAKKDEDDSGDDDDDYHVPLS	S	94
1378	LOC Os03g14580.1	SGKTTAQKKRRASSDTDDDDDDFIV	S	294			1379	LOC Os03g14710.1	IDGVRRSSRDWASGEFVPKSGGAVS	S	517
1381	LOC Os03g15000.1	NKAEEQQQPSPDSPIKVNSKAEEHQ	S	403			1382	LOC Os03g15010.1	ASVGGFRFSRSDSFVQRFGDDDLIA	S	299
1384	LOC Os03g15040.1	GEPNQDNEPRDSSIELPRVITDMET	S	22			1385	LOC Os03g15050.1	RSAPTTPIKDGASSPFAAALSEEER	S	61
1387	LOC Os03g15050.1	TIDELHSLQRKRSAPTTPIKDGASS	S	50			1388	LOC Os03g15420.1	NKMAGVERMLAPSPSTGAKRERLKK	S	641
1390	LOC Os03g15560.1	GSGSGGEQRRLLSIPKEGERIIAPT	S	19			1391	LOC Os03g15580.1	MRNRRGVVLFAESDDDLADAHAAKD	S	777
1393	LOC Os03g15650.1	EQKSQTLKASRDSDVNLSLKDNFQG	S	304			1394	LOC Os03g15650.1	TLKASRDSDVNLSLKDNFQGPGLEM	S	309
1396	LOC Os03g15740.1	KEMNETDVPISYSGAGNPEKSIRKA	S	609			1397	LOC Os03g15810.1	LPAAAGADQPRRSPQETTAAPSKPA	S	53
1399	LOC Os03g15940.1	PASEKLTPELTRSPSKAARMFSGTQ	S	93			1400	LOC Os03g16000.1	KTAATHEHSRSTSEDHIFTNTSEPT	S	288
1402	LOC Os03g16369.1	ASKKYPAKIDDDSESEDGSPFRKDK	S	674			1403	LOC Os03g16369.1	KKYPAKIDDDSESEDGSPFRKDKRK	S	676
1405	LOC Os03g16369.1	DSEHRKLTKSLNSPNKPERNSTRDS	S	513			1406	LOC Os03g16369.1	ADLELKKAHDDLSPGEKSPSQHSGK	S	582
1408	LOC Os03g16369.1	AHDDLSPGEKSPSQHSGKETRRKQN	S	589			1409	LOC Os03g16369.1	DLSPGEKSPSQHSGKETRRKQNNQL	S	592
1411	LOC Os03g16369.1	RRRSSLDRHWSPSPGRRRPRSPSPG	S	338			1412	LOC Os03g16369.1	GMHNSYSKDGMNSEDAIKGLRDGMA	S	650
1414	LOC Os03g16369.1	TDKHLPSQVRTDSSGEEEGSRAREN	S	542			1415	LOC Os03g16369.1	DKHLPSQVRTDSSGEEEGSRARENA	S	543
1417	LOC Os03g16400.1	LDIFKGTKGGDLSEDEGVVKDCHPF	S	312			1418	LOC Os03g16800.1	SANGPSPRDRWGSPDPYGRRSPSYS	S	180
1420	LOC Os03g17020.1	DDDFRPDMRRNQSVGSFGESGAESG	S	173			1421	LOC Os03g17020.1	FRPDMRRNQSVGSFGESGAESGRQP	S	176
1423	LOC Os03g17020.1	GAESGRQPPRSKSTQDMYTRQQLEA	S	193			1424	LOC Os03g17050.1	STSNASSILNWSSFRRRSDRQFKRG	S	526
1426	LOC Os03g17180.1	FWRSDTNKNSHDSPKDQSPPSEQTI	S	717			1427	LOC Os03g17180.1	NSHDSPKDQSPPSEQTIDNGIPLVA	S	725
1429	LOC Os03g17180.1	QSSKNSEPDSPISPLLTSDPKNERS	S	780			1430	LOC Os03g17180.1	KSLQIEDLSASQSFQESSSPKMAKS	S	681
1432	LOC Os03g17310.1	QEEGKRRDGSDASGSEPAAAAFPAW	S	18			1433	LOC Os03g17310.1	EGKRRDGSDASGSEPAAAAFPAWAR	S	20
1435	LOC Os03g17540.1	WKDKKGLATKLYSFKHDPLCPQVNS	S	395			1436	LOC Os03g17710.1	PPPKDNDERNGDSPWGRENSRSPSD	S	260
1438	LOC Os03g17980.1	MHDGHFLKTSCGSPNYAAPEVISGK	S	220			1439	LOC Os03g17980.1	SNVMHDGHFLKTSCGSPNYAAPEVI	S	217
1441	LOC Os03g18180.1	GKNEEKENEEVSSTAGLVEKLSVTE	S	174			1442	LOC Os03g18310.1	EQRAIFVDRGVGSPEFARPTNETIS	S	194
1444	LOC Os03g19040.1	KPPGTTTTTTTRSPPPPLPVTKVGG	S	53			1445	LOC Os03g19220.1	LISKDHERAYFDSADWVLGKLQTAT	S	44
1447	LOC Os03g19340.1	ECSTQDREGLALSPTNREAVETKPN	S	946			1448	LOC Os03g19340.1	AVNLENHKIDDCSEDNLLDKERIAS	S	1069
1450	LOC Os03g19530.1	AKAVDAGMMEYDSDDNPIVVDKKKI	S	151			1451	LOC Os03g19530.1	PAPKPEALRRADSDDEDDPVESFLR	S	99
1453	LOC Os03g19570.1	ESSKGESHRYTPSPADDRYHKATKR	S	464			1454	LOC Os03g19570.1	YVETNRKDEHVLSDFCRHASKSDQE	S	440
1456	LOC Os03g19590.1	VEIQVRPNIKQNSDGTDTMLFLFRF	S	1087			1457	LOC Os03g19590.1	GRSFDYTQSLRASPTPSSEQQIAAY	S	94
1459	LOC Os03g19870.1	KGKSEELADSIGSLSIGPERTNFKK	S	39			1460	LOC Os03g19930.1	GASRVPPRRLRCSASGAAAVEVRAP	S	47
1462	LOC Os03g20340.1	SVGVVGGGGGAGSPPGSPGRSLSPR	S	59			1463	LOC Os03g20340.1	VGGGGGAGSPPGSPGRSLSPRMFVP	S	63
1465	LOC Os03g20600.1	AVGQLGCIRKSWSNDSLSSYGGGGR	S	40			1466	LOC Os03g20670.1	DSLNEPLLGKSSSDTGGSEVYDPRR	S	41
1468	LOC Os03g20910.1	LFAISRQMGFVGSPRCSPASSPATP	S	234			1469	LOC Os03g21080.1	GYMWNRVSPQLSSPNQPYSLREALA	S	231
1471	LOC Os03g21140.1	HPLQGFDGAECRSPGPGEGGGMFPY	S	192			1472	LOC Os03g21140.1	AFPYSPTGSLPGSPSAATKCLSLLL	S	307
1474	LOC Os03g21160.1	DFHQLGARMLQHSNSANELMLRRKL	S	477			1475	LOC Os03g21160.1	DSDQSGEHNLPDSPFASSTKSTAFF	S	604
1477	LOC Os03g21160.1	HALQAFDGAECRSPGPGESGGMLPY	S	190			1478	LOC Os03g21160.1	LGPAAFPFTPTGSLPASPSATSKCL	S	295
1480	LOC Os03g21160.1	YRKQQQGDFCCMSPTGLDARDPFDF	S	454			1481	LOC Os03g21270.1	TNSILSAQSTTGSFNSTNSDRLRDR	S	1031
1483	LOC Os03g21380.1	RKPSPSFRLRNGSLNALRLRRVFDL	S	26			1484	LOC Os03g21470.1	HPYHVLRINKMLSCAGADRLQTGMR	S	104
1486	LOC Os03g21530.1	AKFDAEYDGSDLSGEEVDNDTKKSK	S	726			1487	LOC Os03g21540.1	SSAKSHTTGSHASSLPSNLCRHFSF	S	518
1489	LOC Os03g21940.1	KSKAEKAREKTLSDQFEAKRAKSKA	S	156			1490	LOC Os03g22120.1	FNASVPRPNRSSSIGNGVQFLNRHL	S	152
1492	LOC Os03g22340.1	KTKVTVTSDGPFSKRYLKYLTKKYL	S	82			1493	LOC Os03g22350.1	DVSDYVTKAGYGSESEVDDEAATVS	S	263
1495	LOC Os03g22350.1	NQVPDLRDLNDVSDYVTKAGYGSES	S	253			1496	LOC Os03g22580.1	SRRNKNSREAKGSPVESVSSSPLKN	S	447
1498	LOC Os03g22730.1	KKKKKKSKDSEDSPVADADGGKKKK	S	499			1499	LOC Os03g22730.1	GQKKKKKKKSKDSEDSPVADADGGK	S	496
1501	LOC Os03g22740.1	EKKEKKKKKKSDSQDAEDVAMETEE	S	540			1502	LOC Os03g22880.1	TNDVDDGEKVDASAKKSKKKKSKAE	S	448
1504	LOC Os03g22880.1	RKNLDVMKAAIDSMVNDATNDVDDG	S	430			1505	LOC Os03g22900.1	LHRKRKRNLDGASDDDEVDAYSNQI	S	87
1507	LOC Os03g24050.1	TSSLFARRAGDPSAAIAGFQRRLYG	S	34			1508	LOC Os03g24180.1	DLYDYKVVHADDSDAERSEDAKAAE	S	464
1510	LOC Os03g24220.1	SWDNTRSVIHGNSFQKKLSLLFGMR	S	733			1511	LOC Os03g24339.1	TPAFKEGSQKQLSPLNRIIRSTSTN	S	857
1513	LOC Os03g24339.1	LKELNHRNLKDKSTSPDSMPMKELI	S	802			1514	LOC Os03g24890.1	PPAPTGLLVRNISLTARPEDIRIPF	S	69
1516	LOC Os03g24890.1	ASPDELERHVSPSNNGHGPPVDGKS	S	231			1517	LOC Os03g24890.1	ARRDYRDHRDDYSPGESLSPHGQDK	S	197
1519	LOC Os03g24890.1	DHRDDYSPGESLSPHGQDKRHHRSN	S	203			1520	LOC Os03g24890.1	RSVSRSPRSRYHSYSPSPSPARRDY	S	177
1522	LOC Os03g24890.1	RSPRSRYHSYSPSPSPARRDYRDHR	S	181			1523	LOC Os03g24890.1	GQDKRHHRSNGRSASPDELERHVSP	S	218
1525	LOC Os03g24890.1	LSPHGQDKRHHRSNGRSASPDELER	S	214			1526	LOC Os03g24930.1	FRKLSSMTNSPASSVAGAAEGGKDD	S	64
1528	LOC Os03g25140.1	DTDAKEAIPRADSGLAFEEERPGLL	S	598			1529	LOC Os03g25260.1	SARTRRMATRMASALASSDNRAQAA	S	28
1531	LOC Os03g25450.1	SAYADGEVKAELSDGEKGGSEKKKK	S	545			1532	LOC Os03g25464.1	HVSTSQADDCILSSMSRISIPAQTK	S	30
1534	LOC Os03g25600.1	AMAGLQRPSKSFSETTFRTISGASV	S	115			1535	LOC Os03g25620.1	QFDLLALEEYGDSDDDDDDPGVRNG	S	263
1537	LOC Os03g25720.1	AGASSAASADGASPRGRGGGGGGLA	S	64			1538	LOC Os03g25890.1	AISNVIIGGIEDSDDVKTYLENIDR	S	18
1540	LOC Os03g25970.1	YGAYGGYSGRSGSSGLSGPSSFSYG	S	525			1541	LOC Os03g25970.1	GAYGGYSGRSGSSGLSGPSSFSYGK	S	526
1543	LOC Os03g26460.1	DLEDDDEDDTADSANKDDEDIKAEG	S	236			1544	LOC Os03g26630.1	EKLNLDRSSGDESMEEDVMEIKQVE	S	380
1546	LOC Os03g26630.1	EGGSPEKLNLDRSSGDESMEEDVME	S	375			1547	LOC Os03g26630.1	GGSPEKLNLDRSSGDESMEEDVMEI	S	376
1549	LOC Os03g26630.1	NDQVPEVSPDLGSPIKCESISSDDI	S	283			1550	LOC Os03g26630.1	YIDSTANVDEGGSPEKLNLDRSSGD	S	366
1552	LOC Os03g27030.1	RYQEKGYRRRSRSISPDYDRRHRKN	S	178			1553	LOC Os03g27030.1	QEKGYRRRSRSISPDYDRRHRKNGR	S	180
1555	LOC Os03g27080.1	AAARIFQAFRVESFHRKKVVEYGDD	S	330			1556	LOC Os03g27460.1	EVRGININEEAQSTDGATRGHVHAR	S	351
1558	LOC Os03g27460.1	RQPQNGSARGGSSRGRVKKTTARKK	S	288			1559	LOC Os03g27780.1	KGGIKALFRRRPSDELTEDQMDRGC	S	334
1561	LOC Os03g27840.1	SDDTSDLKVAGISPPSSPIRVGIPN	S	360			1562	LOC Os03g27840.1	TSDLKVAGISPPSSPIRVGIPNRSS	S	363
1564	LOC Os03g27840.1	TPRVEYITEFGGSDDTSDLKVAGIS	S	348			1565	LOC Os03g27840.1	EYITEFGGSDDTSDLKVAGISPPSS	S	352

1567	LOC Os03g27840.1	YRDPYREQRRSPSYDSYSRGRRSRS	S	295			1568	LOC Os03g27930.1	FHANMNNPVKADSPVRENETAEVVL	S	219
1570	LOC Os03g27930.1	KKKRVRRSKSYHSCQDLAGNISNDI	S	457			1571	LOC Os03g27990.1	EKNEPLTLRPIASGKFNQLRTISII	S	371
1573	LOC Os03g29260.1	EGEGVKVESTACSVSPTADQKAPAA	S	85			1574	LOC Os03g29260.1	EGVKVESTACSVSPTADQKAPAADE	S	87
1576	LOC Os03g29750.1	SCVMLPKSDSFVSVSEYQHPLTEDM	S	420			1577	LOC Os03g30460.1	VNDEYMKTLETLSRKIKFIEVDAMV	S	189
1579	LOC Os03g30790.1	RTPPPPSPSPSLSLSRSQQQTPRGR	S	25			1580	LOC Os03g30790.1	PPPPSPSPSLSLSRSQQQTPRGRQP	S	27
1582	LOC Os03g30790.1	LHRTPPPPSPSPSLSLSRSQQQTPR	S	23			1583	LOC Os03g30790.1	ESPSLHRTPPPPSPSPSLSLSRSQQ	S	19
1585	LOC Os03g30790.1	IALSEAEKNAQVSKILMEQKLMEKD	S	273			1586	LOC Os03g31730.1	SSCKDVHVTTDVSPGISPKGDIPSD	S	601
1588	LOC Os03g31750.1	AAGILTARGGMTSHAAVVARGWGKC	S	468			1589	LOC Os03g32630.1	ASSYMGDIDLPPSDEEEDDADLVAM	S	65
1591	LOC Os03g33110.1	AVASTDVVVVPRSREATPSGPVGDL	S	192			1592	LOC Os03g34300.1	SAVRAGSSTTFLSPPSSPRSGIKNP	S	310
1594	LOC Os03g35340.1	ESNIPSERKFSVSSPEKVMFPDHAD	S	1719			1595	LOC Os03g35340.1	SNIPSERKFSVSSPEKVMFPDHADE	S	1720
1597	LOC Os03g37810.1	KKGGGGGGSSGNSEKKTVAGNVKAA	S	36			1598	LOC Os03g37984.1	EKLRNTAITRANSACLPMEDCVGIK	S	71
1600	LOC Os03g39020.1	NKARKSITSPGLSPSKLSVSDCPGD	S	633			1601	LOC Os03g39170.1	RRQLVAACERMDSFDMAELVLRSGR	S	474
1603	LOC Os03g40010.1	KSREAKSLLNTASPYGTDRPQRERK	S	299			1604	LOC Os03g40010.1	LDFIAEPHAMADSGHSDDQGSNSRK	S	446
1606	LOC Os03g40010.1	DGNKDEAEEEEVSEADKKVEENKEE	S	250			1607	LOC Os03g40010.1	EKQGEEEAEEKGSADNDNEEGDKNA	S	212
1609	LOC Os03g40010.1	ENKNAEEQNKGGSENGTKGPSDGDV	S	58			1610	LOC Os03g40010.1	NKGGSENGTKGPSDGDVEMAEAETA	S	66
1612	LOC Os03g40010.1	IAEPHAMADSGHSDDQGSNSRKRKR	S	449			1613	LOC Os03g40110.1	MAKLKRESKYNLSDDEEDEVDVHLP	S	149
1615	LOC Os03g40110.1	DFGNMSARDWEQSDDDEVTVEEDEM	S	439			1616	LOC Os03g40110.1	KERQKRMLGTAESSDEDDDDDEEGD	S	328
1618	LOC Os03g42220.1	KVKIYGARVRVDSMAKVAEIEAAEK	S	253			1619	LOC Os03g42810.1	VCIITWNMNGKMSVEDVTKLVSSNR	S	64
1621	LOC Os03g43180.1	PTVAMEGGGDGYSGGAARLERRRRR	S	93			1622	LOC Os03g43390.1	GGGEGTKLRRALSFEDAATASASAT	S	73
1624	LOC Os03g43760.1	SDIPEGREKLKRSGSLGSSDTAYVR	S	18			1625	LOC Os03g43760.1	IPEGREKLKRSGSLGSSDTAYVRAD	S	20
1627	LOC Os03g43800.1	QVDADFPEGSLGSREAETGDYPKYA	S	382			1628	LOC Os03g43800.1	SAQIKDENKDNDSDEKPSMPAAVSV	S	522
1630	LOC Os03g43800.1	RSGDRMFLSPVKSTDRKNADSEMGS	S	351			1631	LOC Os03g43800.1	TDDQLEDDREACSGTEEGHSAKKTK	S	438
1633	LOC Os03g44310.1	DHLAPGSPSPAASPSELGDDESWSR	S	104			1634	LOC Os03g44310.1	PPPRHADHLAPGSPSPAASPSELGD	S	98
1636	LOC Os03g44310.1	LAPGSPSPAASPSELGDDESWSRAP	S	106			1637	LOC Os03g44310.1	ELGDDESWSRAPSAAELESNNNDLA	S	119
1639	LOC Os03g44420.1	GAARPRSFQKKDSFLDLHPEVTLLR	S	50			1640	LOC Os03g44484.1	PNRENTMGMRHGSYDKLDDDGLAPP	S	193
1642	LOC Os03g44500.1	ATAARKLLDRQMSINSVPKKVIASL	S	643			1643	LOC Os03g44800.1	NQCSDDDDELPESPAARNGSEEEGS	S	304
1645	LOC Os03g44830.1	QDPRFPGRARTLSSAGTEPTAREAS	S	305			1646	LOC Os03g44900.1	RINSGGISQQLISPLGNKAQPQQVL	S	457
1648	LOC Os03g45180.1	KGSPNPRQIQFLSPSKRDIGRTTSN	S	408			1649	LOC Os03g45180.1	LSPSKRDIGRTTSNSSATQYGWSNL	S	419
1651	LOC Os03g45300.1	EVSPVAAGSGEDSGRGRRTRAVTRV	S	141			1652	LOC Os03g45344.1	RAGATYDLQRNKSNLESLFCYDKSV	S	56
1654	LOC Os03g46330.1	SSGLMGRFSQLLSLDSEEPRSQPTE	S	689			1655	LOC Os03g46340.1	DNSPPLTKHVDASDDDVDKACVSDS	S	413
1657	LOC Os03g46340.1	MLKLRQSEEKVISLSKEKDQLLKER	S	293			1658	LOC Os03g46480.1	GDADEDAEPEQPSSPSVKKAEEKGI	S	241
1660	LOC Os03g46480.1	DEDAEPEQPSSPSVKKAEEKGIVVV	S	244			1661	LOC Os03g46590.1	DGEGPSAEVNETSVEEVNAREDRGV	S	134
1663	LOC Os03g46600.1	RLPSPSLLGRYPSLEELSVAGARLS	S	57			1664	LOC Os03g46710.1	LEEQEILGEADESDDDLAALIRNMD	S	416
1666	LOC Os03g46710.1	NDLASELKDLGWSDADLHDETRTTA	S	327			1667	LOC Os03g46710.1	DLETEQSESKVPSPQGHRSTRTEDI	S	554
1669	LOC Os03g46920.1	ADQAEPVVEKTLSGSGSSEGHLRVK	S	656			1670	LOC Os03g46920.1	QAEPVVEKTLSGSGSSEGHLRVKGS	S	658
1672	LOC Os03g46920.1	PVVEKTLSGSGSSEGHLRVKGSQLQ	S	661			1673	LOC Os03g47760.1	RGQKNTGLTLQDSLPSSPRETTPYQ	S	1051
1675	LOC Os03g47930.1	VHSPVSTTNLSKSQSFDSPTEKELT	S	146			1676	LOC Os03g47930.1	SPVSTTNLSKSQSFDSPTEKELTID	S	148
1678	LOC Os03g48140.1	ENKVLRQQAVAISPTAKSLAAYPKS	S	1100			1679	LOC Os03g48600.1	EITEYRQSQPRRSAELQGIAREAMS	S	263
1681	LOC Os03g48600.1	PVQCRVTSLENMSPESEPQRTLLSR	S	186			1682	LOC Os03g48930.1	GAREYARRAYTDSPPEAATSEAATV	S	198
1684	LOC Os03g49170.1	LRVVTRAANSMLSPVSSPTAEDARS	S	226			1685	LOC Os03g49170.1	TRAANSMLSPVSSPTAEDARSPSAA	S	230
1687	LOC Os03g49170.1	SPVSSPTAEDARSPSAAVMMTTKFA	S	238			1688	LOC Os03g49500.1	GLEECALWMPSRSGSSLQLSHTLRH	S	187
1690	LOC Os03g49640.1	SEVRTDDERVDASHMSDTEEEEEFA	S	309			1691	LOC Os03g49640.1	RTDDERVDASHMSDTEEEEEFADEG	S	312
1693	LOC Os03g49770.1	SSGSDFQRSEAGSDDEDDYPMDGID	S	385			1694	LOC Os03g49770.1	ENIYFDTRDFLSSSSFKSSGSDFQR	S	368
1696	LOC Os03g50120.1	SFDDSFDREDDGSDPDEGERRRAKV	S	427			1697	LOC Os03g50120.1	SIHEDGNGREDDSEDVLPYEGQRRH	S	471
1699	LOC Os03g50120.1	TRGADRSRSFDDSFDREDDGSDPDE	S	419			1700	LOC Os03g50290.1	MQLLRDNLTLWTSDNAEDGGDEIKE	S	239
1702	LOC Os03g50300.1	SRGPKKYAEDVESGSSDVETKYKKM	S	563			1703	LOC Os03g50330.1	LYGSDDGQPRKRSEDIILSRAQNGF	S	101
1705	LOC Os03g50330.1	LLNHKSAEDPDGSFYTGDDPDRSFY	S	49			1706	LOC Os03g50340.1	PIAVEKGWSESASPNVRGPPALVFT	S	989
1708	LOC Os03g50390.1	SNSSINEESDGPSPKLSDWSRRGSA	S	656			1709	LOC Os03g50390.1	PRKRVPADIKSFSHELNSKGVRPFP	S	277
1711	LOC Os03g50390.1	DEEDDVVRSLRASPVHPVKDRTSID	S	839			1712	LOC Os03g50390.1	PKHQHKTSWGHWSDQPNISEEGSIM	S	563
1714	LOC Os03g50480.1	GSKATGAFILTASHNPGGPTEDFGI	S	124			1715	LOC Os03g50730.1	ANGGGKKLEKEDSLDWERYMKENGN	S	170
1717	LOC Os03g51000.1	PAKIPVPVPPSPSGKDDEILTEPQE	S	394			1718	LOC Os03g51000.1	RPPAKIPVPVPPSPSGKDDEILTEP	S	392
1720	LOC Os03g51020.1	ETWFLAHEIDYPSDNEKATGHTSGP	S	423			1721	LOC Os03g51020.1	PFVEDDQHGSVRSIGVGISSDAADI	S	553
1723	LOC Os03g51020.1	SNAGPRGAASVRSADSSREFIVKEI	S	72			1724	LOC Os03g51020.1	GPRGAASVRSADSSREFIVKEIDVG	S	75
1726	LOC Os03g51020.1	KEDDEQDGSEAGSNAGPRGAASVRS	S	60			1727	LOC Os03g51030.1	ADSCHRQLNKILSDLDQDSVMNKSS	S	951
1729	LOC Os03g51140.1	AGTSGKAILEGGSDDEGASTEAHGR	S	399			1730	LOC Os03g51220.1	RKDKDKTQDSIDSEVEGINKKGETV	S	637
1732	LOC Os03g51250.1	PAAAPEKTSRAGSFSFKSGPPKLEL	S	325			1733	LOC Os03g51390.1	TVPLPEYERLSQSARLPDDADPLMK	S	84
1735	LOC Os03g51520.1	ESFVKQAEEDPMSDDENHEDSRRGR	S	1230			1736	LOC Os03g51520.1	DEGSESKDGPEGSPAAADEAADKLS	S	691
1738	LOC Os03g51520.1	LISRAEQPTGHNSSDQDELRTRNSD	S	766			1739	LOC Os03g51600.1	ALEKDYEEVGAESDENEDGDDGDEY	S	439
1741	LOC Os03g52310.1	FAYSSGSKIAGTSHLKRHITLGSCP	S	108			1742	LOC Os03g52310.1	CCKLCKQTFAYSSGSKIAGTSHLKR	S	100
1744	LOC Os03g52490.1	DRYQRVERPRPESAIEENEIRITAQ	S	14			1745	LOC Os03g52700.1	PGKGVSRWRSDESSEDEDDKRTSGY	S	885
1747	LOC Os03g52700.1	QKTKVPPKENVLSFGKKSSVDEAPP	S	861			1748	LOC Os03g52794.1	ETSAKSMLAEASSLAEKSRTSFELH	S	2695
1750	LOC Os03g52980.1	KIMHNKQYNMSKSIEDEYDFGDAPS	S	499			1751	LOC Os03g53270.1	PLNELKPVPRVDSQGQMCGSNYKVD	S	217
1753	LOC Os03g53280.1	LPKFGDDMSSIGSHGDLDDFSAQKD	S	1527			1754	LOC Os03g53280.1	DDLTLPKFGDDMSSIGSHGDLDDFS	S	1523
1756	LOC Os03g53280.1	SYSDGRQLTASPSMSSTISGIDLDS	S	2111			1757	LOC Os03g53280.1	TERKDLDAADFASFKDEDDIFKGGS	S	2668
1759	LOC Os03g53630.1	SGGVVPKKQEDSSESEEPIILRKNH	S	458			1760	LOC Os03g53630.1	GVVPKKQEDSSESEEPIILRKNHQR	S	460
1762	LOC Os03g53630.1	GDELSMGLSRSASGRESIEQEDTVM	S	65			1763	LOC Os03g53630.1	AIPSLKKARDTSSDTELEQPKMSSG	S	366
1765	LOC Os03g53670.1	KRAKQQLLQGQGSLDNASHEKEKNA	S	610			1766	LOC Os03g53670.1	GSASAGDAQNAASPVDRSITPLLQE	S	54
1768	LOC Os03g53880.1	ANGRHDILTNMESRSSAVLGNDDKA	S	175			1769	LOC Os03g53960.1	PGDLTPKDESPESPPRVKVEDALGE	S	206
1771	LOC Os03g54780.1	KIRAKVAAETELSGPDSDATVRIND	S	546			1772	LOC Os03g54780.1	QQQQNSRRKPSVSSVPESVTREDPS	S	246
1774	LOC Os03g54780.1	HPDMQEAKTMPPSTGTVKKLKVAEG	S	662			1775	LOC Os03g54890.1	VDARHHMLGRLASIIAKELLNGQRV	S	26
1777	LOC Os03g55300.1	HLAGGNKAKALLSDGDDDGELGQRM	S	172			1778	LOC Os03g55330.1	DSPAESYLESRVSFSEERVMDADSK	S	130
1780	LOC Os03g55450.1	LQSARNVGAVVDSGDGDFARERAVA	S	118			1781	LOC Os03g55570.1	HIWPLNSDGPVTSASPERSTPKEKP	S	220
1783	LOC Os03g55704.1	KTSPKVLSKLRRSPIPDNHVVSFDA	S	682			1784	LOC Os03g55704.1	EAERVSSMGRKVSFSMEDSRLCRNS	S	826
1786	LOC Os03g56060.1	LAPKELKHQKTESAPNLDAIAKEQS	S	382			1787	LOC Os03g56430.1	KSLSMQIVPHKNSQGFSTPNSSSVL	S	293
1789	LOC Os03g56800.1	DGHRVKKYRGMGSLEAMTKGSDARY	S	405			1790	LOC Os03g56900.1	MDTIAENLPKQKSGKFDVGAASDKM	S	29

1792	LOC Os03g57160.1	KIYGSTKADSVESGEKIGGHAVGHA	S	776			1793	LOC Os03g57160.1	LGQAQNHAMRSDSAASLYMDDHVTP	S	914
1795	LOC Os03g57160.1	LGRDLMIGQPTGSEVGDHATSYESR	S	457			1796	LOC Os03g57160.1	GSEVGDHATSYESRGPSSRKRNHEE	S	468
1798	LOC Os03g57160.1	RWLPNVQDCEENSTHSAKNADSVEP	S	284			1799	LOC Os03g57160.1	PNVQDCEENSTHSAKNADSVEPFFP	S	287
1801	LOC Os03g57310.1	LDDIETHVGRARSFVDRGREQLVVA	S	262			1802	LOC Os03g57430.1	LRKDGSSKLLSHSFKDLGNKDIRSD	S	344
1804	LOC Os03g57430.1	KHSLDPELNGSPSSKDKLRKDGSSK	S	327			1805	LOC Os03g57430.1	HSLDPELNGSPSSKDKLRKDGSSKL	S	328
1807	LOC Os03g57430.1	SDLTDGGSPIRDSPSSKRLLLGTGQ	S	209			1808	LOC Os03g57430.1	TDGGSPIRDSPSSKRLLLGTGQSVE	S	212
1810	LOC Os03g57430.1	ISSDNVLAASPQSNKMAEKIFEQLN	S	249			1811	LOC Os03g57854.1	SGGKSQSVWGQLSDGGDTSGRRVRP	S	417
1813	LOC Os03g58250.1	KIGRTASLQRVASLEHLQKRMCGGP	S	406			1814	LOC Os03g58480.1	TRAKSPKFTRRRSCSDAPPTPEAAN	S	306
1816	LOC Os03g58480.1	KLIDQEKSGPPKSPSKPGISGSDRS	S	110			1817	LOC Os03g58480.1	IDQEKSGPPKSPSKPGISGSDRSKR	S	112
1819	LOC Os03g58590.1	LRKHLHRLEHPDSIEQDKESEDEID	S	408			1820	LOC Os03g58740.1	PKPAPSRLESSLSFSAPLHKVEGRA	S	254
1822	LOC Os03g58810.1	FKNPPKVNIDLESSASKHRRKMRKV	S	550			1823	LOC Os03g59020.1	IQSMLKEGHKHLSGLDEAVLKNIDA	S	24
1825	LOC Os03g59250.1	PFVSDNRHSMSLSPEKDRYYSTRGS	S	94			1826	LOC Os03g59340.1	KIAERMLTWRMNSGRNDDIVHSKYD	S	112
1828	LOC Os03g59390.1	AEQVLEHPWLHDSKKMPDIPLGDAV	S	325			1829	LOC Os03g59390.1	SRQYSRQRFSNLSLKLQKDGSISDD	S	524
1831	LOC Os03g59480.1	DFLDVEAEEEDDSDDDMMRFKDNEE	S	463			1832	LOC Os03g59680.1	GGHKHHQDKHDVSPSSDLVRKSKRI	S	201
1834	LOC Os03g59680.1	FKDLSCTPSRSISPEDDAGVEGSGH	S	49			1835	LOC Os03g59760.1	DDDDDDEDDEDDSDHELEDLFRRQR	S	96
1837	LOC Os03g60180.1	MVSSVSRGGRSNSMVSGITREGKSQ	S	166			1838	LOC Os03g60240.1	DNSRHDVQLQSSSPCREELETVKAP	S	929
1840	LOC Os03g60460.1	QVGRMKYLRPLYSSLARCSGEEKML	S	571			1841	LOC Os03g60460.1	MEPESAIYKKICSLAAEFKSGKLPS	S	476
1843	LOC Os03g60620.1	VFDAKRLIGRRFSDASVQSDIKMWP	S	83			1844	LOC Os03g61010.1	GIQHDKENTERISLSAIAKVYARAD	S	335
1846	LOC Os03g61160.1	PRAAANVVSPLASPRAAGDISPLAA	S	75			1847	LOC Os03g61160.1	GDEKKVRLMRNRSLTREEVDAFWRR	S	26
1849	LOC Os03g61160.1	PRAAGDISPLAASPGRAQQEMSSSR	S	88			1850	LOC Os03g61160.1	CTLALRRLERMNSMPSPLARTVMTR	S	113
1852	LOC Os03g61220.1	GGRSFRSDNDSWSDDDFGGGRRSNR	S	691			1853	LOC Os03g61220.1	GGGSRGGRGGWDSDGEDRFRRGGRS	S	670
1855	LOC Os03g61560.1	VPSLISKDEASESAAEDIPENKVDV	S	463			1856	LOC Os03g61640.1	RFESLKKRKDPGSFSEQDLDERILK	S	139
1858	LOC Os03g61890.1	PASKGRRFGLGDSDDEFHDDDVEEE	S	420			1859	LOC Os03g61930.1	GTSSVLEPEVELSHDGKIREIAMDS	S	510
1861	LOC Os03g61930.1	QSKTKEHASDGTSSVLEPEVELSHD	S	500			1862	LOC Os03g61930.1	SKTKEHASDGTSSVLEPEVELSHDG	S	501
1864	LOC Os03g62340.1	SFGAKGLIRRSSSFSSDIQKDVSVC	S	426			1865	LOC Os03g63140.1	NHHGDTEALSKASPLHAGSDPAMRK	S	55
1867	LOC Os03g63320.1	AARARVSGSRDASGDESDGVADWGL	S	168			1868	LOC Os03g63320.1	RVSGSRDASGDESDGVADWGLPNGR	S	172
1870	LOC Os03g63320.1	AEHYYDDADQSDSAAAAAARARVSG	S	151			1871	LOC Os03g63650.1	ESFYSQVGSTRGSPAATPSPAPLTP	S	175
1873	LOC Os03g63670.1	RSANNEHIEGELSTDESDSESSAYL	S	615			1874	LOC Os03g63670.1	NEHIEGELSTDESDSESSAYLSSRD	S	619
1876	LOC Os03g63940.1	MDVDNDNFQRTVSLSDGIIQEGPQR	S	136			1877	LOC Os03g63940.1	VDNDNFQRTVSLSDGIIQEGPQRIS	S	138
1879	LOC Os03g64210.1	IQARIAQLKRELSQTDSAYDSEKLA	S	394			1880	LOC Os03g64210.1	PRGRNVVLDEFGSPKVVNDGVTIAR	S	86
1882	LOC Os03g64400.1	DSSPSPDAFAGWSDGDEQDEQDKSP	S	56			1883	LOC Os04g01480.1	GAQHPMWKTSLCSFFRRRAASSADG	S	58
1885	LOC Os04g01490.1	VKFFNARISRTSSLPVTDVSQEQAD	S	156			1886	LOC Os04g01490.1	KLNNGKSFKRMNSLGGVYRVVPSTP	S	206
1888	LOC Os04g01490.1	STSVAPKPQRSSSFMLRQTVKSLLP	S	124			1889	LOC Os04g01490.1	VQNLPVTLVRVQSMQQPELQTNPAN	S	314
1891	LOC Os04g02150.1	SDDIEAMKRDGVSGDEIVEALIANS	S	148			1892	LOC Os04g02870.1	RSRPSPDYGRQRSPVYGSYDRSPVN	S	210
1894	LOC Os04g02870.1	GYGRRDDSPYRRSVSPVYRSRPSPD	S	192			1895	LOC Os04g02870.1	GRRDDSPYRRSVSPVYRSRPSPDYG	S	194
1897	LOC Os04g02870.1	DGARGGYGRRDDSPYRRSVSPVYRS	S	187			1898	LOC Os04g02870.1	RRSVSPVYRSRPSPDYGRQRSPVYG	S	202
1900	LOC Os04g06770.1	FLKFEDMSDASSSQGGHTSVGSVPV	S	882			1901	LOC Os04g06770.1	TFLKFEDMSDASSSQGGHTSVGSVP	S	881
1903	LOC Os04g08310.1	IQHKKDAQEASTSVEDEGFKNVGGN	S	112			1904	LOC Os04g08410.1	CYQNRMTPNNIDSDDEDETEFGFLG	S	497
1906	LOC Os04g08450.1	CSSRVLSPPRYESGDEELSVLPRHT	S	20			1907	LOC Os04g08740.1	HNQDWMLRRANFSGSYVCVKFEIRI	S	538
1909	LOC Os04g08740.1	VMQTSTVNREYLSLVRRAFNLHSLV	S	443			1910	LOC Os04g14510.1	AIHKDKNYVRTLSICGDKVDGVEHS	S	219
1912	LOC Os04g15800.1	EDVMEGMRLDFSSPLGKIDSDEEGE	S	284			1913	LOC Os04g16360.1	LNTKNQYDFRTKSIGGWSGYVAAYV	S	37
1915	LOC Os04g17700.1	RPAGPLPSDVEDSDDSNAGDGAGEA	S	56			1916	LOC Os04g17700.1	GPLPSDVEDSDDSNAGDGAGEALRK	S	59
1918	LOC Os04g18010.1	SNVKRVPLSPQGSADHHNASDSRLR	S	360			1919	LOC Os04g18090.1	GASLPANYKKMLSIQLRGFASKGKL	S	80
1921	LOC Os04g24170.1	LETEVLQSQQVSSPAQSKTGSSVHE	S	458			1922	LOC Os04g24210.1	IKKFVSQLGVMTSTSRPMDLYCDNS	S	620
1924	LOC Os04g24520.1	RNPSLPVLIRECSGVEPQLWARYDK	S	54			1925	LOC Os04g25550.1	ASCSKLVKDVAYSFNDEDEVLPVKK	S	446
1927	LOC Os04g28090.1	GSTATRALLSSYSQTPRLGMTPLRT	S	340			1928	LOC Os04g28090.1	QCKARWYEWLDPSIKKTEWTREEDE	S	56
1930	LOC Os04g28210.1	LKSNKFVGNVGPSVPGDKNSCEFIK	S	782			1931	LOC Os04g28420.1	MKDELDEEGDSSSPVAMKVGEEKEI	S	34
1933	LOC Os04g28460.1	AVYTGNQTKSGMSRGTAEPKLTAAD	S	241			1934	LOC Os04g30780.1	LVSAFMTELEPESPVSQCDFDRLKL	S	217
1936	LOC Os04g31190.1	GSSSSKSVARVHSLDNLISIIQLRE	S	607			1937	LOC Os04g31330.1	SKNAADDMIRILSGFDHRFSSITAD	S	59
1939	LOC Os04g31330.1	GLFCSLRRLSLESMDDLDTSSEFDP	S	194			1940	LOC Os04g31340.1	GSPVANLVSRLGSFTFRRTSSGRVE	S	137
1942	LOC Os04g32950.1	KPKSTESEAAGSSGGKEDEKEEEEK	S	511			1943	LOC Os04g32950.1	PVAEVKKPKSTESEAAGSSGGKEDE	S	505
1945	LOC Os04g33110.1	THAGSFKCRLHRSSSHGHPSSSPTA	S	77			1946	LOC Os04g33110.1	LHRSSSHGHPSSSPTAAAAPAAAVQ	S	86
1948	LOC Os04g33110.1	HSSGTTPTGAAASPKMRRSWSSAAS	S	33			1949	LOC Os04g33110.1	GAAASPKMRRSWSSAASASSGGGGH	S	41
1951	LOC Os04g34450.1	RGGGRAGARRAASRDDGDFDDDEPR	S	118			1952	LOC Os04g34450.1	KSLRLITDASVSSPVSREKAQGLDP	S	205
1954	LOC Os04g35130.1	TDSEVTSSMAASSPPRAAYYVQSPS	S	172			1955	LOC Os04g35170.1	VAKEEDKFGQSSSFKICDLNLVGSP	S	547
1957	LOC Os04g35260.1	LVDSGATGKKCESPVHMDGGSSLEK	S	295			1958	LOC Os04g35700.1	AGAASAASSPRKSLDLVDLSASPSV	S	34
1960	LOC Os04g35700.1	VRTNMSLPVSPCSSPLRQFKQSNWS	S	641			1961	LOC Os04g35700.1	RTNMSLPVSPCSSPLRQFKQSNWSC	S	642
1963	LOC Os04g35800.1	PSPPLPPRKRRLSPTPSPTRRSSRS	S	54			1964	LOC Os04g35800.1	LPPRKRRLSPTPSPTRRSSRSRSRS	S	58
1966	LOC Os04g35800.1	SRSRSRSRGRSASPRYPDGKRRRHN	S	90			1967	LOC Os04g35910.1	KTSKVGAEVSDRSPKHKRDSGEGAE	S	226
1969	LOC Os04g36790.1	QMELSQVKDHSSSPANAAAPPGATG	S	326			1970	LOC Os04g36790.1	PGGGGGGREDAWSEGATAALIDAWG	S	69
1972	LOC Os04g36890.1	KLQLVVRTPPAESLESEDEDGFPVS	S	228			1973	LOC Os04g36890.1	VGGVEKEIKQEDSPADLVDAKQKKN	S	317
1975	LOC Os04g37540.1	VNTAKESTSNPASPVLGRSYSVEPL	S	374			1976	LOC Os04g37950.1	DILEKESNAAKDSPQSSSPEKVPNG	S	140
1978	LOC Os04g38260.1	EEIARNQRASEVSDNDGYEEFNDRA	S	120			1979	LOC Os04g38480.1	ELAPRHNDWIVDSTYNLRAMELSGP	S	615
1981	LOC Os04g38570.1	RSGSLRNLSYSYSTGADGRIEMVSN	S	654			1982	LOC Os04g38620.1	PYFEDTKLTKTYSFSDDEAVKVKAT	S	141
1984	LOC Os04g38630.1	RENEEDNDVEQASDDNAKNDSEDGY	S	693			1985	LOC Os04g38870.1	LRDNLTLWTSDISEDTAEEIREAPK	S	243
1987	LOC Os04g39040.1	PSGFPGGGGGGGSDSDEPQEYYTGG	S	46			1988	LOC Os04g39040.1	GFPGGGGGGGSDSDEPQEYYTGGEK	S	48
1990	LOC Os04g39090.1	TSALPSRIPSARSMDDLLKLDSSRE	S	174			1991	LOC Os04g39240.1	VYSVPCPVEKTDSFSDIVYGALRGF	S	330
1993	LOC Os04g39629.1	DVNKSHARRRTHSPDMHHQRKRSRE	S	181			1994	LOC Os04g39629.1	DSPLDVRLQRTNSDVRVPGVTAAAL	S	261
1996	LOC Os04g39629.1	YDREAAKGTDSASDIDRHDDTGAAR	S	359			1997	LOC Os04g39980.1	EFKYDDYRRLRLSTGERAGEALARL	S	286
1999	LOC Os04g40080.1	AIVAATDAEAGGSRRVPEGDLEACR	S	56			2000	LOC Os04g40400.1	PQIVVSIVDRPRSRGGMEPFPKPAE	S	491
2002	LOC Os04g40400.1	SRDKKPMPSRYPSLGSGGGGGGGFR	S	212			2003	LOC Os04g40430.1	SLPREPKPEIDDSDNSDDPDFWVPP	S	185
2005	LOC Os04g40430.1	AAEAAAPDAGVSSPQGGGGEKEGSF	S	21			2006	LOC Os04g40430.1	RWDDDDGKDRWTSSPVKDKDDAAED	S	213
2008	LOC Os04g40720.1	EYKDPTMGDMVNSSEVVAGAEKSTK	S	388			2009	LOC Os04g40720.1	CCRDTSMEDRGKSSEGCPGAEVHVD	S	127
2011	LOC Os04g40720.1	KDKGTGTIFNDASSDGSDDELGIGS	S	1299			2012	LOC Os04g40720.1	TGTIFNDASSDGSDDELGIGSEKAA	S	1303
2014	LOC Os04g40720.1	EPNEKINFLDHFSPKGTNDQYVSAE	S	1015			2015	LOC Os04g40720.1	SRVKKLNQSKSGSDNQGNLPIDKDH	S	1189

2017	LOC Os04g40720.1	YPAAIHSDAPISSPSHNKARGEKLE	S	749			2018	LOC Os04g40720.1	AAIHSDAPISSPSHNKARGEKLEVL	S	751
2020	LOC Os04g40874.1	MSGGSSPRSRRSSFNSLSRDLELPS	S	13			2021	LOC Os04g40940.1	KLIENPQMVKLHSFDTKIHKVDTLV	S	185
2023	LOC Os04g41030.1	IPDSLGRLDRLKSLDLSDNRLDGQI	S	178			2024	LOC Os04g41030.1	KSKGGRRRRRGGSESGGGSAEDGSW	S	265
2026	LOC Os04g41100.1	SRDGESVVAASASSPSKKRKFSPII	S	131			2027	LOC Os04g41100.1	RDGESVVAASASSPSKKRKFSPIIW	S	132
2029	LOC Os04g41100.1	DEEEGAPHRKKKSASPADSAELGQR	S	253			2030	LOC Os04g41100.1	EEGAPHRKKKSASPADSAELGQRKK	S	255
2032	LOC Os04g41100.1	QDHIPERLAVEKSPMDVEPAVASES	S	193			2033	LOC Os04g41100.1	DSAELGQRKKALSPELGEVVASDIS	S	270
2035	LOC Os04g41100.1	SPELGEVVASDISGGRTMSRSSDSG	S	282			2036	LOC Os04g41100.1	NELDRRTKRYLKSPDPLEEQRLKEL	S	687
2038	LOC Os04g41100.1	QSGMDSEYEVRRSETPEPVKPPHRC	S	340			2039	LOC Os04g41100.1	ASASSPSKKRKFSPIIWDRDSPKPM	S	140
2041	LOC Os04g41820.1	SLSSRDVAAMPDSPPRRAARHRRAQ	S	131			2042	LOC Os04g42010.1	KIPENSDIKEDLSDDVEKSKEAAAQ	S	280
2044	LOC Os04g42010.1	AAAQSVKKGESPSENADDPISREDF	S	301			2045	LOC Os04g42030.1	DVREEVETEEVESDDESEKYWDEQW	S	497
2047	LOC Os04g42320.1	DHNLKKSHKLDTSTDSEVVDHSKAV	S	393			2048	LOC Os04g42320.1	LKKSHKLDTSTDSEVVDHSKAVNNN	S	396
2050	LOC Os04g42320.1	STKRKQKEKEGESSEEEEHVSAKIS	S	823			2051	LOC Os04g42320.1	TKRKQKEKEGESSEEEEHVSAKIST	S	824
2053	LOC Os04g42320.1	DTPPDEDTDEDLSLKDIVSPKSSAK	S	539			2054	LOC Os04g42320.1	DTDEDLSLKDIVSPKSSAKTGKNKG	S	545
2056	LOC Os04g42320.1	ATRRLAKDDDIKSSFKKTGEGESSK	S	503			2057	LOC Os04g42320.1	FEDALDAGVADNSDAPGKDMVADGK	S	272
2059	LOC Os04g43130.1	AGTDGCITSPMQSSSPKVRPDQEYL	S	345			2060	LOC Os04g43140.1	RNAESMGLLLETSDSEEERVQGHKQ	S	688
2062	LOC Os04g43300.1	QSSDGPRDLDCDSNDLEGELITSRS	S	197			2063	LOC Os04g43300.1	ANDDNRELKRQKSTDQDDRQPAVAG	S	234
2065	LOC Os04g44640.1	GGGHRRYSGSKRSYDFDRERRGGGG	S	165			2066	LOC Os04g44740.1	SFGIHLWNRESRSLEMEEGSVIGRL	S	435
2068	LOC Os04g44800.1	GEAAAAVAARSRSKSIEADEEERSK	S	25			2069	LOC Os04g45190.1	LKVVYVQPPRPPSPVREGSEEGSSP	S	135
2071	LOC Os04g45190.1	PPSPVREGSEEGSSPRASLSEGGNL	S	145			2072	LOC Os04g45190.1	PSPVREGSEEGSSPRASLSEGGNLN	S	146
2074	LOC Os04g45190.1	RAHKDAEDVTSDSETSALISRLTEE	S	188			2075	LOC Os04g45570.1	AVRGPRPLQPAASASFRRRDRWFHL	S	44
2077	LOC Os04g45800.1	GSRRSQKSAGQQSSPTVFPEKKGKS	S	35			2078	LOC Os04g45800.1	SRRSQKSAGQQSSPTVFPEKKGKSK	S	36
2080	LOC Os04g45930.1	EAPKVEEQKRQPSPPLQQPAAEEKA	S	55			2081	LOC Os04g46190.1	LYPQDRKKESVASPSSTVESKKLSK	S	66
2083	LOC Os04g46320.1	SSPNARDIHLPSSFKDLVLERKISD	S	307			2084	LOC Os04g46320.1	SSFKDLVLERKISDAFSSELVKQSK	S	318
2086	LOC Os04g47380.1	KRGSTDDDKQILSESDAFINKNLEG	S	97			2087	LOC Os04g47380.1	GSTDDDKQILSESDAFINKNLEGIQ	S	99
2089	LOC Os04g47700.1	ANFAPVSEKRNSSIKDDENIELDID	S	614			2090	LOC Os04g47870.1	AFRARFYMEPETSDSGSMASGAATS	S	1071
2092	LOC Os04g47912.1	NPCEAYCGRSTSSLDEFEVRSAMAV	S	207			2093	LOC Os04g48160.1	KRWGFGKSFREKSPAHPPPPPPPSA	S	44
2095	LOC Os04g49060.1	VLLQDRVAELIGSCSPGNQFNDADS	S	164			2096	LOC Os04g49060.1	LQDRVAELIGSCSPGNQFNDADSSD	S	166
2098	LOC Os04g49510.1	DTDDNNNAAAADSPKKPSRPPAAAK	S	37			2099	LOC Os04g49510.1	NACGGSLRSKYLSFKQTASQRHDTD	S	15
2101	LOC Os04g49520.1	IMMNGYAKQGSLSDAEELFDRMPRR	S	88			2102	LOC Os04g50204.1	KDGATNGSHKEESNVKGDSEDDEPV	S	668
2104	LOC Os04g50890.1	ATVDVPLDTMGDSKSKARELSSWET	S	63			2105	LOC Os04g51040.1	LEALRRLMKQHLSLKSEKALRELME	S	695
2107	LOC Os04g51060.1	LLQRYAKRQSPGSDACDPYSIQASK	S	367			2108	LOC Os04g51080.1	RAVATTDGGGADSFSDFARAPSRSY	S	82
2110	LOC Os04g51090.1	GSQSNPDRASEWSGCSDDEQETGGS	S	2565			2111	LOC Os04g51090.1	SNPDRASEWSGCSDDEQETGGSNVV	S	2568
2113	LOC Os04g51120.1	ETAAKDPFDFSSSNLGKTPLADPKA	S	448			2114	LOC Os04g51120.1	SSTGITYKSSSASFGSNYSSGERYG	S	178
2116	LOC Os04g51370.1	NPGGGHWLAAGLSPQVGSVNRSLPP	S	507			2117	LOC Os04g51370.1	HWLAAGLSPQVGSVNRSLPPNNPYP	S	512
2119	LOC Os04g51700.1	QHAKIDDVEPEESDHGETGLDDQIP	S	879			2120	LOC Os04g51700.1	RGKQHAKIDYGQSEESDPGETGQDD	S	933
2122	LOC Os04g51710.1	SGPLSNAPSRNASFGGAASNSGPVP	S	74			2123	LOC Os04g51710.1	SRNASFGGAASNSGPVPNAGDRSNY	S	82
2125	LOC Os04g51710.1	QRKVSGPLDSAASKKTRATSFSHNQ	S	185			2126	LOC Os04g51710.1	LDSAASKKTRATSFSHNQAVTKITT	S	192
2128	LOC Os04g51710.1	PLNKHGEPVKRSSGPQSGGVTPMAR	S	129			2129	LOC Os04g51710.1	HGEPVKRSSGPQSGGVTPMARQNSG	S	133
2131	LOC Os04g52050.1	GRAAASAAAEGGSPEPLEVRCAGCG	S	16			2132	LOC Os04g52050.1	IDRIINSICPSSSPRHMTPEKVSNE	S	522
2134	LOC Os04g52050.1	DIDRIINSICPSSSPRHMTPEKVSN	S	521			2135	LOC Os04g52074.1	PIERAIDDTYHISPNQHRYPQSVSN	S	397
2137	LOC Os04g52074.1	PHLRRSQRLTKGSPDPIDIEPIHRI	S	360			2138	LOC Os04g52120.1	LIPNQVHSEKRMSSFRLKLPTPELE	S	223
2140	LOC Os04g52180.1	KSKSFLKPIGSISKKKVQLHLKIKK	S	93			2141	LOC Os04g52180.1	NNKSKSFLKPIGSISKKKVQLHLKI	S	91
2143	LOC Os04g52460.1	KEAHNDEDDGTGSDGFELIDVKENF	S	57			2144	LOC Os04g52540.1	FHYDIDIKLDISSPKASDKELSKGD	S	237
2146	LOC Os04g52540.1	ELFKDESFRRLSSAVAYDGKRNLFT	S	268			2147	LOC Os04g52570.1	PKLTPGSTPKFLSGPTSPTKSLSEV	S	201
2149	LOC Os04g52570.1	LEAFKKQLMKSLSEDNLLQLSEIGD	S	124			2150	LOC Os04g52920.1	WASKEELELVSASPSIADLERMKKE	S	403
2152	LOC Os04g52940.1	GRPTTLQDRGRDSDEEDFRDRDYDV	S	507			2153	LOC Os04g52960.1	TNSSSSDDSSDESLSDDEPVKKPAA	S	149
2155	LOC Os04g52960.1	RDNSSFKKPAQSSGNTIFIKGFDTS	S	547			2156	LOC Os04g52960.1	PLKKPVALATNGSKKVETDSSSSDS	S	174
2158	LOC Os04g52960.1	RGAYTPGSGRDNSSFKKPAQSSGNT	S	538			2159	LOC Os04g52960.1	GAYTPGSGRDNSSFKKPAQSSGNTI	S	539
2161	LOC Os04g52960.1	SAVEVAPTSVSVSEGKSGKKGKRNA	S	20			2162	LOC Os04g53350.1	LVHHQTLKNEISSPISVLERKSGEL	S	154
2164	LOC Os04g53670.1	RNLGSGGIDNTFSPRKLSRQNSFGS	S	1118			2165	LOC Os04g53670.1	NTFSPRKLSRQNSFGSIGTPRTPNL	S	1127
2167	LOC Os04g53700.1	WWDSVALPEDVDSCDEDTFRQQIRD	S	338			2168	LOC Os04g54340.1	GGRSVTAQSNLNSFSDDEDTREMLL	S	537
2170	LOC Os04g54440.1	MAPKKRKADPAESPVASSEAGAGTN	S	13			2171	LOC Os04g54670.1	GFDGGEIFGGSSSPVVDPSENQQER	S	672
2173	LOC Os04g54820.1	SSSSLLNKSHEVSDDDFDLDALANA	S	138			2174	LOC Os04g54940.1	KSLQANRLSSANSADGGPCVGKVNV	S	166
2176	LOC Os04g55220.1	KEETPNPFRQQFSMTSLERTMTSME	S	406			2177	LOC Os04g55500.1	KFSGVSCPISLDSDDELDDTVYRES	S	185
2179	LOC Os04g55960.1	TFFSVHADAEDGSPRKGGGSLAPPF	S	369			2180	LOC Os04g55960.1	DKSDVSFQDKSWSLANGSGVIDIHH	S	280
2182	LOC Os04g56140.1	AEPEIHMESDINSASELRTKDTGPH	S	464			2183	LOC Os04g56140.1	ESRRSRLGSSGSSPDASKVRQYGST	S	334
2185	LOC Os04g56140.1	LRTKDTGPHIHASLNGSTKKTSANG	S	480			2186	LOC Os04g56160.1	PSSALLGQNKDASLEALPVDELIEK	S	545
2188	LOC Os04g56290.1	KLELSAVPSSYYSGQLDPDRVLQDS	S	324			2189	LOC Os04g56350.1	RKRAKSADRLSVSDGDDVSSEGTII	S	453
2191	LOC Os04g56440.1	LDAIARLDDASDSESEDMAVGAQMV	S	375			2192	LOC Os04g56530.1	SKTRLDHHHSSGSLQSLQADADRHH	S	813
2194	LOC Os04g56530.1	SVTQNGSTPRRRSGQLDPPMLEISK	S	790			2195	LOC Os04g56530.1	STVRDIDEDDEGSSGDNHSGFSGPP	S	304
2197	LOC Os04g56630.1	DQADSRSVDSAKSTDDDEEDEDEIE	S	482			2198	LOC Os04g56630.1	VSSADHSDGTGLSSPKGALLGLASY	S	584
2200	LOC Os04g56630.1	PKGALLGLASYDSDDEDDEGDNEDK	S	598			2201	LOC Os04g56640.1	LEDQGVRQLYPKSPDIDFKKELRTL	S	66
2203	LOC Os04g56720.1	SESEEDDDEEDDSDDDENGEIKGGK	S	477			2204	LOC Os04g56740.1	KILACAGGGSDPSAGSDGDADDHVD	S	22
2206	LOC Os04g56740.1	LKFEELTSNLTVSRSNSLDGANKPK	S	565			2207	LOC Os04g56740.1	ELTSNLTVSRSNSLDGANKPKVHTP	S	569
2209	LOC Os04g56740.1	RAKASASVSPKLSPDVQDNNPRKRH	S	836			2210	LOC Os04g56740.1	QFTESARAKASASVSPKLSPDVQDN	S	830
2212	LOC Os04g56740.1	FAAAQLKFEELTSNLTVSRSNSLDG	S	560			2213	LOC Os04g56740.1	KDNSVLHSEVNTSGGAIEPEKEVQT	S	669
2215	LOC Os04g57140.1	LIAYWKEQAGKRSEDDDLEEIQEER	S	1171			2216	LOC Os04g57300.1	SPLLLTRNSINRSCSTPCLCPVSKD	S	100
2218	LOC Os04g57300.1	SGSNGSVHALNRSADEQLPSSVYFV	S	547			2219	LOC Os04g57600.1	RRRRINESSLAISPERNGEQRRRDE	S	410
2221	LOC Os04g57600.1	RHRLRRRRINESSLAISPERNGEQR	S	406			2222	LOC Os04g57600.1	EAGQHEEQTQRRSSELALGSRNADG	S	508
2224	LOC Os04g57600.1	LAEAEKEGDNIISSDEYKSGSGDEE	S	611			2225	LOC Os04g57600.1	KERDRRRLRDRLSPVRRTEFQGTRH	S	482
2227	LOC Os04g58110.1	DWTISSEGPARHSLIYRGLVPLLAE	S	447			2228	LOC Os04g58140.1	AIDTLPPPSRDVSKPLRLPICDVFS	S	480
2230	LOC Os04g58620.1	EGIEQLESSREMSDDESTDKLLVEP	S	391			2231	LOC Os04g58620.1	DLAVEGIEQLESSREMSDDESTDKL	S	387
2233	LOC Os04g58720.1	PEPQQPKIVRMYSAASQQPMDYALK	S	229			2234	LOC Os04g59040.1	PEYRHYNDISELSPHRLQEIKRFFE	S	159
2236	LOC Os04g59320.1	AQNPENAFCQSQSWRCPEGQSPMFQ	S	306			2237	LOC Os04g59330.1	AKTDSEVTSLAPSSPPRSPTSRGGR	S	15
2239	LOC Os04g59400.1	FSDAEEGMEGPISPRDSCLVEPVAE	S	199			2240	LOC Os04g59400.1	PSQSDEEPLHRQSQFSDAEEGMEGP	S	185

2242	LOC Os04g59460.1	DSVLPSSKKKSLSFSSSFSKRTQPS	S	1262			2243	LOC Os04g59460.1	LPSSKKKSLSFSSSFSKRTQPSHLF	S	1265
2245	LOC Os04g59460.1	SKKKSLSFSSSFSKRTQPSHLFSGN	S	1268			2246	LOC Os04g59460.1	IEDANKAIDVNNSSPGIEPFNAISK	S	413
2248	LOC Os04g59570.1	VAARPLRSRRALSVFTCAAPPRQRP	S	25			2249	LOC Os04g59624.1	NELDEKLTVIESSPEPLERKGPTEV	S	574
2251	LOC Os05g01060.1	PQQAYSHVQSPMSSPVRARRDWDLL	S	388			2252	LOC Os05g01060.1	QQAYSHVQSPMSSPVRARRDWDLLG	S	389
2254	LOC Os05g01350.1	RPLPPLPRVGPPSGEFASRSSASDP	S	326			2255	LOC Os05g01540.1	PRRRLTPPRRMWSPPRRPQSLRHRS	S	335
2257	LOC Os05g01590.1	QSNKSRERVWNESDVDEEEPCYDNL	S	152			2258	LOC Os05g01610.1	CFLKLKNALDSDSFNKRKDIVSHLA	S	495
2260	LOC Os05g01910.1	VGLPGIDLDRQGSFSSIFQESSYQL	S	127			2261	LOC Os05g01990.1	SSAHGKNHTSLLSDDEEEILEKHNV	S	267
2263	LOC Os05g02240.1	HHRKLHGPSAPSSPSAAATAGAKEL	S	457			2264	LOC Os05g02240.1	LHHRKLHGPSAPSSPSAAATAGAKE	S	456
2266	LOC Os05g02400.1	SSACPNKVAAVNSDMEKSYGGSSTI	S	491			2267	LOC Os05g02500.1	RKFISRSLARVYSDSALIRDLDPRV	S	475
2269	LOC Os05g02500.1	ERRGGFSSLKLPSFQKDLVLPPRVP	S	596			2270	LOC Os05g02650.1	GSDGKDLGDNDTSSIGASSDKGPKK	S	434
2272	LOC Os05g03060.1	AAEASRLDPAGDSPEMRRVVDPDGA	S	51			2273	LOC Os05g03060.1	SHTSESFSTRSSSLSPSLRNSNDHA	S	428
2275	LOC Os05g03060.1	ESFSTRSSSLSPSLRNSNDHAPTRA	S	432			2276	LOC Os05g03060.1	DGATEEAPEQSESPKRRKAKAGVNL	S	73
2278	LOC Os05g03120.1	AHNKDDVDGSFGSPDHGEAVEKVVV	S	304			2279	LOC Os05g03120.1	AGITPTRTTPERSPKVTERRSPRSP	S	50
2281	LOC Os05g03120.1	EYTNSMIGGKLASSPFSDEPEEESP	S	571			2282	LOC Os05g03120.1	YTNSMIGGKLASSPFSDEPEEESPK	S	572
2284	LOC Os05g03120.1	GGGIGRNVERTGSLEPAEYTNSMIG	S	554			2285	LOC Os05g03190.1	DKESNLDRASAKSASLNLGEGEITK	S	286
2287	LOC Os05g03320.1	VKSMLSGENSEGSDGSKSSDTVEWD	S	24			2288	LOC Os05g03320.1	TDAVKSMLSGENSEGSDGSKSSDTV	S	21
2290	LOC Os05g03440.1	KDAAAAAEIAAPSPRKSQKASAAAE	S	268			2291	LOC Os05g03630.1	RLAMKHHPDKNRSPHADDSLFKQVS	S	38
2293	LOC Os05g03630.1	RAPGAGFPGFGGSPRAGETSATKAP	S	132			2294	LOC Os05g03740.1	RRKAAAAARRTGSTGGVEGEVEAML	S	235
2296	LOC Os05g03760.1	RLLLKSPAVSPSSSPKKSASPPSPP	S	173			2297	LOC Os05g03760.1	LLLKSPAVSPSSSPKKSASPPSPPP	S	174
2299	LOC Os05g03760.1	QGDELHKLRKSASFAFRGQSAMPVA	S	551			2300	LOC Os05g03760.1	SSRASAFAKRSQSFIDRGGRAPAAR	S	498
2302	LOC Os05g03780.1	PQIAEVRMDISSSTSVAAGNKVCRG	S	21			2303	LOC Os05g03820.1	KEGKPNGYLSLRSHIWTDTDNNRSG	S	271
2305	LOC Os05g05230.1	RPDGSELPKGSESDDCSQISGLKVE	S	377			2306	LOC Os05g05240.1	PKLIHPLPPPVNSPESSPERAMDAT	S	849
2308	LOC Os05g05240.1	AVGDLGCLVRQLSLDQFENESRRMH	S	501			2309	LOC Os05g05300.1	GTTEIKEPHDQQSEDSDQEELDRFI	S	331
2311	LOC Os05g05300.1	DPVAMASIQRVASTFFRAIDEQQGT	S	294			2312	LOC Os05g05300.1	EKPRNSATASGRSVFRESEDDELDT	S	488
2314	LOC Os05g05480.1	RGLAVDFKESQVSDFEDLEEDKFLN	S	93			2315	LOC Os05g05590.1	FGGRGFVPFVPGSPVERSIHGSQLG	S	524
2317	LOC Os05g05720.1	SNTRSSVTATGRSPSPNSSSLAPLN	S	94			2318	LOC Os05g05720.1	TRSSVTATGRSPSPNSSSLAPLNEN	S	96
2320	LOC Os05g05950.1	IMKELGEGSASVSPVSGLSSSREYT	S	471			2321	LOC Os05g05950.1	AAKQIMKELGEGSASVSPVSGLSSS	S	467
2323	LOC Os05g05950.1	TNSMDGQIVLDDSEEDGDDDDNEDD	S	495			2324	LOC Os05g06280.1	ANLQEEEKVTKVSPPRRKAYRDDKP	S	639
2326	LOC Os05g06760.1	VQRCRARLNRLASASSGDDAEWEEL	S	122			2327	LOC Os05g06980.1	SRAQITEDQDKSSGNISVENIEGLK	S	247
2329	LOC Os05g07000.1	SNERYDGTRRYASPSYGRDRSPGGN	S	238			2330	LOC Os05g07000.1	RSHNLTSDGMNPSPRERDDQNGSHR	S	269
2332	LOC Os05g07130.1	KKLQVSGIVESKSVDTLTTLQKQDG	S	298			2333	LOC Os05g07130.1	HNNCNKDNVALPSDSFKKLQVSGIV	S	282
2335	LOC Os05g07220.1	KDNWADSDVENGSISRSSDEKESRE	S	235			2336	LOC Os05g07220.1	QSGGAVKDNWADSDVENGSISRSSD	S	229
2338	LOC Os05g07680.1	KNMRCHCNFGANSELSEHEEKQQVS	S	214			2339	LOC Os05g07680.1	SELSEHEEKQQVSDVETSSLQKDLD	S	226
2341	LOC Os05g08370.1	IQRQGEDVDLSSSSRHEQHRIPRLT	S	141			2342	LOC Os05g08370.1	QRQGEDVDLSSSSRHEQHRIPRLTS	S	142
2344	LOC Os05g08790.1	AKGEILRLRRQISASGYLRTELAET	S	687			2345	LOC Os05g08840.1	KMDTDDALGDPASGTDENMQESKCS	S	526
2347	LOC Os05g09540.1	PAASVPWRWLAFSLCRFLDLPADGS	S	86			2348	LOC Os05g09630.1	NIMLSRPFTRSFSFEPGRLVSLIDN	S	581
2350	LOC Os05g10730.1	AFQYQVPLIQQNSEAEISTGNLKSV	S	888			2351	LOC Os05g10770.1	RPPIDYSRFEHISDEDSDVEIVEKD	S	98
2353	LOC Os05g11370.1	KVPQGEDFDKLESDSDDCKEVAALS	S	197			2354	LOC Os05g11370.1	PQGEDFDKLESDSDDCKEVAALSAA	S	199
2356	LOC Os05g11770.1	EDIVESVMMLGGSKSDTELDAEPER	S	757			2357	LOC Os05g11770.1	IVESVMMLGGSKSDTELDAEPERTA	S	759
2359	LOC Os05g11770.1	IVCLSNHDEKNESPPADSYNHLDLR	S	648			2360	LOC Os05g11770.1	EPSQDFPKLVCCSEQLDKTHGSENA	S	551
2362	LOC Os05g12100.1	SVQHCRHAQHTTSTPRRPACPEGDE	S	133			2363	LOC Os05g12680.1	LEDLNEGVRPGHSRYFGSYSSSDYD	S	592
2365	LOC Os05g15190.1	DCSDLSPPKSSSSGGSSHRSRECRP	S	264			2366	LOC Os05g18770.1	SSVDKITDKKDLSHTIRGESVLGKS	S	67
2368	LOC Os05g18774.1	IFHKEVPFDEDCSDDEPDGGGGRRS	S	84			2369	LOC Os05g22670.1	PPQLTKDFLMMSSGKRKRDEYPGQL	S	204
2371	LOC Os05g22920.1	RRELDGVGLAEESPPPGSSLGKILS	S	53			2372	LOC Os05g22940.1	TKLSELRARIARSLSELEMFTEESK	S	1200
2374	LOC Os05g22990.1	KVSEPTGSPAKDSPVGKEKAIEKSQ	S	722			2375	LOC Os05g22990.1	TVNLKATDNPSSSNDGLTQVRTLLP	S	790
2377	LOC Os05g23320.1	PTPLRQKYSRMSSLFNNKRTMAARI	S	550			2378	LOC Os05g23610.1	QDDDPEAEKSRLSFKEHRRAHYDEF	S	106
2380	LOC Os05g23610.1	SSKTDPKDDGWASSDDDADAMEQDD	S	84			2381	LOC Os05g23610.1	SKTDPKDDGWASSDDDADAMEQDDD	S	85
2383	LOC Os05g23610.1	PYHPMVDDDGSLSPTRPFDKCLDET	S	44			2384	LOC Os05g23840.1	PPRNPIIPRDPPSPEMEATAEALTR	S	49
2386	LOC Os05g26890.1	SQVESDSSKYVISPDNQEIGEKLSD	S	73			2387	LOC Os05g27820.1	MSSLMEATALLRSSSFKEDSYVASA	S	44
2389	LOC Os05g27820.1	SLMEATALLRSSSFKEDSYVASALP	S	46			2390	LOC Os05g27820.1	TALLRSSSFKEDSYVASALPASDLR	S	51
2392	LOC Os05g28190.1	SEEQKDADNKGQSSSPTPLFSFKNL	S	246			2393	LOC Os05g28190.1	EQKDADNKGQSSSPTPLFSFKNLSS	S	248
2395	LOC Os05g28190.1	LKGSDEKAKSSDSIEPPSQPVETTD	S	152			2396	LOC Os05g28190.1	ETTDEAKDLGGGSVVAGEAKEDNSK	S	173
2398	LOC Os05g28190.1	AETEKDGSDLKGSDEKAKSSDSIEP	S	143			2399	LOC Os05g28190.1	GSDLKGSDEKAKSSDSIEPPSQPVE	S	149
2401	LOC Os05g28280.1	TSHSLQELQPTKSIEDNAEIKAWLA	S	344			2402	LOC Os05g28860.1	IQKLFKGTTGGLSGLSKKILGAGLN	S	500
2404	LOC Os05g29010.1	ELHDKWQGNRRSSISKEDVNATRWE	S	101			2405	LOC Os05g30240.1	QAPGNHALQGQNSFNARRGQVGFGN	S	852
2407	LOC Os05g30340.1	PCKRLRVSVKRESGAHSEDESVAPT	S	388			2408	LOC Os05g30420.1	ARAPVDEFEPVDSGSDLESDDLKSA	S	110
2410	LOC Os05g30420.1	EFEPVDSGSDLESDDLKSALGKQRR	S	116			2411	LOC Os05g30510.1	GGEQQSSAAAASSGDGVAAAANVDR	S	48
2413	LOC Os05g31920.1	ETKSNAQLSRLSSLESFKHMDSRTS	S	763			2414	LOC Os05g31920.1	ESRSDMLSSGTTSPRTYTRRSSPLS	S	150
2416	LOC Os05g32070.1	RRRERQQLAGAASSPPTSSAFPAAT	S	161			2417	LOC Os05g32070.1	SRTSTSNATTPRSFDTSSSHQVASF	S	204
2419	LOC Os05g32580.1	EEEEEEERDLFGSDNEDYVKTPARS	S	118			2420	LOC Os05g32600.1	KLADFGLARIFGSPERNFTHQVFAR	S	168
2422	LOC Os05g32760.1	IQEPEDEKYVPASPPRPPLSPPTPV	S	269			2423	LOC Os05g32760.1	KYVPASPPRPPLSPPTPVAAPAPQE	S	276
2425	LOC Os05g33100.1	PPEVVKQFEQVLSSKSGIGHFVKIF	S	190			2426	LOC Os05g33100.1	VVKQFEQVLSSKSGIGHFVKIFPGV	S	193
2428	LOC Os05g33310.1	RPSTPVDLSPLPSPTKAMTLWQAPP	S	247			2429	LOC Os05g33440.1	MDLLRPLQRSNISFFERFVRKYEED	S	362
2431	LOC Os05g34500.1	HQQQQQPHRRNLSLEELAGEVGLSP	S	71			2432	LOC Os05g34500.1	LSLEELAGEVGLSPGRRLPRVALPK	S	82
2434	LOC Os05g34780.1	AENITPEDESLDSSEDNTDSIMERK	S	339			2435	LOC Os05g34780.1	ENITPEDESLDSSEDNTDSIMERKS	S	340
2437	LOC Os05g34790.1	HGKSGDMTVDLDSDGSEDHGTRQHE	S	156			2438	LOC Os05g34790.1	SGDMTVDLDSDGSEDHGTRQHEAKP	S	159
2440	LOC Os05g36090.1	GRSASDVIRRSASGHEAAPPFQTET	S	416			2441	LOC Os05g36090.1	EVRVRHSSWDRRSGNWDMSLDVIGR	S	393
2443	LOC Os05g37434.1	RFLDGELDPQVGSPQNKTSPTKHSL	S	125			2444	LOC Os05g37434.1	LDPQVGSPQNKTSPTKHSLAEPMNM	S	131
2446	LOC Os05g37500.1	VSKVQEPQKDIASPTSSQSVVLSSG	S	738			2447	LOC Os05g37720.1	GREEEDELGAFLSGMRLLRYLYYRT	S	141
2449	LOC Os05g37930.1	EEKAAVAARSDSSDEEDCLAGLSRR	S	39			2450	LOC Os05g38150.1	GRQRLRYRKLVNSSFADLQKPQMEL	S	78
2452	LOC Os05g38710.1	DVSNEDTELEIASPSFGKYDTFKSC	S	509			2453	LOC Os05g38710.1	ASCEPEPPARSKSDGDLYIGAKGRP	S	119
2455	LOC Os05g38810.1	IRRAASQLTGSSSIVKSQNEQVPHK	S	563			2456	LOC Os05g38810.1	SFKIGECIRRAASQLTGSSSIVKSQ	S	556
2458	LOC Os05g38810.1	NIAETENGDFDVSSDDAINELSVEK	S	588			2459	LOC Os05g38810.1	IAETENGDFDVSSDDAINELSVEKR	S	589
2461	LOC Os05g38810.1	DGLQSKKKRSLDSFEDPETKAAAPS	S	528			2462	LOC Os05g38810.1	KPQDGLELTEKKSTSNLSTDNAYDH	S	490
2464	LOC Os05g38810.1	LELTEKKSTSNLSTDNAYDHPAERQ	S	495			2465	LOC Os05g38810.1	SFEDPETKAAAPSFGSSFKIGECIR	S	540

2467	LOC Os05g38810.1	PETKAAAPSFGSSFKIGECIRRAAS	S	544			2468	LOC Os05g38830.1	TSGSGVMTERVPSFSRKKFKVDRDN	S	125
2470	LOC Os05g38950.1	TSQQKALLSPLPSPRTSMIDASPGR	S	703			2471	LOC Os05g38950.1	PLPSPRTSMIDASPGRANHDSSQEF	S	712
2473	LOC Os05g38950.1	QVETPKLDVLEESMGSPKEDNKSRV	S	786			2474	LOC Os05g38950.1	VIHPVKVQEGKLSEDSDDEFYDVDK	S	143
2476	LOC Os05g39390.1	EREKVQAVFKRLSSDPVGIRVHDVI	S	61			2477	LOC Os05g39390.1	REKVQAVFKRLSSDPVGIRVHDVII	S	62
2479	LOC Os05g39730.1	PATSRTSLEKRYSFIQSEERRFVES	S	309			2480	LOC Os05g39730.1	IFIMESPSAPRRSPGPSPLGGSPTM	S	521
2482	LOC Os05g39730.1	PRRSPGPSPLGGSPTMGDRTMKAPT	S	530			2483	LOC Os05g39840.1	LGQILSQKYDALSPLRIDHGGASRR	S	503
2485	LOC Os05g40420.1	AVGLPSDDDMGNSEVGHNALGAGRI	S	81			2486	LOC Os05g41100.1	KKKDILAGSDNESDGGGGEEDLSKI	S	22
2488	LOC Os05g41100.1	EKKYSPDDIQEVSDEEEHLIEQEDY	S	285			2489	LOC Os05g41100.1	RPYLETGSEKKYSPDDIQEVSDEEE	S	277
2491	LOC Os05g41480.1	QKKISQADEEAGSLLQERDGHSDRK	S	337			2492	LOC Os05g41480.1	QALKAKEAQKKISQADEEAGSLLQE	S	329
2494	LOC Os05g41510.1	ADEEEEEEDERESDDERRKKKRKKK	S	74			2495	LOC Os05g41670.1	NDDEDEEPHMGSSSHGGDRIKNRSL	S	222
2497	LOC Os05g41790.1	PDSNALSSQRFGSSSPGGDMKNKTR	S	109			2498	LOC Os05g41790.1	SNALSSQRFGSSSPGGDMKNKTRKR	S	111
2500	LOC Os05g42220.1	TENESGKIKKSQSLGDMLEMEKLYD	S	80			2501	LOC Os05g42230.1	LAGGAAAAIQPNSPRFFFSSLAAAS	S	38
2503	LOC Os05g42300.1	GENDEDQTDPEFSDDEKEAEYKRSL	S	379			2504	LOC Os05g42300.1	NMKELYAKGYDGSGENDEDQTDPEF	S	366
2506	LOC Os05g43280.1	RDEDRSLSGESLSEWRSCEQVESES	S	30			2507	LOC Os05g43280.1	STAGMRDEDRSLSGESLSEWRSCEQ	S	25
2509	LOC Os05g43380.1	KRFLYTGADLDHSEDEQDFIVERSP	S	231			2510	LOC Os05g43380.1	HSEDEQDFIVERSPRLQSPISKESS	S	242
2512	LOC Os05g43520.1	ETKSLAIEPKIHSETDEDEVDEWPD	S	375			2513	LOC Os05g43520.1	EEGRDGEEESAQSPRIAGIRSDLAE	S	111
2515	LOC Os05g43530.1	YEWVVTKKSGRSSEGDLVGLVEKHS	S	593			2516	LOC Os05g43670.1	EEEEDDDEDAPASLPPPPRRARAPP	S	188
2518	LOC Os05g43860.1	SRDMVPPLPPLPSMKSPSESWLSRA	S	510			2519	LOC Os05g43950.1	VPSPASSLQNSQSQGNNREASDPPS	S	93
2521	LOC Os05g44310.1	KSAVSYALNRQISMDDLTKPLDEES	S	458			2522	LOC Os05g44320.1	DAGALDLIDDEDSDVEEGIDQQTRG	S	681
2524	LOC Os05g44380.1	AAASELLLPVASSPSPRSAAAAASR	S	87			2525	LOC Os05g44380.1	ASELLLPVASSPSPRSAAAAASRSP	S	89
2527	LOC Os05g44922.1	DSYPNLRALRNASSVSLADAAYVKI	S	71			2528	LOC Os05g44922.1	PNLRALRNASSVSLADAAYVKISEG	S	74
2530	LOC Os05g45060.1	VPRVIFVADDSDSPGSSSRGGAGGG	S	86			2531	LOC Os05g45280.1	MGDEKSPLSQMGSRDRDRELLIPVS	S	13
2533	LOC Os05g45930.1	NEAVQEVGRGENSSLQGEVVVRDVS	S	76			2534	LOC Os05g45930.1	EAVQEVGRGENSSLQGEVVVRDVSQ	S	77
2536	LOC Os05g45930.1	NDMEQRSKEEPLSTESLKSSKRRSS	S	457			2537	LOC Os05g45930.1	EQRSKEEPLSTESLKSSKRRSSFST	S	460
2539	LOC Os05g45930.1	GKDAGFESSPVISEPVLVTPHNNEA	S	54			2540	LOC Os05g45930.1	SSLQGEVVVRDVSQDLEKQNTVVSD	S	88
2542	LOC Os05g46340.1	KFRQQLQLQRLNSLLNYKEMLNRGS	S	361			2543	LOC Os05g46490.1	DVDFYPHFDANHSAKHQSKENAIVA	S	218
2545	LOC Os05g46560.1	AFIEADEAKELLSPLIKPGNAYKRI	S	146			2546	LOC Os05g46620.1	FQGAKSLYHRENSLQVETSPRQGAA	S	323
2548	LOC Os05g47470.1	EDEDEAAAPATPSKEAEVGYHGLMA	S	178			2549	LOC Os05g48020.1	IGEGLETLKNMASDMNEELDRQVPL	S	198
2551	LOC Os05g48260.1	ERDVEAAVPVPMSPAGRLFRETNFN	S	93			2552	LOC Os05g48260.1	TPSPSSLRKRVLSIDTSSRDSRGSP	S	39
2554	LOC Os05g48820.1	EKDVDDAERDEDSECDDADDDPDGD	S	910			2555	LOC Os05g49050.1	DGLAPHSHEPIYSPGDFSKRAPPLI	S	62
2557	LOC Os05g49180.1	VRGGADRGGRNSSFHSSSIDNVASR	S	112			2558	LOC Os05g49180.1	DRGGRNSSFHSSSIDNVASRSISGP	S	117
2560	LOC Os05g49230.1	DSENEDIQTREYSDEEMDSDEEQQR	S	441			2561	LOC Os05g49230.1	IQTREYSDEEMDSDEEQQRYDAQLE	S	447
2563	LOC Os05g50280.1	AGGGGGMGSRLGSSAYSLRDSSVSA	S	50			2564	LOC Os05g50280.1	GGGGGMGSRLGSSAYSLRDSSVSAV	S	51
2566	LOC Os05g50480.1	GDEEVAAVSDNHSIDQSDVRIHEYE	S	457			2567	LOC Os05g50480.1	VAAVSDNHSIDQSDVRIHEYENKDT	S	461
2569	LOC Os05g50480.1	PAADEHVASSTKSDTENVDVAPVQQ	S	488			2570	LOC Os05g50480.1	TQPGLGHEHRNPSDGNLSSAAAAAQ	S	841
2572	LOC Os05g50530.1	RNLPKDIPLPTESVSRGKRRAVSAI	S	179			2573	LOC Os05g50530.1	TESVSRGKRRAVSAIESSFNLDARA	S	189
2575	LOC Os05g50530.1	SRGKRRAVSAIESSFNLDARAKLDA	S	193			2576	LOC Os05g51500.1	APAPVEPDAAAKSDAEDDDLDFDFS	S	47
2578	LOC Os05g51500.1	EEEEDEDEWDAKSWDDIDVNLPKTS	S	525			2579	LOC Os05g51630.1	RRNTPAQSKYEGSDTLSLPETVHER	S	754
2581	LOC Os05g51830.1	KRKVAEIVLKTPSSDKKAKIATPSG	S	218			2582	LOC Os05g51830.1	RKVAEIVLKTPSSDKKAKIATPSGQ	S	219
2584	LOC Os05g51850.1	ETRPADAGGVVVSEEKTSIDPVEAG	S	504			2585	LOC Os05g51850.1	AVQNGGEVRCLLSDGASSMGNRGPG	S	376
2587	LOC Os05g51850.1	GEVRCLLSDGASSMGNRGPGSPRKE	S	381			2588	LOC Os05g51850.1	AAAASPVAPPPASPLKRGVGRPRKN	S	250
2590	LOC Os06g01400.1	KFFYKMDTQKKLSEFRDQLSSILFH	S	721			2591	LOC Os06g01650.1	PSSTIALRESSASPPPSVQSIPVQS	S	1070
2593	LOC Os06g01680.1	LLTKSTDNKGSSSFGLHIGSSDRSH	S	616			2594	LOC Os06g01680.1	LSDGAVQNLLPISPTHKLDVASTHP	S	511
2596	LOC Os06g01680.1	NAFVEKKLQPQSSNISLEPKVIDAT	S	305			2597	LOC Os06g01700.1	DDSDVEPRSDDESDEDDDDDDDTEA	S	123
2599	LOC Os06g01700.1	DKIVPREIDADDSDVEPRSDDESDE	S	113			2600	LOC Os06g02028.1	LTTYDFEADCFSSPYDDLNKRKLAY	S	110
2602	LOC Os06g02160.1	KAYGISRMNLVPSLMEIILPSLEKN	S	299			2603	LOC Os06g02160.1	NLVPSLMEIILPSLEKNLSWGHNPL	S	307
2605	LOC Os06g02170.1	DSPHSQASPLSPSALRRHYKSLSES	S	92			2606	LOC Os06g02170.1	DSSHRATLLLPDSPHSQASPLSPSA	S	81
2608	LOC Os06g02180.1	LSIMRSQKAMTRSQTGDWDHNRWLF	S	240			2609	LOC Os06g02370.1	EKRLGMEKGEGMSAVADSGEGRGRV	S	573
2611	LOC Os06g02380.1	QKVGAEIVRRALSYPLKLIAKNAGV	S	505			2612	LOC Os06g03676.1	ASDSQLLEKFDLSDSEDATSDESNE	S	504
2614	LOC Os06g03910.1	LYEMAKREGLLPSSPTTSRRRGSSS	S	309			2615	LOC Os06g03910.1	YEMAKREGLLPSSPTTSRRRGSSSS	S	310
2617	LOC Os06g04560.1	SAAGGGAYRRTSSGPLPSAGGGGGR	S	32			2618	LOC Os06g04560.1	SGKIFPGFDSLMSHSRNSQPREQSN	S	608
2620	LOC Os06g04940.1	ADKKILSLARRHSFENAPEHLKNTS	S	113			2621	LOC Os06g05000.1	ADKKILSLARKHSFEGAPEHLKNTS	S	89
2623	LOC Os06g05190.1	HDNDKKHQSKPRSDDAQYDSDDTIE	S	315			2624	LOC Os06g05190.1	QSKPRSDDAQYDSDDTIEMTEEEID	S	322
2626	LOC Os06g05310.1	RFVTPLPMGVDMSQQRRRTFLLNAG	S	229			2627	LOC Os06g05530.1	GGLAAGTTARGPSPLEDPALVRRSS	S	87
2629	LOC Os06g05660.1	LNASLPAAAAALSAEDRAGLVNALK	S	25			2630	LOC Os06g05740.1	ILKSMEGIPGAPSMKMYSRDDLMKN	S	213
2632	LOC Os06g06014.1	QENNVDAVEKTISSEDQVAEKEDVE	S	271			2633	LOC Os06g06014.1	REESGPEWPLAPSPPASPTTWHDNK	S	81
2635	LOC Os06g06770.1	RNLQVALTVEPGSPVKRPLYQGRPP	S	110			2636	LOC Os06g06830.1	DRSAPHATEGASSSRVSPQHGRGNQ	S	124
2638	LOC Os06g07350.1	PAPPGAERTLYPSMDPQRMGALVKS	S	370			2639	LOC Os06g07820.1	QSPDDRVLIKTRSNQSIDQWVFAKS	S	268
2641	LOC Os06g07840.1	KEKQQQMKVTQKSELGKASSVHAID	S	36			2642	LOC Os06g08023.1	DEFLIKCDGVKNSLLPSMAKLLKLN	S	176
2644	LOC Os06g08280.1	GSRGVGMGVGDPSSPSARAGAAAEE	S	15			2645	LOC Os06g08280.1	SRGVGMGVGDPSSPSARAGAAAEEE	S	16
2647	LOC Os06g08280.1	GQMRLISVPRVASFGELMRKVEAVD	S	70			2648	LOC Os06g08480.1	CEETKPDASEETSSSESGSKKKPVK	S	19
2650	LOC Os06g08480.1	ETKPDASEETSSSESGSKKKPVKRY	S	21			2651	LOC Os06g08480.1	KPDASEETSSSESGSKKKPVKRYLI	S	23
2653	LOC Os06g08550.1	VVWSYTYNRKSASSDEIVEARKNSQ	S	191			2654	LOC Os06g08550.1	VWSYTYNRKSASSDEIVEARKNSQP	S	192
2656	LOC Os06g08740.1	PSPLGLRLRKSPSLLDLIQMRLSQA	S	89			2657	LOC Os06g08740.1	TSDERSLMARVDSLCCLIQKDTPPV	S	453
2659	LOC Os06g08740.1	DEHELSGFSGSLSPCAGSSMSAKIE	S	320			2660	LOC Os06g08790.1	DDVYVKRRDGAESDAEDPEAEECRV	S	153
2662	LOC Os06g08840.1	PRRRSVSPARARSYSRSPQYNRGRD	S	159			2663	LOC Os06g08840.1	RRSVSPARARSYSRSPQYNRGRDES	S	161
2665	LOC Os06g08840.1	PRYRRSPSYGRRSYSPAGRSPRRRS	S	139			2666	LOC Os06g08840.1	SYGRRSYSPAGRSPRRRSVSPARAR	S	146
2668	LOC Os06g08850.1	SFFSRRSFEDDGSPEHSLVLNAVRK	S	648			2669	LOC Os06g09570.1	RTFTTNRMGRQLSSMGFDPRAALDR	S	551
2671	LOC Os06g09570.1	GRKRERSLSRAASDGDDMDIDGQQS	S	582			2672	LOC Os06g09930.1	FKRSLPTLTRFKSQQENELTSLIVD	S	305
2674	LOC Os06g10710.1	RRPAARAPNPSLSPRGGGGAPSRKS	S	58			2675	LOC Os06g11380.1	TDTVLLTKEEGGSPIRNPPTAAEDA	S	330
2677	LOC Os06g11610.1	GAPLRRYDVVDESGTDSGDEYDATD	S	77			2678	LOC Os06g11970.1	LMFSPSGRLSHFSGRRRIEDVLTRY	S	58
2680	LOC Os06g12030.1	VEDEKNEGSESISSGNIGSDAEPKS	S	182			2681	LOC Os06g12030.1	EKNVAVTEGSTNSEEKDQDEDLLRR	S	20
2683	LOC Os06g12160.1	VFHNLARLSPLTSSLKRLVGGLKAR	S	512			2684	LOC Os06g12260.1	ASLGDAGGNADASPAAPALRPAAVA	S	78
2686	LOC Os06g12400.1	KQIDASDLPSDDSADNDYDPTLAQG	S	349			2687	LOC Os06g12400.1	KSAEKLIPGLENSDEARRKAVQREL	S	773
2689	LOC Os06g12580.1	PQQEESYGSEYGSGYGRKPQAESYG	S	225			2690	LOC Os06g12610.1	PRKADGVERDDFSFGNRGVAERDAE	S	386

2692	LOC Os06g13810.1	QASNAPAPTRLASVYSEVQTSRLKH	S	28			2693	LOC Os06g13820.1	KGPNTDGGSMRQSNSDGALDTMARR	S	720
2695	LOC Os06g13820.1	NKIRAVIQSKGGSFKGPNTDGGSMR	S	706			2696	LOC Os06g13820.1	QSSLLSKLTRQLSVHDNRAASYAND	S	826
2698	LOC Os06g14080.1	RGPARRRGTVRASLDADEFIALMHG	S	26			2699	LOC Os06g14406.1	MSQKSGRSSGMESPASLQIEDTRSM	S	171
2701	LOC Os06g14412.1	EVEEKLIDDISGSPSSHLPVALKST	S	1987			2702	LOC Os06g14412.1	QAKKEPASSVGDSPPAKRRRGRPRR	S	140
2704	LOC Os06g14412.1	TEEEFEKLCQVDSPDSPQPGGISRD	S	95			2705	LOC Os06g14412.1	EFEKLCQVDSPDSPQPGGISRDADV	S	98
2707	LOC Os06g14412.1	TRSDIETPAMDASESKSPESDVHEL	S	1366			2708	LOC Os06g14412.1	IETPAMDASESKSPESDVHELSERV	S	1370
2710	LOC Os06g14412.1	RRGRPRRSDASLSPVTAPPNTGKQE	S	158			2711	LOC Os06g14412.1	IEKNDDSPIERSSPSADKIAQVAYG	S	933
2713	LOC Os06g14412.1	GDNLPTIEKNDDSPIERSSPSADKI	S	927			2714	LOC Os06g14412.1	TIEKNDDSPIERSSPSADKIAQVAY	S	932
2716	LOC Os06g14490.1	TPPAQQKMERLQSFEEEHKNALERA	S	367			2717	LOC Os06g15420.1	VHDAAYEDTLQKSPASANPVLDNGF	S	561
2719	LOC Os06g16280.1	AMAKVFDEDDDGSYDDDLAKADSLN	S	1016			2720	LOC Os06g16390.1	YYDTNCGEALICSDSEDEAVEDEEE	S	178
2722	LOC Os06g16430.1	HKASLEEEDDILSDEDDFYDRTKKK	S	415			2723	LOC Os06g16980.1	RHPKCKYIARTLSDDDDETEASDET	S	1008
2725	LOC Os06g17290.1	SNHSGSQVGNWRSANEQLPTSASFV	S	641			2726	LOC Os06g17840.1	EINLDNQENLGESEKSKPKKKRMDA	S	1197
2728	LOC Os06g17840.1	PKEEEPDDHNLNSKEESDNETLSVW	S	1541			2729	LOC Os06g17840.1	GAKKPHAEILSSSPKKSDEAGSSKR	S	1306
2731	LOC Os06g17840.1	KGAKKPHAEILSSSPKKSDEAGSSK	S	1305			2732	LOC Os06g17840.1	KSRPHSTKGNKYSDELLLQSVDPDE	S	1242
2734	LOC Os06g17840.1	LLQSVDPDETINSFENKVEGAKKRD	S	1258			2735	LOC Os06g17840.1	ENKVEGAKKRDDSVDTELVTSPASV	S	1272
2737	LOC Os06g18130.1	IQNKDARSGKEASSSGGGDCTGVNF	S	74			2738	LOC Os06g19390.1	MDLPWSEMFRSASLRLPKQEEPTTT	S	32
2740	LOC Os06g19660.1	ADGSVSTLLKHPSGAGDENTVSEAP	S	838			2741	LOC Os06g19660.1	KRMDSDGASGQGSFGRKDSFGKEEP	S	698
2743	LOC Os06g19660.1	GASGQGSFGRKDSFGKEEPRGSQGD	S	704			2744	LOC Os06g19980.1	AIYKRARILLYRSDERKWTPEEYEK	S	420
2746	LOC Os06g20090.1	SGGHSSGKSPYSSRIRDADPTDQSA	S	755			2747	LOC Os06g20370.1	QKLQLIWDEVGESDEDRDKVLYQLD	S	24
2749	LOC Os06g20390.1	HVDQDFSRRDSFSAVQPGNNALRHS	S	208			2750	LOC Os06g20390.1	TNSHLTDMGRSQSFGSGQHGNEASS	S	182
2752	LOC Os06g21580.1	LTNGRSHEDKGSSGEEVVPARKRRG	S	23			2753	LOC Os06g22070.1	RVIDSEIECIVQSEEGAASAKQIDL	S	96
2755	LOC Os06g22100.1	LHMLRAQQHQPLSPAGDVKAERSML	S	32			2756	LOC Os06g22650.1	EKPKSVQARGDKSPSIKPEGAKQPD	S	288
2758	LOC Os06g22700.1	GWGSDPKGYPARSPPDHAGRYADPV	S	171			2759	LOC Os06g22700.1	GGGRGVARFRDGSPPYGRGGRSYGR	S	44
2761	LOC Os06g22820.1	STEEWANFRGGDSDSDFDEFGSMDV	S	455			2762	LOC Os06g22820.1	EEWANFRGGDSDSDFDEFGSMDVTK	S	457
2764	LOC Os06g23530.1	FLRGQGRSTIDVSPVRISTNPDGSL	S	327			2765	LOC Os06g24540.1	ASSGSDRVGRRHSFSAETEARRSSF	S	552
2767	LOC Os06g27970.1	LDEEDAFGRDFNSSPSPTAPPARSG	S	21			2768	LOC Os06g27970.1	EDAFGRDFNSSPSPTAPPARSGEKR	S	24
2770	LOC Os06g29310.1	SRKRNGEFSPTFSMRLRSRKVVEEV	S	195			2771	LOC Os06g29400.1	KEEIANASADVPSDNVEELGLKYEK	S	837
2773	LOC Os06g29400.1	PVDLYKAIFSDDSDDDMAEPLANQP	S	733			2774	LOC Os06g29430.1	DSNSNRSVKKSSSSNSQNTEGWISS	S	1124
2776	LOC Os06g29430.1	SNRSVKKSSSSNSQNTEGWISSQER	S	1127			2777	LOC Os06g29430.1	FFHLRRKKQKPESPPHAHPQGRNHP	S	1561
2779	LOC Os06g29430.1	RCRDCHRRKDAESDYYDEDEYSGRQ	S	243			2780	LOC Os06g30020.1	YSRYHSYDYGPASPSHGRSHPDRGW	S	477
2782	LOC Os06g30320.1	AREEVDAELRSVSFTLDPKERRRIQ	S	455			2783	LOC Os06g30750.1	RSRGFRLLGEDTSVHKALGGGKTAD	S	70
2785	LOC Os06g30750.1	ADTAAASPEKVSSPPPEPAPAVRSR	S	48			2786	LOC Os06g33520.1	SRKGGRGSDLDDSEDDENDSIDSRG	S	325
2788	LOC Os06g33520.1	SRRGGRGSDLDDSEDDEYSRGSSPR	S	288			2789	LOC Os06g33520.1	DLKTDSFHLGLSSGDEGEASTRGPA	S	89
2791	LOC Os06g33520.1	SDLKTDSFHLGLSSGDEGEASTRGP	S	88			2792	LOC Os06g33520.1	RGYDFDDEPGFQSPKGQRGQGGRYS	S	238
2794	LOC Os06g33520.1	AAKSLIEDEAELSDWISDLKTDSFH	S	72			2795	LOC Os06g34420.1	FSEPPKVNLDLESSASKHRKKRNVN	S	484
2797	LOC Os06g34440.1	DCHGNGDDGFVSSPSLKRLRKGGLF	S	385			2798	LOC Os06g34440.1	HGNGDDGFVSSPSLKRLRKGGLFHG	S	387
2800	LOC Os06g34710.1	DEEDGEDDTDVKSKSMEELESGDAD	S	286			2801	LOC Os06g34710.1	EDGEDDTDVKSKSMEELESGDADAD	S	288
2803	LOC Os06g34710.1	TDVKSKSMEELESGDADADAKNSNL	S	294			2804	LOC Os06g34710.1	ELVSGDADVRGTSAEELESHVEDKV	S	335
2806	LOC Os06g36360.1	SEPGRSVKRASISPSISPVHQKTSS	S	554			2807	LOC Os06g36360.1	RSVKRASISPSISPVHQKTSSPPSG	S	558
2809	LOC Os06g36360.1	PGRSVKRASISPSISPVHQKTSSPP	S	556			2810	LOC Os06g36700.1	TGGRIVPRFQELSPEKLGKAGIVRE	S	345
2812	LOC Os06g36940.1	EGQREDEDWIGESEDDKDSLSRGSS	S	270			2813	LOC Os06g37440.1	APKEAPKPKVEASEEEEAPKPKPKN	S	246
2815	LOC Os06g38470.1	DQEDPDERHHADSDVEMDDVKPLDD	S	420			2816	LOC Os06g38960.1	PCRPKPSLDVSKSVCGLDLSSVKDN	S	60
2818	LOC Os06g39480.1	DPQTLSPQDKFESKLQSPKKDAGSI	S	212			2819	LOC Os06g39600.1	LTTPRPRVQRSISAEGLRELQSFLD	S	351
2821	LOC Os06g39912.1	TEEPQPELRHSKSVGSIGMMRRVAA	S	57			2822	LOC Os06g39912.1	RTKDIAPDVDPPSPRVSGCILCSIF	S	118
2824	LOC Os06g40460.1	VQESQKRKFRALSHGVEVENSSSKV	S	248			2825	LOC Os06g40510.1	EQALNQELIKQGSLHVNIPDQDPED	S	291
2827	LOC Os06g40650.1	PPPMRNAYSRSTSFDQHSGVYSRSS	S	351			2828	LOC Os06g40650.1	TSFDQHSGVYSRSSSFGPQTSGFQQ	S	362
2830	LOC Os06g40704.1	SRKDIGNASNSYSFKSEAGMVEFIG	S	153			2831	LOC Os06g40704.1	QAMDSRKDIGNASNSYSFKSEAGMV	S	149
2833	LOC Os06g40950.1	DDAEKEEGFDMDSESEEDTKSKPES	S	63			2834	LOC Os06g40950.1	AEKEEGFDMDSESEEDTKSKPESED	S	65
2836	LOC Os06g42030.1	KKAAANGNALALSGRGGGGGAAAGV	S	159			2837	LOC Os06g42200.1	GRASGSRDRGPGSSRPADAREKRKQ	S	342
2839	LOC Os06g42490.1	PFCGSPDINSRRSDSPDLEARRQPE	S	438			2840	LOC Os06g42490.1	CGSPDINSRRSDSPDLEARRQPEPP	S	440
2842	LOC Os06g42490.1	AVNELRLKRCMTSFGDSSKAADKKS	S	374			2843	LOC Os06g42790.1	GLAKVDKDYAGFSPILIKSAFTDKK	S	332
2845	LOC Os06g42810.1	LSDLGGGKDSAGSEDSEDDEYKPPQ	S	175			2846	LOC Os06g42810.1	LGGGKDSAGSEDSEDDEYKPPQELY	S	178
2848	LOC Os06g43560.1	DHDSNPGHARKDSAESIGSDLSSLR	S	301			2849	LOC Os06g43560.1	SNPGHARKDSAESIGSDLSSLRGSE	S	304
2851	LOC Os06g43610.1	TRKNGGGKEKPGSDYSEDDGETAEP	S	354			2852	LOC Os06g43790.1	CGAPEKSLDRNMSISASLVSDKRMM	S	1478
2854	LOC Os06g43840.1	KRINDRCFAGKPSEQDKMRNDFWNE	S	877			2855	LOC Os06g43840.1	THALPPRPKRVASRENISPKDPHSH	S	708
2857	LOC Os06g43840.1	SPNNQLTGEGSLSGNARHREDGLTR	S	472			2858	LOC Os06g43840.1	IKGDDMTSPDLVSVEDSLPTSNTKS	S	769
2860	LOC Os06g44030.1	DMSKIGQPPKTSSPQGENGLSQGER	S	376			2861	LOC Os06g44200.1	NEGTYLATEVDQSDSEIETPVEIMR	S	438
2863	LOC Os06g44970.1	DVGASIASFRVDSDVVSLNGREALQ	S	188			2864	LOC Os06g44970.1	SIASFRVDSDVVSLNGREALQADAE	S	192
2866	LOC Os06g45560.1	TKMSNIDVAIVGSLGGSYLRSYNYK	S	794			2867	LOC Os06g45560.1	NIDVAIVGSLGGSYLRSYNYKPATR	S	798
2869	LOC Os06g45910.1	DPPQLSELIPENSPVGKPQDEIAEE	S	180			2870	LOC Os06g45990.1	AVTLAAVVSKNASFREESNFLDDLK	S	46
2872	LOC Os06g46400.1	YGRSALRDDDSESDEEAAPRRSRKR	S	653			2873	LOC Os06g46450.1	NRAIKAIEALPDSDDEDVLTSRRAL	S	406
2875	LOC Os06g46890.1	AAKEDPYNAYEMSPSDQRQNHETTR	S	268			2876	LOC Os06g46890.1	KEDPYNAYEMSPSDQRQNHETTRKR	S	270
2878	LOC Os06g47220.1	SGGGGGAAAKSYSFNGPSARDDPEA	S	40			2879	LOC Os06g47320.1	FLVNGVAFKKTFSYAGFEQQPKKFL	S	224
2881	LOC Os06g48350.1	GSDEDHSNSPTRSHDGDNVAADEDD	S	221			2882	LOC Os06g48530.1	GGPPQPAATRKRSRSPPPPPPPPSL	S	51
2884	LOC Os06g48530.1	DSDKRRKHGRGSSKETDPLSGAPVA	S	313			2885	LOC Os06g48530.1	ERDGSRYGRGDRSPSLDGADDQMFD	S	599
2887	LOC Os06g48530.1	PRARRPPPPPPDSPEGRSPPLPPPP	S	25			2888	LOC Os06g48530.1	PPPPPPDSPEGRSPPLPPPPPGGPP	S	30
2890	LOC Os06g48950.1	NTAMQSEYLRSLSNPNMQNLGAADL	S	440			2891	LOC Os06g49430.1	RVPKRKMPLPVESHKKNTVEMEFFT	S	67
2893	LOC Os06g49760.1	AQELGGNMGRAGSPGFKWHLSNVQT	S	135			2894	LOC Os06g49800.1	QASDTEKIHGQASDTEISSELTTAA	S	431
2896	LOC Os06g50350.1	PADVPDEPIEGLSDDEGDAPAKNPP	S	72			2897	LOC Os06g50490.1	KDSIKYLEMEIVSPRTVTTTIPTST	S	285
2899	LOC Os06g50890.1	PPYGGGRARRERSRSLPYSPYRMPE	S	247			2900	LOC Os06g50890.1	YGGGRARRERSRSLPYSPYRMPERG	S	249
2902	LOC Os06g50890.1	GRDEYYGNSYRRSPPPMYPSYRDTR	S	188			2903	LOC Os06g50890.1	MAETPERRRYSGSPSPYRGNPKSRS	S	13
2905	LOC Os06g50890.1	PDPRSQARSRSRSREREPDAVNHGN	S	53			2906	LOC Os06g50890.1	RDYYDGRGGRGYSPHRSPPYGGGRA	S	230
2908	LOC Os06g51250.1	DQGGGNSFTRYDSMNSSSDHDRTDA	S	1127			2909	LOC Os06g51250.1	GGNSFTRYDSMNSSSDHDRTDAFAR	S	1130
2911	LOC Os06g51250.1	NSFTRYDSMNSSSDHDRTDAFARFD	S	1132			2912	LOC Os06g51250.1	DHDRTDAFARFDSMKSTDYNSRGYS	S	1145
2914	LOC Os06g51250.1	ATPSDSKTVAAKSPPVSPVKNTKDG	S	805			2915	LOC Os06g51250.1	DSKTVAAKSPPVSPVKNTKDGHSDE	S	809

2917	LOC Os06g51250.1	SNDTFGRFDSFRSNADQGGGNSFTR	S	1112			2918	LOC Os06g51250.1	AIESVSNNGGADSPVHGEKRDDSHY	S	848
2920	LOC Os06g51250.1	SFGQRDSFSRFDSFRSNADQGSNDT	S	1091			2921	LOC Os06g51250.1	QRDSFSRFDSFRSNADQGSNDTFGR	S	1094
2923	LOC Os06g51400.1	LSDSDAYFPSIASDSESEGVRSKTP	S	132			2924	LOC Os06g51400.1	DSDAYFPSIASDSESEGVRSKTPMG	S	134
2926	LOC Os07g01110.1	LGSGSTRILLIVSLLLCLRQQAVVD	S	17			2927	LOC Os07g01320.1	STSRDRDVDDADSDLEEEIQDVPRG	S	84
2929	LOC Os07g01550.1	DNQYFDVDPDELSPRTFSIKRIEDW	S	421			2930	LOC Os07g01550.1	DSKESGHLKKSLSLGNMLHKDHDHH	S	112
2932	LOC Os07g01880.1	WPVASGRRGVVASESEGSEDDYYAG	S	172			2933	LOC Os07g01880.1	GRRGVVASESEGSEDDYYAGRGHED	S	177
2935	LOC Os07g01880.1	KYTQPARQRRQLSPGFLEDALDEDE	S	496			2936	LOC Os07g01880.1	EDVVADEDMRYESDENRELKPKEKP	S	267
2938	LOC Os07g01880.1	VVDDDDDEDDVRSEGHARSGYHSEE	S	70			2939	LOC Os07g01920.1	RTFTTDRMGRQLSSMGFDPSAAMDR	S	551
2941	LOC Os07g02230.1	RILIENDDGSCGSETKNVVYHEILD	S	1399			2942	LOC Os07g03070.1	EAPRDEQRDPPPSPPNPSEEAGAGE	S	21
2944	LOC Os07g03070.1	GKDDNASAADGGSEKSAPEGEIEQL	S	317			2945	LOC Os07g03070.1	STGKGQEEENGDSDKDDDEMVGDED	S	175
2947	LOC Os07g03230.1	DADIEDPEKMVMSESEEEGDDDEEG	S	212			2948	LOC Os07g03230.1	DIEDPEKMVMSESEEEGDDDEEGDQ	S	214
2950	LOC Os07g04240.1	DGKIYQRAFGGQSLDFGKGGQAYRC	S	161			2951	LOC Os07g04530.1	LLVQKRLLERQKSLQNPGSFWSGDS	S	665
2953	LOC Os07g04530.1	TTSDQQLMAVSKSVDGLESVGDSRN	S	1298			2954	LOC Os07g04530.1	SKSVDGLESVGDSRNRRSQLHDLLA	S	1308
2956	LOC Os07g04530.1	DLQDDAEELFWGSPIQVESSSKSAD	S	1185			2957	LOC Os07g04530.1	LGLESSLVLPAKSFSEDYDTNKGEW	S	1212
2959	LOC Os07g04530.1	TSQQPRQETLVGSDQGGAKHDLQDD	S	1165			2960	LOC Os07g05050.1	SRGFGDFDGFGSSPNRGGRSRDAGS	S	512
2962	LOC Os07g05190.1	PSKGSRFSWSPDSGEAYTQEGLARL	S	743			2963	LOC Os07g05190.1	GFSPSKGSRFSWSPDSGEAYTQEGL	S	740
2965	LOC Os07g05190.1	LTGRCAGDVVSGSEGGVDLTDWVRL	S	995			2966	LOC Os07g05190.1	SQEEKLSGVGGFSPSKGSRFSWSPD	S	730
2968	LOC Os07g05610.1	SRNARVYSGIDLSKDEVTQIAKLPA	S	820			2969	LOC Os07g05610.1	PAAESNAREIGGSSMNGGEEVLNAE	S	663
2971	LOC Os07g05610.1	QPTGSELGGFAASFESRGPSSRKRN	S	476			2972	LOC Os07g05610.1	GSELGGFAASFESRGPSSRKRNLEE	S	479
2974	LOC Os07g05610.1	GIGHESNLQHSLSRNARVYSGIDLS	S	808			2975	LOC Os07g05610.1	PRGFNLFDVNQPSSSGAGPSRNLSF	S	548
2977	LOC Os07g05610.1	GFNLFDVNQPSSSGAGPSRNLSFDL	S	550			2978	LOC Os07g05610.1	PDMRKGVLTRGISATSGINGWVAEG	S	390
2980	LOC Os07g05610.1	MGRDLMIGQPTGSELGGFAASFESR	S	468			2981	LOC Os07g05610.1	RWLPNVQDWEENSTRSAKHTASADP	S	293
2983	LOC Os07g05610.1	PSSRKRNLEEGGSTADKPLNRLHPA	S	494			2984	LOC Os07g06130.1	VRDNLQGSAFLGSSRDLMSTKNEMS	S	750
2986	LOC Os07g06130.1	NSLRHSSLVATNSIPRQIRGSFTTA	S	1215			2987	LOC Os07g06740.1	SSTAPERVTIADSDLSSSTPNKGGN	S	65
2989	LOC Os07g07000.1	LTDSFELPRGGSSRDGDIEMGMQAD	S	17			2990	LOC Os07g07040.1	AKIVDKEEVKLDSDDGAKSAVKSKD	S	66
2992	LOC Os07g07194.1	HRLRFVDSSPLNSYSSKEDLVMMPK	S	253			2993	LOC Os07g07490.1	PVKGQELSASDSSLSVMKDQYNRAD	S	332
2995	LOC Os07g08190.1	NRYGRNGPYGARSPVRRYRGSPRAS	S	497			2996	LOC Os07g08190.1	DKGKQTATLDNISNEGSKPSNTDGN	S	366
2998	LOC Os07g08190.1	AGIRDDPGARARSSPIRADASLTKV	S	392			2999	LOC Os07g08190.1	GIRDDPGARARSSPIRADASLTKVD	S	393
3001	LOC Os07g08190.1	FARRYRTPSPERSPVRSRYNDGRND	S	467			3002	LOC Os07g08330.1	KAAGKAWYKTMISDSDYAEFDNFSK	S	387
3004	LOC Os07g08330.1	RPDVVRFVHRLLSCNKRQPYAVSRR	S	49			3005	LOC Os07g08710.1	GANLPPNFRKLLSGNLKKLTAAGKL	S	122
3007	LOC Os07g08960.1	SSGGGGGRTGRYSPPSRYGSGTGGG	S	155			3008	LOC Os07g09010.1	ITVLEMSKETERSPRPSSPAPAETS	S	328
3010	LOC Os07g09010.1	EMSKETERSPRPSSPAPAETSWVKG	S	332			3011	LOC Os07g09340.1	PAATAAVFRDMTSYNDLNQLAEEAR	S	907
3013	LOC Os07g09384.1	PEPTLARIESTESFSLPLHKVDGRA	S	235			3014	LOC Os07g09384.1	PTLARIESTESFSLPLHKVDGRAIH	S	237
3016	LOC Os07g10350.1	ASVLPLQVSLDESEGSDQENDNKGQ	S	561			3017	LOC Os07g10350.1	LPLQVSLDESEGSDQENDNKGQEIA	S	564
3019	LOC Os07g10460.1	NDLLFKRVLVRRSYDEEEGDDIDHK	S	260			3020	LOC Os07g10600.1	GGAPVIDAAMSRSGAMDLASGLGGK	S	53
3022	LOC Os07g10770.1	GGGGDVGRPKYDSGEIGLTKYDSGE	S	131			3023	LOC Os07g10940.1	AKKAGFLRRRRRSPPLREFAASLEE	S	632
3025	LOC Os07g12220.1	HEVNYDGRNSFLSPRSSSVRRNSMA	S	162			3026	LOC Os07g12250.1	YPGKGIRFIRADSQVFLFSNSKCKR	S	28
3028	LOC Os07g12250.1	RFIRADSQVFLFSNSKCKRYFHNRL	S	34			3029	LOC Os07g12490.1	YNHQGFSPARTLSPQIRSNPEADSQ	S	18
3031	LOC Os07g13240.1	DFTMPSETTIKASNVAPESEAKVED	S	47			3032	LOC Os07g13340.1	VKANNGGAKPKASNEANTTRMVNQN	S	1072
3034	LOC Os07g18300.1	EKCCDAKVRRTFSKIDLKLIERLYL	S	808			3035	LOC Os07g19320.1	MEMQDLEALARISELRFLSVNVEAG	S	788
3037	LOC Os07g22024.1	NSKHGEEEDIIGSPEDEEFDHPSLR	S	464			3038	LOC Os07g24020.1	GKRELQPAYKDLSGDEVHSMVKDIS	S	397
3040	LOC Os07g24020.1	SGDEVHSMVKDISNEFGKKRKRSKT	S	409			3041	LOC Os07g25460.1	ELQPLEEFTTPPSSPDNSKSPVAQS	S	577
3043	LOC Os07g25460.1	EKKQLEAALKMDSPDGAGGEGQSDT	S	353			3044	LOC Os07g25550.1	DYKEELRVPGNKSGSNMGARGSSHP	S	79
3046	LOC Os07g25680.1	LKSSLQPSDSAKSLETYTASKAMSA	S	509			3047	LOC Os07g25680.1	SIQEGLKSSLQPSDSAKSLETYTAS	S	504
3049	LOC Os07g25680.1	AGSLNRLSKSDDSLNSQFLILQSQS	S	587			3050	LOC Os07g25680.1	KRIKKSCFTGRSSELERLANEFWRE	S	980
3052	LOC Os07g25680.1	IPREQAGSLNRLSKSDDSLNSQFLI	S	582			3053	LOC Os07g25810.1	GGGIGKGGGLGGSFGKGGGLGGGFG	S	334
3055	LOC Os07g26440.1	SDSKESEVLDEGSDGSSSERGSSHG	S	366			3056	LOC Os07g26440.1	KESEVLDEGSDGSSSERGSSHGHKS	S	369
3058	LOC Os07g28430.1	PVQLVVEDDEPDSTSGSKPASGKAT	S	230			3059	LOC Os07g28800.1	NFRSRGGFEFCTSDEDEPETVFRNA	S	144
3061	LOC Os07g29360.1	SAIERKRRAWSESEDDEPVQRPVQP	S	1048			3062	LOC Os07g29360.1	PGHLLAAAGLEDSDAEEDDMGHPQS	S	1024
3064	LOC Os07g29450.1	NDRRIGIQLGGNSVERRENQREDRD	S	274			3065	LOC Os07g30300.1	WRQKATNFFSSSSFKLKQAGQSAGD	S	21
3067	LOC Os07g30774.1	QSPSKTRLTKLQSEKFEHLSEQELC	S	700			3068	LOC Os07g30820.1	YGYGFRGSPMPVSSPWSGGALVENN	S	178
3070	LOC Os07g30820.1	FRGSPMPVSSPWSGGALVENNDSSA	S	182			3071	LOC Os07g30820.1	NRGACGAPRPSPSLSPRVPPPPAAG	S	70
3073	LOC Os07g30820.1	VCNRGACGAPRPSPSLSPRVPPPPA	S	68			3074	LOC Os07g31450.1	LKVKKHLHPMELSPKKYKNKKQHNH	S	198
3076	LOC Os07g31450.1	RETFATIPNEALSEDSDEEDEPKRE	S	1268			3077	LOC Os07g31450.1	FATIPNEALSEDSDEEDEPKREYTA	S	1271
3079	LOC Os07g32350.1	DEDETRMLDHDDSDSEMHTSKRKKS	S	404			3080	LOC Os07g32350.1	DETRMLDHDDSDSEMHTSKRKKSGE	S	406
3082	LOC Os07g32430.1	MLAAASPDARLVSPWLGGNTPRYAA	S	275			3083	LOC Os07g32480.1	PSGAEFYGDCRTSGFSCVEMQEQRA	S	234
3085	LOC Os07g32530.1	TPAAASGSRGGCSDSDDNYEEAEML	S	65			3086	LOC Os07g32530.1	AAASGSRGGCSDSDDNYEEAEMLRQ	S	67
3088	LOC Os07g33630.1	RRGGGPMLGFDSSPGRSSMKYNGFH	S	593			3089	LOC Os07g34740.1	EERLALLLQGDKSEEEQTDGEDMEI	S	399
3091	LOC Os07g35580.1	GSFVGTLPSSGRSAPMSPNEVSITE	S	680			3092	LOC Os07g35580.1	GTLPSSGRSAPMSPNEVSITELEPR	S	684
3094	LOC Os07g36200.1	GIDALASDLDKTSPVGRSKSEFAKG	S	381			3095	LOC Os07g36200.1	GIERSVAPVRPASHEGRVGEARGLE	S	225
3097	LOC Os07g36420.1	GAAKNPEVETSSSIHRSENPGLRTT	S	170			3098	LOC Os07g36600.1	VKAPTPRPPTPASLQPESPGVFFTA	S	32
3100	LOC Os07g36940.1	NKESEQTNAASASPTEMLKAADASS	S	692			3101	LOC Os07g36940.1	TRNLSQEPQSASSAEDLKVHTSVKD	S	654
3103	LOC Os07g36940.1	TDQGQLLKEPSSSVSDENVIMDRSH	S	801			3104	LOC Os07g36940.1	QGQLLKEPSSSVSDENVIMDRSHQS	S	803
3106	LOC Os07g36940.1	MLKDQSSSAPAASARPVSREKPSVE	S	897			3107	LOC Os07g36940.1	SSSAPAASARPVSREKPSVEITRTK	S	902
3109	LOC Os07g36940.1	NLAGKSYVVDHPSPGRGADRPASRG	S	1065			3110	LOC Os07g36940.1	GRIAPNTQFAGPSNRPASQEGRSGN	S	1584
3112	LOC Os07g36940.1	DFIGASESLDSSSIADHELPDESSE	S	962			3113	LOC Os07g36940.1	SSSIADHELPDESSEKEVNMGEDEG	S	972
3115	LOC Os07g37140.1	RRRRRKAGDRGDSPPPLPDHLRCRR	S	282			3116	LOC Os07g37140.1	EAEADGDEGEDKSDEMDAEAGAAEE	S	105
3118	LOC Os07g37140.1	DLPNGLMRISPGSSEPAASLPSPVT	S	380			3119	LOC Os07g37140.1	KNKRKGRRNVNASPASPPAATSDVT	S	354
3121	LOC Os07g37610.1	DLNSQPEREDEQSPKSDATRLLRDN	S	941			3122	LOC Os07g37650.1	NGAASAAANKSTSPTSSSLPSQSGK	S	449
3124	LOC Os07g37750.1	DKAVELEDGKKVSGGIDLDTVNIIQ	S	260			3125	LOC Os07g37750.1	QADEKITLGDALSLNRATEASPASV	S	216
3127	LOC Os07g38090.1	DEGNRSPSSRSSSLSPITVDRGKKE	S	203			3128	LOC Os07g38090.1	AAHWLQGSRLRSSFNARDAAVDDLG	S	404
3130	LOC Os07g38120.1	KASRQYSRERFTSLSLKLQKDGSLQ	S	533			3131	LOC Os07g38120.1	SRQYSRERFTSLSLKLQKDGSLQLT	S	535
3133	LOC Os07g38530.1	ERRMKSSLNKLQSFHIGKNHNFRIP	S	615			3134	LOC Os07g38970.1	KALREAGVTVVESPAKIGSTMFEIF	S	312
3136	LOC Os07g39280.1	EKKASAPIPRNKSDMLDDSEDVPLK	S	315			3137	LOC Os07g39470.1	DSSEGAGCMRHDSPSSQSFTTRSGS	S	85
3139	LOC Os07g39470.1	AGCMRHDSPSSQSFTTRSGSPLSQE	S	90			3140	LOC Os07g39480.1	IMNKSANPDILPSPRDKTSGSTHED	S	109

3142	LOC Os07g39560.1	VPKEQSPGPAARSPAGGQNYAMSRV	S	194			3143	LOC Os07g39560.1	GSFYNGSSNRLGSPIGYVGLNDDSG	S	292
3145	LOC Os07g39620.1	EVESSRTVQSSASPAANSPGAMSES	S	329			3146	LOC Os07g39620.1	RTVQSSASPAANSPGAMSESAVDKP	S	334
3148	LOC Os07g39620.1	THVAGVRVVQSFSTDNSTADSAGTF	S	255			3149	LOC Os07g39620.1	AVSSAEQKEKAASDAADAEVESSRT	S	311
3151	LOC Os07g41180.1	AKVTDNVEKAVSSPVKPTNAADTTS	S	295			3152	LOC Os07g41180.1	SLQETLLTETNDSPQERLLTEINDS	S	148
3154	LOC Os07g41190.1	IEADDDSDEGLGSPVAAGAEDGNET	S	29			3155	LOC Os07g41190.1	WADLDRKAPLPGSSDNESDEEGRVD	S	206
3157	LOC Os07g41190.1	ADLDRKAPLPGSSDNESDEEGRVDD	S	207			3158	LOC Os07g41190.1	GEDDLPVYDEGLSSEDEGVDMVGRE	S	126
3160	LOC Os07g41230.1	AAAAALVLGQRSSFGGGGGGGGGAF	S	61			3161	LOC Os07g41694.1	EAALQAAGEGSSSPARSLTLDGAVK	S	22
3163	LOC Os07g41694.1	GAKRKRDDDPSGSGDDDEDDDGVED	S	457			3164	LOC Os07g41800.1	PSPIRNSPSRSLSFSHLSRLRGRVH	S	179
3166	LOC Os07g42300.1	GQGVKVESTAVPSASTPDVADAKAP	S	86			3167	LOC Os07g42400.1	LVVRQIGFLRSISLLPADYKNYLRS	S	273
3169	LOC Os07g42410.1	AAPRTQPKSFIRSKSIIEESKNISK	S	982			3170	LOC Os07g42410.1	PRTQPKSFIRSKSIIEESKNISKDQ	S	984
3172	LOC Os07g42410.1	NAEMRAKFSRSSSVPDSTYISRCAH	S	851			3173	LOC Os07g42410.1	QSRKSQSMRKNLSPGELRDATSMND	S	1008
3175	LOC Os07g42410.1	IHQGTNLSRQSSSAEQELSLHGGKV	S	667			3176	LOC Os07g43030.1	FKIEPDGLKKFDSFSRWMSSELPEV	S	369
3178	LOC Os07g43030.1	TDGIRYPLLKQSSLDLFKIEPDGLK	S	353			3179	LOC Os07g43030.1	EMHLHIRLEKLLSLGPDDYEKYVMS	S	548
3181	LOC Os07g43240.1	LDAAEKEEEEKDSEAEAEAEARISK	S	104			3182	LOC Os07g43316.1	QDTDMSLKKKEGSPDSIYPEKLNLD	S	195
3184	LOC Os07g43316.1	EKLNLDRSSGDESMEEDAMETKHMD	S	214			3185	LOC Os07g43316.1	DSIYPEKLNLDRSSGDESMEEDAME	S	209
3187	LOC Os07g43316.1	LEGKSEVTLEHVSSGDESMEEDVME	S	246			3188	LOC Os07g43316.1	EGKSEVTLEHVSSGDESMEEDVMET	S	247
3190	LOC Os07g43316.1	LEESSAKGEPCVSIGCEILEQETKS	S	29			3191	LOC Os07g43316.1	LGLESKQDIVKPSSSNPSSVGDDLQ	S	155
3193	LOC Os07g43316.1	QDIVKPSSSNPSSVGDDLQTPDDDK	S	161			3194	LOC Os07g43316.1	LESKQDIVKPSSSNPSSVGDDLQTP	S	157
3196	LOC Os07g43316.1	NTKVEHVSSGDESMEEDVMETKHVD	S	277			3197	LOC Os07g43316.1	HVDSNTKVEHVSSGDESMEEDVMET	S	273
3199	LOC Os07g43316.1	EMITESLILKKDSNENDLMYEKDQK	S	71			3200	LOC Os07g43350.1	RFHEEPVAFEVYSPDRLVGELIFLD	S	754
3202	LOC Os07g43810.1	GPPPPRDDFAPRSPRGGGSNFDSSN	S	168			3203	LOC Os07g44190.1	ASVPVKKIKVEESAEEVEGEKSEKK	S	507
3205	LOC Os07g45360.1	PPAPDVASAAAPSPEKKRKRQSKAK	S	44			3206	LOC Os07g45400.1	LKEVPPSFREQSSPGLIAGVTQVKM	S	310
3208	LOC Os07g46180.1	RPTSSMDHNVTKSPGSNIPAVESGR	S	172			3209	LOC Os07g46180.1	LKVKGCAASGASSPSNNSLDAIAKV	S	532
3211	LOC Os07g46540.1	PNIDSLSRASMSSPSDGIPVTDRIA	S	115			3212	LOC Os07g46590.1	VVEPEAREEGEISESEAETKYRQDK	S	762
3214	LOC Os07g47030.1	KLLSYFDGEGDISPEIMDNQNRWPV	S	601			3215	LOC Os07g47100.1	RLLLPASGHPVTSEPSSPKSLHSPL	S	454
3217	LOC Os07g47100.1	LPASGHPVTSEPSSPKSLHSPLLTS	S	457			3218	LOC Os07g47180.1	HAHAHAEVRIGGSFANKARGEQVAA	S	89
3220	LOC Os07g47350.1	RSSRSETLRSLQSIYEQESGSLSRR	S	710			3221	LOC Os07g47360.1	QRRKQNFHVAKTSPIESVSSSPPRI	S	1089
3223	LOC Os07g47840.1	RATDGGSAADRLSDVPGKVAEDTAD	S	190			3224	LOC Os07g48020.1	VRNFASNAAAFSSAFTTAMVKMGNI	S	286
3226	LOC Os07g48410.1	HQMPMFDGGECRSPGGGDGGLFSYN	S	191			3227	LOC Os07g48602.1	TVQLGKITETVQSPVKAKPEVAFQP	S	119
3229	LOC Os07g49040.1	IPSDYRLTSPQLSPKRNQSKFKSLL	S	310			3230	LOC Os07g49270.1	VPTVSAFETIEDSDDDDENIAPDAK	S	126
3232	LOC Os07g49270.1	KPVPSTNMIRSQSATGSLHGAQHNP	S	178			3233	LOC Os07g49270.1	STNMIRSQSATGSLHGAQHNPVAAD	S	182
3235	LOC Os07g49380.1	EDWDPRKKGATDSDSEVDFDRKRVS	S	262			3236	LOC Os07g49380.1	QEVAPQDEASADSPAVKQADGPAAL	S	470
3238	LOC Os07g49460.1	MPSLELSLKRSRSTGDGANAIQEEQ	S	416			3239	LOC Os07g49460.1	NDDFKGKDLEIGSPRNLNTAYQSSP	S	316
3241	LOC Os08g01054.1	KNLLEIEHEHGKSPKTRQSSDSLSG	S	807			3242	LOC Os08g01100.1	AELKAEYHNGERSDENNVGGNAGEQ	S	159
3244	LOC Os08g01794.1	PSKQETVLENVESETALEPRSSQEL	S	211			3245	LOC Os08g01900.1	HPTVAAKGNIPGSPEADLYMRSLNR	S	345
3247	LOC Os08g01900.1	SERGFPRIISSSSMDTRSDLSAIEN	S	527			3248	LOC Os08g01930.1	KDAEADANAPTRSVELVGTLESIDK	S	201
3250	LOC Os08g01930.1	IKNVIAEADAGGSPALIARGFGSGQ	S	230			3251	LOC Os08g01930.1	PANPQPAVAKGVSPQPAGYGGQWTA	S	688
3253	LOC Os08g02410.1	KRTPPSWLKTAASDVEEMIMKAAKK	S	31			3254	LOC Os08g02690.1	QNAETLSLASPRSPTGGSTSALLQQ	S	35
3256	LOC Os08g02690.1	IAVQNAETLSLASPRSPTGGSTSAL	S	32			3257	LOC Os08g02690.1	DACLDRNDPNYDSDEEPYELVEAPV	S	118
3259	LOC Os08g03360.1	TKPALQPKKRKASPLVQGSLEIPVV	S	21			3260	LOC Os08g03390.1	EKDGEEGDENVASEEEDEEDGLKID	S	193
3262	LOC Os08g03520.1	LNDGDVVEFSVGSGNDGRTKAVDVT	S	58			3263	LOC Os08g03520.1	PGGGALTGGSRPSGGGDRGYGGGGG	S	84
3265	LOC Os08g03840.1	SDEPCAKDDAKQSDVAPLKEEENWE	S	106			3266	LOC Os08g04270.1	EIVLWEPKTKEQSPGEGSIDILQKY	S	277
3268	LOC Os08g05660.1	RSRGKRDPDTCNSPTGLSPTNSTAR	S	327			3269	LOC Os08g05840.1	PTNAALKRGGNVSDDSEDEKPLAAR	S	211
3271	LOC Os08g05840.1	KTTPKIEQPGDDSEDDKPLASRLPT	S	188			3272	LOC Os08g05840.1	KSKLKRPFVEDKSDDSEDDHKPIGL	S	115
3274	LOC Os08g05840.1	KKPAVGSGTANDSDDDKPLSLKINS	S	149			3275	LOC Os08g06020.1	ESEEGSPSRVSESENGDSLGGGFTR	S	156
3277	LOC Os08g06020.1	LRVTYIAPPQPPSPVPEESEEGSPS	S	139			3278	LOC Os08g06020.1	APPQPPSPVPEESEEGSPSRVSESE	S	145
3280	LOC Os08g06070.1	SSSMHENLKRQRSSVDDDLYDHPKH	S	423			3281	LOC Os08g06070.1	SSMHENLKRQRSSVDDDLYDHPKHS	S	424
3283	LOC Os08g06140.1	FLNQTIADPDEHSSTTSKAQYDSDT	S	335			3284	LOC Os08g06140.1	LNQTIADPDEHSSTTSKAQYDSDTG	S	336
3286	LOC Os08g06140.1	TILRRRHAPTASSFSDGDDAESTGI	S	537			3287	LOC Os08g06140.1	LRRRHAPTASSFSDGDDAESTGITI	S	539
3289	LOC Os08g07760.1	LGPHRNSEWIVDSTDNLHAVELSGP	S	611			3290	LOC Os08g07830.1	IPTPFAQPSMPVSPNEKKHFVPFAS	S	373
3292	LOC Os08g08080.1	DISPPRRRTRHDSEEPQDLSPPRRK	S	237			3293	LOC Os08g08080.1	KGSEVAGGHDDLSPPRKSRQQRDPS	S	198
3295	LOC Os08g08080.1	PLKRQALGDGDISPPRKGRKEVAPK	S	326			3296	LOC Os08g08080.1	RTRHDSEEPEDLSPPRRRTRHDSHE	S	295
3298	LOC Os08g08220.1	SHRVLPPSFGRNSSANHSEFANGID	S	223			3299	LOC Os08g08240.1	NEARDVEMDGFDSDSLEDWDQHLSA	S	684
3301	LOC Os08g08820.1	DAGGLVDEFESKSCSENVDGAGDGL	S	82			3302	LOC Os08g08820.1	CSENVDGAGDGLSGDDQDPNQRPRK	S	95
3304	LOC Os08g08830.1	SVLRGIEDELQFSPLENKRSATATD	S	167			3305	LOC Os08g09350.1	IFVKGFDSSLEESKIRESLEGHFAD	S	425
3307	LOC Os08g10580.1	FSKTRSGLVRINSTVESNEVSLSKT	S	41			3308	LOC Os08g12140.1	MNTKNQFDFKTKSIGGWSGYVAAYM	S	123
3310	LOC Os08g13350.1	SGPLGNQPSRNTSFGGAGSNSGPVS	S	74			3311	LOC Os08g13350.1	PLNKHGEPGKKSSGPQSGGVTPMAR	S	129
3313	LOC Os08g13350.1	KMFDLHVEKSRKSGPLGNQPSRNTS	S	62			3314	LOC Os08g13350.1	GPLNSSGAPRKVSGPLDPSVSMKMR	S	177
3316	LOC Os08g13350.1	GSISSSVPGAGGSARAKSNSGPLNK	S	108			3317	LOC Os08g14340.1	ERKDRKSANMIISEGGGTSGYSKIL	S	386
3319	LOC Os08g14460.1	KHFTKSDKPRRLSGLDAEREAVAAQ	S	413			3320	LOC Os08g14460.1	PAVVGNGRQEAGSPQSPLGGKTDVF	S	320
3322	LOC Os08g14570.1	KPDEAAHLERNFSLANGYAVHDAQH	S	305			3323	LOC Os08g15840.1	HKSSVEGILTKRSEVPVRILPSSYL	S	280
3325	LOC Os08g16559.1	PHRRSQNTFFLSSPKKLQSSKDDVG	S	21			3326	LOC Os08g16610.1	ADGQPEPELNPKSPGEAGTSHFDDM	S	536
3328	LOC Os08g17120.1	IDPSVMNFFRDMSAPAKEAIKLSDY	S	496			3329	LOC Os08g17120.1	DMSAPAKEAIKLSDYERTLKKASSR	S	506
3331	LOC Os08g19310.1	DAAREVFDEMRESRNVALNEYSYTA	S	214			3332	LOC Os08g20020.1	QELGVDVVRRRSSFASLEKAYYSMS	S	413
3334	LOC Os08g20660.1	RRYAAADMSEDLSEGEKGENINESS	S	142			3335	LOC Os08g20660.1	REKARRYAAADMSEDLSEGEKGENI	S	138
3337	LOC Os08g20660.1	DESTRGRMPRIGSTDAIEAWASQHK	S	170			3338	LOC Os08g20660.1	LSRGVSANRKTESVENMEATTGNKW	S	761
3340	LOC Os08g20660.1	ATEVSEADSPGDSLRDVHDISLNLK	S	711			3341	LOC Os08g20660.1	PRWQKSDDATEVSEADSPGDSLRDV	S	703
3343	LOC Os08g23200.1	SGSGSTMGGHARSILNAARVIPPND	S	61			3344	LOC Os08g23360.1	EENVPERLSKGVSTLSLGERKQAFL	S	539
3346	LOC Os08g23440.1	KSMTGEQIQAPSSPRDGEDVAITIG	S	111			3347	LOC Os08g23680.1	YNTILNRYGISKSDLSRSKDSSGDA	S	246
3349	LOC Os08g23930.1	PSGRGPIMNKNLSGGLPTEPISVNP	S	231			3350	LOC Os08g24434.1	APAGASSGGGEQSGDDEAAAAAAQD	S	98
3352	LOC Os08g25080.1	NINDTPMDESQASDEARYSGKNGGG	S	155			3353	LOC Os08g25080.1	VPVSVVEDERQESGKSDDENKANDG	S	116
3355	LOC Os08g25090.1	NRRRRGTKRTAFSDSDTEDLDLSKE	S	135			3356	LOC Os08g25090.1	RRRGTKRTAFSDSDTEDLDLSKEDL	S	137
3358	LOC Os08g25670.1	WLSRTVRVAFAASGDRQWRVSSWSM	S	207			3359	LOC Os08g25850.1	ETKSNQPPTKKASSKPAPKKNDKVQ	S	49
3361	LOC Os08g28670.1	MLSGAEYLEGDGSPGSLRIFKLGPA	S	46			3362	LOC Os08g29124.1	SVDQGAICPRSDSPSVASLPKEETS	S	704
3364	LOC Os08g29500.1	AAYEHHKHDTLDSPKASKFTGVEMT	S	105			3365	LOC Os08g29650.1	MSQSKEVRYTARSITPPADRNGTSK	S	13

3367	LOC_Os08g30060.1	TAGGAESEEAAESDAKSDEAEAQEE	S	536			3368	LOC_Os08g30060.1	AESEEAAESDAKSDEAEAQEEEPAA	S	540
3370	LOC_Os08g30060.1	VEKFMAQWCSSKSFREDYEKRILVS	S	355			3371	LOC_Os08g31470.1	TNVEERQFLIGMSPAREGQEANGDL	S	539
3373	LOC_Os08g32100.1	ERKREIMKQEDDSDEEDNRKRRRRS	S	535			3374	LOC_Os08g33082.1	RRRASPAAEKGKSPAKVEMESALED	S	53
3376	LOC_Os08g33320.1	ENSNNSRTTWRKSPDYYNYGLPGPR	S	1080			3377	LOC_Os08g33320.1	NGKHSRNGGGDVSPAALKCSWSTTC	S	877
3379	LOC_Os08g33380.1	LQVQAMRSSEESSSDEDDEILSELK	S	63			3380	LOC_Os08g33380.1	QVQAMRSSEESSSDEDDEILSELKE	S	64
3382	LOC_Os08g34060.1	QQADYDLMTRGVSWNRSQNENELNL	S	167			3383	LOC_Os08g34800.1	GPSPARTVTRTSSAASSELLRTSSA	S	102
3385	LOC_Os08g35060.1	SVLSHGRNDDDDSADGDDGELFPIS	S	37			3386	LOC_Os08g35110.1	EEGSAAAAGRAASFHGADEVPKGLH	S	46
3388	LOC_Os08g36760.1	PEISRKDVATQTSPELSRSSSPSGR	S	415			3389	LOC_Os08g36760.1	RKDVATQTSPELSRSSSPSGRPSFS	S	419
3391	LOC_Os08g36790.1	VDEVWRDLEREASPGAAAADGGGGG	S	73			3392	LOC_Os08g37104.1	TNVKQTPRRTIPSMNHGGDRPGKEN	S	182
3394	LOC_Os08g37280.1	SKKVRKQKGLKESPASEHMGEDTKD	S	146			3395	LOC_Os08g37280.1	VRKQKGLKESPASEHMGEDTKDQHS	S	149
3397	LOC_Os08g37280.1	KKELSHVNSPCDSTEEAGNLKAKTS	S	121			3398	LOC_Os08g37280.1	MEAFHEAVKKELSHVNSPCDSTEEA	S	113
3400	LOC_Os08g37444.1	QKKKGWFSSMFKSIAGNNVLEKSDI	S	326			3401	LOC_Os08g37920.1	LGVADRSSIAARSFNAGYVNENGAS	S	653
3403	LOC_Os08g39140.1	SLWTEKTTEKEISDDEDEEEKKDAE	S	220			3404	LOC_Os08g39140.1	VSATKEGLKLDESEDEKKRKEELKE	S	527
3406	LOC_Os08g39390.1	PSPPLREALPLLSLSPTPARRGGVV	S	97			3407	LOC_Os08g39390.1	PPLREALPLLSLSPTPARRGGVVDA	S	99
3409	LOC_Os08g39630.1	PGGSGGRLKGQLSFSSRQGSLMSQI	S	206			3410	LOC_Os08g39630.1	DFLPPAAAPRAASPDADNVFSRFLA	S	53
3412	LOC_Os08g40150.1	LMAGRDLFGMPKSPPAAAAAPPQSV	S	21			3413	LOC_Os08g40230.1	EAVLERKLADVHSETDDELIEELRS	S	89
3415	LOC_Os08g40510.1	RGLSNFYAGKSKSFTSLAEAAAKAA	S	131			3416	LOC_Os08g40510.1	SSNPASSFRSPRSFSLSDLQNAGSY	S	248
3418	LOC_Os08g40830.1	TESAVRLIGGTGSGNWSKDFGAFDS	S	808			3419	LOC_Os08g40830.1	LSTHREEPEDDRSPRLDSSSAEDAQ	S	971
3421	LOC_Os08g41340.1	KILNSDSYKAELSLNRVSIEGDSKD	S	193			3422	LOC_Os08g41400.1	GNVADDDGNAERSSSPPHVSEVQVG	S	140
3424	LOC_Os08g41400.1	STSSTSHEIDKVSSKDGIDERNPNV	S	520			3425	LOC_Os08g41710.1	SRVRKYLSPEDWSPTETKGRPWYGL	S	298
3427	LOC_Os08g42600.1	PKKVSAAHNVYVSPLRTSKMDTLLS	S	916			3428	LOC_Os08g42600.1	YDVMASPAKSITSPSPMSPPRFCAS	S	392
3430	LOC_Os08g42600.1	SPLRTSKMDTLLSPSSKSYYACVGE	S	928			3431	LOC_Os08g42600.1	SPSPMSPPRFCASPTGNGYCSSKMA	S	404
3433	LOC_Os08g42600.1	CTERRNVLVDNNSFRSPVKDTLKSK	S	693			3434	LOC_Os08g42600.1	EKCAADAGPYPESPRLSRFPNLPDM	S	890
3436	LOC_Os08g43060.1	ELRDRTGAPKDGSQTAQESSAESTL	S	299			3437	LOC_Os08g43060.1	EQQKAQDSSSIVSPIRKFEHQEQTN	S	518
3439	LOC_Os08g43090.1	ASGTRTGSVADSSENEAESHSTPVE	S	222			3440	LOC_Os08g43090.1	DGGGKSRHCRSLSVDSFIEKLNFDE	S	249
3442	LOC_Os08g43090.1	LPSPSGGLSRSGSGSLDGGAASLFG	S	279			3443	LOC_Os08g43090.1	PVVPRSPTPLDLSSAAAAAAAASYR	S	34
3445	LOC_Os08g43400.1	STIDEVAKDMCPSDAEQETLTSDIS	S	767			3446	LOC_Os08g43410.1	FKGILHDVGPEHSSGGGGGMGGRHA	S	230
3448	LOC_Os08g43560.1	GGHTLGRAHPERSGFEGAWTQEPLK	S	170			3449	LOC_Os08g44420.1	KDYEPTGETPVRSEPEVPSKDAIES	S	357
3451	LOC_Os08g44480.1	WSKGKQKEKVNNSVLFDQATYDKLL	S	40			3452	LOC_Os08g44540.1	EEKTIAQEVNDDSDDDSGETESRTS	S	111
3454	LOC_Os09g01640.1	RSSSRGRSKHRRSTKRSDTEDDLEE	S	233			3455	LOC_Os09g01640.1	RGRSKHRRSTKRSDTEDDLEEERSK	S	237
3457	LOC_Os09g02810.1	CNDILATLEKCNSDAEDACDTIECV	S	663			3458	LOC_Os09g03600.1	IENCEKALVEKDSDGEDSVAPVVTK	S	722
3460	LOC_Os09g03610.1	PSRWSSGGGGGGSGSPPHRFSRGGG	S	43			3461	LOC_Os09g03610.1	RWSSGGGGGGSGSPPHRFSRGGGGG	S	45
3463	LOC_Os09g03610.1	GRHMPPDSWHPSSPRSAPHQFNNFG	S	345			3464	LOC_Os09g06560.1	RKSTYHDHSNGDSEDEDLILQMESR	S	499
3466	LOC_Os09g07294.1	TSYLSSSPSTPESPVRLSALSGARG	S	521			3467	LOC_Os09g07300.1	LMGFKKPLIKLVSSIGEQEIDSQQK	S	3559
3469	LOC_Os09g07300.1	ALKRLSSKSSGNSFGSGSLIPKQLK	S	1524			3470	LOC_Os09g07300.1	KPQMSCTKQVTLSELESVASHEEEA	S	541
3472	LOC_Os09g07300.1	GSLIPKQLKNSDSLVLRTNQESNST	S	1540			3473	LOC_Os09g07690.1	PPRKDASADLGPSSPAKVQEPARPR	S	494
3475	LOC_Os09g07900.1	GDASVSNLSERDSDDDTNAASVTRH	S	859			3476	LOC_Os09g08450.1	EERQYSMIRIIDSLNASSNCSGTRH	S	113
3478	LOC_Os09g10710.1	SLIDAIARLAEASDGESDEHNRVRR	S	366			3479	LOC_Os09g10710.1	AIARLAEASDGESDEHNRVRRNAPY	S	370
3481	LOC_Os09g11170.1	EGDLAVVEMAKGSRWRRCPRCKFFV	S	283			3482	LOC_Os09g11240.1	NLNLGLEDPDDDSDADLSAEEQRDS	S	128
3484	LOC_Os09g11250.1	QRFGFSSTSPELSDKEENQRKDQEN	S	81			3485	LOC_Os09g12240.1	SEIFDPTLTDRKSGEAELYQYLKIA	S	1151
3487	LOC_Os09g12270.1	RRRLKKKHPADSSDDNNDDSSHRPV	S	186			3488	LOC_Os09g12270.1	VRCKAYSMFDSCSEDEDNMSVPVSL	S	211
3490	LOC_Os09g12730.1	RMSEDPYEQHRRSPAGDAPWHNIPF	S	358			3491	LOC_Os09g13630.1	IRHVHPHIVDSGSPEDAQAGSEDDY	S	403
3493	LOC_Os09g14490.1	YETCKAGAVRLISIVAMGGMGKTTL	S	666			3494	LOC_Os09g14670.1	GGGGVGKVERLSSIDAQLRQLVPAK	S	21
3496	LOC_Os09g17730.1	ADTEASTESDNRSDGAAEAQAEDDS	S	539			3497	LOC_Os09g17730.1	SSLDADDDYEAESPKEKPKPKEVDV	S	456
3499	LOC_Os09g19640.1	IQHEIQYGGDNASASPRSQHASQST	S	93			3500	LOC_Os09g19640.1	HEIQYGGDNASASPRSQHASQSTQA	S	95
3502	LOC_Os09g19734.1	VDELTPGLKSILSFAVPDQRSGKFE	S	94			3503	LOC_Os09g19830.1	VANQGGAEGVAVSPDRSVPLSGGPT	S	81
3505	LOC_Os09g20010.1	EGINVVGFEVPTSPDSSYNNPVPGN	S	22			3506	LOC_Os09g20590.1	GLPEAEEDMFADSPKDKTTASLLDM	S	276
3508	LOC_Os09g21110.1	PDEEITEALKNCSIGQETNFKQVQK	S	988			3509	LOC_Os09g21770.1	NGTKRPSNSYPASSNSDSKVEPKKR	S	202
3511	LOC_Os09g23740.1	SYSKAIPPVRIHSFDSIFRLHQRES	S	1185			3512	LOC_Os09g24440.1	DSRLDALPKDHFSPVVNGQEVLDKP	S	1391
3514	LOC_Os09g26540.1	SDLKGAINKDGSSDEEDFTEEGLSA	S	50			3515	LOC_Os09g26560.1	PPDYRGEWDPYVSDDEFGTSFELKR	S	120
3517	LOC_Os09g26820.1	RAVDDLRRLAPPSPATVGSPRRTSS	S	67			3518	LOC_Os09g26830.1	PIASKNGWSLSSSPDTKATKNVTVT	S	508
3520	LOC_Os09g27850.1	DAAAAAAAAAGGSASEGSDAEASAE	S	23			3521	LOC_Os09g27850.1	AAAAAAAAGGSASEGSDAEASAEAA	S	25
3523	LOC_Os09g28220.1	KSMKRCIWNITPSPPRREGEDEDYG	S	146			3524	LOC_Os09g28220.1	KSRSSRRKRSKQSDSEDQAPSDADL	S	331
3526	LOC_Os09g28220.1	RSKQSDSEDQAPSDADLGVKEIDET	S	339			3527	LOC_Os09g28220.1	SKKKSRSSRRKRSKQSDSEDQAPSD	S	328
3529	LOC_Os09g28220.1	PSPYRSRRDRSPSPYRDRRRQWSPY	S	60			3530	LOC_Os09g28220.1	RRGGGGAGAGAWSASDDDDDEQLKG	S	106
3532	LOC_Os09g28220.1	SPSPYRDRRRQWSPYHRDRGRDVER	S	70			3533	LOC_Os09g29070.1	APHSIADLSRSSSSSSEQQPSSTPA	S	48
3535	LOC_Os09g29070.1	MAANDRALIAEGSCGSPRSIKVYLG	S	602			3536	LOC_Os09g29080.1	RAKDSDDLRVLISEKPAEPAPAKRE	S	53
3538	LOC_Os09g29810.1	FFRDKNSHERSDSPSLTADSAAKNN	S	1641			3539	LOC_Os09g29890.1	TQMAVAVLDRSFSSEYPASSRTEGR	S	23
3541	LOC_Os09g30070.1	EFERFDVPIQFDSPCAETKTSEALC	S	1035			3542	LOC_Os09g30070.1	DSATLKQHLQCGSPDLSPTHSRNKD	S	376
3544	LOC_Os09g30350.1	IPREHHEEGSPGSGGGGGGGGSVAA	S	58			3545	LOC_Os09g30350.1	SDGEPPSEEAARSGAGGFYRENGSV	S	292
3547	LOC_Os09g31470.1	LKMVPMSGTGPSSPPSRGTLSESSG	S	321			3548	LOC_Os09g31486.1	EITIKSSGGLSESDIEKMVREAELH	S	557
3550	LOC_Os09g31486.1	GGGGSGGGSSSSSGGDQTPEAEYQD	S	665			3551	LOC_Os09g32090.1	EELELTRKRKAESPLFEEAHSNHVS	S	286
3553	LOC_Os09g32090.1	KGQDELGENHSPSLQKSANNPNRGM	S	61			3554	LOC_Os09g32430.1	KTVGKFESPNLKSFIQQFPDTSLDQ	S	123
3556	LOC_Os09g32540.1	KKQAADTVSRETSALQNIEERENVE	S	760			3557	LOC_Os09g32540.1	PKPGADDNKFWSSPARLPENNAEKK	S	936
3559	LOC_Os09g32540.1	GGGESEGNAGSPSLGSARGDADLVE	S	88			3560	LOC_Os09g32740.1	PENGKVALLKTGSSLVDTTEQADSK	S	781
3562	LOC_Os09g32820.1	RLSSPSAPSSPSSAKAAAAAAAHSN	S	39			3563	LOC_Os09g32820.1	LEALRLSSPSAPSSPSSAKAAAAAA	S	35
3565	LOC_Os09g33450.1	VEGRAGMHSRRSSWGHRSGTSESMD	S	403			3566	LOC_Os09g33450.1	LLDEKEGRIAHASPDLSLPGELVEG	S	381
3568	LOC_Os09g33450.1	LQQTRTPARGFMSPNMGKPVGDLEL	S	331			3569	LOC_Os09g33510.1	SSSHQPSIHVRVSPNTSPPLLSSPK	S	486
3571	LOC_Os09g33510.1	QPSIHVRVSPNTSPPLLSSPKIKEL	S	490			3572	LOC_Os09g33510.1	NKEGTEAGHASRSPTEELLDRSKAE	S	289
3574	LOC_Os09g33510.1	ASDNKKVKRGSFSGPIVPRSRSTEN	S	451			3575	LOC_Os09g33510.1	SKASDNKKVKRGSFSGPIVPRSRST	S	449
3577	LOC_Os09g33510.1	SFPGERQRSGSQSAPLFPEKKLEAA	S	316			3578	LOC_Os09g33510.1	TNVGDLRDNKLPSPTRLSNAHSVLK	S	397
3580	LOC_Os09g33600.1	ISPRDSLSLASTSARIDELDDCVEG	S	731			3581	LOC_Os09g33600.1	ISEDCMLERNSGSGYFDHSGRAIQH	S	480
3583	LOC_Os09g33600.1	KALLLQEQPTSPSDGSSRCFDRSSL	S	704			3584	LOC_Os09g33870.1	PRRKSSDIYREESPPKRRSKDRVDS	S	223
3586	LOC_Os09g33940.1	AYSNMVHQHEKSSGSPGEGVNLSVT	S	103			3587	LOC_Os09g33940.1	SNMVHQHEKSSGSPGEGVNLSVTDK	S	105
3589	LOC_Os09g33960.1	GAFSSGGVVRSASNENFCPTSEEKH	S	431			3590	LOC_Os09g33970.1	AAEVEREEEEEASPPRVAARNPFDL	S	38

3592	LOC Os09g33980.1	HPYQESEDDQESSEEQPAQDRRENG	S	165			3593	LOC Os09g33980.1	SIRGQRLHPYQESEDDQESSEEQPA	S	158
3595	LOC Os09g33980.1	ARPKRKKLSNSSSIPRREGLRPRRS	S	134			3596	LOC Os09g33980.1	VSARPKRKKLSNSSSIPRREGLRPR	S	132
3598	LOC Os09g34040.1	GKVLLTRRSEPLSPPEYSPRYENDR	S	53			3599	LOC Os09g34040.1	TRRSEPLSPPEYSPRYENDRDEYEQ	S	58
3601	LOC Os09g34060.1	AHGARPKHQHSLSMDESMSIKAEEL	S	138			3602	LOC Os09g34060.1	SMSIKAEELVGASPGTEGMSSAEAK	S	154
3604	LOC Os09g34070.1	RSRGRAEWLETGSFNGRTGPAKGYG	S	207			3605	LOC Os09g34180.1	EPHAAHTKAVLLSPGNSTALYDGDH	S	190
3607	LOC Os09g35710.1	NTESAQPVYTVDSPPSSSQPQGNLN	S	318			3608	LOC Os09g35760.1	RSLAMMGNGGSGSGGDGDSLGRGRE	S	80
3610	LOC Os09g35760.1	DSRSGSDNLDGASGDELDPDNSNPR	S	108			3611	LOC Os09g35920.1	SASQLPLLQRLNSLVAELDTMQKHA	S	78
3613	LOC Os09g36090.1	HGESAGYTSKSDSPNIGTHAVKVGA	S	1179			3614	LOC Os09g36090.1	PRESESVGTSTSSAEETSWASKELL	S	728
3616	LOC Os09g36090.1	RGSFSKKDNNPVSPGKGEGRSPAQR	S	1122			3617	LOC Os09g36090.1	NNPVSPGKGEGRSPAQRDLKTNWES	S	1130
3619	LOC Os09g36090.1	EPHDTNDEEEGNSDSSSVRHLEGNS	S	645			3620	LOC Os09g36090.1	RNTWGESSTHIESPLGRRPAFSSHG	S	1156
3622	LOC Os09g36090.1	QPSKSAVLSSTQSFSGRAGQGNDAA	S	1318			3623	LOC Os09g36090.1	SSSDVDGRPRNDSLLEEGTRAGEQP	S	1295
3625	LOC Os09g36160.1	RARPSATTPTTSSGDQQMVTVAERF	S	182			3626	LOC Os09g36270.1	ISSKQLHRGGVDSPSWRSGAQPDNI	S	166
3628	LOC Os09g36270.1	AATQAPAAMLPRSGSRPQLDLSGAA	S	50			3629	LOC Os09g36270.1	LPRSGSRPQLDLSGAAIHGNLEDRN	S	59
3631	LOC Os09g36270.1	YWGLGRLMTKCKSFDELLELSQRGD	S	292			3632	LOC Os09g36320.1	KSTGSTSSHRSISSLYEERGHGQLR	S	60
3634	LOC Os09g36440.1	ETSEWRTFANESSDNDPVRVGGPTN	S	52			3635	LOC Os09g36480.1	PRLSFCDEIENGSDEDEFENQEPQK	S	129
3637	LOC Os09g37006.1	KKELDTSSIGYQSPLPADKVKPLVE	S	126			3638	LOC Os09g37040.1	SVSSSYRSELGASSNSLSWTDKSET	S	317
3640	LOC Os09g37270.1	DIEKVWTYAGNLSARKDAGDAPERD	S	549			3641	LOC Os09g37520.1	SDSEGGGHIKFHSDDEGDAPAHRRP	S	144
3643	LOC Os09g37720.1	QARMSRRRQQPGSDSDGEDDSFLYR	S	23			3644	LOC Os09g37720.1	RMSRRRQQPGSDSDGEDDSFLYRYP	S	25
3646	LOC Os09g37860.1	APKKKQELLEPSSDDSELEQQQERL	S	47			3647	LOC Os09g37860.1	KKQELLEPSSDDSELEQQQERLQEV	S	50
3649	LOC Os09g38200.1	ARNLVFRGAGSASSSTSRQKQGEQW	S	404			3650	LOC Os09g38200.1	RNLVFRGAGSASSSTSRQKQGEQWN	S	405
3652	LOC Os09g38200.1	VFRGAGSASSSTSRQKQGEQWNEAV	S	408			3653	LOC Os09g38390.1	NPEHEPYLDDTNSGPLERNVNSDNG	S	322
3655	LOC Os09g38570.1	EGRAAESDVNVSSPRIKEANEDAEE	S	215			3656	LOC Os09g38570.1	ENSGHVEGRAAESDVNVSSPRIKEA	S	209
3658	LOC Os09g38620.1	KPEEAMHINKSFSLSNGHAVYDIQH	S	300			3659	LOC Os09g39020.1	NDPFRKGTQERKSLSPNIPHFPADQ	S	174
3661	LOC Os09g39210.1	MADPASPATGELSPRTPQPFMEYVR	S	858			3662	LOC Os09g39380.1	DGKVLGAFLEGGSPDENKAIAKVAK	S	413
3664	LOC Os09g39870.1	RSLSESFRSRPRSAPSLVELTPKHK	S	690			3665	LOC Os10g02509.1	PPPQQPKEECISSPLIEENVPVIDE	S	174
3667	LOC Os10g02630.1	STRDQQHAGRPRSEEPRRDERRADR	S	259			3668	LOC Os10g03830.1	LMEGFEGLVREGSFSWALPRRGASP	S	23
3670	LOC Os10g04580.1	VDIAPRDGKRVSSGNLVDVARQWVE	S	182			3671	LOC Os10g05690.1	QHGGGGEVEEESSEMGEKTAARTRL	S	41
3673	LOC Os10g07270.1	FSPLLRDAEDGYSFSNGTKDDIHGL	S	298			3674	LOC Os10g07270.1	PLLRDAEDGYSFSNGTKDDIHGLSG	S	300
3676	LOC Os10g08550.1	KISGDSLKNVYKSFVSEYPIVSIED	S	325			3677	LOC Os10g08570.1	GDNHKGEVADQLSNDEDVGGRENDE	S	218
3679	LOC Os10g08570.1	GGFNVHSGATSKSPDKSKMKKDQTD	S	443			3680	LOC Os10g10620.1	VDAALKDCKSTISDAGDLARQSSAE	S	118
3682	LOC Os10g10990.1	QKDQPKDEPVDNSPAKPTPSGHARG	S	389			3683	LOC Os10g14230.1	NNPLVTKEYDGESDDELDADVLQYL	S	576
3685	LOC Os10g19200.1	ARPRRHLPSPSPSGLPRAARATPWC	S	21			3686	LOC Os10g20240.1	DVVETLPLTNSRSCQGFDGLFQRSK	S	294
3688	LOC Os10g20600.1	TRKGANYVERQNSEISYYADDEDAN	S	254			3689	LOC Os10g20600.1	GANYVERQNSEISYYADDEDANRKK	S	257
3691	LOC Os10g21000.1	PIRTLQRDGAVASFSKEKTPPSSPT	S	48			3692	LOC Os10g21000.1	RTLQRDGAVASFSKEKTPPSSPTNR	S	50
3694	LOC Os10g22950.1	EERLQKALARVKSMVQYPEARDQYQ	S	962			3695	LOC Os10g22950.1	HALSLLSMKPSKSGQLDPLHAAASR	S	835
3697	LOC Os10g22950.1	GFPFLKTQSSNLSDILKDSFKKSDS	S	373			3698	LOC Os10g24690.1	DPVLVRDFFRLLSLGSEASVPRDGV	S	33
3700	LOC Os10g25090.1	RNQSFDDDDDDFSNKPVAKKSNSAS	S	387			3701	LOC Os10g25110.1	LADLAGKEKLSASEVRKLLKTEMPG	S	825
3703	LOC Os10g25110.1	SKGRGKSCHSDSSIEMNPGHMKNDN	S	82			3704	LOC Os10g25110.1	QNIEELLTSRSPSKKRKQKDKTDVT	S	694
3706	LOC Os10g25220.1	VDAVLRSRMARMSLSSARKDRKGSR	S	96			3707	LOC Os10g25320.1	TLLEDIPVSTALSRSASSAGDSDGK	S	151
3709	LOC Os10g26540.1	MRKQNKRELSRASLGGDDDLLAAQQ	S	59			3710	LOC Os10g28200.1	EKTQGVDIAGYGSSKVVSTQAPVQL	S	356
3712	LOC Os10g28610.1	RDLGINKLEVSTSSVEPHEQWSNNV	S	1850			3713	LOC Os10g28710.1	IPKFSLRQNRSISLELPLFERSDEV	S	134
3715	LOC Os10g29560.1	MESAIPVQPKVRSPAVSAASKEEPK	S	389			3716	LOC Os10g29560.1	GDYYGEEEEDDESEEEQHFKRRPSS	S	861
3718	LOC Os10g30100.1	DMEMSRSPPEHSSAGAKQRFQSLIE	S	796			3719	LOC Os10g31520.1	DTATPYESAKSTSNNDEIADMAKSL	S	343
3721	LOC Os10g31770.1	TRENDLGISNQGSPDHVFSRRHWRL	S	199			3722	LOC Os10g31770.1	LYDWTRENDLGISNQGSPDHVFSRR	S	195
3724	LOC Os10g32710.1	AEKDPKFIAPAYSQTQPKPVFAESD	S	627			3725	LOC Os10g32850.1	AVAGEKGSEGDGSSDELQIVAEKGP	S	109
3727	LOC Os10g32960.1	LVVLPYFKSKLQSIYNKEREARLQA	S	133			3728	LOC Os10g33230.1	KAANPSAGGRHASGGGGGGGGAGGG	S	102
3730	LOC Os10g33680.1	RRGSGAGPSSLGSPLKVVSEPVREV	S	119			3731	LOC Os10g33680.1	PRVMRRGSGAGPSSLGSPLKVVSEP	S	115
3733	LOC Os10g34380.1	TDAAPMGHQVSISGEKSKDNSGDGN	S	661			3734	LOC Os10g34400.1	LREKLGMDSINISSSSFGAKHEDHN	S	341
3736	LOC Os10g34750.1	GGAVAKPEEKPHSPVKPKRSSSPTK	S	67			3737	LOC Os10g34820.1	SIDISGTSPKDRSNDLLEKHNNLLN	S	115
3739	LOC Os10g34820.1	NTQLDLETEGSSSPAATQKMKVKME	S	18			3740	LOC Os10g35150.1	RSFGEDNGMDYWSDDEDNEKMSRSW	S	124
3742	LOC Os10g35220.1	TRLPNRELPSSISPGADYMSRRSYL	S	597			3743	LOC Os10g35280.1	EFDDDDFDDNEGSAEKHSSVPKEEP	S	78
3745	LOC Os10g35280.1	TEEDIVEDLILSSDEEDEDEDKNME	S	681			3746	LOC Os10g35450.1	GKMLAHLVRSKSSDADNLSQCSSVY	S	116
3748	LOC Os10g35450.1	FDKIPVSQIPDVSKEIAVDEVREHA	S	67			3749	LOC Os10g35580.1	ERPAKQEAHKRKSISDLELEEFGMA	S	296
3751	LOC Os10g35690.1	PFIYGRSVEVNLSEEEYGYDFVDRR	S	238			3752	LOC Os10g35870.1	KTVPVEDGAPSTSPETTETAAAAEP	S	183
3754	LOC Os10g36350.1	LVPMQRILDNGRSGSELSSGNVSLR	S	159			3755	LOC Os10g36350.1	ILDNGRSGSELSSGNVSLRSAEMGT	S	165
3757	LOC Os10g36350.1	RILDNGRSGSELSSGNVSLRSAEMG	S	164			3758	LOC Os10g36350.1	SLRSAEMGTELGSPLGRFVRNEDML	S	181
3760	LOC Os10g37480.1	MGRNGSVKRTSSSGAAAAFTANPRD	S	13			3761	LOC Os10g37480.1	LGKSSSTIENFFSGRTSTTAANSDG	S	403
3763	LOC Os10g37480.1	EALSMSEYQRGVSAWNFDVEDLKAQ	S	345			3764	LOC Os10g37480.1	DDTARTIEVEKDSFSRNHLGKSSST	S	385
3766	LOC Os10g37480.1	KLRTETRRATNLSGPLSLPTRASAN	S	519			3767	LOC Os10g37630.1	AWVAGGVLSRSLSILGLAQGGGGGG	S	126
3769	LOC Os10g37640.1	EEEDMDSEGEDESILSEELIQKVMS	S	116			3770	LOC Os10g38850.1	VLSDNLVRLECISEIDSRNDKAKNP	S	1611
3772	LOC Os10g38850.1	NLVRLECISEIDSRNDKAKNPSVDH	S	1615			3773	LOC Os10g38850.1	KPKASDDVLCHRSLGETIEQDAETR	S	266
3775	LOC Os10g39200.1	SELPEVNNPTVDSPCWKGAPIAYPP	S	349			3776	LOC Os10g39200.1	HSEGKDSVSGLDSPGDATCSDEDNG	S	1011
3778	LOC Os10g39440.1	DAESQREGEDYGSDHGGDDIEDSLQ	S	357			3779	LOC Os10g39440.1	YGSDHGGDDIEDSLQSPLISRQATS	S	367
3781	LOC Os10g39440.1	EQQQAKGDWDAESQREGEDYGSDHG	S	348			3782	LOC Os10g39520.1	STPTPTPPERHDSFSFPRLPAHNLQ	S	494
3784	LOC Os10g39820.1	NCGKSGHIARECSAPPMLPGEMRLC	S	112			3785	LOC Os10g40110.1	SVVVEELNGSSSSDDESKATSAPPA	S	232
3787	LOC Os10g40590.1	DEDEFHFYDTRQSFSDTAASPDLKM	S	303			3788	LOC Os10g40590.1	YDTRQSFSDTAASPDLKMRCSNSGS	S	310
3790	LOC Os10g40780.1	GEPTTTNKDLVTSAPLSPTTAVDRM	S	1616			3791	LOC Os10g40780.1	TTNKDLVTSAPLSPTTAVDRMGSVF	S	1620
3793	LOC Os10g41030.1	SSPHLVIPLPTISGEFIQSPKGKQL	S	434			3794	LOC Os10g41030.1	IPLPTISGEFIQSPKGKQLAHVAGV	S	440
3796	LOC Os10g41030.1	PQKSRKRKKAPASPEQPIIAPLLKT	S	1198			3797	LOC Os10g41030.1	AKHDHAIQNLEPSSSGKGLQLDVVH	S	1512
3799	LOC Os10g41030.1	HDHAIQNLEPSSSGKGLQLDVVHSG	S	1514			3800	LOC Os10g41030.1	NLASFSSLTPTSSWKTKDDISKGSI	S	1390
3802	LOC Os10g41030.1	GEMRSRVPKSTKSQDGATNIIPGKG	S	1871			3803	LOC Os10g41030.1	LSHGGDRTLVTVSSENKNGLEHGTG	S	733
3805	LOC Os10g41030.1	SKHYDADQADKISEGNASTRPVKHL	S	1825			3806	LOC Os10g41120.1	SEQSLKSLGRIESWMSTLGSSAGRN	S	204
3808	LOC Os10g41400.1	WLGKLGLGGLGGSPRASAASAALAQ	S	73			3809	LOC Os10g41440.1	SGAVVTIQESLGSPDDITVEMKGTS	S	372
3811	LOC Os10g41790.1	ENDHKGSTEDLGSDTDELGDEEIGN	S	708			3812	LOC Os10g41960.1	RPYRMELRDGEASDDEEEYDTKEVE	S	689
3814	LOC Os10g42196.1	TKQHTGATTRSVSDEILANTQARVS	S	1348			3815	LOC Os10g42320.1	EYEEFLRDLEHNSDIRFQIDLYKDE	S	463

3817	LOC Os10g42420.1	SSEARQKFSNAKSISSSQFFGDQAS	S	313			3818	LOC Os10g42420.1	EARQKFSNAKSISSSQFFGDQASFE	S	315
3820	LOC Os10g42439.1	DIVPGSSVTEDASDSETLPRISWNY	S	1013			3821	LOC Os10g42439.1	APPPQPALVRLPSTAPALPSAPANP	S	2611
3823	LOC Os10g42724.1	VSRKMSAGVSNMSFKMKEFFQGQNM	S	35			3824	LOC Os10g42724.1	RTKDGTVGKRAGSGADELLQDLDDM	S	351
3826	LOC Os10g43040.1	KRKSDGIAAALRSPELKKAKVSLGH	S	239			3827	LOC Os11g01190.1	GAVGAVRIDNIGSFDKAVSVNKDES	S	735
3829	LOC Os11g01200.1	IAAPAKKLGSFFSEVATESAHRFAL	S	579			3830	LOC Os11g01300.1	RRASTAGGSGGFSGGGGSNMLRFYT	S	51
3832	LOC Os11g01300.1	GMRRRRASTAGGSGGFSGGGGSNML	S	47			3833	LOC Os11g01836.1	HEFRVGDKSHPLSDQIYKKLDELGL	S	535
3835	LOC Os11g01880.1	EICHLYDAVQVDSDEEKHPAEPRIT	S	172			3836	LOC Os11g02620.1	VSGLAHLECDVGSGKLRMHCQDWWV	S	198
3838	LOC Os11g03810.1	IVEKMVVLGGNNSFKFAEQAWHRSE	S	87			3839	LOC Os11g03890.1	IESNPYFAVTAGSPLDVSKRARMME	S	39
3841	LOC Os11g04190.1	DEEKGAAGGGGHSPQPVVGKRKDLH	S	293			3842	LOC Os11g04190.1	KRKDLHMFVWSSSASPVSERAAAAA	S	312
3844	LOC Os11g04190.1	HMFVWSSSASPVSERAAAAAAGAVH	S	317			3845	LOC Os11g04190.1	VGKRKDLHMFVWSSSASPVSERAAA	S	310
3847	LOC Os11g04190.1	KDGPTLSKLGSNSTAQLRPKDDGEG	S	370			3848	LOC Os11g04190.1	LQSSRNPTPRGSSFNHAEFFNIVGN	S	264
3850	LOC Os11g04190.1	HSQSMQPRVSNLSGVEIYSLQSSRN	S	245			3851	LOC Os11g04190.1	GDAKGAQAYDEYSFGNKNEKDGPTL	S	351
3853	LOC Os11g04670.1	DIIQIPTVLREYSMTSVTEGIDAIA	S	550			3854	LOC Os11g04890.1	GSAGANGRLPKPSSGDDELVLTPAP	S	147
3856	LOC Os11g06170.1	GAPPRGHHRRAHSETFIRLPDADLL	S	25			3857	LOC Os11g06170.1	GHKRSGSMDGESSLFEGESAPPDYA	S	132
3859	LOC Os11g06410.1	HHHHRHHSKNHGSDEEEHDMNEDAA	S	136			3860	LOC Os11g06440.1	SSSSSSSHFRSLSWSVSRNWSAARQ	S	235
3862	LOC Os11g06440.1	SSSSSHFRSLSWSVSRNWSAARQLQ	S	237			3863	LOC Os11g06650.1	GGNCGAAAATTPSPRGAAVAEGEDD	S	76
3865	LOC Os11g06680.1	NDKAVRHHPPTCSDDEFSDDENHEV	S	812			3866	LOC Os11g06680.1	RHHPPTCSDDEFSDDENHEVVQEAG	S	817
3868	LOC Os11g07040.1	NAGGAGRRTMRNSLQLNLGEILNPS	S	499			3869	LOC Os11g07280.1	IDFYHLKSRRGMSQLELEDAVQDDL	S	689
3871	LOC Os11g07440.1	LTLERKRSFDEQSWSELSHRQNDGF	S	42			3872	LOC Os11g07500.1	MSFRDKQRVIYTSPLKALSNQKYRE	S	133
3874	LOC Os11g08440.1	HDGIDFCGKMSRSRFEELNKELFGK	S	330			3875	LOC Os11g08550.1	ETINKEKSSIMFSRNIKEVNKQIFM	S	1158
3877	LOC Os11g09160.1	KSPNPPFLMRFLSGEVSRRGRCVSK	S	250			3878	LOC Os11g09160.1	AHPSKNVIIKTESDEGGMVTAAITT	S	145
3880	LOC Os11g10060.1	GNQLLNGLRAANSFNNLQYDWKPSQ	S	1065			3881	LOC Os11g10060.1	NQLQQQQLLRQQSLNMRTPGKSAPY	S	316
3883	LOC Os11g10710.1	ELPIDHALEKLPSYNNESINLESKL	S	320			3884	LOC Os11g10710.1	NNESINLESKLASHLNLNETRENQD	S	334
3886	LOC Os11g12810.1	QELGSREAAADLSELSEGEKDGKPD	S	136			3887	LOC Os11g12810.1	GSREAAADLSELSEGEKDGKPDTHP	S	139
3889	LOC Os11g16400.1	DVRRMSSSLNMSSSSLRSSGGADVD	S	35			3890	LOC Os11g16400.1	TTTVAAMDVRRMSSSLNMSSSSLRS	S	28
3892	LOC Os11g16400.1	TVAAMDVRRMSSSLNMSSSSLRSSG	S	30			3893	LOC Os11g16470.1	GALVSASTGVMESLLGKLSSMLEKE	S	15
3895	LOC Os11g19130.1	KKQPMKRRFNDLSEDEEDPLAMIRS	S	476			3896	LOC Os11g19810.1	GKATNTGSCASFSDVDEDIPEKAAG	S	848
3898	LOC Os11g19810.1	IYGKATNTGSCASFSDVDEDIPEKA	S	846			3899	LOC Os11g20040.1	DGVVSYVAGDMLSFIPPAQAVMLKV	S	259
3901	LOC Os11g22180.1	CRKATNSTGSDASPPPQRRQRLVMI	S	113			3902	LOC Os11g26130.1	NVEKFRPVSTKTSPAESEMMKIQVV	S	517
3904	LOC Os11g28270.1	GAVTNWPLSRSASFIASPRWPGHSS	S	215			3905	LOC Os11g28270.1	NWPLSRSASFIASPRWPGHSSYAQV	S	219
3907	LOC Os11g28270.1	VPAHSEVSPDNVSGRSRRITHSDSQ	S	418			3908	LOC Os11g28270.1	SARRMLAPVPAHSEVSPDNVSGRSR	S	410
3910	LOC Os11g30430.1	GRAGGGVPVGRSSPVWQIRRPPSPA	S	217			3911	LOC Os11g31880.1	PPWDLLYRLNRHSLQRKKSGKSRLQ	S	305
3913	LOC Os11g31890.1	GQKSNFASDDLPSPTPSGDGDKSGD	S	457			3914	LOC Os11g31890.1	NFASDDLPSPTPSGDGDKSGDKGGD	S	461
3916	LOC Os11g32900.1	TKTIKNMLAAPSSPSSPANGGSIKI	S	369			3917	LOC Os11g32900.1	IKNMLAAPSSPSSPANGGSIKIVQM	S	372
3919	LOC Os11g32900.1	FGCSKRVFETLASPTKTIKNMLAAP	S	355			3920	LOC Os11g32900.1	SPLRQTKMDALLSPSSRSFYACIGE	S	895
3922	LOC Os11g32970.1	MRASGQNPIKAFSDISILRETTADE	S	165			3923	LOC Os11g33120.1	TLLARTLEKRSSSFGHSVIRNASSR	S	127
3925	LOC Os11g34190.1	KKFIKQCVDKVFSGSDDDNTNNNAP	S	72			3926	LOC Os11g34190.1	FIKQCVDKVFSGSDDDNTNNNAPEK	S	74
3928	LOC Os11g34190.1	SEINDSHCERGDSDEDARPKKKKAE	S	248			3929	LOC Os11g34200.1	VVNKVPDLGGADSCGDDRALPRRRK	S	95
3931	LOC Os11g34450.1	MQLLRDNLTLWTSDANDDGGDEIKE	S	243			3932	LOC Os11g34660.1	SYIRRKRLARKTSIPGSSSKWANHS	S	192
3934	LOC Os11g35050.1	SGKSPSSPVDVFSSDDEHRHDTESS	S	131			3935	LOC Os11g35050.1	GKSPSSPVDVFSSDDEHRHDTESSA	S	132
3937	LOC Os11g35590.1	GRTRVAGSGTLLSLCLRAVASTLGV	S	1321			3938	LOC Os11g36060.1	DSFYEDLVDGKGSDGKPKSIPDKPL	S	79
3940	LOC Os11g36340.1	KVGKKPALSQSASFPARGAAAKKAA	S	88			3941	LOC Os11g36340.1	AAASVDGSVSCESPRSITNLAKLTE	S	314
3943	LOC Os11g37090.1	VARTASLSTGRASPSQFAPTVGRYH	S	53			3944	LOC Os11g37100.1	SPNGATSRDKTPSPQASERPISIDD	S	447
3946	LOC Os11g37990.1	QDEEQARLNAASSFSSRTEAPVEPT	S	500			3947	LOC Os11g37990.1	DVELYAKLDSAYSSDEEDVEDEDDE	S	754
3949	LOC Os11g38620.1	SKHQRAVLPRLRSVLDQELDRSAFE	S	436			3950	LOC Os11g38870.1	SVSTERERSRAGSGSTPATSSGLTD	S	237
3952	LOC Os11g38870.1	SLPGQLRLSLPQSSQEENSSGSKKG	S	262			3953	LOC Os11g39030.1	VAAPAMKPLAPRSPAAAAAAVVPSP	S	368
3955	LOC Os11g40080.1	RVTILERMFGRKSIKDGPDGVDRVS	S	170			3956	LOC Os11g40080.1	DLERSDSFQLSRSLQSNGENNGVEP	S	932
3958	LOC Os11g40080.1	WRIWPFSFKRTRSVNTVQPVSESTE	S	1097			3959	LOC Os11g40080.1	LRSHIHDLERSDSFQLSRSLQSNGE	S	926
3961	LOC Os11g40090.1	TVDDSLAAAASPSPKKKEKHSKKKR	S	39			3962	LOC Os11g40090.1	KREAIDATMAAASPKKKEKKHSKKQ	S	62
3964	LOC Os11g40140.1	TRTEYDRGVNTFSPEGRLFQVEYAI	S	16			3965	LOC Os11g40510.1	DSKGMIELTNGSSEGGKDNANEDGP	S	266
3967	LOC Os11g41890.1	MVEVKRAVPKELSPGPSMRSPVGGF	S	187			3968	LOC Os11g41890.1	KRAVPKELSPGPSMRSPVGGFNYAV	S	191
3970	LOC Os11g41890.1	GSIYGDTTWRSGSSELDGTSPFGYG	S	436			3971	LOC Os11g41890.1	SIYGDTTWRSGSSELDGTSPFGYGL	S	437
3973	LOC Os11g41890.1	YGRQLNPYYSGNSGRYNSNVSYGGV	S	288			3974	LOC Os11g41890.1	SLQYGRQLNPYYSGNSGRYNSNVSY	S	285
3976	LOC Os11g41890.1	HALSKSGGSAHGSPGPSRTKKIFVG	S	99			3977	LOC Os11g42040.1	CRMPIVVHELAVSISQKEEFGAICH	S	551
3979	LOC Os11g42420.1	AADISDFDSLDESKLFGSGKKRASL	S	92			3980	LOC Os11g42420.1	LLEDIKQEAADISDFDSLDESKLFG	S	84
3982	LOC Os11g42420.1	FGSGKKRASLDASDAGFSSGRQAVR	S	107			3983	LOC Os11g42790.1	FGGRGFVPFVPGSPTERSVPLLQGN	S	531
3985	LOC Os11g42930.1	LDSADRATVRASSMDSDLPDSATAK	S	166			3986	LOC Os11g42930.1	ATAKLPDVEEALSPRSERKRKIHLY	S	187
3988	LOC Os11g43590.1	SAPDVEPLLYEDSPPSSKELSDHLN	S	418			3989	LOC Os11g43590.1	SGRADDLAWRCSSDTFDLNGRAFEN	S	69
3991	LOC Os11g43610.1	AALREVFGDSSDSEADDLPVGGAGR	S	27			3992	LOC Os11g43970.1	PPKRPKAEAAPASPTASVPGRIEED	S	39
3994	LOC Os11g43970.1	GSEKLAILHKNRSSLADAMHFRRPA	S	505			3995	LOC Os11g43970.1	DQLKDLSAAPPASAGENNQRLVRPM	S	265
3997	LOC Os11g44940.1	RRHKIEKDLGIGSEVGFSKNAEVAR	S	156			3998	LOC Os11g46240.1	GREGPVGFGGQESPGGFGGRKGPGA	S	1080
4000	LOC Os11g47770.1	KRKSPAATAAVGSPRKTRSMAVGKQ	S	16			4001	LOC Os11g47830.1	SGQAKEHDEKRRSYTPEYNDRRDAD	S	358
4003	LOC Os11g47830.1	ERSSVLGRSPRPSPPGRSHCHSHSR	S	294			4004	LOC Os11g47830.1	APDGERSSVLGRSPRPSPPGRSHCH	S	290
4006	LOC Os11g47830.1	ERSWALGRSPQPSPPGRSHFHSHSR	S	395			4007	LOC Os11g47830.1	PQPSPPGRSHFHSHSRSRSPELRGR	S	404
4009	LOC Os11g47830.1	GRSHFHSHSRSRSPELRGRARSRSC	S	410			4010	LOC Os12g01940.1	EICHLYDAVQVDSDEEKHPTEPRIT	S	178
4012	LOC Os12g02690.1	TKAPELPSRRQLSFDSGDYPVKRLS	S	129			4013	LOC Os12g04440.1	DVSDSKHALTGHSFNRAFSGSGAAL	S	583
4015	LOC Os12g05110.1	AMTKIVGTLGPKSRSVDTISSCLKA	S	40			4016	LOC Os12g05110.1	TKIVGTLGPKSRSVDTISSCLKAGM	S	42
4018	LOC Os12g05420.1	PTYQRTLSGGLKSPRAADVPREAIL	S	376			4019	LOC Os12g06380.1	NESAEDIQCTQTSPESRVKHRSILE	S	513
4021	LOC Os12g06520.1	GSGGSGGHKRSGSMDGVNSPFEGES	S	124			4022	LOC Os12g06520.1	GHKRSGSMDGVNSPFEGESALSGGL	S	130
4024	LOC Os12g06560.1	RPQHKQYSFVSDSEEDTEKGKLVES	S	963			4025	LOC Os12g06560.1	VDKEMCYRPPTLSDDDFSEDENHAD	S	879
4027	LOC Os12g06560.1	AIHNDKPPPLPVSTSFSEKNAKTEN	S	366			4028	LOC Os12g06560.1	HNDKPPPLPVSTSFSEKNAKTENSS	S	368
4030	LOC Os12g06640.1	EGNNLPSGSLMRSNSGQMHAPNPGK	S	14			4031	LOC Os12g06640.1	NNLPSGSLMRSNSGQMHAPNPGKQG	S	16
4033	LOC Os12g06640.1	DEEHGVNEDATDSQSGKGKKGAAWQ	S	93			4034	LOC Os12g06640.1	GNSSSSHGCSRRSDSHGISLDINKA	S	328
4036	LOC Os12g06670.1	FLRIEEVDPTYDSDASEDGADDGDD	S	134			4037	LOC Os12g06670.1	IEEVDPTYDSDASEDGADDGDDLSV	S	137
4039	LOC Os12g06670.1	LEASILRNEGSWSGSSHSSAAERTL	S	232			4040	LOC Os12g06810.1	FQKGSSFLAGNLSFSRARASLEGSA	S	386

4042	LOC_Os12g06810.1	LAGNLSFSRARASLEGSAPVPIHKE	S	393			4043	LOC_Os12g06850.1	GDGGSSVLTDGDSPDKIQGRSYQNA	S	196
4045	LOC_Os12g06850.1	QNAQVSDFKYSPSPQRHNGLSSERS	S	218			4046	LOC_Os12g06870.1	FNKTAISPTRSTSIENGIHRDDRAS	S	767
4048	LOC_Os12g06890.1	KGPLAISPTRSTSIENGIQRDDRAS	S	745			4049	LOC_Os12g06980.1	EVIEPLKRSRRKSVVSTLLEEGTKC	S	523
4051	LOC_Os12g06980.1	SLPVPEDISAPKSPEERAIHQDDSV	S	2334			4052	LOC_Os12g07190.1	ANGNGKASSKPTSPGQLTGERTVRK	S	46
4054	LOC_Os12g07190.1	MQRFWDSTLALESPDEECDSRSEVS	S	375			4055	LOC_Os12g07260.1	AHKKDLDLTSDESSDEDTIDAIGLS	S	115
4057	LOC_Os12g07260.1	PPEAHKKDLDLTSDESSDEDTIDAI	S	112			4058	LOC_Os12g07260.1	SGDKQDELEEGFSDLEVPPEAHKKD	S	95
4060	LOC_Os12g07530.1	YRDRLDDPYVERSREREIDRLYAGR	S	318			4061	LOC_Os12g07530.1	FDDTHEFSDQEVSPVVSRGRPRKAM	S	575
4063	LOC_Os12g07530.1	EYYDDDVDSYDLSPQRMAEPYDMVD	S	618			4064	LOC_Os12g07530.1	PLGREYYDDDVDSYDLSPQRMAEPY	S	614
4066	LOC_Os12g07530.1	RDHVTEHYQTDDSPLGREYYDDDVD	S	601			4067	LOC_Os12g08800.1	EPDDHTERIGYYSDPEKQLDAERIP	S	532
4069	LOC_Os12g08800.1	LDAERIPELSRPSPEECEISDMPEL	S	550			4070	LOC_Os12g09000.1	RELLGPGKIIGVSCKTPAQAQQAWN	S	443
4072	LOC_Os12g09880.1	FSSSSGRLSAAGSTASGGSGGGWER	S	25			4073	LOC_Os12g10070.1	LGLWKNTFKSWPSAEKSCPAWYMRV	S	76
4075	LOC_Os12g10700.1	LKMAGLLDSPPNSPERKNTIIEGGC	S	905			4076	LOC_Os12g10700.1	IGDTPEFLRSDKSVPDIMSIVEGED	S	406
4078	LOC_Os12g11600.1	RDAEGNEEEDSGSHPSAGQKRARGQ	S	63			4079	LOC_Os12g12370.1	PPPEVALLARNFSVAVDTVARSAML	S	110
4081	LOC_Os12g13340.1	KSGSAILRKSNTSDDELDELSSPLT	S	858			4082	LOC_Os12g13670.1	SHSRGGSPSPPISGRDMGSRGRRQS	S	294
4084	LOC_Os12g14110.1	ELKSKQKLEKKLSFYTKVKDAVTSL	S	31			4085	LOC_Os12g14920.1	DDSRYSSGRGLASISTRREYSLSPW	S	222
4087	LOC_Os12g15420.1	KSRASPPRRPRDSDPAESNGGGAPK	S	78			4088	LOC_Os12g15420.1	APKPGGDNGGDRSPRRARVPDAEEE	S	100
4090	LOC_Os12g17310.1	PAPEAQAHGTASSPVQKASAGKANE	S	588			4091	LOC_Os12g17310.1	VGDLHVKLQKCKSELEAAVAAESKA	S	372
4093	LOC_Os12g18860.1	RKSVDCFEPSRRSVDFFEPSRKSTD	S	294			4094	LOC_Os12g18860.1	RESLDRIEPSRKSVDCFEPSRRSVD	S	284
4096	LOC_Os12g19040.1	IRFESAGGESDKSPVKEIQTTFQWS	S	239			4097	LOC_Os12g19040.1	PVVVRPGHIRFESAGGESDKSPVKE	S	231
4099	LOC_Os12g19040.1	LPFVRHEADEEESDTSDDIVPVVVR	S	211			4100	LOC_Os12g19040.1	VRHEADEEESDTSDDIVPVVVRPGH	S	214
4102	LOC_Os12g19040.1	SSNNRDVLPSPSSNQNNSSLPFVRH	S	192			4103	LOC_Os12g19040.1	PSSNNRDVLPSPSSNQNNSSLPFVR	S	191
4105	LOC_Os12g21700.1	HPMGTLMYGSATSPRGDVSSMHYQL	S	401			4106	LOC_Os12g21710.1	RGSSIGGAGEDDSDGEVDGAPRLVG	S	68
4108	LOC_Os12g21870.1	AGREAQSSIFSTSGLSSWAKNLKIP	S	771			4109	LOC_Os12g21890.1	SPTGKNTFSRLTSGLGLRLSPKGPQ	S	114
4111	LOC_Os12g21890.1	KESARNDKDDVFSDSEAEDGSSKGR	S	543			4112	LOC_Os12g21890.1	SARNDKDDVFSDSEAEDGSSKGRRE	S	545
4114	LOC_Os12g21890.1	NLKIPQPSSGQESPTGKNTFSRLTS	S	102			4115	LOC_Os12g22090.1	AKAIHQKKRKANSDPGESPLARHKP	S	449
4117	LOC_Os12g24080.1	RSKIKHQHDHHHSPVRISVRRAYIL	S	636			4118	LOC_Os12g24080.1	LRPGQPDAIQEASTSDMEDASTSSG	S	548
4120	LOC_Os12g24080.1	HRYHSGSLFGMNSRGRRGESSRRGD	S	59			4121	LOC_Os12g24080.1	NMLRERFAHRYHSGSLFGMNSRGRR	S	51
4123	LOC_Os12g25700.1	FDWDHPRHLQPTSPTAFKQVSVVWD	S	393			4124	LOC_Os12g27090.1	AKDGDDILRRSDSYDVMDLEELLKQ	S	152
4126	LOC_Os12g31160.1	VSASMGVMVPLLSKLIESMEQPRFK	S	17			4127	LOC_Os12g32986.1	WSANMERLMRAQSMGDMSSLDFMRS	S	691
4129	LOC_Os12g33090.1	DDEELGRLKRCSSFELRSGAANGNH	S	541			4130	LOC_Os12g34330.1	DEIFLEKRNETKSLGSLGDQIADVT	S	322
4132	LOC_Os12g34330.1	RAAAALPSLEDVSPSHPNSDDEKLG	S	238			4133	LOC_Os12g34330.1	AAALPSLEDVSPSHPNSDDEKLGAY	S	240
4135	LOC_Os12g34370.1	FRSVPSTAFVEDSDSEQDNTKNGAN	S	519			4136	LOC_Os12g34370.1	SVPSTAFVEDSDSEQDNTKNGANLD	S	521
4138	LOC_Os12g34920.1	EEEEESKRPRDGSEEEEELLTDLSR	S	32			4139	LOC_Os12g35030.1	ITDETRDDEFDESPSRLDPLKFDDV	S	130
4141	LOC_Os12g35030.1	RNRFKDLIPQSGSPPRRRAGSPPSD	S	95			4142	LOC_Os12g35290.1	VSQLLTVPARSNSSGISIERRQSGS	S	530
4144	LOC_Os12g35620.1	LKLDDTPKNHDESSDANDEKNETIA	S	169			4145	LOC_Os12g35620.1	KLDDTPKNHDESSDANDEKNETIAE	S	170
4147	LOC_Os12g36180.1	KDRNGSTKDRDSSPVQNFSRKNTTQ	S	302			4148	LOC_Os12g36180.1	RSNQNHNHHRSSSNQAGSSSIDELE	S	426
4150	LOC_Os12g37060.1	MMKKRHKIERDLSIGEEVGWSKNVQ	S	156			4151	LOC_Os12g37430.1	EHQRREIHLNAPSPDRAARRAAMEG	S	90
4153	LOC_Os12g37570.1	EGGARAWARGSASFPHSSPLQREGR	S	82			4154	LOC_Os12g37570.1	PPHQPPLPARRLSGGFEGGARAWAR	S	66
4156	LOC_Os12g37720.1	SNAELDGELDSDSETGKEKHDDESS	S	751			4157	LOC_Os12g37720.1	SETGKEKHDDESSLDNINVKKIDNP	S	763
4159	LOC_Os12g37780.1	GRDSASAAAAVASPVKAKARALLEL	S	33			4160	LOC_Os12g37840.1	AQDIDVALARTQSAEILDDIVTRSR	S	627
4162	LOC_Os12g37870.1	QFPLPFGRVLSPSESFIHELDEKTS	S	246			4163	LOC_Os12g37870.1	PLPFGRVLSPSESFIHELDEKTSSS	S	248
4165	LOC_Os12g37890.1	AVARVREEGEVSSGADDDEPLRARF	S	71			4166	LOC_Os12g38000.1	HTHHRKGPARFRSLDFGERNGYLKG	S	31
4168	LOC_Os12g38430.1	SPAPRRRDDYSASPPRKDTHPTKSP	S	177			4169	LOC_Os12g38440.1	ESEARNIQAEQQSGIVVELMQPRGV	S	109
4171	LOC_Os12g38970.1	DQGESDGEKVQSSPGELSDRVMQND	S	130			4172	LOC_Os12g38970.1	DDVVADEDMRYESDENRELKPKEKP	S	263
4174	LOC_Os12g38970.1	RDQEVVRDVFGESDEDEPAPYRDQQ	S	215			4175	LOC_Os12g38970.1	HEDEEPRQTRKASSSPVEEERDQEV	S	195
4177	LOC_Os12g38970.1	DEEPRQTRKASSSPVEEERDQEVVR	S	197			4178	LOC_Os12g38970.1	RDQQEIDEDSHRSPMEDEGHYEKDL	S	236
4180	LOC_Os12g39160.1	GEKSKVGLLQCHSAKVAALEKADDN	S	447			4181	LOC_Os12g39370.1	TLDSLLDENGQDSESDEHGLVKPRY	S	105
4183	LOC_Os12g39980.1	AVLKRQRVPRSLSFTSDIFERSGVD	S	613			4184	LOC_Os12g40010.1	EANIIADRLHRASDAAEASGYAFRD	S	692
4186	LOC_Os12g40010.1	SWFAGRKSNKRMSPNLTSRISRSPL	S	551			4187	LOC_Os12g40520.1	EKVDIKEIVIESSSPPDDRILDDLS	S	503
4189	LOC_Os12g40520.1	QNAHRQTDVMSPSKLRMTESSVSPI	S	687			4190	LOC_Os12g40570.1	QKLQAEMFKRSNSGISLKFDSPSAT	S	191
4192	LOC_Os12g41180.1	KEYTTTQSVKGLSVDSSEAGDEVTS	S	346			4193	LOC_Os12g41180.1	TTTQSVKGLSVDSSEAGDEVTSKAI	S	349
4195	LOC_Os12g41200.1	SEASAERGRRSCSSEVSQLTVVKDL	S	1372			4196	LOC_Os12g41920.1	LIKVNRKYRIAPSSSLSEGRSSKVV	S	187
4198	LOC_Os12g42140.1	DDALYGVFAEGDSDYDSDDGRRRSR	S	54			4199	LOC_Os12g42140.1	YGVFAEGDSDYDSDDGRRRSRRKRR	S	58
4201	LOC_Os12g42370.1	VDPAQPDMRHVLSDLESFDLATEGS	S	528			4202	LOC_Os12g42550.1	SQPPSAAAPAKESSPVNNGQLPAGA	S	285
4204	LOC_Os12g42600.1	KDELALTMSRSMSNGSIMGMDLDSS	S	248			4205	LOC_Os12g42840.1	SGSTEILYRKPESVADVENKVNDAE	S	171
4207	LOC_Os12g44130.1	IPIRQARAEDIRSPTKPENGARGMA	S	424			4208	LOC_Os12g44140.1	HKLKDGISSITSSETDNDDVNKSIK	S	260
4210	LOC_Os12g44330.1	SADMFCDDIFGESPAGIRKLGKDDG	S	566			4211	LOC_Os12g44330.1	PSPSSSCAKHHRSPDPADPAASPKR	S	19
4213	LOC_Os12g44330.1	TGVSGLGEGTPKSERSADMFCDDIF	S	551			4214	LOC_Os12g44330.1	ETKSNHTAHEPESGGDADDTKGDRQ	S	127
4216	LOC_Os12g44360.1	RPEIEADLQRSASLISQTLELPRTQ	S	963			4217	LOC_Osm1g00310.1	HEPMQTGLKAVDSLVPIGRGRRELI	S	284
4219	LOC_Osm1g00440.1	RLQRKIDLKRSTSSIGIVERIEYDP	S	52			4220	LOC_Osm1g00500.1	KKRIANDRFTIDSFNPKRRDIVIIA	S	154
3	LOC_Os01g01150.1	DDLHRPSSPAQASRDAYSDADDDDD	S	62			4222	LOC_Os01g01150.1	SKSRKDDCLLDDTLSSDRRKVQSED	T	873
6	LOC_Os01g01150.1	LDVSSVAHKRSSSASEDEMLNSNSK	S	731			4225	LOC_Os01g04190.1	RKSPAIAAPPPATVRMPGRVLCCGM	T	44
9	LOC_Os01g01150.1	SRSPKRHREESLSPSKSRRSARAGS	S	631			4228	LOC_Os01g06290.1	RSPADHERRDMSTAANGRSPSPRDY	T	296
12	LOC_Os01g01150.1	SPIKYRRSRRSRSYSPPVRHTRGRR	S	543			4231	LOC_Os01g12480.1	MYRKPAAEKSDDTSDDDEEPDIDIG	T	67
15	LOC_Os01g01510.1	ERAVKQIAGGDDSMDEHEDDHKDKN	S	112			4234	LOC_Os01g13160.1	GPYKSPNLRRSLTTEMDKYGRYDPS	T	214
18	LOC_Os01g01510.1	RPPTVKPPVMAASASDDDEIDAFHK	S	24			4237	LOC_Os01g16100.1	MAAPQSHSHPAKTLRASPPPPSTAG	T	46
21	LOC_Os01g01800.1	DIVTQTPYPHIQSPMSSPVQVRGDW	S	397			4240	LOC_Os01g16240.1	EFLNLMARKMKDTDSEEELKEAFRV	T	80
24	LOC_Os01g01880.1	SSLKRKPTVICLSIERILVFFLFSR	S	139			4243	LOC_Os01g16870.1	EMSETSSSHGGHTSAGSAPVPELPR	T	880
27	LOC_Os01g02700.1	RRSAALQAMAAFSVLALVVPDQVQG	S	18			4246	LOC_Os01g19894.1	KREFRVKVSNRTTYDVKCIQGGCPW	T	849
30	LOC_Os01g03520.1	DGDNVSNGVDVDSQSSLPDNKVRVL	S	102			4249	LOC_Os01g27730.1	PHEKLLSLYRVPTFTSVDDFLQKVA	T	522
33	LOC_Os01g03650.1	AARASSAAVPAVSDDLVLRIAEQLE	S	51			4252	LOC_Os01g32660.1	ETPVDKFLTASGTFKDGELRLNQRG	T	30
36	LOC_Os01g03820.1	KPLIGAARIVINSIHIKGDLLLLPI	S	201			4255	LOC_Os01g33370.1	DANNYRNEIPSSTRVIRHRRKGIPR	T	200
39	LOC_Os01g04190.1	QHVVVAARGDRKSPAIAAPPPATVR	S	34			4258	LOC_Os01g36860.1	SPGASRRHERSATGSGSALPDSGHG	T	658
42	LOC_Os01g04650.1	RLFLFPAKPESSSSLGSLLDDSSKS	S	171			4261	LOC_Os01g37920.1	AAETAEMAATARTARMARALDPAAA	T	47
45	LOC_Os01g04650.1	SLDGIPRGISTDSASVNCLLGLEDD	S	210			4264	LOC_Os01g42350.1	EALRWAALQKLPTYDRVRAAILPMV	T	67

48	LOC Os01g04650.1	DSPMIDKNSSFGSTSSAPSLSNLPP	S	279			4267	LOC Os01g43774.1	LGHFEKPKLPALTKLLADGLTSHDG	T	267
51	LOC Os01g04650.1	STESSPRSRGGDSWDEPFPSSAAAA	S	35			4270	LOC Os01g48320.1	ESPSRSPSPPSPTPAVALPLPNYRQ	T	152
54	LOC Os01g04650.1	DPTNRAMYYNDRSPPAELKRDMPVG	S	399			4273	LOC Os01g49020.1	AFLAGMVTKGSLTLNKAAVIASSQC	T	49
57	LOC Os01g05420.1	EPLMDPYDTRDPSPEPQQQPYDDLE	S	23			4276	LOC Os01g49350.1	SSPAGSAVMHAATKSNMVSQEHHRS	T	261
60	LOC Os01g05420.1	DDTGVDPADRYGSDNDGHSPRHYPQ	S	223			4279	LOC Os01g52390.1	VDKDLQSMQPLSTKKGNDEIFIKLG	T	285
63	LOC Os01g06270.1	STPKAETVVSPKSPAKPDQPLDLDS	S	488			4282	LOC Os01g53000.1	PPRARRMQRTMTTPGTLAELDEERA	T	51
66	LOC Os01g06290.1	RDMSTAANGRSPSPRDYEDNGNHRA	S	304			4285	LOC Os01g53560.1	KTCKRDHTAVGTTEESSPKKVKASP	T	106
69	LOC Os01g07350.1	GHKGGASGGSAISDSEDPFRASQLR	S	400			4288	LOC Os01g56100.1	IPSDSEKANLHITSDDDDKEVEDWL	T	352
72	LOC Os01g07890.1	LQRSSQTFRRSGSSGLVWDERLMLD	S	16			4291	LOC Os01g57450.1	AAQAASAAAIHPTSPRYFFSSLAGT	T	42
75	LOC Os01g08380.1	CFAMNARPLLHASLPRGGAGFYGNC	S	296			4294	LOC Os01g62650.1	ELNMFRSGSAPPTIEGSLNAISGLL	T	65
78	LOC Os01g08430.1	RNKDGHLEHSVNSGEVAVIRQPRGV	S	114			4297	LOC Os01g67160.1	LGRAFTVPMKSYTHEIVTLWYRAPE	T	170
81	LOC Os01g08970.1	LERIKNQAGDEESDEEDEDFVADKD	S	480			4300	LOC Os01g67860.1	TPGKGILAADESTGTIGKRFASINV	T	33
84	LOC Os01g09550.1	QRRRDSGSGSCSSTRDHEVSATSYS	S	302			4303	LOC Os01g70020.1	RPAKGVSKPSQDTEEDDEPEVELES	T	334
87	LOC Os01g09620.1	AAIRRGSWPGVGSPVNDVLASFRQL	S	258			4306	LOC Os01g72790.1	ARSFSSDHNGNLTNRVITLWYRPPE	T	198
90	LOC Os01g09620.1	ASFRQLRLNKVKSSPSGGWSYPSSS	S	277			4309	LOC Os02g02360.1	RDALKESEQSIETSSAAPSIDDDKI	T	289
93	LOC Os01g10350.1	WSSSREEMAREGSPVDGVVQPQQQP	S	117			4312	LOC Os02g03080.1	DDDVEDKDDDDETEDEDSDAEEDPE	T	195
96	LOC Os01g10690.1	AQICAKEEANADSSDDEQAVHYKRG	S	295			4315	LOC Os02g04490.1	RIKRNAPYYTVGTGDTRHFFCIPCY	T	952
99	LOC Os01g10690.1	QSAKGQQQGLPGSSSPKKGTSNKAE	S	197			4318	LOC Os02g05310.1	PERHDPFAAGEATPDPSVRTYADAM	T	139
102	LOC Os01g10840.1	LVPGEPNISYICSRYYRAPELIFGA	S	228			4321	LOC Os02g05310.1	IRTPARKLLATPTPLGTPLYAIPEE	T	414
105	LOC Os01g11140.1	VAQEQQVLSYLLSSLSRDVLTQVAT	S	91			4324	LOC Os02g05310.1	ATPSVRRNRWDETPTPGRMADADAT	T	256
108	LOC Os01g11580.1	TADDNERYVGSSSPVHLEDQKGENA	S	302			4327	LOC Os02g08190.1	KPSAAKSSAAAATPASIDAIDRHLR	T	53
111	LOC Os01g11920.1	APASALASRRSHSGEIPLPSDGPPR	S	81			4330	LOC Os02g09750.1	IPTFLYAMPFSPTRIFLEETSLVAR	T	277
114	LOC Os01g12390.1	ALAFDLEDLLRASAEVLGKGAFGTA	S	341			4333	LOC Os02g15220.1	PNPSPSSSPLAQTLASIRRSIRRPE	T	70
117	LOC Os01g12650.1	ADKLHIGGDGSSSDSDADERKQPKP	S	34			4336	LOC Os02g18660.1	KSGTAKVTSTIATDDDDGTLGGSKR	T	401
120	LOC Os01g12650.1	IADKLHIGGDGSSSDSDADERKQPK	S	33			4339	LOC Os02g19150.1	LLSSRSAGDCSETDSCASDTDGVEL	T	198
123	LOC Os01g12660.1	SFPKLKRNASTSSDMSSLASQGPPN	S	219			4342	LOC Os02g21150.1	RTTSEVFLRAMRTTLPKVQQSGISL	T	154
126	LOC Os01g12660.1	GGGSSTDSSFKRSISETTLEKVSGL	S	159			4345	LOC Os02g33360.1	KYKVKVVADVPGTGSSMDGEVKQAP	T	88
129	LOC Os01g12880.1	FNGQSPAGRNMRSPDQQDEAESSAG	S	160			4348	LOC Os02g34270.1	PPPPSRSVAAATTTLLGGSHGSNGA	T	199
132	LOC Os01g13470.1	KADDSSVAKDTNSEPEAQLELEKGM	S	187			4351	LOC Os02g34680.1	PRNVLYEDDSEETEEEGDNVGDGAR	T	315
135	LOC Os01g14050.1	EKNAGEPGRSLTSILSRKISRKKPE	S	603			4354	LOC Os02g38480.1	LKYEIASRESAITDLREQVEHLSSQ	T	873
138	LOC Os01g14514.1	TATRRLPSPRGGSVHARCSPFRNLV	S	113			4357	LOC Os02g39030.1	TPYHSMIDEDDGTVSPKRTIEESVD	T	54
141	LOC Os01g14920.1	VNDEKRPLEREKSDEEDGPPDPNAV	S	49			4360	LOC Os02g39920.1	KDVVDDGKLSERTISDELPQEPAKL	T	278
144	LOC Os01g15020.1	SPIMNGGDPASRSIDIKPRISEERP	S	723			4363	LOC Os02g43270.1	ARVKAAQDGSRGTSSVSPAVASTDV	T	317
147	LOC Os01g15020.1	PNIGRMDHIDRNSPAKPSPIMNGGD	S	706			4366	LOC Os02g44970.1	RRALVVSDYILETCIVNLYTRCGAY	T	172
150	LOC Os01g15460.1	ISTKDQSVNQVTSPVAASEPVGSIL	S	435			4369	LOC Os02g45110.1	ALPDPARSTRRKTSPGSSPASVAAA	T	102
153	LOC Os01g15630.1	PTDADSMRKARRSPGYQLYRQVSDS	S	179			4372	LOC Os02g48010.1	AAKKGSKQTVEATADDTEGTSKAEE	T	1051
156	LOC Os01g16100.1	QSHSHPAKTLRASPPPPSTAGSAPK	S	50			4375	LOC Os02g49070.1	GSFDATNAVKSVTSSISSASGKHVA	T	157
159	LOC Os01g16110.1	TNNRSHYGGVSESPPSNSIGYFYGS	S	695			4378	LOC Os02g49450.1	DGLDDDFDKIDGTSGLESDDDDDKE	T	280
162	LOC Os01g16110.1	YWAFHHFRNQDSSPIIKHPELERLL	S	876			4381	LOC Os02g52250.1	PVPVMHSPPRPVTVKDQQDWKIPPC	T	253
165	LOC Os01g16110.1	LSSSPHGIPTGSSPVGSVPKSFPPF	S	731			4384	LOC Os02g53580.1	AACANKRLKVEATVTSDGAVVRQRH	T	53
168	LOC Os01g16240.1	FRVFDKDQNGFISAAELRHVMTNLG	S	102			4387	LOC Os02g54110.1	EDGDNMSMTSRATRQSRWNHTQMFS	T	1044
171	LOC Os01g16390.1	RLRYKLEPPAAASSSGEYAPRCYDD	S	153			4390	LOC Os02g54500.1	LLRSGRDFENASTDSEKLSPRFLSY	T	931
174	LOC Os01g16850.1	GREKISCRGFHSSFRPTDSGLSLNV	S	55			4393	LOC Os02g57390.1	ASAVGSSISRSTTPDPQLIRRTPSP	T	294
177	LOC Os01g16870.1	QFIKFDEMSETSSSHGGHTSAGSAP	S	874			4396	LOC Os02g58110.1	EIHKAIKARIQRTLSSMRTPPYVGD	T	142
180	LOC Os01g16870.1	TSSSHGGHTSAGSAPVPELPRLHNK	S	884			4399	LOC Os03g02010.1	WDEYDTAGNCDRTPHSDGSGDEDFF	T	164
183	LOC Os01g18070.1	EKTEFEKLFRKKSAHQDEEHDSMIG	S	235			4402	LOC Os03g04680.1	WRRQRNKAQMLMTTPRFRAFVARSS	T	147
186	LOC Os01g19490.1	EEKDARSLYRRLSALGGAGEGSVSR	S	62			4405	LOC Os03g05390.1	PAGPEVVADDEVTSHRFTPARMSHV	T	230
189	LOC Os01g20940.1	PICSKNVGLSTSSLKLHLQKAHKRL	S	870			4408	LOC Os03g11140.1	SADETDFCSDDETLIEKLENNKTDL	T	550
192	LOC Os01g21590.1	NGHHKRRRGDDVSSAGAGDDDDEDG	S	379			4411	LOC Os03g12360.1	AGLIDDSPLSPSTPSPSPRPRTIVV	T	52
195	LOC Os01g21590.1	DEDGVKRARGAASAAGGGDDEGPSA	S	400			4414	LOC Os03g15050.1	ELHSLQRKRSAPTTPIKDGASSPFA	T	53
198	LOC Os01g23530.1	TVPTVVPARPPNSEGERKSTNFHPS	S	21			4417	LOC Os03g15810.1	AGADQPRRSPQETTAAPSKPADEVS	T	57
201	LOC Os01g23540.1	SPPATAELPKKVSKTNKTPKPDGTI	S	784			4420	LOC Os03g16700.1	FRNKKMYYTRQRTQRSTFKRRKMFD	T	336
204	LOC Os01g23590.1	KRMDDLEKQEPRSPASSSSGSEANG	S	826			4423	LOC Os03g17980.1	LSNVMHDGHFLKTSCGSPNYAAPEV	T	216
207	LOC Os01g23930.1	RRSSVDRSGPSSSRRGYEAAHTSHG	S	1377			4426	LOC Os03g20680.1	ATAQETRDATADTARKAKDATKDTT	T	221
210	LOC Os01g25330.1	DVHDMMLSSPHLSGPEKTSSMSEEP	S	781			4429	LOC Os03g22320.1	AIAAQLQQNALLTRKMNSTKDDSSS	T	428
213	LOC Os01g26940.1	PGKHETNRRRASSRELSPHGRQNSP	S	344			4432	LOC Os03g26460.1	PDDDLEDDDEDDTADSANKDDEDIK	T	233
216	LOC Os01g26940.1	VSLPVGRSPRTHSPKELDSPRPENE	S	301			4435	LOC Os03g27260.1	KKGDNDLPGLTDTEKPRMRGPKRAS	T	129
219	LOC Os01g27790.1	RSGRRDRRRKKGSARFDDEGHVLAV	S	440			4438	LOC Os03g29260.1	VKVESTACSVSPTADQKAPAADEED	T	89
222	LOC Os01g28560.1	KLNSDEITSTSNSKTKVQKTGPAKD	S	216			4441	LOC Os03g31750.1	AAAGILTARGGMTSHAAVVARGWGK	T	467
225	LOC Os01g32130.1	ASSGKADSTKDASDTDDALEVDFNN	S	131			4444	LOC Os03g40010.1	SSSKKFDDDDDITPSKKRNKALEYD	T	479
228	LOC Os01g32870.1	GAGSSSSRYPTESPENGNIDGKDKS	S	374			4447	LOC Os03g43800.1	SGDRMFLSPVKSTDRKNADSEMGSS	T	352
231	LOC Os01g33000.1	LGRVKHLTQEGFSSDDGESGDLHGR	S	201			4450	LOC Os03g46046.1	RILGGQLRSTVGTGTKLREELRSRR	T	40
234	LOC Os01g33090.1	LANIAGGSPFTQSDHAGIEKAQSQQ	S	567			4453	LOC Os03g46590.1	DDGEGPSAEVNETSVEEVNAREDRG	T	133
237	LOC Os01g33800.1	GELADNENKNPASPTRQEQPSSNFD	S	225			4456	LOC Os03g49170.1	AANSMLSPVSSPTAEDARSPSAAVM	T	232
240	LOC Os01g34330.1	RRGRKPKRPAEESEEDEEEEEEEAK	S	113			4459	LOC Os03g50480.1	ADGSKATGAFILTASHNPGGPTEDF	T	122
243	LOC Os01g34780.1	HKRHHHDTADDVSSERDEKDDSKKS	S	378			4462	LOC Os03g51520.1	KKQKLISRAEQPTGHNSSDQDELRT	T	762
246	LOC Os01g34780.1	KRHHHDTADDVSSERDEKDDSKKSR	S	379			4465	LOC Os03g52310.1	TPAVGTDNDGEGTVERPSKRRYRYT	T	144
249	LOC Os01g35050.1	YFPKWYLKGARESPSHGGAFVRKFV	S	50			4468	LOC Os03g53920.1	DTQYEDVEMRDATDDEDLEVKHVHD	T	838
252	LOC Os01g36860.1	EDGKVGKVDLDRSPTPQDKSGPYSP	S	709			4471	LOC Os03g54780.1	MQEAKTMPPSTGTVKKLKVAEGTMP	T	665
255	LOC Os01g36860.1	KAAEVSKNPDPSSPPHHGKTREDEE	S	754			4474	LOC Os03g57430.1	RSSPRGNFLPSRTIGSDLTDGGSPI	T	194
258	LOC Os01g36930.1	GKASSSKENEGSSSNAGESSSMDID	S	380			4477	LOC Os03g61930.1	LQSKTKEHASDGTSSVLEPEVELSH	T	499
261	LOC Os01g37080.1	ASILTIMMAVGASKERAPKGHDGAG	S	305			4480	LOC Os03g63940.1	MNMDVDNDNFQRTVSLSDGIIQEGP	T	134
264	LOC Os01g37790.1	KSATSPRKMRRLSSFASSRRDGASR	S	724			4483	LOC Os04g01290.1	ASAINTIQANKVTDDGSQGIQGLMI	T	403
267	LOC Os01g37825.1	SHVEPRFSCFVESEAAGSAVVVSCK	S	299			4486	LOC Os04g06770.1	DMSDASSSQGGHTSVGSVPVPELPR	T	887
270	LOC Os01g39700.1	GKKHQDCRPKRLSIVKKERRRAISQ	S	66			4489	LOC Os04g17064.1	LVEQMLEPGKRVTIAMGTNRDITTA	T	213

273	LOC_Os01g40050.1	NEDHQLTKSRKFSMKKKGIGSEASA	S	358			4492	LOC_Os04g23060.1	RDNTSAINIAKNTVQHSRTRHIDIR	T	646
276	LOC_Os01g40110.1	NNLSVNSGEVVQSSNEEAETKAKSK	S	202			4495	LOC_Os04g24210.1	KKFVSQLGVMTSTSRPMDLYCDNSG	T	621
279	LOC_Os01g40370.1	RRPTSLHDCGRDSDDDDFRDMDYDV	S	67			4498	LOC_Os04g28090.1	MTPSRDGSSFGLTPKSTPFRDELRI	T	439
282	LOC_Os01g40590.1	RAASLGNPIGSQSMKAQGTFARNGQ	S	455			4501	LOC_Os04g33110.1	RSSSHGHPSSSPTAAAAPAAAVQSS	T	88
285	LOC_Os01g41280.1	PHISAAAGASLRSIRDAVANAVTRV	S	106			4504	LOC_Os04g35240.1	IVEEARTPPRSSTPVAGFGWQEEDE	T	309
288	LOC_Os01g41610.1	ELSKQLFYTRLASLPGRYEAFWKEF	S	61			4507	LOC_Os04g36890.1	NGDDSKLQLVVRTPPAESLESEDED	T	223
291	LOC_Os01g41880.1	KENVERDERTRKSLSINEFLKPAEG	S	313			4510	LOC_Os04g38570.1	SGSLRNLSYSYSTGADGRIEMVSNA	T	655
294	LOC_Os01g42250.1	ARIPTEHIDLDKSPERHEAREKVVV	S	154			4513	LOC_Os04g41100.1	GMDSEYEVRRSETPEPVKPPHRCIN	T	342
297	LOC_Os01g42380.1	EIQKVASMRLGGSMRGDSGSMWRRG	S	18			4516	LOC_Os04g42320.1	HNLKKSHKLDTSTDSEVVDHSKAVN	T	394
300	LOC_Os01g42820.1	PGPGFRGQEGGMSPGPGGRAPMYPN	S	430			4519	LOC_Os04g46894.1	PERKAAPKLQSFTSFQKPIETAPNK	T	1537
303	LOC_Os01g42830.1	AEEMVQHYEKSSSVDYSLKEGETLV	S	162			4522	LOC_Os04g51700.1	DDVEPEESDHGETGLDDQIPDTDNI	T	884
306	LOC_Os01g42850.1	MRHGAGPGTNCGSPDVVAAADTLSA	S	865			4525	LOC_Os04g52570.1	TPGSTPKFLSGPTSPTKSLSEVHST	T	204
309	LOC_Os01g43050.1	TEKLAVDINNALSPSKGKEQRGNNK	S	673			4528	LOC_Os04g52960.1	PAAPLKKPVALATNGSKKVETDSSS	T	171
312	LOC_Os01g43170.1	RDYTNPYLPVAPSAIDGSGPDGKKQ	S	220			4531	LOC_Os04g55220.1	ETPNPFRQQFSMTSLERTMTSMENG	T	408
315	LOC_Os01g43330.1	LLLSPSRSGDIESPKDILCERDAEC	S	369			4534	LOC_Os04g56630.1	QADSRSVDSAKSTDDDEEDEDEIEA	T	483
318	LOC_Os01g43330.1	AECQVEPSDARVSPCSPVLEDKLVD	S	391			4537	LOC_Os04g58750.1	LMKNSRDGKSYSTNLAFTPPEYMRT	T	80
321	LOC_Os01g43330.1	KHEFESKKDDTNSLEGESLNKEHEQ	S	514			4540	LOC_Os05g02670.1	EEEYLVDEPSCSTPRRRQIDLPSME	T	988
324	LOC_Os01g43420.1	DQPGVRHLSSFSSFDNPRGRQIQKA	S	268			4543	LOC_Os05g03120.1	NETDSAGITPTRTTPERSPKVTERR	T	45
327	LOC_Os01g43920.1	TNKTVFKSAGIMSTLIVSSSKSNVV	S	309			4546	LOC_Os05g03550.1	AADVIPSDPCGKTGSGIDVDIEQRE	T	489
330	LOC_Os01g45550.1	VALGGGGGGENFSFGGGKTVDGAEA	S	393			4549	LOC_Os05g05370.1	CDHYECSFRKDKTPDKIRYYSRANI	T	281
333	LOC_Os01g46710.1	SAREQRGSADKSSGSGRLDALVERM	S	464			4552	LOC_Os05g08970.1	GRAGGVTAVLQSTDDDAVDPHLERI	T	459
336	LOC_Os01g46710.1	HRSHRRGGSVERSESEGDEAGGGAG	S	24			4555	LOC_Os05g11980.1	PEISQKQEALQDTYSGDEIIDSMHR	T	1083
339	LOC_Os01g46710.1	LALHKNQTAKIVSPTHSILETDRKT	S	1020			4558	LOC_Os05g22390.1	RYCKWHNSRSHSTNDCKLFRQQIQV	T	253
342	LOC_Os01g46980.1	LVDHDVYLPPSPSSHDSHERFCSGG	S	177			4561	LOC_Os05g23940.1	EARESDVENNITTPPKNIIGDKHST	T	372
345	LOC_Os01g48180.1	MLSNAKAATVEESPRSSGADEWGLE	S	1464			4564	LOC_Os05g28190.1	TDKSAGSSEVAETEKDGSDLKGSDE	T	133
348	LOC_Os01g48180.1	FERTAPSTYCVKSPYRKDPADSEVV	S	623			4567	LOC_Os05g32600.1	LARIFGSPERNFTHQVFARWYRAPE	T	174
351	LOC_Os01g48280.1	PRRIIKETQRLLSEPAPGISASPSE	S	19			4570	LOC_Os05g36350.1	TRKAIRSRSMRKTPGCSLIEVGNVV	T	489
354	LOC_Os01g48320.1	MRRRLESPSRSPSPPSPTPAVALPL	S	147			4573	LOC_Os05g39520.1	LRPDRWFVLRDTTEMIKKMRPVLKS	T	615
357	LOC_Os01g48320.1	GLPMMRRRLESPSRSPSPPSPTPAV	S	143			4576	LOC_Os05g42230.1	PVAGGEKATPPSTPPPVRLAGGAAA	T	20
360	LOC_Os01g48330.1	NGSTQKAMSLPSSPHEYRAQISETI	S	491			4579	LOC_Os05g43380.1	QSPISKESSFQQTPPHIRATNRDTH	T	258
363	LOC_Os01g49190.1	HLDATTVLSRQISELGIYPAVDPLD	S	418			4582	LOC_Os05g46780.1	PRSSRSAAHGEDTSSVTDAASRSVD	T	301
366	LOC_Os01g49250.1	SDDERSVAADSWSVRSEYGSTLDDD	S	61			4585	LOC_Os05g50160.1	ERAKGGPAPALPTATGGGKRLLEET	T	186
369	LOC_Os01g49250.1	MASDDERSVAADSWSVRSEYGSTLD	S	59			4588	LOC_Os05g50530.1	SARNLPKDIPLPTESVSRGKRRAVS	T	177
372	LOC_Os01g49350.1	DTGKYQAAPQSPSSPAGSAVMHAAT	S	249			4591	LOC_Os06g01700.1	DESDEDDDDDDDTEALMAELERIKK	T	133
375	LOC_Os01g49529.1	LLKYSGSGGTPRSMGGDMESGSVKD	S	353			4594	LOC_Os06g03780.1	EQSREYLALHPQTSLKEPRLIEEHF	T	529
378	LOC_Os01g49640.1	NPDLRKYINSQNSRKVAAACSVPAP	S	89			4597	LOC_Os06g05190.1	PRSDDAQYDSDDTIEMTEEEIDLAC	T	325
381	LOC_Os01g50310.1	AAVAAEKTLPNGSVEEDEVTVTAAE	S	56			4600	LOC_Os06g06014.1	EWPLAPSPPASPTTWHDNKAASSPA	T	87
384	LOC_Os01g50320.1	RARKYRKKHHPESDEENYDSEESYK	S	725			4603	LOC_Os06g06830.1	ERRRNDRSAPHATEGASSSRVSPQH	T	119
387	LOC_Os01g50450.1	GVLVKDVVEVSRSLGAADTTEVARG	S	219			4606	LOC_Os06g08480.1	EEVMLDASSKETTSPKDPATEVPSS	T	1204
390	LOC_Os01g51010.1	TKTAKSYFDSDDSEDELAQRKEVQT	S	950			4609	LOC_Os06g13390.1	KKVYLNEENIYITKTTPQSVVKLFQ	T	155
393	LOC_Os01g51010.1	EFNHSRERKSSVSGSNWNIEFKDAT	S	340			4612	LOC_Os06g14412.1	SQAPYDASDKEDTAADLIGPKQATL	T	2483
396	LOC_Os01g51200.1	TSEPSRTRATDASPGAFRRTSGPQK	S	416			4615	LOC_Os06g17840.1	KKRDDSVDTELVTSPASVKTPVSKG	T	1279
399	LOC_Os01g51390.1	PLTNLPELSWFRSHTYSDDEFSSRT	S	485			4618	LOC_Os06g26340.1	VAFTDAPTTVFWTDYVATRWYRAPE	T	188
402	LOC_Os01g51640.1	EEVPTVESEENESPQSKHCTSVEVI	S	335			4621	LOC_Os06g30710.1	KAKKYDLEDLRKTPNPFYVESGKNA	T	330
405	LOC_Os01g51890.1	PSMKIFDRSHNLSFKDYVSSLEEPI	S	318			4624	LOC_Os06g34710.1	VTTPATEKVEVTTPATEEAKITTAA	T	896
408	LOC_Os01g53000.1	ERAGSVTSDVPSSLASDRLIVVANT	S	73			4627	LOC_Os06g34710.1	VTTPATEKVEVTTPATEKVEVTTPA	T	886
411	LOC_Os01g53000.1	AELDEERAGSVTSDVPSSLASDRLI	S	68			4630	LOC_Os06g37770.1	PMAVGDAATAVSTLSCRAPRPRRSS	T	72
414	LOC_Os01g53680.1	PNLRALRNASAMSLPDDDAAYAKLE	S	70			4633	LOC_Os06g46890.1	RFERFADLLASDTLKPCSKDQGSSA	T	244
417	LOC_Os01g53980.1	GYLARKALRALRSLVKLQALVRGYL	S	114			4636	LOC_Os06g50890.1	ITVEKSRRGRPRTPTPGSYLGHRYD	T	148
420	LOC_Os01g54920.1	EEDDIEESEEEESEDESEGKAKHKG	S	57			4639	LOC_Os06g51250.1	TDAFARFDSMKSTDYNSRGYSFDED	T	1149
423	LOC_Os01g55260.1	KEAAMQPLRRLMSVLEARDELVPEE	S	326			4642	LOC_Os07g02230.1	LIENDDGSCGSETKNVVYHEILDAT	T	1401
426	LOC_Os01g55280.1	VVRTESGDKENQSAAGAEDGEEVSC	S	174			4645	LOC_Os07g04530.1	RSAVAQQQPPSPTPAKVSINQRNHA	T	1481
429	LOC_Os01g55410.1	KGDDDDDDEERFSDALDTLSRTESF	S	170			4648	LOC_Os07g05610.1	VYSGIDLSKDEVTQIAKLPANDDYD	T	825
432	LOC_Os01g55650.1	SWNLIARENSSGSLEEEFLISPRTN	S	615			4651	LOC_Os07g08330.1	IKARKEKLDSKRTKLSPEEAAKVKA	T	364
435	LOC_Os01g56100.1	QILKAREVLLQSSPTKKRLGPDDGS	S	262			4654	LOC_Os07g10400.1	GVAHFQDQGGNMTAETGTYRWMAPE	T	627
438	LOC_Os01g56140.1	LSLLQHDKSKLGSDINVKDNHHDNS	S	752			4657	LOC_Os07g18300.1	PLEKCCDAKVRRTFSKIDLKLIERL	T	806
441	LOC_Os01g56300.1	QQGDAKPLSRTSSYPQQPLQHRASE	S	169			4660	LOC_Os07g25680.1	SLQPSDSAKSLETYTASKAMSAAQD	T	512
444	LOC_Os01g56800.1	KVDTSEEGASDVSDNLDGSIEIQKK	S	549			4663	LOC_Os07g32430.1	DARLVSPWLGGNTPRYAASPWDNVS	T	282
447	LOC_Os01g56910.1	SAIAKTSFRRKQSQLQADSATIEPI	S	871			4666	LOC_Os07g35580.1	YIRKGGGDDGGGTGSYSGSFVGTLP	T	663
450	LOC_Os01g57450.1	AQAASAAAIHPTSPRYFFSSLAGTN	S	43			4669	LOC_Os07g37140.1	NVNASPASPPAATSDVTRDLPNGLM	T	362
453	LOC_Os01g58580.1	RMTLFNIFGDDASPKVKKFMKVMLG	S	264			4672	LOC_Os07g38730.1	DGAINVDVTEFQTNLVPYPRIHFML	T	257
456	LOC_Os01g59670.1	ASHRDMFFRRHDSFNIGRTDATLER	S	579			4675	LOC_Os07g41180.1	NVEKAVSSPVKPTNAADTTSPNDKN	T	300
459	LOC_Os01g59760.1	QHQPGSGLLRQGSITMPPELSKKTV	S	86			4678	LOC_Os07g42950.1	IVKKGENDLPGLTDTEKPRMRGPKR	T	127
462	LOC_Os01g59990.1	IRADSQVFLFANSKCKRYFHNRLKP	S	36			4681	LOC_Os07g47100.1	IRLLLPASGHPVTSEPSSPKSLHSP	T	453
465	LOC_Os01g60040.1	PIAPIPSPKFAPSPVHTKSINNKTD	S	1869			4684	LOC_Os07g48020.1	FASNAAAFSSAFTTAMVKMGNISPL	T	289
468	LOC_Os01g60190.1	AVGLPSEDDMGNSEVGHNALGAGRI	S	81			4687	LOC_Os07g49460.1	PSLELSLKRSRSTGDGANAIQEEQR	T	417
471	LOC_Os01g61370.1	SWESSPGVPKRSSACMHMAQEIMPP	S	84			4690	LOC_Os08g02690.1	AETLSLASPRSPTGGSTSALLQQYE	T	37
474	LOC_Os01g61590.1	KASRHYSRGRFNSLSMKLIKDGSVK	S	535			4693	LOC_Os08g03520.1	AVDVTAPGGGALTGGSRPSGGGDRG	T	78
477	LOC_Os01g61780.1	GTSEMDPDSEPDSARSRHDDFNFSE	S	689			4696	LOC_Os08g08080.1	EEPKDLSPPRRRTRHDSEEPEDLSP	T	284
480	LOC_Os01g61860.1	ELQSANNEEIKPSEDSDDEFYDVEK	S	152			4699	LOC_Os08g14340.1	KSANMIISEGGGTSGYSKILPTVLS	T	391
483	LOC_Os01g61860.1	NFLDSQAEYDESSGEDQDAKVSPSA	S	62			4702	LOC_Os08g20660.1	ESTRGRMPRIGSTDAIEAWASQHKD	T	171
486	LOC_Os01g62310.1	FSDPPSPLSPPLSPASAAAAALANA	S	31			4705	LOC_Os08g25570.1	SRLWSGGAYESDTEDPDNMKTETND	T	431
489	LOC_Os01g62650.1	SKDGTLGDSNSISQSKFQSEISDHQ	S	371			4708	LOC_Os08g27150.1	RRPTKSAPSNLLTKREALQRSIEVQ	T	686
492	LOC_Os01g62650.1	RINSNEKKLNCSSSFNTASSKAVEP	S	334			4711	LOC_Os08g28880.1	RRQATAAAAALPTPRSPPPAFGSIV	T	44
495	LOC_Os01g62650.1	DGLIGLSLGRQRSFADILQDNIGRR	S	214			4714	LOC_Os08g35270.1	KDVDRKLNLVQCTPVEKTLYASHQQ	T	455

498	LOC_Os01g63950.1	LASNICDQLDPASPKCSANSTMSDL	S	219			4717	LOC_Os08g39140.1	ISYPISLWTEKTTEKEISDDEDEEE	T	215
501	LOC_Os01g64250.1	TTVSPYALARSPSVSAAEADGDDGV	S	50			4720	LOC_Os08g40230.1	RDFESDFEEMHDTDEELDNLYNARQ	T	122
504	LOC_Os01g64650.1	IESVLQRGERLDSLVEKSSDLSAAS	S	187			4723	LOC_Os08g42980.1	YGHSRDANHPIGTPPSNHSALADHP	T	385
507	LOC_Os01g64790.1	GYYQAQEMSTMVSALARVVAGGGGG	S	24			4726	LOC_Os09g01640.1	SSSRGRSKHRRSTKRSDTEDDLEEE	T	234
510	LOC_Os01g65230.1	VRSHISRLASIDSFDSRWQDFLEPG	S	389			4729	LOC_Os09g07294.1	KAVTSYLSSSPSTPESPVRLSALSG	T	518
513	LOC_Os01g65560.1	RASRRPQEPAFPSEDEDDFAGGEVA	S	42			4732	LOC_Os09g17730.1	RRRAGAADTEASTESDNRSDGAAEA	T	533
516	LOC_Os01g66130.1	ITDGSASADKSGSPEHCQLVAALHP	S	327			4735	LOC_Os09g26820.1	DDLRRLAPPSPATVGSPRRTSSASG	T	70
519	LOC_Os01g66140.1	LALFVEKNSEANSDGETVEFRYTEN	S	123			4738	LOC_Os09g32430.1	KGHDEVSRSKDGTDTEDATIDTDRS	T	149
522	LOC_Os01g67126.1	KASLVERLNALNSSAGADDDDEEED	S	287			4741	LOC_Os09g33510.1	VGDLRDNKLPSPTRLSNAHSVLKES	T	399
525	LOC_Os01g67170.1	LVQNDGNSIALDSDDEDLIRGSHKR	S	422			4744	LOC_Os09g36090.1	IKEMVTIACDAATDDEGRKVDAVSS	T	201
528	LOC_Os01g67250.1	TVPSRVHESPVLSPQRKASPSSNDE	S	320			4747	LOC_Os09g36160.1	PTKRPRARPSATTPTTSSGDQQMVT	T	177
531	LOC_Os01g67330.1	EHRSKESVVVNVSDSIATEAKHRHG	S	380			4750	LOC_Os09g38390.1	EANPEHEPYLDDTNSGPLERNVNSD	T	320
534	LOC_Os01g67580.1	GPVGSGKSSLLYSIMGEIPRINGAE	S	582			4753	LOC_Os10g10990.1	EKGDDWEHEETFTDDDEAVDIDPEE	T	295
537	LOC_Os01g68120.1	VKIASSRESGNLSPGFVPNEEINEY	S	507			4756	LOC_Os10g30350.1	KIELFRTTEERTTPNAIPINDTDAQ	T	201
540	LOC_Os01g68810.1	KSSIVKDESDPNSASKRRKIKRDQS	S	1696			4759	LOC_Os10g34370.1	NCRIQWFAKKGETFKGEELLDAMES	T	275
543	LOC_Os01g68810.1	KMPRNPVYMMDTSPSSSQSFSVDPP	S	457			4762	LOC_Os10g41030.1	SACISYDSHTEETGLRKEWVPLRQE	T	1597
546	LOC_Os01g69030.1	SDGDDGKDFEIASPRSLPPIWAEVM	S	420			4765	LOC_Os11g02190.1	PSPIESPLSRSRTFEDNPIRHSTGK	T	623
549	LOC_Os01g69100.1	QQQAAAAGVGVRSPGSGSKDEMFFD	S	60			4768	LOC_Os11g07040.1	MRKGNAGGAGRRTMRNSLQLNLGEI	T	495
552	LOC_Os01g69130.1	YTASTSHLEHSLSTIQLREPPVVLK	S	641			4771	LOC_Os11g19250.1	VVSHFTEKFDEETIGGTEMADDENL	T	443
555	LOC_Os01g69260.1	EAETDRVSTRESSEKWSASAYVYLQ	S	71			4774	LOC_Os11g28270.1	DHPMGTVMYGLATSPTGDVSARRML	T	391
558	LOC_Os01g69900.1	AMSIGPLDDDDDSRSEGSTKVASEA	S	388			4777	LOC_Os11g29380.1	DRKLPRMLHDQDTDEDMSFRRPKRH	T	148
561	LOC_Os01g69990.1	NSGKLGLRSSSGSMLDMQRGDFSGI	S	1321			4780	LOC_Os11g32900.1	SILKAKYHPLQATFASPTVSNPVSG	T	686
564	LOC_Os01g69990.1	STNKEAGSFISPSGTSVDGPSFREM	S	1457			4783	LOC_Os11g35590.1	KSLGRTRVAGSGTLLSLCLRAVAST	T	1318
567	LOC_Os01g69990.1	HFDLLQSLQRSSSVEQHEHLERSLS	S	1068			4786	LOC_Os11g41890.1	TTWRSGSSELDGTSPFGYGLGNAAS	T	442
570	LOC_Os01g70020.1	VKHIIEEVINSMSDDEEGEEDNAED	S	477			4789	LOC_Os11g43970.1	KRPKAEAAPASPTASVPGRIEEDSA	T	41
573	LOC_Os01g70020.1	DDEDSEAKDNAGSDEEPDEPPAKKK	S	287			4792	LOC_Os11g47830.1	SDRRDAFIGHDETPPSAEWGSKLGT	T	478
576	LOC_Os01g70160.1	VQREGLVSRREKSINLTQEESLALR	S	110			4795	LOC_Os12g06200.1	EKFNAEDFLEVSTPRQQDKPSSDIM	T	427
579	LOC_Os01g70330.1	NPGIQLPLKKAVSFQDDNRHTVGPS	S	315			4798	LOC_Os12g06850.1	HNSGGDGGSSVLTDGDSPDKIQGRS	T	192
582	LOC_Os01g71000.1	EDRRSVRSPNHHSPDLRREAARSSR	S	402			4801	LOC_Os12g06980.1	RKSVVSTLLEEGTKCMHGSVRGDVL	T	533
585	LOC_Os01g71230.1	KDIELVMTQATVSRSRAVKALKAAN	S	178			4804	LOC_Os12g07530.1	HPVQHGYSSFDDTHEFSDQEVSPVV	T	566
588	LOC_Os01g71970.1	AAAVAGGDKERRSPESGLSPSTSRA	S	275			4807	LOC_Os12g09739.1	EALPFHMEHLRVTDLDSNRYRWLCI	T	521
591	LOC_Os01g72320.1	VHKDGSDHYRLRSPFGSPPKNALDK	S	177			4810	LOC_Os12g10410.1	LGVWGMGGAGKTTLLKLARDPRVQT	T	220
594	LOC_Os01g72710.1	LAQLGRKLESLPSDLAAAVEGGRVT	S	121			4813	LOC_Os12g19040.1	LKDTDSVQPAVSTPLTETGIKGGSH	T	447
597	LOC_Os01g72890.1	RRSPSPPPRRRRSPMRPSPRRLRGS	S	303			4816	LOC_Os12g25700.1	WDHPRHLQPTSPTAFKQVSVVWDAY	T	395
600	LOC_Os01g72890.1	RRLRGSPSPRRRSPGPIRRRSPPPP	S	322			4819	LOC_Os12g37720.1	SASVADDIDDLDTKKTSVRDKPYIS	T	858
603	LOC_Os01g73160.1	GEKGGAPAEFQPSFRGSRPGFGRGG	S	155			4822	LOC_Os12g41140.1	QPYRGVVGGGGKTVGRWLKERKEKK	T	161
606	LOC_Os01g73620.1	GFTASSNLRASYSPNSICSENASDA	S	855			4825	LOC_Os12g44330.1	QRTTGVSGLGEGTPKSERSADMFCD	T	548
609	LOC_Os01g73880.1	HETRPPSAGRPPSSGRGRADDADER	S	17			4827	LOC_Os01g01150.1	PIKYRRSRRSRSYSPPVRHTRGRRS	Y	544
612	LOC_Os01g74020.1	ASAATVSRLRRASSSSSSSFPAFAS	S	76			4830	LOC_Os01g05420.1	FIDDTGVDPADRYGSDNDGHSPRHY	Y	221
615	LOC_Os01g74030.1	ASPDPELVRRVPSPCLPPIGVKLGA	S	236			4833	LOC_Os01g11580.1	PPKENTADDNERYVGSSSPVHLEDQ	Y	297
618	LOC_Os01g74030.1	ASIIGSSLSRSASPDPELVRRVPSP	S	225			4836	LOC_Os01g35050.1	RQSRRNTPVPSKYNGSESPSLAEIV	Y	751
621	LOC_Os02g01020.1	RALSRDSVYDLDSEDEQWLTQLNHS	S	650			4839	LOC_Os01g48610.1	FSLYITTHGGYGYASPVTLGSAMVD	Y	456
624	LOC_Os02g01170.1	PSDTGMQIDRSASPDDDLAAKELSS	S	469			4842	LOC_Os01g53140.1	VAKTKKPIIWGVYLRNSQTKANSYW	Y	46
627	LOC_Os02g01380.1	AGGNGGEEAKGSSELLDAVVKYPDV	S	297			4845	LOC_Os01g67580.1	CGPVGSGKSSLLYSIMGEIPRINGA	Y	581
630	LOC_Os02g01490.1	GKQSHSRSNSNSSVQFNSLTEDLHE	S	1499			4848	LOC_Os02g07070.1	DRDYDRYSYDSDYEKSRRDGSWRRR	Y	143
633	LOC_Os02g01510.1	SRSRSRDMKKASSLSELGFDAEGAS	S	45			4851	LOC_Os02g13250.1	ELENLSSSQKKGYFKPKREKDVLST	Y	251
636	LOC_Os02g01960.1	KQKSLENLFVTNSQRVKKDAGRRWS	S	581			4854	LOC_Os02g18910.1	FVLEKEDYPVVDYVLVIYAAKSATA	Y	47
639	LOC_Os02g02290.1	PPAPSSSSKKLRSLSALDSRPGALS	S	1707			4857	LOC_Os02g31220.1	PSKSRSSGDHHAYLQVDAVGRVSFS	Y	462
642	LOC_Os02g02290.1	VKKKRDRRPSLGSPSSSGRTGPLSP	S	2111			4860	LOC_Os02g35190.1	QHRAPEREGSHNYDIESTDGSGGLW	Y	18
645	LOC_Os02g02360.1	SEQSIETSSAAPSIDDDKILSENGL	S	295			4863	LOC_Os02g38840.1	PPEADELSKRMGYVQTHGYVWIPPT	Y	501
648	LOC_Os02g02740.1	PPPVEKRPIKTASDLAAAIRAAADA	S	71			4866	LOC_Os02g47220.1	FGLAREVTSVPPYTEYVSTRWYRAP	Y	155
651	LOC_Os02g03040.1	GSPDYGRGGDRGSPDYHRGASPQGG	S	229			4869	LOC_Os02g54510.1	RYGEMDSPWSPAYISPGHYGVHDPR	Y	213
654	LOC_Os02g03040.1	GDRGSPDYHRGASPQGGNKGDERGS	S	237			4872	LOC_Os03g19530.1	AAAKAVDAGMMEYDSDDNPIVVDKK	Y	149
657	LOC_Os02g03080.1	SDAEEDPEKIAMSESDEGDDDEEDD	S	212			4875	LOC_Os03g21530.1	KKLALKAKFDAEYDGSDLSGEEVDN	Y	720
660	LOC_Os02g03740.1	ESDTKAFSALSLSSSSRDTQDGSKN	S	234			4878	LOC_Os03g24890.1	SVSRSPRSRYHSYSPSPSPARRDYR	Y	178
663	LOC_Os02g04050.1	HVSKTEVMNLLESAGFSRSNPYYVV	S	127			4881	LOC_Os03g53280.1	EPQKSNMNRTASYASGISPESSLDD	Y	1500
666	LOC_Os02g04270.1	GDGAEVTKDGDDSDDSDGMDDDAMF	S	992			4884	LOC_Os04g02870.1	VSPVYRSRPSPDYGRQRSPVYGSYD	Y	205
669	LOC_Os02g04560.1	SSDLSGKGTHEFSSELDGDKSSASV	S	361			4887	LOC_Os04g06910.1	LCHHFKVMGVNRYFIPSRRRFGSMG	Y	333
672	LOC_Os02g05330.1	LGVKVHACVGGTSVREDQRILASGV	S	148			4890	LOC_Os04g16360.1	KSIGGWSGYVAAYVGQGSVTPRECM	Y	48
675	LOC_Os02g05450.1	YTSRRALQQRTESSSELISVSKRAT	S	23			4893	LOC_Os04g40660.1	LDIPNAQEKFGAYVDLATERGWLLP	Y	647
678	LOC_Os02g05510.1	YSKKMNFPHRMASLMRFREKRKERN	S	164			4896	LOC_Os05g11980.1	EISQKQEALQDTYSGDEIIDSMHRS	Y	1084
681	LOC_Os02g05900.1	DVGEDPEERQMVSLNRLFEKHRDVS	S	29			4899	LOC_Os05g40230.1	KSLLRVTKDATAYRKVLKGLIVQSL	Y	110
684	LOC_Os02g06920.1	GQQVKATTSRSFSFTKVINSLSAKR	S	129			4902	LOC_Os05g45830.1	ADREKAEETEEEYESDLDDAPLPAV	Y	14
687	LOC_Os02g07070.1	GRNQRDGFYDDNSFGRRREYDWDER	S	218			4905	LOC_Os06g02130.1	YESKEFDDVSEQYVAVTKKEKRGRT	Y	186
690	LOC_Os02g07190.1	WLLKRAGLDDSLSESFRRTEIFKDK	S	807			4908	LOC_Os06g07350.1	NIPAPPGAERTLYPSMDPQRMGALV	Y	368
693	LOC_Os02g07210.1	GRSLGKRLSHLDSIPHTPRVNGQIP	S	52			4911	LOC_Os06g08840.1	RYRRSPSYGRRSYSPAGRSPRRRSV	Y	140
696	LOC_Os02g07870.1	FGCGKTVISQALSKYSNSQAVVYVG	S	264			4914	LOC_Os06g13390.1	QVPEELNGRKKVYLNEENIYITKTT	Y	146
699	LOC_Os02g08300.1	LVVMLSKSKASGSSGALSSLTSSTP	S	82			4917	LOC_Os06g26340.1	FTDAPTTVFWTDYVATRWYRAPELC	Y	190
702	LOC_Os02g08350.1	GHVISGSKDASDSLLSAFKANDCNL	S	703			4920	LOC_Os06g39600.1	TEKTFENMDAVAYMPVVRSGGWADI	Y	62
705	LOC_Os02g08360.1	DRKGGRGLGLSYSSGSDIAGDSGKA	S	790			4923	LOC_Os06g48530.1	REYDRAERDGSRYGRGDRSPSLDGA	Y	593
708	LOC_Os02g08360.1	GGRGLGLSYSSGSDIAGDSGKADAT	S	793			4926	LOC_Os06g50890.1	RRERSRSLPYSPYRMPERGYGRQAG	Y	255
711	LOC_Os02g09750.1	RRIPTFLYAMPFSPTRIFLEETSLV	S	275			4929	LOC_Os07g01880.1	VVASESEGSEDDYYAGRGHEDEEPH	Y	181
714	LOC_Os02g10020.1	EPVPQVPKSTKPSILKLRQATSRNL	S	365			4932	LOC_Os07g08190.1	RGFTQQYAFARRYRTPSPERSPVRS	Y	459
717	LOC_Os02g10220.1	SSIMSKCDPKGSSSSEPTEEEFEQA	S	257			4935	LOC_Os07g31460.1	SSTTRFPRYNDPYKLLETRKGRCGE	Y	314
720	LOC_Os02g10650.1	ELASCKELIKMGSLHVSIPEPDPDD	S	289			4938	LOC_Os07g41190.1	RSGGDGEDDLPVYDEGLSSEDEGVD	Y	121

723	LOC Os02g10920.1	SGAVRASRPGWKSGDWICTRSGCNE	S	132			4941	LOC Os08g02690.1	DTDACLDRNDPNYDSDEEPYELVEA	Y	116
726	LOC Os02g10970.1	DDKSKRHHRGRKSKASSQVSGENHT	S	342			4944	LOC Os08g17120.1	SAPAKEAIKLSDYERTLKKASSRKS	Y	508
729	LOC Os02g10970.1	KRHHRGRKSKASSQVSGENHTALAA	S	346			4947	LOC Os09g28220.1	PYRSRRDRSPSPYRDRRRQWSPYHR	Y	62
732	LOC Os02g11750.1	NDRVKMSEKELQSLIDDAMGEVDIG	S	314			4950	LOC Os09g33980.1	PRRSIRGQRLHPYQESEDDQESSEE	Y	155
735	LOC Os02g11750.1	SRDLSVSSDDLHSDAKRQLNKAIQM	S	226			4953	LOC Os10g20600.1	ANYVERQNSEISYYADDEDANRKKY	Y	258
738	LOC Os02g11780.1	AEKIVSEADLPSSQDKSESKLESPK	S	615			4956	LOC Os11g01836.1	VGDKSHPLSDQIYKKLDELGLEMKT	Y	539
741	LOC Os02g11820.1	SENRRSDGNWGGSRSPPYNESYSDR	S	154			4959	LOC Os11g44880.1	ESKQVGRSAPLKYGGILKPSMSGKH	Y	184
744	LOC Os02g11820.1	NGSPNYQKETDGSSPVVRPVRDILG	S	246			4962	LOC Os12g06850.1	RSYQNAQVSDFKYSPSPQRHNGLSS	Y	215
747	LOC Os02g12360.1	DQDISGAGKKWDSSEEDDLDGYKEL	S	640			4965	LOC Os12g41920.1	TASGKLIKVNRKYRIAPSSSLSEGR	Y	182
750	LOC Os02g13130.1	DHYSSKRKYDDPSPPPRRTGFSSAP	S	16							
753	LOC Os02g13170.1	KELLLKDVARANSIEVSILKDRLYK	S	419							
756	LOC Os02g14530.1	RQVSNQKAVSLPSSPHRLRSDGSGL	S	480							
759	LOC Os02g14530.1	WSSHAEHKLGSISLTKQERNSGSPG	S	33							
762	LOC Os02g14530.1	TSNMRRGRRRSISFTPEIGDDIVSA	S	401							
765	LOC Os02g14780.1	DEQPKQVIWDGHSGSIGRTATQALS	S	513							
768	LOC Os02g15220.1	VRSVMQRGPLTVSVTHTESFEKEKR	S	245							
771	LOC Os02g15220.1	NPKPPPNPSPSSSPLAQTLASIRRS	S	65							
774	LOC Os02g15310.1	SYSPAPRRRDDYSASPQRKDTHRAK	S	177							
777	LOC Os02g16090.1	QPVFDKISGRVASWRGKNMAVAGRT	S	786							
780	LOC Os02g16890.1	EYYLYGVEVNLGSFRYFKQDDLSVE	S	55							
783	LOC Os02g18660.1	TTAASDDGTLVGSKKGKADNGKLDG	S	447							
786	LOC Os02g18660.1	GDEEGEEVEGDSSESEPEPEPVKKE	S	99							
789	LOC Os02g18660.1	PPQKPSGRGAASSDEEEEEEEDSDT	S	60							
792	LOC Os02g18660.1	GKVSKYNLAPEPSPSSKSGKALSRW	S	191							
795	LOC Os02g19150.1	CELLSSRSAGDCSETDSCASDTDGV	S	196							
798	LOC Os02g19220.1	YGRDMFEDEGGGSFTGVDDLAGAFS	S	71							
801	LOC Os02g19860.1	VAPPSRRPLKRMSSPERWEAKQLIA	S	418							
804	LOC Os02g20570.1	IAKDRPKRNIKPSQRYIEEANIVAY	S	679							
807	LOC Os02g20970.1	DQHSSPERGESESPTKRHLRKKAVH	S	622							
810	LOC Os02g22370.1	NDGDGPELLRSASDGETSTHADSLT	S	396							
813	LOC Os02g24080.1	GEIRGPMLTRDDSFIRKGSHLEQRH	S	217							
816	LOC Os02g24330.1	PPPERRSFGRRGSPVRGEDNGSRPL	S	386							
819	LOC Os02g25060.1	VDVTYKPFKEGDSDVDTSDESGTIE	S	396							
822	LOC Os02g25580.1	TWARPRGSDDGYSSDSVLKHSSSDF	S	587							
825	LOC Os02g26140.1	GKVQVAAAAASVSKTRRCTFSPSRL	S	318							
828	LOC Os02g28980.1	GNMISKLSKLEDSETEGGTTQAPSK	S	610							
831	LOC Os02g29410.1	IVPDKLLNKIRHSVRIIGGNTVVKA	S	355							
834	LOC Os02g30140.1	LSSSLRSLSLSSSPRGRHHRGATTR	S	39							
837	LOC Os02g30230.1	SKNAADDMIRILSGFDNRLSQITSD	S	50							
840	LOC Os02g31220.1	SGGAEEGLYRLSSVGEEIDNLSNLS	S	75							
843	LOC Os02g32350.1	ERLQIWQYGDVESDEEEQAPAARRT	S	970							
846	LOC Os02g32550.1	VARSRSVGCGSRSFSGDFLERISNG	S	278							
849	LOC Os02g33320.1	KHTRGYQQDIKDSRLSGESPVIPLR	S	490							
852	LOC Os02g33320.1	PEGLRSMAVSNVSPLLVSGSPQHNL	S	436							
855	LOC Os02g33320.1	HSPSHSAHSSSKSFQPTQPEGLRSM	S	418							
858	LOC Os02g33770.1	MLDLGNVVKTSPSDEEDVDDGHHHG	S	68							
861	LOC Os02g34080.1	PRIMKASMVRGRSFGTSSLPRGRSF	S	1270							
864	LOC Os02g34560.1	INVERQRSFDDRSLSDVSYSGGGHG	S	48							
867	LOC Os02g34560.1	LNKPRINVERQRSFDDRSLSDVSYS	S	43							
870	LOC Os02g34560.1	LAVGAGGMRRSASHTSLSESDDFDL	S	15							
873	LOC Os02g34570.1	TAGGSDETQEAISPKSLKDAVKPKH	S	373							
876	LOC Os02g34840.1	KRRAAGDDGPSESADDDIVVAQISK	S	30							
879	LOC Os02g35150.1	HDTSDDRWVATNSDVDSDLETQYQR	S	574							
882	LOC Os02g35190.1	KMFMKDQLKTSGSFVLQRFGAFDFA	S	689							
885	LOC Os02g35750.1	MDKAMAFLDLMVSRGCYPDIVSYNT	S	361							
888	LOC Os02g36974.1	AFDEAISELDTLSEESYKDSTLIMQ	S	217							
891	LOC Os02g37130.1	SSKENNGVMWISSPDRLEDPRKPFQ	S	134							
894	LOC Os02g37880.1	KEALLVMEFCEKSLVSAMESRGTGY	S	113							
897	LOC Os02g38050.1	TSLGSQPLDTQNSKSSGARGLKTLS	S	237							
900	LOC Os02g38220.1	SGSGKVTAHLSNSPDESQITESMEQ	S	463							
903	LOC Os02g38480.1	PTAAHSRLMRKGSSDHLVLNIGRES	S	1680							
906	LOC Os02g38840.1	SPSSGRASFSSLSGLKDLELSSESG	S	21							
909	LOC Os02g38920.1	EEDLVSTDFQGDSRSSIFDAKAGIA	S	292							
912	LOC Os02g38920.1	EYKSDVNIVSNASCTTNCLAPLAKV	S	153							
915	LOC Os02g38980.1	SEGAAGSPVRVDSEDSSAPKRKCIS	S	54							
918	LOC Os02g39010.1	NEQDRRFKKHMKSPDPLEEQRMKEQ	S	671							
921	LOC Os02g39550.1	FSRALAVLASASSVSKERQEFLSLV	S	609							
924	LOC Os02g39700.1	STGESEERTTVNSDMEEQKSSDDMT	S	278							
927	LOC Os02g39840.1	QFNNQDKLSSQFSAKAQVGPAPALV	S	176							
930	LOC Os02g39920.1	VVDDGKLSERTISDELPQEPAKLEQ	S	280							
933	LOC Os02g40430.1	SGRQNFSAMRSTSLDLGVDPPSSRD	S	574							
936	LOC Os02g40880.1	DMVRCQINFKRLSLTDIKIDIKRVP	S	54							
939	LOC Os02g42040.1	AASVEKPENEKLSDSESQKVQPHDT	S	824							
942	LOC Os02g42040.1	EGSKDPRAVESESGEETVDEEGGSA	S	974							
945	LOC Os02g42580.1	LLAPLPAASLVRSASLSEILDVKTE	S	335							

948	LOC Os02g43020.1	RPEASESSQPSSSPPSQPQEQPEAK	S	209							
951	LOC Os02g43270.1	VKAAQDGSRGTSSVSPAVASTDVVV	S	319							
954	LOC Os02g43370.1	PEIERCDAGDVESDHDGAAAAAERV	S	18							
957	LOC Os02g44642.1	RMTSPGPSSRVHSGSVSPLHPRAGG	S	335							
960	LOC Os02g44654.1	LEFTTRTLRTAMSRWVSRSIHHRLL	S	148							
963	LOC Os02g44740.1	DVTSLAPSSPPRSPRRSAYYVLSPA	S	22							
966	LOC Os02g44770.1	GLMTGKSGQIAKSGPLEEEEEDPFM	S	334							
969	LOC Os02g44810.1	PPHPKRRKMSSSSDPDQEPTSPSAA	S	22							
972	LOC Os02g45070.1	MEPDTSDSGSMASGAHTRGGGPLPG	S	1041							
975	LOC Os02g45110.1	ARSTRRKTSPGSSPASVAAAPGGLD	S	107							
978	LOC Os02g45110.1	KAAYPDSEVQPPSPPRASAPIDGDQ	S	679							
981	LOC Os02g46090.1	LRSKYFASFRGASQRHDEAGYAPVA	S	21							
984	LOC Os02g46956.1	AEGAMNFMRSTISGDLGSFPRTDAA	S	282							
987	LOC Os02g46962.1	SEVSKKMTAGMSSMSFKMKEIFQGQ	S	33							
990	LOC Os02g47130.1	EIPQADLTGDKTSEEEDLNEVKEGS	S	400							
993	LOC Os02g47150.1	PAAQKKQPAKKASTQLSDDEDDEVL	S	1288							
996	LOC Os02g47220.1	NKLTTNRVPARNSPGHPVLRHSRSL	S	381							
999	LOC Os02g47860.1	KTKLSIDDELVSSPKEALSRVSADK	S	437							
1002	LOC Os02g47900.1	YSAFKKSLRPSGSLKKRTSSGVEKH	S	54							
1005	LOC Os02g48000.1	TVANAMSRQNSLSFTSSVDEARHGS	S	226							
1008	LOC Os02g48010.1	TVEATADDTEGTSKAEEPATGSKGA	S	1059							
1011	LOC Os02g48010.1	FAGATISEQDEDSEAHSESVSLGGQ	S	986							
1014	LOC Os02g48180.1	KTQTDDEINVDGSHSSEDSLVEKKR	S	589							
1017	LOC Os02g48360.1	LKGKAFELLRNNSTSFLIDDIYRNP	S	526							
1020	LOC Os02g48640.1	QGSYGVELVRQDSFGVELVRQDSFA	S	497							
1023	LOC Os02g48730.1	RAEAMSSAVDAISCSKGIAAPPTEE	S	132							
1026	LOC Os02g48810.1	LPEGNLNNDAALSSEKTGNVGPCSS	S	362							
1029	LOC Os02g48810.1	VAIEEKPKSTVISEVCTTKDTEMTT	S	565							
1032	LOC Os02g49070.1	SFDATNAVKSVTSSISSASGKHVAD	S	158							
1035	LOC Os02g49070.1	FLKPSLRITSKGSFDATNAVKSVTS	S	146							
1038	LOC Os02g49080.1	SPAYSWRGGSQGSSSGLIVKKTMKV	S	129							
1041	LOC Os02g49150.1	NIPHVNIDSDSDSEGFVEELPRVNS	S	52							
1044	LOC Os02g49450.1	GLDDDFDKIDGTSGLESDDDDDKEK	S	281							
1047	LOC Os02g50320.1	GKSIGNGPSRRLSLGGADNMSKISP	S	386							
1050	LOC Os02g50550.1	NTPEGGSMRQSHSDGSLDTMARKPA	S	726							
1053	LOC Os02g50550.1	ASYTNDSSGAESSPRTSGQSGEDWR	S	851							
1056	LOC Os02g50620.1	VLEMDTYRYHGHSMSDPGSTYRTRD	S	293							
1059	LOC Os02g50700.1	TMHETKEAVVGESDDEKEKFKQRVE	S	142							
1062	LOC Os02g50840.1	HRLIVRFPNPGRSPARSASGGSFED	S	679							
1065	LOC Os02g50840.1	SQGDKGTTSAGSSPLPEIDSKAKTE	S	928							
1068	LOC Os02g50840.1	SGGSFEDPSFTGSRASSPVADKHEQ	S	697							
1071	LOC Os02g50840.1	EISKSELISSSPSPRNSSANEQGCE	S	844							
1074	LOC Os02g50850.1	EISKSELISSSASPRNSPGNEEGCE	S	761							
1077	LOC Os02g50880.1	KHKAAAENHAPASPVASTAADNPAP	S	29							
1080	LOC Os02g50910.1	KEEKEGGGVVEVSSSDDVDDAKVQE	S	400							
1083	LOC Os02g50960.1	LQSSRNPTPRGSSFNHTDFYSMVGR	S	255							
1086	LOC Os02g51000.1	DVEAKETIVRILSHGLPKRAMDPAT	S	325							
1089	LOC Os02g51490.1	VRNQSNTVLESISETDEDTGAVKQR	S	243							
1092	LOC Os02g52250.1	SKILALTVDAKGSVAFDAVVKQGEN	S	110							
1095	LOC Os02g52250.1	RASGSPPVPVMHSPPRPVTVKDQQD	S	247							
1098	LOC Os02g52390.1	SLGSDPWLSRLPSVNEAIVVPTQVN	S	883							
1101	LOC Os02g52810.1	PSKWEDAEKWILSPVSCDGIGRMSA	S	246							
1104	LOC Os02g53270.1	APAKYDLLPYSPSPPASDEERRDRR	S	111							
1107	LOC Os02g53580.1	VNKRPKGEDTVTSDGEGEAACANKR	S	35							
1110	LOC Os02g54010.1	SDAVGMMDEKVDSDGEDIIDHHVDE	S	493							
1113	LOC Os02g54200.1	GGKLQMPGSQLVSDDDNAESDDEVL	S	466							
1116	LOC Os02g54500.1	DFENASTDSEKLSPRFLSYFQLRSP	S	937							
1119	LOC Os02g54510.1	FDERIPDDFVRHSPKYRHYEVHSPQ	S	252							
1122	LOC Os02g54510.1	HYGVHDPRDFPISPRFQVGAEDFDE	S	230							
1125	LOC Os02g55060.1	ALGKMSKDDADVSGDLSGLSDKELG	S	69							
1128	LOC Os02g56210.1	APVENPVITRTGSDPQTLDSRPGKS	S	289							
1131	LOC Os02g56270.1	PTQDERPMERKPSNQQIASPPPNYE	S	295							
1134	LOC Os02g56480.1	AKNDDRHTTNPVSDHVVVSPVVSPG	S	280							
1137	LOC Os02g56480.1	YDRLHLLRPAPGSGGGSSRGGSTPR	S	137							
1140	LOC Os02g56530.1	LQPLDEFATPPSSPTQFQDAKGKES	S	535							
1143	LOC Os02g56740.1	PTVLDQDDDSDVSAVEDDDYSKFEK	S	557							
1146	LOC Os02g56960.1	VKGIKGRLNRLPSACVGDMVMATVK	S	54							
1149	LOC Os02g57390.1	LSDSSGLGSRRKSFADALQENISRP	S	205							
1152	LOC Os02g57390.1	SGSTSPSLVRVQSLGSSISHNFASA	S	272							
1155	LOC Os02g57500.1	APAAGGERAPSPSPPPPPRLPALKS	S	30							
1158	LOC Os02g57600.1	EHKLKDDLHWIPSFDKGKNLSLVDM	S	120							
1161	LOC Os02g57640.1	SSPESAPKRAPRSSPPPPQQQASSE	S	22							
1164	LOC Os02g57640.1	SPESAPKRAPRSSPPPPQQQASSED	S	23							
1167	LOC Os02g57660.1	DATGSSGLERSESINDSDLPMLERE	S	346							
1170	LOC Os02g57980.1	RADRPRPPRRIVSVGGNGGDRRKLD	S	111							

1173	LOC_Os02g57980.1	AAGRFGRKLRPDSSDEDDEDISTAK	S	237								
1176	LOC_Os02g58090.1	GPKRVDLLKNQKSDKVDVTIQPEEL	S	515								
1179	LOC_Os02g58110.1	TQGERKILDKLRSYQGTPFIEGLMG	S	52								
1182	LOC_Os02g58220.1	SPAKTYSAMGSSSSNGFSEMTTPTS	S	184								
1185	LOC_Os02g58340.1	LEVFVSFRGREPSPEALLRHNGLLP	S	754								
1188	LOC_Os02g58410.1	QLTKDASSLGSSSPIQEQNQKAYYV	S	1902								
1191	LOC_Os02g58410.1	VRGSLDGAIRNVSGNLSWRTSAVTG	S	1423								
1194	LOC_Os02g58440.1	NATDFGSKREEHSGGDEEGSQNDFR	S	534								
1197	LOC_Os02g58570.1	MAQAQWGVDGYGSMKGLIRLRTSPH	S	150								
1200	LOC_Os03g01470.1	GSAEPTARAAAASARRRLVVAVRRQ	S	120								
1203	LOC_Os03g01710.1	SGPNVLQGNTPKSFGNGFGQLSLGT	S	284								
1206	LOC_Os03g01710.1	PTNDDIDSKSDGSDSVANRSVRSSS	S	177								
1209	LOC_Os03g01970.1	SDNAAIDEGRKQSPEEVSGPESGQC	S	605								
1212	LOC_Os03g02010.1	YDTAGNCDRTPHSDGSGDEDFFQEM	S	167								
1215	LOC_Os03g02160.1	SSPDNHDIKCEYSSPTPIPESKELS	S	359								
1218	LOC_Os03g02310.1	MKAGRKNLRRACSEGAVTLGEGESI	S	13								
1221	LOC_Os03g03034.1	SDDPAKKIRLSTSFNVRKETVHNWR	S	111								
1224	LOC_Os03g03460.1	ARRRSQQKTDELSAVESGSNYSSRR	S	223								
1227	LOC_Os03g03460.1	FASPEDSKYKQKSPAESPMEITSET	S	266								
1230	LOC_Os03g03560.1	VEGANTQEVECNSPEMATERFRSDS	S	623								
1233	LOC_Os03g03830.1	YDFGEESVRSPSSAGRSASGSPFVS	S	855								
1236	LOC_Os03g03830.1	SPKSSTNFGRFDSFDDADPFGSSGP	S	1044								
1239	LOC_Os03g03870.1	SNQKSGLTSNEDSGEEHLARTEPVA	S	298								
1242	LOC_Os03g04710.1	HDRSLGGLRRACSDGNLAALGASGD	S	70								
1245	LOC_Os03g04920.1	SSVPIKRLTPSVSNIDNLKNKVSNN	S	878								
1248	LOC_Os03g05260.1	DDDGIGEEYMAPSDDEEAQLPDELF	S	29								
1251	LOC_Os03g05390.1	QLAIKSLRASSMSHEMVEVSTVTDR	S	296								
1254	LOC_Os03g05390.1	RSTSANENLRSRSVNSEADIQLAIK	S	276								
1257	LOC_Os03g05390.1	EVTSHRFTPARMSHVSSLNPDDMDC	S	240								
1260	LOC_Os03g05390.1	MDCISEPIIRSNSVRSTSANENLRS	S	262								
1263	LOC_Os03g05480.1	VEVKEENVEMDDSAICDQYDDKVAS	S	484								
1266	LOC_Os03g06120.1	SEKLQVELQRSQSNMDMGQCEVIQH	S	208								
1269	LOC_Os03g06220.1	ADGGKPPTPEKKSWADVEEEEEAKA	S	14								
1272	LOC_Os03g06710.1	VWGKHRRMEQVASLMRTMEEFQCLP	S	309								
1275	LOC_Os03g06950.1	EVPLQEEHSILRSCDSDLLQTKEKN	S	1064								
1278	LOC_Os03g07580.1	NSSAKGLRLRSLSPLHGSSLKVSRR	S	249								
1281	LOC_Os03g08220.1	ISEEVPRLAASPSSGIKGGGAGERR	S	60								
1284	LOC_Os03g08360.1	VSKAEFIDLARKSGKFDEDSLAFQS	S	150								
1287	LOC_Os03g08430.1	LTEETGRSFIKRSLSDGNILCDNTG	S	609								
1290	LOC_Os03g08820.1	SPHGKKVLQRELSDLELGEFREASL	S	859								
1293	LOC_Os03g08830.1	YTDARHGRLRSLSDLLFRSDASGSS	S	524								
1296	LOC_Os03g10070.1	SLERHPVLARSASAFSGQLAELERA	S	116								
1299	LOC_Os03g10180.1	LGQEFYKDIFAGSESMSPRRGGAAG	S	80								
1302	LOC_Os03g10460.1	AAPKGEIHYFRDSDDEQPDSDEDPD	S	367								
1305	LOC_Os03g10590.1	RFPESRDARRPRSFFQHDERGSAGQ	S	138								
1308	LOC_Os03g10800.1	TRASAAVTIRADSICVGSYESSRSG	S	493								
1311	LOC_Os03g11140.1	RRDLSADETDFCSDDETLIEKLENN	S	546								
1314	LOC_Os03g11220.1	EDDKKKKKHGEGSEGGMGDYLKLAQ	S	348								
1317	LOC_Os03g11240.1	NGQNRSRFARTRSSPELTDPSVEGY	S	505								
1320	LOC_Os03g11290.1	KFRRRSASEFSSSEGLSARRIYKMN	S	663								
1323	LOC_Os03g11500.1	SRTAVLTASPSSSLASRRFGSRWGR	S	126								
1326	LOC_Os03g11550.1	PSAPPSGLFRTSSLPTVAAAEAAKK	S	83								
1329	LOC_Os03g11580.1	NSPLAKRANLLESKSPDGVGQTFRA	S	415								
1332	LOC_Os03g11580.1	NLSPEPLGAKDWSSDMPGDIDNDES	S	873								
1335	LOC_Os03g11580.1	ISKDGFYNDKHDSPVKVATPFDIHP	S	447								
1338	LOC_Os03g11790.1	MKNLQVIFPNSISFGRAELPFSMPN	S	315								
1341	LOC_Os03g11890.1	IHEQATIRNETASDVDEAGQSTSSN	S	377								
1344	LOC_Os03g12010.1	DQAGQAASSPLNSPTRGIVVDISDG	S	442								
1347	LOC_Os03g12300.1	HLKNQLQSQKSASAIVLSQEIQRYE	S	677								
1350	LOC_Os03g12450.1	EDANLTSNRKSLSFVERRRFLYLSK	S	1070								
1353	LOC_Os03g12450.1	NALSHQRSSIHLSPQSKNQTTGALW	S	729								
1356	LOC_Os03g12460.1	NNRDSISVDAILSKNSSTSFLSGES	S	510								
1359	LOC_Os03g13190.1	ISEKDNDAEKENSEEEKNETGEYTA	S	259								
1362	LOC_Os03g13560.1	GEMANGDVSPKSSPAKQSDAVEQKP	S	604								
1365	LOC_Os03g13614.1	SKRKTPLKRSKGSLGNLDVVATKNK	S	148								
1368	LOC_Os03g13720.1	AMEDSNKGIDSPSREDSSSVSHRCR	S	709								
1371	LOC_Os03g13970.1	SSGAENKDHASSSNADSVMAEAEPA	S	264								
1374	LOC_Os03g14260.1	SPAAAPPPTPPESDPRLVEAFVPFL	S	15								
1377	LOC_Os03g14580.1	GKTTAQKKRRASSDTDDDDDDFIVS	S	295								
1380	LOC_Os03g14980.1	PPAITALNGDSRSLVDVKPRIADEP	S	732								
1383	LOC_Os03g15033.1	AIESAHDRIIMQSFSDRKKPKVDLK	S	142								
1386	LOC_Os03g15050.1	SAPTTPIKDGASSPFAAALSEEERQ	S	62								
1389	LOC_Os03g15420.1	MAGVERMLAPSPSTGAKRERLKKSI	S	643								
1392	LOC_Os03g15650.1	SDASSNPVERVQSISDLEKKGAAEL	S	337								
1395	LOC_Os03g15740.1	LRKEMNETDVPISYSGAGNPEKSIR	S	607								

1398	LOC Os03g15810.1	KPNFNYKGLLSESFHSLTSRAEQEA	S	182								
1401	LOC Os03g16369.1	PSPHRRSHIRRKSPPFVRQRSPSPH	S	293								
1404	LOC Os03g16369.1	AKIDDDSESEDGSPFRKDKRKAHGN	S	680								
1407	LOC Os03g16369.1	KKAHDDLSPGEKSPSQHSGKETRRK	S	587								
1410	LOC Os03g16369.1	SPRRRSSLDRHWSPSPGRRRPRSPS	S	336								
1413	LOC Os03g16369.1	DSSLKGTDKHLPSQVRTDSSGEEEG	S	536								
1416	LOC Os03g16369.1	LKLKHAETVDMASDLEKDRESDSDA	S	789								
1419	LOC Os03g16800.1	EEFMRDRAKRPKSPPREPEPEPVKE	S	363								
1422	LOC Os03g17020.1	ESGAESGRQPPRSKSTQDMYTRQQL	S	191								
1425	LOC Os03g17170.1	ERLEARDFKHLGSGSGLGLVKPAPK	S	291								
1428	LOC Os03g17180.1	IQRQSSKNSEPDSPISPLLTSDPKN	S	777								
1431	LOC Os03g17310.1	KGGQEEGKRRDGSDASGSEPAAAAF	S	15								
1434	LOC Os03g17490.1	KSRAERFGLASSSADEDAKKKARLE	S	113								
1437	LOC Os03g17950.1	TPSEPIVPIRRSSFSYTPSRSLGQS	S	171								
1440	LOC Os03g18070.1	WALRVSAPTRLTSVVEEDNRGEEVV	S	64								
1443	LOC Os03g18454.1	TDCYKGERGHQLSLKYGRQTDALQP	S	165								
1446	LOC Os03g19290.1	GAVSDSTGARKRSFPDLNGGNSSKS	S	82								
1449	LOC Os03g19520.1	EEENKHVWARKNSAYFSKLVFSFTD	S	587								
1452	LOC Os03g19530.1	ALAADAMHAGYDSDEEVYAAAKAVD	S	131								
1455	LOC Os03g19590.1	HAVFEQSGASGRSFDYTQSLRASPT	S	84								
1458	LOC Os03g19870.1	KSEELADSIGSLSIGPERTNFKKKP	S	41								
1461	LOC Os03g19990.1	TADPMSLPLFLHSVRPYGDIFTVDI	S	640								
1464	LOC Os03g20530.1	VASEKLASENSVSFSRKQENSDSFS	S	299								
1467	LOC Os03g20860.1	ASTSPWPLRKLQSFTPGLCSQYKAY	S	29								
1470	LOC Os03g21140.1	DLHQLGARMLQHSNSTNEMMLRRKL	S	484								
1473	LOC Os03g21160.1	HQLGARMLQHSNSANELMLRRKLEE	S	479								
1476	LOC Os03g21160.1	PSPSSWAQAQPFSRSNSMGNGGAAD	S	108								
1479	LOC Os03g21160.1	AFPFTPTGSLPASPSATSKCLSLLL	S	299								
1482	LOC Os03g21350.1	NTPSNQALSLQRSPSVRIEILRSLF	S	385								
1485	LOC Os03g21530.1	ALKAKFDAEYDGSDLSGEEVDNDTK	S	723								
1488	LOC Os03g21800.1	SSPPRPKHRHSSSVDGSGFFAAARK	S	92								
1491	LOC Os03g22340.1	TVSRDKTKVTVTSDGPFSKRYLKYL	S	77								
1494	LOC Os03g22350.1	SDYVTKAGYGSESEVDDEAATVSLA	S	265								
1497	LOC Os03g22580.1	EAKGSPVESVSSSPLKNSNTDKISK	S	455								
1500	LOC Os03g22730.1	TNAESLDFSEILSDDEVEAQLKEAA	S	225								
1503	LOC Os03g22880.1	TPKKKKKKNREVSEDAEPKTATEGK	S	528								
1506	LOC Os03g23970.1	EELRGGSAYGADSLCIGVARLGSDD	S	218								
1509	LOC Os03g24180.1	PYGSSRPLTRKLSLDASSSDSRGME	S	778								
1512	LOC Os03g24339.1	ELNHRNLKDKSTSPDSMPMKELIAV	S	804								
1515	LOC Os03g24890.1	RSASPDELERHVSPSNNGHGPPVDG	S	229								
1518	LOC Os03g24890.1	YRDHRDDYSPGESLSPHGQDKRHHR	S	201								
1521	LOC Os03g24890.1	VSRSPRSRYHSYSPSPSPARRDYRD	S	179								
1524	LOC Os03g24890.1	DKRHHRSNGRSASPDELERHVSPSN	S	220								
1527	LOC Os03g25070.1	LCGSRPFWARTESGIFRAVLKADPS	S	357								
1530	LOC Os03g25430.1	RPTSDKHGRDADSDDDALPDAAAAV	S	193								
1533	LOC Os03g25600.1	AAAMAGLQRPSKSFSETTFRTISGA	S	113								
1536	LOC Os03g25720.1	KRNLEADAAADHSDDDDARRVALEE	S	19								
1539	LOC Os03g25970.1	GGYGAYGGYSGRSGSSGLSGPSSFS	S	523								
1542	LOC Os03g26030.1	ARVWSSLLARLPSLSDYSRLLSVGR	S	45								
1545	LOC Os03g26630.1	KLEPPPPAAAPISPATTPKEPPFQQ	S	613								
1548	LOC Os03g26630.1	IAPKPNDQVPEVSPDLGSPIKCESI	S	278								
1551	LOC Os03g26630.1	TAELSSDHVKEVSLPDTVVDNSSVD	S	412								
1554	LOC Os03g27080.1	EGRLQRALDRVRSMTQYPEAREQYR	S	483								
1557	LOC Os03g27460.1	GGRGGYGGRGGGSAGRQPQNGSARG	S	273								
1560	LOC Os03g27800.1	HVANPWTAPPIRSPEQKGSYKEDDA	S	721								
1563	LOC Os03g27840.1	SDLKVAGISPPSSPIRVGIPNRSSG	S	364								
1566	LOC Os03g27840.1	VSYRDPYREQRRSPSYDSYSRGRRS	S	293								
1569	LOC Os03g27930.1	RTKKKKRVRRSKSYHSCQDLAGNIS	S	454								
1572	LOC Os03g28330.1	FNASFPRPSLSKSIGNGVQFLNRHL	S	157								
1575	LOC Os03g29750.1	YQHPLTEDMEVLSPGVFGEHRTNDM	S	436								
1578	LOC Os03g30570.1	SPAAPLPLLRTSSLPTETEEERWRR	S	91								
1581	LOC Os03g30790.1	PPSPSPSLSLSRSQQQTPRGRQPPP	S	29								
1584	LOC Os03g30790.1	PSLHRTPPPPSPSPSLSLSRSQQQT	S	21								
1587	LOC Os03g31730.1	DVHVTTDVSPGISPKGDIPSDCATF	S	605								
1590	LOC Os03g33012.1	KDGGALLNEADVSPEKEDASTRSEQ	S	276								
1593	LOC Os03g35340.1	RQESNIPSERKFSVSSPEKVMFPDH	S	1717								
1596	LOC Os03g37330.1	AVKARLKRKRIKSDLAEDERQERMI	S	161								
1599	LOC Os03g39020.1	PKIHNKARKSITSPGLSPSKLSVSD	S	629								
1602	LOC Os03g39380.1	PVSNAPRLRHLPSLRRTFSLYDQIN	S	35								
1605	LOC Os03g40010.1	ADSGHSDDQGSNSRKRKRGSTPKSS	S	456								
1608	LOC Os03g40010.1	SKKFDDDDDITPSKKRNKALEYDTD	S	481								
1611	LOC Os03g40010.1	NDNEEGDKNANISDEHLKKDGGKDG	S	227								
1614	LOC Os03g40110.1	DDYSDEEGHMILSKNRIPLQSGDVP	S	189								
1617	LOC Os03g40110.1	ERQKRMLGTAESSDEDDDDDEEGDR	S	329								
1620	LOC Os03g43020.1	ENLDDAGNELILSDEDVVRFQIGEV	S	56								

1623	LOC Os03g43390.1	DAATASASATPSSPEKKPRVVPDAD	S	88							
1626	LOC Os03g43800.1	HGFQVDADFPEGSLGSREAETGDYP	S	379							
1629	LOC Os03g43800.1	QTPGRSGDRMFLSPVKSTDRKNADS	S	347							
1632	LOC Os03g44310.1	PRHADHLAPGSPSPAASPSELGDDE	S	100							
1635	LOC Os03g44310.1	ASPSELGDDESWSRAPSAAELESNN	S	115							
1638	LOC Os03g44310.1	SWSRAPSAAELESNNNDLAEIRNDN	S	125							
1641	LOC Os03g44500.1	TGGALGGMVRQLSIDQFENEGRRVS	S	612							
1644	LOC Os03g44800.1	LPEGTVQERNQCSDDDDELPESPAA	S	295							
1647	LOC Os03g44900.1	YVERNQEDFDEFSDVEELYSTLPME	S	217							
1650	LOC Os03g45300.1	RGKNEVSPVAAGSGEDSGRGRRTRA	S	137							
1653	LOC Os03g46040.1	PQAPLVPILRTPSGEVVYGITDDPV	S	171							
1656	LOC Os03g46340.1	EERSESCSGTDNSPPLTKHVDASDD	S	403							
1659	LOC Os03g46480.1	DADEDAEPEQPSSPSVKKAEEKGIV	S	242							
1662	LOC Os03g46600.1	DGAVKSTTGRLPSPSLLGRYPSLEE	S	48							
1665	LOC Os03g46710.1	GDGMQTKFSRRESLDPELPGASSSS	S	174							
1668	LOC Os03g46920.1	SSSLSSGYGRRKSAKEDESLSLDPV	S	706							
1671	LOC Os03g46920.1	EPVVEKTLSGSGSSEGHLRVKGSQL	S	660							
1674	LOC Os03g47800.1	PARYDGGSYRDRSGPYDRPSRGGRF	S	291							
1677	LOC Os03g47930.1	RILGEENNKGMESPDRDQIDRYITS	S	574							
1680	LOC Os03g48600.1	ESLIRADNNALNSFRILEEDDIIMP	S	306							
1683	LOC Os03g49170.1	NGEELGKLRRSSSFELRSGGDDPDL	S	678							
1686	LOC Os03g49170.1	GSSTGNHTSRTKSLNPSNLDDLFSA	S	510							
1689	LOC Os03g49640.1	SADRSLGLRPSRSLDLAVGGGGTDI	S	93							
1692	LOC Os03g49770.1	SSFKSSGSDFQRSEAGSDDEDDYPM	S	381							
1695	LOC Os03g49770.1	IYFDTRDFLSSSSFKSSGSDFQRSE	S	370							
1698	LOC Os03g50120.1	RWRNTRGADRSRSFDDSFDREDDGS	S	415							
1701	LOC Os03g50300.1	PKKYAEDVESGSSDVETKYKKMYED	S	566							
1704	LOC Os03g50330.1	FYDGDDPDHLYGSDDGQPRKRSEDI	S	92							
1707	LOC Os03g50390.1	PSPKLSDWSRRGSADMLDYLQEADS	S	667							
1710	LOC Os03g50390.1	STVMDEEDDVVRSLRASPVHPVKDR	S	835							
1713	LOC Os03g50390.1	SYTQKDLPNAVGSPDVAKVSNSSIN	S	637							
1716	LOC Os03g50810.1	YKRKQKPFGRVQSPHAMVVHPRHSG	S	538							
1719	LOC Os03g51020.1	PDDPFVEDDQHGSVRSIGVGISSDA	S	550							
1722	LOC Os03g51020.1	HGSVRSIGVGISSDAADIGSEVRES	S	560							
1725	LOC Os03g51020.1	VAEPKEDDEQDGSEAGSNAGPRGAA	S	56							
1728	LOC Os03g51030.1	TLNDDIKPTRAASLDNQVGDLKLDG	S	601							
1731	LOC Os03g51250.1	AAPEKTSRAGSFSFKSGPPKLELQM	S	327							
1734	LOC Os03g51520.1	SGSSKDNQKRLESGEEVLQDRRSRR	S	796							
1737	LOC Os03g51520.1	ISRAEQPTGHNSSDQDELRTRNSDN	S	767							
1740	LOC Os03g51790.1	KRSSPTASPPADSDDEGSPRGLSLL	S	54							
1743	LOC Os03g52310.1	TDNDGEGTVERPSKRRYRYTGYANA	S	149							
1746	LOC Os03g52700.1	GKGVSRWRSDESSEDEDDKRTSGYM	S	886							
1749	LOC Os03g52970.1	EGVRSIVLKPSESLDEGRFTRIAGA	S	28							
1752	LOC Os03g53280.1	DLTLPKFGDDMSSIGSHGDLDDFSA	S	1524							
1755	LOC Os03g53280.1	NMNRTASYASGISPESSLDDLTLPK	S	1505							
1758	LOC Os03g53630.1	RSGGVVPKKQEDSSESEEPIILRKN	S	457							
1761	LOC Os03g53630.1	ANGDELSMGLSRSASGRESIEQEDT	S	63							
1764	LOC Os03g53630.1	LTDCTNQKKGIDSPVNETKGDSTPD	S	125							
1767	LOC Os03g53880.1	FLIQKNILKRPRSPDGDHGLAVGNF	S	91							
1770	LOC Os03g53970.1	TPRSCFDDEYCSSPRTPVLNEETAF	S	181							
1773	LOC Os03g54780.1	QQQNSRRKPSVSSVPESVTREDPST	S	247							
1776	LOC Os03g55070.1	FDWDHPMHLQPTSPTAFKQVSVVWD	S	393							
1779	LOC Os03g55330.1	GTVARRQAGPADSPAESYLESRVSF	S	119							
1782	LOC Os03g55570.1	WPLNSDGPVTSASPERSTPKEKPQK	S	222							
1785	LOC Os03g55704.1	HVVSFDAAVRSVSQELEHGKDFTEE	S	700							
1788	LOC Os03g56460.1	DQWGVELGKSLASQVRKSLHASRVE	S	520							
1791	LOC Os03g57160.1	SGSKIYGSTKADSVESGEKIGGHAV	S	773							
1794	LOC Os03g57160.1	AQNHAMRSDSAASLYMDDHVTPNHK	S	917							
1797	LOC Os03g57160.1	VDKPQDRLQHADSIEGSVIDRDGEE	S	497							
1800	LOC Os03g57160.1	EENSTHSAKNADSVEPFFPRFAEHQ	S	293							
1803	LOC Os03g57430.1	SVKHSLDPELNGSPSSKDKLRKDGS	S	325							
1806	LOC Os03g57430.1	SRTIGSDLTDGGSPIRDSPSSKRLL	S	204							
1809	LOC Os03g57430.1	DGKISSDNVLAASPQSNKMAEKIFE	S	246							
1812	LOC Os03g57854.1	SSFFRSELFEHESDEEKNEVDTSNE	S	252							
1815	LOC Os03g58480.1	AKSPKFTRRRSCSDAPPTPEAANTT	S	308							
1818	LOC Os03g58590.1	LEHPDSIEQDKESEDEIDAKEKDAT	S	415							
1821	LOC Os03g58810.1	PPKVNIDLESSASKHRRKMRKVDGG	S	553							
1824	LOC Os03g59250.1	TEPFVSDNRHSMSLSPEKDRYYSTR	S	92							
1827	LOC Os03g59340.1	YDSGEIGHPKYDSGEIPRIYIPSLT	S	135							
1830	LOC Os03g59480.1	GSKDLDKELLENSQDNLEDKAQSSD	S	370							
1833	LOC Os03g59680.1	KRIPKKRTLDGDSGDEDGGSGYLVK	S	223							
1836	LOC Os03g60110.1	LYSPGFSPARNLSPQIRSNPTDVDS	S	16							
1839	LOC Os03g60240.1	RSLVEQNAMLSNSDRMQSVQEENLL	S	366							
1842	LOC Os03g60530.1	FFSIARDIKQRLSETDSKPEDRTIK	S	182							
1845	LOC Os03g61160.1	PLASPRAAANVVSPLASPRAAGDIS	S	71							

1848	LOC Os03g61160.1	SPLASPRAAGDISPLAASPGRAQQE	S	83							
1851	LOC Os03g61220.1	EDRFRRGGRSFRSDNDSWSDDDFGG	S	685							
1854	LOC Os03g61220.1	RRGGRSFRSDNDSWSDDDFGGGRRS	S	689							
1857	LOC Os03g61890.1	LSARVASPANLFSLRFRMPPLPHFL	S	811							
1860	LOC Os03g61930.1	KSDLQSKTKEHASDGTSSVLEPEVE	S	496							
1863	LOC Os03g61930.1	TNGTSVPEPKTISENGSSVETQDKV	S	143							
1866	LOC Os03g63140.1	YDNAVRKTGIEKSPVHPRHQARAAN	S	117							
1869	LOC Os03g63320.1	EEAEHYYDDADQSDSAAAAAARARV	S	149							
1872	LOC Os03g63670.1	NPSQNEDKDEDESEEEEEEEEGPVI	S	216							
1875	LOC Os03g63670.1	HIEGELSTDESDSESSAYLSSRDEL	S	621							
1878	LOC Os03g63940.1	LTRDFDQINTILSPSTPGSRMNMDV	S	114							
1881	LOC Os03g64340.1	LSTGKLTVTGTVSGDKLAEYIHRRT	S	198							
1884	LOC Os04g01490.1	LRQTVKSLLPVGSFKSSVKFFNARI	S	139							
1887	LOC Os04g01490.1	AVDKAGHMYRSQSLPMNMKKLNNGK	S	187							
1890	LOC Os04g02000.1	DTSSTLGTKRLASEELANEWDNKRL	S	231							
1893	LOC Os04g02870.1	RQRSPVYGSYDRSPVNDRYRSRSPV	S	219							
1896	LOC Os04g02870.1	PDYGRQRSPVYGSYDRSPVNDRYRS	S	215							
1899	LOC Os04g04680.1	GTIARTRTKPVLSLSKRRPQLEHEP	S	37							
1902	LOC Os04g06770.1	MSDASSSQGGHTSVGSVPVPELPRL	S	888							
1905	LOC Os04g08450.1	MMETELCSSRVLSPPRYESGDEELS	S	14							
1908	LOC Os04g08740.1	PVAAIRMPMLKASNFKGGTPEVMET	S	291							
1911	LOC Os04g14580.1	RRGARDRASTLSSQRPLGRRVQGEE	S	21							
1914	LOC Os04g17700.1	LPRALRPAGPLPSDVEDSDDSNAGD	S	51							
1917	LOC Os04g18010.1	EASESNVKRVPLSPQGSADHHNASD	S	356							
1920	LOC Os04g21220.1	LLLTFRPGPSQRSLSLFAGSVWGFQ	S	292							
1923	LOC Os04g24210.1	KFVSQLGVMTSTSRPMDLYCDNSGA	S	622							
1926	LOC Os04g28040.1	RSECDILQGAPRSPKQNVGSAQEQR	S	482							
1929	LOC Os04g28180.1	RTLDSHIEEQFGSGRLLACISSRPG	S	177							
1932	LOC Os04g28460.1	CGVAVYTGNQTKSGMSRGTAEPKLT	S	238							
1935	LOC Os04g31090.1	VEDLWEVAEPQLSPSEKLNSCFEDI	S	111							
1938	LOC Os04g31330.1	DASGLFCSLRRLSLESMDDLDTSSE	S	191							
1941	LOC Os04g32920.1	ATATASRLKRHDSLFGDAEKVSGGK	S	31							
1944	LOC Os04g32950.1	KKPKSTESEAAGSSGGKEDEKEEEE	S	510							
1947	LOC Os04g33110.1	CRLHRSSSHGHPSSSPTAAAAPAAA	S	84							
1950	LOC Os04g33300.1	SSSPASSKALVASDDDNKPRQPQQH	S	20							
1953	LOC Os04g35080.1	ERRGGKAPGQVKSGEIPPVEAGDSS	S	488							
1956	LOC Os04g35170.1	SSFKICDLNLVGSPEVAELRNDPGL	S	558							
1959	LOC Os04g35700.1	PRKSLDLVDLSASPSVTPRAREKAR	S	43							
1962	LOC Os04g35700.1	ISVVRTNMSLPVSPCSSPLRQFKQS	S	638							
1965	LOC Os04g35800.1	SRSRSRSRSRGRSASPRYPDGKRRR	S	88							
1968	LOC Os04g36790.1	NTQMELSQVKDHSSSPANAAAPPGA	S	324							
1971	LOC Os04g36890.1	LVVRTPPAESLESEDEDGFPVSFSE	S	231							
1974	LOC Os04g37540.1	SNPASPVLGRSYSVEPLRKPIVNQK	S	382							
1977	LOC Os04g38090.1	ESVTNWKQPKTVSEIHRFLGLAGYY	S	535							
1980	LOC Os04g38570.1	SLSLRSGSLRNLSYSYSTGADGRIE	S	650							
1983	LOC Os04g38620.1	FEDTKLTKTYSFSDDEAVKVKATSI	S	143							
1986	LOC Os04g39040.1	RLEDYQAPLRPPSPFQGVGRTLGGG	S	196							
1989	LOC Os04g39040.1	TIRTLADISRGPSGFPGGGGGGGSD	S	35							
1992	LOC Os04g39240.1	SLRSDSSTTSTRSFAFPVLQTEWNS	S	623							
1995	LOC Os04g39629.1	YMYDREAAKGTDSASDIDRHDDTGA	S	357							
1998	LOC Os04g40080.1	LEEERKSIAAAESPQAPTTPSAWLA	S	401							
2001	LOC Os04g40400.1	LDDKVRFGQRPGSGAGRVSAVPPAI	S	463							
2004	LOC Os04g40430.1	REPKPEIDDSDNSDDPDFWVPPVGS	S	188							
2007	LOC Os04g40430.1	WDDDDGKDRWTSSPVKDKDDAAEDE	S	214							
2010	LOC Os04g40720.1	CRDTSMEDRGKSSEGCPGAEVHVDK	S	128							
2013	LOC Os04g40720.1	DKGTGTIFNDASSDGSDDELGIGSE	S	1300							
2016	LOC Os04g40720.1	KYPAAIHSDAPISSPSHNKARGEKL	S	748							
2019	LOC Os04g40720.1	QQKKRKKAKKVSSVDMESLDISGEK	S	547							
2022	LOC Os04g41030.1	HSLKSIGQGRDVSEQFDEFPLAYNK	S	604							
2025	LOC Os04g41030.1	KGGRRRRRGGSESGGGSAEDGSWWA	S	267							
2028	LOC Os04g41100.1	GESVVAASASSPSKKRKFSPIIWDR	S	134							
2031	LOC Os04g41100.1	PHRKKKSASPADSAELGQRKKALSP	S	259							
2034	LOC Os04g41100.1	KALSPELGEVVASDISGGRTMSRSS	S	279							
2037	LOC Os04g41100.1	SPMDVEPAVASESPEQLQEHAESRV	S	205							
2040	LOC Os04g41160.1	PSPPEPALPRELSLGDLRAVSMLGR	S	20							
2043	LOC Os04g42010.1	KEAAAQSVKKGESPSENADDPISRE	S	299							
2046	LOC Os04g42140.1	LDNDNWEVPRSKSMPRGDSLRNQGP	S	529							
2049	LOC Os04g42320.1	NNNEDILVSRELSPETDDGDNKLPP	S	418							
2052	LOC Os04g42320.1	TRRLAKDDDIKSSFKKTGEGESSKK	S	504							
2055	LOC Os04g42320.1	DDNQAKDTDDLISGAKEIPEPITTE	S	363							
2058	LOC Os04g42600.1	DMGGFPVRDAGLSPAPIGTLTSALA	S	557							
2061	LOC Os04g43140.1	AESMGLLLETSDSEEERVQGHKQRK	S	690							
2064	LOC Os04g44320.1	KKNSTLPVSNNGSPMVATEAISFDD	S	394							
2067	LOC Os04g44800.1	AAAAVAARSRSKSIEADEEERSKGR	S	27							
2070	LOC Os04g45190.1	QPPRPPSPVREGSEEGSSPRASLSE	S	141							

2073	LOC Os04g45190.1	ATRAHKDAEDVTSDSETSALISRLT	S	186								
2076	LOC Os04g45580.1	SSKVLGRSRSCRSLTGSSLFEDLEK	S	636								
2079	LOC Os04g45800.1	SRQKHLVFDNKDSKKAKNNEQKNDV	S	62								
2082	LOC Os04g46320.1	SSSPNARDIHLPSSFKDLVLERKIS	S	306								
2085	LOC Os04g46892.1	PPPTTPTTPRSPSVSNLTEIRRRRI	S	177								
2088	LOC Os04g47700.1	CANFAPVSEKRNSSIKDDENIELDI	S	613								
2091	LOC Os04g47912.1	TNPCEAYCGRSTSSLDEFEVRSAMA	S	206								
2094	LOC Os04g48290.1	KQLTGAGAANMESDDRVAADEKSRL	S	532								
2097	LOC Os04g49450.1	GKRVMVNDLHQMSAPDAGNLQTVAD	S	265								
2100	LOC Os04g49520.1	HNIMMNGYAKQGSLSDAEELFDRMP	S	86								
2103	LOC Os04g50204.1	GSHKEESNVKGDSEDDEPVMVVAKM	S	674								
2106	LOC Os04g51060.1	DAELLQRYAKRQSPGSDACDPYSIQ	S	364								
2109	LOC Os04g51080.1	VATTDGGGADSFSDFARAPSRSYQH	S	84								
2112	LOC Os04g51120.1	VETAAKDPFDFSSSNLGKTPLADPK	S	447								
2115	LOC Os04g51120.1	FGSNYSSGERYGSFSGTREGDSYGD	S	191								
2118	LOC Os04g51700.1	LDADYISKMEEDSSDRDQGAHPTAP	S	960								
2121	LOC Os04g51700.1	QHAKIDYGQSEESDPGETGQDDQRL	S	936								
2124	LOC Os04g51710.1	GPLNSSGAQRKVSGPLDSAASKKTR	S	177								
2127	LOC Os04g51710.1	GPLNKHGEPVKRSSGPQSGGVTPMA	S	128								
2130	LOC Os04g51710.1	KMFELHAVKSRKSGPLSNAPSRNAS	S	62								
2133	LOC Os04g52050.1	PDIDRIINSICPSSSPRHMTPEKVS	S	520								
2136	LOC Os04g52074.1	DNADATTPALNHSMQQKERIPQCYS	S	424								
2139	LOC Os04g52120.1	IPNQVHSEKRMSSFRLKLPTPELER	S	224								
2142	LOC Os04g52200.1	TLEGSTVQEAGNSDNKEVENLDSQL	S	331								
2145	LOC Os04g52540.1	AARSSGTVERIASTEVVRVQPPAPP	S	169								
2148	LOC Os04g52570.1	PGSTPKFLSGPTSPTKSLSEVHSTF	S	205								
2151	LOC Os04g52920.1	IPSTPVGGRSTASPGDDNTDDGPYF	S	342								
2154	LOC Os04g52960.1	SSSSDDSSDESLSDDEPVKKPAAPL	S	151								
2157	LOC Os04g52960.1	GRDNSSFKKPAQSSGNTIFIKGFDT	S	546								
2160	LOC Os04g52960.1	KKSAVEVAPTSVSVSEGKSGKKGKR	S	18								
2163	LOC Os04g53540.1	SDPLGGDEFESKSGSENVDGVSVDD	S	84								
2166	LOC Os04g53670.1	SPRKLSRQNSFGSIGTPRTPNLHST	S	1130								
2169	LOC Os04g54340.1	RSVTAQSNLNSFSDDEDTREMLLGA	S	539								
2172	LOC Os04g54820.1	SSEPSSSSLLNKSHEVSDDDFDLDA	S	134								
2175	LOC Os04g55040.1	AKESMGEAMRASSFSLAEAKYVAGD	S	67								
2178	LOC Os04g55920.1	RLVSKAPYPTKSSEGMEASGMEVTA	S	200								
2181	LOC Os04g56140.1	KAPVAEPEIHMESDINSASELRTKD	S	460								
2184	LOC Os04g56140.1	DTGPHIHASLNGSTKKTSANGLRDQ	S	484								
2187	LOC Os04g56160.1	TLFNDKSSYRELSEIAEQAKRRAEI	S	906								
2190	LOC Os04g56440.1	SLLDAIARLDDASDSESEDMAVGAQ	S	373								
2193	LOC Os04g56530.1	HVSGQLDYVRHLSGLERHESILPLL	S	916								
2196	LOC Os04g56530.1	SLLEKEPPSRHVSGQLDYVRHLSGL	S	906								
2199	LOC Os04g56630.1	SSADHSDGTGLSSPKGALLGLASYD	S	585								
2202	LOC Os04g56720.1	SNKRKKTKDVSESEEDDDEEDDSDD	S	467								
2205	LOC Os04g56740.1	ACAGGGSDPSAGSDGDADDHVDENK	S	25								
2208	LOC Os04g56740.1	GGKFQDSLICSSSEKLLNNGFALKL	S	338								
2211	LOC Os04g56740.1	TESARAKASASVSPKLSPDVQDNNP	S	832								
2214	LOC Os04g56740.1	DENKAISDKSRWSFRRRSTRHRVLK	S	46								
2217	LOC Os04g57300.1	LLLTRNSINRSCSTPCLCPVSKDFE	S	102								
2220	LOC Os04g57600.1	LRRNTDCVYFLASPLTCKKGNECDF	S	43								
2223	LOC Os04g57600.1	SRLQGRIKLPGRSPDRVDTRSEKER	S	460								
2226	LOC Os04g57600.1	REAAWANGTSACSPKQDKSAEVSHR	S	583								
2229	LOC Os04g58280.1	QKQVAHAPAELNSPRSSAAKPKNPD	S	18								
2232	LOC Os04g58620.1	GSSLGYGYSRVMSISKSKTVTSDDE	S	1132								
2235	LOC Os04g59320.1	KCGVHRNELIPHSGDMRSLHSSSST	S	638								
2238	LOC Os04g59330.1	KTDSEVTSLAPSSPPRSPTSRGGRP	S	16								
2241	LOC Os04g59400.1	SDEEPLHRQSQFSDAEEGMEGPISP	S	188								
2244	LOC Os04g59460.1	PSSKKKSLSFSSSFSKRTQPSHLFS	S	1266								
2247	LOC Os04g59494.1	LKPRFHASRLHGSETGEDDDDDDAV	S	371								
2250	LOC Os05g01040.1	YSAGSPKLKACMSDISMGNSRFFTY	S	46								
2253	LOC Os05g01060.1	DIVPQQAYSHVQSPMSSPVRARRDW	S	385								
2256	LOC Os05g01540.1	SPPPPPPPPRKASPPPESTVLHVDH	S	106								
2259	LOC Os05g01910.1	GSGFASPRIGSRSPGGTLSSRQNLV	S	365								
2262	LOC Os05g02240.1	RRRLHHRKLHGPSAPSSPSAAATAG	S	453								
2265	LOC Os05g02260.1	NPKDVPTYERSVSLPPTLKIISAMK	S	112								
2268	LOC Os05g02500.1	EEPSKSGLGSIRSPSKTSSIAERRG	S	575								
2271	LOC Os05g02650.1	SDGKDLGDNDTSSIGASSDKGPKKK	S	435								
2274	LOC Os05g03060.1	TSESFSTRSSSLSPSLRNSNDHAPT	S	430								
2277	LOC Os05g03120.1	LKKAHNKDDVDGSFGSPDHGEAVEK	S	301								
2280	LOC Os05g03120.1	ASSPFSDEPEEESPKRRNSGVLRRM	S	582								
2283	LOC Os05g03120.1	SMIGGKLASSPFSDEPEEESPKRRN	S	575								
2286	LOC Os05g03190.1	ESNLDRASAKSASLNLGEGEITKAF	S	288								
2289	LOC Os05g03430.1	IQCAPDLATRSHSGSDFSFRPIEEA	S	94								
2292	LOC Os05g03630.1	HPDKNRSPHADDSLFKQVSEAYDVL	S	44								
2295	LOC Os05g03760.1	TAAEKAIRLLLKSPAVSPSSSPKKS	S	166								

2298	LOC Os05g03760.1	KAIRLLLKSPAVSPSSSPKKSASPP	S	170							
2301	LOC Os05g03780.1	PPQIAEVRMDISSSTSVAAGNKVCR	S	20							
2304	LOC Os05g05220.1	SRRGKKAWRANISTDDIEDFFEKQT	S	20							
2307	LOC Os05g05240.1	HPLPPPVNSPESSPERAMDATWMQE	S	853							
2310	LOC Os05g05300.1	EIKEPHDQQSEDSDQEELDRFIAEI	S	334							
2313	LOC Os05g05300.1	SATASGRSVFRESEDDELDTEDDEL	S	493							
2316	LOC Os05g05700.1	GTALVKQAEDSASNKKPEKKDMVGM	S	327							
2319	LOC Os05g05950.1	GIRHGRQTNRAFSLDNARKKAMLLE	S	646							
2322	LOC Os05g05950.1	KQIMKELGEGSASVSPVSGLSSSRE	S	469							
2325	LOC Os05g06630.1	SKPDSDIAEHDDSPKSSSGRLTRSR	S	39							
2328	LOC Os05g07000.1	RRYASPSYGRDRSPGGNALPANGRS	S	246							
2331	LOC Os05g07000.1	ALPANGRSHNLTSDGMNPSPRERDD	S	263							
2334	LOC Os05g07130.1	RGVGSTKFGKSSSACSLASGLNFGT	S	330							
2337	LOC Os05g07680.1	RCHCNFGANSELSEHEEKQQVSDVE	S	217							
2340	LOC Os05g07980.1	PAQDRWRRKRKVSFPPRHALSPPIF	S	586							
2343	LOC Os05g08600.1	RRPPSVKASLSVSFGQRAPHRASTR	S	207							
2346	LOC Os05g08970.1	LERIRNQTGDDESDEEDEDFVADKD	S	480							
2349	LOC Os05g09630.1	QDLVHLGVARTSSGFLGGGTSTSTG	S	376							
2352	LOC Os05g10770.1	DYSRFEHISDEDSDVEIVEKDVSST	S	102							
2355	LOC Os05g11770.1	IEPSLFFPKQYFSDTEEFEDVIYPK	S	280							
2358	LOC Os05g11770.1	VSSANKDAIVCLSNHDEKNESPPAD	S	640							
2361	LOC Os05g11980.1	ISQKQEALQDTYSGDEIIDSMHRSG	S	1085							
2364	LOC Os05g13520.1	ADFDDVAAAMKGSMKLRAEADFDPL	S	309							
2367	LOC Os05g18770.1	RRRTVVVARKKASPELLADGGFNGT	S	42							
2370	LOC Os05g22870.1	GGPRFLGQDDADSDADEEDEEEWRW	S	69							
2373	LOC Os05g22990.1	WLTSTKVSEPTGSPAKDSPVGKEKA	S	717							
2376	LOC Os05g23320.1	IEAVHLDKNGQASPDTSVVIRRTPK	S	614							
2379	LOC Os05g23610.1	EFLKVKELMRSGSLIDDEADEDDRG	S	129							
2382	LOC Os05g23610.1	KTPYHPMVDDDGSLSPTRPFDKCLD	S	42							
2385	LOC Os05g24160.1	TGCVALVLCLNISVDPPDVIKISPC	S	376							
2388	LOC Os05g27820.1	SSLMEATALLRSSSFKEDSYVASAL	S	45							
2391	LOC Os05g28190.1	NKGQSSSPTPLFSFKNLSSGQNAFT	S	254							
2394	LOC Os05g28190.1	EEQKDADNKGQSSSPTPLFSFKNLS	S	247							
2397	LOC Os05g28190.1	GSSEVAETEKDGSDLKGSDEKAKSS	S	138							
2400	LOC Os05g28190.1	DALPVTDKSAGSSEVAETEKDGSDL	S	128							
2403	LOC Os05g28860.1	LFKGTTGGLSGLSKKILGAGLNKTR	S	503							
2406	LOC Os05g30340.1	LRVSVKRESGAHSEDESVAPTVSVK	S	392							
2409	LOC Os05g30420.1	APVDEFEPVDSGSDLESDDLKSALG	S	112							
2412	LOC Os05g30980.1	QGSSIRGVNSELSPNSAPRSFNSMQ	S	305							
2415	LOC Os05g32070.1	QQLAGAASSPPTSSAFPAATTASAK	S	166							
2418	LOC Os05g32570.1	DDGILRVGFPGASRGWRADPAEIVR	S	170							
2421	LOC Os05g32610.1	DFTRPLTFQKDGSEAPVGSEAFTEQ	S	578							
2424	LOC Os05g33030.1	EFGDQGDDSLVPSPKLKQINTPDRL	S	106							
2427	LOC Os05g33310.1	FVRVRPSTPVDLSPLPSPTKAMTLW	S	243							
2430	LOC Os05g33940.1	IDTGVDGLRRLRSIALSDAAALGDP	S	162							
2433	LOC Os05g34780.1	TLLRSGLNCNELSDMEKLPNSEQSS	S	195							
2436	LOC Os05g34780.1	MAIMIEKNVDVDSDGERVDFSDRET	S	143							
2439	LOC Os05g34900.1	VDKVNEMSVGAGSDGNRSSTSMKKR	S	625							
2442	LOC Os05g36160.1	GGAADLARFRSTSSGIGSMNMDDIL	S	41							
2445	LOC Os05g37480.1	GGWTGWGTGAAVSMRQQRFRLWWSN	S	113							
2448	LOC Os05g37930.1	EEEKAAVAARSDSSDEEDCLAGLSR	S	38							
2451	LOC Os05g38150.1	RQRLRYRKLVNSSFADLQKPQMELD	S	79							
2454	LOC Os05g38730.1	TKYYASATALANSPEEKKRREHRSK	S	503							
2457	LOC Os05g38810.1	CIRRAASQLTGSSSIVKSQNEQVPH	S	562							
2460	LOC Os05g38810.1	DEFDGLQSKKKRSLDSFEDPETKAA	S	525							
2463	LOC Os05g38810.1	QDGLELTEKKSTSNLSTDNAYDHPA	S	492							
2466	LOC Os05g38810.1	DPETKAAAPSFGSSFKIGECIRRAA	S	543							
2469	LOC Os05g38950.1	LQYQTSQQKALLSPLPSPRTSMIDA	S	699							
2472	LOC Os05g38950.1	TPKLDVLEESMGSPKEDNKSRVEVP	S	789							
2475	LOC Os05g38950.1	PVKVQEGKLSEDSDDEFYDVDKVET	S	146							
2478	LOC Os05g39610.1	MERTVRTLDRQISQFVAMDRLIWAD	S	116							
2481	LOC Os05g39730.1	ESPSAPRRSPGPSPLGGSPTMGDRT	S	525							
2484	LOC Os05g39840.1	GAKKKVSLGQILSQKYDALSPLRID	S	496							
2487	LOC Os05g41100.1	EEEKKKKDILAGSDNESDGGGGEED	S	18							
2490	LOC Os05g41240.1	QPSPKKPKMDNLSPDSVRDVTQSDF	S	217							
2493	LOC Os05g41500.1	ADGGSSFSFHARSFSGVETTPKFGS	S	93							
2496	LOC Os05g41790.1	SQHVDTNVIRGGSLEKPLTFADKLA	S	508							
2499	LOC Os05g41790.1	DSNALSSQRFGSSSPGGDMKNKTRK	S	110							
2502	LOC Os05g42300.1	DSLFEEGELMLASDDDDEEAPKGPI	S	230							
2505	LOC Os05g43060.1	WGEACKETEKTGSDTDPWGSKVKEI	S	795							
2508	LOC Os05g43280.1	GMRDEDRSLSGESLSEWRSCEQVES	S	28							
2511	LOC Os05g43520.1	RTSLGQEEDVSFSDLEDDDDDGNKG	S	414							
2514	LOC Os05g43530.1	LVEKHSKQQRVGSAPNLDALTKEES	S	612							
2517	LOC Os05g43860.1	GWPLLLEDKAAASRDMVPPLPPLPS	S	498							
2520	LOC Os05g43970.1	QESDEEYDRSQESDEEESDNFQKNK	S	60							

2523	LOC Os05g44380.1	DAAASELLLPVASSPSPRSAAAAAS	S	86								
2526	LOC Os05g44380.1	PRSAAAAASRSPSWDRSRGRASAPG	S	102								
2529	LOC Os05g45060.1	ITVPRVIFVADDSDSPGSSSRGGAG	S	84								
2532	LOC Os05g45830.1	REKAEETEEEYESDLDDAPLPAVRR	S	16								
2535	LOC Os05g45930.1	DSAEKNGFTRRHSLPSSNGKLNSHS	S	524								
2538	LOC Os05g45930.1	YAAAGKDAGFESSPVISEPVLVTPH	S	50								
2541	LOC Os05g46330.1	RKTTGRFIDAPPSPPQSKRPRKLKA	S	424								
2544	LOC Os05g46500.1	LQRSSETFRRSGSSGLVWDDRHLSG	S	16								
2547	LOC Os05g46780.1	RRESRQSPRSSRSAAHGEDTSSVTD	S	294								
2550	LOC Os05g48220.1	VRGTILGYKRSKSNQYENTSLLQIE	S	25								
2553	LOC Os05g48290.1	QVGDMEKLGISESFKAKQAVLMSAT	S	501								
2556	LOC Os05g49150.1	AAASRDSVFDFTSVDDSLAAAAEDS	S	76								
2559	LOC Os05g49180.1	GAFSGLLPVPSRSADDNNVELPVIE	S	427								
2562	LOC Os05g49230.1	SAELNVEDAQGDSENEDIQTREYSD	S	430								
2565	LOC Os05g50480.1	KTANGDEEVAAVSDNHSIDQSDVRI	S	453								
2568	LOC Os05g50480.1	DTVAPAADEHVASSTKSDTENVDVA	S	484								
2571	LOC Os05g50530.1	LPKDIPLPTESVSRGKRRAVSAIES	S	181								
2574	LOC Os05g50530.1	RGKRRAVSAIESSFNLDARAKLDAL	S	194								
2577	LOC Os05g51500.1	DIDKLLAEIDDTSPPTEEAEPVEEV	S	141								
2580	LOC Os05g51754.1	SSDSKAKKRVDYSDDDDDFIVKLKR	S	149								
2583	LOC Os05g51830.1	SQPAEEDEMDFDSEEVEDEEEEEKI	S	106								
2586	LOC Os05g51850.1	GGEVRCLLSDGASSMGNRGPGSPRK	S	380								
2589	LOC Os05g51850.1	PGRPRKNAAAAASPVAPPPASPLKR	S	242								
2592	LOC Os06g01650.1	GSSFSRSRAASWSGAYSEQLSGNAV	S	1114								
2595	LOC Os06g01680.1	SNAFVEKKLQPQSSNISLEPKVIDA	S	304								
2598	LOC Os06g01700.1	RGNPLININNAGSFNVKRRWDDDVV	S	184								
2601	LOC Os06g02130.1	DSRYESKEFDDVSEQYVAVTKKEKR	S	183								
2604	LOC Os06g02170.1	LPDSPHSQASPLSPSALRRHYKSLS	S	90								
2607	LOC Os06g02180.1	GGLPASRMERRLSIMRSQKAMTRSQ	S	229								
2610	LOC Os06g02380.1	GVNGSVVTEKVLSNDNFKFGYNAAT	S	528								
2613	LOC Os06g03860.1	THDSRKPATSIGSAYRLLTPSVKVQ	S	501								
2616	LOC Os06g04330.1	SRIRRMIEGLDDSDEPEDIFDPVFK	S	403								
2619	LOC Os06g04560.1	EIAVLKSQLLQLSLDADETRRSLDR	S	580								
2622	LOC Os06g05190.1	GKPWRKNKELVESDEDQKKPHKEHQ	S	153								
2625	LOC Os06g05250.1	QKEGKKRMKRVGSVDIPQEAFHELL	S	645								
2628	LOC Os06g05660.1	LAGQHTDVLEALSPNVRKRVEYLRE	S	55								
2631	LOC Os06g06014.1	EDVETGKNDGSLSDVDAMKENSNVG	S	292								
2634	LOC Os06g06014.1	GPEWPLAPSPPASPTTWHDNKAASS	S	85								
2637	LOC Os06g07160.1	ATGLGSTDPSSSSTPRKRASSGRSY	S	268								
2640	LOC Os06g07820.1	DDRVLIKTRSNQSIDQWVFAKSKSE	S	271								
2643	LOC Os06g08140.1	RVSRPGRVARSISAEGLNSLRTLLR	S	346								
2646	LOC Os06g08280.1	LGLSKVKCQTLISGGVRGTLPWMAP	S	1160								
2649	LOC Os06g08480.1	EETKPDASEETSSSESGSKKKPVKR	S	20								
2652	LOC Os06g08480.1	DASEETSSSESGSKKKPVKRYLIKW	S	25								
2655	LOC Os06g08740.1	FGDLLMNLPRIASLPQFLFKIPEDS	S	526								
2658	LOC Os06g08740.1	GPAQPPAMSRKDSFGDLLMNLPRIA	S	513								
2661	LOC Os06g08840.1	SRSPQYNRGRDESPAYDNGYRRSRS	S	173								
2664	LOC Os06g08840.1	SVSPARARSYSRSPQYNRGRDESPA	S	163								
2667	LOC Os06g08850.1	DLSIRESFFSRRSFEDDGSPEHSLV	S	642								
2670	LOC Os06g09570.1	LRALSRSRSRSKSRPPEEVVPGEGF	S	610								
2673	LOC Os06g10710.1	GDRRPAARAPNPSLSPRGGGGAPSR	S	56								
2676	LOC Os06g11610.1	RRYDVVDESGTDSGDEYDATDDGRR	S	81								
2679	LOC Os06g12030.1	ESVEDEKNEGSESISSGNIGSDAEP	S	180								
2682	LOC Os06g12080.1	AVAFLSGLGLSRSGIAAAVAADPRL	S	92								
2685	LOC Os06g12400.1	NGSKQIDASDLPSDDSADNDYDPTL	S	346								
2688	LOC Os06g12580.1	GRKTEEESYGGGSGYGYGKKAQEES	S	284								
2691	LOC Os06g12780.1	PSDAGAAPHAPPSPPRRGAKKGAAA	S	126								
2694	LOC Os06g13820.1	PNTDGGSMRQSNSDGALDTMARRPA	S	722								
2697	LOC Os06g13820.1	AASYANDISEAESPRTPNRPGEDWR	S	844								
2700	LOC Os06g14412.1	IMEVEEKLIDDISGSPSSHLPVALK	S	1985								
2703	LOC Os06g14412.1	CEVDHKIDCTIFSPIGDQDTRNRID	S	1436								
2706	LOC Os06g14412.1	EVDKEIVHCTISSPIGDQENLQGNI	S	2427								
2709	LOC Os06g14412.1	PAMDASESKSPESDVHELSERVEFM	S	1373								
2712	LOC Os06g14412.1	KNDDSPIERSSPSADKIAQVAYGGE	S	935								
2715	LOC Os06g14412.1	TDLAGSQAPYDASDKEDTAADLIGP	S	2478								
2718	LOC Os06g16160.1	AFRSALRAAATGSHRHATRRTVIVR	S	690								
2721	LOC Os06g16390.1	DTNCGEALICSDSEDEAVEDEEEKK	S	180								
2724	LOC Os06g17290.1	TQMAVSALNRALSSEYPSKSRSEGR	S	73								
2727	LOC Os06g17840.1	EPDDHNLNSKEESDNETLSVWKKRT	S	1545								
2730	LOC Os06g17840.1	KKGAKKPHAEILSSSPKKSDEAGSS	S	1304								
2733	LOC Os06g17840.1	PHAEILSSSPKKSDEAGSSKRTVDS	S	1310								
2736	LOC Os06g18130.1	AYKVAIQNKDARSGKEASSSGGGDC	S	69								
2739	LOC Os06g19650.1	VGPAVAPEASVDSDQHIEDAATEDG	S	49								
2742	LOC Os06g19660.1	TDKYSVEDSQSRSADGSVSTLLKHP	S	825								
2745	LOC Os06g20090.1	YKAGPIIGSGGHSSGKSPYSSRIRD	S	747								

2748	LOC_Os06g20390.1	SQHVDQDFSRRDSFSAVQPGNNALR	S	206							
2751	LOC_Os06g21580.1	KLTNGRSHEDKGSSGEEVVPARKRR	S	22							
2754	LOC_Os06g22070.1	ASADENLVRVIDSEIECIVQSEEGA	S	88							
2757	LOC_Os06g22650.1	PKSVQARGDKSPSIKPEGAKQPDQR	S	290							
2760	LOC_Os06g22820.1	LQVPESKDEDDDSTEEWANFRGGDS	S	443							
2763	LOC_Os06g23460.1	RSSKRGYHYDQDSPPPRSKPRFDRR	S	17							
2766	LOC_Os06g24540.1	SSFSSLKDSRRASFSIDRDGRRWSF	S	574							
2769	LOC_Os06g27970.1	DEEDAFGRDFNSSPSPTAPPARSGE	S	22							
2772	LOC_Os06g29400.1	VQRPVDLYKAIFSDDSDDDMAEPLA	S	730							
2775	LOC_Os06g29430.1	SNSNRSVKKSSSSNSQNTEGWISSQ	S	1125							
2778	LOC_Os06g29430.1	MGGPSDEMWDVQSATSQETFKTADK	S	1049							
2781	LOC_Os06g30320.1	GHARALATLDRKSIDSYVTRVAHHD	S	55							
2784	LOC_Os06g30750.1	PADTAAASPEKVSSPPPEPAPAVRS	S	47							
2787	LOC_Os06g33520.1	RMSGVSRRGGRGSDLDDSEDDEYSR	S	283							
2790	LOC_Os06g33520.1	ELSDWISDLKTDSFHLGLSSGDEGE	S	82							
2793	LOC_Os06g33520.1	RMSGVSRKGGRGSDLDDSEDDENDS	S	320							
2796	LOC_Os06g34440.1	SRQKQEVKYNEDSDGDDTDCHGNGD	S	367							
2799	LOC_Os06g34710.1	ADLRSNCLEELVSGDADVRGTSAEE	S	326							
2802	LOC_Os06g34710.1	EGERGGEVVLEVSDMDEEDGEDDTD	S	271							
2805	LOC_Os06g36360.1	NKNDVEDGTANRSPDGQPKRKHDTA	S	525							
2808	LOC_Os06g36360.1	YDSEPGRSVKRASISPSISPVHQKT	S	552							
2811	LOC_Os06g36830.1	GYRFIAVMPAGYSLDKQMLLRFLGA	S	130							
2814	LOC_Os06g37770.1	AVGDAATAVSTLSCRAPRPRRSSEV	S	74							
2817	LOC_Os06g39344.1	NPSVKCVLVESSSPRAFSAGMDIKG	S	117							
2820	LOC_Os06g39600.1	AGRKLPGLSRHASFVRSPANSTKSG	S	37							
2823	LOC_Os06g40440.1	GGRKVCNGGGGGSGGKGDGGRRDAD	S	140							
2826	LOC_Os06g40630.1	GVVSKGVLGLPGSFKSTTSSVPSSK	S	82							
2829	LOC_Os06g40650.1	FGPQTSGFQQSESFKQRQPVATTAP	S	377							
2832	LOC_Os06g40740.1	DLADEFFRARAESEDDDDDEEAVVV	S	105							
2835	LOC_Os06g41730.1	PVDEANAVEEEKSGKGEDGIEEAAD	S	32							
2838	LOC_Os06g42200.1	RASGSRDRGPGSSRPADAREKRKQG	S	343							
2841	LOC_Os06g42490.1	REVIDYRGPFCGSPDINSRRSDSPD	S	430							
2844	LOC_Os06g42810.1	VRTLSDLGGGKDSAGSEDSEDDEYK	S	172							
2847	LOC_Os06g43170.1	DDDDGDLKKRSMSFIGRSTPQHHSK	S	494							
2850	LOC_Os06g43560.1	GHARKDSAESIGSDLSSLRGSELSV	S	307							
2853	LOC_Os06g43790.1	EPRKTRKIVELSSFEKRDREDDNGF	S	1575							
2856	LOC_Os06g43840.1	QNSPNNQLTGEGSLSGNARHREDGL	S	470							
2859	LOC_Os06g43840.1	HREDGLTRRDNVSPVAPAAVPTYMA	S	489							
2862	LOC_Os06g44970.1	GGGELEIEDGLKSPAAGLAAKFPVS	S	425							
2865	LOC_Os06g45560.1	KPHIAYLQETKMSNIDVAIVGSLGG	S	785							
2868	LOC_Os06g45870.1	SLAHKDRLGELLSDDDELGLGGRGG	S	60							
2871	LOC_Os06g46400.1	DRYGRSALRDDDSESDEEAAPRRSR	S	651							
2874	LOC_Os06g46880.1	GGVAYPLPPRHVSPDPSTFSGSVHQ	S	389							
2877	LOC_Os06g46890.1	ADLLASDTLKPCSKDQGSSAAKEDP	S	249							
2880	LOC_Os06g47330.1	KSSKKIPLNAVTSVIDGLKKLYIEK	S	158							
2883	LOC_Os06g48530.1	PPQPAATRKRSRSPPPPPPPPSLPP	S	53							
2886	LOC_Os06g48530.1	DGSRYGRGDRSPSLDGADDQMFDAF	S	601							
2889	LOC_Os06g48640.1	TLARDIEVMEPKSPEDIYKVHLIDG	S	352							
2892	LOC_Os06g49740.1	GGDDDAEQEDAGSDDGYPQMTEKQK	S	118							
2895	LOC_Os06g49800.1	LGVPSPSVSRGISFDENTLTDPVEL	S	238							
2898	LOC_Os06g50840.1	PYGARGTAPRSNSPLFDDYGRSIGS	S	145							
2901	LOC_Os06g50890.1	RARRERSRSLPYSPYRMPERGYGRQ	S	253							
2904	LOC_Os06g50890.1	ETPERRRYSGSPSPYRGNPKSRSRS	S	15							
2907	LOC_Os06g50890.1	DGRGGRGYSPHRSPPYGGGRARRER	S	234							
2910	LOC_Os06g51250.1	GNSFTRYDSMNSSSDHDRTDAFARF	S	1131							
2913	LOC_Os06g51250.1	RTDAFARFDSMKSTDYNSRGYSFDE	S	1148							
2916	LOC_Os06g51250.1	DQGSNDTFGRFDSFRSNADQGGGNS	S	1109							
2919	LOC_Os06g51250.1	RSLFDSGPSRAESPTASSIYGKEQR	S	1021							
2922	LOC_Os06g51270.1	MDEQDQLGGLLDSVHGGNRKHEGMT	S	893							
2925	LOC_Os06g51400.1	DAYFPSIASDSESEGVRSKTPMGAN	S	136							
2928	LOC_Os07g01550.1	GNMLHKDHDHHFSEGAECDIIDCDH	S	126							
2931	LOC_Os07g01720.1	DETQDILPAGSGSPDAQDLELILRQ	S	252							
2934	LOC_Os07g01880.1	VASGRRGVVASESEGSEDDYYAGRG	S	174							
2937	LOC_Os07g01880.1	RDHEVVRDVFGESDEDGPAPYRDQH	S	219							
2940	LOC_Os07g01920.1	TFTTDRMGRQLSSMGFDPSAAMDRA	S	552							
2943	LOC_Os07g03070.1	KDALLAGKDDNASAADGGSEKSAPE	S	311							
2946	LOC_Os07g03180.1	RLTCPEKCFKSFSFKDKHGGGGGGG	S	109							
2949	LOC_Os07g03230.1	DKDDDDETDDDASDADIEDPEKMVM	S	199							
2952	LOC_Os07g04530.1	HANVPSWQASGSSPSKAVAPVQMSS	S	1504							
2955	LOC_Os07g04530.1	ERRSAVAQQQPPSPTPAKVSINQRN	S	1479							
2958	LOC_Os07g04530.1	EFPTLSSQTRSSSIKDQSPMNRQKS	S	1558							
2961	LOC_Os07g05190.1	VGGFSPSKGSRFSWSPDSGEAYTQE	S	738							
2964	LOC_Os07g05190.1	ELLTGRCAGDVVSGSEGGVDLTDWV	S	993							
2967	LOC_Os07g05610.1	LQHSLSRNARVYSGIDLSKDEVTQI	S	815							
2970	LOC_Os07g05610.1	AAESNAREIGGSSMNGGEEVLNAET	S	664							

2973	LOC Os07g05610.1	PSSSGAGPSRNLSFDLDIDVNKFDT	S	559								
2976	LOC Os07g05610.1	RGFNLFDVNQPSSSGAGPSRNLSFD	S	549								
2979	LOC Os07g05610.1	RKGVLTRGISATSGINGWVAEGTER	S	393								
2982	LOC Os07g05610.1	EENSTRSAKHTASADPDQIHSRLPE	S	302								
2985	LOC Os07g06130.1	RDNLQGSAFLGSSRDLMSTKNEMSN	S	751								
2988	LOC Os07g07000.1	LLTDSFELPRGGSSRDGDIEMGMQA	S	16								
2991	LOC Os07g07194.1	DTREHRLRFVDSSPLNSYSSKEDLV	S	249								
2994	LOC Os07g08190.1	PVPTNGKDLAMGSTDNGQPQRVRKG	S	434								
2997	LOC Os07g08190.1	PDNTKTISLEKDSTLEDADKGKQTA	S	348								
3000	LOC Os07g08190.1	QQYAFARRYRTPSPERSPVRSRYND	S	463								
3003	LOC Os07g08330.1	RKEKLDSKRTKLSPEEAAKVKAAGK	S	367								
3006	LOC Os07g08960.1	SGGYDRGGMRMGSGDRYGTGGPARF	S	270								
3009	LOC Os07g09010.1	MSKETERSPRPSSPAPAETSWVKGM	S	333								
3012	LOC Os07g09384.1	PPPPEPTLARIESTESFSLPLHKVD	S	232								
3015	LOC Os07g10350.1	KSYIGPDSTGDTSDDEDDEIVPEEI	S	514								
3018	LOC Os07g10350.1	PESRASVLPLQVSLDESEGSDQEND	S	557								
3021	LOC Os07g10600.1	AAMSRSGAMDLASGLGGKITKDEVK	S	60								
3024	LOC Os07g11290.1	QGPEKPVSLRSRSPDDYWRREALDG	S	204								
3027	LOC Os07g12250.1	IRADSQVFLFSNSKCKRYFHNRLKP	S	36								
3030	LOC Os07g12910.1	AVVDDGYDEEEHSETLCGTCGGRYN	S	186								
3033	LOC Os07g15490.1	DAGTCKECYEEASETEEELKREIDD	S	26								
3036	LOC Os07g20150.1	AHMNRKVLQLSASFERAKGTGTELA	S	62								
3039	LOC Os07g24020.1	PAYKDLSGDEVHSMVKDISNEFGKK	S	403								
3042	LOC Os07g25460.1	LQPLEEFTTPPSSPDNSKSPVAQSS	S	578								
3045	LOC Os07g25550.1	KEELRVPGNKSGSNMGARGSSHPTP	S	81								
3048	LOC Os07g25680.1	QEGLKSSLQPSDSAKSLETYTASKA	S	506								
3051	LOC Os07g25680.1	KNPPPHSGRVFHSERIPREQAGSLN	S	567								
3054	LOC Os07g26440.1	SKKKEGAKTNFDSGDDYNNKDESSY	S	455								
3057	LOC Os07g26440.1	ESEVLDEGSDGSSSERGSSHGHKSH	S	370								
3060	LOC Os07g29360.1	PQSAIERKRRAWSESEDDEPVQRPV	S	1046								
3063	LOC Os07g29450.1	RPLDDSSLYSSPSGKIIQPGSSDFH	S	32								
3066	LOC Os07g30774.1	TSIPNEVPARQNSLGLWKYLDDDSP	S	340								
3069	LOC Os07g30820.1	GYGFRGSPMPVSSPWSGGALVENND	S	179								
3072	LOC Os07g30820.1	GACGAPRPSPSLSPRVPPPPAAGYD	S	72								
3075	LOC Os07g31450.1	SAVDDMDTGSCRSPVRDTPDPDNQK	S	2089								
3078	LOC Os07g31840.1	KLAAAREAPYAKSRTQFTRDMQMAK	S	604								
3081	LOC Os07g32400.1	TIKVERATRDVVSPSSQGTVRKAAG	S	322								
3084	LOC Os07g32530.1	RSLESDQYDGTDSEEEVASASSRAL	S	729								
3087	LOC Os07g33340.1	RTASKGKKRYFNSDDEEGSKDAAKD	S	740								
3090	LOC Os07g34740.1	KKGTKGKKGKEQSVEDELPDIDLSK	S	554								
3093	LOC Os07g35580.1	GGGDDGGGTGSYSGSFVGTLPSSGR	S	667								
3096	LOC Os07g36420.1	AGAAKNPEVETSSSIHRSENPGLRT	S	169								
3099	LOC Os07g36600.1	PRPPTPASLQPESPGVFFTAAAAAA	S	37								
3102	LOC Os07g36940.1	SNKNATKDTRNLSQEPQSASSAEDL	S	646								
3105	LOC Os07g36940.1	ASSIDRSSARSTSESTENVQEVGKS	S	714								
3108	LOC Os07g36940.1	VGPVAPQMSRSGSDADRWQQKGIFP	S	1142								
3111	LOC Os07g36940.1	NTQFAGPSNRPASQEGRSGNKLYSE	S	1589								
3114	LOC Os07g36940.1	PEEKVDFIGASESLDSSSIADHELP	S	957								
3117	LOC Os07g37140.1	VTRDLPNGLMRISPGSSEPAASLPS	S	377								
3120	LOC Os07g37140.1	RKGRRNVNASPASPPAATSDVTRDL	S	357								
3123	LOC Os07g37750.1	KSKGRTDDIVPPSPVKAPDADGADK	S	1444								
3126	LOC Os07g38090.1	GNRSPSSRSSSLSPITVDRGKKEYP	S	205								
3129	LOC Os07g38090.1	RHVPHNSGAGLLSPRASSSIDMTAA	S	349								
3132	LOC Os07g38160.1	GLVVKVPEFPNDSDDELEEEKETEV	S	562								
3135	LOC Os07g38970.1	PGRRMGHAGAIVSGGKGTAQDKIKA	S	289								
3138	LOC Os07g39470.1	EGAGCMRHDSPSSQSFTTRSGSPLS	S	88								
3141	LOC Os07g39560.1	SITSKNNGSSIGSPGPGRTRKIFVG	S	99								
3144	LOC Os07g39620.1	AKMKLPLDLDNGSDKSAAVSGAGAA	S	392								
3147	LOC Os07g39620.1	VQSFSTDNSTADSAGTFRSDTPVSS	S	263								
3150	LOC Os07g40270.1	RFVLGSLEGFFPSDHTSSPGNKQII	S	232								
3153	LOC Os07g41190.1	KRRLETIEADDDSDEGLGSPVAAGA	S	23								
3156	LOC Os07g41190.1	RKAPLPGSSDNESDEEGRVDDILRN	S	211								
3159	LOC Os07g41190.1	EDDLPVYDEGLSSEDEGVDMVGRER	S	127								
3162	LOC Os07g41694.1	SQGAKRKRDDDPSGSGDDDEDDDGV	S	455								
3165	LOC Os07g41810.1	AKGARVSFGENFSPARAKGYQFGMV	S	206								
3168	LOC Os07g42400.1	ILTELQSGEAELSDDSVMTPTTKAT	S	246								
3171	LOC Os07g42410.1	RSNAEMRAKFSRSSSVPDSTYISRC	S	849								
3174	LOC Os07g42410.1	SIHQGTNLSRQSSSAEQELSLHGGK	S	666								
3177	LOC Os07g43030.1	YTDGIRYPLLKQSSLDLFKIEPDGL	S	352								
3180	LOC Os07g43050.1	RYRERDYRRRSRSRSYTPDDYRRRG	S	161								
3183	LOC Os07g43316.1	QDLESQIKCVSISPDDILVNKNDNV	S	120								
3186	LOC Os07g43316.1	SIYPEKLNLDRSSGDESMEEDAMET	S	210								
3189	LOC Os07g43316.1	EVTLEHVSSGDESMEEDVMETKHVD	S	251								
3192	LOC Os07g43316.1	GLESKQDIVKPSSSNPSSVGDDLQT	S	156								
3195	LOC Os07g43316.1	KHVDSNTKVEHVSSGDESMEEDVME	S	272								

3198	LOC Os07g43316.1	DMSLKKKEGSPDSIYPEKLNLDRSS	S	198							
3201	LOC Os07g43350.1	AMEPSPRVVLPTSPHFIDSRFHEEP	S	735							
3204	LOC Os07g44970.1	AKLEAAEEEGAGSEGEEEAGRKNGM	S	335							
3207	LOC Os07g46170.1	QTLDTTPLPGSDSIEANRRRERGQR	S	277							
3210	LOC Os07g46440.1	EDPWAKDFRAEGSEKGDVFEEIFKE	S	116							
3213	LOC Os07g46590.1	EPEAREEGEISESEAETKYRQDKEE	S	764							
3216	LOC Os07g47100.1	PASGHPVTSEPSSPKSLHSPLLTSM	S	458							
3219	LOC Os07g47350.1	SLTRSSRSETLRSLQSIYEQESGSL	S	707							
3222	LOC Os07g47360.1	VAKTSPIESVSSSPPRISNNDKVSH	S	1097							
3225	LOC Os07g48410.1	HQLGARMLQHSNSANEMLLRRKLEE	S	486							
3228	LOC Os07g49040.1	VVDIIPSDYRLTSPQLSPKRNQSKF	S	306							
3231	LOC Os07g49270.1	PLHTGREGKPIISPASTKRVGPLVR	S	94							
3234	LOC Os07g49380.1	KRKEVEEEILDDSDEDWDPRKKGAT	S	248							
3237	LOC Os07g49380.1	WDPRKKGATDSDSEVDFDRKRVSKG	S	264							
3240	LOC Os08g01054.1	SDEQWRMTSGNFSPDDQRNTWLPGA	S	1349							
3243	LOC Os08g01420.1	QHPNGAAGGKVVSADAAIQDAAAAA	S	128							
3246	LOC Os08g01900.1	RRPSERGFPRIISSSSMDTRSDLSA	S	524							
3249	LOC Os08g01930.1	SAKVDEPVATENSDAAPAEASVDAV	S	100							
3252	LOC Os08g02354.1	VKSTKDNFSSPGSPRIDDQSAGAGQ	S	428							
3255	LOC Os08g02690.1	LSLASPRSPTGGSTSALLQQYEQQR	S	40							
3258	LOC Os08g03290.1	VVDLTVRIEKAASYDAIKSAIKSAS	S	256							
3261	LOC Os08g03520.1	KCGEEGHMARDCSQGGGGGGGYGGG	S	151							
3264	LOC Os08g03840.1	APLKEEENWELDSEPELTWDEKVVE	S	121							
3267	LOC Os08g05540.1	ETRVRLEDLGGESDEDEDDEVDKVM	S	135							
3270	LOC Os08g05840.1	AALKRGGNVSDDSEDEKPLAARFSK	S	214							
3273	LOC Os08g05840.1	LKRPFVEDKSDDSEDDHKPIGLRRK	S	118							
3276	LOC Os08g06020.1	SPSRVSESENGDSLGGGFTRALRER	S	161							
3279	LOC Os08g06020.1	PPSPVPEESEEGSPSRVSESENGDS	S	149							
3282	LOC Os08g06140.1	ITILRQHQAPNASLLSDGDDAESTG	S	561							
3285	LOC Os08g06140.1	APEESTAEKDMPSDGDDAEPTGITI	S	514							
3288	LOC Os08g06344.1	NAGKKWTLDGEESDEEGNQEDGKKS	S	233							
3291	LOC Os08g08080.1	RTRHDSEEPQDLSPPRRKGRHDSEE	S	244							
3294	LOC Os08g08080.1	LSRHDSKESQDISPPRRRTRHDSEE	S	227							
3297	LOC Os08g08080.1	DLSPPRRRTRHDSEEPEDLSPPRRR	S	288							
3300	LOC Os08g08570.1	KAFVRAAGGPISSFAEKRGSFFSGP	S	20							
3303	LOC Os08g08820.1	GGLVDEFESKSCSENVDGAGDGLSG	S	84							
3306	LOC Os08g09910.1	LVDDYNYGSDIDSDDDLHEYSQIAH	S	394							
3309	LOC Os08g12760.1	ALQENRRQQQPASPELQKPAENKAL	S	507							
3312	LOC Os08g13350.1	HGEPGKKSSGPQSGGVTPMARQNSG	S	133							
3315	LOC Os08g13350.1	PRKVSGPLDPSVSMKMRATSFAHNP	S	185							
3318	LOC Os08g14340.1	SANMIISEGGGTSGYSKILPTVLSV	S	392							
3321	LOC Os08g14460.1	VGNGRQEAGSPQSPLGGKTDVFDLP	S	323							
3324	LOC Os08g16130.1	GGFIKHQNEVGMSMTDYGLDKGGSM	S	186							
3327	LOC Os08g16610.1	KNITPPALERTISPGQGGLSGTSIP	S	275							
3330	LOC Os08g18060.1	GDEDVGRPAQPPSAAVPANNPRRPV	S	43							
3333	LOC Os08g20390.1	KASGVATTGSASSRRRARRAAAVTR	S	142							
3336	LOC Os08g20660.1	SPGDSLRDVHDISLNLKLSLDSEKS	S	719							
3339	LOC Os08g20660.1	KSDDATEVSEADSPGDSLRDVHDIS	S	707							
3342	LOC Os08g22864.1	VNSMQPLSRRTRSIKGDNHFGASAD	S	256							
3345	LOC Os08g23360.1	VPERLSKGVSTLSLGERKQAFLLDM	S	542							
3348	LOC Os08g23930.1	VNNAQRSHDRVPSPTTSARKRQKTS	S	143							
3351	LOC Os08g24760.1	SSVIEGTQHEEDSDQEDIDEKDMLK	S	389							
3354	LOC Os08g25080.1	SVVEDERQESGKSDDENKANDGEPS	S	119							
3357	LOC Os08g25570.1	QRSRLWSGGAYESDTEDPDNMKTET	S	429							
3360	LOC Os08g27870.1	GGDGEGVGGMARSDDGGDGGGGNVV	S	78							
3363	LOC Os08g29124.1	DQGAICPRSDSPSVASLPKEETSPD	S	706							
3366	LOC Os08g30060.1	IEPLQKSLGKFRSENNAMRAQGAGL	S	162							
3369	LOC Os08g30060.1	ARKRSATAGGAESEEAAESDAKSDE	S	530							
3372	LOC Os08g32100.1	APAKKLGFGLIGSGKRTSVPSVFAE	S	684							
3375	LOC Os08g33190.1	RDPNAPPPPPKPSSPRPRGRPPKSK	S	110							
3378	LOC Os08g33380.1	LLQVQAMRSSEESSSDEDDEILSEL	S	62							
3381	LOC Os08g34060.1	ALARGESFEDEDSDDDDDMIPKAGK	S	481							
3384	LOC Os08g34950.1	HQESSIGVRRVLSDGQFPVNADISD	S	1316							
3387	LOC Os08g36480.1	AEAAAPGLKRSTSTPFMNTTDGKQF	S	532							
3390	LOC Os08g36760.1	SPSGRPSFSRSLSVQQVKELESCFS	S	435							
3393	LOC Os08g37280.1	KGRRTREGARQLSPTDGEDQASGKK	S	192							
3396	LOC Os08g37280.1	SEHMGEDTKDQHSAEQHQELGYTAT	S	161							
3399	LOC Os08g37280.1	HEAVKKELSHVNSPCDSTEEAGNLK	S	117							
3402	LOC Os08g38320.1	LEEQKPAHRRQESSESILDKGPSNV	S	55							
3405	LOC Os08g39140.1	QALRDSSMAGYMSSKKTMEINPENA	S	602							
3408	LOC Os08g39590.1	RRLSEPDDGDGGSDSGWRRRRAFET	S	394							
3411	LOC Os08g40150.1	SKAVPTAGMGPNSPPSRGTLSESSG	S	314							
3414	LOC Os08g40230.1	KPLPEVRDRDFESDFEEMHDTDEEL	S	114							
3417	LOC Os08g40820.1	EVKRRLKELRKNSFMVLIPEEECAE	S	95							
3420	LOC Os08g41010.1	INYPFRTKCNRPSCEAEKPFQTNNA	S	267							

3423	LOC Os08g41400.1	VADDDGNAERSSSPPHVSEVQVGLS	S	142								
3426	LOC Os08g42110.1	EVESSYEGEKPGSPISLSSGLRRRP	S	99								
3429	LOC Os08g42600.1	SPAKSITSPSPMSPPRFCASPTGNG	S	397								
3432	LOC Os08g42600.1	RRNVLVDNNSFRSPVKDTLKSKLPP	S	696								
3435	LOC Os08g42700.1	GKTTLVKSIYQSSELEKLGFERRAW	S	477								
3438	LOC Os08g43090.1	RASGTRTGSVADSSENEAESHSTPV	S	221								
3441	LOC Os08g43090.1	GKSRHCRSLSVDSFIEKLNFDESPK	S	252								
3444	LOC Os08g43130.1	RTGERQLRSMRRSGPLDYTGTRKVK	S	1316								
3447	LOC Os08g43410.1	KGILHDVGPEHSSGGGGGMGGRHAA	S	231								
3450	LOC Os08g44430.1	VGKTETLQSQADSFHRHGRELRRKM	S	197								
3453	LOC Os08g44540.1	IAQEVNDDSDDDSGETESRTSAFSK	S	115								
3456	LOC Os09g02810.1	DAEDACDTIECVSPISSGEKDGKGT	S	676								
3459	LOC Os09g03600.1	KALVEKDSDGEDSVAPVVTKLSSGE	S	727								
3462	LOC Os09g03610.1	SRGGPAFGGPGFSPRSDAALVIRPT	S	313								
3465	LOC Os09g06560.1	KRLRRKSTYHDHSNGDSEDEDLILQ	S	495								
3468	LOC Os09g07300.1	NRSALKRLSSKSSGNSFGSGSLIPK	S	1521								
3471	LOC Os09g07300.1	KQVTLSELESVASHEEEALFGNLFA	S	548								
3474	LOC Os09g07900.1	ATEVKELAGSSSSPSLKTGGVCNIT	S	3211								
3477	LOC Os09g09360.1	QDGLEQLLPMDDSPTSVKSFTDCQL	S	946								
3480	LOC Os09g10710.1	TESLIKEVERVFSINNPDPLEVEKA	S	332								
3483	LOC Os09g11240.1	LEDPDDDSDADLSAEEQRDSGSNQR	S	133								
3486	LOC Os09g12270.1	KRRRLKKKHPADSSDDNNDDSSHRP	S	185								
3489	LOC Os09g12730.1	ILKFEQSDPEVSSPTRKPETFVSHL	S	627								
3492	LOC Os09g13630.1	VDSGSPEDAQAGSEDDYVQRENGSS	S	411								
3495	LOC Os09g14960.1	NPEELTEVEPSISNEKLLLASLKQQ	S	323								
3498	LOC Os09g17850.1	SKRTSSLKMVPPSDLAETVRQKLKI	S	327								
3501	LOC Os09g19734.1	TVTVDELTPGLKSILSFAVPDQRSG	S	91								
3504	LOC Os09g19910.1	VGAVHDKMEHNSSPHGNNADLHKDS	S	56								
3507	LOC Os09g21000.1	RRRFGKQPRRVDSLDVEAMSVRGAH	S	108								
3510	LOC Os09g23730.1	AAPAPAPAAPAASSPRPRGRPPKPK	S	127								
3513	LOC Os09g26540.1	ASDLKGAINKDGSSDEEDFTEEGLS	S	49								
3516	LOC Os09g26820.1	RRLAPPSPATVGSPRRTSSASGGGG	S	73								
3519	LOC Os09g27080.1	SFKRGEIELQIGSPRSPRGDGVGSP	S	64								
3522	LOC Os09g27850.1	AAAAAGGSASEGSDAEASAEAARGH	S	28								
3525	LOC Os09g28220.1	RSSRRKRSKQSDSEDQAPSDADLGV	S	333								
3528	LOC Os09g28220.1	RSPSPYRSRRDRSPSPYRDRRRQWS	S	58								
3531	LOC Os09g28220.1	GGGGAGAGAWSASDDDDDEQLKGLT	S	108								
3534	LOC Os09g29070.1	NDRALIAEGSCGSPRSIKVYLGECN	S	605								
3537	LOC Os09g29750.1	MECTDMKPSKKVSEPCDLLDDKFSS	S	586								
3540	LOC Os09g29890.1	SGKGSEHQSGSRSANELLPPSASFV	S	600								
3543	LOC Os09g30350.1	SSTNAAAPRHDFSFGGGFGRERDRP	S	182								
3546	LOC Os09g31230.1	LRASMVQRRGYTSDDDLDDLGNPLA	S	724								
3549	LOC Os09g31486.1	GGGGGSGGGSSSSSGGDQTPEAEYQ	S	664								
3552	LOC Os09g32090.1	VAKGQDELGENHSPSLQKSANNPNR	S	59								
3555	LOC Os09g32430.1	LDQRKGHDEVSRSKDGTDTEDATID	S	145								
3558	LOC Os09g32540.1	RAGGGESEGNAGSPSLGSARGDADL	S	86								
3561	LOC Os09g32820.1	LRLSSPSAPSSPSSAKAAAAAAAHS	S	38								
3564	LOC Os09g32820.1	EALRLSSPSAPSSPSSAKAAAAAAA	S	36								
3567	LOC Os09g33450.1	KEGRIAHASPDLSLPGELVEGRAGM	S	385								
3570	LOC Os09g33510.1	RVSPNTSPPLLSSPKIKELHELPRP	S	496								
3573	LOC Os09g33510.1	RHEQRVDRRGGHSPAAAAAANELLA	S	27								
3576	LOC Os09g33510.1	LPTPPGPISRSFSIPSRGMRTSGIS	S	571								
3579	LOC Os09g33510.1	TSQTVTANPTSGSPLGNKGAFYSSP	S	368								
3582	LOC Os09g33600.1	LGKALLLQEQPTSPSDGSSRCFDRS	S	702								
3585	LOC Os09g33870.1	TEDDGDRRKRRRSREPGELSPYKED	S	300								
3588	LOC Os09g33960.1	RAGAFSSGGVVRSASNENFCPTSEE	S	429								
3591	LOC Os09g33980.1	LHPYQESEDDQESSEEQPAQDRREN	S	164								
3594	LOC Os09g33980.1	SARPKRKKLSNSSSIPRREGLRPRR	S	133								
3597	LOC Os09g33980.1	KSLGDVDLSKPISPEEAPKEPDSNG	S	1083								
3600	LOC Os09g34060.1	AAAHGARPKHQHSLSMDESMSIKAE	S	136								
3603	LOC Os09g34070.1	DAAADPYDTRAGSEGLYSAGYSQRE	S	400								
3606	LOC Os09g35710.1	GPMYPFSMPSHRSPSLYPHVEHAPS	S	380								
3609	LOC Os09g35760.1	GNGGSGSGGDGDSLGRGREEENDSR	S	86								
3612	LOC Os09g36090.1	SSHGESAGYTSKSDSPNIGTHAVKV	S	1177								
3615	LOC Os09g36090.1	ASIDLVKASDSVSEEGTGMDILVQP	S	173								
3618	LOC Os09g36090.1	ADGVPEVNVVAISRDNSAENEAAAA	S	297								
3621	LOC Os09g36090.1	GGDSGGRSWRSQSGGDGGSWRPKRG	S	1728								
3624	LOC Os09g36160.1	PRARPSATTPTTSSGDQQMVTVAER	S	181								
3627	LOC Os09g36270.1	SKQLHRGGVDSPSWRSGAQPDNIVI	S	168								
3630	LOC Os09g36270.1	TQAPAAMLPRSGSRPQLDLSGAAIH	S	52								
3633	LOC Os09g36360.1	STLRRRRPRRGWSLKQHEDDTAASP	S	53								
3636	LOC Os09g37006.1	TITLSKKELDTSSIGYQSPLPADKV	S	121								
3639	LOC Os09g37230.1	EEVASRLLNRQQSIHPPPAFGSSTN	S	166								
3642	LOC Os09g37520.1	TSRGEVTPPAPPSPPRVSTWDFLNP	S	357								
3645	LOC Os09g37860.1	RAPKKKQELLEPSSDDSELEQQQER	S	46								

3648	LOC Os09g37860.1	VPEEAIEKTDPSSDDLQEQPIQSKI	S	571								
3651	LOC Os09g38200.1	NLVFRGAGSASSSTSRQKQGEQWNE	S	406								
3654	LOC Os09g38500.1	PPPDAPLFRRVESLERGPRLHLSLI	S	115								
3657	LOC Os09g38570.1	GSIQEKLDKVENSGHVEGRAAESDV	S	199								
3660	LOC Os09g39020.1	PFRKGTQERKSLSPNIPHFPADQPG	S	176								
3663	LOC Os09g39462.1	EVTLPNTLHRSSSLELSSGNRLGEG	S	244								
3666	LOC Os10g02630.1	MMRGNPDGGGMQSRTKGQRVNVQQL	S	20								
3669	LOC Os10g04580.1	KVDIAPRDGKRVSSGNLVDVARQWV	S	181								
3672	LOC Os10g06130.1	DDDGYRRHRREDSDDPEEEDPDERQ	S	37								
3675	LOC Os10g07510.1	GSSSSRGGDDDGSDGGDLQALAREL	S	237								
3678	LOC Os10g08570.1	SKGGFNVHSGATSKSPDKSKMKKDQ	S	441								
3681	LOC Os10g10990.1	PKKGKNNEEGNNSDKGEEDEEEEAA	S	240								
3684	LOC Os10g19200.1	SPAPARPRRHLPSPSPSGLPRAARA	S	17								
3687	LOC Os10g20600.1	PPAFASDPLKRVSISGDRESNGANI	S	165								
3690	LOC Os10g20600.1	FPEQESNFIRSPSPHGPSAMTRKGA	S	234								
3693	LOC Os10g22950.1	AAIGFPFLKTQSSNLSDILKDSFKK	S	370								
3696	LOC Os10g22950.1	TQSSNLSDILKDSFKKSDSFTRWMS	S	379								
3699	LOC Os10g25090.1	SLKPPPKIERNQSFDDDDDDFSNKP	S	378								
3702	LOC Os10g25110.1	KRKSPGFLSDKPSFKRQKHHKPNAG	S	771								
3705	LOC Os10g25220.1	ADGDLEVDAVLRSRMARMSLSSARK	S	90								
3708	LOC Os10g26140.1	AEHVDRTLLNTSSDSDDIIDASGAS	S	481								
3711	LOC Os10g28610.1	RKGFDVKVRKGLSFGSPEVKGSDAI	S	184								
3714	LOC Os10g29400.1	VRSLDLDLIRPISPQKMAEGKIRWS	S	108								
3717	LOC Os10g30100.1	GHLVPDDMEMSRSPPEHSSAGAKQR	S	790								
3720	LOC Os10g31770.1	KVSLENGRKSSFSDHESLYSHEEEE	S	148								
3723	LOC Os10g32550.1	KGRNVVIEQSYGSPKVTKDGVTVAK	S	137								
3726	LOC Os10g32850.1	THGTGPQFSRCISLTAQRTQLLPEP	S	393								
3729	LOC Os10g33680.1	TLDPELLQLPELSPGALRENPALAE	S	24								
3732	LOC Os10g33930.1	AKNGNCACIEFSSGELTRDMRLQQG	S	50								
3735	LOC Os10g34580.1	NLVEAKTSINLHSDQEKDEIVKPME	S	399								
3738	LOC Os10g34820.1	KNTQLDLETEGSSSPAATQKMKVKM	S	17								
3741	LOC Os10g35220.1	ATTRLPNRELPSSISPGADYMSRRS	S	595								
3744	LOC Os10g35280.1	ATEEDIVEDLILSSDEEDEDEDKNM	S	680								
3747	LOC Os10g35450.1	LVRSKSSDADNLSQCSSVYQCDRAG	S	122								
3750	LOC Os10g35580.1	PAKQEAHKRKSISDLELEEFGMASS	S	298								
3753	LOC Os10g36350.1	GRSGSELSSGNVSLRSAEMGTELGS	S	169								
3756	LOC Os10g36350.1	PMQRILDNGRSGSELSSGNVSLRSA	S	161								
3759	LOC Os10g36880.1	SAGRGRDEVGTWSLDLEDMKMELQA	S	859								
3762	LOC Os10g37480.1	DSFSRNHLGKSSSTIENFFSGRTST	S	396								
3765	LOC Os10g37480.1	LAKVQEVPLSSLSRKSPQASPLKKS	S	583								
3768	LOC Os10g37640.1	LKRFHLEEEDMDSEGEDESILSEEL	S	110								
3771	LOC Os10g38850.1	KMTGDNCTQRSYSNENACLINIRDH	S	1649								
3774	LOC Os10g38850.1	YSRIRIPDEEDSSSQKQTCRSSASQ	S	1720								
3777	LOC Os10g39440.1	DHGGDDIEDSLQSPLISRQATSVEG	S	370								
3780	LOC Os10g39440.1	GSVHEKMPEIMGSMRSTLFPNFGSM	S	319								
3783	LOC Os10g39520.1	PTPTPPERHDSFSFPRLPAHNLQQK	S	496								
3786	LOC Os10g40110.1	EELNGSSSSDDESKATSAPPARSIS	S	236								
3789	LOC Os10g40780.1	VVDALSKLAIRPSSPESLQQLIEIA	S	1813								
3792	LOC Os10g41030.1	SDRKSNYTLESVSERCIVSGRSSSP	S	213								
3795	LOC Os10g41030.1	SAKSQGEPDLKNSSPHLVIPLPTIS	S	422								
3798	LOC Os10g41030.1	KHDHAIQNLEPSSSGKGLQLDVVHS	S	1513								
3801	LOC Os10g41030.1	TDPSNMPSKTSDSPTGNIIVPAEND	S	148								
3804	LOC Os10g41030.1	SHGGDRTLVTVSSENKNGLEHGTGE	S	734								
3807	LOC Os10g41260.1	EVEGGLFIKKSSSMPNLTSIDPLPV	S	53								
3810	LOC Os10g41770.1	RPLGSAKRIGSASMDDIKSINNCEG	S	352								
3813	LOC Os10g42196.1	AVNSSVTLPRSYSLGNLANVKTPGP	S	704								
3816	LOC Os10g42420.1	VANEKTEPEVIRSPRAPTHSFKKPI	S	178								
3819	LOC Os10g42439.1	APPPAPDASSPSSPRASSASSSSSA	S	23								
3822	LOC Os10g42724.1	PAMIPVTVEPENSPRTKDGTVGKRA	S	337								
3825	LOC Os10g42724.1	NSVELLSTVLSSSPQKEALKDDLTT	S	254								
3828	LOC Os11g01200.1	TVVIAAPAKKLGSFFSEVATESAHR	S	576								
3831	LOC Os11g01300.1	AGGSGGFSGGGGSNMLRFYTDEAPG	S	56								
3834	LOC Os11g01860.1	KANVAAFGSVQISLKIIEDAGKMEL	S	453								
3837	LOC Os11g02720.1	LRRPLKAVQLSPSICRKLDFDSVSP	S	368								
3840	LOC Os11g04190.1	RKSTSSRSEAACSHGTQSHSQSMQP	S	227								
3843	LOC Os11g04190.1	KDLHMFVWSSSASPVSERAAAAAAG	S	314								
3846	LOC Os11g04190.1	NEKDGPTLSKLGSNSTAQLRPKDDG	S	368								
3849	LOC Os11g04190.1	TQSHSQSMQPRVSNLSGVEIYSLQS	S	242								
3852	LOC Os11g04670.1	RIFDTTLRDGEQSPGATMTSAEKLV	S	104								
3855	LOC Os11g04890.1	SAGANGRLPKPSSGDDELVLTPAPR	S	148								
3858	LOC Os11g06320.1	HQFSSHNDNLAHSPDHNSNVRKDLV	S	408								
3861	LOC Os11g06440.1	SSSHFRSLSWSVSRNWSAARQLQAI	S	239								
3864	LOC Os11g06650.1	GDASNSIITDGNSPEKIQGKNQQSS	S	195								
3867	LOC Os11g06750.1	PKDDVNKPCHLTSFVGYKAGMTHIV	S	45								
3870	LOC Os11g07440.1	IDKPRLTLERKRSFDEQSWSELSHR	S	37								

3873	LOC Os11g07500.1	QRVIYTSPLKALSNQKYRELSQEFS	S	139								
3876	LOC Os11g08550.1	DKLWTCTGVKQISQASCFCINKYPM	S	413								
3879	LOC Os11g09329.1	SESNRPAVNRQESLPDDDFINPTAI	S	452								
3882	LOC Os11g10420.1	PSSEDNKLLKRLSIGPKVRDAASFF	S	237								
3885	LOC Os11g11100.1	QPQPTTTLSRSNSRDLDSQFANLSL	S	334								
3888	LOC Os11g14220.1	LAALEKDYEEVGSEFDDGDEGDEGD	S	437								
3891	LOC Os11g16400.1	RRMSSSLNMSSSSLRSSGGADVDRR	S	37								
3894	LOC Os11g17060.1	KPECPWSFGQRLSSIKPLINKVENI	S	79								
3897	LOC Os11g19810.1	EKEIYGKATNTGSCASFSDVDEDIP	S	843								
3900	LOC Os11g22180.1	LGTCRKATNSTGSDASPPPQRRQRL	S	110								
3903	LOC Os11g28270.1	HPMGTVMYGLATSPTGDVSARRMLA	S	392								
3906	LOC Os11g28270.1	RMLAPVPAHSEVSPDNVSGRSRRIT	S	413								
3909	LOC Os11g29380.1	NFRPPREPRTPRSDDDGDGATPSSP	S	175								
3912	LOC Os11g31890.1	ADEDEVEHVVDTSDEEKEEGEIEEG	S	135								
3915	LOC Os11g32900.1	PSAGPDGNCYPQSPKRLCTESRNSL	S	644								
3918	LOC Os11g32900.1	APSSPSSPANGGSIKIVQMTPVTSA	S	378								
3921	LOC Os11g32900.1	PKSSPFSNLPDMSPKKVSSSHNVYV	S	870								
3924	LOC Os11g33380.1	RGSGDKVFGKKGSWDLKSFRVQAFD	S	29								
3927	LOC Os11g34190.1	ARQDSDTSEINDSHCERGDSDEDAR	S	241								
3930	LOC Os11g34200.1	ALPRRRKALPNGSDPENEESRSSKM	S	113								
3933	LOC Os11g35050.1	PCTSDNAILRRCSFGGNSMVSDISK	S	589								
3936	LOC Os11g35050.1	NAILRRCSFGGNSMVSDISKCQLTG	S	594								
3939	LOC Os11g36060.1	GETTPSEQQKEVSDTPVVPSKDNEE	S	136								
3942	LOC Os11g36480.1	APHDARKTSKRKSPLFEERRNSSHF	S	128								
3945	LOC Os11g37890.1	KAEGVDLSAYGSSKVVQTQAPVQLG	S	350								
3948	LOC Os11g37990.1	VELYAKLDSAYSSDEEDVEDEDDEA	S	755								
3951	LOC Os11g38870.1	GLTDSLPGQLRLSLPQSSQEENSSG	S	258								
3954	LOC Os11g39030.1	RSPAAAAAAVVPSPVKIAAGISGGA	S	379								
3957	LOC Os11g40080.1	LPVQDTQLRKSKSISCDSSTMEANA	S	133								
3960	LOC Os11g40090.1	NATVDDSLAAAASPSPKKKEKHSKK	S	37								
3963	LOC Os11g40140.1	DGVVLAVEKRVTSPLLEPSSVEKIM	S	56								
3966	LOC Os11g41890.1	VPKELSPGPSMRSPVGGFNYAVNRA	S	194								
3969	LOC Os11g41890.1	GGGSIYGDTTWRSGSSELDGTSPFG	S	434								
3972	LOC Os11g41890.1	TWRSGSSELDGTSPFGYGLGNAASD	S	443								
3975	LOC Os11g41890.1	RDDQHALSKSGGSAHGSPGPSRTKK	S	95								
3978	LOC Os11g42420.1	IKQEAADISDFDSLDESKLFGSGKK	S	88								
3981	LOC Os11g42420.1	ESKLFGSGKKRASLDASDAGFSSGR	S	103								
3984	LOC Os11g42930.1	ATTTDMPKFKTLSLNDSPSALDSAD	S	146								
3987	LOC Os11g43590.1	VVLQNEHCFSWRSAPDVEPLLYEDS	S	406								
3990	LOC Os11g43610.1	RAAALREVFGDSSDSEADDLPVGGA	S	25								
3993	LOC Os11g43970.1	PKAEAAPASPTASVPGRIEEDSAAT	S	43								
3996	LOC Os11g44940.1	EKDLGIGSEVGFSKNAEVARTNPTL	S	161								
3999	LOC Os11g47330.1	NIEFPLPFGRVLSSTEGFIHDLDEK	S	244								
4002	LOC Os11g47830.1	GQSHSRSYSRSHSLHLHDHARTRSC	S	209								
4005	LOC Os11g47830.1	APDGERSWALGRSPQPSPPGRSHFH	S	391								
4008	LOC Os11g47830.1	PSPPGRSHFHSHSRSRSPELRGRAR	S	406								
4011	LOC Os12g02690.1	LETTGKDLIGSNSPRSCETPSSEPL	S	183								
4014	LOC Os12g05000.1	RREEEFYASLFDSPKGGDAVKSRGQ	S	18								
4017	LOC Os12g05420.1	KVQTKPTYQRTLSGGLKSPRAADVP	S	371								
4020	LOC Os12g06520.1	SGGGRGHHRRAHSETFLRLPDADLL	S	23								
4023	LOC Os12g06560.1	PTRPQHKQYSFVSDSEEDTEKGKLV	S	961								
4026	LOC Os12g06560.1	CYRPPTLSDDDFSEDENHADQQPRK	S	884								
4029	LOC Os12g06560.1	DKPPPLPVSTSFSEKNAKTENSSQN	S	370								
4032	LOC Os12g06640.1	HHHHRQHAKNGMSDDEEHGVNEDAT	S	79								
4035	LOC Os12g06640.1	SSSSHGCSRRSDSHGISLDINKAFP	S	330								
4038	LOC Os12g06670.1	KALEASILRNEGSWSGSSHSSAAER	S	230								
4041	LOC Os12g06810.1	AHEPLKLFQKGSSFLAGNLSFSRAR	S	379								
4044	LOC Os12g06850.1	SYQNAQVSDFKYSPSPQRHNGLSSE	S	216								
4047	LOC Os12g06890.1	VDTDKHKGPLAISPTRSTSIENGIQ	S	739								
4050	LOC Os12g06980.1	NGDASLSLFDSGSPGNETDNGSFKA	S	1051								
4053	LOC Os12g07190.1	CDSRSEVSLLLASETGDGKSSIYPP	S	392								
4056	LOC Os12g07260.1	HKKDLDLTSDESSDEDTIDAIGLSE	S	116								
4059	LOC Os12g07300.1	PRLKEIDTAPLKSLDEDLDEAAREV	S	565								
4062	LOC Os12g07530.1	SPGRDHRIRRTPSPYRDRRRIPDYH	S	133								
4065	LOC Os12g07530.1	RQKHSNHSPRDASMEYVRRDPVNAY	S	443								
4068	LOC Os12g08800.1	EKQLDAERIPELSRPSPEECEISDM	S	547								
4071	LOC Os12g09089.1	WAPGATYGMKLGSPEVTFPTSYGDN	S	269								
4074	LOC Os12g10700.1	VEEALKMAGLLDSPPNSPERKNTII	S	901								
4077	LOC Os12g11600.1	VERDAEGNEEEDSGSHPSAGQKRAR	S	61								
4080	LOC Os12g13170.1	GKEKSPIKRSKGSLGSLNMITGKNS	S	153								
4083	LOC Os12g13770.1	AGLTGGTRGLTGSAWPVGRSRAARE	S	602								
4086	LOC Os12g15420.1	SPPRRHRHRHDDSPPRRRRRHDDDS	S	22								
4089	LOC Os12g16350.1	TRTAVEASSDVDSDNEDGVAGGVLQ	S	156								
4092	LOC Os12g18120.1	VDSLGFADKGSASNLENFKKYSYEI	S	198								
4095	LOC Os12g19040.1	SEKIVAEKGKSSSEPLDFDSLYPLT	S	332								

4098	LOC Os12g19040.1	PGHIRFESAGGESDKSPVKEIQTTF	S	236								
4101	LOC Os12g19040.1	DCPSSNNRDVLPSPSSNQNNSSLPF	S	189								
4104	LOC Os12g19040.1	LLKDTDSVQPAVSTPLTETGIKGGS	S	446								
4107	LOC Os12g21710.1	ASAVRPARARGSSIGGAGEDDSDGE	S	59								
4110	LOC Os12g21890.1	FSRLTSGLGLRLSPKGPQQDESAEG	S	121								
4113	LOC Os12g21890.1	DDVFSDSEAEDGSSKGRREKVSRNV	S	551								
4116	LOC Os12g22620.1	LQRRSMKRERAASPEVVGVCADEGD	S	192								
4119	LOC Os12g24080.1	PGQPDAIQEASTSDMEDASTSSGGQ	S	550								
4122	LOC Os12g24080.1	LRERFAHRYHSGSLFGMNSRGRRGE	S	53								
4125	LOC Os12g29660.1	RLEAEREMLEDESEDEEEAKESKGG	S	696								
4128	LOC Os12g32986.1	AEMNLQRDSPVSSEPIEAEVIEPEL	S	792								
4131	LOC Os12g34330.1	FLEKRNETKSLGSLGDQIADVTRDK	S	325								
4134	LOC Os12g34330.1	PSLEDVSPSHPNSDDEKLGAYVMKN	S	244								
4137	LOC Os12g34500.1	LEAMVAQLERNNSALFGEKDELAKQ	S	189								
4140	LOC Os12g35030.1	GYRNRFKDLIPQSGSPPRRRAGSPP	S	93								
4143	LOC Os12g35620.1	NDGNKGSGEENASEGVSDTLGNLKL	S	147								
4146	LOC Os12g36180.1	LKDRNGSTKDRDSSPVQNFSRKNTT	S	301								
4149	LOC Os12g36510.1	YGKKCDGLGSRKSRGPNLACPEQAV	S	127								
4152	LOC Os12g37560.1	INGGLANLAEEGSLRRIDSNAEESD	S	285								
4155	LOC Os12g37720.1	LESNAELDGELDSDSETGKEKHDDE	S	749								
4158	LOC Os12g37720.1	LGRSSLLSSNSASVADDIDDLDTKK	S	848								
4161	LOC Os12g37860.1	SNHLQTTTNRPRSAETRPGSSQLSR	S	133								
4164	LOC Os12g37890.1	AAVARVREEGEVSSGADDDEPLRAR	S	70								
4167	LOC Os12g38430.1	SYSPAPRRRDDYSASPPRKDTHPTK	S	175								
4170	LOC Os12g38970.1	LDQGESDGEKVQSSPGELSDRVMQN	S	129								
4173	LOC Os12g38970.1	DRVMQNDAAGMDSEDEAYQQRPVAS	S	148								
4176	LOC Os12g38970.1	EDEEPRQTRKASSSPVEEERDQEVV	S	196								
4179	LOC Os12g39090.1	SRLLKSCMGVAKSRGKRVMMNYPNV	S	271								
4182	LOC Os12g39370.1	DSLLDENGQDSESDEHGLVKPRYGR	S	107								
4185	LOC Os12g40010.1	SGYAFRDDDGCVSDGYDNAQDWGRR	S	710								
4188	LOC Os12g40520.1	KVDIKEIVIESSSPPDDRILDDLSS	S	504								
4191	LOC Os12g40790.1	LKRKKVNAEEVESPIAPPAKRKERS	S	78								
4194	LOC Os12g41200.1	PQSEASAERGRRSCSSEVSQLTVVK	S	1370								
4197	LOC Os12g41920.1	GHSTSTAIAAVTSDGDDDVAEPVPL	S	97								
4200	LOC Os12g42260.1	TTTAEPAAAAASSSSSPRGSDVYPG	S	38								
4203	LOC Os12g42550.1	QPPSAAAPAKESSPVNNGQLPAGAS	S	286								
4206	LOC Os12g42876.1	GAGIGPGVYDIHSPRIPSTEEIADR	S	703								
4209	LOC Os12g44330.1	SGLGEGTPKSERSADMFCDDIFGES	S	554								
4212	LOC Os12g44330.1	HHRSPDPADPAASPKRRRRHHRRRR	S	28								
4215	LOC Os12g44340.1	LSKTNGFLSKRPSFQMRSSVSTTTT	S	389								
4218	LOC Osm1g00400.1	LRNKRRSIKRNLSQRENLNSNIKSQ	S	44								
4221	LOC Os01g01150.1	SPGQHKGSKLSPTRDHHSSRRSRRS	T	657								
4224	LOC Os01g02020.1	GALLGSLSSLPATKLGSVAIQAALR	T	40								
4227	LOC Os01g05420.1	DLGDDWNRGRSPTPVHGDDGAGSSS	T	49								
4230	LOC Os01g08560.1	ETDDAPNEAASGTDVNMQEAKAPAD	T	529								
4233	LOC Os01g12780.1	DDGKLRASSRSSTQPPPRRRRRRRR	T	116								
4236	LOC Os01g14440.1	DQERGDSPDAPSTAAAWLPGRAMAP	T	251								
4239	LOC Os01g16110.1	SSRLSSSPHGIPTGSSPVGSVPKSF	T	728								
4242	LOC Os01g16870.1	VSQFIKFDEMSETSSSHGGHTSAGS	T	872								
4245	LOC Os01g19894.1	QKREFRVKVSNRTTYDVKCIQGGCP	T	848								
4248	LOC Os01g23540.1	QCDKRSLDMVEHTPSPPATAELPKK	T	770								
4251	LOC Os01g32130.1	SGKADSTKDASDTDDALEVDFNNYI	T	133								
4254	LOC Os01g32870.1	SVGAGSSSSRYPTESPENGNIDGKD	T	372								
4257	LOC Os01g36860.1	GKVGKVDLDRSPTPQDKSGPYSPAY	T	711								
4260	LOC Os01g37832.1	VYEIMPNPSDHKTKSETGGGFSHVE	T	192								
4263	LOC Os01g40050.1	KSDFRELEHDSETDDYPDDENEEDF	T	284								
4266	LOC Os01g43170.1	NVKFQSHRSRDYTNPYLPVAPSAID	T	211								
4269	LOC Os01g46710.1	EPDQGMEEVASPTIKEPDEDIEVMP	T	1159								
4272	LOC Os01g48610.1	PVTLGSAMVDGGTLTADDAFSRHAV	T	471								
4275	LOC Os01g49250.1	ADSWSVRSEYGSTLDDDQRYADAAE	T	69								
4278	LOC Os01g50310.1	PAKSYATVAAEKTVPDGSVAEDEVT	T	144								
4281	LOC Os01g53000.1	GGRPPRARRMQRTMTTPGTLAELDE	T	48								
4284	LOC Os01g53560.1	NKTCKRDHTAVGTTEESSPKKVKAS	T	105								
4287	LOC Os01g55040.1	QRPRRRLERGTSTVSQLFKGQEDGA	T	1065								
4290	LOC Os01g57280.1	IWEADSDSDIRRTLLSEHSSMKKLT	T	942								
4293	LOC Os01g58580.1	GKDDIDVGSIRMTLFNIFGDDASPK	T	254								
4296	LOC Os01g65330.1	PVRSPTQRGHTITLSPNKASSNSSP	T	552								
4299	LOC Os01g67860.1	GKGILAADESTGTIGKRFASINVEN	T	35								
4302	LOC Os01g69130.1	TASTSHLEHSLSTIQLREPPVVLKP	T	642								
4305	LOC Os01g72270.1	DSPEAKRRFLRDTVKTFVLAGKDTT	T	327								
4308	LOC Os02g01490.1	NAVAGARSPDQQTMPSPLMPGKQSH	T	1479								
4311	LOC Os02g03060.1	LARAFGIPVRTFTHEVVTLWYRAPE	T	244								
4314	LOC Os02g04270.1	AHESKDGDGAEVTKDGDDSDDSDGM	T	986								
4317	LOC Os02g05310.1	PTPGQIASRGPMTPEQYQLLRWERD	T	356								
4320	LOC Os02g05310.1	ARKLLATPTPLGTPLYAIPEENRGQ	T	418								

4323	LOC Os02g05310.1	ATPKLPGGLVTPTPKKQRSRWDETP	T	298							
4326	LOC Os02g05330.1	YLGVKVHACVGGTSVREDQRILASG	T	147							
4329	LOC Os02g09170.1	DAGDPSVAYGDSTTGSSPKTSSIDK	T	192							
4332	LOC Os02g14530.1	SHAEHKLGSISLTKQERNSGSPGSS	T	35							
4335	LOC Os02g17870.1	TLNEEANIEDVDTDDELNEAEEYES	T	249							
4338	LOC Os02g18910.1	DYVLVIYAAKSATARSFEKKVINGL	T	58							
4341	LOC Os02g20970.1	HSSPERGESESPTKRHLRKKAVHMV	T	624							
4344	LOC Os02g28980.1	MISKLSKLEDSETEGGTTQAPSKKH	T	612							
4347	LOC Os02g34270.1	GPPPPSRSVAAATTTLLGGSHGSNG	T	198							
4350	LOC Os02g34560.1	VGAGGMRRSASHTSLSESDDFDLSR	T	17							
4353	LOC Os02g38250.1	KAFVMRVEEEDDTDEDDYDSDGESR	T	71							
4356	LOC Os02g38920.1	IFDAKAGIALSDTFVKLVSWYDNEW	T	308							
4359	LOC Os02g39700.1	NAGEATESGDKCTVDALLESEKKGD	T	308							
4362	LOC Os02g43020.1	VEPEPEPEPMEVTDEEKERKERKSS	T	237							
4365	LOC Os02g44870.1	PPPAASPAAPATTPAPAHPPPATEE	T	233							
4368	LOC Os02g45070.1	AAFRARFYMEPDTSDSGSMASGAHT	T	1033							
4371	LOC Os02g47220.1	GLAREVTSVPPYTEYVSTRWYRAPE	T	156							
4374	LOC Os02g48010.1	DTEGTSKAEEPATGSKGASQSADDA	T	1066							
4377	LOC Os02g49090.1	VVGAESSMPTSFTDSYSVGSPSKSS	T	611							
4380	LOC Os02g50970.1	NKTFLSSRSTAGTAEWMAPEVLRNE	T	999							
4383	LOC Os02g53580.1	SVNKRPKGEDTVTSDGEGEAACANK	T	34							
4386	LOC Os02g53700.1	LPGGVQVHAVERTPSFSELNEILLG	T	200							
4389	LOC Os02g54254.1	VSSRDMVTPKDPTRCFNKADYYAHP	T	286							
4392	LOC Os02g57080.1	IEGARDPRFALSTGGHAQSPALQNR	T	698							
4395	LOC Os02g58080.1	DAEMELVSLNGGTPRGGSPKDPDAT	T	34							
4398	LOC Os03g01470.1	RRGPHGDGSAEPTARAAAASARRRL	T	113							
4401	LOC Os03g03460.1	KKRPARRRSQQKTDELSAVESGSNY	T	219							
4404	LOC Os03g05390.1	SEPIIRSNSVRSTSANENLRSRSVN	T	266							
4407	LOC Os03g10590.1	KHDLYEQTNRSPTPKTEEEQIAKIE	T	346							
4410	LOC Os03g11970.1	TRRTVQFVDWCPTGFKCGINYQPPT	T	349							
4413	LOC Os03g14580.1	TTAQKKRRASSDTDDDDDDFIVSKT	T	297							
4416	LOC Os03g15160.1	QNQQGTRAPRPPTPAGQPVQGRRDA	T	608							
4419	LOC Os03g16369.1	KGTDKHLPSQVRTDSSGEEEGSRAR	T	540							
4422	LOC Os03g17780.1	RQSELYREHEPATADLASDDDESFF	T	443							
4425	LOC Os03g20670.1	LNEPLLGKSSSDTGGSEVYDPRRQD	T	43							
4428	LOC Os03g21530.1	GSDLSGEEVDNDTKKSKREETNGGG	T	734							
4431	LOC Os03g22880.1	VAHANGDAEENGTPKKKKKKNREVS	T	516							
4434	LOC Os03g27260.1	IVKKGDNDLPGLTDTEKPRMRGPKR	T	127							
4437	LOC Os03g29260.1	EDDDDVDLFGEETEEEKKAAEERAA	T	112							
4440	LOC Os03g30790.1	SPSLSLSRSQQQTPRGRQPPPPGAD	T	33							
4443	LOC Os03g33110.1	TDVVVVPRSREATPSGPVGDLAPAR	T	196							
4446	LOC Os03g40550.1	KEPKKRGRRKVKTVEELSDNEGEDL	T	154							
4449	LOC Os03g46046.1	TRVRILGGQLRSTVGTGTKLREELR	T	37							
4452	LOC Os03g46570.1	QVWRDLAQSNEATANALRGELEHVL	T	217							
4455	LOC Os03g46920.1	APADQAEPVVEKTLSGSGSSEGHLR	T	654							
4458	LOC Os03g50480.1	IRERVGADGSKATGAFILTASHNPG	T	116							
4461	LOC Os03g51230.1	KMETIADVEGVDTPDEEEPVEEKKK	T	210							
4464	LOC Os03g52310.1	QEHKLALTPAVGTDNDGEGTVERPS	T	137							
4467	LOC Os03g53630.1	PSLKKARDTSSDTELEQPKMSSGKK	T	368							
4470	LOC Os03g54780.1	PDMQEAKTMPPSTGTVKKLKVAEGT	T	663							
4473	LOC Os03g55070.1	KFDWDHPMHLQPTSPTAFKQVSVVW	T	392							
4476	LOC Os03g61010.1	HALLGIQHDKENTERISLSAIAKVY	T	331							
4479	LOC Os03g63670.1	SANNEHIEGELSTDESDSESSAYLS	T	616							
4482	LOC Os03g64320.1	IRDGDPGGKSPPTLEVTDGKKADVE	T	1000							
4485	LOC Os04g04254.1	SPADDRGLPRSSTMPGGINNVEITN	T	55							
4488	LOC Os04g13960.1	GTVGRRVGEADVTEVEDEDGEVFGR	T	144							
4491	LOC Os04g21220.1	FAGSVWGFQMVTTRRNVNTGEGNQP	T	308							
4494	LOC Os04g24210.1	WIKKFVSQLGVMTSTSRPMDLYCDN	T	619							
4497	LOC Os04g28090.1	TATRALLSSYSQTPRLGMTPLRTPQ	T	342							
4500	LOC Os04g28460.1	TGNQTKSGMSRGTAEPKLTAADAMI	T	244							
4503	LOC Os04g34450.1	AAATRKALTNVQTLPRGVEVLDPLG	T	174							
4506	LOC Os04g35800.1	PRKRRLSPTPSPTRRSSRSRSRSPR	T	60							
4509	LOC Os04g37640.1	DCQARTKGDNGKTVRHVRHCEDLKD	T	568							
4512	LOC Os04g39240.1	TSTRSFAFPVLQTEWNSSPVKMAKA	T	631							
4515	LOC Os04g42320.1	KKQKENLKQQEDTPPDEDTDEDLSL	T	528							
4518	LOC Os04g42320.1	EDILVSRELSPETDDGDNKLPPETG	T	421							
4521	LOC Os04g49950.1	CSGGGSRTLVSTTGAGGGGVRRGKV	T	146							
4524	LOC Os04g52460.1	QVKEAHNDEDDGTGSDGFELIDVKE	T	55							
4527	LOC Os04g52960.1	SSFKKPAQSSGNTIFIKGFDTSLDI	T	550							
4530	LOC Os04g54564.1	GHDERRRRLASSTVGFPPRYLRHWP	T	66							
4533	LOC Os04g56620.1	PSHLFPSAKSVSTDLSQVPASQNTE	T	242							
4536	LOC Os04g56740.1	AQLKFEELTSNLTVSRSNSLDGANK	T	563							
4539	LOC Os05g01990.1	DEQNSSAHGKNHTSLLSDDEEEILE	T	263							
4542	LOC Os05g03120.1	GGNETDSAGITPTRTTPERSPKVTE	T	43							
4545	LOC Os05g03460.1	AKNTEPPKRIPITAKAERSFTFREL	T	71							

4548	LOC Os05g05300.1	SVFRESEDDELDTEDDELWGPDLKE	T	500								
4551	LOC Os05g08840.1	DTDDALGDPASGTDENMQESKCSAD	T	528								
4554	LOC Os05g11770.1	ESVMMLGGSKSDTELDAEPERTAGE	T	761								
4557	LOC Os05g14940.1	QEHKLLLTPAGGTDNDGEGTVERPS	T	137								
4560	LOC Os05g23610.1	HPMVDDDGSLSPTRPFDKCLDETVN	T	46								
4563	LOC Os05g28190.1	KDADNKGQSSSPTPLFSFKNLSSGQ	T	250								
4566	LOC Os05g32360.1	ARSFSNDHNGNLTNRVITLWYRPPE	T	198								
4569	LOC Os05g34790.1	VDLDSDGSEDHGTRQHEAKPKLCSG	T	164								
4572	LOC Os05g38810.1	ELTEKKSTSNLSTDNAYDHPAERQM	T	496								
4575	LOC Os05g42230.1	HPASPVAGGEKATPPSTPPPVRLAG	T	16								
4578	LOC Os05g43060.1	DNWGEACKETEKTGSDTDPWGSKVK	T	793								
4581	LOC Os05g44320.1	DGVLDFLNSKKMTDAGALDLIDDED	T	668								
4584	LOC Os05g48820.1	ERTYTEDPYPNETMRAELSVKLGLT	T	84								
4587	LOC Os05g50480.1	VAPAADEHVASSTKSDTENVDVAPV	T	486								
4590	LOC Os05g51830.1	TGKRKVAEIVLKTPSSDKKAKIATP	T	216								
4593	LOC Os06g02510.1	PRRARKVKAGDSTPEELATATQVQG	T	139								
4596	LOC Os06g04660.1	LNIIPLETAVQETGRSSDEGKGKQP	T	430								
4599	LOC Os06g05660.1	DEDEPILEKAIGTEIEWYPGKNLTQ	T	214								
4602	LOC Os06g06090.1	GLARTTSETDFMTEYVVTRWYRAPE	T	225								
4605	LOC Os06g08480.1	ATEVPSSASKEATPPVQDPVIEAVK	T	1222								
4608	LOC Os06g11610.1	ESGTDSGDEYDATDDGRRLTVPFFF	T	88								
4611	LOC Os06g13820.1	YANDISEAESPRTPNRPGEDWRSAF	T	847								
4614	LOC Os06g17840.1	VEGAKKRDDSVDTELVTSPASVKTP	T	1275								
4617	LOC Os06g22820.1	QVPESKDEDDDSTEEWANFRGGDSD	T	444								
4620	LOC Os06g30570.1	VNENERTNVNEPTNVNENERTNMNE	T	15								
4623	LOC Os06g34710.1	EVTTPATEKVEVTTPATEEAKITTA	T	895								
4626	LOC Os06g34710.1	EVTTPATEKVEVTTPATEKVEVTTP	T	885								
4629	LOC Os06g36710.1	DRKHPADSRREHTPPRTPGDRRRSS	T	392								
4632	LOC Os06g45980.1	KLSQAKNNTECETDQDGEANLAVNK	T	184								
4635	LOC Os06g49800.1	SDTEKIHGQASDTEISSELTTAASQ	T	433								
4638	LOC Os06g50890.1	REREPDAVNHGNTLYVTGLSSRVTE	T	66								
4641	LOC Os07g01930.1	MDVNYYNKLEDPTDEENDMLDLAFG	T	128								
4644	LOC Os07g03980.1	LSLESTKFKRSFTLPARMSSSTTST	T	122								
4647	LOC Os07g05610.1	GGMGRDLMIGQPTGSELGGFAASFE	T	466								
4650	LOC Os07g08190.1	FTQQYAFARRYRTPSPERSPVRSRY	T	461								
4653	LOC Os07g10350.1	KKSYIGPDSTGDTSDDEDDEIVPEE	T	513								
4656	LOC Os07g13340.1	GAKPKASNEANTTRMVNQNNVPMLG	T	1078								
4659	LOC Os07g25460.1	KFEELQPLEEFTTPPSSPDNSKSPV	T	574								
4662	LOC Os07g31370.1	FLNTSKWIEEVRTERGSDVIIVLVG	T	109								
4665	LOC Os07g34740.1	LLLQGDKSEEEQTDGEDMEITFNTE	T	404								
4668	LOC Os07g36940.1	ESEQTNAASASPTEMLKAADASSID	T	694								
4671	LOC Os07g38730.1	SLRFDGAINVDVTEFQTNLVPYPRI	T	253								
4674	LOC Os07g40270.1	LGSLEGFFPSDHTSSPGNKQIILQG	T	235								
4677	LOC Os07g42300.1	VKVESTAVPSASTPDVADAKAPAAD	T	89								
4680	LOC Os07g46750.1	EDDDDLDLFGDETEEDKKAADERAA	T	111								
4683	LOC Os07g47920.1	PGGVFPNYRTLITDKQPVIQSALLS	T	83								
4686	LOC Os07g49380.1	SDEDWDPRKKGATDSDSEVDFDRKR	T	260								
4689	LOC Os08g01930.1	NESSAKVDEPVATENSDAAPAEASV	T	97								
4692	LOC Os08g02690.1	AGGKGTWGKLIDTDTDACLDRNDPN	T	103								
4695	LOC Os08g08080.1	KESQDISPPRRRTRHDSEEPQDLSP	T	233								
4698	LOC Os08g13350.1	GKKSSGPQSGGVTPMARQNSGPLPP	T	137								
4701	LOC Os08g19320.1	AGDTSAAAAVAPTPARLREALAALS	T	35								
4704	LOC Os08g25090.1	RGTKRTAFSDSDTEDLDLSKEDLTK	T	139								
4707	LOC Os08g27040.1	ADEGHGAISLRDTSPILDTMESGRR	T	297								
4710	LOC Os08g28880.1	LTVDPALGRRQATAAAAALPTPRSP	T	36								
4713	LOC Os08g33370.1	LRDNLTLWTSDLTEDGGDEVKEASK	T	237								
4716	LOC Os08g37280.1	KELSHVNSPCDSTEEAGNLKAKTSS	T	122								
4719	LOC Os08g40230.1	VLERKLADVHSETDDELIEELRSKP	T	91								
4722	LOC Os08g42600.1	VYVSPLRTSKMDTLLSPSSKSYYAC	T	925								
4725	LOC Os08g44420.1	QSSYKDYEPTGETPVRSEPEVPSKD	T	353								
4728	LOC Os09g07170.1	ASSSKAPKKTKMTFIMLLNKYEKQG	T	260								
4731	LOC Os09g16980.1	GMCTRMTGQDRPTMREVEMGLENLR	T	584								
4734	LOC Os09g23790.1	TSAPTAPRQRIATCDEVSVKEEDAL	T	780								
4737	LOC Os09g28220.1	LRKSMKRCIWNITPSPPRREGEDED	T	144								
4740	LOC Os09g33510.1	HQPSIHVRVSPNTSPPLLSSPKIKE	T	489								
4743	LOC Os09g34070.1	FQRSRGRAEWLETGSFNGRTGPAKG	T	205								
4746	LOC Os09g36160.1	RPRARPSATTPTTSSGDQQMVTVAE	T	180								
4749	LOC Os09g37520.1	PADIPSTSRGEVTPPAPPSPPRVST	T	351								
4752	LOC Os10g08570.1	SSKGGFNVHSGATSKSPDKSKMKKD	T	440								
4755	LOC Os10g30350.1	FKIELFRTTEERTTPNAIPINDTDA	T	200								
4758	LOC Os10g32550.1	LLYASKELDKLPTANFDQKIGVQII	T	522								
4761	LOC Os10g40780.1	LEMYQQVSQKLDTLIAKDGKDAEIQ	T	1654								
4764	LOC Os10g42724.1	PGRDDESLAPIFTPPRSAPSAEPYS	T	195								
4767	LOC Os11g06650.1	GGGGNCGAAAATTPSPRGAAVAEGE	T	74								
4770	LOC Os11g10480.1	HTDVYFWEAKGQTPVFPRIFGHEAG	T	60								

4773	LOC Os11g26130.1	WRCDDFTISDSDTFGSFNVPRKYNG	T	900								
4776	LOC Os11g29380.1	QPKGANLEDRVPTDVEDDPLAAARQ	T	750								
4779	LOC Os11g32900.1	KYHPLQATFASPTVSNPVSGNEKCA	T	691								
4782	LOC Os11g34660.1	CSYIRRKRLARKTSIPGSSSKWANH	T	191								
4785	LOC Os11g37100.1	SASPNGATSRDKTPSPQASERPISI	T	445								
4788	LOC Os11g43590.1	RADDLAWRCSSDTFDLNGRAFENSE	T	71								
4791	LOC Os11g47830.1	NDRRDADNGYDQTPPAPDGERSWAL	T	376								
4794	LOC Os12g05420.1	VPKVQTKPTYQRTLSGGLKSPRAAD	T	369								
4797	LOC Os12g06560.1	IHNDKPPPLPVSTSFSEKNAKTENS	T	367								
4800	LOC Os12g06890.1	TDKHKGPLAISPTRSTSIENGIQRD	T	741								
4803	LOC Os12g07260.1	LDLTSDESSDEDTIDAIGLSEVEAD	T	120								
4806	LOC Os12g07530.1	AMYRDHVTEHYQTDDSPLGREYYDD	T	598								
4809	LOC Os12g10410.1	VLGVWGMGGAGKTTLLKLARDPRVQ	T	219								
4812	LOC Os12g19040.1	FVRHEADEEESDTSDDIVPVVVRPG	T	213								
4815	LOC Os12g24080.1	RPGQPDAIQEASTSDMEDASTSSGG	T	549								
4818	LOC Os12g36510.1	TDCANIGRAMVNTGDDRSIPPLAGD	T	576								
4821	LOC Os12g40010.1	AGLSMGRRVSFSTEANIIADRLHRA	T	679								
4824	LOC Os12g43500.1	YSTEPPDDGKCVTKEDLESDEAVWA	T	92								
4223	LOC Os01g01270.1	QHIRAVNAILRETPYDPVLNDDLAR	T	312								
4226	LOC Os01g04650.1	SPMIDKNSSFGSTSSAPSLSNLPPI	T	280								
4229	LOC Os01g06630.1	ARDDGKTVIKSGTGGKSLDAQERLA	T	36								
4232	LOC Os01g12660.1	TSSFPKLKRNASTSSDMSSLASQGP	T	217								
4235	LOC Os01g13470.1	DKKADDSSVAKDTNSEPEAQLELEK	T	185								
4238	LOC Os01g16110.1	GPGAPASPWRKTTPPPVAGEAVVMG	T	40								
4241	LOC Os01g16500.1	ELPEDDAEAEKITRQAKIYYMIGND	T	1459								
4244	LOC Os01g17320.1	VPPEADKKDLDLTSDESSDEDTIDA	T	111								
4247	LOC Os01g20940.1	VCPICSKNVGLSTSSLKLHLQKAHK	T	868								
4250	LOC Os01g28660.1	RQAWRREAQPMATEAGSARETWAAE	T	168								
4253	LOC Os01g32720.1	NIDDISRKFSPGTDFEVNRQHFTAP	T	851								
4256	LOC Os01g34330.1	MGRRRRFAQLATTSDDDDDDAPAAD	T	13								
4259	LOC Os01g37080.1	PLCSDPDRASILTIMMAVGASKERA	T	297								
4262	LOC Os01g38950.1	LQSSEAEPSRSRTPDASPGTFQRSA	T	412								
4265	LOC Os01g42960.1	GRSHSGELTAESTPPRLPGHRRCGS	T	90								
4268	LOC Os01g43920.1	NKTVFKSAGIMSTLIVSSSKSNVVH	T	310								
4271	LOC Os01g48320.1	LPYREDCWSDGETAALVDAWGRRYV	T	24								
4274	LOC Os01g49190.1	LGRIPSAVGYQPTLATDLGGLQERI	T	362								
4277	LOC Os01g50310.1	PAKSYAAVVAEKTSPNGSVAEDEVT	T	82								
4280	LOC Os01g53000.1	RPPRARRMQRTMTTPGTLAELDEER	T	50								
4283	LOC Os01g53000.1	LAELDEERAGSVTSDVPSSLASDRL	T	67								
4286	LOC Os01g54590.1	FTKNEFCLESKSTIGVEFATRSIQV	T	50								
4289	LOC Os01g56140.1	QKRLNHLVSCDQTEVLEPKRKHRRS	T	848								
4292	LOC Os01g58400.1	GRGKTIAPDIAPTGSDNEDREEGNA	T	264								
4295	LOC Os01g62890.1	SFRTPNLKARLNTFREEFRAVWRNY	T	194								
4298	LOC Os01g67250.1	QVNNVEGVTEQLTDNEKETAVVEKA	T	778								
4301	LOC Os01g67860.1	KDELIKNAAYIGTPGKGILAADEST	T	21								
4304	LOC Os01g70810.1	LFAMKDTIGKKETREISASFGVTVT	T	103								
4307	LOC Os01g73230.1	HQDMGTSRSNHYTAQDNDDFIASES	T	126								
4310	LOC Os02g03030.1	NIARFINGINNHTPDGRKKQNLKCV	T	308								
4313	LOC Os02g04050.1	DEYYLDGKHVSKTEVMNLLESAGFS	T	119								
4316	LOC Os02g05260.1	DRRKFRELDKTVTGQVRFGDASSVQ	T	377								
4319	LOC Os02g05310.1	QPIRTPARKLLATPTPLGTPLYAIP	T	412								
4322	LOC Os02g05310.1	WDATPKLPGGLVTPTPKKQRSRWDE	T	296								
4325	LOC Os02g05310.1	PSVRRNRWDETPTPGRMADADATPA	T	258								
4328	LOC Os02g08500.1	SRVHSSSADPFSTLVGESPQFPDLG	T	424								
4331	LOC Os02g13840.1	QQVYTGVWLRHYTPVRERVPASQGE	T	468								
4334	LOC Os02g17320.1	DRYFGTDDYQSSTPFFCDEKNRVRL	T	476								
4337	LOC Os02g18660.1	EEEEEEEDSDTDTYAQGFQLRKVGG	T	74								
4340	LOC Os02g19804.1	VRKPPKNIRKRPTAPAGSDDDDEDG	T	34								
4343	LOC Os02g26140.1	VQVAAAAASVSKTRRCTFSPSRLVS	T	320								
4346	LOC Os02g33770.1	MNKMLSEGSGGPTAEKSPSTPGMRD	T	325								
4349	LOC Os02g34270.1	PPPSRSVAAATTTLLGGSHGSNGAD	T	200								
4352	LOC Os02g37880.1	EKSLVSAMESRGTGYYEEKKALLIL	T	123								
4355	LOC Os02g38840.1	ADELSKRMGYVQTHGYVWIPPTLSK	T	504								
4358	LOC Os02g39210.1	PLPRRVKDKEAMTMAQKEQLVGLLS	T	66								
4361	LOC Os02g42210.1	KTLELEDPSSDETECMAEASTSEEM	T	438								
4364	LOC Os02g44810.1	KMSSSSDPDQEPTSPSAAGADGAQP	T	29								
4367	LOC Os02g45070.1	TSDSGSMASGAHTRGGGPLPGARST	T	1045								
4370	LOC Os02g47130.1	VEIPQADLTGDKTSEEEDLNEVKEG	T	399								
4373	LOC Os02g48010.1	EVKKDDGQGDSVTVGGTRKRRFAGA	T	965								
4376	LOC Os02g49070.1	ETQDFLKPSLRITSKGSFDATNAVK	T	142								
4379	LOC Os02g50960.1	RNPTPRGSSFNHTDFYSMVGRSSNF	T	259								
4382	LOC Os02g52780.1	VGAPPVQRQGSLTLPRTLSQKTVDE	T	100								
4385	LOC Os02g53580.1	CANKRLKVEATVTSDGAVVRQRHEA	T	55								
4388	LOC Os02g54110.1	DTGELPQTPRSATGDDELSLMDGEP	T	17								
4391	LOC Os02g56530.1	KFEELQPLDEFATPPSSPTQFQDAK	T	531								

4394	LOC Os02g57390.1	RSTTPDPQLIRRTPSPCLPPVGVRM	T	303							
4397	LOC Os02g58110.1	RKILDKLRSYQGTPFIEGLMGSQDD	T	56							
4400	LOC Os03g03034.1	YSDDPAKKIRLSTSFNVRKETVHNW	T	110							
4403	LOC Os03g04710.1	TIQSFTQRGGASTDEEEEEDDDDGD	T	118							
4406	LOC Os03g06340.1	PPPQPQQQPRVETPPPPSYGFHNLD	T	95							
4409	LOC Os03g11580.1	LLESKSPDGVGQTFRAQPGVKLGIS	T	424							
4412	LOC Os03g14260.1	GDIDGAITPLARTTSSAAAGAGGAA	T	282							
4415	LOC Os03g15050.1	LHSLQRKRSAPTTPIKDGASSPFAA	T	54							
4418	LOC Os03g15810.1	GADQPRRSPQETTAAPSKPADEVSE	T	58							
4421	LOC Os03g16910.1	FTGVMRHASGPVTAAKTIYEDSKGY	T	409							
4424	LOC Os03g19340.1	STQDREGLALSPTNREAVETKPNFE	T	948							
4427	LOC Os03g21270.1	PKTNSILSAQSTTGSFNSTNSDRLR	T	1029							
4430	LOC Os03g22340.1	VTVSRDKTKVTVTSDGPFSKRYLKY	T	76							
4433	LOC Os03g27260.1	KVSKAPKIQRLVTPLTLQRKRARIA	T	185							
4436	LOC Os03g27840.1	VEYITEFGGSDDTSDLKVAGISPPS	T	351							
4439	LOC Os03g30460.1	IVDGEVNDEYMKTLETLSRKIKFIE	T	184							
4442	LOC Os03g32630.1	EEEDDADLVAMATKPKAARATVDLN	T	79							
4445	LOC Os03g40110.1	ARDWEQSDDDEVTVEEDEMEGLKQK	T	445							
4448	LOC Os03g43800.1	DQLEDDREACSGTEEGHSAKKTKDE	T	440							
4451	LOC Os03g46046.1	LGGQLRSTVGTGTKLREELRSRRSR	T	42							
4454	LOC Os03g46710.1	EGTRSTLSRPSFTDPLVTAERLHSP	T	859							
4457	LOC Os03g50330.1	HKSAEDPDGSFYTGDDPDRSFYDRD	T	52							
4460	LOC Os03g51020.1	RHHKLDDAQEEDTGTTLDDEEAAAL	T	744							
4463	LOC Os03g51600.1	TKRTIQFVDWCPTGFKCGINYQPPS	T	349							
4466	LOC Os03g52490.1	PRDLDKNSPGYQTPVYVEQPRQQPR	T	126							
4469	LOC Os03g54780.1	YLDEHPDMQEAKTMPPSTGTVKKLK	T	658							
4472	LOC Os03g55070.1	WDHPMHLQPTSPTAFKQVSVVWDAY	T	395							
4475	LOC Os03g60530.1	SIARDIKQRLSETDSKPEDRTIKIK	T	184							
4478	LOC Os03g63650.1	GSPAATPSPAPLTPDRVTSWSAFVR	T	186							
4481	LOC Os03g63940.1	DFDQINTILSPSTPGSRMNMDVDND	T	117							
4484	LOC Os04g01490.1	SSVKFFNARISRTSSLPVTDVSQEQ	T	154							
4487	LOC Os04g09860.1	DKDADVDPLQRSTPDDTEPNSEENM	T	161							
4490	LOC Os04g21220.1	LFAGSVWGFQMVTTRRNVNTGEGNQ	T	307							
4493	LOC Os04g23330.1	IYGSRRRREPSSTRYRETLPPDKAI	T	100							
4496	LOC Os04g26660.1	RIDDLFDQLKGTTVFSKIDLRSGYH	T	925							
4499	LOC Os04g28090.1	RDGSSFGLTPKSTPFRDELRINEEV	T	443							
4502	LOC Os04g33110.1	GRFHGHGHHSSGTTPTGAAASPKMR	T	25							
4505	LOC Os04g35800.1	PPLPPRKRRLSPTPSPTRRSSRSRS	T	56							
4508	LOC Os04g36890.1	AHQNTDKTKHIHTDAEEDTAQEASK	T	353							
4511	LOC Os04g38870.1	NLTLWTSDISEDTAEEIREAPKRDS	T	246							
4514	LOC Os04g42320.1	LKQQEDTPPDEDTDEDLSLKDIVSP	T	534							
4517	LOC Os04g42320.1	SDHNLKKSHKLDTSTDSEVVDHSKA	T	392							
4520	LOC Os04g49560.1	SSFARHYKGAWCTPPRIFIDQDEII	T	327							
4523	LOC Os04g52460.1	KELHISKLERQITLSELKYMEEIQT	T	1528							
4526	LOC Os04g52820.1	LSVVEKEHPRSLTPMLWAKKIYSNL	T	168							
4529	LOC Os04g53670.1	KLSRQNSFGSIGTPRTPNLHSTTDA	T	1133							
4532	LOC Os04g56160.1	EEREAQWATAQRTLHGLQPPEVASN	T	881							
4535	LOC Os04g56720.1	ASTNARSNKRKKTKDVSESEEDDDE	T	461							
4538	LOC Os04g59570.1	RPLRSRRALSVFTCAAPPRQRPPPG	T	28							
4541	LOC Os05g02680.1	TASDAKKQKTDSTASGEDKDSEDPA	T	1236							
4544	LOC Os05g03120.1	ETDSAGITPTRTTPERSPKVTERRS	T	46							
4547	LOC Os05g03740.1	KRRRKAAAAARRTGSTGGVEGEVEA	T	233							
4550	LOC Os05g08370.1	SYMDSKNRMMKRTESSAPIFNMEDI	T	681							
4553	LOC Os05g11770.1	PSLFFPKQYFSDTEEFEDVIYPKGD	T	282							
4556	LOC Os05g12100.1	RGSVQHCRHAQHTTSTPRRPACPEG	T	131							
4559	LOC Os05g22580.1	DTGDAVLPASRITADRHLSLPCKIM	T	16							
4562	LOC Os05g24160.1	AATLQPKEEAMKTGCVALVLCLNIS	T	364							
4565	LOC Os05g28280.1	RVTSHSLQELQPTKSIEDNAEIKAW	T	342							
4568	LOC Os05g33310.1	STPVDLSPLPSPTKAMTLWQAPPPQ	T	249							
4571	LOC Os05g37480.1	DEGGRVTARGGWTGWGTGAAVSMRQ	T	104							
4574	LOC Os05g40230.1	AGKSLLRVTKDATAYRKVLKGLIVQ	T	108							
4577	LOC Os05g42300.1	GYDGSGENDEDQTDPEFSDDEKEAE	T	374							
4580	LOC Os05g43520.1	KSLAIEPKIHSETDEDEVDEWPDDD	T	377							
4583	LOC Os05g47470.1	DAEDEDEAAAPATPSKEAEVGYHGL	T	176							
4586	LOC Os05g50480.1	ADEHVASSTKSDTENVDVAPVQQPE	T	490							
4589	LOC Os05g51690.1	YSEGDIQNLGASTPRPGNSGNAQLA	T	228							
4592	LOC Os06g02180.1	IMRSQKAMTRSQTGDWDHNRWLFET	T	242							
4595	LOC Os06g04660.1	DDPELLNIIPLETAVQETGRSSDEG	T	425							
4598	LOC Os06g05520.1	ESTIGKFLTQSGTFKDGDLLVNKDG	T	25							
4601	LOC Os06g06014.1	WPLAPSPPASPTTWHDNKAASSPAQ	T	88							
4604	LOC Os06g07160.1	TGLGSTDPSSSSTPRKRASSGRSYK	T	269							
4607	LOC Os06g11610.1	PLRRYDVVDESGTDSGDEYDATDDG	T	79							
4610	LOC Os06g13680.1	AEHGLRNYVRDKTPEIMPAINKFFT	T	56							
4613	LOC Os06g17840.1	DELLLQSVDPDETINSFENKVEGAK	T	1255							
4616	LOC Os06g19660.1	LLKHPSGAGDENTVSEAPEKTSDGY	T	845							

4619	LOC Os06g27970.1	AFGRDFNSSPSPTAPPARSGEKRPF	T	26							
4622	LOC Os06g30750.1	VRSRGFRLLGEDTSVHKALGGGKTA	T	69							
4625	LOC Os06g34710.1	PATEKVEVTTPATEEAKITTAATEE	T	899							
4628	LOC Os06g36360.1	PQQSNKNDVEDGTANRSPDGQPKRK	T	521							
4631	LOC Os06g43610.1	QVAENDDAKEIDTDPEDADNLNTKK	T	306							
4634	LOC Os06g49500.1	LDPLLVEDEDPPTPKTQESSFTGSI	T	399							
4637	LOC Os06g50890.1	VEKSRRGRPRTPTPGSYLGHRYDRR	T	150							
4640	LOC Os07g01020.1	AALLEPSNHKSATFSVKVGLAQMLR	T	31							
4643	LOC Os07g03230.1	DDDVEDKDDDDETDDDASDADIEDP	T	194							
4646	LOC Os07g05610.1	MRKGVLTRGISATSGINGWVAEGTE	T	392							
4649	LOC Os07g08190.1	KHSSKKDPDNTKTISLEKDSTLEDA	T	341							
4652	LOC Os07g09384.1	PPPEPTLARIESTESFSLPLHKVDG	T	233							
4655	LOC Os07g12910.1	VDDGYDEEEHSETLCGTCGGRYNAN	T	188							
4658	LOC Os07g22750.1	KTLTRAWLNYASTEKELLAVVFAID	T	309							
4661	LOC Os07g28800.1	GNFRSRGGFEFCTSDEDEPETVFRN	T	143							
4664	LOC Os07g32530.1	QERSLESDQYDGTDSEEEVASASSR	T	727							
4667	LOC Os07g36420.1	KDAGAAKNPEVETSSSIHRSENPGL	T	167							
4670	LOC Os07g37750.1	PVKAPDADGADKTDDEENPMMETVR	T	1457							
4673	LOC Os07g39620.1	FSTDNSTADSAGTFRSDTPVSSVST	T	266							
4676	LOC Os07g42300.1	DDDDDVDLFGEETEEEKKAAEERAA	T	115							
4679	LOC Os07g43316.1	TIPDYLEGKSEVTLEHVSSGDESME	T	241							
4682	LOC Os07g47360.1	SQRRKQNFHVAKTSPIESVSSSPPR	T	1088							
4685	LOC Os07g49270.1	APWEKEIITDPSTPKPNPNPFYYEQ	T	256							
4688	LOC Os08g01620.1	RTLVQPVGQKRLTNVAVVRLRKHGQ	T	15							
4691	LOC Os08g02690.1	SLASPRSPTGGSTSALLQQYEQQRA	T	41							
4694	LOC Os08g07830.1	DIAKLLEAGQEATARIRVEHIIREE	T	61							
4697	LOC Os08g08220.1	SRQQFKAYAAAGTLKQNYANILLML	T	692							
4700	LOC Os08g19310.1	ESRNVALNEYSYTAMIKALCKAGKV	T	225							
4703	LOC Os08g24760.1	TEEVDDEDDGNDTDEEQERIRDQQE	T	171							
4706	LOC Os08g25850.1	KIDSETKSNQPPTKKASSKPAPKKN	T	45							
4709	LOC Os08g27710.1	VAATTTTPTAAATKTTSDRPHRRRH	T	25							
4712	LOC Os08g29650.1	QSKEVRYTARSITPPADRNGTSKSK	T	15							
4715	LOC Os08g37104.1	KRVTNVKQTPRRTIPSMNHGGDRPG	T	179							
4718	LOC Os08g39390.1	LREALPLLSLSPTPARRGGVVDAAA	T	101							
4721	LOC Os08g41710.1	VRKYLSPEDWSPTETKGRPWYGLLA	T	300							
4724	LOC Os08g43090.1	SPGQPPVVPRSPTPLDLSSAAAAAA	T	29							
4727	LOC Os09g01640.1	RSKHRRSTKRSDTEDDLEEERSKDK	T	239							
4730	LOC Os09g07900.1	RDGIGSVWSSSGTPSRDLHTFSFGT	T	2230							
4733	LOC Os09g19952.1	VLDEIRWRYYEETVMILNLMPYRAS	T	21							
4736	LOC Os09g26830.1	SKNGWSLSSSPDTKATKNVTVTSFD	T	511							
4739	LOC Os09g33450.1	SIDGGSDMGNGQTPQQLRLPRTKDV	T	130							
4742	LOC Os09g33600.1	TLGKALLLQEQPTSPSDGSSRCFDR	T	701							
4745	LOC Os09g36090.1	VIRDNDLSEPHDTNDEEEGNSDSSS	T	637							
4748	LOC Os09g36160.1	KRPRARPSATTPTTSSGDQQMVTVA	T	179							
4751	LOC Os09g39870.1	LAMRDCMENGFLTDDGVVHTVFDGN	T	616							
4754	LOC Os10g26140.1	NLAEHVDRTLLNTSSDSDDIIDASG	T	479							
4757	LOC Os10g31520.1	EDTATPYESAKSTSNNDEIADMAKS	T	342							
4760	LOC Os10g39200.1	DSVSGLDSPGDATCSDEDNGRKAPS	T	1016							
4763	LOC Os10g42110.1	LMKNSRDGKSYSTNLAYTPPEFLRT	T	234							
4766	LOC Os11g03360.1	SGERKLWLALWKTKVQAKIKIFAWK	T	193							
4769	LOC Os11g08440.1	ERMAREARSYNRTRSSLAITSGNLV	T	562							
4772	LOC Os11g19250.1	FTEKFDEETIGGTEMADDENLTKEQ	T	447							
4775	LOC Os11g29380.1	HRANFRPPREPRTPRSDDDGDGATP	T	172							
4778	LOC Os11g31890.1	KSNFASDDLPSPTPSGDGDKSGDKG	T	459							
4781	LOC Os11g34190.1	VDKVFSGSDDDNTNNNAPEKDEAKD	T	79							
4784	LOC Os11g36390.1	DGEEDKMDEDAKTPSKAAGRGRGGA	T	212							
4787	LOC Os11g42930.1	LGATTTDMPKFKTLSLNDSPSALDS	T	144							
4790	LOC Os11g47830.1	QAKEHDEKRRSYTPEYNDRRDADNG	T	360							
4793	LOC Os12g02690.1	SVADEDPSYGCQTPRESIFDPFAPG	T	92							
4796	LOC Os12g06560.1	QTVDKEMCYRPPTLSDDDFSEDENH	T	877							
4799	LOC Os12g06870.1	VPFFADDEESPATPKADAFFIPREN	T	701							
4802	LOC Os12g06980.1	PLKRSRRKSVVSTLLEEGTKCMHGS	T	527							
4805	LOC Os12g07530.1	TPSPGRDHRIRRTPSPYRDRRRIPD	T	131							
4808	LOC Os12g09880.1	SSSSGRLSAAGSTASGGSGGGWERR	T	26							
4811	LOC Os12g13770.1	SDRHCTAGLTGGTRGLTGSAWPVGR	T	596							
4814	LOC Os12g21890.1	GPQQDESAEGSTTPTTAQSGVFGSF	T	136							
4817	LOC Os12g30020.1	QVTGNGHLGKQNTPETAQDAPMDDI	T	498							
4820	LOC Os12g38730.1	EDGSARKLKKVPTWERILKSREPNR	T	574							
4823	LOC Os12g42520.1	MPNRDLDKWLHPTIDDDDESFSRVL	T	840							
4826	LOC Os01g01150.1	RPSSPAQASRDAYSDADDDDDDRPH	Y	66							
4829	LOC Os01g03760.1	DAARIAAATASAYSDDDDDSDEAQA	Y	101							
4832	LOC Os01g10840.1	AKVLVPGEPNISYICSRYYRAPELI	Y	225							
4835	LOC Os01g29590.1	NTIPCIPAHGVIYAFRGGVRRNRML	Y	182							
4838	LOC Os01g43170.1	FQSHRSRDYTNPYLPVAPSAIDGSG	Y	214							
4841	LOC Os01g50320.1	RKKHHPESDEENYDSEESYKHSRKK	Y	730							

4844	LOC Os01g57790.1	TVQGALSDQLGNYVEAYVNDIVVKT	Y	69							
4847	LOC Os02g05310.1	LLATPTPLGTPLYAIPEENRGQQFD	Y	421							
4850	LOC Os02g08360.1	EDRKGGRGLGLSYSSGSDIAGDSGK	Y	789							
4853	LOC Os02g18660.1	EEEEEEDSDTDTYAQGFQLRKVGGG	Y	75							
4856	LOC Os02g28520.1	GDEGGITLVQSHYVDKVLSRFGYSD	Y	699							
4859	LOC Os02g33360.1	FMQTRKGTVVQKYKVKVVADVPGTG	Y	77							
4862	LOC Os02g37880.1	LVSAMESRGTGYYEEKKALLILRDV	Y	126							
4865	LOC Os02g46090.1	FRGASQRHDEAGYAPVATSAAAAAA	Y	29							
4868	LOC Os02g47410.1	GSACLEGKTIYSYIQSRYYRSPEVL	Y	291							
4871	LOC Os03g10460.1	FPKSAAPKGEIHYFRDSDDEQPDSD	Y	363							
4874	LOC Os03g19530.1	AMHAGYDSDEEVYAAAKAVDAGMME	Y	136							
4877	LOC Os03g24890.1	PARRDYRDHRDDYSPGESLSPHGQD	Y	196							
4880	LOC Os03g50330.1	NHKSAEDPDGSFYTGDDPDRSFYDR	Y	51							
4883	LOC Os04g02870.1	DYGRQRSPVYGSYDRSPVNDRYRSR	Y	216							
4886	LOC Os04g02870.1	DDSPYRRSVSPVYRSRPSPDYGRQR	Y	197							
4889	LOC Os04g13960.1	DGEVFGRDDTMDYRTIIVQRVLSTQ	Y	162							
4892	LOC Os04g38570.1	LRSGSLRNLSYSYSTGADGRIEMVS	Y	653							
4895	LOC Os05g11770.1	WNIEPSLFFPKQYFSDTEEFEDVIY	Y	278							
4898	LOC Os05g40060.1	ARTSEDGFLWRKYGQKEIKNSKHPR	Y	135							
4901	LOC Os05g43970.1	EVYDRRQESDEEYDRSQESDEEESD	Y	54							
4904	LOC Os05g51754.1	NSSDSKAKKRVDYSDDDDDFIVKLK	Y	148							
4907	LOC Os06g06090.1	ARTTSETDFMTEYVVTRWYRAPELL	Y	227							
4910	LOC Os06g08840.1	RRRSVSPARARSYSRSPQYNRGRDE	Y	160							
4913	LOC Os06g11610.1	RLYNTEGAPLRRYDVVDESGTDSGD	Y	71							
4916	LOC Os06g17840.1	SKSRPHSTKGNKYSDELLLQSVDPD	Y	1241							
4919	LOC Os06g34440.1	PRRSSRQKQEVKYNEDSDGDDTDCH	Y	363							
4922	LOC Os06g45560.1	IDVAIVGSLGGSYLRSYNYKPATRP	Y	799							
4925	LOC Os06g50890.1	GRARRERSRSLPYSPYRMPERGYGR	Y	252							
4928	LOC Os06g51250.1	AFARFDSMKSTDYNSRGYSFDEDDP	Y	1151							
4931	LOC Os07g05610.1	NLQHSLSRNARVYSGIDLSKDEVTQ	Y	814							
4934	LOC Os07g25680.1	LQPSDSAKSLETYTASKAMSAAQDS	Y	513							
4937	LOC Os07g38730.1	VDVTEFQTNLVPYPRIHFMLSSYAP	Y	262							
4940	LOC Os08g02690.1	RNDPNYDSDEEPYELVEAPVSTPVE	Y	123							
4943	LOC Os08g16930.1	EPSSEEMEELHGYQEPVRVGRVMRY	Y	171							
4946	LOC Os09g19952.1	YEETVMILNLMPYRASYPILKLVYS	Y	30							
4949	LOC Os09g33870.1	RRRSREPGELSPYKEDDDNSKRGRS	Y	309							
4952	LOC Os10g14230.1	TDVQNNPLVTKEYDGESDDELDADV	Y	572							
4955	LOC Os10g39440.1	DWDAESQREGEDYGSDHGGDDIEDS	Y	355							
4958	LOC Os11g20040.1	VIGDKTTDGVVSYVAGDMLSFIPPA	Y	252							
4961	LOC Os12g05420.1	EVEVRSSTNEDYYEDPVPNKQNPLA	Y	308							
4964	LOC Os12g38970.1	QPDDVVADEDMRYESDENRELKPKE	Y	261							
4828	LOC Os01g01270.1	IRAVNAILRETPYDPVLNDDLARWT	Y	314							
4831	LOC Os01g09620.1	SGGWSYPSSSAVYGSPKAATGLYSL	Y	292							
4834	LOC Os01g16110.1	SESPPSNSIGYFYGSTPENHSYRSS	Y	705							
4837	LOC Os01g43170.1	LNVKFQSHRSRDYTNPYLPVAPSAI	Y	210							
4840	LOC Os01g49640.1	LCRYAKNPDLRKYINSQNSRKVAAA	Y	83							
4843	LOC Os01g53680.1	FSATCVRISRDSYPNLRALRNASAM	Y	57							
4846	LOC Os02g04490.1	SPCGARIKRNAPYYTVGTGDTRHFF	Y	947							
4849	LOC Os02g07070.1	SYERDRDYDRYSYDSDYEKSRRDGS	Y	139							
4852	LOC Os02g14130.1	AKMLVKGEANISYICSRYYRAPELI	Y	234							
4855	LOC Os02g25580.1	GTWARPRGSDDGYSSDSVLKHSSSD	Y	586							
4858	LOC Os02g32350.1	EKAKKERLQIWQYGDVESDEEEQAP	Y	965							
4861	LOC Os02g37880.1	SLVSAMESRGTGYYEEKKALLILRD	Y	125							
4864	LOC Os02g42134.1	SKFTTAFRELGTYKELLRSQVDHML	Y	92							
4867	LOC Os02g47220.1	AREVTSVPPYTEYVSTRWYRAPEVL	Y	158							
4870	LOC Os02g56270.1	PSNQQIASPPPNYEDVTRDTQDNNH	Y	306							
4873	LOC Os03g19530.1	GLALAADAMHAGYDSDEEVYAAAKA	Y	129							
4876	LOC Os03g23970.1	LLEVEELRGGSAYGADSLCIGVARL	Y	214							
4879	LOC Os03g40110.1	KDDFDEEVPLDDYSDEEGHMILSKN	Y	179							
4882	LOC Os03g57160.1	QSVGQADSGSKIYGSTKADSVESGE	Y	766							
4885	LOC Os04g02870.1	ARGGYGRRDDSPYRRSVSPVYRSRP	Y	189							
4888	LOC Os04g08450.1	ELCSSRVLSPPRYESGDEELSVLPR	Y	18							
4891	LOC Os04g35800.1	RSRSRGRSASPRYPDGKRRRHNDLN	Y	93							
4894	LOC Os04g51370.1	GSACLEGKTVYSYIQSRYYRSPEVI	Y	291							
4897	LOC Os05g27820.1	ALLRSSSFKEDSYVASALPASDLRA	Y	52							
4900	LOC Os05g40960.1	LTLYHLKSHLQKYRLSKNLQGQANV	Y	96							
4903	LOC Os05g51630.1	RQSRRNTPAQSKYEGSDTLSLPETV	Y	751							
4906	LOC Os06g05190.1	KHQSKPRSDDAQYDSDDTIEMTEEE	Y	320							
4909	LOC Os06g08840.1	PARARSYSRSPQYNRGRDESPAYDN	Y	166							
4912	LOC Os06g11610.1	VVDESGTDSGDEYDATDDGRRLTVP	Y	85							
4915	LOC Os06g13390.1	GRKKVYLNEENIYITKTTPQSVVKL	Y	153							
4918	LOC Os06g33520.1	RGSDLDDSEDDEYSRGSSPRGRRGR	Y	293							
4921	LOC Os06g43610.1	KNGGGKEKPGSDYSEDDGETAEPNK	Y	356							
4924	LOC Os06g49740.1	DAEQEDAGSDDGYPQMTEKQKKLFE	Y	122							
4927	LOC Os06g50890.1	PERRRYSGSPSPYRGNPKSRSRSRS	Y	17							

4930	LOC Os07g05190.1	SRFSWSPDSGEAYTQEGLARLDVRS	Y	747								
4933	LOC Os07g19130.1	SKVCEEDIQNMKYLKMIIKENFRLH	Y	81								
4936	LOC Os07g31840.1	RAQKLAAAREAPYAKSRTQFTRDMQ	Y	601								
4939	LOC Os08g01100.1	PYLDKAAELKAEYHNGERSDENNVG	Y	153								
4942	LOC Os08g09910.1	NSRHGNLVDDYNYGSDIDSDDDLHE	Y	388								
4945	LOC Os08g34230.1	NLAHKLKGMENWYEELMRNSKSYNE	Y	25								
4948	LOC Os09g31230.1	PDLRASMVQRRGYTSDDDLDDLGNP	Y	722								
4951	LOC Os09g36090.1	RPAFSSHGESAGYTSKSDSPNIGTH	Y	1173								
4954	LOC Os10g37740.1	AKKLVPGEPNISYICSRYYRAPELI	Y	300								
4957	LOC Os11g18470.1	EREKRETQGLVDYYNDSESKIKALH	Y	232								
4960	LOC Os12g03899.1	KKGSTGTGVDDGYCIEGCPGCVVDR	Y	29								
4963	LOC Os12g07530.1	LGREYYDDDVDSYDLSPQRMAEPYD	Y	615								
4966	LOC Os12g42140.1	ALYGVFAEGDSDYDSDDGRRRSRRK	Y	56								

Table S2. The 25-mer sequences (-12 to +12) around Ser, Thr and Tyr residues not annotated as phosphorylation sites were extracted from protein sequences and compiled into the negative dataset.

No.	Protein Name	Sequence	Amino acid	Position			No.	Protein Name	Sequence	Amino Acid	Position
4967	LOC_Os01g01150	RHSRDRCSRHSSSRSHRSSRHSREK	S	831			4968	LOC_Os01g01880	LAREIERLLLQGSEARRKNEAIKQE	S	42
4970	LOC_Os01g02020	SQIDYYEINEAFSVVALANQKLLGI	S	334			4971	LOC_Os01g02700	CIQMRSCDRPMMSEVIEMLEGGVDC	S	621
4973	LOC_Os01g02720	SKCRALGRAFLRSGGSTIAVGVVTR	S	636			4974	LOC_Os01g03500	DADDSDELRSICSESDEDDMALWSG	S	139
4976	LOC_Os01g03520	ASLDDGTASDGASWETVDDNQTDLP	S	667			4977	LOC_Os01g03650	DAGQETKCLLLSSKALVNVKPNLQI	S	394
4979	LOC_Os01g04100	ERLLLHLREVDGSDVVADAEVAAAV	S	91			4980	LOC_Os01g04160	LPVVESSPSVVGSSEKEEFSRLLKA	S	516
4982	LOC_Os01g04190	IGISSALVPLYISEISPTEIRGALG	S	236			4983	LOC_Os01g04720	EFPQDLTSISAISAKRNRYQRAKSV	S	606
4985	LOC_Os01g05420	MKKPSSSSSGMESRDDDLDADFSQR	S	440			4986	LOC_Os01g05420	QLKAPKKKNLQASKLSVEGRGMIKY	S	520
4988	LOC_Os01g06270	FSEIEKLRERFESVERPTLDIEVRR	S	444			4989	LOC_Os01g06270	YHLSTFKEILSPSIERICTYVDNLA	S	350
4991	LOC_Os01g07350	ADLENIGSYSWGSATLAYLYRAMCD	S	156			4992	LOC_Os01g07350	PFPLIADLENIGSYSWGSATLAYLY	S	151
4994	LOC_Os01g07880	EQEKQQVKTSTTSSLPSSSERSSSS	S	19			4995	LOC_Os01g07890	AALQRSSQTFRRSGSSGLVWDERLM	S	14
4997	LOC_Os01g08420	TVDPLKSLANNISFATEVEVQLGKR	S	960			4998	LOC_Os01g08430	DPIIFCLDERSKSVQNHLDVAAAPS	S	753
5000	LOC_Os01g08560	QLNVAFVDVGHASMQVCIAGYKKGQ	S	205			5001	LOC_Os01g08560	SVPMETDAKVEPSKKKVKKTNVPVA	S	570
5003	LOC_Os01g09510	IACGREKFASVPSGGGGVAVAAAAP	S	67			5004	LOC_Os01g09570	IYEEVRRRGLKCSVVGVPKTIDNDI	S	246
5006	LOC_Os01g09850	KAAQMGATLSRPSSHAHMAAAAAAS	S	358			5007	LOC_Os01g10610	PRASYNASPTSSSFPSGASSPFLPH	S	136
5009	LOC_Os01g10690	VNKHGADLKVIYSVSGWLKKNPTNY	S	64			5010	LOC_Os01g11140	KQRYGIDYEDTFSPVVKATTIRLVL	S	883
5012	LOC_Os01g11580	SSSAYADTDTDSSSADGSDAAQSPR	S	342			5013	LOC_Os01g11960	EGCSLEKSVNDQSIPDGLLEGSGSN	S	280
5015	LOC_Os01g12390	GDEEVSRVVSGKSGEKKGRESPESK	S	298			5016	LOC_Os01g12480	ARRPRRPGAPSSSSSTGEWHNFMGS	S	38
5018	LOC_Os01g12660	SSMTDMKLRSSESTSSFPKLKRNAS	S	204			5019	LOC_Os01g12750	LRLGRVPAVVASSAAAAEEVMRTRD	S	97
5021	LOC_Os01g12770	GGDGRRRRRLPPSPRGLPLLGHLHL	S	55			5022	LOC_Os01g12780	ARLEEERRLKEESDQRQQLAAAADE	S	82
5024	LOC_Os01g12880	TTPSSSSSMGQPSKPEDKLFGDLLS	S	690			5025	LOC_Os01g13080	ASAADSAAGVQSSFAMVSPSSAVFQ	S	54
5027	LOC_Os01g13270	SSIARSNSLDSFSYGQAKRHTGGDS	S	107			5028	LOC_Os01g13470	VQTKENDGGVACSVDGNHGMEKDHT	S	118
5030	LOC_Os01g13530	FSDSLKDLRNLRSQLYSAAEYFEVF	S	48			5031	LOC_Os01g13680	QVIADRLYADMVSAGAVQGNGNVTT	S	37
5033	LOC_Os01g14010	VPPQLYVDLFAPSSGAPPPQFDAAQ	S	313			5034	LOC_Os01g14050	KLEILEGSDMKPSDMNGLSDPYVKG	S	300
5036	LOC_Os01g14440	APQMGAAGAAGKSHDQQAQDANMRK	S	274			5037	LOC_Os01g15039	TVSEAVARAPSHSRRRYVIYVKRGV	S	329
5039	LOC_Os01g15460	IAYGGPYVPYASSTGQSSNNQQEHG	S	291			5040	LOC_Os01g15630	RWSVDSELLGSVSSKMTRSNASNNP	S	262
5042	LOC_Os01g16100	TLNDKRERLVKASRDLTMNSKKAIF	S	110			5043	LOC_Os01g16110	KSLMDEHGWVPISKVADFNRLKKMT	S	364
5045	LOC_Os01g16390	TTRNRQVAESCCSLPVDRVYSMDVL	S	348			5046	LOC_Os01g16400	LGEFLSDKYVLLSGVRHEIQELKDD	S	32
5048	LOC_Os01g16790	VQASGPEGSAPSSLRLIAWIAEIQA	S	1477			5049	LOC_Os01g16790	VTGCMAALSRFISRLGEKALPLFKL	S	1003
5051	LOC_Os01g16870	TVVLEDFNTGKSSANGGSPGNDSPG	S	145			5052	LOC_Os01g18890	VLFTSDATLVSQSDTAALVDLYARN	S	189
5054	LOC_Os01g19480	APRPTLHHPILMSSAIPPQQQQQRP	S	543			5055	LOC_Os01g19480	NTNGTLRAAGGVSPRTLKTDQFLRT	S	451
5057	LOC_Os01g20940	ESSIPTITQLVQSDKRTHTANATIN	S	369			5058	LOC_Os01g21590	QQSYSPYSAGASSRVIKAPGHDDGM	S	66
5060	LOC_Os01g21860	EDEMEFLDKLASSRKEYEQQVANEE	S	93			5061	LOC_Os01g21960	SSPSSPSLKDHLSSPTGPLHLKVWE	S	18
5063	LOC_Os01g25330	LSDVSGAAIIVGSCGYSTSDCPIVE	S	738			5064	LOC_Os01g25370	VRRWTTKRKLGYSLLECDKIFVPIH	S	372
5066	LOC_Os01g27730	IANLASAASAQGSEFAEKDAAKENA	S	263			5067	LOC_Os01g27730	SSFLLLPSSALPSLLPSAAAAAASN	S	132
5069	LOC_Os01g29590	SDSGVAASAAPGSAARSGRRDRRRK	S	239			5070	LOC_Os01g31510	CQKRKLNSDEITSTSNSKTKVQKTG	S	223
5072	LOC_Os01g32130	QDHGDKSGSSSSSSSSSGSDSDRE	S	21			5073	LOC_Os01g32660	KITDFGVSAVLASSMGQRDTFVGTY	S	220
5075	LOC_Os01g32870	MYFLHFQVYRMDSTVNALAMAKDPE	S	228			5076	LOC_Os01g32964	KLSNAGGRLIIASDGIWDALSSEAA	S	153
5078	LOC_Os01g33000	DGEYHDSSSDASSDYELGRVKHLTQ	S	185			5079	LOC_Os01g33090	LACVQANPERRPSMRHVAETLDHLN	S	690
5081	LOC_Os01g33370	RVVYLTERPQLRSCGVSNTKLSSSQ	S	109			5082	LOC_Os01g33440	DQSDKAQLDVLKSLLQDVRLDVLPD	S	343
5084	LOC_Os01g33800	PDPDSASSPSPSSASSPSRSPDRRS	S	43			5085	LOC_Os01g34030	RLEEEVGETIKLSSASLARAAVELV	S	767
5087	LOC_Os01g34410	PPGGFLSYFQDPSILQNHRPFVPPN	S	102			5088	LOC_Os01g35050	ILMFMSKVEGLTSVSSLERRSAFKY	S	452
5090	LOC_Os01g35730	ISNAQNAADKIASDVVDQFRNNNLQ	S	828			5091	LOC_Os01g36250	TEWVEFEPREKWSLLYDTDGSRYGV	S	644
5093	LOC_Os01g36860	EAVKFGRSSRISSTCLYGGAPKGPQ	S	253			5094	LOC_Os01g36860	RHSRSRTRSRSRSRSRSYTRNRRAS	S	628
5096	LOC_Os01g36940	AVGGEQKKAPGPSPAAAAAAAAAGA	S	80			5097	LOC_Os01g36940	DYTSLCRLSMTNSAMRRAANDDGAW	S	123
5099	LOC_Os01g37790	GHSSAQYSGGKMSNAFEPSRGKFGF	S	338			5100	LOC_Os01g37825	PEEIVSQSPEPGSILDKVEHPGIGA	S	531
5102	LOC_Os01g38950	QMLDYSDPRHSSSGRHAANKNYEST	S	443			5103	LOC_Os01g38950	RELQSSEAEPSRSRTPDASPGTFQR	S	410
5105	LOC_Os01g39700	APLILGETPIIGSSKKNIVGKKHQD	S	47			5106	LOC_Os01g39700	MGFKESPLLLLPSVGNPHWPSDHHI	S	166
5108	LOC_Os01g40340	ERGITDRLAAVPSSSPNSEEISPDT	S	500			5109	LOC_Os01g40340	NFLHHHVENIIVSCLEGKRTELVEH	S	285
5111	LOC_Os01g40480	LARWLDSVQRWRSPPTTVLVHVLYL	S	906			5112	LOC_Os01g40480	PYAAYTRSKVYQSPKLWYLRASVIE	S	493
5114	LOC_Os01g40590	STTSSNTGSISPSSIVGEELKLAFQ	S	112			5115	LOC_Os01g40810	AHATEGASSSGVSPQHGRGDQPSVP	S	202
5117	LOC_Os01g41090	EITDASVIRTFKSSVRDRYTTQELA	S	562			5118	LOC_Os01g41190	GGNGIPSNNQSTSSLHQNPRAPTHQ	S	453
5120	LOC_Os01g41280	DAVANAVTRVLASHPGPLRFVGVIN	S	121			5121	LOC_Os01g41610	LRSKAAQASELVSKHGCAYYKEVME	S	20
5123	LOC_Os01g41620	AGLLTFARLVEPSRARSERIHIDAA	S	925			5124	LOC_Os01g41630	SRSFFTEIIASISDIKFSKDGRHIL	S	340
5126	LOC_Os01g41880	GRGDRGGFRDGYSSRGPVAAPRIED	S	353			5127	LOC_Os01g42260	GHDGEFVNELSCSGNKFQSCVFHPS	S	805
5129	LOC_Os01g42294	AEDFAGNLQLCGSPLPPCKSFFPSP	S	228			5130	LOC_Os01g42350	WLSADGNGAFSRSSSSSSRRMRGEE	S	39
5132	LOC_Os01g42380	LMGHSWNKIVNSSASNETLGVQVLK	S	740			5133	LOC_Os01g42380	TQRGMVLPFTPLSLSFDNVRYSVDM	S	859
5135	LOC_Os01g42820	KKGKQSQQQQQQSGPGGAQPPQMLQ	S	268			5136	LOC_Os01g42820	PSSMGGGGAGLGSLGNQMPSGMGGA	S	343
5138	LOC_Os01g42850	ENPALLLRFLVISFADLKNWKVYYN	S	140			5139	LOC_Os01g42960	GHRRCGSGPLIFSGGSSGGSGGGGG	S	108
5141	LOC_Os01g43050	LPDFNVPGDTPASNTTEQKNPMSDH	S	300			5142	LOC_Os01g43170	FPGYGSPQFPPGSAQAQMHQQHPVQ	S	433
5144	LOC_Os01g43330	EDGNAFAESGGSSFNERTNSGFSRG	S	610			5145	LOC_Os01g43330	RKECDEDSLVDESMCKTHEQKSKDD	S	461
5147	LOC_Os01g43420	APTTVDEASMERSKSFVKALQELKN	S	41			5148	LOC_Os01g43750	CKLFYFAGMETTSVLLTWTILLLSM	S	344
5150	LOC_Os01g43920	DVNAAHDLKNRYSLACSFSLHTRTL	S	126			5151	LOC_Os01g44310	EKGSMGLTFPSSSSLKSASSDSVAD	S	241
5153	LOC_Os01g45550	KRKKWGQRERKKSDPVHLKQRMLIC	S	613			5154	LOC_Os01g45550	QKLLVLAGLAAWSRLPSRTGAPRLD	S	90
5156	LOC_Os01g46810	EQANEELREKISSLERQLEEARSKL	S	877			5157	LOC_Os01g47490	DSADWALGKQGGSHKPKGPLEALRP	S	83
5159	LOC_Os01g48210	LPTSDIDVVIFDSRVKTPQVGLYAL	S	259			5160	LOC_Os01g48210	PKQRSRKKAKADSSSGNNAENGFKR	S	575
5162	LOC_Os01g48330	ERDKKLFMLKQLSGALVKGLNSSPA	S	127			5163	LOC_Os01g48330	SPSYVGEDQNNASDCPNNDDTSGGV	S	456
5165	LOC_Os01g48720	AIFDNIVHRQRFSKFLSIIRADIPE	S	95			5166	LOC_Os01g49020	GSLTLNKAAVIASSQCFCSKPSITL	S	58
5168	LOC_Os01g49190	REGNDLYREMIESGVIKLGDKQSES	S	279			5169	LOC_Os01g49350	GTKCKTCLNLVISRIKLLRNRRELQ	S	26
5171	LOC_Os01g49529	VVPVNGSILNSSSQSALPSGEYGQV	S	231			5172	LOC_Os01g49890	PLSRPINGVGCASTGDTSAALSAYC	S	233
5174	LOC_Os01g50320	LDRVPPRPRRSYSAALLSAPSLDGV	S	62			5175	LOC_Os01g50450	GEEVLIVEAAAVSAVQEPEVKRDEV	S	175
5177	LOC_Os01g50470	LQISDKKTKTVVSMATEEYTRCSAV	S	830			5178	LOC_Os01g50470	WRENQERVFKKYSHPTVPQTTSTKT	S	1022
5180	LOC_Os01g51010	PQENRRGSDAPMSPRSQHGPNSFSH	S	286			5181	LOC_Os01g51154	IEALCEIGDPNGSDVDAICHYIEQR	S	135

5183	LOC_Os01g51210	GDAALQLGQELVSRGIELVYGGGSV	S	48			5184	LOC_Os01g51210	PKALMPTEISGASVGEVKIVSDMHE	S	94
5186	LOC_Os01g51300	HDSTVEDVQFCPSSAQEFCSVGDDS	S	260			5187	LOC_Os01g51300	KATYKNRQRLYLSEQTDGTVPNTLV	S	60
5189	LOC_Os01g51620	VRLESYVASNEGSRPKKKHKLSKYS	S	406			5190	LOC_Os01g51640	DAELEDLGCLINSLSVVEPEQFDSP	S	475
5192	LOC_Os01g51710	PRSTTVEVEVGASGYIVKQDNVHSS	S	403			5193	LOC_Os01g51890	ASGSQQEPAATASHHAAGAGASSRP	S	37
5195	LOC_Os01g52380	LSSFNPLKQFCFSTTTRKMVSEVSE	S	115			5196	LOC_Os01g52380	TNQAMLLQLRMLSNMMYRGYYFLDN	S	70
5198	LOC_Os01g52950	MAEQAKAHNLSPSVSGDDGELNPRR	S	13			5199	LOC_Os01g53140	GVYLRNSQTKANSYWRRHKQIRRSF	S	56
5201	LOC_Os01g53520	SSDSDDDKKGSSSSSAAAAMKAKIY	S	38			5202	LOC_Os01g53560	NLKKEVVHSMEDSKGTGNNIEGVKS	S	221
5204	LOC_Os01g53700	IDCGSIYETPASSGASHLLERMAFK	S	109			5205	LOC_Os01g53980	NHAVSDGAEKEKSRWSFAKHRRSGV	S	37
5207	LOC_Os01g54290	EKKMPYYLFASSSGQAVTVLLKELE	S	1090			5208	LOC_Os01g54290	LVFSDVWGPAINSVGNKVYYVSFID	S	513
5210	LOC_Os01g54660	EKIRAIERMRPPSKLRDVQCVTGCM	S	1052			5211	LOC_Os01g54660	RPPLLSSDPEVISTEDKCAPRGCLD	S	1282
5213	LOC_Os01g55120	ALGPEVDASVFRSEEAIAAARGFLR	S	100			5214	LOC_Os01g55120	TYLSNGLQRLKDSTTIPDLNNGKPQ	S	575
5216	LOC_Os01g55280	PRSRGSELPQRASPRAPLHLKTTAC	S	14			5217	LOC_Os01g55350	EWSEEKRQEWLLSELNGKRPLFGPD	S	445
5219	LOC_Os01g55420	PSRRQPLLRPGKSTAFKREERRKRK	S	28			5220	LOC_Os01g55490	LPSRRSSSPFAISSPSPLRPAAAMP	S	101
5222	LOC_Os01g55560	QEEASGAAPFGRSSSLIGAAGFDGA	S	29			5223	LOC_Os01g55650	SVDCEAETITSKSKGSSLFDIGMEI	S	869
5225	LOC_Os01g55799	DLIQHGALSAVKSIANFMFLSEYDK	S	173			5226	LOC_Os01g55799	KIIKEKFKNFNLSFEDAYRTQTGWS	S	525
5228	LOC_Os01g56100	ARSIAATLSSSHSDEDGDNLEASSG	S	17			5229	LOC_Os01g56100	VPSLSYLRTELCSTNMSEACFWKIY	S	218
5231	LOC_Os01g56190	YNNVSTLHSSWVSKANARQRQGRAG	S	685			5232	LOC_Os01g56300	SEEIESILRMQHSASHSSDPYVVDY	S	404
5234	LOC_Os01g56790	KLAGAQSMCERCSDSLVENNDDRTD	S	104			5235	LOC_Os01g56910	KSSLQSTSDDSTSRSPWKSYSNGER	S	985
5237	LOC_Os01g57450	PSSPSAGGGGDVSASSPTLPPVRLA	S	17			5238	LOC_Os01g57450	YGADWGSIPVSVSDDADGEVAPAAS	S	114
5240	LOC_Os01g57790	PGQANLGKSITKSANGKVTTRIGLG	S	503			5241	LOC_Os01g58400	IQATIEETIRRQSEAVGKKRSTAKA	S	223
5243	LOC_Os01g58550	VNNKPLSPSYKYSRKIPVKTSKSES	S	333			5244	LOC_Os01g58870	YTSSVLPIGALYSLSLWFSNSAYIY	S	110
5246	LOC_Os01g59790	QRHWYIQSTCATSGEGLYEGLDWLS	S	162			5247	LOC_Os01g59850	DQQLPPTTSVQQSQPAEPSISSTPQ	S	493
5249	LOC_Os01g60040	QVEKNAAAGRTPSSLPLSTPPPIAP	S	1848			5250	LOC_Os01g60330	SLHSLYLQHNNLSGIIPTSLTSTLT	S	131
5252	LOC_Os01g61180	DVVIDALPPGSVSDVHQIARRMVDA	S	230			5253	LOC_Os01g61210	VFVDTIVRCNLQSLARTVERLAPEA	S	186
5255	LOC_Os01g61590	KRRALRVIADHLSAEEVEDIKEMFK	S	376			5256	LOC_Os01g61590	NIDFKREPWPNVSENAKDLVRRMLE	S	305
5258	LOC_Os01g61780	QAPAGEPVTDPISGEEVEKTVFVVF	S	232			5259	LOC_Os01g61814	GVRFKVVKVSGVSLLALFKEKKEKP	S	128
5261	LOC_Os01g61830	DLDIDELNELEASLSRTSIQIREPG	S	349			5262	LOC_Os01g61830	YRHALPPGYVLKSQMKALLEEESEK	S	205
5264	LOC_Os01g61860	SADKENAANELQSANNEEIKPSEDS	S	143			5265	LOC_Os01g61930	IGKFLQSRQAPLSQYNQRPSKKKRI	S	610
5267	LOC_Os01g62650	AGERERELNMFRSGSAPPTIEGSLN	S	59			5268	LOC_Os01g62810	AADQGVSPRSQVSRTSSGAASGPSE	S	197
5270	LOC_Os01g62950	DTAGQERYRAITSAYYRGAVGALLV	S	79			5271	LOC_Os01g63940	LEAKDVLLHAERSWNITLPGFSGDE	S	235
5273	LOC_Os01g63950	SAYDAPPPHHEDSDGDDTDEAAAED	S	51			5274	LOC_Os01g63950	TESHRQGRGRRASRFRGGGGGGDED	S	20
5276	LOC_Os01g64250	APLGRPVLRLSASDPELCGTADELL	S	109			5277	LOC_Os01g64280	KAIKLLGACEGDSLEKVFSEVGSKS	S	238
5279	LOC_Os01g64600	QERREGKSPAVPSGNRRSEAKQKAP	S	519			5280	LOC_Os01g64720	CANTDVVAGWCESGSVFVADVRQDC	S	344
5282	LOC_Os01g64790	NALVYPATAASWSESSYHHHPPPPH	S	337			5283	LOC_Os01g65230	GNDGASSSSEVRSHISRLASIDSFD	S	379
5285	LOC_Os01g65330	PWDGVPNLARLPSQKSGMGVAKASA	S	97			5286	LOC_Os01g65480	VKKWHPDKHPPSSKPEAEARFKAIT	S	43
5288	LOC_Os01g65900	LSCKEPPTLYQLSAMQILFEICPCF	S	258			5289	LOC_Os01g66130	LVAALHPKAQCASDDSHHYNLIHEN	S	345
5291	LOC_Os01g66520	AEVPDCTLASKDSNEPETFAKENET	S	383			5292	LOC_Os01g67160	GSTHYSTGVDIWSVGCIFAEMVRRQ	S	198
5294	LOC_Os01g67250	IHNMEIHVRLDMSQTMADGSNDVGT	S	707			5295	LOC_Os01g67550	SLGSVIPRNLQKSVLEGLFSIGRAQ	S	485
5297	LOC_Os01g67640	GIERTRSQAGILSFLKHSTNVCAHN	S	248			5298	LOC_Os01g67640	RRLLHTGIERTRSQAGILSFLKHST	S	242
5300	LOC_Os01g67750	NTLNEGLVLHHASVGCPKPQWSKYL	S	340			5301	LOC_Os01g67750	RALNKALYWHHKSRDCPRPSPVDGA	S	699
5303	LOC_Os01g68120	SEKVQPSFSSVCSSPGGSSQKATPQ	S	1531			5304	LOC_Os01g68370	ELADIDQLLDLASLSVPWEAEQPLF	S	118
5306	LOC_Os01g68620	FWVFISPFIFKKSVMITVARGSDEG	S	401			5307	LOC_Os01g68620	TARFGEVLPATGSDGDKRKAVVPAP	S	90
5309	LOC_Os01g68860	PLRPGEELCKFYSRYGICKFGANCK	S	388			5310	LOC_Os01g68920	WTWNPSGSGVAASAAPGSAARSGRR	S	404
5312	LOC_Os01g69090	MYRGRSAPLMCKSTSSLAAVMAQML	S	350			5313	LOC_Os01g69100	QPVSAAVKPVSESSSTCSTEPTPIR	S	179
5315	LOC_Os01g69260	QVQLHVHGPMLNSELHYSQEKNKRK	S	29			5316	LOC_Os01g69900	VGRSAFYPASGLSNTFGFKIQDKQG	S	415
5318	LOC_Os01g69990	DRLSFDDPLAERSLFAKAGQAGQEG	S	1284			5319	LOC_Os01g69990	MDESGHFIRSGTSPNQGHASRLGHF	S	1045
5321	LOC_Os01g70100	LYVTMFLIIAPASIFCLFVAKELMN	S	58			5322	LOC_Os01g70320	RPTTTVVPPIKTSTTLRTPSPIPPV	S	171
5324	LOC_Os01g71230	PDLSNVISKAEPSAAAQDDEEVDES	S	149			5325	LOC_Os01g71590	MDPSRPFLVIKKSDLDKAGEWVKIK	S	53
5327	LOC_Os01g71970	AAANQSILEAMESEKIVHVIDLGGA	S	156			5328	LOC_Os01g71990	APPQPVPAPAAASPEVFRLGFIGPG	S	15
5330	LOC_Os01g72080	MSKNGSGVKRAISRSEASSFASASS	S	46			5331	LOC_Os01g72320	ALTTSGHVYTMGSAVYGQLGNPQAD	S	553
5333	LOC_Os01g72710	STSVAYGVYMAVSSNLRYQILAGVI	S	326			5334	LOC_Os01g72860	LRFVESNPLNSYSSNEELVMMPKRR	S	256
5336	LOC_Os01g72890	SSRSRSRSRSFSSSSSPSRSRSPPA	S	44			5337	LOC_Os01g72940	ATCAADSRLMTFSSVDESTRLWIKG	S	425
5339	LOC_Os01g72980	RRHRLLSTAVASSSSSSKVSTSLEE	S	54			5340	LOC_Os01g73040	TIITENNSVPVVSPSDPVIAAAKKM	S	235
5342	LOC_Os01g73160	WYLTNDGIEHLRSYLNLPSEVVPNT	S	78			5343	LOC_Os01g73230	QVDELEKTILVASRDPAYYGLDEVE	S	68
5345	LOC_Os01g73470	LEQQAKVSPSSSSEKISTPSSGPKR	S	55			5346	LOC_Os01g73470	SPSSSSEKISTPSSGPKRRGRGSFL	S	62
5348	LOC_Os01g73560	LDQLHQLNTLVLSKNPIFTIGDALM	S	136			5349	LOC_Os01g73560	NLRIFNSKPMEASSKSKNSREENLP	S	262
5351	LOC_Os01g73644	ILSRSSCRTLARSPKYLFLPPCNAN	S	15			5352	LOC_Os01g73880	FDYGDEICGAVVSVRGKQERIAIWT	S	168
5354	LOC_Os01g74030	SEQGHVKVPQEYSGASMNPSVMRSQ	S	341			5355	LOC_Os01g74030	VERQVLIGEMLGSSNESEHLEVMMK	S	816
5357	LOC_Os01g74200	TTTTSHHHRSPQSRMLLGGRGFNAS	S	375			5358	LOC_Os01g74680	GKSPAVPSGNRRSEAKQKAPAEDID	S	524
5360	LOC_Os02g01020	RSCRRGKVRNLASFLSSDAVATALA	S	239			5361	LOC_Os02g01030	DPNLAFFRDYLESLGAHLPAAAFTK	S	37
5363	LOC_Os02g01380	AYDGEGSLQLSSSRWPKHEVEALIR	S	183			5364	LOC_Os02g01510	ELTEEEARRLRRSAKTLWENCQLLD	S	379
5366	LOC_Os02g02050	SPHVSCISEPLQSRVTKSQTMDEVN	S	1063			5367	LOC_Os02g02050	TYRAAGAQPHAPSPHLQQLRMPSPY	S	2513
5369	LOC_Os02g02570	LPLLVPIIKAHFSSTLPPGIDTVWF	S	66			5370	LOC_Os02g02740	ASASQPPAPPRPSNPRHRKTPAKAS	S	36
5372	LOC_Os02g03030	DSMMTLLSAATPSRSLVICPDKRSN	S	284			5373	LOC_Os02g03040	ALRDDDEKRNGYSPERRGRDRSPDR	S	176
5375	LOC_Os02g03740	NILATAFHPELTSDSRWHRFFLDMD	S	208			5376	LOC_Os02g04050	RLDLLKEIGGETSCEWTFIHALLTA	S	167
5378	LOC_Os02g04230	LDLITDLLGTPSSETLSRIRNENAR	S	240			5379	LOC_Os02g04230	WYRAPELCGSFFSKYTPAIDIWSIG	S	194
5381	LOC_Os02g04240	RERERERERGRSPSGELSLQLEHVSL	S	39			5382	LOC_Os02g04450	VIHGLPSSMDASSSLLAAHNSTAGR	S	178
5384	LOC_Os02g04560	FSSELDGDKSSASVQDVEALADSST	S	372			5385	LOC_Os02g04660	VWYEWAVTAPSPSPIHNSNGRSYWV	S	635
5387	LOC_Os02g05410	ILMGCAVLPHLKSLVEIIEHGLSDE	S	196			5388	LOC_Os02g05410	TTVAIAIVAETCSPFTVLPALMNEY	S	636
5390	LOC_Os02g05510	AAECHHCGISAASTPMMRRGPDGPR	S	245			5391	LOC_Os02g05620	FKDYYLTVFMRDSWGVVSPLLNREG	S	523
5393	LOC_Os02g05686	LRALPEAIQCFTSLWRLSILGCGEL	S	187			5394	LOC_Os02g05686	SMDNLEEWWTTRSSKQDNELSLIPD	S	81
5396	LOC_Os02g05840	WHRVAATGSFSSSPTGIILAPLKTL	S	389			5397	LOC_Os02g05880	FLGLPLETGKCESCGASENGKCEGH	S	60
5399	LOC_Os02g05900	DSSIIPSMLRSPSVDSNRNDEMTTE	S	156			5400	LOC_Os02g06370	ASYTVFKGKAALSMQPILPSFSKLE	S	60
5402	LOC_Os02g06920	KNEVETQIRRSLSAPGNHDSKDLRR	S	182			5403	LOC_Os02g06920	LPVTPVAASGPSSHEGHADNLPSTV	S	157
5405	LOC_Os02g07370	LLRHPPVVPDPESPVQRWLDDVANL	S	100			5406	LOC_Os02g07370	MAEQAKAHNLSPSVSGDDGEPNPRR	S	13

5408	LOC_Os02g07940	ALERVEGSATSTSTGDGEVRRDGER	S	55			5409	LOC_Os02g07960	EKRVSTPPRTGPSTSATSFSVASLQ	S	432
5411	LOC_Os02g08300	SNAYGQAASNLLSGSNLDTTINQLM	S	142			5412	LOC_Os02g08350	LSNSGTSGNLSKSEQYPPNADDKMD	S	568
5414	LOC_Os02g08364	FCAKYLHSQVLRSEAYSAGDLGTAV	S	79			5415	LOC_Os02g09740	SSWNSPSQLAGWSAGGGGDPCGAGW	S	49
5417	LOC_Os02g09920	SPAFFLEPAIVQSQNRKDDELKDWT	S	755			5418	LOC_Os02g10220	IIDEAIAEGELKSTKAYDKWAKKIS	S	188
5420	LOC_Os02g10650	EGLPVFAVGVGQSTYDKASVHYYVQ	S	112			5421	LOC_Os02g10690	KAPVARVSHADSSTGSKINGDSSVD	S	119
5423	LOC_Os02g10920	RGYGFGSGAVRASRPGWKSGDWICT	S	126			5424	LOC_Os02g10970	KKRYSSSESESSSESESELSDSDSE	S	241
5426	LOC_Os02g11750	KLEDELSAARKVSSERQLVVTKLYK	S	281			5427	LOC_Os02g11760	KIMDVLSAMRIVSSGKRPISILHDI	S	161
5429	LOC_Os02g11830	IGREVPVASMDSSGKIIWSKHNEIQ	S	311			5430	LOC_Os02g11840	LWVGDCFIYNNSSSRLNYCVGGEVT	S	246
5432	LOC_Os02g12360	YHAKPPLQAVKSSLFPIHSGSPAAT	S	284			5433	LOC_Os02g12530	MLQLGETPLVVVSSREVAREVLRTH	S	89
5435	LOC_Os02g12710	TDGWKKKKAASSSPTAWRDTAAHLA	S	247			5436	LOC_Os02g13130	INEVLAEADAASSGNLSSRKYNAPQ	S	221
5438	LOC_Os02g13500	RTFSVAAACRDDSGGAPSFSKGKKD	S	25			5439	LOC_Os02g13840	LASSGVDVFTALSGAVGALYGPLHG	S	302
5441	LOC_Os02g14530	ADLMSTWNKVLRSSPFLNKPLLPFE	S	513			5442	LOC_Os02g14530	RRKVVEEHQRTASSSPEHPLSRTRG	S	354
5444	LOC_Os02g14760	VKIFFQQLCGKVSRLRLLGDYVHST	S	299			5445	LOC_Os02g14770	TPETEYGRMNIGSRPSKRKPSGGIE	S	756
5447	LOC_Os02g15060	TRAAEREAQPGASSSSTFGLEKAAV	S	63			5448	LOC_Os02g15220	QNASRSSSSSSSSSDTGSSSSDSDS	S	630
5450	LOC_Os02g16070	RCSRSIPLFIEISKGKTARGCSIVY	S	380			5451	LOC_Os02g16090	DGAESVSDYRPISLIHSIAKIFAKM	S	522
5453	LOC_Os02g16600	LSELLNGTYKYSSLGREKGKKVLRT	S	248			5454	LOC_Os02g16620	KFLGVIVALWILSEIGSLCDFLTLF	S	160
5456	LOC_Os02g16890	LFFVIKRALVRASCNGLLHQVGRSR	S	27			5457	LOC_Os02g16890	MAVPRLGFGFYWSNLFFVIKRALVR	S	13
5459	LOC_Os02g17980	GMLGVLSRVQTNSTQEKCWIEGTVP	S	455			5460	LOC_Os02g17980	HAGCVDLEILNSSFITLVPKKSSPE	S	81
5462	LOC_Os02g18000	VPHNVRHLMILDSEKFDCSNLLSLC	S	560			5463	LOC_Os02g18660	IIAAVGDSLERKSIKYSDMYEKVRR	S	243
5465	LOC_Os02g18910	QYGAYGGRQEGSSAISNHDEFVLEK	S	27			5466	LOC_Os02g19150	LDEHNKESTLRRSEKVDGEPDIDTE	S	547
5468	LOC_Os02g19220	QTSESSDDVTEKSASKTLDQEPMLA	S	518			5469	LOC_Os02g19330	IISHGGSISRQSSTPDWAQDSYWPT	S	109
5471	LOC_Os02g19420	PALSPPTLSLGASNRVCNALALLQC	S	80			5472	LOC_Os02g19804	DGSSEPRRFQYESSRTIQASTDSRA	S	83
5474	LOC_Os02g20570	DSDFAGDLDRRRSLTGYVFTIGGCA	S	987			5475	LOC_Os02g20970	GTPKGGKASTPRSSFSSRTNNESES	S	449
5477	LOC_Os02g21350	GRSPSWGSAISQSFRQVEAEDPFRR	S	27			5478	LOC_Os02g21920	SADEKIYFNGCRSFLLWQKAARDTM	S	100
5480	LOC_Os02g22090	IAPSLQPEVFPDSEGDVATQSAKNK	S	1400			5481	LOC_Os02g22370	KLEGELWDERISSSSGQNSHLVSKA	S	246
5483	LOC_Os02g23900	LVDEALLLKEARSWSHANLPYANVF	S	33			5484	LOC_Os02g24080	SGRRSFFASKKPSRSSNPFDSDSDD	S	53
5486	LOC_Os02g24330	DAVQAVGESEDGSDKLNTAEAARKF	S	423			5487	LOC_Os02g24330	EEEKGEMQPKSRSSNPFGAARPREV	S	295
5489	LOC_Os02g24800	LNPNSIAFLSIFSHLCEAYIGVEPF	S	89			5490	LOC_Os02g24800	PRSREATPNGLASDLAAGRGLPAAV	S	345
5492	LOC_Os02g25480	FEEHARAERFNTSKSLFACRLTEGN	S	153			5493	LOC_Os02g25580	SERLHDKMSNFVSKNKAAQLQQART	S	338
5495	LOC_Os02g26140	KFLVKSPPSSLGSKLKSHGKLLPSR	S	286			5496	LOC_Os02g26349	MMKMMGIPVGFDSTKGKHVPDADVS	S	157
5498	LOC_Os02g26740	KRTKISESGAYTSSSNQDTEEENRR	S	560			5499	LOC_Os02g26740	VNWTEEENIRLLSSWLNNSVDPING	S	427
5501	LOC_Os02g27790	TEPVNPAARSLASYSGHAIASSSSG	S	127			5502	LOC_Os02g28520	NDINGGNTASKSSGGTFSGYTFILP	S	23
5504	LOC_Os02g28980	YTGTLLDGTKFDSSRDRDAPFKFTL	S	132			5505	LOC_Os02g29400	EGEGILASFSRSSVAAHGREAATMA	S	25
5507	LOC_Os02g30140	ERRTGAKPLALVSSSAAEASSPCSA	S	106			5508	LOC_Os02g30230	CGIRRDLLDEYLSALGVERLSIDEV	S	276
5510	LOC_Os02g30240	VKARSAAVRAKTSAVRTRLIVLGIL	S	31			5511	LOC_Os02g31150	AMLFEHTSFSPSSGPHGDFMFSLDD	S	624
5513	LOC_Os02g32030	AYLPVIESFGFSSQLRAATSGQAFP	S	783			5514	LOC_Os02g32030	ETVLEKSCRTVMSKSPNKHNRLYME	S	578
5516	LOC_Os02g32340	RDSSGTIPGDTLSTTYFPRLDGTDY	S	587			5517	LOC_Os02g32469	HGHGHPHPSPPTSPAAGGASAAPRP	S	91
5519	LOC_Os02g32550	AHEQQQPRAAATSSVSGRRLEAIGE	S	237			5520	LOC_Os02g32550	VHSSHVVAHSNGSTPAAALSISSPV	S	424
5522	LOC_Os02g32690	PDPDIDTYMKAISVEGLERSMQTDY	S	304			5523	LOC_Os02g32760	YLLATLAGNPNPSAEDLTTILESVG	S	19
5525	LOC_Os02g33320	SRAHSPSHSAHSSSKSFQPTQPEGL	S	415			5526	LOC_Os02g33360	KWVETIPLKKVDSGDAIQFVQEHII	S	1600
5528	LOC_Os02g33610	KISIVGKDSSDPSSAPVKSSKKNKW	S	779			5529	LOC_Os02g33770	IDEDVDMDYGTGSAARRKHAMLKRK	S	124
5531	LOC_Os02g34080	VNKINTSIGQDPSSKLLIGVLDIYG	S	425			5532	LOC_Os02g34080	YRQQTKAAVIIQSYCRSYLAHSQYM	S	839
5534	LOC_Os02g34460	GGAPAEFQPSFRSSGGRPGFGRGGG	S	158			5535	LOC_Os02g34500	DDTPIEVGGFDSSKAKSDMICSNMD	S	711
5537	LOC_Os02g34560	PIVGDAWEALRRSLVFFRGQPLGTI	S	107			5538	LOC_Os02g34570	SDGALETQSTKPSSDGQQINKEPPS	S	321
5540	LOC_Os02g34840	ESADDDIVVAQISKNRRVAVRTWNG	S	41			5541	LOC_Os02g35110	EATATDFYLRVRSRPIIEDCCGVRF	S	273
5543	LOC_Os02g35130	SEDAGKEIVKCMSMAKDGIHAVLMV	S	95			5544	LOC_Os02g35150	CGEYMRSRFKTCSRGIACNFIHCFR	S	315
5546	LOC_Os02g35190	TDGSGGLWRRNGSSSGALLRYNDSG	S	35			5547	LOC_Os02g35690	MPSPPQVSASSESSDGFPPEPPMAA	S	225
5549	LOC_Os02g35800	EQAKAHNLSPSISGDDGEPNPCRRA	S	15			5550	LOC_Os02g35910	GPLRCWPGGLCLSRSIGDQDVGQFI	S	220
5552	LOC_Os02g37030	PICYEDLDPTDSSFLPCPCGFHLCL	S	289			5553	LOC_Os02g37030	RSFSSGSSAWSSSRSVTDSDDDTGG	S	154
5555	LOC_Os02g37130	SLPLVKIPGPESSKENNGVMWISSP	S	123			5556	LOC_Os02g37856	DMVLLFDIGCVSSTPTEILRKGRSR	S	184
5558	LOC_Os02g37880	PIEQSNSSLSHGSSKAGDAPLGAFW	S	350			5559	LOC_Os02g37920	DDEVTPENQKVPSSVTTRVATGSTP	S	405
5561	LOC_Os02g38220	GGGFRDSPTSTDSDRWSRAAPLPPH	S	234			5562	LOC_Os02g38220	KVTAHLSNSPDESQITESMEQPRSR	S	467
5564	LOC_Os02g38480	QLDGCMTELQQKSDAMQAAESRVEE	S	391			5565	LOC_Os02g38480	VEHKLSTTKEKLSMAVTKGKSLVQH	S	353
5567	LOC_Os02g39010	RRDGGRSGGRELSNGYSHRRDSPRP	S	58			5568	LOC_Os02g39030	TALMEAVSSGKLSAREHLESCSNEE	S	88
5570	LOC_Os02g39210	PSHVADFMRRRCSWRAVPGVDGDDD	S	98			5571	LOC_Os02g39420	WTLNPPGALPWSSELHELTGYSSQD	S	366
5573	LOC_Os02g39550	SSLLILSRAFTVSGKMKPEEAVVAT	S	483			5574	LOC_Os02g39700	LLKADEIIKQVDSRTVAASPLPYNV	S	120
5576	LOC_Os02g39750	RASSCAATASTSSPPPAHPAAAVQP	S	150			5577	LOC_Os02g39820	GVEPEEGGAVELSDDLWAAVWEVSA	S	134
5579	LOC_Os02g39920	DDLPEDGSNKSASKPKEASSGGKDL	S	672			5580	LOC_Os02g40430	NSSEDGYALTPKSSYAFGRFSASSL	S	1022
5582	LOC_Os02g40690	QWSRKNPSHPAASAADDDMDDDAIT	S	52			5583	LOC_Os02g40880	EEADVKNKWENSSWGKKLIVQKRRA	S	88
5585	LOC_Os02g41890	DASALLGFMQGLSGSGSGWTVPNAT	S	55			5586	LOC_Os02g42040	TAKGELSDENTASYEKLRKSFDHLQ	S	211
5588	LOC_Os02g42210	KELYRSLGEKGCSANAATYKHMMAQ	S	313			5589	LOC_Os02g42550	CQTESDSIKNEDSISLGLDTKSDPT	S	226
5591	LOC_Os02g42560	AEATTSPQVKRKSGKIAADDKGKKT	S	31			5592	LOC_Os02g42580	CCFERALAAGTSSAACSKVAKQAAL	S	197
5594	LOC_Os02g43270	RRAARVKAAQDGSRGTSSVSPAVAS	S	314			5595	LOC_Os02g43740	CGDIGSVYLSELSGTRCYFAMKVMD	S	314
5597	LOC_Os02g43870	AFSSDLVRQSKKSGKEKNLNAFFEL	S	372			5598	LOC_Os02g44050	RKEEAWTWNPSGSGVAASAAPGSAA	S	405
5600	LOC_Os02g44642	TDVTFVGSAQCSSPGSGQTSGHNSV	S	285			5601	LOC_Os02g44654	TLRTAMSRWVSRSIHHRLLPILDDA	S	154
5603	LOC_Os02g44740	YQPRRSGRVVTVSVVGKQVPLYGAG	S	253			5604	LOC_Os02g44870	HHAEGEKKESLLSKLHRSSSSSSSS	S	77
5606	LOC_Os02g44970	LKFGLSGDKFIGSSLIVLYSKLHRM	S	68			5607	LOC_Os02g45054	HGASPDLLRFGGSGGGAMAARLEHL	S	284
5609	LOC_Os02g45110	GDDLAAMREQCRSLEEAIGFRRETQ	S	21			5610	LOC_Os02g45110	NLTKEDCRRQSGSFVACDKVHFRRI	S	394
5612	LOC_Os02g46962	SRGVRFPGRDNESLAPIFTPARSVA	S	189			5613	LOC_Os02g47130	DNADNFSDDETESDATVVISSASKA	S	531
5615	LOC_Os02g47150	KVGSSAIVLNLLSWAEFKLSKELQK	S	413			5616	LOC_Os02g47170	LGPDELPPSQIMSPRSGTPVKLLGR	S	55
5618	LOC_Os02g47220	KICNVIGSPDEQSWPQGLSLAETMK	S	222			5619	LOC_Os02g47220	PHSNASLKSSGLSKTGVQRKLQMDR	S	343
5621	LOC_Os02g47860	QNLALPNETCNGSAPTNLFVSHLGN	S	549			5622	LOC_Os02g47970	RLFLDSIGLSDLSAKEIKKWMPEDR	S	1281
5624	LOC_Os02g48000	SMTEYVSKQKHRSNDPGRDLSIQVL	S	259			5625	LOC_Os02g48010	RQDIADDVQMESSLGVADNCVDIHG	S	863
5627	LOC_Os02g48640	AGGSRAEEWGPGSSETVQTGDVVEE	S	30			5628	LOC_Os02g48730	IKDQLEKDKDDESLRRWKEQLLGSV	S	218
5630	LOC_Os02g48910	AWTQKHSSTPIVSHYPPYAVIGSTP	S	174			5631	LOC_Os02g49080	YLAELLAERHKLSPFIPVLPNSVRL	S	50

5633	LOC_Os02g49150	ASESLKTGGKASSKGQTGKGGNAGK	S	83			5634	LOC_Os02g49150	GKGGNAGKGGKGSASARVSAKSDAE	S	100
5636	LOC_Os02g49320	KGEVTGHWYGYVSPDDVPVLLHKHI	S	272			5637	LOC_Os02g49320	RRRSHMADPSRSSPTAAAAGDALLA	S	37
5639	LOC_Os02g49590	PLHYYVFNCLLFSLLVLNIYWWVLM	S	261			5640	LOC_Os02g49620	KTTDTETGTAVGSVSLAAMQEKMAN	S	305
5642	LOC_Os02g50530	KLWCPETKKVVISRNVVFHESVMLH	S	521			5643	LOC_Os02g50530	LHDKPSTNVPVESQEKANVQVEHLI	S	544
5645	LOC_Os02g50570	LPPGRESPSRPQSIAAAAVASPAPP	S	302			5646	LOC_Os02g50570	RLAINRNYSLRRSGSLAKSTDGGNP	S	676
5648	LOC_Os02g50700	LACSHGYVPRFSSATLQKETRPVFP	S	19			5649	LOC_Os02g50760	SDDDQTVKKEKKSKSKSMEGSRSDD	S	399
5651	LOC_Os02g50790	LAEKERNIAGLSSQRIDSHGEDDLH	S	221			5652	LOC_Os02g50799	LRKSNMDEEARMSAKVAELERETSE	S	222
5654	LOC_Os02g50850	SCSSSQSLNNSQSCSSEHAKTIGSS	S	492			5655	LOC_Os02g50880	NFSLPWQRKRQYSSSSSGFIIGGRR	S	130
5657	LOC_Os02g51000	VAQRRSDLFLDPSAPGVVAEMAVEA	S	108			5658	LOC_Os02g51200	HEIPLQNRKFTWSNERRRATLVKLD	S	943
5660	LOC_Os02g51400	SSKGGVGSAEARSAAAAEGSSGRCS	S	64			5661	LOC_Os02g51490	PQRVICASNSYSSNYRSTSTSSPMN	S	25
5663	LOC_Os02g52280	AIKLYSLLHDVLSPEAQLRLCGYFQ	S	562			5664	LOC_Os02g52810	EEEEGEAVSDSFSHSLRECQKQRKL	S	112
5666	LOC_Os02g53270	KRLWLDGFEKLSSILTLKELSDLQE	S	1128			5667	LOC_Os02g53420	ANGIVTVSAKDKSTGKEQQITIRSS	S	537
5669	LOC_Os02g53520	SVLKRLTEEAGRSPNNEKIQMEMKK	S	685			5670	LOC_Os02g53700	VEEIVQRPSKLVSMLTSEKPVFPRR	S	254
5672	LOC_Os02g54110	EEAILAEDEISKSERRAKERYTKEF	S	630			5673	LOC_Os02g54170	TVQKMADALHEGSALSHLSLGNNNP	S	105
5675	LOC_Os02g54200	MELMKNSLYFFKSEMKRPISELLDY	S	382			5676	LOC_Os02g54280	VVGRIAKRVVELSQFDVHFVPRTAI	S	1423
5678	LOC_Os02g54490	KTLMKQFNELDESLEEAFSMPFTTI	S	557			5679	LOC_Os02g54500	GWKFRGLTVWSVSGCRLMCTIRQTG	S	334
5681	LOC_Os02g54510	DALNSLGDVRTPSSPVSVEQLFGIG	S	164			5682	LOC_Os02g54550	IFESEKIWFQPESPKHLAPLLRNLR	S	313
5684	LOC_Os02g54910	RNRRNQTSKLLGSMIPGAITRALDP	S	275			5685	LOC_Os02g54990	ACACEACRTAVGSIGRRSARIAYCG	S	25
5687	LOC_Os02g55070	LLEKITQQLLSDSHVAPASDEKRVM	S	435			5688	LOC_Os02g55940	DDIKKTRSMQGLSFLANIGGPATPQ	S	295
5690	LOC_Os02g56270	PPQTAMAASQATSISRGASQPLAVP	S	700			5691	LOC_Os02g56270	QSSSSAPPETILSALQVSQSEPVKK	S	736
5693	LOC_Os02g56640	TKASGAATTGSASSRRRARRAAAAV	S	141			5694	LOC_Os02g57080	EIAVKLLTREDRSGDREFIAEVEML	S	380
5696	LOC_Os02g57180	KNWGFLPLVDSGSTKIQPVYYVVDVA	S	257			5697	LOC_Os02g57390	AFDSPNPIRPDSSRAQLQSRSESMN	S	244
5699	LOC_Os02g57470	SLRLVSPLLPSSSSAAAASGDDDAQ	S	184			5700	LOC_Os02g57500	KADEDESKRFLKSQREALLKCLEIA	S	306
5702	LOC_Os02g57510	VDKFKMVFETKSSRRMGTLVASFVA	S	100			5703	LOC_Os02g57640	KISDRGDFIAGTSERKVTITGTSEA	S	313
5705	LOC_Os02g57690	LAAEGSRCQKLESQIAAAHKRLEST	S	461			5706	LOC_Os02g57690	YVVGGSRNGRYLSDIQVFDFRTLKW	S	60
5708	LOC_Os02g58090	TASDPSLLVYLKSYRNTVPVPRHWC	S	193			5709	LOC_Os02g58110	KASTKVPLEGKTSSGSSTQGERKIL	S	35
5711	LOC_Os02g58210	WPEMLKLKDWPPSSSFDQRLPRHGA	S	594			5712	LOC_Os02g58340	RIVEAQIKEAVLSGVALEDEQREKF	S	214
5714	LOC_Os02g58410	QNQKAYYVASNISVWCRVKSLDDLA	S	1919			5715	LOC_Os02g58570	PSDDDDDANSSGSDVEEHVEVERRL	S	178
5717	LOC_Os03g01080	RPVGVQASGPEGSVPGSPRLIAWIT	S	1380			5718	LOC_Os03g01470	ATGGGSGPPAARSSEDCRGGSSELA	S	86
5720	LOC_Os03g01970	SSALAAATSAAVSSHTDRDRIIGVF	S	40			5721	LOC_Os03g02010	FGGRKKTRFVDGSKKRKRYGSGPSG	S	256
5723	LOC_Os03g02090	DLCDWKIAFFHPSKPHVVYFSQKQH	S	284			5724	LOC_Os03g02150	TDVVKNVNKNNASHAVLFEALALVM	S	298
5726	LOC_Os03g02230	AAQAQVGMAGEGSAPIAVGKAASAR	S	135			5727	LOC_Os03g02230	ASSSRAGWRKKKSQGHFRPGWHFGE	S	168
5729	LOC_Os03g02270	LLGELKDSGEPSSGRSTLSEGNGIG	S	80			5730	LOC_Os03g02310	MEKSEEWPAIFKSTSKEGSEAGTQE	S	112
5732	LOC_Os03g02320	SPGIVKLGDFGVSACMFDRGDRQRS	S	177			5733	LOC_Os03g02350	VPRRDNMIADELSRLASSRAQTPPG	S	1556
5735	LOC_Os03g02480	AIFFYWVTSNLFSLGYGFVLRKPAV	S	358			5736	LOC_Os03g02680	RAPEILLGSRQYSTPVDMWSVGCIF	S	220
5738	LOC_Os03g03430	IKEPGASIEKTYSDRSDYKTNSDRS	S	321			5739	LOC_Os03g03460	FVFASHTFDVWESWMLEGSLLEGCK	S	380
5741	LOC_Os03g03550	KRVLANRQSAARSKERKMRYIAELE	S	292			5742	LOC_Os03g03550	YLNLEGLDGLNSSDDRHDEGDSRGS	S	144
5744	LOC_Os03g03830	PSMGFNQQPPPSSTGFMRPTQPGAP	S	215			5745	LOC_Os03g04330	NRRSHKPHASDASFPLTWIGATPSI	S	124
5747	LOC_Os03g04460	LSAGAEVGFDSTSASVTKYNSGICY	S	152			5748	LOC_Os03g04680	EVVPGHVVEPRLSVILHMKNGLLVR	S	516
5750	LOC_Os03g04710	RLTPGGSDAHDRSLGGLRRACSDGN	S	61			5751	LOC_Os03g05200	GEATTESSSPSASRTLAGDEPAAAG	S	64
5753	LOC_Os03g05330	LLAPLMEVDGLSSLLSKKYFTSDRR	S	901			5754	LOC_Os03g05330	SQSPDAVVRAASSSALGLLIKRSNV	S	1002
5756	LOC_Os03g05460	RRSNRWSRVWDRSITSPLKEFVRKG	S	309			5757	LOC_Os03g05480	ASKVQPFRDNAESVGGSINSTGNGH	S	403
5759	LOC_Os03g05812	STQPPNIRPPPISESQEFVPEKPAK	S	456			5760	LOC_Os03g06260	GTGRVVKWAIELSEFDLHFEPRHAI	S	1216
5762	LOC_Os03g06340	LVMHAYNPPNVDSFIDHLGLHKALC	S	271			5763	LOC_Os03g06410	FMDTSMPSRNPESVSPSFARSHKLD	S	707
5765	LOC_Os03g06710	LKSLNADTNLYASNCMIDLYSDHCM	S	637			5766	LOC_Os03g07380	IREMRKQNKKELSRTSLGGDDDLLA	S	57
5768	LOC_Os03g07580	QVTVWELIRVLFSERATGNQLEPTG	S	473			5769	LOC_Os03g08220	KRVFFSNKPYLPSETPPGLRELREK	S	225
5771	LOC_Os03g08360	LVVNCSLFNPTPSLSAMIVNHYKMR	S	234			5772	LOC_Os03g08430	PVSDCNVGKNNTSSELLPMQPLEDI	S	635
5774	LOC_Os03g08820	GQPSNLQRNNAESSPHGKKVLQREL	S	846			5775	LOC_Os03g08820	KRCSANENSSVMSGSAKRAKADYLG	S	644
5777	LOC_Os03g09260	EALDGENQYFCESCQKRVDATRCIK	S	278			5778	LOC_Os03g10070	SLRAYVQIVDVASILRLDRDIADHA	S	162
5780	LOC_Os03g10140	LRCLGLDMSSSSSAPPSSSGQQQQH	S	407			5781	LOC_Os03g10180	LESSIANNKFHFSFYKWGGKGAVLV	S	224
5783	LOC_Os03g10460	DSVYEEEIPGNISFLEENYSKAKFY	S	236			5784	LOC_Os03g10590	RYRFNNSYDGRNSMHQAAGDQAEKW	S	321
5786	LOC_Os03g10710	SDDEGDDEVVVFSEKEEAAMAKIFD	S	47			5787	LOC_Os03g10740	RGVGGKPYRNDSSQKDATRIKAGIP	S	22
5789	LOC_Os03g10990	KLKANGASCLAISCELLDAIAKIPW	S	285			5790	LOC_Os03g11050	SATIIGDVYIHPSAKVHPTAKIGPN	S	306
5792	LOC_Os03g11210	HLSKPCEVSFKDSSVIRYAPRVKVT	S	99			5793	LOC_Os03g11220	NKKPSGGYGYGASSGGGHGGRYEED	S	284
5795	LOC_Os03g11240	SGKAASDSNSVSSSYREDNGFIMNE	S	583			5796	LOC_Os03g11500	IHKQAAATDILMSFIHGLVLAHLMQ	S	402
5798	LOC_Os03g11890	GLLQVTPQEGMASIDGTANAVEPEK	S	518			5799	LOC_Os03g11910	MRSYVMFKDDSLSAGVTGGATREHG	S	72
5801	LOC_Os03g11980	QVWPKPTSISWPSAVYAPLSPSFSV	S	59			5802	LOC_Os03g12120	PNLDKLSYFNVVSNMHIGNQINDHD	S	291
5804	LOC_Os03g12250	YNALTGPIPDDLSRLPELRAIYFQH	S	113			5805	LOC_Os03g12300	KILRTRLKTVPFSENLKRTSSTNPY	S	612
5807	LOC_Os03g12360	DEDSLLRHLQHSSSSPAMEFIYIGC	S	99			5808	LOC_Os03g12450	HWTTTREAEFLASTIVSSLTEKQQK	S	659
5810	LOC_Os03g12590	AISKMVSGLTRGSVRSPLAQCLLIR	S	236			5811	LOC_Os03g12590	EPSEEPFDISLVSREVKSQPLQEKK	S	599
5813	LOC_Os03g13190	PLPRAFLAAAASSPRRAAASPAPAP	S	38			5814	LOC_Os03g13190	PPFTGRPLNPNPSHHATAAHGILYP	S	65
5816	LOC_Os03g13614	GEVATRSKSQKSSATQNEQSTPTNP	S	16			5817	LOC_Os03g13720	AFCKAPFDDFLISVVTERHLMVFDI	S	338
5819	LOC_Os03g13790	SFNLPASRKREGSNNSASSSPDSDS	S	587			5820	LOC_Os03g13790	TVNADARMLSPSSSNACTVDGLGTN	S	251
5822	LOC_Os03g13970	PPGENALSDVLISTPIFTGEEGGSG	S	186			5823	LOC_Os03g14250	EQDDKAPKYVQISPETYAHLTESEE	S	41
5825	LOC_Os03g14260	EEEFVPQQQKSTSSNKILGLDNILN	S	836			5826	LOC_Os03g14450	VYKSFVSEYPIVSIEDPFDQDDWVH	S	299
5828	LOC_Os03g14710	ITVALLCIQRSPSRRPSSEEVLEML	S	634			5829	LOC_Os03g14860	AAAAAASDDGEYSARAPAPQFVTEF	S	39
5831	LOC_Os03g14980	GKIKAWLYDNLGSRVDYDAPGHWCT	S	537			5832	LOC_Os03g15010	QVSADRGVDVNISSNSTSLIQGDRR	S	787
5834	LOC_Os03g15040	WMVSKFEKEHNHSLLSSRAVPITSN	S	298			5835	LOC_Os03g15050	FPCNRYTHYMTSSTSVDINLARREM	S	301
5837	LOC_Os03g15160	PHAGWEVTAVILSGSPERTSLAVTA	S	83			5838	LOC_Os03g15420	DLVGDSKALVAASDVVTSSAIAESY	S	28
5840	LOC_Os03g15560	DSVTKQISGIAISESLVVATSSTDA	S	141			5841	LOC_Os03g15580	GRSAGKTRKPKKSKLKHGRSADCND	S	727
5843	LOC_Os03g15740	FVRAAEMYTRVLSIRPNHWRAQLNK	S	480			5844	LOC_Os03g15810	ANEGDKRNEVSVSEKKSALVSTKST	S	899
5846	LOC_Os03g15940	FKCSHGGCAISPSNYAALEGILYCK	S	145			5847	LOC_Os03g16310	HEIPLQNRKFTWSNERRKATLVKLD	S	190
5849	LOC_Os03g16400	EVRKKVQPGQSSSSDAEIEYRRARS	S	349			5850	LOC_Os03g16450	DDGFQPDVVTWTSRVSGSVLNFQYG	S	190
5852	LOC_Os03g17020	ASVVQVGTKEFQSKMREGGYDQKVN	S	293			5853	LOC_Os03g17050	DDAPSDPCSTQSSQESNIALTDSLK	S	500
5855	LOC_Os03g17240	SGNRLDSSGPSPSPSPTAAAAAAPV	S	128			5856	LOC_Os03g17490	AKKKARLERFGQSTNVDKGEEEKRK	S	130

5858	LOC_Os03g17710	DDRRSRSLSYSRSPRRSISPAANGK	S	215			5859	LOC_Os03g17710	GRSPSPRNNGSPSPMDNNSRSPRDN	S	294
5861	LOC_Os03g17950	PISSPSTPWSRKSSGSAKGIQTEAM	S	224			5862	LOC_Os03g17980	EDEARRIFQQIISGVEYCHRNMVVH	S	169
5864	LOC_Os03g18180	TTKMQEHAGSDKSCVWHALDFADGE	S	119			5865	LOC_Os03g18310	YYEKVYEPVEEGSYVKMIDMVSGTG	S	412
5867	LOC_Os03g19040	SDPEMKRRRRVASYKAYSVEGKVKS	S	89			5868	LOC_Os03g19220	TATVQRPRLRLSSPSSRERLITSSL	S	66
5870	LOC_Os03g19520	LVLSDMSYLEKSSGNEQLILTSVIR	S	335			5871	LOC_Os03g19520	RLQSDLGNYGSDSDQEIARSVLSDC	S	754
5873	LOC_Os03g19870	DFEAFQTALDSDSSYSSTFTRSLSL	S	251			5874	LOC_Os03g20340	SGKDHSLLLILPSGVYRYRFVVDGE	S	155
5876	LOC_Os03g20530	ENSDSFSRKQENSDSQIDRPNNDKE	S	337			5877	LOC_Os03g20530	RSSIAALPMNGKSKEPFHDVRNGAK	S	384
5879	LOC_Os03g20600	QACVCAPTTHPGSFRCKHHRQNASN	S	71			5880	LOC_Os03g20670	DPSTDFRGGGFISLENLIFFARNYP	S	184
5882	LOC_Os03g20860	IRSLIGSIYKAESTATGLMDRLRTI	S	162			5883	LOC_Os03g20910	YFLVTIHLQALTSWSHRSLSTPISQ	S	333
5885	LOC_Os03g21080	RILQGSVAVQVNSGVLSVCTAFLSG	S	885			5886	LOC_Os03g21140	DSDQSLEHNLPDSPFASPTKSSVSA	S	610
5888	LOC_Os03g21350	LDLLQCTDQEKVSFASHQLHGPASE	S	66			5889	LOC_Os03g21380	NGDGEITLDEMASALDALGLGADRA	S	54
5891	LOC_Os03g21800	MEAKKAMTPEQLSELAAIDPKRAKR	S	126			5892	LOC_Os03g21800	QDPLGRLQGLDISKGPLVVKSESSS	S	307
5894	LOC_Os03g22190	DNLEKCSQRPTQSKLNQCHSLLSFV	S	1025			5895	LOC_Os03g22580	KGGDNENLNERLSKIKKSEARPEEV	S	350
5897	LOC_Os03g22730	QDAKDVAMETEASGKKDKKKKKKKH	S	553			5898	LOC_Os03g22740	EVEAQLKEAAVISMGTEVSELDLLN	S	240
5900	LOC_Os03g23970	CGLQLYRYGETISIPFFTETWRPDS	S	137			5901	LOC_Os03g24050	GLSAFTLWLLRHSSLMGSSDIDNWL	S	297
5903	LOC_Os03g24220	HNNKVLQVDAVSSNLSPTDCFVLQS	S	546			5904	LOC_Os03g24339	SGWSMPPRSSNYSPSRHSMEPPVPH	S	1471
5906	LOC_Os03g24890	ELNHQVIGGREISIVFAEENRKTPQ	S	133			5907	LOC_Os03g24890	RYMDGSHRRRSVSRSPRSRYHSYSP	S	168
5909	LOC_Os03g25260	RRMATRMASALASSDNRAQAALARL	S	32			5910	LOC_Os03g25464	PAQTKVKGIDRSSVAIVTIICAVEC	S	50
5912	LOC_Os03g25620	SLTGDAAAGDHFSSASGPLPDHVRR	S	70			5913	LOC_Os03g25620	TVPGFGDDDAFDSSSLSLTGDAAAG	S	54
5915	LOC_Os03g25890	TYLENIDRNFRSSSKSYASSIIKRL	S	35			5916	LOC_Os03g26000	GHTLLFALDGSPSELLFERLPEIDY	S	232
5918	LOC_Os03g26460	KLNLKPEGQFIFSAKGPADDTPYEL	S	128			5919	LOC_Os03g27080	DEKWSDQGSPSGSKDVVTPRNQSLK	S	121
5921	LOC_Os03g27260	ERRRKSVRGCIVSQDLSVINLVIVK	S	105			5922	LOC_Os03g27260	GYGRRDGERRRKSVRGCIVSQDLSV	S	98
5924	LOC_Os03g27780	VMKPGEGISVHGSIPRNSRVDPWGG	S	400			5925	LOC_Os03g27840	RRRGDPHQSLQVSGARCRVHRDDAL	S	44
5927	LOC_Os03g27990	RLVQRSSMGSVLSGGESISRRYDNS	S	736			5928	LOC_Os03g27990	SLKYLNLGHNQLSSINVMFNQLTNL	S	170
5930	LOC_Os03g28980	TSGAWTDTHCSWSPREGSDWIVFSS	S	558			5931	LOC_Os03g29260	LQIMMTIVDDLVSVDSLIEDYFYTE	S	197
5933	LOC_Os03g30390	LYYMEKSNARKPSKATDILGQHDGK	S	385			5934	LOC_Os03g30570	GGGERRAANGSGSAVGGESDEIELS	S	25
5936	LOC_Os03g30790	DCTRYAPGIEIISVRVTKPNIPDSI	S	215			5937	LOC_Os03g30860	APQSVGNNKYYVSFIDDYSKFTWIY	S	536
5939	LOC_Os03g31730	IKKENTNIGHECSDENVHTVRKHHS	S	739			5940	LOC_Os03g31750	KQLHLSVLAVFNSWDSARAKKYRSI	S	228
5942	LOC_Os03g34300	AGSSTTFLSPPSSPRSGIKNPLLGD	S	314			5943	LOC_Os03g34300	VTVVCYLSTFAFSGLLFTWFNPSGH	S	198
5945	LOC_Os03g36530	DDMVDFDMTYYRSRDPNRARLFEDV	S	480			5946	LOC_Os03g37330	FGQAPDRVVEGFSEEFLDAFLTLLR	S	69
5948	LOC_Os03g37554	YLDISCLMLATMSPKLQRQYEALDA	S	136			5949	LOC_Os03g37810	DGSFKKGGGGGGSSGNSEKKTVAGN	S	32
5951	LOC_Os03g37984	LSNMGNFVTEMSSDSYQLLGMAERG	S	384			5952	LOC_Os03g37984	YEVSGGPFGIEDSVKAAGPLLAIAG	S	125
5954	LOC_Os03g39020	PMIESALAGFNSSLVCYGQSGTGKT	S	169			5955	LOC_Os03g39380	VAALPHLVKSLRSEPVSNAPRLRHL	S	21
5957	LOC_Os03g40010	DTASKKKAATGSSHKTGTPRTVSKS	S	539			5958	LOC_Os03g40010	NSRKRKRGSTPKSSSKKFDDDDDIT	S	467
5960	LOC_Os03g40180	GITQLKFQRNKRSVAEERAGRKLGG	S	100			5961	LOC_Os03g40550	PQFLCSLPCSTNSIRSHLHYKPHFL	S	23
5963	LOC_Os03g42770	HIQAFSGHRGAISCLSFGPDSSELF	S	265			5964	LOC_Os03g42810	IPARKLIEENRQSKLRGKDQLLQEA	S	236
5966	LOC_Os03g43180	TTTDDDVRRPATSSDDGTARLDVNG	S	47			5967	LOC_Os03g43670	VAEIAFAVILVFSVTAIACLYVRYK	S	437
5969	LOC_Os03g44484	QRLKHMVDDKIHSRGRGPVQILTRQ	S	407			5970	LOC_Os03g44800	SPAARNGSEEEGSDSDGGRSGSGSD	S	316
5972	LOC_Os03g44900	IVPTTTAAVLPASVTARSAPENIPA	S	353			5973	LOC_Os03g45180	YGWSNLANGGLVSPTLTKRVVPDNQ	S	438
5975	LOC_Os03g45300	SKVGPGNSEWLYSVYNCRQHWVPVY	S	446			5976	LOC_Os03g45344	TKGAVLCATCAGSGLYVDSILESQG	S	109
5978	LOC_Os03g46046	GQEQQQDVQAGKSRRLGSARKLVHR	S	96			5979	LOC_Os03g46330	ACVDGFLGVAKISAFHHLEDVLDDL	S	556
5981	LOC_Os03g46340	GILQSQVQREQSSTKQVCEERSESC	S	385			5982	LOC_Os03g46340	LHAVEKEKEELLSLVGILQSQVQRE	S	370
5984	LOC_Os03g46590	AAECGIPDAVYVSQYFSKQAIYHTW	S	769			5985	LOC_Os03g46600	EEDEAGADEEDESKVANGSKGSSGS	S	242
5987	LOC_Os03g46920	RPGCAVRRSADSSPAASPCDLAALR	S	28			5988	LOC_Os03g46970	PREVIEHRLAVRSGARPVRQKVRRQ	S	824
5990	LOC_Os03g47760	MRDINLSSAETVSNLKTNTQLGKVI	S	55			5991	LOC_Os03g47800	TQRHRGFGFVTFSDPEAVDSAIKEM	S	56
5993	LOC_Os03g48600	PQRTLLSRAGSASPAEFRVYKPTGC	S	203			5994	LOC_Os03g49170	GKLRRSSSFELRSGGDDPDLSWVHT	S	683
5996	LOC_Os03g49770	DENIYFDTRDFLSSSSFKSSGSDFQ	S	367			5997	LOC_Os03g50120	KRQLIPGEWECPSCSFVNFRRNKVC	S	370
5999	LOC_Os03g50180	GMLNCKPATTPLSVSEKLSLHEGSL	S	606			6000	LOC_Os03g50210	GREFVSSAAELRSGCGINAKIVQKL	S	148
6002	LOC_Os03g50300	YKELGLRDKITLSSGRFLLGNKYAR	S	607			6003	LOC_Os03g50330	QIFEDDDDLLWRSPGTPVFTAPECC	S	302
6005	LOC_Os03g50340	NAVLICSDVDGGSYELYIVPKDSAG	S	390			6006	LOC_Os03g50390	VGSPDVAKVSNSSINEESDGPSPKL	S	647
6008	LOC_Os03g50810	GGAAASETYSQASSGPRDIHVVETG	S	579			6009	LOC_Os03g51000	FCLVWRPFCDESSLECVCVQIKVVL	S	62
6011	LOC_Os03g51020	NNTYDEIDDTMNSRHHKLDDAQEED	S	731			6012	LOC_Os03g51030	SLLNPILVQCKTSGKPFYAIVHRAT	S	164
6014	LOC_Os03g51140	PHILPKLVQPPLSSFNAHALGALAE	S	630			6015	LOC_Os03g51140	SAGHVFVEHYATSSLPLLLPAIEDG	S	351
6017	LOC_Os03g51200	RGKAKTTKAVSRSSKAGLQFPVGRI	S	22			6018	LOC_Os03g51220	SSSDAEPVVKENSVDVDTSRENASN	S	515
6020	LOC_Os03g51390	DCVGSGKEGGASSEETVPLPEYERL	S	69			6021	LOC_Os03g51390	RARRQRRRAGGGSHQQLLLMDCVGS	S	49
6023	LOC_Os03g51470	FRAAGEYVSQKSSSVFGKKKVEPVV	S	83			6024	LOC_Os03g51470	IKDKFRAAGEYVSQKSSSVFGKKKV	S	79
6026	LOC_Os03g52310	WWKLNTLKFPTLSRMARDILAIPMS	S	650			6027	LOC_Os03g52450	ESAAAEALLSAASEQLTLVYQGEVY	S	37
6029	LOC_Os03g52700	PPKENVLSFGKKSSVDEAPPGKGVS	S	866			6030	LOC_Os03g52700	REAVDVLFLHGSSDMVIAKQAIFLY	S	337
6032	LOC_Os03g52794	TEEVVVASEAEYSWPQLREDLPPRR	S	38			6033	LOC_Os03g52910	RGATAKAFHGDGSSHHRMMPSSSSS	S	32
6035	LOC_Os03g53280	DFSAQKDSFSHLSELENADLAGETS	S	1545			6036	LOC_Os03g53670	SNGHQRSNTNSTSYGSNSYSAKNQS	S	272
6038	LOC_Os03g53970	AAASRYASYDSPSPSPSPRRAGAAA	S	18			6039	LOC_Os03g54000	HVIITIFANCGVSYGGGDAYSIGAI	S	139
6041	LOC_Os03g55070	DYQKSRFVNRVVSSMFNTVSGKKIA	S	316			6042	LOC_Os03g55300	DDDGELGQRMQASDDAPPSPWWQQH	S	187
6044	LOC_Os03g55570	RRRLEEDFGVDLSDKKLFIREQVDL	S	41			6045	LOC_Os03g55704	RDGASTIYEENESVSVGEVMKSPVF	S	609
6047	LOC_Os03g55800	TGGYRVLSYLDPSEPNHAPLKTLLF	S	154			6048	LOC_Os03g55960	FAGFVALMERGDSQEEDLRSAFEVF	S	118
6050	LOC_Os03g56060	YEWVVTKKSGRSSEGDLISLAPKEL	S	363			6051	LOC_Os03g56250	GRRSSSVNPVMLSSMAASPAANGAV	S	359
6053	LOC_Os03g56260	KQKTNGRSMPYFSTPGKPKEMYNFS	S	679			6054	LOC_Os03g56430	VYKSANLQVSNSSSIPAADKQTRQT	S	422
6056	LOC_Os03g57340	SKKLSLSRNVLCSKCNGKGSKSGAS	S	147			6057	LOC_Os03g57430	FSLGSGGGGGDKSSRRVIKVKRNPK	S	1128
6059	LOC_Os03g57820	PSAPPAQDRHAQSYDVQLQYDMDFG	S	506			6060	LOC_Os03g57854	DYSKPHSIHVPESDIGYHFGTLLDN	S	202
6062	LOC_Os03g58740	ASPQPPPPKPAPSRLESSLSFSAPL	S	247			6063	LOC_Os03g59020	TEIELARRLKEFSLKETGLDQYAIA	S	431
6065	LOC_Os03g59340	THSQISGEIPGASPDHMMSPVGNIG	S	159			6066	LOC_Os03g59480	ASPPRRLKRLKKSSQIATAAHPVVG	S	32
6068	LOC_Os03g59740	THLNFLIWLTGISRALVLQQVRDCT	S	166			6069	LOC_Os03g59760	DDLRGLDDIGRDSDRDRERERRERE	S	134
6071	LOC_Os03g60110	LHQERLGFPQGTSMDWQGAPPSPSS	S	117			6072	LOC_Os03g60110	NSTLREDSPHPGSVSPFSNGGMKRA	S	265
6074	LOC_Os03g60180	IREKTSIASLLSSRRNWSGGGVDGG	S	19			6075	LOC_Os03g60240	HHAVIAHSSDKQSSQKSSGLDGSSI	S	551
6077	LOC_Os03g60400	LEKIGIEAKQPNSAIRKCARVQLVK	S	63			6078	LOC_Os03g60460	AVHSASTASGPPSPIPFSLADAADP	S	69
6080	LOC_Os03g60870	IGDSGVGKSNILSRFTRNEFCLESK	S	30			6081	LOC_Os03g61150	LLMTLIFGYMCSSCEEEQVADEGGG	S	16

6083	LOC_Os03g61160	SSSTSSEPNATTSPLASPRAAANVV	S	58			6084	LOC_Os03g61560	DPFQRRHELLIKSELAEVLIQTLIE	S	508
6086	LOC_Os03g61640	PLKHRDYEVDLESRLGKTQVVTPIA	S	62			6087	LOC_Os03g61640	RASLEQVQKRFESLKKRKDPGSFSE	S	130
6089	LOC_Os03g61930	TYGKPPVSRGGQSVTLVGTTLVLFG	S	298			6090	LOC_Os03g61930	YAGFGAGGATSSSAISRFQNDVALL	S	30
6092	LOC_Os03g62600	KRWLKELRDHADSNIVVMLIGNKID	S	122			6093	LOC_Os03g62640	LADEDAKAAIAISVSEVTQTDEVLQ	S	117
6095	LOC_Os03g63074	EQIDNKTFASYSSRFSFPSTESGSF	S	295			6096	LOC_Os03g63074	HAFRTMPAVGPRSYPGKIAIVGDLG	S	184
6098	LOC_Os03g63710	YRQILQKEVAKSSADNVLPSSPVAA	S	125			6099	LOC_Os03g63940	FFNGQRCQMCLRSDTLLKVMERLAN	S	450
6101	LOC_Os03g64130	EERDELYGMLTASVRAQLRARLSGT	S	361			6102	LOC_Os03g64210	MATMPSTCASSSSLFLLLRRDRRSS	S	13
6104	LOC_Os03g64400	SKLRSEVEEQLQSVLSKKVEISFEK	S	707			6105	LOC_Os04g01490	LPMNMKKLNNGKSFKRMNSLGGVYR	S	200
6107	LOC_Os04g01620	TAHWVMQFDGSLSLQGAGAEVTLTS	S	1390			6108	LOC_Os04g02000	WMCSNCNNHNYASRAFCNRCKTQKE	S	323
6110	LOC_Os04g02870	SYDRSPVNDRYRSRSPVRRSRSPLA	S	227			6111	LOC_Os04g03450	KPGTFLYVLTKPSVKGANDEDSTVV	S	1197
6113	LOC_Os04g04220	IQCRDKSRRGINSVDDTARKEGVDS	S	135			6114	LOC_Os04g04680	LSKRRPQLEHEPSQPTQKGVHAEYV	S	50
6116	LOC_Os04g06910	CWIIRTMFKELFSLQRDEPNSDSWR	S	538			6117	LOC_Os04g06910	VLRCGNIDTIRSSLRKEPRTKCYFY	S	152
6119	LOC_Os04g07060	LAEFLRIRPPTFSSSNNPVDALDWL	S	372			6120	LOC_Os04g08000	FDKTIVMASSSSSTSTTNPLFSVHI	S	238
6122	LOC_Os04g08310	RGADWEVVTLTASAYAAAPGPGGAG	S	28			6123	LOC_Os04g08440	LTDKTLSEDCEGSERVQRISKRYVL	S	1548
6125	LOC_Os04g08740	KVLAADSALGIASRGKLEAGPVAAI	S	271			6126	LOC_Os04g08740	QDWMLRRANFSGSYVCVKFEIRIRE	S	540
6128	LOC_Os04g11080	FGSVNFNVRPERSDLWPVNATMSKW	S	141			6129	LOC_Os04g11080	GVSNLVLPASAPSLFTELNEFDEEC	S	526
6131	LOC_Os04g11990	LQRQAPSQWLRRSSVITWLHMLPTC	S	86			6132	LOC_Os04g13340	DGERRLLVYGDGSTPQGALQAAGAL	S	76
6134	LOC_Os04g13960	KELYALIPSFPESPFSRKKSLKSSK	S	594			6135	LOC_Os04g13960	PESPFSRKKSLKSSKASKASHTDPK	S	604
6137	LOC_Os04g14640	CKLLKFTSEEGSSAFFLWRSDCSFL	S	97			6138	LOC_Os04g15800	EYHQLTSQDLAESYEDVMEGMRLDF	S	270
6140	LOC_Os04g16360	PVGIHQGRNIQVSYEFYHPMSSARQ	S	153			6141	LOC_Os04g16360	YEGFQKNARIWFSYEDSAHLELPSD	S	101
6143	LOC_Os04g16450	NGGGANMVASGYSTGTALGAEIIGT	S	161			6144	LOC_Os04g17700	AAAAPLRRSLLPSLNPSCLFSSLAS	S	20
6146	LOC_Os04g18090	AIAKYMEEKHGASLPANYKKMLSIQ	S	70			6147	LOC_Os04g18280	IPPNQFGKKGIDSRGFRGGTALGFR	S	278
6149	LOC_Os04g19770	QGIKTRVYDKLMSHDRKWVEELPSV	S	532			6150	LOC_Os04g19990	TVSCHFIGYPETSKGYRFYCPDRVT	S	593
6152	LOC_Os04g21220	PVVFFAPPTSVRSSELHQIGLSEIP	S	805			6153	LOC_Os04g22060	EAADARSRCDHNSSLEVVAISQHLN	S	948
6155	LOC_Os04g22830	RQVSQEYKKHKLSKAQKAARLKAQL	S	262			6156	LOC_Os04g23010	YCPIHKTKNHDLSSCKVFLSAMKAP	S	125
6158	LOC_Os04g23990	REISRIILVFLVSNHFRLRLRTRDI	S	373			6159	LOC_Os04g24170	ARDDLEAFLGDDSAKFVSWLWGYLS	S	89
6161	LOC_Os04g24210	VKRLQSDKLTTKSDKCFFVGYPKET	S	266			6162	LOC_Os04g24410	IEIADRWFEGLLSKDISVTNEIYAI	S	206
6164	LOC_Os04g24520	RGVKEIRFLFCQSSPASAPAREFVK	S	21			6165	LOC_Os04g24700	FDKKRIIEEIFKSITKKDSSQHSSN	S	297
6167	LOC_Os04g24880	STWQGAAPKMKWSEQKIEFSDEDHP	S	537			6168	LOC_Os04g25550	EASNADRENGAESDSEEERRRRKVK	S	1019
6170	LOC_Os04g26660	TLHQHYPDELQHSPYRYHPRRGGAR	S	183			6171	LOC_Os04g26900	KIENFDQNVGALSLELTRDEMAELE	S	194
6173	LOC_Os04g28040	NVGSAQEQRPEQSMSQGAARSPMIR	S	498			6174	LOC_Os04g28210	LFELTSLRYLDLSENSLNANDSELP	S	137
6176	LOC_Os04g28460	ACLQLWSSITPVSPATTWGPLAIIF	S	64			6177	LOC_Os04g29180	WQERLRESVTNLSHAFEEAAAATHQ	S	199
6179	LOC_Os04g30730	LQAQLKDLKAELSLLRVAKVTGGAP	S	29			6180	LOC_Os04g30730	PNKLSKIKVVRTSIARVLTVISQKQ	S	53
6182	LOC_Os04g31180	WEIQKNGHFSVNSMYKAIMYREVVP	S	644			6183	LOC_Os04g31260	FFRFGSFKIRDSSQVRFWEDKWLGY	S	53
6185	LOC_Os04g31340	MLAECVGLPWFESWGTKKLSELGLP	S	251			6186	LOC_Os04g32710	KHKKKRLVQSPNSFFMDVKCQGCFS	S	32
6188	LOC_Os04g32920	VSWAVTLHLAFQSVGIIYGDIGTSP	S	62			6189	LOC_Os04g32950	LLLSALLVQIRASDPLFYEPFDESF	S	23
6191	LOC_Os04g33110	KMRRSWSSAASASSGGGGHGSGPKC	S	47			6192	LOC_Os04g33260	FCLQEKLGMLVSSSKSSPFHPPPAS	S	66
6194	LOC_Os04g33300	PTFGGSSRLAPPSNPKWKRVLLKIG	S	57			6195	LOC_Os04g34140	LTRLLLLLQSGCSPAARQMALELLK	S	416
6197	LOC_Os04g34500	GSSFPARLPVGSSGLPLIRCPRCGA	S	39			6198	LOC_Os04g34500	VNGLDGDYDSFVSALGMRVEPISMT	S	281
6200	LOC_Os04g35080	VGAAFVLLLVLLSLLCLVASRRRRR	S	37			6201	LOC_Os04g35090	LPSEIVPATLKKSARPPGRPFGSGP	S	94
6203	LOC_Os04g35130	YKLDTQIPGTRSSSSPRPHASPPKT	S	96			6204	LOC_Os04g35170	GRDKGRSRGNSDSRSYDDEDDDGPP	S	185
6206	LOC_Os04g35330	QARGWLMNLPPASVHSWEDLCQQFT	S	295			6207	LOC_Os04g35700	GQFCAIKEVQVISDDPHSKERLKQL	S	339
6209	LOC_Os04g35800	SPRRGRSRSRSRSRSRGRSASPRYP	S	82			6210	LOC_Os04g35910	DHLYGDKDKFVTSAYRKKLEEERKW	S	143
6212	LOC_Os04g35930	YLTSDDGGVVVISAPRLSSLRLGIY	S	249			6213	LOC_Os04g35950	DAIKAPGMVLRLSQPIIGVTPGHTW	S	599
6215	LOC_Os04g37520	KFIVSCLLLVTASQFSALLAATRPH	S	260			6216	LOC_Os04g37520	LPRRAYDGQVQASLTLSAALAYLTL	S	94
6218	LOC_Os04g37950	SYRVVFPMELKLSTSDDVDTEYSLF	S	285			6219	LOC_Os04g37960	HPANIGELFSEGSLNPYAEDPYAFG	S	875
6221	LOC_Os04g38260	IDGEEIARNQRASEVSDNDGYEEFN	S	117			6222	LOC_Os04g38290	EEIRLFDGAISESRDLRRYVIAVME	S	415
6224	LOC_Os04g38480	GELQFQTEVEMISMAVHRNLLRLRG	S	356			6225	LOC_Os04g38620	SFFTWFNSEKNRSFAHGAMDEVADV	S	194
6227	LOC_Os04g38630	VTHLADELRKHESVMAAADLTAPHA	S	182			6228	LOC_Os04g38910	LLALSAGANDIASDAAALQAFIAPF	S	27
6230	LOC_Os04g39040	DEPQEYYTGGEKSGMLVQDPTRRNT	S	61			6231	LOC_Os04g39090	LRTQPYAGKSTISIDTTQPANGVAS	S	257
6233	LOC_Os04g39240	DQRSPVENSDSLSDPVDRALSSTET	S	405			6234	LOC_Os04g39240	KIPVDQRSPVENSDSLSDPVDRALS	S	401
6236	LOC_Os04g39629	SQQSGWPRGSRGSTFPSRLTRTAYP	S	607			6237	LOC_Os04g39740	REGRACRPLTPRSSTQKPSAPRVAS	S	1110
6239	LOC_Os04g39780	AVAFKNSKTYRSSSLTDGESEVVEL	S	256			6240	LOC_Os04g40080	LRILNVGSNRLRSLPDSISKCRSLI	S	222
6242	LOC_Os04g40430	LNKLEKLEEKMASGEMAEGEAEQSN	S	138			6243	LOC_Os04g40430	PLNAFKPHPEHKSKLICNITGDIIN	S	98
6245	LOC_Os04g40660	TGDVELAASELRSLGSDQFHSYFIK	S	112			6246	LOC_Os04g40740	HLTKSLTLNKAGSGNSSDILSDSTT	S	413
6248	LOC_Os04g41030	LANCRFLNSLKLSGNSFSGQIPDSL	S	158			6249	LOC_Os04g41100	GRPHRARENGVSSSSRDGESVVAAS	S	117
6251	LOC_Os04g41160	VSREAARHKKNGSGGEDGHRRIWFE	S	70			6252	LOC_Os04g41820	SPAYPRLASSAASASAPARLRSRIT	S	85
6254	LOC_Os04g41840	TTTPTFQRGSEWSILFTSRGASGSA	S	1374			6255	LOC_Os04g42010	LRVTEDGKKVGRSIELSKLDEIMEQ	S	106
6257	LOC_Os04g42030	QWTLSAEVAIQASEEQRLDKWNLMG	S	288			6258	LOC_Os04g42140	LRFMVRDVVDLRSNNWVPRREEIKA	S	433
6260	LOC_Os04g42320	GSKHKDSKDEAKSSGSNPKGASTPK	S	788			6261	LOC_Os04g42320	KNVGKGKVSGLESKKADAVSDSGGR	S	478
6263	LOC_Os04g42840	ALSLVAEMLNNQSGAMEESIEIVLE	S	1287			6264	LOC_Os04g42840	SSLDAEGGKWMNSVQESTPRNAPMA	S	1087
6266	LOC_Os04g44320	VAFGGYVAVAAVSIGTLPQIFPQLK	S	434			6267	LOC_Os04g44480	GVSHQHGRGDQPSVPPVGGVGCRAF	S	206
6269	LOC_Os04g44640	GKVAGGGTLVVESEVVVKDDLMEPS	S	515			6270	LOC_Os04g44640	SRFSTKYFSTVLSSLSPDQKRIIGD	S	780
6272	LOC_Os04g44800	SESESESSYSDCSAGSESETDSEEE	S	118			6273	LOC_Os04g44870	ATELIIKLMDSDSGTDDDFVGEATI	S	76
6275	LOC_Os04g45180	AIALSLAESLENSVPAMSGETPSTG	S	162			6276	LOC_Os04g45800	AKRLSLGRSNIVSEPEEVLHPQPHH	S	513
6278	LOC_Os04g45930	VENFDDLLLVEDSNNPGMNRGYALL	S	307			6279	LOC_Os04g46180	SMGVVTAMNTMSSCTSTYKSEAVSS	S	211
6281	LOC_Os04g46892	KLNVAEAHAVCLSSQGVILVWNESK	S	249			6282	LOC_Os04g46894	LLSENDFLRNDDSDTKAASGEAKSQ	S	721
6284	LOC_Os04g47250	YMAWFWRMSRGLSGPRVWPVVGSLP	S	28			6285	LOC_Os04g47320	PTSMNASRELRVSFQDPHKRFSPST	S	177
6287	LOC_Os04g47380	SRPLTDDDLHNISLIKDMEIVQKTP	S	286			6288	LOC_Os04g47700	DMSSSILICGDRSGTVRIITFRKDS	S	432
6290	LOC_Os04g47860	QQHQHQQQQLAASLTGVATAAPTAA	S	45			6291	LOC_Os04g48160	KTPGSERLTRERSESCGRNWLDKWV	S	240
6293	LOC_Os04g48770	KRVNQETLAPASSGASNSTRQKSGK	S	265			6294	LOC_Os04g49010	GTSTTSEPEKDPSAAKPYISDKNHE	S	127
6296	LOC_Os04g49194	ASFMCPCNSAVISPARKLVADGDAP	S	297			6297	LOC_Os04g49210	GPDRRKVVEAIGSACETDGFFMVKN	S	67
6299	LOC_Os04g49300	GTSPALPAMSSPSRQGAPVYVAFVA	S	266			6300	LOC_Os04g49450	RNGNRNEDEMVPSPDSRKCVSPGSN	S	414
6302	LOC_Os04g49510	DVRREIQIMHHLSGHKNVVAIKGAY	S	144			6303	LOC_Os04g49520	LPNAFTFCCVMKSCGALGCRELAPQ	S	147
6305	LOC_Os04g49950	PRENLGTGGGGESSLVSATDAGGGG	S	338			6306	LOC_Os04g49954	SPREFTQEAARQSLIAISRSVPAAG	S	18

6308	LOC_Os04g50830	DVQEVRIGRFSISAQITDIRSGVTF	S	96			6309	LOC_Os04g50990	FVRVTGGEVGAASSLAPKIGPLGLS	S	26
6311	LOC_Os04g51060	LKRPMGKTDGNISLDRSNSSLKGSL	S	443			6312	LOC_Os04g51080	LPERAVALALAVSLDCDYFSKRGGW	S	315
6314	LOC_Os04g51090	GRKILDGPNVMSSVYNKDYTNLPFG	S	598			6315	LOC_Os04g51120	PPPSSGTSEANTSTSKNTTPEPFDP	S	364
6317	LOC_Os04g51370	GDSGRSPEASLPSPSSSGNGVATRI	S	38			6318	LOC_Os04g51390	KNDRGDELLFVSSKATFKPPKAIRG	S	67
6320	LOC_Os04g51710	FVLGLIAGILILSAVHNAILLIVVL	S	238			6321	LOC_Os04g51830	PPYTTWFPFEENSTTKDSNAENQGI	S	376
6323	LOC_Os04g52120	LSLELVFFSSSLSSVGDGGWALIIF	S	571			6324	LOC_Os04g52180	MDVEMADEGNNKSKSFLKPIGSISK	S	82
6326	LOC_Os04g52540	TLPPPSLKLGTSSGQPPKFNIDQPN	S	582			6327	LOC_Os04g52700	PPPSSAAAAASTSAAVCTLDMVRSA	S	107
6329	LOC_Os04g52820	FSDQRYLPMHMLSHEIAEPEPMVLS	S	266			6330	LOC_Os04g52920	LVCDVLEEAVSGSSDGSKCSSAINK	S	68
6332	LOC_Os04g53040	IMSEDRYEGVNESLGMEEGDESKRT	S	463			6333	LOC_Os04g53040	KCWSPSERRTFVSMDVTFRESIPFY	S	422
6335	LOC_Os04g53060	TLRIGRHPNDIFSFDSDDILELMIL	S	98			6336	LOC_Os04g53090	DFPPRDGRPLADSLHRYSRKGLGLS	S	902
6338	LOC_Os04g53670	LHVENLYFCESPSEKCLLLNLDKDP	S	537			6339	LOC_Os04g53670	PLFSLYCLQLTPSPLPPKFASKTVI	S	434
6341	LOC_Os04g53700	QSCADSDWETYHSLLCTGSKTEPSQ	S	246			6342	LOC_Os04g54340	LLGGDLFHENKPSRSTLVKTIEILR	S	63
6344	LOC_Os04g54370	PAEEHHSYDNSASESNQDESTGAST	S	209			6345	LOC_Os04g54370	VDTVVVDKYANSSDSTPATTEEPHP	S	240
6347	LOC_Os04g54440	KGFALFVYKSVESARRALEEPTRNF	S	283			6348	LOC_Os04g54670	RPRGWSHKRGAESSPESRGGSSSTP	S	1315
6350	LOC_Os04g54680	NEDQDQGPWKKISKGHLKKTTSKDQ	S	368			6351	LOC_Os04g54940	MDSAEKERDFYFSKLRDIEILCQRP	S	214
6353	LOC_Os04g55040	LQSVRSASLRVRSHQENVAGVKLPK	S	98			6354	LOC_Os04g55520	AYDTAVFYLRGRSARLNFPEEISSL	S	112
6356	LOC_Os04g55810	GDREEDKGGGSESGSGSDEDDSDGS	S	141			6357	LOC_Os04g55960	VAPRLQPRYYSISSSPKMAPSRIHV	S	478
6359	LOC_Os04g56160	RTLHGLQPPEVASNTLFNDKSSYRE	S	892			6360	LOC_Os04g56350	KSSDRVMVAKVKSSVFERFLESGKQ	S	218
6362	LOC_Os04g56440	LRVRSFSANGRASVMPFSYIRPHGD	S	39			6363	LOC_Os04g56530	GKYVEDESKDGSSLEDGDAFSFQAG	S	438
6365	LOC_Os04g56640	FGATYTTDVVLPSLEDQGVRQLYPK	S	53			6366	LOC_Os04g56720	RVEVQVAKKASASTNARSNKRKKTK	S	450
6368	LOC_Os04g56740	TPRSQDNVSPKQSNDTSIPESSVGH	S	592			6369	LOC_Os04g57140	VQRLERAKSEEKSNMERVYEDECCK	S	709
6371	LOC_Os04g57520	LLINLQSTEEFSSHMLNRDTWANEA	S	206			6372	LOC_Os04g57600	SYYEPIEGQDYKSDDENAYEDDDEE	S	683
6374	LOC_Os04g58140	CLLKAIDTLPPPSRDVSKPLRLPIC	S	476			6375	LOC_Os04g58140	DAAILVIDASIGSFEAGMGINGIGQ	S	368
6377	LOC_Os04g58620	GSVVDNTEDEANSSGLDEEKGDDDA	S	105			6378	LOC_Os04g58720	ELGLKVYITNDPSIKASNPLPAMDP	S	135
6380	LOC_Os04g58900	FRTTATRRRLLPSSALRLTRPLSSS	S	31			6381	LOC_Os04g58900	SSALRLTRPLSSSCSASPLAVVASM	S	43
6383	LOC_Os04g59040	RRAPKLNERILSSLSRRSVAAHPWH	S	26			6384	LOC_Os04g59060	SALRWHPDKHPGSSQAVAEEKFKLC	S	248
6386	LOC_Os04g59330	SRDSHDGEKTATSVHSTPALSPMGS	S	48			6387	LOC_Os04g59460	AAVRSAAARLLLSCYSAWTPQYPHA	S	122
6389	LOC_Os04g59494	REFLPRLIPTLLSNMSYSDDDESLA	S	331			6390	LOC_Os04g59570	TRTRGPPARPQQSYTDDDEEEDDQD	S	82
6392	LOC_Os05g01230	VVIKEEDLQPKKSKGEDEKEQGDKK	S	655			6393	LOC_Os05g01400	LQMKMPGPGGPISVHGNLKVALACM	S	650
6395	LOC_Os05g01540	VRRGRSSSSYSDSPTPPRRGPRRVL	S	384			6396	LOC_Os05g01610	NREKVYERYNVRSSERVSSSSAAST	S	901
6398	LOC_Os05g01990	SHKVVVFLSTCDSVDFHHTVLSQLE	S	335			6399	LOC_Os05g02110	RAVLAHQAEPDLSHVVLPRGSHHDG	S	379
6401	LOC_Os05g02240	VGTFAVAMACGMSAREALVLGVVMN	S	375			6402	LOC_Os05g02260	SADGGKSLPEKHSALADATSGVAAE	S	36
6404	LOC_Os05g02500	PPFLSPSSLSSDSSISSKYSSDSPS	S	513			6405	LOC_Os05g02500	TSLRREEEVTDKSNNNSVKQLTGVC	S	621
6407	LOC_Os05g02650	SYITGPDATKILSLCPSLQKAIKSS	S	359			6408	LOC_Os05g02670	ELKELYDAEQLLSAELSEKLGKTQK	S	483
6410	LOC_Os05g03060	NLETELFDNVRASIQRSHGNPGKAP	S	161			6411	LOC_Os05g03120	TPERSPKVTERRSPRSPITEKRPSR	S	58
6413	LOC_Os05g03190	VPEAVPVPAQTSSLPPGVSREEQAT	S	98			6414	LOC_Os05g03320	DDHGFAPTGKGISIGDLECRPYDCN	S	411
6416	LOC_Os05g03430	CLGGGDTAENADSDSDLEVVAESVT	S	348			6417	LOC_Os05g03440	GGGGRSRRAPATSPMFVSRGRGGAG	S	45
6419	LOC_Os05g03460	AKLGPFEGDKHVSTRVMGTFGYCAP	S	250			6420	LOC_Os05g03550	HKRTDQSNRSSQSGSQRQRSSNRSR	S	640
6422	LOC_Os05g03630	VKGEGMPITKEPSKKGDLKIRFQIK	S	290			6423	LOC_Os05g03780	APPQIAEVRMDISSSTSVAAGNKVC	S	19
6425	LOC_Os05g03820	ALQPIATAIFANSPFKEGKPNGYLS	S	256			6426	LOC_Os05g04340	RSSLGFQNETSTSGDADRLPNEMSN	S	20
6428	LOC_Os05g05230	AKVIQAGLFNTTSTAQDRRALLQEI	S	905			6429	LOC_Os05g05230	FNAGHRLDSTVHSIEEKVTEQPSAL	S	424
6431	LOC_Os05g05300	RSRNYTSRGSRSSIEKPRNSATASG	S	474			6432	LOC_Os05g05370	FLCSSLLVFKGHSLVMNLSHSLLLL	S	88
6434	LOC_Os05g05480	DILKEHAIPSVRSSDLSEPYVDTEQ	S	557			6435	LOC_Os05g05590	PRPDIAADLSSQSIIDPLLGSLLGS	S	460
6437	LOC_Os05g05950	EGSASVSPVSGLSSSREYTNSMDGQ	S	477			6438	LOC_Os05g06280	AVGKISFIDLAGSERGADTTDNDRQ	S	436
6440	LOC_Os05g06630	DIAEHDDSPKSSSGRLTRSRAKAIE	S	44			6441	LOC_Os05g06760	CFDGNCPKEDPLSLPGFRKLAEPLP	S	325
6443	LOC_Os05g07130	VDRGVGSTKFGKSSSACSLASGLNF	S	328			6444	LOC_Os05g07220	CLNNKKGSLAAVSLDGKIYAIGGGD	S	871
6446	LOC_Os05g07350	QEKFLAGMDPELSVRLVSGDYPDFQ	S	522			6447	LOC_Os05g07680	HDSLQNHSLEKSSLSAELSKLSEAN	S	446
6449	LOC_Os05g07700	CYRQIKNKPYPKSRYCRGVPDPKIR	S	20			6450	LOC_Os05g07980	YAESEISTESSLSISEADKSPSHSS	S	67
6452	LOC_Os05g08600	RPSHDRDRDRDRSSRDYDRSSRDRD	S	293			6453	LOC_Os05g08720	LMMVKNIFLSCGSLKVHNGTQVRFW	S	354
6455	LOC_Os05g08790	YLRTELAETSVTSNTSQPEQDVNDP	S	703			6456	LOC_Os05g08840	IEIRYKEWAERSSSINQLVHCINGF	S	696
6458	LOC_Os05g08970	VLAEKYKDRLENSYQGLIHEVFSKV	S	314			6459	LOC_Os05g09630	KFQKYLVSGNPRSSVDHPASADLKA	S	216
6461	LOC_Os05g10540	PHTAHWVMQFDGSLSLQGAGAGVTL	S	1386			6462	LOC_Os05g10730	NGADVYLLDDPFSAVDAHTAATLFN	S	784
6464	LOC_Os05g10810	SGDEAASGEPSSSSSSSPSRRRGGD	S	78			6465	LOC_Os05g11710	VQKLVLNISVGESGDRLTRASKVLE	S	28
6467	LOC_Os05g11770	KGSHSGLKDIIQSYLWEEWKERHPE	S	441			6468	LOC_Os05g11780	WKTVKPFVNGGASGMLATCVIQPID	S	32
6470	LOC_Os05g13510	LKLHTFFNEIGISHHVSCPHTHQQN	S	556			6471	LOC_Os05g13790	DSSRASKRRVADSRKSRSSEGSKLP	S	289
6473	LOC_Os05g14110	HSWLMHLPPYSISSWVDLWQQFITN	S	386			6474	LOC_Os05g14750	DDGHIMLSDFDLSLRCTVCPTLVKS	S	312
6476	LOC_Os05g17000	FVVVFIDDILIYSQTEEDHQHHLRL	S	870			6477	LOC_Os05g17750	SRQMKTTRTAEESIKKNSNHMMVMH	S	32
6479	LOC_Os05g18770	SEAFRKAKSFMPSRNSLLEQAKKML	S	307			6480	LOC_Os05g18774	RRSPPGDAGEGTSGGGGCCSSSSHG	S	106
6482	LOC_Os05g19100	SAVLHLLEDYGSSSTSTPREVLAID	S	55			6483	LOC_Os05g19370	PVHKHRELRGLTSAGKKYRGLRGKG	S	166
6485	LOC_Os05g20230	EAHESPYSIHPGSTKMYLDLKEKYW	S	1316			6486	LOC_Os05g22670	DGSADQIVEFVVSKDNAQQLWSSIV	S	238
6488	LOC_Os05g22920	HCNKKPFYLKGGSTVDSSTMDSYLM	S	214			6489	LOC_Os05g22940	VKSGGGIHEFADSQFGHVFAYGTTR	S	665
6491	LOC_Os05g23320	MKQLIGGVIMDLSSIINMQNDDGRT	S	341			6492	LOC_Os05g23740	IIPRNTTLPTSKSEVFSTAADGQTS	S	466
6494	LOC_Os05g24160	VLMQPTVITQEASCGTLFLQPCEEA	S	276			6495	LOC_Os05g24430	HRDGLLHSFDFKSFETCESCLLGKM	S	427
6497	LOC_Os05g24460	ITGDEVPEDALVSFLRANADVFAWR	S	791			6498	LOC_Os05g25750	DNSVNTGPIQGVSTSTMLVWTQVGE	S	19
6500	LOC_Os05g28280	EVREEKELDLSSSDVVTKYKDAADI	S	18			6501	LOC_Os05g28680	HYFQAHRVTVVSSCPLGEVVRNKDV	S	1151
6503	LOC_Os05g28860	LCQRYSLEGYVNSLGNHKHQRYLSY	S	333			6504	LOC_Os05g29010	EDVNATRWEQLKSTLQSGKHKGLRR	S	117
6506	LOC_Os05g30050	RRARRAAAVARHSAATPSSAPPTQE	S	156			6507	LOC_Os05g30240	SSSSTASPAAAASATHHLLPPHAPL	S	37
6509	LOC_Os05g30340	VKRESGAHSEDESVAPTVSVKKEAQ	S	396			6510	LOC_Os05g30510	MYRSSGDGASADSATGGNAGGGGER	S	109
6512	LOC_Os05g31000	IELVMTQATVSRSKAVKALKAANGD	S	183			6513	LOC_Os05g31490	FLSPSFSPSDFSSAALSSGLAASRA	S	66
6515	LOC_Os05g31920	SLRSQVENLTRKSQLLEADLERTTK	S	883			6516	LOC_Os05g32070	NATTPRSFDTSSSHQVASFRDALPR	S	210
6518	LOC_Os05g32570	KSSIGSRPTYDWSVGKISIAVVHSI	S	459			6519	LOC_Os05g32570	MGVAFCFRSKPFSCSVADVEKAQPF	S	363
6521	LOC_Os05g32610	LSKLVFSTEDEFSNVRNMLSKAEME	S	1384			6522	LOC_Os05g32760	TVLNLKSVDISDSHIETSDHHHSHT	S	565
6524	LOC_Os05g32960	ILLFRSFEGLKFSSLRFKYHAEMRR	S	249			6525	LOC_Os05g33030	SRSHSLVKVTVRSEHLVTGQKWRSH	S	390
6527	LOC_Os05g33310	RHVVCPSSNRRVSITFVRVRPSTPV	S	228			6528	LOC_Os05g33310	SADMARHVVCPSSNRRVSITFVRVR	S	223
6530	LOC_Os05g33570	ARLGDPARPLLLSVRSGAAVSMPGM	S	166			6531	LOC_Os05g34090	PEATVGRPPLSSSDPEVVGTEDECA	S	1234

6533	LOC_Os05g34540	LYAESLARFQGGSPYIYPLYGLGEL	S	222			6534	LOC_Os05g34620	MTARQRLGAGGRSSATKTSGAATTG	S	126
6536	LOC_Os05g34790	NRKCEQFAYGMSSELDVFIQAEFEE	S	19			6537	LOC_Os05g34790	VFIQAEFEEKARSMHADIMSHWINK	S	35
6539	LOC_Os05g36090	VIGRSASDVIRRSASGHEAAPPFQT	S	414			6540	LOC_Os05g36160	MASSRVMAAAAASSSSSPPPPPPAA	S	13
6542	LOC_Os05g36260	AVFNVVVEITKGSKVKYELDKKTGL	S	71			6543	LOC_Os05g36350	LRVHGHVVRLRISQSSYLATQIVHL	S	43
6545	LOC_Os05g37330	SPSADDIKNILESVGVEANDERLEF	S	29			6546	LOC_Os05g37480	LVNERKHLALAMSTSGAPPYGISGF	S	162
6548	LOC_Os05g37930	STLCGLPKSGQESPNGGASQVSSPP	S	89			6549	LOC_Os05g38150	VARLTIKPGKIASLAKSIRTLANME	S	368
6551	LOC_Os05g38710	FIINPKGEVAINSSVDVKSYTSLHT	S	1030			6552	LOC_Os05g38710	RISRSLQRSNSDSSEDIFLDTETVL	S	791
6554	LOC_Os05g38730	TVPNPAGDPVGNSNSIVNTTSSYSY	S	158			6555	LOC_Os05g38810	GPLKESEIEVHASSNTVKVVFKKRA	S	800
6557	LOC_Os05g38950	EQIMSVRVEEKQSPASGNQDTKDVI	S	120			6558	LOC_Os05g39090	KASRHYSRGRFNSLSIRLIKDGSVK	S	531
6560	LOC_Os05g39610	LPDLDPVFADPYSAALRAEVTAVCN	S	389			6561	LOC_Os05g39610	RYVMNYLRAACGSRQTLEEVMEGDF	S	449
6563	LOC_Os05g39730	TTVDNDTGAPGASTLPTQGVPRQGK	S	31			6564	LOC_Os05g39840	KSTKAMSIGQILSMKLDHRELEVAG	S	468
6566	LOC_Os05g40080	CSGDDAQNNSETSHESSPPEAPAGE	S	245			6567	LOC_Os05g40200	IHGNIKSSNILLSQGLSACISEFGL	S	479
6569	LOC_Os05g40420	SVGFVETLENDLSQLRAKGIDACIA	S	189			6570	LOC_Os05g40960	AKIKISELVSQVSNECLSNAVTEIR	S	218
6572	LOC_Os05g41070	ERRQKRMIKNRESAARSRARKQAYT	S	276			6573	LOC_Os05g41070	PLLSMNLDELLKSVFPDGADLDGGG	S	67
6575	LOC_Os05g41210	LNLMAKKMKDTDSEEELKEAFRVFD	S	82			6576	LOC_Os05g41240	KFSVPFGPQSTQSNEHMSNIGAFGG	S	19
6578	LOC_Os05g41500	SFSGVETTPKFGSFNPADDLLVAFQ	S	105			6579	LOC_Os05g41510	NFGSDDGGGGGWSGGGGGGGNSGGW	S	1530
6581	LOC_Os05g41730	SSSSFSHWRRSLSSSRRRVTPHGAA	S	146			6582	LOC_Os05g41770	PRRKVQRPVYFVSEALRDAKTRYPQ	S	619
6584	LOC_Os05g41800	PSPSSSPYSVSSSKVSPLLLLLSAR	S	42			6585	LOC_Os05g41800	RAGDRGAWRGVSSCGATRAVDVGDE	S	71
6587	LOC_Os05g42220	VSTYCLVNSEEQSFLPEPQPLLSRS	S	174			6588	LOC_Os05g42230	WGSIPVSVDDDDSAPDAAQHANAHA	S	116
6590	LOC_Os05g43060	ELLKFSSTPTITSSTDDLDWVSSMY	S	388			6591	LOC_Os05g43280	RLPQVDKAASLSSELPQTSTTSNSE	S	868
6593	LOC_Os05g43380	SKRQSNDRVIDDSGEYKNSNHDWQL	S	299			6594	LOC_Os05g43520	PDSDEAEQPDTPSRRGVKDDISELT	S	56
6596	LOC_Os05g43530	LAVASAARRRVASGRTDADAAPGES	S	96			6597	LOC_Os05g43530	VIRDGYKAGNLKSAMNCSYVKDYEF	S	311
6599	LOC_Os05g43670	GSDADESSETSSSAEAEARKRSNSK	S	371			6600	LOC_Os05g43730	VDGGACAHVSRRSLKAMKRELLAAA	S	550
6602	LOC_Os05g44070	SKSTIGVEFATRSLQVDGKVIKAQI	S	58			6603	LOC_Os05g44270	AQKGFFLHPSPRSAEPPRLLPLFPV	S	233
6605	LOC_Os05g44320	IFTWNLVEGSDDSPPPSPGTPEQEA	S	227			6606	LOC_Os05g44320	RALLLSLRLNEDSLIKKCIFSVDPS	S	781
6608	LOC_Os05g44590	LRDTDREGVASTSGQFTFNTSLASL	S	64			6609	LOC_Os05g44590	SRATGLALDGNPSTVHSWFPGYSWT	S	500
6611	LOC_Os05g45060	VIFVADDSDSPGSSSRGGAGGGAAS	S	89			6612	LOC_Os05g45400	GDLWRLESSRGGSNSGDENAPLFLV	S	256
6614	LOC_Os05g45670	LFSAILGDLTSRSISYVMDRYCSNQ	S	15			6615	LOC_Os05g45740	ADPPTIERCQELSKQLFYTRLASLP	S	54
6617	LOC_Os05g45830	HPSFHSSTSSSTSQFPIATNQVTGN	S	467			6618	LOC_Os05g45930	DLEKQNTVVSDASNDPERLREEQAA	S	102
6620	LOC_Os05g46430	FRKMAKAVKNQVSDNYYRPDLTKPA	S	107			6621	LOC_Os05g46430	RPDLTKPALARLSSVYRSLQVSKSG	S	124
6623	LOC_Os05g46500	GGGGGAAARVERSRSAGHGGYRAAG	S	47			6624	LOC_Os05g46560	VPVADTPLEPADSVFDISGGKRAFI	S	124
6626	LOC_Os05g46780	DSARERRADRDESNGESGRSSRRGR	S	258			6627	LOC_Os05g46780	ERRADRDESNGESGRSSRRGRSVSP	S	262
6629	LOC_Os05g47470	VKRVKELLAAEKSNHEAERRRAADL	S	123			6630	LOC_Os05g48070	VYPPQDLSEMKSSVKRLYGGANAMR	S	312
6632	LOC_Os05g48260	PLWELHVLDFPTSEAAATVAVRMHH	S	212			6633	LOC_Os05g48290	GGDIASTFDNPESVKLGHCKVIEEI	S	334
6635	LOC_Os05g49150	HHHPGFRGNQSSSAKSSVDIQPDWT	S	160			6636	LOC_Os05g49150	VDDSLAAAAEDSSFRLVDAKPPPRH	S	89
6638	LOC_Os05g49180	AQDSIHPKDHNHSAAVNKQAYDNDW	S	251			6639	LOC_Os05g49230	GGAWAQEATAQSSLVIDAVRLATMF	S	136
6641	LOC_Os05g50480	PRAGDAAAIPPASRKLVQGLKGILT	S	40			6642	LOC_Os05g50530	LKFLAIADTRFASTIVMLKRFRAIK	S	438
6644	LOC_Os05g50560	IPMERKHASLPRSTTVHSTPIPPKE	S	145			6645	LOC_Os05g50810	LKRLYGAEADIWSAGVILYILLSGV	S	298
6647	LOC_Os05g51140	VLKQMYHAPVTASYHQSAWAKIGRI	S	98			6648	LOC_Os05g51220	SFPSQIARSFGRSFSYCLVDRTSSV	S	274
6650	LOC_Os05g51630	TNDTLDSMKVVHSKIDKLSISNIPY	S	138			6651	LOC_Os05g51630	VLVRNIPPDPDESISELVEHFFLVN	S	212
6653	LOC_Os05g51830	RFLHLSQAALGESKKGSDNAVMYVK	S	34			6654	LOC_Os05g51850	ATPLVKPGPGRPSGFKRGPGRPRKN	S	275
6656	LOC_Os06g01320	LDDFVRQRFGFESYERVERGLLVPK	S	851			6657	LOC_Os06g01400	SVFEKDEERALWSAYLEVADKIHPG	S	996
6659	LOC_Os06g01680	EKAVDDLNYRLQSLVPGDSEHVQVL	S	803			6660	LOC_Os06g01700	DELEERERKHYSSKDKSYAEERDRR	S	73
6662	LOC_Os06g02160	LSSGLPTQVLIPSRDGRLYSFDTTS	S	1035			6663	LOC_Os06g02170	AADADDDDDDELSRLLSLAEADLEA	S	20
6665	LOC_Os06g03780	MKLQLYKKLLATSAVDPENIQEKVK	S	425			6666	LOC_Os06g03860	EGVNLSLLSSVMSSRLSRGTYNGGL	S	631
6668	LOC_Os06g04280	RARGRREAVVVASASSSSVAAPAAK	S	60			6669	LOC_Os06g04690	CLKVPCLLQRLISLKVFGCDKLKLI	S	270
6671	LOC_Os06g04940	AVATIASAVPTVSSLSSFSNASSFL	S	29			6672	LOC_Os06g04940	SSFLCQLILYQKSRFSNLAAYACVC	S	50
6674	LOC_Os06g05000	SFEGAPEHLKNTSFQGTGRPHPAFF	S	101			6675	LOC_Os06g05000	YLDQKLALAKRCSREATLAGAKAAA	S	20
6677	LOC_Os06g05020	VATIASAVPTLASVRMLPWAKANIN	S	45			6678	LOC_Os06g05190	KSSKRNLPSWMGSKDGEENPGKKKH	S	26
6680	LOC_Os06g05530	PRHPPSEGSYYASRMAALHGTSDIL	S	35			6681	LOC_Os06g05870	HSAAASPAGNVVSVTEADLESSEVT	S	223
6683	LOC_Os06g06530	GDNPAKVKEFVKSYNALHEMGFTSS	S	195			6684	LOC_Os06g06770	GRPPMGRSEAMESCPRKGVVNSKEK	S	131
6686	LOC_Os06g07160	EADSDSSATGLGSTDPSSSSTPRKR	S	261			6687	LOC_Os06g07200	NSNEESKIVTKASAMKEIKKRMEKD	S	77
6689	LOC_Os06g07820	EHTSEVDENVPGSVHHVNEIESDSA	S	227			6690	LOC_Os06g07840	KVTQKSELGKASSVHAIDKHQHRAI	S	43
6692	LOC_Os06g08280	LEDAVNNQSDKTSQGVKVLDDIDNL	S	976			6693	LOC_Os06g08480	KKSSPSNEDKKQSRIKFDVLLTSYE	S	294
6695	LOC_Os06g08840	GRGGRDRYGSSESKCYECGETGHFA	S	87			6696	LOC_Os06g08840	SPAGRSPRRRSVSPARARSYSRSPQ	S	153
6698	LOC_Os06g09450	FVLELDFEPFNASFPRPSMSKSIGN	S	140			6699	LOC_Os06g09570	IAQQAALFHSIKSLFMNKPLVIVCN	S	277
6701	LOC_Os06g09930	LCYLLFRELRKFSFKLVYFLAVSDM	S	53			6702	LOC_Os06g09930	VLDAVGTSAAALSLAGSSFIVLCYL	S	32
6704	LOC_Os06g10880	DGSALARQGSIYSLTFDEFQSALGS	S	18			6705	LOC_Os06g11380	FLEKSHTSRSRSSSRGSSPGRSPVH	S	107
6707	LOC_Os06g11970	ASWVSEGMPPTTSSVASIFAGTSDS	S	229			6708	LOC_Os06g12080	SPLSPLHRLFSSSTAAASIAAEPFA	S	33
6710	LOC_Os06g12400	SDSSDDEEWYGNSTPEKGNLEDSET	S	546			6711	LOC_Os06g12400	TAENNNNTNTVDSIQLRGSNDIVSV	S	692
6713	LOC_Os06g12590	GLGTYHPSDSRNSTGSSHMYEPSAP	S	637			6714	LOC_Os06g12610	SLQSSRNPTPRGSSFNHTDFYSMVG	S	254
6716	LOC_Os06g13720	YGMGTAEWRASKSPAYYKRGDYVPG	S	248			6717	LOC_Os06g13810	ALLQCGKTGLITSVGNLAAPVEEWT	S	476
6719	LOC_Os06g14080	GNADEGTIENTNSNTDESNKETANN	S	481			6720	LOC_Os06g14160	EDREGSERVHRISKRYVLVEGTLYR	S	1218
6722	LOC_Os06g14412	GIGTADIAGTDMSTGEKIGIVKEID	S	485			6723	LOC_Os06g14490	VDLSEVEIQEGSSPTPPSDTHSEAT	S	400
6725	LOC_Os06g15750	LPSIECTEINKSSHLGLSIHVDSFV	S	875			6726	LOC_Os06g16250	AAKSHDYVVLSASHWFARPAVYYQH	S	310
6728	LOC_Os06g16390	DESICRQEAHGRSWKVIEQGLLLKG	S	535			6729	LOC_Os06g16390	SDTRSGIKDSVVSSQCNSPSTRSFR	S	467
6731	LOC_Os06g16430	RKSDQSAANEVESSVESEASAADAV	S	677			6732	LOC_Os06g16980	EDGSVAHSMFHRSEKRKSTLAACGT	S	147
6734	LOC_Os06g17820	EKALPLFKLLKRSGPFTWTEEAENA	S	1089			6735	LOC_Os06g17840	DTFSGLNDVNGKSFGRRVAILETVA	S	119
6737	LOC_Os06g18130	WHYDQKGVFSVKSAYKVAIQNKDAR	S	56			6738	LOC_Os06g18830	KSYFAMKVMDKASLASRKKLLRAQT	S	230
6740	LOC_Os06g19090	EFTDLPSITYNASRFDQKTLINLCT	S	552			6741	LOC_Os06g19650	GKHGSTEENFDVSPEEMRSVIEIIA	S	73
6743	LOC_Os06g19660	VLNHQRSLPASSSSEATNIRLIMHV	S	1313			6744	LOC_Os06g19980	AEASGLKVEQHCSEHVQGDMSKCGS	S	108
6746	LOC_Os06g20080	MDVEMVDEGNNKSMSFLKPIGSISK	S	13			6747	LOC_Os06g20090	DGKRLFLSTSGGSSSVGLSTGLQRP	S	359
6749	LOC_Os06g22070	IQMVVTVEKPEASILEFECHFNDDE	S	179			6750	LOC_Os06g22650	VKSIKKRIGHKNSKIQLLALTLLET	S	53
6752	LOC_Os06g22810	NLEYVLQLQDADSSTVSDVNSMNRI	S	796			6753	LOC_Os06g22820	DVDKMSLQDGNASCLATCSTADIDD	S	255
6755	LOC_Os06g23460	KSAVASLTMRAGSKLAQISEMSGAT	S	605			6756	LOC_Os06g23460	QEDVGVDIRLTDSLDGSDERIIIIT	S	377

6758	LOC_Os06g24460	TDNGVLLRSRASSRGEEDDDDEEAL	S	17			6759	LOC_Os06g24540	AEGTSDAKNTEESVAPASANLVSMP	S	763
6761	LOC_Os06g25710	DVDVTVFRRDDSSVAFKGVLKGDLY	S	489			6762	LOC_Os06g25710	KNGENGNQDGASSKGTSPPKGATIP	S	19
6764	LOC_Os06g26190	KFPQESCSHKESSEFCPNSITSLCE	S	114			6765	LOC_Os06g26190	VVDTFIYHKYCKSLGTCIFCQEEMH	S	155
6767	LOC_Os06g26340	IQGVHANIAGEPSTIVRPAVSSERS	S	461			6768	LOC_Os06g26340	QSDTATAQAHAASHGPVPAVSYSLP	S	552
6770	LOC_Os06g27600	VVGRIAKWVVELSQFDVHFVPRTAV	S	136			6771	LOC_Os06g27970	PAGTNPDPVSVVSYLNNLKSSEESL	S	124
6773	LOC_Os06g28140	TSDMVVYLMRHKSESFEKFKEFQNE	S	296			6774	LOC_Os06g29310	GEFSPTFSMRLRSRKVVEEVVLLDG	S	200
6776	LOC_Os06g29310	LLEKSRRIQGELSGGIERRLKDRGA	S	37			6777	LOC_Os06g29310	SFGIDEEAGVDVSHLETSACIGDPK	S	112
6779	LOC_Os06g29400	PLANQPVDPVKTSEDANMVLNRLVA	S	752			6780	LOC_Os06g29410	EKDARLEAKIRASIKAELTSEFDEK	S	313
6782	LOC_Os06g30020	EKSCAVKSFWVDSQAKEGVVLDDDS	S	236			6783	LOC_Os06g30020	IPPNLNEVNPLASEGESGPVDNTKA	S	40
6785	LOC_Os06g30020	YSSRQYSSQYSTSSETSSRHSRDRH	S	522			6786	LOC_Os06g30320	EAYRDLSMERKLSKPANKLLPLNCR	S	750
6788	LOC_Os06g30320	KKMRYYEYTLLRSYKAYLQKLISLE	S	286			6789	LOC_Os06g30570	ASGKAAKNYVRHSGWVVRDNVPVST	S	133
6791	LOC_Os06g30710	LRDRLDWLQALRSRDPLVLRDAQLK	S	94			6792	LOC_Os06g30710	PKTRFSRDPSLGSRSPSTRQGSTPP	S	491
6794	LOC_Os06g30901	DALLLLENALEGSGGGGSGAAYLEA	S	180			6795	LOC_Os06g31250	ETWTMAFDGALNSQGAGAGFILTSP	S	1113
6797	LOC_Os06g32060	DLFDQLKGATVFSKIDLRSGYHQLR	S	853			6798	LOC_Os06g32060	VIFAAHQLQGPASLWWDHFQATQPE	S	332
6800	LOC_Os06g33570	IERKDACWRDMCSNNSTVCNQAYLY	S	277			6801	LOC_Os06g33570	MRVKSRDTDQWMSYRLLPENLKERI	S	408
6803	LOC_Os06g34420	VEGLKQGYCVIPSERRFLVLYAFLK	S	323			6804	LOC_Os06g34420	VGRTARGDNGKGSAILFLLPKELQL	S	374
6806	LOC_Os06g34710	DTAMAAVENDAGSLAETVVMTHEEF	S	427			6807	LOC_Os06g34710	EIRNEVSYDDGFSGKMGSLHGMTST	S	551
6809	LOC_Os06g35730	VLEQLSGQSPVFSKARYTVRSFGIR	S	50			6810	LOC_Os06g35800	WQWRVVEVASTGSWRKFVSKVREKG	S	116
6812	LOC_Os06g35814	SNLLSRFTRNEFSLESKSTIGVEFA	S	40			6813	LOC_Os06g35814	VLTQIYRIVSKRSVEAGDDAGSGPG	S	183
6815	LOC_Os06g36360	RQCAHCAFFMGLSRKEGAKIQEGQQ	S	440			6816	LOC_Os06g36700	ASRIAFDHLEHISHKFEFSATNIEP	S	148
6818	LOC_Os06g36710	PNLPEPSKFSSGSVGSSIARPNDDY	S	96			6819	LOC_Os06g36710	PYGQQQSYGGQQSYGQIPGSSGFSS	S	21
6821	LOC_Os06g36830	KNPSINIVCVEPSESAVISGSSIKH	S	236			6822	LOC_Os06g36830	SIPTEEPRKESSSSFWRNRRGDLDM	S	356
6824	LOC_Os06g36940	AEAEAEAACSGDSDAEAEDLNFDLS	S	184			6825	LOC_Os06g36940	DWNWFLEEVRKGSDDEDDEEGKLKG	S	238
6827	LOC_Os06g37290	DTSPGPRHDAAGSSAAPEVIAEIEQ	S	885			6828	LOC_Os06g37290	LVSWSARKQATVSRSSIEAEYKALA	S	1285
6830	LOC_Os06g38470	DVEMDDVKPLDDSGRRSSIQNVRVK	S	433			6831	LOC_Os06g38490	NLQFMDFGKNKLSGSIPASLGNLFS	S	137
6833	LOC_Os06g39344	TNPSVKCVLVESSSPRAFSAGMDIK	S	116			6834	LOC_Os06g39480	QHAVIASAVPLSSKPTNERQHHGQD	S	454
6836	LOC_Os06g39600	KEFGVESFEDGPSAFYGVFDGHGGK	S	104			6837	LOC_Os06g39600	TDAAFADACSVNSSLASGTTALAAL	S	163
6839	LOC_Os06g39906	ARVFLARPLLRRSPRGVACALRRRP	S	32			6840	LOC_Os06g39912	AGLQRSATTFRRSGSSGLVWDERFL	S	15
6842	LOC_Os06g40440	LGAAVPLRRLLTSTGLDADTGRGRE	S	101			6843	LOC_Os06g40460	LQEGDCRNQNDGSGLAEIENIIKQK	S	133
6845	LOC_Os06g40630	GTGPSASSASSSSSPPQPSLLAEWN	S	19			6846	LOC_Os06g40650	PLPPPMRNAYSRSTSFDQHSGVYSR	S	349
6848	LOC_Os06g40740	GRQRYASVDRRASMDRHRWCDSDND	S	195			6849	LOC_Os06g40740	PKTQGASTACSTSSRTMSSRGSWSP	S	510
6851	LOC_Os06g41120	RLPGRRRRCGRGSPPLPLLDPAPTR	S	67			6852	LOC_Os06g41440	PSFPSYSPIPRLSVSEISDDHGGGV	S	309
6854	LOC_Os06g42200	EGAEAAAQQLAVSLSLREAAQEEQA	S	812			6855	LOC_Os06g42490	DEEAAITTSNVPSFWNRQQCNGDWS	S	653
6857	LOC_Os06g42790	KDYAGFSPILIKSAFTDKKSFFYMN	S	338			6858	LOC_Os06g42810	DAARAPPAAEAQSLVESFCGVTSAT	S	17
6860	LOC_Os06g43170	QMPGAGASPCGSSSAATSAAAAAAP	S	738			6861	LOC_Os06g43370	MLRLGEVPTLVVSSRDAAREVMRTH	S	87
6863	LOC_Os06g43560	NREKQRSTRARFSREKILHECRLLR	S	561			6864	LOC_Os06g43610	NLWLRLDAPPFPSFQFGMAGAIYIK	S	75
6866	LOC_Os06g44030	TELALQEERNRISTSSVSDVALARS	S	34			6867	LOC_Os06g44130	RRGQPIPTTRVISSPSRALRGPPER	S	262
6869	LOC_Os06g44150	FRALELALEAACSFLDAQAAELEIE	S	183			6870	LOC_Os06g44950	GVPAGKLLGFLVSARGIEANPEKIR	S	1032
6872	LOC_Os06g45560	KEITLQNRRYTWSNEQDNPTLDTAF	S	934			6873	LOC_Os06g45710	AKGVTTIIGGGDSVAAVEKAGLADK	S	360
6875	LOC_Os06g45980	QIGDAFLSYERVSGEFRDADKDTAK	S	694			6876	LOC_Os06g45990	KDIALWGVPLLPSKGDDATDVVLLK	S	175
6878	LOC_Os06g46680	AAVLAAMWWRRCSRTGGADGLPPGP	S	89			6879	LOC_Os06g46890	HRPRSRDRGSSHSRSPIRKRHRKKL	S	103
6881	LOC_Os06g47350	RRDDMEVAEEDISDSQAFRFVEHVL	S	18			6882	LOC_Os06g48330	APLLSREGIILFSKGRATARDLVKQ	S	548
6884	LOC_Os06g48350	KKKHEASHKEGASNGSAKHVVEEAK	S	286			6885	LOC_Os06g48350	YDDLVKEMKDNLSKGATAVQLKGLM	S	314
6887	LOC_Os06g48530	RGGGGGRGGGSSSPPPYRSGRRHSP	S	95			6888	LOC_Os06g48780	SAAGVQSSFSMVSPSSAVFQVIIGA	S	59
6890	LOC_Os06g49090	GYIKMPGETRRESNEPLKATKLSRI	S	655			6891	LOC_Os06g49380	PSNVAMNRPVTTSGIIDPEFYGRKG	S	272
6893	LOC_Os06g49760	IRGKIVEVAQVTSNALALVNRVAPA	S	187			6894	LOC_Os06g49830	NNSAAAAAAAAASKRPRELVRTLGR	S	203
6896	LOC_Os06g50030	ALTRYLTDAMKESRVLEFLHALEPL	S	514			6897	LOC_Os06g50350	ASLVLGTESFAISSESGILSEQLAA	S	29
6899	LOC_Os06g50580	GVRGYTSTLKNASTASCRLSAGHPI	S	31			6900	LOC_Os06g50706	RRSREPQPTRRRSVFHPGSWRITTW	S	321
6902	LOC_Os06g50818	WSEFTSTLRLYRSSCSSGSWTSEEV	S	167			6903	LOC_Os06g50840	GLDGIRMLDPNTSRTLRIYPLETVT	S	229
6905	LOC_Os06g51140	RGWRGMEPPREPSPGDLALAADGKR	S	93			6906	LOC_Os06g51220	YNKAIAAYNKGESTAKKAPAKEEEE	S	115
6908	LOC_Os06g51400	DRDHSSSGSASTSAQAKKPASTNAE	S	21			6909	LOC_Os06g51400	NGKRKQTQAGWLSEEGRKKRTPASQ	S	160
6911	LOC_Os07g01320	ADYIRKQGRVSISHLASNSNQFIDL	S	268			6912	LOC_Os07g01320	GPAARRMRRRPLSTSADASTSRDRD	S	66
6914	LOC_Os07g01550	KSNLDVHCAGPSSPDVYERMNFEDN	S	277			6915	LOC_Os07g01720	GRDSKRVRRSRRSTSYSTSGESNSS	S	135
6917	LOC_Os07g01880	LAERRIVSAKKSSMGRNIPRKPSFP	S	546			6918	LOC_Os07g01930	NRAVGNLASSTTSYYRGQLSRRFVP	S	28
6920	LOC_Os07g03070	DSSTLSTAATTGSTGSTVTDLGVVG	S	407			6921	LOC_Os07g03180	LLLVLALSVILVSATAFHHHHHHDG	S	25
6923	LOC_Os07g03980	KRSFTLPARMSSSTTSTPRRCVNGT	S	130			6924	LOC_Os07g04190	QLRHLHLGGNFFSGGIPPEYGRWGR	S	179
6926	LOC_Os07g04530	GDTRKLERWSDDSSKYSVDGRRPPQ	S	134			6927	LOC_Os07g05120	SDPVAPATATEDSALLRARHLLSKA	S	136
6929	LOC_Os07g05190	SSKNLSLQVLDISGNHFNGSLPDEI	S	515			6930	LOC_Os07g05350	DGGGCGVSGPAVSGSLTRTDGVGCK	S	140
6932	LOC_Os07g05610	AAACCINTGSSTSTGLSGWQLTVDA	S	887			6933	LOC_Os07g06175	WDHDFAASLLKLSKLPMPVGSKGEI	S	307
6935	LOC_Os07g06300	GGMTKDDFSYKESEAKLLQSLRFCI	S	1136			6936	LOC_Os07g06300	VPSTLDGNENAGSSAYSKKYHSSPD	S	1005
6938	LOC_Os07g06740	DFSSDPWPAISDSAKDLVRKMLNRD	S	336			6939	LOC_Os07g06740	IFEKVLKGDLDFSSDPWPAISDSAK	S	326
6941	LOC_Os07g07040	PIEEKPAETVEGSRECKNNEKEETH	S	259			6942	LOC_Os07g07194	TGCWQKDAANTKSLAFDGWCITLYT	S	127
6944	LOC_Os07g08190	SSKKDPDNTKTISLEKDSTLEDADK	S	343			6945	LOC_Os07g08710	KASPKAKAKTATSPVKPRGRPAKSA	S	220
6947	LOC_Os07g09010	LGYPARSTNTFVSLISIWNYAGRVA	S	406			6948	LOC_Os07g09340	AIEEMAGMDVLCSDKTGTLTLNKLT	S	332
6950	LOC_Os07g10050	LDAGGRSSATKTSGTATTGSASSRR	S	132			6951	LOC_Os07g10110	SEEQQLALLANASLLAEELLPRAAM	S	528
6953	LOC_Os07g10770	DALLNDETRQPLSRKVPLPSSRINP	S	262			6954	LOC_Os07g10940	SKTKALIHSYGGSPGQNVVVVVVVP	S	461
6956	LOC_Os07g12220	TPEEKYGQLFVLSLKLALWMQIIGF	S	123			6957	LOC_Os07g12490	FSPARTLSPQIRSNPEADSQYLSEL	S	23
6959	LOC_Os07g13240	ETTIKASNVAPESEAKVEDVKYKSK	S	53			6960	LOC_Os07g13240	PMTHQDVHGDMTSDNSIIVAGNSLI	S	91
6962	LOC_Os07g14800	NLLEEHRERVAVSAASYQQALRRYH	S	1677			6963	LOC_Os07g15490	RLPSPSLDASSLSHSDLLLHAIPAD	S	59
6965	LOC_Os07g15890	ARHGVFLEKEFISRKDSGSMLWKHR	S	643			6966	LOC_Os07g15890	KLQKSIYGLKQASRSWNIRFDEVLK	S	840
6968	LOC_Os07g17440	IWGCEVYVKRLQSDKLTTKLDKCFF	S	376			6969	LOC_Os07g17440	LEKEFISRKDNGSMLWKHRLHEGPK	S	429
6971	LOC_Os07g20240	VSCRSAADVVQTSGRRPSPPLPRRS	S	652			6972	LOC_Os07g22024	EFADSSKARNSLSVDNTSSRGGSKF	S	361
6974	LOC_Os07g22534	NLPPLQSLRKVCSANGGSESRAVIV	S	333			6975	LOC_Os07g22640	GTLHLQVTYHPFSKEEQMEALESEK	S	392
6977	LOC_Os07g23290	FADLDSCPTCGTSKYKTGNGASDGE	S	222			6978	LOC_Os07g24020	ACWVKHGEQESGSGAAADRSGVHNQ	S	91
6980	LOC_Os07g25460	HKDKEVMNALEGSGAPASEAEVQQE	S	240			6981	LOC_Os07g25620	YPFPANAFSSAASARTFTATESTTD	S	478

6983	LOC_Os07g25680	SDSAKSLETYTASKAMSAAQDSECN	S	516			6984	LOC_Os07g25810	AKGDDNAAGEEKSLWLKKQFGKGLG	S	42
6986	LOC_Os07g26440	ASFGYDPAMDYESNMMVRAVKVEDS	S	697			6987	LOC_Os07g27520	QGIKTRVYDRLLSHDKKWVEELPLV	S	1479
6989	LOC_Os07g28430	AALQEEMKALVESVPVGAGAAFTAM	S	106			6990	LOC_Os07g28800	FCQRNHRRSHSESSRRWSYETDDED	S	184
6992	LOC_Os07g29450	VQRIEPRDDKRPSLAHRMPIGPSNF	S	68			6993	LOC_Os07g30040	NIKVARVLIDGDSLINLLFASTLDA	S	514
6995	LOC_Os07g30774	EEINSHGGISASSETGNHDSSLEEF	S	204			6996	LOC_Os07g30774	VVMTVDAEAEPASTAEEDFFQAGRQ	S	864
6998	LOC_Os07g31370	MVDVNLKSSNANSSQSQAQAGGCSC	S	196			6999	LOC_Os07g31450	QMNSYKGSVCGPSTKEQKRASGELA	S	1426
7001	LOC_Os07g31630	AYWGAGAGVTLTSPSGDVLRYLVRL	S	1111			7002	LOC_Os07g31630	CMEQRADHLAAASKPAGGDERLSTS	S	486
7004	LOC_Os07g32350	QRRPVISVDFRESPIKAVAEDPNGH	S	262			7005	LOC_Os07g32420	PKGVTAGTNSAVSKTLATALSSKVK	S	206
7007	LOC_Os07g32430	RSHQLTFSSTSASNDRESDRSPSDA	S	329			7008	LOC_Os07g33340	KRHLKSRILVFLSSVKQVKFVYEVF	S	333
7010	LOC_Os07g33630	DNQGDERGPIWESWTRAMDAIHVGD	S	757			7011	LOC_Os07g33630	RVVEEMARDISLSSGRRGGGPMLGF	S	578
7013	LOC_Os07g34740	EGDAEEQQGEEESSSSDDDDDDEDE	S	101			7014	LOC_Os07g36200	KVISGRWNSSKPSSPPAAPVSVPVV	S	177
7016	LOC_Os07g36940	HRDAAQERHAQSSRLARGSVVGSGP	S	1435			7017	LOC_Os07g37140	GNHSGVKDAVAESNGGPDRLSVGQQ	S	676
7019	LOC_Os07g37270	VLQKCKVKFKILSLKVKGPMDYAPE	S	186			7020	LOC_Os07g37610	ITDQELQHLSGDSNSVITPLFEKML	S	445
7022	LOC_Os07g38090	MSPRSPRTMEPTSPLSARVGAPATQ	S	537			7023	LOC_Os07g38160	IYEIYYTTDPKDSSKDYLCTVRCGP	S	97
7025	LOC_Os07g38360	TNMSTGGGDLSRSSNAPVASGSPSN	S	1235			7026	LOC_Os07g38530	RQPYLEHAYSNVSMMSNGLSESIDE	S	317
7028	LOC_Os07g38730	PQVSTAVVEPYNSVLSTHSLLEHTD	S	187			7029	LOC_Os07g38730	WYVGEGMEEGEFSEAREDLAALEKD	S	419
7031	LOC_Os07g39480	HPNCDVKKLLERSLDGQITEVVYKG	S	275			7032	LOC_Os07g39480	MQESQPESVAEKSAEDGYNWRKYGQ	S	234
7034	LOC_Os07g39620	AETHVAGVRVVQSFSTDNSTADSAG	S	253			7035	LOC_Os07g39620	INGGGRNPVFDQSLRLGVRAGDVDG	S	81
7037	LOC_Os07g41160	KRKRLERRNSMNSGRSAGAGGGGRD	S	133			7038	LOC_Os07g41230	PSASPPPVAKRRSSVSSRRGGGGGG	S	25
7040	LOC_Os07g41694	DEDAVVEVHDVPSSSDEEEDGIEEE	S	297			7041	LOC_Os07g41694	QAAGEGSSSPARSLTLDGAVKCLHG	S	26
7043	LOC_Os07g41800	TAAQPSPIRNSPSRSLSFSHLSRLR	S	175			7044	LOC_Os07g41810	SSSRAAAAAAMSSAPATAIAAPVEH	S	39
7046	LOC_Os07g42220	LPPPRKVRSTPSSPRRDVPTSATAI	S	124			7047	LOC_Os07g42410	SKQKEQTSSSGNSAACTSKVVPTKG	S	328
7049	LOC_Os07g42770	KESRVQEILVSLSALQYRRMDFQEF	S	353			7050	LOC_Os07g43050	RSPSASPARRSPSASPVRRSPSRSP	S	237
7052	LOC_Os07g43350	RLVEVDLSRNGFSGSVPVVNSTTLK	S	309			7053	LOC_Os07g43750	QERREGKAPAAPSGNRRSEAKQKAP	S	406
7055	LOC_Os07g43810	DAKVIYDRESGRSRGFGFVTYGSAE	S	220			7056	LOC_Os07g43810	LYVGNLAWGVDNSTLENLFSEQGTV	S	194
7058	LOC_Os07g44640	ENKCWSCGQVFSSNSELCGHLHALE	S	121			7059	LOC_Os07g44970	EEAGRKNGMDKLSKKFLADCWLNDE	S	351
7061	LOC_Os07g45360	AAILRKKFDIHISGQNVPAPLENFE	S	133			7062	LOC_Os07g45400	NNTSLAATVSDSSGLRNNAKSVDEK	S	662
7064	LOC_Os07g45740	EKAAIETSSDDASSSADSGISSAAH	S	102			7065	LOC_Os07g45740	SLSQKSRKGGLGSILRGSCSYKKAK	S	138
7067	LOC_Os07g46170	GYVELTIPGVIISIVGLLLFGKLLE	S	82			7068	LOC_Os07g46180	PSNNSLDAIAKVSRSVMTKSPTISS	S	545
7070	LOC_Os07g46440	TPASAATAAAASSSPVSAPLPFRRP	S	33			7071	LOC_Os07g46540	LAALESLVSSLVSKGEISSNTITAL	S	648
7073	LOC_Os07g46590	KKGGSMFDKNELSAILRFGAEELFK	S	359			7074	LOC_Os07g46750	KKAADERAASKASSKKKESGKSSVL	S	127
7076	LOC_Os07g47030	VGKMTDNLIGDCSTSSKKRKKACKS	S	33			7077	LOC_Os07g47180	FEKIDKIGQGTYSNVYKARDSVSGK	S	138
7079	LOC_Os07g47360	NDVNCAFDATRKSQMAIASAASNQE	S	1552			7080	LOC_Os07g47840	PSDDYPDRPPKFSGAEEATGGGDHG	S	64
7082	LOC_Os07g48020	AAAMASVASAQLSATFYDTSCPNAL	S	28			7083	LOC_Os07g48020	EALANTDLPAPSSSLAELIGNFSRK	S	163
7085	LOC_Os07g48410	AAAAAAHSPFMLSRQNSGRCGTAPS	S	89			7086	LOC_Os07g48602	STLPDLSPLMTGSKFSLSPLTVKTL	S	32
7088	LOC_Os07g49270	LNVDLLDVHADKSTFHRFDKFNLKY	S	431			7089	LOC_Os07g49380	KLSHPAWPGEVISAAPTGAQLLVSF	S	83
7091	LOC_Os08g01054	DIKDGSCDIRDSSLAAVKRKKPKDL	S	184			7092	LOC_Os08g01054	EICKAYDDLPKSSETTTCMLPDDQS	S	117
7094	LOC_Os08g01130	ESESSFSTQSSTSDVDNQLIDLSSP	S	539			7095	LOC_Os08g01420	IELAEEREGSSVSSMGEDNEPDLST	S	404
7097	LOC_Os08g01620	NVISKDESGSQPSVVCEIEPSILRS	S	210			7098	LOC_Os08g01794	LAVIENDTAAVNSNISDQQIMEVDS	S	413
7100	LOC_Os08g01900	QGCSLQLCQLVNSLLPSLTGRACTC	S	114			7101	LOC_Os08g01930	PPNYPPGPGNYNSYGPSQGPNYGQP	S	433
7103	LOC_Os08g02060	VKPLPPVMAYSVSRNVNFFFKIFTQ	S	147			7104	LOC_Os08g02340	WPGLFAKLLEHRSVDDLILSVGSGG	S	54
7106	LOC_Os08g02354	QEALVKSTKDNFSSPGSPRIDDQSA	S	424			7107	LOC_Os08g02410	SSSALPYKRTPPSWLKTAASDVEEM	S	24
7109	LOC_Os08g02690	EKEMASILLSSLSMELFSTEDIVKG	S	498			7110	LOC_Os08g02700	AVPAIVFLSGGQSEEEATRNLNAMN	S	272
7112	LOC_Os08g03290	VLPDLNGKLTGMSFRVPTVDVSVVD	S	234			7113	LOC_Os08g03390	QAAPSQAELLFKSFKIKKEKLKSEN	S	327
7115	LOC_Os08g03520	PDDGGEDLFVHQSSLKSDGYRSLND	S	36			7116	LOC_Os08g03640	SALLAKLGIRPFSYGLVITNVYDSG	S	187
7118	LOC_Os08g03840	DSVNVIAIKVAESDVGYPIRVFGTV	S	205			7119	LOC_Os08g04270	WSMKEFWPYVEQSFTWTDLPSKFPT	S	218
7121	LOC_Os08g05540	VMLKLQANSHLVSSSSSATAPNLPP	S	43			7122	LOC_Os08g05840	HKIRDTYTKDFRSKDKTKKQIAVAT	S	622
7124	LOC_Os08g06020	VKDITGEMFTKESGNKVEEVKLRVT	S	118			7125	LOC_Os08g06070	NDREKQIKAIEDSFRAAKSRPVHQT	S	223
7127	LOC_Os08g06200	STDLPSLSSGMPSPDMPDDHDKTFS	S	356			7128	LOC_Os08g06344	QAAAAMVAAKAASNANQQTQGTSVG	S	895
7130	LOC_Os08g07760	PPPPYNPPTPVQSPGSSSSTGAIAG	S	229			7131	LOC_Os08g08080	DLSPPRRKGRHDSEEPKDLLPPWRR	S	254
7133	LOC_Os08g08240	HILMSCKGSDGHSNGTSHLWRHQKI	S	69			7134	LOC_Os08g08570	ATKTHYDFQQKISDLFLEYQRELNM	S	112
7136	LOC_Os08g08610	SVLWAVCTTPTTSNKETPFFLVYGS	S	728			7137	LOC_Os08g08820	NMLILQESCTDASGSYVVYAPVDIV	S	677
7139	LOC_Os08g09350	KQPQQKKAQEESSEESSEEDSDEED	S	263			7140	LOC_Os08g10350	AINVMIWDKHASSFGLVCLLFTLAG	S	280
7142	LOC_Os08g10580	SGLVRGNTTVAASNGSYSATPSRFV	S	173			7143	LOC_Os08g11390	TARRRERRTPTDSGRHGELTGDQRN	S	74
7145	LOC_Os08g12760	DGQPYLPLHFPFSSPYYQPPASPSM	S	149			7146	LOC_Os08g13350	LGRSNYSGSISSSVPGAGGSARAKS	S	101
7148	LOC_Os08g14330	RLRVALGPNGDLSLTSRIRNTNTDG	S	169			7149	LOC_Os08g14460	LPFHMARDQIEESLRTKMASIKARM	S	346
7151	LOC_Os08g15660	IVALQSLVTVERSVRGDLEEHIPKP	S	71			7152	LOC_Os08g15720	LTWLKQAELEDFSESDLKEAINDLP	S	35
7154	LOC_Os08g15840	NDGSTIDLIGSGSTPLHYAACGGSA	S	225			7155	LOC_Os08g16130	LINLLASLMYYWSYSRAATGGFIKH	S	167
7157	LOC_Os08g16200	RLKAELEKEKAHSVVLIDYYNLTEP	S	242			7158	LOC_Os08g16530	DDWRTPLTEFQSSDELPEDDAEAEK	S	662
7160	LOC_Os08g17120	YAHGGTLNPVDGSLVFSDQIREAAH	S	173			7161	LOC_Os08g17750	DASFQTDKDDFRSQSGFVFCLNGGA	S	1246
7163	LOC_Os08g17990	CLGMVKLWSDTGSSSSSSKYNGPES	S	1641			7164	LOC_Os08g17990	IDCKWVYKIKRKSDGSIDRYKARLV	S	829
7166	LOC_Os08g19320	WDIANAIIEFFVSNCNPRDMLSILC	S	145			7167	LOC_Os08g20020	SQLRDIVQNKFPSLKALLIKYKDKE	S	353
7169	LOC_Os08g20660	NERLSGSVGFVLSTSRAISEVHSLL	S	820			7170	LOC_Os08g22130	PTLLLSIPGTKCSTAFLSKFFPMGK	S	113
7172	LOC_Os08g23200	FRSGSSNDSGSASNPSGNTGFDASR	S	34			7173	LOC_Os08g23200	RAAGLMVEQKFASTIVMLKMFRAIK	S	326
7175	LOC_Os08g23440	VIQMTSMEPGSSSSTAVAAVSGITP	S	47			7176	LOC_Os08g23680	TKDNPVELEVGQSLRFAASTRTYIL	S	188
7178	LOC_Os08g23930	EFPPSQNGVGKKSSDCIEILHTESL	S	361			7179	LOC_Os08g24160	CRATQYPAVCVQSQASYGGAPLPRS	S	43
7181	LOC_Os08g24434	PLMPAGGEVTGGSKAAKKRGMQKLL	S	63			7182	LOC_Os08g24730	PGATAGRPHLSPSDPEGAGAGVTLT	S	1213
7184	LOC_Os08g25090	LLDVADNLSRASSVVKESFSKIDTS	S	219			7185	LOC_Os08g25090	SSPGTENASQAGSQDSVPQSNRRRR	S	115
7187	LOC_Os08g25850	GGGKEREVLKIDSETKSNQPPTKKA	S	36			7188	LOC_Os08g27040	IKEGHKSFPSGHSSWSFAGLGFLAW	S	168
7190	LOC_Os08g27150	ALQRSIEVQRRGSMKYFAQERKKSI	S	702			7191	LOC_Os08g27150	GKNYRRPTKSAPSNLLTKREALQRS	S	682
7193	LOC_Os08g27170	LIPFHDEKPMISSGMPSYINQAEGL	S	421			7194	LOC_Os08g27840	YNTRERARQLLSSGYSDIPEETTLT	S	388
7196	LOC_Os08g28570	IWNIIKGAFSMSSQCSKIITTTRIN	S	254			7197	LOC_Os08g28670	LKEVYDPYHVTFSFAPVPGKEGEQC	S	100
7199	LOC_Os08g31240	LKVFYDHWKEHNSDLWGSSNAIAIA	S	40			7200	LOC_Os08g31350	IPIEQAMATRRRSKRSAVRVEPDSD	S	89
7202	LOC_Os08g31470	TLLDLMIIRAFHSKILRRFSLGTAI	S	108			7203	LOC_Os08g32100	HVQASKMLELLQSILDVEAEMFVLK	S	885
7205	LOC_Os08g33190	HGGQIIPEQEPQSKIEFPDLDGLGG	S	210			7206	LOC_Os08g33320	SPERRFSTSGNRSVRYGPDMDKDHA	S	1181

7208	LOC_Os08g33380	LKEKWDAIENKSSVLFYGGGAIIAV	S	86			7209	LOC_Os08g34060	NDEFYDALARGESFEDEDSDDDDDM	S	475
7211	LOC_Os08g34770	LTLPESYNHLENSIQPVPSTQNEST	S	161			7212	LOC_Os08g34800	PARTVTRTSSAASSELLRTSSAGSS	S	105
7214	LOC_Os08g34950	KYCDTLGGLRVGSRSSDSNDKSVDT	S	1213			7215	LOC_Os08g35060	ASELQLQEVIMSSSVAATTARSSSA	S	66
7217	LOC_Os08g35110	WHSSSRIARAPPSAGELEEGSAAAA	S	29			7218	LOC_Os08g35270	PSYTPGHTPVASSRRGPRFIRTARK	S	238
7220	LOC_Os08g36790	VAPVDTSVGQMDSAGKGDGDLSSPM	S	205			7221	LOC_Os08g36930	ALERAEGSATSTSTGGGEGRRDGER	S	55
7223	LOC_Os08g37104	DEDRSSSPSVSGSCESSDGAACAGD	S	291			7224	LOC_Os08g37104	TCLLNIFSQMRSSSDQDEDRSSSPS	S	275
7226	LOC_Os08g37800	YPQATDMLDVNHSFVDDSIIMDNGD	S	446			7227	LOC_Os08g37920	FSGSFSNNDGAASIVLPSSGLNNKI	S	625
7229	LOC_Os08g38050	SFLARRHANTTDSRSSGPPRKIAAI	S	87			7230	LOC_Os08g38310	NSSFEGIEINCQSDHALYSLCSTMS	S	532
7232	LOC_Os08g38410	TSQPRGFGFITYSNPAVVDRVMDDI	S	72			7233	LOC_Os08g38590	TASCSYVPSDENSGTPVMAYDSNPP	S	320
7235	LOC_Os08g39140	EITKEEYAAFYKSLTNDWEEHLAVK	S	288			7236	LOC_Os08g39140	ERIMKAQALRDSSMAGYMSSKKTME	S	596
7238	LOC_Os08g39590	GGPTASPTPLPWSMRLSIVQGAARG	S	462			7239	LOC_Os08g39810	CPDMNREPMKMTSEATKSWCPIHKT	S	579
7241	LOC_Os08g40150	KEPKQHAHSEPASAPSKAVPTAGMG	S	299			7242	LOC_Os08g40230	TDVDEFSKMENKSVKLDDPPGEGPV	S	252
7244	LOC_Os08g40820	QRFVENEPFEQGSRVEIYARSRSSL	S	278			7245	LOC_Os08g40830	YASYDRPSYPPPSPQEQESSQWDFF	S	207
7247	LOC_Os08g41260	GRRAAHATEGASSSGVSHQHGCGDQ	S	198			7248	LOC_Os08g41400	GIDERNPNVNLTSDEVNEVHGIDVE	S	536
7250	LOC_Os08g41710	RKYPEHFVINTRSKGRVTSEFVSLV	S	327			7251	LOC_Os08g42110	RQLKESSLTMHQSVQETEKILDSTE	S	174
7253	LOC_Os08g42370	LSVWKAMLKTPASIVLIIYCFLCVW	S	239			7254	LOC_Os08g42520	DYHLLKISGLNSSEWDTMKLVTALK	S	184
7256	LOC_Os08g42700	VPIGKLHHLKYLSLRGSFGVFNLPN	S	881			7257	LOC_Os08g42710	NEINSETKPSMASSSTGGDNHPEGL	S	603
7259	LOC_Os08g42740	RKLTCGICFEGYSSDVMSSADCDHF	S	163			7260	LOC_Os08g43060	LGSLLPRIPKEPSLLDERSLLACIV	S	603
7262	LOC_Os08g43110	SELGFVPSKADTSLFFYKKGQVSIF	S	1103			7263	LOC_Os08g43130	RKVKFVEGSSSGSHGAGSGSLQRFA	S	1337
7265	LOC_Os08g43400	SYHMTEAASRTASEVSEEHCREVQC	S	548			7266	LOC_Os08g44050	KRYAVQSDWKAHSKTCGTREYRCDC	S	165
7268	LOC_Os08g44430	KQSLIYSFVAKGSVVLAEHTAFSGN	S	17			7269	LOC_Os08g44480	NDLMTRGLIRMVSVHSSQQIYTRAT	S	94
7271	LOC_Os08g45060	HAGHGEAHGSVRSSNVLVDEWFVAR	S	498			7272	LOC_Os08g45060	PIHSLNLSYNNFSGQLPPDLASLPP	S	260
7274	LOC_Os09g01640	HRKRQHHHRKGASDGDSGSGASDSA	S	323			7275	LOC_Os09g02810	NSHVKHEDPCAGSDLEMDSRILSAL	S	467
7277	LOC_Os09g03600	NVKRRSEELACASRQQTDSANGTGN	S	101			7278	LOC_Os09g04110	LVKNLKNEIVKKSPLPIAMDAIYYA	S	449
7280	LOC_Os09g06560	FTLKGEHEQPVCSLCWSPNGRYLVT	S	361			7281	LOC_Os09g07170	DVLIPSGHEVGESSSNKFEARTNSS	S	310
7283	LOC_Os09g07430	YGGHYGAFGTQDSKEFVRRCTSCQR	S	582			7284	LOC_Os09g07500	ETIVCVYRIRDLSHPQTRFKVDVNA	S	485
7286	LOC_Os09g07660	LQATGDLSPVRRSIQFFLDNLLSQT	S	512			7287	LOC_Os09g07690	CKLLRYLNWKNGSKLWRMTKRDSAL	S	587
7289	LOC_Os09g08450	SAAELNHYNNILSIGETQQYKYKFA	S	166			7290	LOC_Os09g08450	SSPQTPLHINIASARTPATKQVIQI	S	61
7292	LOC_Os09g10710	LPKHVSRKDFPPSQNGVGKRSSDDI	S	303			7293	LOC_Os09g10930	NPKPVVVAANTPSASKRPLELKGGE	S	55
7295	LOC_Os09g11170	SSALVPAAECSSSSAAAMTLVASVV	S	93			7296	LOC_Os09g11240	AGGGGGSGDGDESAAVAATRRRFVP	S	86
7298	LOC_Os09g12240	LAACSAVTTLDVSWNHMSGGLPPGL	S	235			7299	LOC_Os09g12270	DESESYGEDVGESDTDQEFNASDDS	S	118
7301	LOC_Os09g12730	VILKFEQSDPEVSSPTRKPETFVSH	S	626			7302	LOC_Os09g13630	GSFKGLGCHLNASHDLFHYEFWISE	S	330
7304	LOC_Os09g14670	MNIGSRPSKRKPSGGIESLRAIPWI	S	770			7305	LOC_Os09g14720	RSWLMHLPPYSISSWADLWQQFVAN	S	483
7307	LOC_Os09g14940	EPETRCNEYLDISCLMLATMSPELQ	S	51			7308	LOC_Os09g14990	TKAPFMGQSERASELLGLVHTDNEV	S	275
7310	LOC_Os09g15130	LMPLDISFKPRTSIKSQALADFVTE	S	287			7311	LOC_Os09g15750	DKLMTAPSDSSGSGSSGNWLFKNKE	S	397
7313	LOC_Os09g16330	GVQNSSSVQPVVSVERTVFYRERAA	S	229			7314	LOC_Os09g16980	CHAYDLGYKCHCSDGYQGNPYIRGG	S	298
7316	LOC_Os09g17850	LRLLETINSISESENIYDVTIEDPS	S	265			7317	LOC_Os09g19400	LYRRRNLGPTTASVTRSADSHIHAG	S	185
7319	LOC_Os09g19590	QYPLYPVYGYPPSTVACQVGGLGVQ	S	25			7320	LOC_Os09g19640	ENGLRLTVADIASHIQHEIQYGGDN	S	79
7322	LOC_Os09g19734	VVKARFNNSGKASALLQHEWRPKSV	S	236			7323	LOC_Os09g19750	CLEVTPFSPPPNSITASLIYYRSPQ	S	80
7325	LOC_Os09g19830	WGVRSLSSISSGSFVCEYAGEVLQE	S	1045			7326	LOC_Os09g19910	KIETHKNETHGTSDDINENTPVERV	S	90
7328	LOC_Os09g20010	HTLLSFPPSQDDSSSLPPPQNVVLN	S	62			7329	LOC_Os09g20460	SSSTPLSTSTPSSSNLAAANANATT	S	249
7331	LOC_Os09g21110	YDLQLARDEYRLSCGAAGMNRDLLW	S	839			7332	LOC_Os09g21770	GTKRPSNSYPASSNSDSKVEPKKRK	S	203
7334	LOC_Os09g23350	QLWPLFHYMLPLSPELGGRFDRALW	S	186			7335	LOC_Os09g23570	LCGGPLSPCTNTSPPSPSPSPSPPI	S	235
7337	LOC_Os09g23740	FFAPGTYFSHDSSDSDSSVSVANSM	S	109			7338	LOC_Os09g23740	KGEAVAVFGEMESLHAEVYGFLSNT	S	934
7340	LOC_Os09g25570	DSSGVLYFVSSDSSQVCLALASLKS	S	425			7341	LOC_Os09g25570	ESSGSLVLGDDTSVYRNSTPIVYTT	S	300
7343	LOC_Os09g26800	TLHIYDCKDVTISDTTILAPIVGAP	S	281			7344	LOC_Os09g26820	PSFPDLDLADPDSQLPLAAHLIWII	S	435
7346	LOC_Os09g26830	VSAAAARLHHLLSLCVAVAGLLLML	S	16			7347	LOC_Os09g26970	RQARKVCLAELFSAKRIESYEHIRR	S	140
7349	LOC_Os09g27080	PRLHSPRISGGGSMTRRANSFKRGE	S	45			7350	LOC_Os09g27810	DDWRTPLIKFIGSEELPEDDAEAEK	S	563
7352	LOC_Os09g28220	DDNSKAEEDSEGSYDSEDSMDRRKK	S	253			7353	LOC_Os09g28220	SSESDSGESDSLSDSSKSDDTRRKK	S	202
7355	LOC_Os09g29070	ELASTLVIVISKSGGTPETRNGLLE	S	258			7356	LOC_Os09g29070	QDVVSGKIKPPSSPAGRFTQILSIG	S	189
7358	LOC_Os09g29080	LNQIGVELDSENSYTALVYGTSALV	S	105			7359	LOC_Os09g29750	CPPVQLGWDMSMSKMVGGCVLQSSM	S	1031
7361	LOC_Os09g29840	IQGLVKNYSQFVSFPIYTWQEKSRT	S	289			7362	LOC_Os09g29840	NGLIGQFGVGFYSAFLVAEKVVVST	S	209
7364	LOC_Os09g30070	THSRNKDPCPTISSEVPNYTSEPLV	S	394			7365	LOC_Os09g30350	GEIHSGLDPYVQSIAMLRSAEEAIE	S	345
7367	LOC_Os09g31230	DVADDKATEGTASYDKATEGKAADD	S	163			7368	LOC_Os09g31300	LPAASKNSSKPPSSASAAASPEMAA	S	292
7370	LOC_Os09g31950	RRDRRRKKRSAHSDDEGHVLAVEGA	S	433			7371	LOC_Os09g31950	WGAGAGVTLTSPSGDVLRYLVRLDF	S	1369
7373	LOC_Os09g32090	ELGENHSPSLQKSANNPNRGMPSIG	S	65			7374	LOC_Os09g32330	EVLTESSSIGAPSPASSSIGENSGE	S	62
7376	LOC_Os09g32430	TDTEDATIDTDRSSEPTGTGATFKP	S	161			7377	LOC_Os09g32650	ASVVPDKFKSKKSKAASKVGMQKFQ	S	153
7379	LOC_Os09g32740	VRTGKIGGRQQRSTGSKSWFYASDA	S	1018			7380	LOC_Os09g32820	DDVMDSAVGAHFSGLRLEALRLSSP	S	19
7382	LOC_Os09g33470	LQTVNTRQQTERSAKKKKANGAAIA	S	37			7383	LOC_Os09g33470	MFSWLGIAAVAGSINVLLTNPIWVL	S	129
7385	LOC_Os09g33870	GDPYCTVFVGRLSRYTDDETLRKEM	S	73			7386	LOC_Os09g33970	RKEAFSSDKNEYSHLLVSDFSDTTP	S	549
7388	LOC_Os09g34040	NDLLTPFLVVFLSEHLEGNMDTWSM	S	264			7389	LOC_Os09g34070	FKKFGKVDGIAFSRDQTSAYIDFDK	S	156
7391	LOC_Os09g34110	ASAAESLLRCLSSYAEVSSTAEEQN	S	450			7392	LOC_Os09g34180	VGRMNERTAIGSSLRLENAPVLARF	S	761
7394	LOC_Os09g35760	VGAPGEPPGVVLSATTSVRLPGTLP	S	673			7395	LOC_Os09g35890	QTASAGMRLKEGSHINRSLLTLGKV	S	257
7397	LOC_Os09g35920	AAAESKQLAQSQSALPNGDVKVKPE	S	177			7398	LOC_Os09g35920	LLHQLNLNVSSFSSASQLPLLQRLN	S	65
7400	LOC_Os09g36090	LDFVANMKNGDKSVLSQFEVQSLLL	S	752			7401	LOC_Os09g36160	SGGGGGGGGGGISCQDCGNQAKKDC	S	96
7403	LOC_Os09g36320	SSKSKESSGRRGSTTTTVSAVSTSR	S	24			7404	LOC_Os09g36440	IRNSYKDLYPYASRLIPNTYAKEED	S	293
7406	LOC_Os09g36480	SSDGQPGLLQFGSSTSEILETAFKK	S	51			7407	LOC_Os09g36540	PEHTPEAMASLASLGSPPGTALGGL	S	620
7409	LOC_Os09g37040	ILTDTIRYSRGISSIAPDRSSAPPL	S	529			7410	LOC_Os09g37170	FVAEWTPAPEPVSIPEASTDPSQLP	S	1228
7412	LOC_Os09g37230	QQSIHPPPAFGSSTNLEALALEASK	S	176			7413	LOC_Os09g37270	EIIYRYITAEQFSPECLLDCLDLSS	S	323
7415	LOC_Os09g37520	STGDELPHESKLSEASSSGSNQEHD	S	454			7416	LOC_Os09g37720	GGGGGGGGGRGASWQSDDEDSAAAF	S	217
7418	LOC_Os09g38070	RAKRAGGVASLVSPCPVTPAVLPTP	S	96			7419	LOC_Os09g38070	RQPGWKQRKAAASAAAVGARWGGAA	S	61
7421	LOC_Os09g38340	LCPEPSCVHHDPSRALGDLTGIKKH	S	130			7422	LOC_Os09g38340	SGFGGAVDDGKLSTRDFLGVGVVQG	S	495
7424	LOC_Os09g38500	KPSFSTVVAPLRSPMDDRLLRLLRS	S	33			7425	LOC_Os09g38570	KWSDWVDIAHEVSAHCAMENAAATG	S	63
7427	LOC_Os09g39020	IPPPLHQEIPGPSLNKLVLSLSQRA	S	461			7428	LOC_Os09g39020	NHTDMVFSGLGNSNRAYMPDHLNKN	S	410
7430	LOC_Os09g39210	RLCVGCSMPIYPSLPKGDLFSFYET	S	195			7431	LOC_Os09g39462	VFMHTPQQFAMPSRPGVTYVHPQEP	S	421

7433	LOC_Os09g39870	EMEGSVDEDCNRSDEEYDDLAMRDC	S	597			7434	LOC_Os09g39870	GSVSKAWPVESSSGDGKGGSENGSK	S	454
7436	LOC_Os09g40050	FVVEWTPAPEPVSIPEASTDPSQLP	S	1523			7437	LOC_Os10g01320	GVPSGKLLGFLVSGRGIEANPEKIK	S	226
7439	LOC_Os10g02584	PAERDTKQLLSGSPFLHHQHQQHVP	S	43			7440	LOC_Os10g03130	VVGRIAKWVVELSQFDVHFVPRIAI	S	89
7442	LOC_Os10g04580	EELPLAESFPGNSGLLTAAKVARYD	S	215			7443	LOC_Os10g05690	GQVEGVAHSGPTSIVLYFTGATNIL	S	246
7445	LOC_Os10g07110	HPEAFVSGREEPSFAKTEPKPSTSS	S	56			7446	LOC_Os10g07150	AGVDLPTRRLSPSPLAYKRDPRLPP	S	19
7448	LOC_Os10g07270	HYSAYAKLVEEDSWYHFDDSHVSSV	S	878			7449	LOC_Os10g07300	IQRSVYFISEVLSESKTRYTQIQKL	S	138
7451	LOC_Os10g08300	GRRAPHATEGASSSGVSPHHGRGDQ	S	198			7452	LOC_Os10g08300	PEATAGRPRLSPSDPEVVGTEAECA	S	549
7454	LOC_Os10g08410	VSKSREAMPSGPSSGLGPTRGPPAD	S	272			7455	LOC_Os10g08550	TIVSVKARQIFDSRGNPTVEVDVCC	S	50
7457	LOC_Os10g08570	HQRTAPYNIRQISGSPYARERVAPR	S	659			7458	LOC_Os10g10620	ARWHVSNVQTWLSAAITDEGTCTDG	S	160
7460	LOC_Os10g10870	KGRSKWSMMKSWSRRSNGSTGNGNG	S	94			7461	LOC_Os10g10990	EPVDNSPAKPTPSGHARGTPPASKS	S	396
7463	LOC_Os10g12410	NTYQAKKLICPLSLGVQKIHACENH	S	190			7464	LOC_Os10g12460	FTFVYGAEAVLPSELTLRSPRATMY	S	313
7466	LOC_Os10g14230	KRFYDATLIFSGSSYPTANLFFTKF	S	450			7467	LOC_Os10g14290	SPRAVIDFVRRVSPATVGRSIDWDV	S	89
7469	LOC_Os10g16520	QRQQGKQCQENNSSLQLQRGSTLLR	S	451			7470	LOC_Os10g17340	CRSQKITARKSTSRPELVRQSLAWT	S	231
7472	LOC_Os10g17980	RITAVDAVVLPTSPRKILCRFRSIC	S	118			7473	LOC_Os10g18440	NLISATKLASDNSVFVEIHSKYFLI	S	299
7475	LOC_Os10g19200	SSPSSPATPPPPSASATATAPSPPP	S	48			7476	LOC_Os10g20180	GNTFQRTIQGALSDQLGNNVEAYVD	S	43
7478	LOC_Os10g20480	LNAERQASQEVCSQRCEDDILTKAL	S	266			7479	LOC_Os10g20540	GTPTSADECRDISTKDLFCLKYLCK	S	30
7481	LOC_Os10g21130	AADAAAVVIQHPSSSSSSSSSSGNG	S	18			7482	LOC_Os10g21130	IQHPSSSSSSSSSSGNGGGGGGGRV	S	26
7484	LOC_Os10g21920	RRSSALLASLLPSIPHHAFPDLLHH	S	116			7485	LOC_Os10g22070	GDAPGLVDVTLGSVIPRTRANEALT	S	181
7487	LOC_Os10g24120	SPGHNETGVESNSSPLRRKDLEGAK	S	451			7488	LOC_Os10g24690	LVRDFFRLLSLGSEASVPRDGVVCC	S	36
7490	LOC_Os10g25110	VLAERLSCGSTRSGGSDPKNQGCGF	S	288			7491	LOC_Os10g26140	WKLHLDLEAIAASTSDAPLILEFLQ	S	360
7493	LOC_Os10g26540	HKAAAPQPAPNASAAADDHRRPLHA	S	84			7494	LOC_Os10g26670	DKCCAHVGGILRSCSGKRSVHVGGA	S	161
7496	LOC_Os10g28710	QQQQLQRRGGKRSRPISISPSTSPP	S	88			7497	LOC_Os10g28710	RPELWAGPAFSNSPPPSSLPIPKFS	S	114
7499	LOC_Os10g29560	DLLPNKTLRETISRILEAPPTSSTE	S	354			7500	LOC_Os10g29560	FGNDLYVSDSVPSQLGSQAVDASEN	S	137
7502	LOC_Os10g30100	GQASRILQMDRNSLEHLLHHFCGVT	S	376			7503	LOC_Os10g31520	VRLESYVASNEGSRPKKKHKSSKSK	S	383
7505	LOC_Os10g32550	FPPPQEKKRLHSSTSSPPPLTSPPL	S	33			7506	LOC_Os10g32550	QRPLLIVAEDVESEALGTLIINKLR	S	347
7508	LOC_Os10g32960	SGFVFCYSCIFKSVSQHKRCPITLM	S	368			7509	LOC_Os10g33230	ESDQGKLFIGGISWETTEEKLRDHF	S	14
7511	LOC_Os10g33930	VGQALEINYFRGSNYLELGVDIGSS	S	343			7512	LOC_Os10g34350	NAAAEKMRKLAVSHDARLLRPTDDG	S	389
7514	LOC_Os10g34370	REGRSDLGVSAHSQTYVVLFRSLVE	S	338			7515	LOC_Os10g34380	APPSLLGRPMTPSASPFPQTSQSPT	S	343
7517	LOC_Os10g34580	ESCLAGYTFILGSSEKYKFGDKLHA	S	239			7518	LOC_Os10g34660	EHRSKNQDKQEPSREYPRYDGEHDK	S	138
7520	LOC_Os10g34720	ADQHVFCSVLSASGGLKDGWLSKSI	S	234			7521	LOC_Os10g34750	PHEGTELGIGDASIIRALAEAYGRR	S	261
7523	LOC_Os10g35280	QGAEDGKIGSIRSILEAFRKACHYG	S	119			7524	LOC_Os10g35450	VSASPLVGLPEFSHLGWGHWFTLRD	S	169
7526	LOC_Os10g35690	LRRLLPSSPPPPSPASAAAAATFRR	S	22			7527	LOC_Os10g35690	YDYRPDVTFRRGSTYNVKDLDLTRP	S	136
7529	LOC_Os10g36350	CVRPNNGVVRPRSASVVVVTMQAQI	S	64			7530	LOC_Os10g36710	ITIEELAQEMNLSSAAYSIVRDWIR	S	591
7532	LOC_Os10g36810	HSLTLEELVNAKSCWTVRSTSARRE	S	295			7533	LOC_Os10g36810	SEQNSLLLTEVLSKQQKDFIQPSPK	S	888
7535	LOC_Os10g37280	SASGEQSGSGSSSNCFDARLTTEAL	S	112			7536	LOC_Os10g37630	LAAHNWDLQLAVSSITANPSSPDPA	S	40
7538	LOC_Os10g38850	LDDGSRNSSDGASHRIEVSACLSNQ	S	293			7539	LOC_Os10g39200	LGQESSKHEPDASTGTNDLIDNAVM	S	919
7541	LOC_Os10g39620	RTGATGGLGGLGSADLSSLFGGLAG	S	360			7542	LOC_Os10g39670	ASAAPAPAFAEFSLAELREATGGFA	S	65
7544	LOC_Os10g39820	NVSGHLARECPKSDAINERGGPPPF	S	178			7545	LOC_Os10g40110	DDDDIEDDLADDSDSDDGSDYDDLP	S	652
7547	LOC_Os10g40590	DASKVNSRNANQSTGATLLWEKNVP	S	610			7548	LOC_Os10g40590	IGSLPRELEVVNSGDASAIEKPQSE	S	245
7550	LOC_Os10g40780	LQQLIEIARSFASVKDENIRQSRDK	S	1830			7551	LOC_Os10g40800	ERYKGTDQIHTASGEGMSIKHVGHA	S	355
7553	LOC_Os10g41030	SAVSGNVVHVVKSGHCEKVAVSTST	S	514			7554	LOC_Os10g41260	DVVAECSDDQLPSPQSVGTKPPTQD	S	174
7556	LOC_Os10g41290	RLGCGDIGTVYLSELSNVAVGGGGG	S	247			7557	LOC_Os10g41400	NRQGNDVGTQYRSGIYYYTPEQEKA	S	190
7559	LOC_Os10g41770	GGGFTVCFWLYLSSSARPSSVILHQ	S	44			7560	LOC_Os10g41790	LAYQKAYADETGSYLPRSSIPASTL	S	118
7562	LOC_Os10g41960	FSASGNISVSGVSWPIFRWGGGKDD	S	321			7563	LOC_Os10g42090	IDSVPEDQLRFQSYDDTGFNINKVK	S	62
7565	LOC_Os10g42110	AATKGFSADLIVSESGEKAPNVVYR	S	83			7566	LOC_Os10g42210	YRMVCHVMRGDFSRDFFEGCRAILL	S	297
7568	LOC_Os10g42420	GDQASFEKEAQVSLQKFSGSSAISS	S	333			7569	LOC_Os10g42420	NSTEDGNNSWPSSPVAASQPTNQAD	S	140
7571	LOC_Os10g43040	ESLCEANPLALNSRDRLSRTPLHLA	S	57			7572	LOC_Os11g01190	CQPSSDKHHDWCSDIQSSVSPQLND	S	611
7574	LOC_Os11g01200	IDQSLDAGYDLQSVWKIAEVATMCV	S	841			7575	LOC_Os11g01200	ISVGSQDANIMASLVSRYPEAGWAQ	S	378
7577	LOC_Os11g01860	HSGLAPDRYTYISAVKACSISAQWD	S	201			7578	LOC_Os11g01880	KEVDDDTTMEGTSSAPYPLLQVDNG	S	244
7580	LOC_Os11g02190	VLNGPQEASKICSSTGAHVGGGFFS	S	777			7581	LOC_Os11g02620	GQSFLLHKFPLVSRCGRIRRMVAES	S	58
7583	LOC_Os11g02720	LNLIYSVELEDPSGLGLDWIDFFFC	S	120			7584	LOC_Os11g03360	PLLVADSCLRCHSPSVVAVTVDVVV	S	472
7586	LOC_Os11g03890	NQGGFFDSEYRHSPPPRPKKPAEDK	S	190			7587	LOC_Os11g04670	YARSLGCPDVEFSPEDAGRSNREFL	S	235
7589	LOC_Os11g04890	RILCLHTNQSMHSIRLDKKKRVRRE	S	251			7590	LOC_Os11g04930	AAEQKTPDPVPGSEICYFKNGVCQG	S	328
7592	LOC_Os11g05850	TENTEEGELSIDSQEYRSPELDNRK	S	234			7593	LOC_Os11g05850	VNQRASGKAPGSSEEPPTHENHLAP	S	84
7595	LOC_Os11g06040	YTSKSMWTNVPMSILILAFLRYLSF	S	87			7596	LOC_Os11g06170	AGHKRSGSMDGESSLFEGESAPPDY	S	131
7598	LOC_Os11g06440	DAAAAAGGGGVASFLSSHRNRSFGR	S	192			7599	LOC_Os11g06440	RQVREVFHRIVRSRMEGLDSPMLNA	S	406
7601	LOC_Os11g06650	QSSQVSEFKYSPSPSRHNNSSSERV	S	217			7602	LOC_Os11g06680	HYRGPLDDLFLNSESDDSMAESEDS	S	239
7604	LOC_Os11g06750	KKAFTKYALKYDSDAGKKEIQMQLE	S	139			7605	LOC_Os11g07440	LNNIYRYKTEEYSHTAVNKFNVIPD	S	335
7607	LOC_Os11g08390	HIGLAGRSTLVKSVLTSQPVFLLTG	S	213			7608	LOC_Os11g08440	KNAVDDVVLVGGSSRIPKLQSMIHD	S	370
7610	LOC_Os11g08470	TLDNNLLGKFVLSGVLPAPRGVPQI	S	483			7611	LOC_Os11g08550	ERCSGETINKEKSSIMFSRNIKEVN	S	1153
7613	LOC_Os11g08800	VKAGRRARCEAASSLAACVLVLSAS	S	80			7614	LOC_Os11g09160	NQCLQNQPTGMVSLVDRSGNKWSAE	S	64
7616	LOC_Os11g09300	KAPNGVLLKCVSSDDGRHLLLDIHE	S	1238			7617	LOC_Os11g09329	VPTASRQTKPEDSLFKDLVDFAKNK	S	562
7619	LOC_Os11g09660	PRREEALERVEGSATSTSTGDGEGR	S	50			7620	LOC_Os11g10040	GYSLEKRMVPRHSVMKILRTMGLMK	S	331
7622	LOC_Os11g10420	IIHIDFGFMLSNSPGGVNFESAPFK	S	1085			7623	LOC_Os11g10480	VLAEMTNGGVDRSVECTGNINAMIQ	S	268
7625	LOC_Os11g10520	SVECTGNAAAMISAFECVHDGWGVA	S	281			7626	LOC_Os11g11100	HGHGHGHHQRTSSESFIEEQPSWLD	S	60
7628	LOC_Os11g11340	KGPELFSKWVGDSEKAVRSLFAKAR	S	810			7629	LOC_Os11g13600	EPVSIPEASTDPSQLPHTAHWVMQF	S	1394
7631	LOC_Os11g13640	GTILTAIGVDADSHVVPVAFAFVES	S	471			7632	LOC_Os11g14220	AKVQRAVCMISNSTSVVEVFSRIDI	S	381
7634	LOC_Os11g14430	KWDELLALPPQGSEVFIGGLPRDTT	S	104			7635	LOC_Os11g14430	PPMRRGDRRDGGSRGGEGSHGRRYR	S	451
7637	LOC_Os11g16470	YIIVIDDIWSISSWEILKCVLPENN	S	289			7638	LOC_Os11g16510	PRQFMAGALVSASTGVMESLLGKLS	S	43
7640	LOC_Os11g17060	GTGKKKNEPRDHSQCKPECPWSFGQ	S	64			7641	LOC_Os11g17060	MAIDTPSPSPLASAAAAAGRWLGEE	S	17
7643	LOC_Os11g18470	PVRWVGATGQALSAQCSVEQMARVR	S	167			7644	LOC_Os11g19060	AAQNLHNLHHFSSLGYTHNFFQQSD	S	441
7646	LOC_Os11g19130	SEDEEDPLAMIRSMFRYDPSKYAGR	S	488			7647	LOC_Os11g19250	AQVNKPHKTRFASKASRHAHKIDKV	S	18
7649	LOC_Os11g19810	TQLSVANGTKVSSEETESSSFAGLV	S	952			7650	LOC_Os11g20420	RHYFQAHRVTVVSSFPLGEVVRNRD	S	184
7652	LOC_Os11g22180	SGSNVPKKGRKFSSVLGTCRKATNS	S	95			7653	LOC_Os11g24450	DMVKVRIQLGEGSAASVTKKMLANE	S	59
7655	LOC_Os11g26130	KVTELNLADRGLSGTLPDSLSSLTS	S	78			7656	LOC_Os11g26130	TNIDEDDYEGETSEMGLHQQLEKWR	S	877

7658	LOC_Os11g29380	RLINEMVLANKCSLEIDYKQFIYIH	S	280			7659	LOC_Os11g30350	LDYSGSSGVRRKSVVPSVGVGGGGG	S	136
7661	LOC_Os11g30430	GGSELNINIWPFSRSRSAGGGGVGS	S	150			7662	LOC_Os11g30430	SELNINIWPFSRSRSAGGGGVGSSK	S	152
7664	LOC_Os11g31880	ILHEANLAPSVTSIAIFSHATPIRC	S	352			7665	LOC_Os11g31890	CLVLDLDHTLLNSAKFIEVDHIHGE	S	973
7667	LOC_Os11g31960	AAIVTGARSRSNSPAISRNGVDTGA	S	349			7668	LOC_Os11g31960	SGDEKFTEAFDLSKEEEEDVLFKQA	S	686
7670	LOC_Os11g33120	EIITLSASANKLSKVQEQSEEYARL	S	297			7671	LOC_Os11g33330	DEAVVGINPWCISVDTAQRYEHAFS	S	174
7673	LOC_Os11g33380	VIDGVRDKNLIDSGMSRNVYQFSPT	S	57			7674	LOC_Os11g34140	LAAAVDLPGQGMSPPCDAPPPPPPS	S	215
7676	LOC_Os11g34190	GKRVERLKSIIKSCGMSIAPTVYRK	S	335			7677	LOC_Os11g34450	EAISELDSLGEESYKDSTLIMQLLR	S	223
7679	LOC_Os11g34660	PGENISVAAPIASVAPTSLVPIPSV	S	127			7680	LOC_Os11g35050	NTDGFSEVNLNSSLFDEELTATINS	S	504
7682	LOC_Os11g35080	FYLDLHLLEKGCSPRLLDGYDTAAA	S	36			7683	LOC_Os11g35274	AKVADFGLSKSLSGGITRYTENVKG	S	176
7685	LOC_Os11g35660	LCGPPLPKSCYKSDASEQGHLMRSK	S	884			7686	LOC_Os11g35850	NFLTGVIPEGIVSLDGIINLNLSWN	S	752
7688	LOC_Os11g36390	KAVLVMDEVDGMSAGDRGGVADLIA	S	547			7689	LOC_Os11g36480	KKGKGKKDFGTRSAPHDARKTSKRK	S	115
7691	LOC_Os11g37090	TLHLNGLNSQFMSLKSEYDLAMKST	S	398			7692	LOC_Os11g37100	GNIMSTIMCTLSSSGGSFPLHQACS	S	96
7694	LOC_Os11g37990	RNLLSKRKALARSIEIQRREALSHH	S	704			7695	LOC_Os11g38130	YRVTLSCSPSAGSECIVLLLHRPDG	S	188
7697	LOC_Os11g38870	SSGSLGGSSNAASDKAVDGSLDKRS	S	29			7698	LOC_Os11g38870	SSNAASDKAVDGSLDKRSQEKAPKK	S	36
7700	LOC_Os11g39660	IKQQFADFSLFGSGFNSFDSIEDRA	S	578			7701	LOC_Os11g40080	NNTGLGNNLENCSDSSRPETIPVPI	S	882
7703	LOC_Os11g40100	ARAAAAATPATASSGSSLPRPDANE	S	102			7704	LOC_Os11g40120	PPTHPRIVRVSNSPIFSCKIIEISR	S	42
7706	LOC_Os11g40510	ALSKMAANPALMSQHAALKQAAAMG	S	407			7707	LOC_Os11g40510	LIGGRQIKCSWGSKPTPPGTASAPL	S	358
7709	LOC_Os11g41470	AEDTFIALKRYLSNPPVLVAPQLNE	S	229			7710	LOC_Os11g41610	VWNSHPKNYGPGSRVCRVCGNPHGL	S	18
7712	LOC_Os11g42000	EKNGLGSYEVNFSYIEAGEQKQALV	S	150			7713	LOC_Os11g42040	GSFLIRSLTRVISEIINSNDILVLR	S	55
7715	LOC_Os11g42790	DPEIGVGIRRPTSLRLLLASPTQSV	S	491			7716	LOC_Os11g42790	PKSFLDPLILNGSDVDPEIGVGIRR	S	476
7718	LOC_Os11g43230	DADWAGSIDDRKSTGGFAVFLGSNL	S	789			7719	LOC_Os11g43230	LHYFLGIEVTKVSNGILLTQEKYAN	S	656
7721	LOC_Os11g43610	DLMRFDDGIAIVSLESPCVMHFSRA	S	171			7722	LOC_Os11g43970	IAPSVIFIDEVDSMLGRRENPGEHE	S	1005
7724	LOC_Os11g44880	QSSPDIYRMRMGSAPPAFRNPMEEV	S	802			7725	LOC_Os11g44940	GVLFAPDALLGGSGRSGAGVAAARL	S	29
7727	LOC_Os11g46200	LRTLDVSNMWNISELPSQIGELKHL	S	784			7728	LOC_Os11g46240	TTPMPTSASGAFSQPSMQSNQGGQV	S	78
7730	LOC_Os11g46960	VTLKKIRKEYVKSIATMGRDENRTA	S	164			7731	LOC_Os11g47030	HSEVVLHCDLKPSNVLFDEEMTAHV	S	930
7733	LOC_Os11g47770	AAAAEKDSGAVVSDGKRIVVEACTQ	S	76			7734	LOC_Os11g47770	VKEDLESSVPGVSVVINPEKPRRGC	S	111
7736	LOC_Os11g48030	KICQYFYWSLHYSSGKETSEFQIEP	S	97			7737	LOC_Os12g01190	SKADPQSNSFSSSSTVSSTKLPSSW	S	348
7739	LOC_Os12g01260	AAAAAGMRRRRASTAGGSGGFSGGG	S	42			7740	LOC_Os12g01850	AALSPLLLRALSSSALHLHALRLHC	S	51
7742	LOC_Os12g02040	GGAALAHHYYNPSSKTNNSSSLDYD	S	72			7743	LOC_Os12g02130	FDANNQGSKTVQSKAKYFGRMTGGL	S	371
7745	LOC_Os12g02670	HTLDILPPSPAASGPRRPNGSPPAF	S	66			7746	LOC_Os12g02670	NDPLYGCQTPRESIFDPFAPGPEEL	S	375
7748	LOC_Os12g02690	TGKDLIGSNSPRSCETPSSEPLLTG	S	186			7749	LOC_Os12g03710	NQGGFFDSEYRHSPPPPRPKKPAED	S	142
7751	LOC_Os12g04040	SGTSIGGPSRAASSSRRLFPTPSVA	S	589			7752	LOC_Os12g04440	GEDGHVPVICGLSRCNKRDIDAAWE	S	169
7754	LOC_Os12g05420	GESKSYQLGHRLSLKWSTGVGPRIG	S	407			7755	LOC_Os12g06200	DGLLMPSDAPSSSQDIGGMMKIVPS	S	369
7757	LOC_Os12g06560	STNSPLPREETLSDCHKLLKDLLLE	S	764			7758	LOC_Os12g06570	GQGGDDGDGHDDSDSAPLLGAGVGA	S	549
7760	LOC_Os12g06670	STVDKPKLLSQLSALLSDIGLNIRE	S	176			7761	LOC_Os12g06780	LAEEGKRKDRRNSCGLLKEIHVLEK	S	299
7763	LOC_Os12g06850	DGLDDDEGGGMESKILAIRDFLEDP	S	102			7764	LOC_Os12g06850	KQNNSIGYSTTSSSAPVRTVREQKD	S	267
7766	LOC_Os12g06870	PPTPVATGGLFGSGPSPSTPTLQQP	S	555			7767	LOC_Os12g07160	ERMAAVDNELRNSDSWASALVAEFD	S	312
7769	LOC_Os12g07260	WSRGFSSQAGASSGDKQDELEEGFS	S	83			7770	LOC_Os12g07300	DFRQRFRRLLGTSFRHLNFKLAMSV	S	412
7772	LOC_Os12g07530	SPPPPPTPPPSPSNRNHRHPASRVG	S	21			7773	LOC_Os12g07590	DTTRVRLKRSTTSQTSSNDYINASF	S	109
7775	LOC_Os12g09000	LGFDGLKTVCLASKLPVVAIGGINA	S	490			7776	LOC_Os12g09089	IDALRQELVHARSACELEQKGNFQL	S	212
7778	LOC_Os12g09590	VANGLQWVPRSHSSGSEMDNGEDYD	S	214			7779	LOC_Os12g09739	AWNNSLRIPLVNSAAQGSWVLITTR	S	301
7781	LOC_Os12g10070	NAEHLSHQVAIPSIVDEYQFYLGPI	S	27			7782	LOC_Os12g10410	DTWPELEMLAMESNRSYLDPWKVSS	S	559
7784	LOC_Os12g10630	GGEEGSLDALRCSACGCHRNFHRKE	S	111			7785	LOC_Os12g10700	DMIDAGEVQQMRSAPVGQLTSMHDG	S	355
7787	LOC_Os12g11600	ASPPAPPSPPAPSPLAPPPPPPPCP	S	492			7788	LOC_Os12g11750	RRDPVAPPPPRHSKSGRSEAKETAT	S	305
7790	LOC_Os12g13150	KDAKQALGTCVVSFDDMQYVCTRIL	S	908			7791	LOC_Os12g13170	PGAHPFTPYAMASPNGNADPTGTTT	S	112
7793	LOC_Os12g13550	ERRKRYKVGHYISNTTSVTVDPRIS	S	146			7794	LOC_Os12g13550	VLTASTAVASAGSESMNGKSALVKG	S	907
7796	LOC_Os12g13670	EPTRPSVTIVGKSIPKEDGLLKLTH	S	1109			7797	LOC_Os12g14070	EKVVGIDLGTTNSAVAAMEGGKPTV	S	73
7799	LOC_Os12g14984	STSQLVAPQPASSQPASTSTASTTA	S	99			7800	LOC_Os12g15420	GSASDSSSSESESSSDESDKKRSKR	S	772
7802	LOC_Os12g15900	FFLVHGSEAMLPSKLRHQSTRAQKY	S	809			7803	LOC_Os12g16300	EQPPSSLEATGLSPAPPLNLLISIN	S	17
7805	LOC_Os12g16350	MLACGLATHFVPSDRLSLLEESLKK	S	426			7806	LOC_Os12g17310	SLKKDFDPAVYKSLKEKLDETNLEI	S	274
7808	LOC_Os12g18120	AAAVGVEGAAADSRPRFPRRPGEPD	S	101			7809	LOC_Os12g18630	RISAFFYKAAELSLLGVVAGSIQGG	S	266
7811	LOC_Os12g18760	STEEYDANIVSCSKFFPAKRSLSLT	S	249			7812	LOC_Os12g18860	DKLKFSFDHMEKSRRSIDIFERPKK	S	344
7814	LOC_Os12g21700	HPGILLDGGSGRSHRVPQSDSQQIP	S	431			7815	LOC_Os12g21870	DIIEGVQFARMSSIPSCDYTPFPRN	S	596
7817	LOC_Os12g21890	TTAPPPPPAAADSSMSEFKAIAADA	S	632			7818	LOC_Os12g22090	ATSASHGTAVPISRQVPWVKLVKDV	S	374
7820	LOC_Os12g22620	PGHSRACEACGNSRPMEVVAVDDDD	S	151			7821	LOC_Os12g23790	SCASTELAGNPTSVDSAVDHITEPT	S	298
7823	LOC_Os12g24020	GGGGGGGGRGDCSKQEETERVMRSV	S	19			7824	LOC_Os12g24080	KKDPDHPAEKDHSDALSQISEINTA	S	460
7826	LOC_Os12g24160	QTDWGGEYRALNSFFSKIGIVHHVS	S	463			7827	LOC_Os12g24580	QAANGSARAAIESLKPKLKVKLECS	S	64
7829	LOC_Os12g25700	THNSKGINYQILSNPEFLAEGTAIE	S	154			7830	LOC_Os12g25700	YQKSRFVNRVVASMFNTVSGKKIAV	S	317
7832	LOC_Os12g28260	EEEEEEAASASSSCQITTTVLVSRF	S	693			7833	LOC_Os12g30020	EETGELGASWYFSRKEIEENSPSRR	S	39
7835	LOC_Os12g31040	RCLHSVKVPTGFSANVRKLVSLKDL	S	841			7836	LOC_Os12g31050	LTGQFTLTTPSPSVPNTAAPPAPLS	S	440
7838	LOC_Os12g31350	RGADHQNAGGATSEPSESHRSTGTE	S	162			7839	LOC_Os12g31840	GFDFQPAQESEESEFSDAAEELADE	S	378
7841	LOC_Os12g32374	YQDTGHCLALSFSDLSIWCFACDSY	S	128			7842	LOC_Os12g32440	MLKTAEESIKKNSNHVMVMHKRKPN	S	284
7844	LOC_Os12g32986	SNLIGQFGVGFYSAFLVSDKVAVST	S	234			7845	LOC_Os12g33090	LVDPNRASASSLSTPLHLAAAGGSA	S	90
7847	LOC_Os12g34090	PVVFFAPPTSVRSSELQQIGLSEIP	S	807			7848	LOC_Os12g34330	SGTRSRSFGQKQSVNHLTENQGNAS	S	517
7850	LOC_Os12g34370	IWILTPHDAVAFSRRFAVVEKRWRV	S	23			7851	LOC_Os12g34370	MTEHSIEWRIVSSGRGLSGRSIEAT	S	476
7853	LOC_Os12g34510	GGGGRGKSKGSKSVSRSSKAGLQFP	S	18			7854	LOC_Os12g34630	ADLHAVQRRDDESLRSYIQRFCQVR	S	333
7856	LOC_Os12g35030	IDDYKLKQLARASSSSSKADKSRRD	S	29			7857	LOC_Os12g35100	VSTQSKVEITTDSQLLDWLDKGNQH	S	85
7859	LOC_Os12g35620	LQDATDNDGNKGSGEENASEGVSDT	S	141			7860	LOC_Os12g36170	AASHDAAAAANPSSGSGSGSGSGKG	S	155
7862	LOC_Os12g36510	KYLGLPTPEGRMSADRFKSISERLA	S	203			7863	LOC_Os12g36690	INSSMLSRLTALSILLKSEYLQPDH	S	787
7865	LOC_Os12g37430	SRLDNSLTRKRRSSSTARRRPRPES	S	25			7866	LOC_Os12g37840	VTRSRGEIKRLNSPKITSSGGTPVA	S	647
7868	LOC_Os12g37870	ESFIHELDEKTSSSLKFTVLNPKGR	S	259			7869	LOC_Os12g38440	EEVGEASYELYSSWQKQLSDLSWNP	S	271
7871	LOC_Os12g38870	KSWIAGLHPQHVSTVNVLNKAVEES	S	240			7872	LOC_Os12g38870	VGNSEGSSHMEKSWIAGLHPQHVST	S	229
7874	LOC_Os12g38970	RPPRRQENEYSESEREESEYETEGE	S	568			7875	LOC_Os12g39160	VVRNRMAARATESGRFVLWQMAPEM	S	206
7877	LOC_Os12g39650	SVYEARETKPKFSHLSASLCPLPVP	S	445			7878	LOC_Os12g39980	LEAQILKSNQKVSALEEQLASKINE	S	2148
7880	LOC_Os12g40010	GSQLPAFSGVYTSAQVREAFESDPG	S	457			7881	LOC_Os12g40510	EDPQVLARETVFSVSEVEALYELFK	S	42

7883	LOC_Os12g40570	HCSKRRKLRIKRSIKVPAISNKIAD	S	281			7884	LOC_Os12g40790	ASSIQKYIMQKLSLPSETEVEISCC	S	333
7886	LOC_Os12g41000	VLIDGGAALNILSPAAFDAIKAPGM	S	508			7887	LOC_Os12g41200	AISTDILGVVFKSLHDERTSQLQSL	S	927
7889	LOC_Os12g41956	PEEPIQVFIGIISATNHFAERMAIR	S	419			7890	LOC_Os12g42020	TAPNSSLSSAASSTGSLARCSSLSR	S	43
7892	LOC_Os12g42260	EAYKLHERVQLDSICSNELGDPDVI	S	530			7893	LOC_Os12g42310	ENLQRPITMEAGSVVLMDLLWSDPT	S	856
7895	LOC_Os12g42370	YFKAHDILYVFWSAKAATATLEGKI	S	239			7896	LOC_Os12g42520	GLQHLDMSQNNLSGPIPDFLATFQY	S	612
7898	LOC_Os12g42600	APIVVFRNQRMESTLTSFGRKSWKS	S	564			7899	LOC_Os12g42790	QSGRRDRRRKKRSAHSDDEGHVLAV	S	430
7901	LOC_Os12g43500	DDGKCVTKEDLESDEAVWALFERYC	S	98			7902	LOC_Os12g43640	MNHLTGSLSELRSLTQLVSLQLFYN	S	289
7904	LOC_Os12g44130	RRTGLDVFHRIRSMKAVHAFLSLYT	S	311			7905	LOC_Os12g44140	DEAGNEHYPPDTSSEEQRLSAIRRI	S	33
7907	LOC_Os12g44330	GKQEVSILEKLASADREDRRHCVRF	S	666			7908	LOC_Os12g44330	RMILNIKPKDIGSLISNFPGEDPKM	S	881
7910	LOC_Os12g44360	GQRRQRHRHTKASSNKAHSSSYPRV	S	1047			7911	LOC_Osm1g00310	TKYAMPALLLTTSISGNGKPEMTIL	S	66
7913	LOC_Osm1g00420	SRYSSGRGLASISTRREYGLSPRQQ	S	94			7914	LOC_Osm1g00420	YVSRRTRVEVNSSAELSAACFQVSS	S	61
7916	LOC_Osm1g00500	EERFFIDLVAGESLIKERAAARFND	S	63			7917	LOC_Osm1g00500	NKVGSKNVVAGESLIKKRIEERFFI	S	44
7919	LOC_Osp1g00800	ALYDKLPWIWEVSLSRYWDRLIDFL	S	95			7920	LOC_Osp1g00800	YDKLPWIWEVSLSRYWDRLIDFLDR	S	97
4969	LOC_Os01g01880	LTFSPRPKNHPPSCRSSSWAERSPI	S	114			7922	LOC_Os01g01510	DAHEELMAIGPVTNEVTAGQSKDKG	T	246
4972	LOC_Os01g02720	QHPTKEKESSKKSSSMVPVLWRCSM	S	51			7925	LOC_Os01g01960	RIDTNGVIIRVKTLFNGYPELILGF	T	20
4975	LOC_Os01g03500	GLPARCLGDPSSSPSALAIARCRNV	S	29			7928	LOC_Os01g03760	TGMFCWAHFLFPTLCAKPSGNPQTR	T	329
4978	LOC_Os01g03840	QTVESSTTTAASSAEASRVTKKKSN	S	36			7931	LOC_Os01g04330	FDVYDVDGDGRITAAELGKVLGRIG	T	121
4981	LOC_Os01g04160	SNAELLLGNSPLSTKTSNLSLHCFI	S	742			7934	LOC_Os01g05010	LPLLRASASAAGTTRGAAALLRPLA	T	27
4984	LOC_Os01g04720	WLHFAEIDAGIGSAGQRVFDVMLAG	S	310			7937	LOC_Os01g07110	NSSVIAVQSMFHTGLVRPDPVSRSS	T	209
4987	LOC_Os01g05820	YVNITGNVSEMLSPAFADKLRQRIV	S	388			7940	LOC_Os01g07880	AQQARERKKAYMTELEAKAKDLELR	T	136
4990	LOC_Os01g07110	MRHVWVAEATESSTYSLSHEIPKKS	S	1440			7943	LOC_Os01g08970	LEKGFFFLPKPPTLILHEEIEFVEF	T	376
4993	LOC_Os01g07520	AASASAAPASWGSLDSGSKAASASY	S	92			7946	LOC_Os01g09550	ICYTHPEKLPGVTRDGLSKHFFHRP	T	153
4996	LOC_Os01g08410	GGDKAKGGSSSSSGRKREQQGAQPS	S	46			7949	LOC_Os01g09850	CDALAEESARAVTAAAAVAGQQQHG	T	211
4999	LOC_Os01g08430	GTAGCINHSGSASSKLNTAKRTVSL	S	845			7952	LOC_Os01g10350	PFPSQHVAPSSFTTRNALAPLPSNS	T	280
5002	LOC_Os01g09280	MKKSASMSCIASSLGSGGGSGGSSP	S	57			7955	LOC_Os01g11330	IKGGGSISGVQGTHFRGRMGLSGAV	T	139
5005	LOC_Os01g09790	VSKRVCAKPPVVSHHQRIKAQSISS	S	346			7958	LOC_Os01g12360	LGAPDPQEGPEATVGRPHLSPFDPE	T	1204
5008	LOC_Os01g10690	RVISYKWLSRNFSVSSNDSKRLLQE	S	38			7961	LOC_Os01g12590	RRAAAVARHSAATPSSAPPTQEDLR	T	159
5011	LOC_Os01g11580	NAGRSSSSGSSSSDSGSSSSAYADT	S	325			7964	LOC_Os01g12780	WIVYDLDAKELRTWYEERLLRDRGM	T	19
5014	LOC_Os01g12280	LRHRPKLKQMFHSSDYASSSYANRS	S	388			7967	LOC_Os01g13680	DRTTFVFWDAYHTSDATNQVIADRL	T	19
5017	LOC_Os01g12480	TGEWHNFMGSSLSDMYRKPAAEKSD	S	53			7970	LOC_Os01g14920	SPKRDRLRSPVRTTSDIRPRHTDSR	T	202
5020	LOC_Os01g12760	RRSEFGTSSLDMSEMNGLSVRLKYG	S	517			7973	LOC_Os01g15020	TVACTLSQGSSVTSMDFHPTRHTLL	T	343
5023	LOC_Os01g12880	LSWDDNPSTTENSLALVPVTDPVVD	S	360			7976	LOC_Os01g15600	GGVGGDVELVSKTLQFEHKLFYFDL	T	41
5026	LOC_Os01g13160	REERLLDTIATSSGVRLQPTRDALQ	S	282			7979	LOC_Os01g16390	SFQGGAPQLERLTLAVLKKPEDGIF	T	932
5029	LOC_Os01g13530	EKSSSKGRGFLKSLLTRRRWRNDES	S	297			7982	LOC_Os01g16500	HLLEDYGSSSSSTPRDMFAIGGTST	T	230
5032	LOC_Os01g14010	LTSATAAAKEAESVPSVFSNQQHAK	S	355			7985	LOC_Os01g18890	FTSDATLVSQSDTAALVDLYARNRK	T	191
5035	LOC_Os01g14050	LVIDVEKFVSTPSDNDWFSIEERPP	S	271			7988	LOC_Os01g19940	LQQGASNSTKGTTEPLKPKLQRTAY	T	105
5038	LOC_Os01g15260	AGGGGGGAALRASSDVDRKPAAAGS	S	22			7991	LOC_Os01g23530	MRNYIPIYEIEATRNETILEFAKLN	T	226
5041	LOC_Os01g16030	GFNVETVEYKNISFTVWDVGGQDKI	S	62			7994	LOC_Os01g23930	SYELVIVTTVQPTIQEWEHAADNLA	T	1227
5044	LOC_Os01g16240	LNLMARKMKDTDSEEELKEAFRVFD	S	82			7997	LOC_Os01g25370	VHESSNIVITRETLQCLNETEWLND	T	303
5047	LOC_Os01g16430	MRVAATTRPASSSAAAPLPLFLLLA	S	13			8000	LOC_Os01g26970	VPYVIFCAPPYRTDDYPGDLRVAAS	T	163
5050	LOC_Os01g16870	KPDQVIIFRDGVSESQFTQVLNIEL	S	725			8003	LOC_Os01g28560	QKRKLNSDEITSTSNSKTKVQKTGP	T	213
5053	LOC_Os01g19150	ELLLGQPVFPGDSGVDQLVEIIKVL	S	277			8006	LOC_Os01g31360	QQQFGQDGSGSDTMVPQAYSPKSKN	T	578
5056	LOC_Os01g19760	EEGSNASINSEKSGQQLLSVDAGPR	S	339			8009	LOC_Os01g32720	PQPDPCLLKDASTGITKPAKEQGKL	T	208
5059	LOC_Os01g21590	VAAVRQQQQQSYSPYSAGASSRVIK	S	58			8012	LOC_Os01g33370	SSQTYKCEICSRTLLDDFRFCSLGC	T	131
5062	LOC_Os01g23930	TPTPEYTRGSAGSGVPTSSTAPPRP	S	1408			8015	LOC_Os01g34780	EDSQEYKALDSETYSRELFEECVVH	T	265
5065	LOC_Os01g26940	MENDAAEQNLHDSYSEAQTTSLGYG	S	114			8018	LOC_Os01g37080	FMPESATGREIVTLGEGRPAPDYPG	T	42
5068	LOC_Os01g27990	TAIVKLEVGDDGSGDVHFEAFQMSE	S	265			8021	LOC_Os01g39780	ANIFIGVYVFAKTYKRDQEKKNAQT	T	46
5071	LOC_Os01g31580	DFAQGETPDLVASHAHLAAPTQAAA	S	66			8024	LOC_Os01g40070	RQRVAVCIWRLATGEPLRLVSKRFG	T	273
5074	LOC_Os01g32660	SGSSYDYKSDIWSLGLVILECAIGR	S	253			8027	LOC_Os01g40370	PGNGGYQRTTDATDFAKEAAAHAHG	T	31
5077	LOC_Os01g32964	TKDHPNKLTKQLSAAGMVEELFEEG	S	242			8030	LOC_Os01g41090	KRHAIEDDLHVLTQNPGESLRDYVR	T	527
5080	LOC_Os01g33090	LPYLQILDLSSNSLNGTLPPAILRC	S	153			8033	LOC_Os01g41430	MAALFAARGVRCTILTTPVNAAVVR	T	41
5083	LOC_Os01g33490	LPLTALLPVAAASRALRRLLRSRAF	S	34			8036	LOC_Os01g41880	EEEEKEPEDKEMTLEEYEKVLEEKR	T	243
5086	LOC_Os01g34290	PYEMQRQDIKVPSGYSARISKYVKL	S	365			8039	LOC_Os01g42350	TAVISLLQPAPETYDLFDDIILLSD	T	413
5089	LOC_Os01g35470	RIAQLQDSNFELSGSSKAQATRMAQ	S	672			8042	LOC_Os01g42850	GMDFVMQVINEPTYLEDLTGLTDLM	T	1008
5092	LOC_Os01g36790	AVFGLGQCYRDLSPVDCKLCFAEVR	S	90			8045	LOC_Os01g43440	AGVADCDSVRSRTTQHLGGRLRNTG	T	444
5095	LOC_Os01g36930	NHLHEGLKHGLKSELEKASPSLGRT	S	276			8048	LOC_Os01g46070	LVSSLSLYDIAGTPGVAADVSHINA	T	64
5098	LOC_Os01g37080	VEVGRKAHRRHVSELETRKKVLAEI	S	656			8051	LOC_Os01g48180	KGLPSMLSLDSDTLQQLDSFRGMLT	T	309
5101	LOC_Os01g37920	MARALDPAAAITSIRWLVVGGGGEK	S	62			8054	LOC_Os01g48720	HNALEGDAELDSTIAENEVLWRANF	T	250
5104	LOC_Os01g39110	HHHHHHHHQLLFSPPPVMAHHQELA	S	256			8057	LOC_Os01g49350	LFNRSTFGTKCKTCLNLVISRIKLL	T	19
5107	LOC_Os01g40070	ATARSKRSRLAASATTAAVVATEEG	S	163			8060	LOC_Os01g49890	AERLGREHLGGMTDLWVKHCGISHT	T	187
5110	LOC_Os01g40390	IAELLDIGHLTFSGYGLTAIGLIII	S	111			8063	LOC_Os01g51080	MLEKIASKEPQTTAELFQLADRVAR	T	359
5113	LOC_Os01g40590	HSVQPMASGSTTSSNTGSISPSSIV	S	103			8066	LOC_Os01g51890	PLDILAQRLPDSTKPFISEKALRSC	T	365
5116	LOC_Os01g40810	LPAVLWANRTTPSRATGETPFFLVY	S	1766			8069	LOC_Os01g53370	EALIFPEGFLRRTKGRGLVVMSWAP	T	354
5119	LOC_Os01g41190	PYIALDPAFYSRSSGQTSSSVPSRE	S	642			8072	LOC_Os01g53670	GSEDKGPGATMGTAPGLVSSSFPSK	T	605
5122	LOC_Os01g41610	RLAQLRSKAAQASELVSKHGCAYYK	S	16			8075	LOC_Os01g53680	STVKQYFVNADDTVPEKVVVQKDSP	T	129
5125	LOC_Os01g41640	SAAPDPDLTNFRSLFSRVLNTYPDP	S	50			8078	LOC_Os01g54530	GVLEALLWRTNSTFAISEHGARSNI	T	332
5128	LOC_Os01g42260	VDQTTKKRKKPGSSSGRANSSGTAN	S	470			8081	LOC_Os01g55050	QHPPVQNQMRPQTPPNYPHYQPHQS	T	406
5131	LOC_Os01g42370	SAVLFIGVLNSQSVQPVVSVERTVF	S	1244			8084	LOC_Os01g55650	ENGILFNVVKRDTLLTPTGGVELQG	T	785
5134	LOC_Os01g42700	DGIGVSEGEEDISERALHSPSITMV	S	230			8087	LOC_Os01g56140	NLEKALHDMRGETAETKVSYESKLA	T	144
5137	LOC_Os01g42830	TAEEMVQHYEKSSSVDYSLKEGETL	S	161			8090	LOC_Os01g56800	ITEDVDDLPSSTTSSDRNTSGCGPA	T	625
5140	LOC_Os01g42960	RARASVATSSSSSSGSAKSPAPSNA	S	58			8093	LOC_Os01g58400	MLAWGKNGTRSQTRHGSVGGSNGRM	T	353
5143	LOC_Os01g43170	MPGQMNHQPPPGSFMQYPGDGGRPV	S	462			8096	LOC_Os01g59250	HIDLHHTVFSGHTVVGNHSAVAGAA	T	131
5146	LOC_Os01g43410	RGHIDFSSEPWPSISNGAKDLVKKM	S	292			8099	LOC_Os01g61210	GNTWEETRVFVDTIVRCNLQSLART	T	178
5149	LOC_Os01g43774	PALTKLLADGLTSHDGEKWVKHRRI	S	276			8102	LOC_Os01g61660	KANGKLRMCIDYTDLNKACPKDPYP	T	885
5152	LOC_Os01g44310	YEETALVNKNGDSSAVNLTHCDHNE	S	525			8105	LOC_Os01g62030	AVVGGTGRYDGATGFAVVRAADAHK	T	133

5155	LOC_Os01g46710	PPVTETSLGKEDSPAVPSSPEVQEI	S	1272			8108	LOC_Os01g62500	EPSFYSQRLAALTPGFAGADIANVC	T	524
5158	LOC_Os01g48180	RLLKLQLSTIEASIPPEAIQPFWTD	S	1212			8111	LOC_Os01g62890	LRKPVSILAAFSTGLSIAFLNDSFA	T	83
5161	LOC_Os01g48320	PLPNYRQASNLPSAGLLFNKMAAAA	S	170			8114	LOC_Os01g63770	IAALLNGQAEGITHTGPTKLVLYFT	T	233
5164	LOC_Os01g48610	GAQPAIEAAQAISRVDTERIMLVTS	S	603			8117	LOC_Os01g63810	RNRILHVADTSNTLIVCEDWDEAKN	T	209
5167	LOC_Os01g49020	KAAVIASSQCFCSKPSITLYLFVFK	S	64			8120	LOC_Os01g64650	RDLDETKIILHKTIESVLQRGERLD	T	174
5170	LOC_Os01g49470	KLFTCRFCHDKISDHTMERKATQEM	S	1018			8123	LOC_Os01g65480	GVVGGGGAHIARTRSDDFGARMAPG	T	100
5173	LOC_Os01g50310	TYPNGNVEEDEVSVTAAVNPAKSYA	S	125			8126	LOC_Os01g66520	GVGKESDIFEVATEDGTVLAMKLHR	T	112
5176	LOC_Os01g50450	SGGVLVKDVVEVSRSLGAADTTEVA	S	217			8129	LOC_Os01g67250	NDLGQVTAEKGTTESDGSNKVGSLT	T	522
5179	LOC_Os01g51010	LGQSPPYSPHIHSDEYDMPTDPHRS	S	469			8132	LOC_Os01g67690	AWRNIEALYSTGTRARAVNTRLALT	T	124
5182	LOC_Os01g51200	KQKAPVGNDASFSKEPVISASNFLG	S	370			8135	LOC_Os01g68750	SSSTEKVATANATPQPAKEQVIPSA	T	822
5185	LOC_Os01g51230	SSSQRGGGNASASEVYSETSSSADA	S	53			8138	LOC_Os01g68920	REEVLERVEGSATSTSTGDGERRLL	T	52
5188	LOC_Os01g51620	ESGECFMSDKKKSAKEWQEKLEKQS	S	322			8141	LOC_Os01g69130	PGLKSRISSQLTTTAKELSFYGDPV	T	333
5191	LOC_Os01g51640	RLSCVGVTEGCNSASRMIRSRSMDA	S	508			8144	LOC_Os01g70020	SSKESDVTVDKTTKKASKSKKDEGK	T	368
5194	LOC_Os01g52304	LHSLRGDHITSVSKILIITSRSRKIA	S	163			8147	LOC_Os01g70330	DRKIMSSGQKAFTGSIIEHDDNIST	T	366
5197	LOC_Os01g52390	AAAPAGKAAQAASAAAKFPTKPAPP	S	53			8150	LOC_Os01g71050	VILPRSTCDFTVTMQAQRTAPPDMQ	T	69
5200	LOC_Os01g53520	HLLTLVCHCLILSLAVLFLWSNAST	S	112			8153	LOC_Os01g72270	KRMHYLHAAITETMRLYPPVPLASR	T	396
5203	LOC_Os01g53700	FGIHATTSPNFASKAVDLAAGELLE	S	386			8156	LOC_Os01g72690	LLTEDSYYNFIQTWDDDEEKKMLHT	T	313
5206	LOC_Os01g53980	VEMDTCHRRSRSSRIAIRHSRDHSS	S	210			8159	LOC_Os01g73620	PGQRLEERAKSATTSKRVRSSLAAD	T	169
5209	LOC_Os01g54560	SSSASPSPSSSSSSSSSPSSSSGSG	S	196			8162	LOC_Os01g74230	RHSAATPSSTPPTREDLRGEPDARI	T	166
5212	LOC_Os01g54920	IEESEEEESEDESEGKAKHKGTEGL	S	61			8165	LOC_Os02g01380	DEEDVGVGVGRATDDHDDQVDESHD	T	356
5215	LOC_Os01g55260	TECKTTFFNISASSIVSKWRGDSEK	S	169			8168	LOC_Os02g02360	VGNVGDVEETGGTDACIEELDNLDI	T	356
5218	LOC_Os01g55410	GGKRIDLGAPLRSVRHADALPYHKA	S	22			8171	LOC_Os02g03060	DGAGEHPSTSAMTMPFAVSDPSASV	T	54
5221	LOC_Os01g55560	FDGALRELKDLRSQLHQTADCCEKA	S	50			8174	LOC_Os02g04490	NGAGYFNGEPTVTSQMQQQKQFPSN	T	248
5224	LOC_Os01g55730	VRQAYHNVCTACSKDLGICAKCCTC	S	88			8177	LOC_Os02g05310	LIDGILYAFQEQTSDDANVMLNGFG	T	882
5227	LOC_Os01g56000	ENILDACFALIASAEDVNGEGLYFL	S	285			8180	LOC_Os02g05510	SKSKAEEATSVITSSEGSPNWGAVE	T	215
5230	LOC_Os01g56130	TMSLLNILPAPKSFPFIVPYLLFEN	S	551			8183	LOC_Os02g06584	SVNEDLNERSPNTAATKKRSIPYST	T	270
5233	LOC_Os01g56790	CHQSDHSIHERYSVLQMTSGSEDEA	S	203			8186	LOC_Os02g07070	APTEMIDSDIKPTPVSNSLGTTIMG	T	757
5236	LOC_Os01g57280	ETEWDQLLDPLVSQKEGSRVLVTSR	S	362			8189	LOC_Os02g07260	KKLADLTTTKGATTIIGGGDSVAAV	T	353
5239	LOC_Os01g57790	AEKAFIALKRYLSNPPVLVAPQPNE	S	203			8192	LOC_Os02g07940	LADRVARKEEAWTWNPSGSGVAASA	T	399
5242	LOC_Os01g58550	TLSGVQVGADWTSILKRRHVYREAF	S	251			8195	LOC_Os02g08360	DSKEGDTLQRWRTEPFIMITGSGRW	T	404
5245	LOC_Os01g59670	FHQGSRVGNDLSSNQDINSKGLLSI	S	209			8198	LOC_Os02g09740	VATMKISPAEKMTPERIYGKTGSMR	T	373
5248	LOC_Os01g60040	GRPGDARAALRRSVRENGSDIAEQS	S	1418			8201	LOC_Os02g10020	LARTRSNNTAWQTLGSQADRNKENN	T	301
5251	LOC_Os01g60700	FKAINASGPLDASGPLPLPPKKCDG	S	142			8204	LOC_Os02g11060	PREIDKSPGSVRTVAWLHSDQSILS	T	150
5254	LOC_Os01g61430	QKTIPLEEVSKSSLQEEISRNSRSL	S	52			8207	LOC_Os02g11840	SSRLNYCVGGEVTTLFHLDRQMYLL	T	258
5257	LOC_Os01g61780	GACVLILREKKLSSQKLGSFMEMAF	S	452			8210	LOC_Os02g12540	GFKIPDLFPTWRTVLAAVTGMRRAL	T	231
5260	LOC_Os01g61814	LGNEWKKPFAGSSHAKGIVLEKIGI	S	44			8213	LOC_Os02g13330	ERAAGPGRRPTCTSLVAQRVDAPLA	T	48
5263	LOC_Os01g61860	LLQSLAGSTFDSSQLVLTACMGYQA	S	456			8216	LOC_Os02g13990	GPKVVAPTPEQITAIKAAIVNSQTL	T	208
5266	LOC_Os01g62030	PVFAGHGPIGHHSGGWLRVLTRPGA	S	34			8219	LOC_Os02g14780	LSVRTSFLKDNLTLAYYNIGPGVVI	T	768
5269	LOC_Os01g62810	RGTDDSTKRRRLSSTKHAPESSTSS	S	223			8222	LOC_Os02g15310	PVRDVYLPKDYYTGEPRGFAFVEFV	T	74
5272	LOC_Os01g63940	QQQQQQNQPQHSSQQSQQTTTLRNQ	S	108			8225	LOC_Os02g17320	IRGMHTIIRDAATTTHDFIFYADRL	T	284
5275	LOC_Os01g64000	PAQGAMVVVSPGSSDGMSAMTHADM	S	261			8228	LOC_Os02g17870	NEDTTDWNAPWATNGPLRAKYNAKM	T	398
5278	LOC_Os01g64450	MRVRLKVMVFVDSVEYRPDVDCYIR	S	204			8231	LOC_Os02g18430	FGVFLYELLHGTTPFKGSGNRATLF	T	369
5281	LOC_Os01g64720	LGTVDSYGHLIVSRLDTVADDIDRA	S	121			8234	LOC_Os02g19220	DVEQTSESSDDVTEKSASKTLDQEP	T	515
5284	LOC_Os01g65330	FGSGTTTKGNKISILAFEVANTIVK	S	162			8237	LOC_Os02g21150	FRPGAIEKYDGSTDPEEFFQVYSMV	T	486
5287	LOC_Os01g65560	EALVKTRVEDGFSLKYIPWHIVNKI	S	91			8240	LOC_Os02g22210	NPADMMTKPVPTTKFELCSSLVGVT	T	1118
5290	LOC_Os01g66140	VGWGSKELIEFLSCIGKDTTKPLDE	S	240			8243	LOC_Os02g23970	SLGATWARFSLLTKSGPDLSLPNHM	T	179
5293	LOC_Os01g67160	RAPEVLLGSTHYSTGVDIWSVGCIF	S	191			8246	LOC_Os02g27230	RSYIQRFCQVRNTIPCIPAHVVIYA	T	341
5296	LOC_Os01g67580	RVKWDIYRKFVNSAYGGALVPVILA	S	823			8249	LOC_Os02g28980	KLKDYKEAKELCTEVLELDSMNVKA	T	529
5299	LOC_Os01g67690	WVQTVLKELGIESPKAAKIWCDNLG	S	1367			8252	LOC_Os02g30050	KAELSLLRVAKVTGGAPNKLSKIKV	T	37
5302	LOC_Os01g67860	TVPAAVPAIVFLSGGQSEEEATVNL	S	266			8255	LOC_Os02g31220	LPSDSSAAETTVTLAKTLSSCVRHM	T	642
5305	LOC_Os01g68370	NLRFLLQKVLKQSDVGSLGRIVLPK	S	544			8258	LOC_Os02g32490	NPSEVCSENLDVTKGPVQISWFKGG	T	112
5308	LOC_Os01g68750	KTQAGRKTAKESSSTEKVATANATP	S	811			8261	LOC_Os02g33960	NRTLAELHGMLKTAEESIKKNSNHV	T	107
5311	LOC_Os01g68970	TSACSATSKGSKSWWMPNSVSNFSS	S	342			8264	LOC_Os02g34570	VAMKESSDGALETQSTKPSSDGQQI	T	315
5314	LOC_Os01g69130	AKELSFYGDPVESKAGQGAKLLNIL	S	347			8267	LOC_Os02g35750	TVSYNIVLKGLCTAERWEDAEELMG	T	275
5317	LOC_Os01g69910	EPGTCRNLADVFSGLEFSKENHPEE	S	260			8270	LOC_Os02g37420	LTIGVEFGARMITIDNKPIKLQIWD	T	49
5320	LOC_Os01g70020	ENDDEDDEGPADSEDASMGEEDDED	S	266			8273	LOC_Os02g37880	EIQELKRTLAETTPPSSKVSSRLPD	T	534
5323	LOC_Os01g71230	GGDASGRSKQSRSEKKSRKAMQKLG	S	63			8276	LOC_Os02g38920	VKVKDSKTLIFGTKEVAVFGCRNPE	T	73
5326	LOC_Os01g71590	VDMIKLLFCHGASANLHTVNDAGIP	S	194			8279	LOC_Os02g39340	GRLLSHPQYSLTTHSVRFPKLQKQV	T	37
5329	LOC_Os01g72080	GVKRAISRSEASSFASASSESESSS	S	52			8282	LOC_Os02g39890	GRSGGQVRDEYRTDYDPGRGGYGKM	T	134
5332	LOC_Os01g72710	LRNGAKLFTVGTSASLIGTGVTNAL	S	280			8285	LOC_Os02g41800	GEEHEKPASFAKTLTQSDANNGGGF	T	123
5335	LOC_Os01g72890	RTPPRHRRGRSISSDSRSSSSPSPR	S	418			8288	LOC_Os02g42540	LLKNIVGIKPKRTKVSSPSQPAEND	T	135
5338	LOC_Os01g72980	DRCYLQKRVLNSSKGPAVRALRAQT	S	148			8291	LOC_Os02g43020	LKDAEKCIELDPTFSKGYTRKGAIQ	T	455
5341	LOC_Os01g73160	VPNTLKKSAKPPSRPFGSGPPGDRP	S	99			8294	LOC_Os02g44410	PSQPIIGVTPGHTWPLGHIDLPVTF	T	614
5344	LOC_Os01g73230	RSVEKGKSNSTFSAHQDMGTSRSNH	S	112			8297	LOC_Os02g44810	AVMASVGSGSELTKCLECSERGRKV	T	195
5347	LOC_Os01g73480	KRASAPATVTGSSKEEEEQMTTTAA	S	847			8300	LOC_Os02g46040	QRISKRYVLVEGTLYRRAANGILLK	T	1610
5350	LOC_Os01g73620	EMAYKKLMGGRGSSSHKGGLNKAAN	S	1001			8303	LOC_Os02g47130	EEKLPADVTDDKTSEEDDLNEVEKL	T	372
5353	LOC_Os01g74020	VPSPYHPHPHHHSYNNAAYAATVSS	S	217			8306	LOC_Os02g47410	GMPWGGRPGHSFTAGGLPSSFAGKD	T	929
5356	LOC_Os01g74200	TKISSDAISRNASGSRRRRSGSRRK	S	19			8309	LOC_Os02g47970	VHRPKPIKVINATHEQFEFGFSILL	T	658
5359	LOC_Os02g01020	GLRLKKHQRKRSSMRHPLSRQRLAA	S	394			8312	LOC_Os02g48360	YKNQGGYDMLGRTKDQIRTTEQVNG	T	159
5362	LOC_Os02g01170	SSPSDTGMQIDRSASPDDDLAAKEL	S	467			8315	LOC_Os02g48740	GKTASSFLKSSTTTLLHNLNQAEKS	T	121
5365	LOC_Os02g01960	PPPPPPPPPEGESSAEEQPPEDQQKG	S	153			8318	LOC_Os02g49090	PPQLEVTPITKYTSDPGLVLGRQIA	T	208
5368	LOC_Os02g02360	NAVISGGAALSSSAARLVTVPEVLE	S	80			8321	LOC_Os02g49620	RENKLDRHGEVDTANVDEIQSVDED	T	217
5371	LOC_Os02g02870	GGGGFGSRGGGGSGGGGRSYGGSWG	S	100			8324	LOC_Os02g50550	LPGIDQRVMDDSTISEYAGHNDAIL	T	165
5374	LOC_Os02g03060	VDLATVVPTLDSSGLDLLSKMLRLD	S	342			8327	LOC_Os02g50840	LPSRGEQPHPVETAAGSGTQRIITS	T	1476
5377	LOC_Os02g04050	VMNLLESAGFSRSNPYYVVQQGKIA	S	133			8330	LOC_Os02g50960	AGADPSKAMAAPTAMPPTSVMTRLI	T	426

5380	LOC_Os02g04240	KESSSHKRGSYVSQSAVALRCRVMP	S	209			8333	LOC_Os02g51520	TFITPIGTYCYTTMPFGLKNAGPTF	T	938
5383	LOC_Os02g04490	LFRHGMQCKTRASGGCVLCKKMWYM	S	1596			8336	LOC_Os02g52250	PCISNWKNPKGYTIPLDKRLAADGR	T	276
5386	LOC_Os02g05310	PAAGGITPGATPSGAWDATPKLPGG	S	281			8339	LOC_Os02g52820	ETEAAKAPVDSTTLQLHNLLYEKNH	T	108
5389	LOC_Os02g05450	RLQRLKENMLSSSSSTDNNVAAESG	S	651			8342	LOC_Os02g53580	EMPDRYRFLDRFTDEQLATMPESLH	T	105
5392	LOC_Os02g05620	VYADARGGVVSASLRALGLDYLRNP	S	246			8345	LOC_Os02g54110	TSSGWAYTGHDYTSKKASGDLKKKD	T	1196
5395	LOC_Os02g05840	PKANSGGQSDPSSEGVDSNNNTAVY	S	471			8348	LOC_Os02g54700	MRRRNDENSVRVTNLSEDTREPDLL	T	213
5398	LOC_Os02g05880	HLDRAHLERINISTEDILQKCQEVS	S	1014			8351	LOC_Os02g55560	HQNALSSGTTALTAMIFGRSLLVAN	T	160
5401	LOC_Os02g06430	LPRAPSSHRKHRSRSAHPRSGGRDD	S	552			8354	LOC_Os02g56560	RGSLPWQGLKAGTKKQKYDKISEKK	T	220
5404	LOC_Os02g07190	CEYAVQEVARACSNLREDPNLGTWL	S	465			8357	LOC_Os02g57510	NDMDSSRGFLSGTVDKFKMVFETKS	T	87
5407	LOC_Os02g07870	DRITTFEDSEKESEYGYVRKVSGPV	S	17			8360	LOC_Os02g57640	AGTSERKVTITGTSEAIQAAESMIM	T	322
5410	LOC_Os02g08190	FYAKYQSCLFENSYGVAATDEGRAK	S	198			8363	LOC_Os02g58110	TETRSHAAAVTPTRPDEVILKDDAS	T	409
5413	LOC_Os02g08350	LVDDCSNCQLSASLRPHDAVSAVGR	S	347			8366	LOC_Os02g58340	RVEPIHVRPAVRTLLARLEGELTDL	T	103
5416	LOC_Os02g09750	KARVVKAVHGEASSLLICDDGVAVP	S	185			8369	LOC_Os03g01040	TAFITPIGTYCYTTMPFGLKNAGPT	T	918
5419	LOC_Os02g10510	VTKLVELGFDRASVIQALKLFNGNE	S	393			8372	LOC_Os03g01470	APPWPDLAGWRLTAGVETAMVGARG	T	19
5422	LOC_Os02g10790	QQCSIKIMGILSSRIVGLVQLLLKA	S	260			8375	LOC_Os03g02160	DSKVKEELYVESTVVIRDDMVVNPA	T	504
5425	LOC_Os02g11060	IGTFEGHKGAVWSCCLDTNALRAAS	S	66			8378	LOC_Os03g03460	DTLKDGFVVALRTKDNSGFHVARQR	T	302
5428	LOC_Os02g11820	EPPPQMQRTSTASSIGSSEGTSEQI	S	326			8381	LOC_Os03g04270	KPYFPRLRWLGVTGSLNRVMVDALV	T	570
5431	LOC_Os02g12360	IEVEKKVEDNEGSKSSSTSKGRKQL	S	430			8384	LOC_Os03g04590	LPSACVGDMVMATVKKGKPDLRKKV	T	64
5434	LOC_Os02g12710	SKKIMAETFPLVSRILEVSTDASLV	S	155			8387	LOC_Os03g05200	GSRCDSFGRTSYTYENGHTSPDLLS	T	568
5437	LOC_Os02g13170	WSVAAATGRSCESVAAGSSSSSPPP	S	79			8390	LOC_Os03g05460	VERCGRHPSQVFTGVCSTCLMERLS	T	45
5440	LOC_Os02g14130	HKRMPPEAIDLASRLLQYAPNLRCT	S	336			8393	LOC_Os03g05812	SSSVVSLNSAPTTPNSSYDNLNESV	T	739
5443	LOC_Os02g14760	LQVGGGGGRDSSSDGSSNGGGQPQN	S	133			8396	LOC_Os03g06260	ASKPEGGNERLSTSVPAAPRQRMIM	T	702
5446	LOC_Os02g14780	MFTFFTKLTDTYSRVLNPVEGMPAL	S	238			8399	LOC_Os03g06950	VDDISGTLAGISTEIDISTFYRGLD	T	1502
5449	LOC_Os02g15910	RRGSLAVIDCDGSNSDIKMKQGYMS	S	38			8402	LOC_Os03g08550	AEFQAAQQSAGCTAPEVDMTGQYTL	T	584
5452	LOC_Os02g16090	WEIIKDDILLVFSSIYNLRCAHLNL	S	485			8405	LOC_Os03g09100	EQDEIQTTLSNPTKEIIDLSKKISS	T	569
5455	LOC_Os02g16620	PAGEAAPQADGVSEEAAARPRGFRL	S	16			8408	LOC_Os03g10180	DYVLGDKSEESGTKHNTNNAKNNVL	T	324
5458	LOC_Os02g17970	PLGNYDLRYSYLSQRGYYPESLDKP	S	113			8411	LOC_Os03g10420	SMTAYGFEPDLRTYNVLAVGLCQAG	T	315
5461	LOC_Os02g18000	LAQHICNHLLVKSHFDPVIWIFVSD	S	251			8414	LOC_Os03g10800	SDLLIPTVDGETTVYDIDMILSIVE	T	338
5464	LOC_Os02g18910	AYGGRQEGSSAISNHDEFVLEKEDY	S	30			8417	LOC_Os03g11200	PPTYPAQAYTQQTYAQPSAMAVAAP	T	749
5467	LOC_Os02g19170	DTDDGKAPNAGNSPQELDPERAKLH	S	405			8420	LOC_Os03g11500	TFISKLQVEGYSTERLLSHSIVWRA	T	445
5470	LOC_Os02g19330	QRPNGLVPPQMLSRRPQHGMAPIQH	S	314			8423	LOC_Os03g11580	PHYNPFASTFEQTDPTLNIGCDVIP	T	720
5473	LOC_Os02g20570	DKSSSSYRGRSKSRGRYKSCKYCKR	S	229			8426	LOC_Os03g11890	EAVCEKAAVRDETANAAEEAGQRTS	T	345
5476	LOC_Os02g21150	RTPEPSEPSSSSSSSSSLSDRHPR	S	431			8429	LOC_Os03g12010	HKCESCGDLIKSTKIKGPSQDDCIE	T	157
5479	LOC_Os02g22090	FVNRDAALVLSYSVIMLNTDQHNIR	S	670			8432	LOC_Os03g12460	NTIWSLRDATQQTFFNILKSRGEKL	T	400
5482	LOC_Os02g23827	GIIYPISYSEWVSPVQVVPKKGGMT	S	531			8435	LOC_Os03g14490	NDEVLSLGNACGTFIQWPKDLIEIR	T	362
5485	LOC_Os02g24280	VCVLHDRHAGLLSTIQKLQEDVTES	S	508			8438	LOC_Os03g15000	ESSPSHYVFAAATPYPPPQYTNPNL	T	59
5488	LOC_Os02g24354	DITLVGVHCHLGSTITKVDIFRDAA	S	272			8441	LOC_Os03g15040	VRARKDITDGGKTTKRPRMITRVGC	T	260
5491	LOC_Os02g25060	PPINDKMQIEVISRPPSIGIHSKEN	S	486			8444	LOC_Os03g15750	DVFLETAKLCKYTEKELGASLFFGL	T	185
5494	LOC_Os02g26140	HGKLLPSRPFTVSPPGKVQVAAAAA	S	303			8447	LOC_Os03g16700	FKVPELLIEIPETATVGSLKKTVLE	T	416
5497	LOC_Os02g26349	SHSHSHRGRSSPSPDAGRHKRRRDG	S	90			8450	LOC_Os03g17050	RTEEVAELVLIATHNECRKKGYFSL	T	918
5500	LOC_Os02g27740	RNQHGLNFQNESSFRNHQYPNFQNQ	S	265			8453	LOC_Os03g17240	LLLLPDMNVNALTRDRKTAFDIAEG	T	422
5503	LOC_Os02g28810	ELGRQPANTKLSSNKTVRRVRVRGG	S	40			8456	LOC_Os03g19150	RQRLDAGGRSSATKASGAATTGSAS	T	129
5506	LOC_Os02g30140	APPPPSLSSSLRSLSLSSSPRGRHH	S	33			8459	LOC_Os03g19290	MSGSSNSSLETRTFLDEVRGLEKNW	T	13
5509	LOC_Os02g30240	EEQGVGGKQQQQSVIELARGAAAAE	S	159			8462	LOC_Os03g19590	EQSGASGRSFDYTQSLRASPTPSSE	T	88
5512	LOC_Os02g31150	FEALGNNAGPSTSPPASERKLFSDV	S	649			8465	LOC_Os03g21040	RIFCKADEGFSVTVRGGSVCLAPTN	T	213
5515	LOC_Os02g32090	HSDKNHEPTRITSEATRDKDKERGA	S	104			8468	LOC_Os03g21350	RRVKAVPAQSNSTAPAPKARVNHVA	T	304
5518	LOC_Os02g32469	STNTTPTGAAASSPRTMRRSFSNAS	S	32			8471	LOC_Os03g22120	RVNVHELSVEQLTVSEYLRFKEELV	T	111
5521	LOC_Os02g32690	NWLKWGFWISPLSYAEIGLTGNEFL	S	719			8474	LOC_Os03g22880	KNREVSEDAEPKTATEGKKKKKKSK	T	535
5524	LOC_Os02g33080	GAEILFYPTAIGSEPQDNNLDSREH	S	188			8477	LOC_Os03g24110	IEELPAVLWANRTTPSRATGETPFF	T	1833
5527	LOC_Os02g33540	EQNMKFSRPPLLSDVHKMYVEKVRN	S	131			8480	LOC_Os03g25260	HYCSVCGSTANYTCVRCGTRFCSCR	T	148
5530	LOC_Os02g33960	LEQLKQKQQDGKSSTSGINVIEINL	S	193			8483	LOC_Os03g25450	VCVARFHAAVPDTARVARALEALTG	T	184
5533	LOC_Os02g34210	EPPPMKKSRKAHSSPQRITLDKPEA	S	74			8486	LOC_Os03g25890	ADFAGCVDTKKSTSGYVFTLANGAI	T	765
5536	LOC_Os02g34560	IVTGCDPKNTRWSYHNGGSWPVLLW	S	456			8489	LOC_Os03g26630	TVENEHLHAENNTEPFVEKTQDVGT	T	163
5539	LOC_Os02g34590	IFNCIAEKFTSPSTIVVVVDKLTED	S	478			8492	LOC_Os03g27800	KSEVDKEKASLVTLQQREGMASIAV	T	483
5542	LOC_Os02g35110	SKRAAAPAPSQDSPVAAFLSRFAAA	S	53			8495	LOC_Os03g28960	SVTSSTHEIKSKTVIFMEKEKIYLQ	T	226
5545	LOC_Os02g35190	EPLLRKRTMNTTSQIAIVGANVCPI	S	68			8498	LOC_Os03g29750	GNFECDVESKAFTLAPDASTNREFN	T	374
5548	LOC_Os02g35690	RHPQWQEVAEVVSSRDGYSKQPKSD	S	101			8501	LOC_Os03g30790	LPWITQFEPIQVTLQTDQVRNIPCG	T	113
5551	LOC_Os02g36974	SKICDGILKLLDSHLVPSSTAPESK	S	113			8504	LOC_Os03g31750	QARAIFEAAISMTEQGVKVFPEIMV	T	709
5554	LOC_Os02g37080	WKCVYFLSGEILSLSVTYNEPWFTN	S	94			8507	LOC_Os03g35340	EVPSLTAIRSLSTGTVLGCQVEKHM	T	1004
5557	LOC_Os02g37856	TDMVLLFDIGCVSSTPTEILRKGRS	S	183			8510	LOC_Os03g39170	AAPPLEARAKEATAMAKKWIAMVAG	T	557
5560	LOC_Os02g38050	LRSKIASAFKFGSDVDFILTYTDED	S	62			8513	LOC_Os03g39570	VWCMMQYIDAKKTKEPIGFLDPTRI	T	721
5563	LOC_Os02g38430	MEPPSGIRASAWSFFKFLPFFLGLL	S	13			8516	LOC_Os03g42220	RSLHDALCVLSQTVNDTRVLFGGGW	T	393
5566	LOC_Os02g38980	PEGENSKPDRQVSPDKLLRAALLRS	S	545			8519	LOC_Os03g42840	INKEEFQLALFKTNKKESLFADRVF	T	78
5569	LOC_Os02g39210	PAAARKPPPLALSSPPAAAEQEDDL	S	177			8522	LOC_Os03g43760	GASMGTTDLKIPTNNSNAGARTNLP	T	153
5572	LOC_Os02g39520	HGQPVLFGTGLLSDLGVDSYVWLFR	S	318			8525	LOC_Os03g44310	RFLKNRIAKLPLTLSESEAKASLQS	T	374
5575	LOC_Os02g39720	SRSPPPARARSYSRSPPPPRERSYS	S	173			8528	LOC_Os03g44440	AVRSDVIKSNDFTSKLLYCLWWGLA	T	355
5578	LOC_Os02g39840	KATDLETGCLLYSSLLDELAIDLPK	S	694			8531	LOC_Os03g45180	SMTLKQKQSKASTQPMYNKGSPNPR	T	390
5581	LOC_Os02g40690	PLRPRHPAPPLPSPQWSRKNPSHPA	S	38			8534	LOC_Os03g46046	RSSAVGIPCEKVTRVRILGGQLRST	T	25
5584	LOC_Os02g41550	SLGCPLVLIRAESTLEALLRCIDSV	S	81			8537	LOC_Os03g47410	TPWQWSAAAGEVTSTEFEVEEPVGA	T	746
5587	LOC_Os02g42134	ASLLNLPFPRQVSYGNLEAEHHGSA	S	434			8540	LOC_Os03g48350	IDKFTKWIEAIPTGEIKADNAIKFI	T	347
5590	LOC_Os02g42550	IIDKTETWRLEESQMTSGTSHYTGE	S	178			8543	LOC_Os03g48930	VGASLYYIDYIATFHKIEKGAFGYG	T	122
5593	LOC_Os02g42580	SAFFTDLREARASLSAPSSQATTQD	S	42			8546	LOC_Os03g49580	DTLKTLEMGAVETLIVWENLDVNRY	T	320
5596	LOC_Os02g43740	VWDASFPPSGNVSPHSSSDSTGAVA	S	183			8549	LOC_Os03g49640	SRSLDLAVGGGGTDIRISGSVEGEV	T	103
5599	LOC_Os02g44070	RRVQTRAGLSKLSPMWEGPYRVVGV	S	1780			8552	LOC_Os03g50290	EEMVEFMEKVAKTTDVGELTVEERN	T	37
5602	LOC_Os02g44690	DVIYITLFVQLMSIFSDKFWWTYLV	S	109			8555	LOC_Os03g50450	WNGSDPGGACSWTGVTCEGGRVAVL	T	65

5605	LOC_Os02g44870	PAHPPPATEEVSSPDGKEKKGILGK	S	248			8558	LOC_Os03g50740	LERQQPSLWRRGTPLAMEVSAGREL	T	71
5608	LOC_Os02g45054	KRYAVQSDWKAHSKICGTREYRCDC	S	191			8561	LOC_Os03g51230	HQKEIQNHLVEQTFDEYLHEKSEIV	T	549
5611	LOC_Os02g46650	DKNVTRLVRSMPSTRRKGIVDCLSQ	S	631			8564	LOC_Os03g51310	IYFVSEALRDAKTRYPQAQKMLYAI	T	1226
5614	LOC_Os02g47150	EHILLRPDTYIGSVEKHTAQLWVYE	S	51			8567	LOC_Os03g52980	RPKHLVVAIGNFTYLMLPQLEPVVP	T	559
5617	LOC_Os02g47170	SDMADGNPWLDKSDGFMEGSPYKIN	S	462			8570	LOC_Os03g53270	MKGFAGAHADAVTVSLGPAGALAYS	T	47
5620	LOC_Os02g47810	STHTKFCYYNNYSLSQPRYFCKTCR	S	82			8573	LOC_Os03g53880	VEDDVGSQGMELTSQGAGTYWYLPP	T	567
5623	LOC_Os02g48000	FATLKKHVFIIRSDDDPNVFLVNDF	S	184			8576	LOC_Os03g54780	GGSSSPRESVSGTFIRRTGSPSSPH	T	168
5626	LOC_Os02g48380	ITSVRYSAALTPSASAATVFVYPSE	S	1148			8579	LOC_Os03g55070	VSKSDKIVVEKSTVPVKTAEAIEKI	T	128
5629	LOC_Os02g48810	DTCARSNLNAVQSSNPVAKNQDTKT	S	131			8582	LOC_Os03g56260	STSSRSHAVLEITVKRKQKGQYGSQ	T	229
5632	LOC_Os02g49090	KFDEAFTMALQRSDVSIVSWLCSQV	S	1226			8585	LOC_Os03g56800	LVSDDSEVIDLITVNDVERIRSYPK	T	209
5635	LOC_Os02g49310	IASQEKKVVEYESVNGSSTSFHTCE	S	105			8588	LOC_Os03g57100	AFPHAEDHLLKKTYSGAIVTIFGLI	T	24
5638	LOC_Os02g49450	FAGEAAKRSQELSAEAAKRSSALVS	S	22			8591	LOC_Os03g57160	ERDPAEGRDEEATNEGKSLSNAGVD	T	406
5641	LOC_Os02g49992	KLIVRILNVLGVSTKTPFSESFAQY	S	1202			8594	LOC_Os03g57430	GLKQSSTPDSEQTNTADVKTEARLG	T	528
5644	LOC_Os02g50550	QNELARLGEQMVSSAEGTRAVALEL	S	341			8597	LOC_Os03g58170	AATDEIFCLFQGTIENIAVLKQQYG	T	81
5647	LOC_Os02g50700	EKEKFKQRVEEGSLQEFNWPSSNQK	S	157			8600	LOC_Os03g58810	FLIYLKAAKISLTELVFNENKVPNL	T	472
5650	LOC_Os02g50760	YREVLAKFTEMTSRYAQEMQTIDEL	S	340			8603	LOC_Os03g59390	EDIINSIIRDVDTDKDGKISYDEFA	T	487
5653	LOC_Os02g50799	TVMKLAELHKESSEEWSKKAGELEG	S	292			8606	LOC_Os03g59680	EQLISEFKDLSCTPSRSISPEDDAG	T	43
5656	LOC_Os02g50960	VKWWRIFTPDQCSGINRFVALFAVP	S	40			8609	LOC_Os03g60620	VPAYFNDSQRQATKDAGVIAGLNVM	T	163
5659	LOC_Os02g51400	GGRPKYSRSSGISKLFTNSLIVPLP	S	506			8612	LOC_Os03g61220	ALDGRDLIARAKTGTGKTLAFGIPM	T	150
5662	LOC_Os02g51860	YQPLNPCIAGTRSQRWISIEHRTTW	S	436			8615	LOC_Os03g61890	AWKKALQLEAEGTEDLEFSCNVLVL	T	578
5665	LOC_Os02g52810	GNVGGMSKSSTASSRSRSGTFPSPG	S	173			8618	LOC_Os03g62379	DQTGTFRGNRLSTSQFGDMDDEYNE	T	23
5668	LOC_Os02g53420	WVETTDGKQYSPSQIGAFVLTKMKE	S	164			8621	LOC_Os03g62700	VPELSLEDLKQKTDNFGSNALIGEG	T	66
5671	LOC_Os02g54010	EEIRATTNDGSESTSLLTETTEIVK	S	450			8624	LOC_Os03g63920	IEQEPNEYNGFVTKLCQKFRPAGDK	T	635
5674	LOC_Os02g54200	KAAAPPPEYEMPSVTWGVIQGRKER	S	42			8627	LOC_Os03g64400	ALDKRQLPEVDKTSMYKASGYMDVD	T	538
5677	LOC_Os02g54490	ISTGLHCLHLDTSIFFNRKNELHQI	S	243			8630	LOC_Os04g02150	PQPTDTAVPDEKTQSPKEQSIDIDI	T	340
5680	LOC_Os02g54500	RSGRDFENASTDSEKLSPRFLSYFQ	S	933			8633	LOC_Os04g04254	AEALKVPIHIFFTMPWTPTSEFPHP	T	299
5683	LOC_Os02g54770	RRSVSPVRGRSYSRSPRGRGGSPYA	S	160			8636	LOC_Os04g06770	GVGRKVIDKLQQTYRSELSSKDFAY	T	107
5686	LOC_Os02g55060	AGREASRALGKMSKDDADVSGDLSG	S	62			8639	LOC_Os04g08450	SGDEELSVLPRHTKVIVTGNNRTKS	T	32
5689	LOC_Os02g55940	TSPQGPNSGHFSSLAESLRNLPSGR	S	152			8642	LOC_Os04g10280	NVEAYVDDLVVKTRNQEMLLSDLAE	T	988
5692	LOC_Os02g56480	PPQPKQESVSVQSPPPAVVPMPQPP	S	209			8645	LOC_Os04g11990	LVDPISCIPPYATLIELPADPFPPD	T	392
5695	LOC_Os02g57080	TVATCTTSVKTFSLSQLEKATDGFD	S	333			8648	LOC_Os04g14580	KLELPPEFGVSPTFNISDLKPYLGE	T	1222
5698	LOC_Os02g57470	HFSQLPPPPVPASAAAAAAAVAEEA	S	49			8651	LOC_Os04g15800	EPKAKRRCSLAETVSFDGETPVAGA	T	116
5701	LOC_Os02g57500	RDKLSSSDAAASSPAAAAATDAAAE	S	58			8654	LOC_Os04g17064	VTIAMGTNRDITTACKRKIVSPSSP	T	224
5704	LOC_Os02g57660	ELRIHRRFDLKGSSLGRSTDKIEID	S	590			8657	LOC_Os04g19770	VEKAFRQGFFWPTALKDACDMVQRC	T	373
5707	LOC_Os02g57980	RFARFGASSEEESGYDELEARSAIR	S	265			8660	LOC_Os04g22830	WARVRGKKNSDGTLSFSNPEDQAVY	T	228
5710	LOC_Os02g58210	CSKENVHTLKDKSNGLDINASPPDD	S	749			8663	LOC_Os04g23400	TEAERVVADIETTNDWWTPLIKFIN	T	407
5713	LOC_Os02g58410	FQDLQPLRYALASVLRYLAPEFIDA	S	930			8666	LOC_Os04g24880	RAQDNRTKSEEDTRARTETMITATT	T	248
5716	LOC_Os02g58570	TPAVLPTPRLSPSREGLSDMAQAQW	S	131			8669	LOC_Os04g27290	NSDEITSTSNSKTKVQKTGPAKDAE	T	239
5719	LOC_Os03g01490	EYVPEFNLWFGFSSDESPHYLAASN	S	290			8672	LOC_Os04g27860	HSQTVVVCPGCQTVLCQPTGGKARL	T	63
5722	LOC_Os03g02010	SIYASQEAGNGYSANLSDYEDTEFS	S	230			8675	LOC_Os04g28180	QVDAAPFKQWYLTHYGVDIGRKKKA	T	116
5725	LOC_Os03g02160	TASSQHEGSCLSSTTNLCTHDELHS	S	86			8678	LOC_Os04g28460	TVVPIKSNDGTITYQAQSQDEEALV	T	428
5728	LOC_Os03g02270	AHALKPNYSVYRSALRLVRSMLPLP	S	341			8681	LOC_Os04g30010	FTLYDREKIEVATNNFAKENIVGKG	T	444
5731	LOC_Os03g02320	QKERTCSGPILCSGVHNKSITESSR	S	487			8684	LOC_Os04g31090	GSQGWLAYMVIYTPLPGTIYVLLCI	T	145
5734	LOC_Os03g02390	SGSGTRDDRTDASGRRLYNPYQDLN	S	21			8687	LOC_Os04g31340	GNISIRDVQYLLTAPNIYKDYRTIT	T	318
5737	LOC_Os03g03390	SDEENSTQSNELSELEEDGDEEESA	S	277			8690	LOC_Os04g32650	GQAVSEGTVNNETLGYFIGRVYLFL	T	340
5740	LOC_Os03g03470	DATLKCSRNRSASCPVAFTDKYCSL	S	304			8693	LOC_Os04g34450	EYFETILRTYLSTEAEQALRSLQEN	T	966
5743	LOC_Os03g03560	GPSVNGENESPRSLGETTDHAGEQD	S	778			8696	LOC_Os04g35800	ADPAAAAPAAAATDPAAAGSPSPPL	T	34
5746	LOC_Os03g04450	DIYWNHLGLSKTSLGLRRIAVNTCL	S	384			8699	LOC_Os04g36890	GFPVSFSESKKRTDGSSKKKGNLNN	T	248
5749	LOC_Os03g04710	PPTISCRLTPGGSDAHDRSLGGLRR	S	55			8702	LOC_Os04g37960	PNPKPSSDIGGSTYGSFANLKITAE	T	41
5752	LOC_Os03g05260	DGCVTRMLNTVDSEGDTPLHHAARG	S	145			8705	LOC_Os04g38620	TLTFSPNPYFEDTKLTKTYSFSDDE	T	134
5755	LOC_Os03g05390	PIIGLLFGTMVVSIFLERADMFKYL	S	76			8708	LOC_Os04g39400	TTGVGPLPACPTTPGAPDPQDGPEA	T	1255
5758	LOC_Os03g05730	FEESMKYARRSVSDADIRKYQAFAQ	S	754			8711	LOC_Os04g39780	DTLIRIVDPDSLTEHQEDGVEGEIW	T	864
5761	LOC_Os03g06340	AISSDMDELDSDSRKRASLKSKREF	S	451			8714	LOC_Os04g40720	GCFLHVKMTVVVTDSHCCRDTSMED	T	111
5764	LOC_Os03g06560	TARQRLDAGGRSSATKASGTATTGS	S	127			8717	LOC_Os04g40950	GILGYVEEDLVSTDFQGDNRSSIFD	T	286
5767	LOC_Os03g07380	KNGALPDYSMDFSKIREMRKQNKKE	S	43			8720	LOC_Os04g42140	DLLKGQLMESGITTADILKDVISLI	T	236
5770	LOC_Os03g08320	AEKAGELMKLAGSRDSTAAAAVSDA	S	128			8723	LOC_Os04g43130	KKAVLWNMETFQTQYTAEEHAVIIT	T	550
5773	LOC_Os03g08710	QRLKAARGREKSSDEVFVCERVCTS	S	44			8726	LOC_Os04g44320	MFSIIVMKLNLTTGIIPSLNVSAGL	T	98
5776	LOC_Os03g08830	TSMIAGPEIDVSSVIENNQRKLSRN	S	714			8729	LOC_Os04g44960	WIAEIQAYLTDKTLPEDREGSERVL	T	1629
5779	LOC_Os03g10140	HQETMQVPLPASSLPEWPPRLQPEP	S	436			8732	LOC_Os04g45800	ISASLLIVKGGFTALDILSVEWIQS	T	352
5782	LOC_Os03g10420	YRRHLSPPSCLPSLVPLLPVLPYRH	S	95			8735	LOC_Os04g47240	TSPISVPRSPGRTENPPSPGSRWKK	T	399
5785	LOC_Os03g10590	VRPDGQGMRRGFSDHRNAGQRNGYD	S	285			8738	LOC_Os04g47330	EAALKGYVRRRVTPAIGDAEFVVVY	T	61
5788	LOC_Os03g10740	TRGVGGKPYRNDSSQKDATRIKAGI	S	21			8741	LOC_Os04g47870	RTVDRSGNILPGTVVDSKICHPTEF	T	983
5791	LOC_Os03g11140	ASSSRAKFMEKSSSRNKSKKTLLGR	S	518			8744	LOC_Os04g48770	ILEKTLRKLSCITTGDSIMVAYNNK	T	143
5794	LOC_Os03g11240	EDNGFIMNEELPSVSEASDKQQEEQ	S	599			8747	LOC_Os04g50890	IKEGDSKLFFLATIYALLGIPLSYL	T	164
5797	LOC_Os03g11550	AEPAAARLPASGSPSSGSSDGEGRR	S	124			8750	LOC_Os04g51390	RVALGPGGDLALTSRIRNTNTDGRP	T	159
5800	LOC_Os03g11970	PQISTAVVEPYNSVLSTHSLIEHTD	S	187			8753	LOC_Os04g52050	NEEPTAADLFLLTHTHRNGKPMKKE	T	808
5803	LOC_Os03g12230	THPNALTKDELNSMIKANRNMYDFI	S	212			8756	LOC_Os04g52460	VGINEALKGELDTKVAMFDELQEQF	T	1262
5806	LOC_Os03g12300	QEIQRYEEAHSSSTSEIGRPPSRTS	S	695			8759	LOC_Os04g52570	TSPTKSLSEVHSTFSSWHGSSSHQY	T	216
5809	LOC_Os03g12550	NGAPKQVWEPMDSQKKNLLDNKNNG	S	449			8762	LOC_Os04g53540	SEASRDSAVVIMTHANLVEILMDAN	T	365
5812	LOC_Os03g12910	TRIGAAPRPQQRSPPPRAERPRRPH	S	66			8765	LOC_Os04g54680	KRKVEDQGPLKKTIRSNKESNDEIN	T	403
5815	LOC_Os03g13560	SGHALPHQMMTASQSPAEPISSTDS	S	567			8768	LOC_Os04g55150	LLEGVVAYHNGNTVKARESLTSAQA	T	284
5818	LOC_Os03g13770	FRGNIGRHGEILSDHAEESQPKGSL	S	481			8771	LOC_Os04g55450	TQETEAGQGPDVTPQQAARDRHPPD	T	1558
5821	LOC_Os03g13970	ALQMSVQDAGGSSQSDMSKVFEDRS	S	343			8774	LOC_Os04g56530	SGSGASVTQNGSTPRRRSGQLDPPM	T	785
5824	LOC_Os03g14250	QSMRPPAESMGSSPNQRMEDFLQDA	S	939			8777	LOC_Os04g57140	EDMLCFQKDPIPTSLLKISSDLVSR	T	42
5827	LOC_Os03g14580	SSATKTNSAVKDSGKTTAQKKRRAS	S	282			8780	LOC_Os04g57600	RHPPIDGMFGAPTTGMPAVSSHYAP	T	96

5830	LOC_Os03g14930	VARKEEAWTWNPSGSGVAASAAPGS	S	403			8783	LOC_Os04g58720	GILGAQGIVPMKTRDGKGSSDTYCV	T	615
5833	LOC_Os03g15033	AANIYFIYQRLKSGWRTFFYGFGSF	S	225			8786	LOC_Os04g59100	HPRSNGQVERANTEILKGLKTKTFN	T	1531
5836	LOC_Os03g15160	EKAFQTLKEKLVSSPVLILPDTRKD	S	1136			8789	LOC_Os04g59330	SGSMAPVPMILRTTVHSRAYVLGAL	T	270
5839	LOC_Os03g15560	AGETDNPKDAVDSVTKQISGIAISE	S	130			8792	LOC_Os04g59550	WIFQKLDFKFPLTVSCVHFICSSIG	T	74
5842	LOC_Os03g15580	GTTTTSLGKFVSSGISCGDGSGNIS	S	1125			8795	LOC_Os05g01020	GASVLNDLWVSVTSGSEDYSFKHMR	T	493
5845	LOC_Os03g15900	DIYRNRHNISTSSGLSDPAVATGIS	S	788			8798	LOC_Os05g01400	WITEIQAYLADKTLPEHREGSERVQ	T	1470
5848	LOC_Os03g16369	PHHRRSPGRAPRSPSPARHRSPRRR	S	316			8801	LOC_Os05g01990	EDKEGLFASCSFTDLGLHPTLCAHL	T	26
5851	LOC_Os03g16690	GETYEMVNHQGISFIAEQVSHHPPM	S	168			8804	LOC_Os05g02670	PIVNEVLEGYNCTIFAYGQTGTGKT	T	130
5854	LOC_Os03g17170	EALRNMKAFWLPSATPEATAKVEAP	S	160			8807	LOC_Os05g03430	NTDDRPQQQHNATDVIVLSDSDEEN	T	601
5857	LOC_Os03g17540	QELRKKHRPDIISAMEERSKDRHSW	S	371			8810	LOC_Os05g03820	GSLGLDMMFRTCTVQVNLDFSSEQD	T	222
5860	LOC_Os03g17780	GRQEIGSSTPRASSRRISGSEGLVM	S	32			8813	LOC_Os05g05700	ENDFEDFLLKDTTDYYSLKAQSWIL	T	204
5863	LOC_Os03g18070	ESWQPLSERLYNSLDYMTKKLRFTM	S	238			8816	LOC_Os05g06630	SWDGATKRAMIVTLFTYFKKKPHEP	T	197
5866	LOC_Os03g18970	GRNFPSKANSSPSRCAICQVDQAPF	S	373			8819	LOC_Os05g07220	SLAYPDQHVPEETQVTAGISCPDQH	T	632
5869	LOC_Os03g19340	DPAKTYSAVYKRSKTNKHKKNLDSD	S	1200			8822	LOC_Os05g08600	SSDYFKELYRLKTYHEVIDEIYNQV	T	36
5872	LOC_Os03g19590	SFKAFLEVVKSRSLPWENAEMDAIH	S	605			8825	LOC_Os05g11370	HDSDDVDVLINNTLVGIKNTLEKPI	T	271
5875	LOC_Os03g20340	SRKPMQKSGKDHSLLLILPSGVYRY	S	148			8828	LOC_Os05g11770	EMAAQYWKISDITCIDCKWAQSVGS	T	193
5878	LOC_Os03g20600	ARAVGQLGCIRKSWSNDSLSSYGGG	S	38			8831	LOC_Os05g11980	TQLGLRNAMKRRTKVPEAPLSAQEL	T	934
5881	LOC_Os03g20670	LIQMLDLQSSVPSSKSGVRFVELLG	S	240			8834	LOC_Os05g13520	ALSGRLARNVLLTGKVLLNGKKRRL	T	100
5884	LOC_Os03g21040	PEYPDESVLWTESGDVGKSFRCIRM	S	123			8837	LOC_Os05g14770	QAYDRTGEEYILTLKYIKANNSYRL	T	328
5887	LOC_Os03g21270	KGENALHTRNISSHTIIQRPITNNV	S	251			8840	LOC_Os05g16640	GGMSKPSGSVEVTTEATTSGQVGAK	T	240
5890	LOC_Os03g21470	TRFKRDEYLKLKSEGRIVPDGVNAK	S	186			8843	LOC_Os05g17750	SFWDYALGTAAFTLNRVPSKSVDKT	T	365
5893	LOC_Os03g21940	GNMFKNKRVLMESIHKSKAEKAREK	S	141			8846	LOC_Os05g19100	TPQSSSKPLGKITLPMTFGQANNFR	T	673
5896	LOC_Os03g22730	MGDRTNAESLDFSEILSDDEVEAQL	S	221			8849	LOC_Os05g20190	ALQQRPKLNPYSTRGTTNKTQPRTS	T	120
5899	LOC_Os03g22900	SLPKKQIIRKDPSEIAREESEKTIK	S	335			8852	LOC_Os05g22580	GGGSSAGSSSATTGGGWIGGSGGGG	T	276
5902	LOC_Os03g24180	EGVDYGRQPMVASSTLVELTTRLDF	S	823			8855	LOC_Os05g22920	EQLNQLPSKEHGTNVMRIRPWYLDQ	T	429
5905	LOC_Os03g24790	HRIITDNGSQFISADFQDYCIGLGV	S	351			8858	LOC_Os05g23610	EIESNKPVRQKITEPKTPYHPMVDD	T	27
5908	LOC_Os03g24930	LASFRKLSSMTNSPASSVAGAAEGG	S	61			8861	LOC_Os05g25550	DAIVLYAARASRTENQDAIDASIVG	T	369
5911	LOC_Os03g25600	VPRGSGPLNTFLSGPLERGFASGPL	S	180			8864	LOC_Os05g27820	FSVLFSMISPFLTERTKSKFVIARE	T	281
5914	LOC_Os03g25720	TVPDHLKVRKKNSEESSTQWTTGIA	S	149			8867	LOC_Os05g28430	EAKIRASIRAELTSEFDEKLESMRA	T	268
5917	LOC_Os03g26030	SLWLLRHSSLMGSSDIDNWIQDAKE	S	326			8870	LOC_Os05g30530	FIKFDVGNIVMVTGGRNTGRVGVIK	T	184
5920	LOC_Os03g27080	HLSALTLKESKDSNAEEACRLTIPE	S	273			8873	LOC_Os05g30980	HKLFIGMLPKNVTDAEMTDLFSQYG	T	146
5923	LOC_Os03g27780	VCQLVMKPGEGISVHGSIPRNSRVD	S	396			8876	LOC_Os05g31056	SPQSSVSTQRRNTRRKVRTKSGQLP	T	476
5926	LOC_Os03g27930	IGRTKKKKRVRRSKSYHSCQDLAGN	S	452			8879	LOC_Os05g32960	SRGHAVPRMFYRTTIATGGYARDLA	T	505
5929	LOC_Os03g28930	FLQGCRPYIAMDSTHLTGKHRGQLA	S	492			8882	LOC_Os05g33570	TEACQQYQAAGKTLPAGLWEEIVEG	T	132
5932	LOC_Os03g29750	SNPAFTQLPSASSYLVMPNGTSQLA	S	572			8885	LOC_Os05g34540	VAPKGKFIAFVSTEAETDHPESELK	T	356
5935	LOC_Os03g30570	MSPDAKRARLARSSSIASVCSVSAA	S	62			8888	LOC_Os05g35800	LLSAILGFAAESTKITISDVRVSGD	T	29
5938	LOC_Os03g31730	AKFEERTREYQSSIDDQKRSNDALQ	S	401			8891	LOC_Os05g37720	AAAAAAAAAATATGKGGASWWGGGA	T	63
5941	LOC_Os03g32630	DDNIKDIVLENFSVSARGKELLKNA	S	164			8894	LOC_Os05g38160	ISIAFLNDSFAVTFNEKVTRTVRQF	T	97
5944	LOC_Os03g35340	VADKYFDGVALCSTSVVQLTTAFRI	S	783			8897	LOC_Os05g39390	FRSSAGVGIILPTKLFRVEVNYCYI	T	487
5947	LOC_Os03g37554	IWGYEVYVKRLQSDKLTPKSDKCFF	S	728			8900	LOC_Os05g39580	VREVRVVSGLPATSSRERLEILDDE	T	122
5950	LOC_Os03g37810	GSFKKGGGGGGSSGNSEKKTVAGNV	S	33			8903	LOC_Os05g39810	AASTKITVGDGNTTRFWDSAWINGR	T	750
5953	LOC_Os03g38260	DQFEAKRAKSKASRERKIARRDERL	S	169			8906	LOC_Os05g40230	MKEDAGKSLLRVTKDATAYRKVLKG	T	104
5956	LOC_Os03g39590	TARQRLGAGGQSSATKTSGAATTGS	S	127			8909	LOC_Os05g41060	NVETVEYKNISFTVWDVGGQDKIRP	T	64
5959	LOC_Os03g40110	RSARMDANKYNSSTVQKDSFGLTAK	S	278			8912	LOC_Os05g41210	FSLFDKDGDGCITTKELGTVMRSLG	T	29
5962	LOC_Os03g42220	MGQACTIVLRGASEHVLDEAERSLH	S	372			8915	LOC_Os05g41510	GRYLQNPLAMAATLCGPGKEILSWK	T	901
5965	LOC_Os03g43020	LERLEQEKESIVSQMAELKKILYGK	S	104			8918	LOC_Os05g42300	SAEEVPADISAGTAVSFVAEFADHI	T	340
5968	LOC_Os03g44020	FSLDSAATATPTSSPPRPHRAADVA	S	73			8921	LOC_Os05g43670	AHFRRFLARRSRTLRHLKELAVLRS	T	225
5971	LOC_Os03g44830	LLDSTTPSDPLTSSQHPAANTVRAD	S	336			8924	LOC_Os05g43970	ESDNFQKNKHKGTEGLIEIENPNLV	T	76
5974	LOC_Os03g45300	LPPGSIPNSVHPSSSHAQPGQCSFG	S	1010			8927	LOC_Os05g44310	VVASTPGQELALTNCAYVSSADIRR	T	32
5977	LOC_Os03g45760	SITHTTTNLLSTSLDVGSTVQEGTV	S	169			8930	LOC_Os05g45660	KREGSGRGNWGTTTDEILAQETGEA	T	186
5980	LOC_Os03g46330	RTELKLQLEAFFSCIILRLAQPRFG	S	153			8933	LOC_Os05g46340	FTLYSFRSGDLATDDIPADAAAAAT	T	274
5983	LOC_Os03g46480	LSPGPPNDEKTISPAAVDSKFTVQS	S	108			8936	LOC_Os05g46720	LDRFARPCFEGFTHNDEKKEIRSDA	T	17
5986	LOC_Os03g46920	AADACALPLEPASGSPRARVLTPEP	S	297			8939	LOC_Os05g47640	LYRYYKSGWTEFTPQVAEPTFASAI	T	383
5989	LOC_Os03g47410	YAGRITTMGVRYSALGATLNDAAMV	S	94			8942	LOC_Os05g48020	EKVDRANTDLKNTNVRLKETVLQLR	T	228
5992	LOC_Os03g48310	TYIDSDGKMYRVSKGAPEQILNLAH	S	426			8945	LOC_Os05g48980	NKADLRHLRAVSTEDAKAFAEKENT	T	137
5995	LOC_Os03g49230	GYIMGKVEGQGESWHGHVTAVSVAT	S	67			8948	LOC_Os05g50160	DSEAFYMMNPDSTSTSSNSNSGGPE	T	277
5998	LOC_Os03g50150	GNQCLEILALSYSCLPYHMKLCFMY	S	431			8951	LOC_Os05g51500	LVVDIMHGLEPQTIESLNLLKSRDA	T	720
6001	LOC_Os03g50210	HEPSSHDEDSGLSHRRRRCDGERRH	S	204			8954	LOC_Os06g01400	ASVGCAVMQCSNTEVGAGTMNPLTF	T	98
6004	LOC_Os03g50340	IIPIAVEKGWSESASPNVRGPPALV	S	987			8957	LOC_Os06g02028	AVKEYDDGKDLTTYDFEADCFSSPY	T	100
6007	LOC_Os03g50740	ALALCKLLPPEDSNLRAAKESSIHI	S	325			8960	LOC_Os06g02380	GTAAKVVLNKESTTIVGDGSTQEEV	T	385
6010	LOC_Os03g51020	LMHSEPVWQGFVSQNSELGMLGNGK	S	516			8963	LOC_Os06g03676	RTIALQEHELEHTASLLGEIGSNED	T	439
6013	LOC_Os03g51080	DFRLPLVKSINVSGHKYGLVYAGIG	S	274			8966	LOC_Os06g04660	EPQRWMRGKGRVTIQFGCCYNYATD	T	255
6016	LOC_Os03g51200	HIQLAVRNDEELSRLLGTVTIAAGG	S	98			8969	LOC_Os06g05010	RMLPWAKANINPTGQALIICTAAGM	T	60
6019	LOC_Os03g51250	GPSWGPVGGAPVSQPKATAPAPKAP	S	227			8972	LOC_Os06g05310	PPADKGYFGNCVTTCVAKAISGDLS	T	316
6022	LOC_Os03g51440	HDGTSVVVKRITSSMAKKDAYMAEL	S	470			8975	LOC_Os06g05660	KKGSKNAKPITKTEVCESFFNFFSP	T	246
6025	LOC_Os03g51790	ISTDSCSSTQPMSTCHVKTSAVTGT	S	582			8978	LOC_Os06g05870	PRLQKLGVYGVITLNEPFETLVPSS	T	87
6028	LOC_Os03g52490	VPLEMTRHVSMISITLSPRDLDKNS	S	109			8981	LOC_Os06g06830	PFTWTEEAERALTQLKAYLSSPPVL	T	1012
6031	LOC_Os03g52794	SKLGWSERMEEASRCLVLLAEILQE	S	652			8984	LOC_Os06g08550	PALTTYDIQLVLTLVGRFMRRAGVT	T	350
6034	LOC_Os03g53060	EIVGYGWADILFSPSGDYWRKLRQL	S	122			8987	LOC_Os06g08790	CHQKTNLEKAKATLLLATLPKSLPC	T	426
6037	LOC_Os03g53920	QLSHLQTLKKEFSVASTKARESETA	S	983			8990	LOC_Os06g09880	APALPKTNSMKLTEGYFMRAGLVPV	T	1354
6040	LOC_Os03g54970	CNKAAEYQCNDYSQIVCNQQTVLLP	S	235			8993	LOC_Os06g12030	EQPTGKGRIIIYTKLGCEDCKMVRS	T	249
6043	LOC_Os03g55450	EVEQVRSAPCVQSRSPITAPLGIST	S	153			8996	LOC_Os06g12970	TELGAISKIRGRTTRLRISMYADDA	T	56
6046	LOC_Os03g55800	RDPRVVALLDAASFPVLFDTSLVDK	S	115			8999	LOC_Os06g13810	TIDGDLKCKEVPTSFGFDTACKIYS	T	239
6049	LOC_Os03g55960	LQRVLAQLGDERSVADCEAMIRAYD	S	156			9002	LOC_Os06g14490	IQEGSSPTPPSDTHSEATSEAKSAG	T	407
6052	LOC_Os03g56250	SRSSWHFAFHGDSRRSSELRPTLGQ	S	703			9005	LOC_Os06g16160	DPARFLSSMRGKTLAFIGDSLARNH	T	139

6055	LOC_Os03g56990	PATVTEPAAATSSAVQSSRASSRSI	S	742			9008	LOC_Os06g17100	LSQGEQERSPARTAVRSQPSHQSGG	T	455
6058	LOC_Os03g57480	NGRRAAHATEGASSSGVSPQHGRGD	S	191			9011	LOC_Os06g17840	LGEKHALHDFVSTLSMRCSYLLVNK	T	593
6061	LOC_Os03g58170	ALGPGGANSLAYSSHGQSPLLPRLF	S	56			9014	LOC_Os06g19650	PGATSDAAVTAETVDASEKSSDPQT	T	285
6064	LOC_Os03g59250	MDPEVKDSRPSRSPGEPNLFLQWGS	S	13			9017	LOC_Os06g20840	ILTQFGMLHCNSTSIPIEHRSQLHK	T	309
6067	LOC_Os03g59480	QTNSSQPKKAKFSSSQSKQAATKTN	S	725			9020	LOC_Os06g21580	DKVVDQAEAKNLTEGDDGDADYQQG	T	55
6070	LOC_Os03g59760	WAPLLLGMMGGSSRRARRLRRQIME	S	69			9023	LOC_Os06g22650	QPSPYSNGLTSNTMAPYDQPSDINA	T	431
6073	LOC_Os03g60180	GQRRRWSDTNGQSDAKKNASQAVRR	S	79			9026	LOC_Os06g24540	VMQGWDVEEEESTVVVGEVEVEKKD	T	285
6076	LOC_Os03g60400	HLGNEWKKPFAGSSHAKGIVLEKIG	S	43			9029	LOC_Os06g25710	MKTYHSKDAIYKTITAHPNKDGHGL	T	359
6079	LOC_Os03g60530	SGVGKSCLLLRFSDGSFTTSFITTI	S	36			9032	LOC_Os06g27600	RRASRRELLEPGTFLDILMKPSVKE	T	240
6082	LOC_Os03g61150	PGEPLEGARLVASRRPYRPGDSRGG	S	70			9035	LOC_Os06g27970	QFNELYKHMDGLTNDVERSNEMVAI	T	286
6085	LOC_Os03g61560	TNKEQDGSESMQSASDSKKDPFQRR	S	489			9038	LOC_Os06g28140	EVYVKRLQSDKLTPKSDKCFFVGYP	T	438
6088	LOC_Os03g61890	SRSSSAATSPSRSPSPLVPRASVLG	S	38			9041	LOC_Os06g29410	LQTRNQHPHQLGTAGYAGKDEEWQR	T	172
6091	LOC_Os03g62379	TDKFYEGLQQFGSDFAMIQHLFPDK	S	86			9044	LOC_Os06g30570	APQAPPPAHRRATKKAKIDAAKNKD	T	643
6094	LOC_Os03g62640	TLLLDAFNKDRASLPQPTPTPQMAS	S	185			9047	LOC_Os06g30901	LRVGVADQMTEPTQREFLVFLGRKL	T	418
6097	LOC_Os03g63320	PLPQRRHHKTSSSSHPRPPRRAHPE	S	84			9050	LOC_Os06g31250	VFCYVKMPFGLITVGNTFQCTVQGA	T	887
6100	LOC_Os03g64130	AWVIISIERMARSPRLVGAEERDEL	S	342			9053	LOC_Os06g33520	SIDSRGRRRDHGTRGRNVGSLGPQR	T	344
6103	LOC_Os03g64210	VEQLGIARKVTISQSSTTIIADVAT	S	366			9056	LOC_Os06g35530	GNDPVTGHIISTTIGGKNDEPKRTI	T	57
6106	LOC_Os04g01620	LADIHEGECGAHSASRTLVGKAFRQ	S	1633			9059	LOC_Os06g35814	SLYFMETSALESTNVENAFAEVLTQ	T	162
6109	LOC_Os04g02370	NLTAAFVAQMVYSKVVRKTSLSPFT	S	541			9062	LOC_Os06g36700	IAAGKAVARILRTSLGPKGMDKMLQ	T	46
6112	LOC_Os04g03450	SEAMVPTELRHQSTRVQKYSDEEQE	S	1447			9065	LOC_Os06g36920	AGSPLHQKGLFFTSVMRERLRCRDA	T	130
6115	LOC_Os04g05360	LLSKEPTSYRMWSGTMGQYNILHEC	S	370			9068	LOC_Os06g38470	VEEAFYTTDRVMTVSFHKFGDYFPG	T	207
6118	LOC_Os04g06960	NPWRLVMKVRRTSNRLYRIELQLAS	S	281			9071	LOC_Os06g38960	SIQTKPGVSPRPTPTGTITPVSLVR	T	93
6121	LOC_Os04g08310	QEGRGSESALFMSGHFVFPPSEHEN	S	78			9074	LOC_Os06g40480	RLASSRAQTPPGTFEERLAQPSARP	T	1569
6124	LOC_Os04g08450	GNEDDDDIDGNNSFCSSSDMGDKDM	S	100			9077	LOC_Os06g40950	MGQFSTSPFGKMTFETATKFIVEAA	T	299
6127	LOC_Os04g09590	VITKSSPELVGLSTKPAPPPPGDIS	S	17			9080	LOC_Os06g41440	REVYRPGDSVTATIEICTPAGLKDD	T	56
6130	LOC_Os04g11810	CVSCTGEGVAFVSATASCALDDVKL	S	104			9083	LOC_Os06g42190	ATRRARWSEVALTFDQTDHPPCVAR	T	595
6133	LOC_Os04g13470	ILLKMLSEYVDISKCLPSLSFEVVQ	S	742			9086	LOC_Os06g42850	FRKFIVASLLIATASQFASLLLTTR	T	280
6136	LOC_Os04g14080	ALPAGVGQCDGKSCRWEGAPLAVGR	S	26			9089	LOC_Os06g43460	QRISKCYVLVEGTLYRRAANGILLK	T	1580
6139	LOC_Os04g16180	VHKDYKCSSPELSKYLAEVRKLKKR	S	86			9092	LOC_Os06g43840	GSLRTALLKNAKTLDKRKRLIIAMD	T	938
6142	LOC_Os04g16450	HVSCSPLTATLCSCVHFTKCLCLHM	S	255			9095	LOC_Os06g44890	ENSSQETPVYVITEGSEPQFFTRFF	T	752
6145	LOC_Os04g18010	DQSDIYCIICYESGKLEIFEVPSFR	S	248			9098	LOC_Os06g45120	PGGDFSDPVTSATLSIVQVFWGLDK	T	425
6148	LOC_Os04g18280	LRGKRRASRGRGSHCDAGRGDGTAG	S	150			9101	LOC_Os06g45710	WMGLDIGPDSIKTFSETLDTTKTVI	T	307
6151	LOC_Os04g20350	DVQCVTGCMAALSRFISRLGEKALP	S	1055			9104	LOC_Os06g46366	VPGTPSEGQQDNTSTLRSFSFRKVI	T	119
6154	LOC_Os04g22180	FPLKYLPNDEVLSLGNARGTFIQWP	S	58			9107	LOC_Os06g46450	SLLHDDVLDDADTRRGVSSLNCIMG	T	197
6157	LOC_Os04g23330	PPDAAPAADVTGSCAARSRRRRTAR	S	127			9110	LOC_Os06g47330	QLVSLAQAGDEITQDSLKRDDLGSL	T	90
6160	LOC_Os04g24170	TAVSESACHVSKSTKGQVPGSSKLG	S	426			9113	LOC_Os06g47950	KMVGSGFMPRTRTTMLLMKVFCENA	T	375
6163	LOC_Os04g24410	NAGDGAGEALRKSRNDLKREARRAV	S	72			9116	LOC_Os06g48640	QESVEATAEVSKTFDEKIRNYCDVT	T	597
6166	LOC_Os04g24700	FLLQIDPKYPDESRYVMHDLIHDMA	S	478			9119	LOC_Os06g48950	KQARPQTEFFCKTLTASDTSTHGGF	T	124
6169	LOC_Os04g25750	PRDVFAIEGTSASARADAEAENQVV	S	47			9122	LOC_Os06g49500	NVLAGTWSSNSYTPQWLTSAMLQYG	T	216
6172	LOC_Os04g28040	NEEVVPNEDISTSVHSDVESLGNPV	S	451			9125	LOC_Os06g49800	ISSELTTAASQDTPNHATKELDGED	T	447
6175	LOC_Os04g28210	SGPIPSSVSNLKSLKNLGVAAAGDS	S	374			9128	LOC_Os06g50490	IVSPRTVTTTIPTSTSADPTSYLEW	T	295
6178	LOC_Os04g29950	VWNNISSYCNDTSQTIMDVVSFADP	S	109			9131	LOC_Os06g50670	RSVFHPGSWRITTWSMPIPVTSWDD	T	332
6181	LOC_Os04g31090	RRRRRLVGVGGGSMEMESPRSPEAE	S	82			9134	LOC_Os06g50742	LRGVAKLDPEKNTLYSMRCYLATGI	T	453
6184	LOC_Os04g31330	VPDPQLREELKISISENVIPAYRAF	S	630			9137	LOC_Os07g01870	KAKVAYERRKQLTRLRVKAEKAAEE	T	180
6187	LOC_Os04g32920	AEKVSGGKHHGGSAVSWAVTLHLAF	S	48			9140	LOC_Os07g02230	SEQIPDFDLLYPTELLNSIDTNNFP	T	1249
6190	LOC_Os04g33110	HAGSFKCRLHRSSSHGHPSSSPTAA	S	78			9143	LOC_Os07g03230	PTKCIIFHNVDQTRLQGLLFQMSDK	T	800
6193	LOC_Os04g33260	GGGGGGASVRRKSVSVASASSASLG	S	189			9146	LOC_Os07g05120	KGSRRRLSSMQTTRCLSSTPNVSIP	T	265
6196	LOC_Os04g34400	DLEDAKNLKLVLSAFERLSGLKINF	S	155			9149	LOC_Os07g06175	YEAQAELPDPTFTIRQLIDNFARKN	T	164
6199	LOC_Os04g35080	IQAPPPQPPPSESIQIETGKEHRIT	S	113			9152	LOC_Os07g08330	QPYAVSRRAGHQTSAESWGTGRAVS	T	66
6202	LOC_Os04g35130	GVRLRRHPSMANSKWICYNVDDEGF	S	37			9155	LOC_Os07g08930	SVNLALICLGLLTSLALSIFSILAP	T	160
6205	LOC_Os04g35330	LVLRCVQTCAGLSKLSPMWEGPYRV	S	1837			9158	LOC_Os07g09890	QTSIDSGILIKWTKGFAVSGTAGKD	T	204
6208	LOC_Os04g35700	YMMNETRRIPTISDTWQDISQLKVQ	S	687			9161	LOC_Os07g10400	SAAAADQCHAAATKLGFLASNLFAT	T	40
6211	LOC_Os04g35920	RGPPRGLKEMEISASTSDGKPSKRA	S	475			9164	LOC_Os07g10600	DNTFDAVYAIEATCHAPDPVGCYKE	T	227
6214	LOC_Os04g36790	TQMELSQVKDHSSSPANAAAPPGAT	S	325			9167	LOC_Os07g11290	VRHRPNSRFLTATVRGVQQANRVVE	T	60
6217	LOC_Os04g37540	IIPAEIYDGSMGSNCSDDETVMLFE	S	553			9170	LOC_Os07g12250	SALREIKERIKKTKDEKKAKKAEVA	T	122
6220	LOC_Os04g38260	ATVEVVISEVQTSFDLCLSCFTSGL	S	349			9173	LOC_Os07g14800	NDALRRKNDKPKTGGEKKPAMDAPE	T	574
6223	LOC_Os04g38290	IEMTGPKRGIDFSCAVLVEYDMRIK	S	313			9176	LOC_Os07g20150	DRITIVRQLWDLTGDVLVLLEPGTP	T	251
6226	LOC_Os04g38630	VKQIAGRAGRRGSIYPDGLTTTFLL	S	473			9179	LOC_Os07g20580	TASVRQTVMFSATWPPAVHQLAQEF	T	281
6229	LOC_Os04g38910	PISIPPSQAPALSPEGAVSAVGRGR	S	238			9182	LOC_Os07g22640	PQGRLTVTVVKATSLKNKELIGKSD	T	272
6232	LOC_Os04g39090	SARSMDDLLKLDSSREAYSSWSGNL	S	183			9185	LOC_Os07g23290	PETRTGRSVFDLTKKKPVKRKKRTD	T	416
6235	LOC_Os04g39629	SESASKRLRSVVSTMPSDSPLDVRL	S	244			9188	LOC_Os07g27520	STDPEEFLQVYSTVLYAAGVDDNVL	T	296
6238	LOC_Os04g39740	TGSGVAASAAPGSAARSGRRDRRRK	S	383			9191	LOC_Os07g30250	ERMFLNRNRFKQTLPIHMAIGIMCL	T	255
6241	LOC_Os04g40400	SRPNSAHSSRPGSPGSQVSAVGSEG	S	337			9194	LOC_Os07g32480	MMLHGTQMSIQKTPRPDGSYMYQPT	T	274
6244	LOC_Os04g40660	DDIMKGFILLLQSAEDTALDIVDAP	S	467			9197	LOC_Os07g35580	DQTQDVTNRVVGTYGYMAPEYAMRG	T	529
6247	LOC_Os04g40874	SFNSLSRDLELPSEQGCLSVIVLGA	S	25			9200	LOC_Os07g37610	PSGKGPKHKQTCTCNVCMTVRRRFR	T	841
6250	LOC_Os04g41160	SSSSSSTATTASSSTAAGVRSPAKS	S	269			9203	LOC_Os07g37860	SPTRDVYCPIHKTKNHDLSSCKAFL	T	199
6253	LOC_Os04g41840	GLCDACVRNSKQSSLPGCPLLLMLW	S	1078			9206	LOC_Os07g38340	RIREEDIPKTAFTTRYGLYEFTVMS	T	919
6256	LOC_Os04g42030	HDEWDRVEQFDMSNQFSNRLNELDA	S	239			9209	LOC_Os07g39230	TNHITGQLDKLTTKERYKGTDQIHT	T	341
6259	LOC_Os04g42290	SEKKIIAPGSPSSTLLSEQKLRGVI	S	234			9212	LOC_Os07g41180	LPNHVDDNTHSVTFTSEPETSGNVN	T	173
6262	LOC_Os04g42600	APLNGKPIRVMYSNRDPSSRRSGSA	S	115			9215	LOC_Os07g41820	VQGHVLPNALDSTAIDWVNAALVPS	T	233
6265	LOC_Os04g43540	MEEADVKNKWENSSWGKKLIVQKRR	S	87			9218	LOC_Os07g42400	LASAMAAAWPNTTQRTCAWHVYQNS	T	428
6268	LOC_Os04g44480	QGAGAGVTLTSPSGDVLRYLVRLDF	S	1421			9221	LOC_Os07g42770	VHRDLKPENFLFTSKDENSQLKAID	T	136
6271	LOC_Os04g44800	RRRRSRSASSGSSSESESESESSYS	S	103			9224	LOC_Os07g43870	NTAMVELGDVQLTGHFAEQRFSKQP	T	198
6274	LOC_Os04g44870	GTDDDFVGEATISLEAIYTEGSIPP	S	89			9227	LOC_Os07g45070	PTVDPMVISNMKTVLKLISERVMAS	T	1322
6277	LOC_Os04g45930	GMYSSNFSGSYMSRGSDVGGSSYSS	S	739			9230	LOC_Os07g45970	FPQTYLGLLLSTTKLNFDAFAPVIS	T	1394

6280	LOC_Os04g46190	GGNSQPISHGPSSPDSLLQDAAKVI	S	190			9233	LOC_Os07g47700	ANLEVRIARIFNTYGPRMCIDDGRV	T	298
6283	LOC_Os04g46894	SSTRKPRSALLWSVLGPILNMPINE	S	1382			9236	LOC_Os07g49270	DEVVIFRDGTYLTLKEVFESLDLTG	T	404
6286	LOC_Os04g47330	LHAVYFLHPGLQSRLFFSTLGRFLF	S	117			9239	LOC_Os08g02340	DDGIPITSEKIATLVKAANIKVEAY	T	29
6289	LOC_Os04g47740	ARATEGASSSGVSHQHGRGDQPSVP	S	202			9242	LOC_Os08g03360	RFADTIFKAREKTLDQVAKKDPEKL	T	321
6292	LOC_Os04g48160	LRGLLGGGKKPNSGSGDPKPAREKK	S	20			9245	LOC_Os08g05180	EFRSLTQGSRSVTEYLHEFNRLARY	T	167
6295	LOC_Os04g49060	AETMDPPPQPPTSYPSSYTKFNSAL	S	75			9248	LOC_Os08g06200	AQSLQIYKHICQTNSLKSGKYLTDA	T	319
6298	LOC_Os04g49300	FNMIYRACDKSNSNINLRRMRRFRA	S	610			9251	LOC_Os08g08240	SIPIVLDPRFKITFIEFRLKRAFGA	T	627
6301	LOC_Os04g49450	TCTQFSNPQVVASDQQHQHQTSEAV	S	354			9254	LOC_Os08g08720	DNGLSILQYADDTIIFMEHDLEEAK	T	133
6304	LOC_Os04g49560	PSSPMSSAPTAFSGSSFARHYKGAW	S	313			9257	LOC_Os08g09160	REEALERVEGSATSTSTGDGEGRRD	T	52
6307	LOC_Os04g50204	QSPGSADRKKSASRITTPPEKQGKA	S	64			9260	LOC_Os08g12140	IENIELAKFRSKTIDRWWGEWKLHL	T	392
6310	LOC_Os04g51040	VGNTNSTVMSRYSGGCVSFCASNGS	S	151			9263	LOC_Os08g13970	RSGESSHAVVEETPLEGGQAENVWR	T	166
6313	LOC_Os04g51090	CSEADALSDEHKSLASITYNLIEGN	S	1888			9266	LOC_Os08g14230	RAVQYREIQGNETDKFLSYFRPCIM	T	107
6316	LOC_Os04g51120	YVGLSSTGITYKSSSASFGSNYSSG	S	174			9269	LOC_Os08g16559	DSLSSPHRRSQNTFFLSSPKKLQSS	T	16
6319	LOC_Os04g51610	DAPALHEADIGLSMGIQGTEVAKES	S	810			9272	LOC_Os08g17120	AKEAIKLSDYERTLKKASSRKSKPV	T	511
6322	LOC_Os04g52074	SMQQKERIPQCYSQMYSPESRWVLP	S	436			9275	LOC_Os08g19320	LESALKDVKGNHTRMWQAINILRYV	T	362
6325	LOC_Os04g52460	VAEKEGSSEEIHSLRKTLDGMIQRK	S	1444			9278	LOC_Os08g23440	CKIQVFCIAEEDTDAEELKADVKKF	T	831
6328	LOC_Os04g52700	SSSSSASTSSSSSSIGKRHRSPPSG	S	153			9281	LOC_Os08g25080	NKANDGEPSSAETEPSNGKTNINDT	T	135
6331	LOC_Os04g52960	AKEESSDDSSDDSSSDDEPAKKPVA	S	116			9284	LOC_Os08g27710	TTTTPTAAATKTTSDRPHRRRHQLL	T	28
6334	LOC_Os04g53060	LMILDDKSLSFHSLRSLTRLVIDGC	S	119			9287	LOC_Os08g29490	RAGRRTSGARSTTLTAIVSPTAARS	T	444
6337	LOC_Os04g53350	EGMEAHDAYLIASQLNLLNEQQDAT	S	207			9290	LOC_Os08g30060	KRLVKEIKQLNGTRSKVIENAAKRA	T	215
6340	LOC_Os04g53700	CRGCEEERYCSQSCADSDWETYHSL	S	235			9293	LOC_Os08g33082	LSQNNTEGSINTTPKQVKVQGCRKS	T	330
6343	LOC_Os04g54340	RFDSFEAFEEICSLAEQNKVDFVLL	S	40			9296	LOC_Os08g33320	NKLQVCQAPFDSTIAGNIPDLSEDK	T	455
6346	LOC_Os04g54440	AGAGTNHHQETPSSELKPRGTIYFP	S	32			9299	LOC_Os08g34230	APSTLYSLPPTVTQEYENVFQDEIP	T	683
6349	LOC_Os04g54670	SLYFTAESMTAVSEINKLVKTNWCE	S	204			9302	LOC_Os08g36760	KKHVTRGSEKNSTNIIEWKKKTVES	T	479
6352	LOC_Os04g54940	QQLSEKIAGLKISMDSAEKERDFYF	S	201			9305	LOC_Os08g37444	VTGSRPTKVTTKTNRGDTQGSGGGR	T	144
6355	LOC_Os04g55810	AVSRSLDNNGSSSLAYSSSSSKIDI	S	432			9308	LOC_Os08g39050	YGCPRTAKSLNATMKVLLRARLFDE	T	187
6358	LOC_Os04g56140	TKKTSANGLRDQSNANSGVESSGND	S	497			9311	LOC_Os08g40150	AAAPPQSVRMAYTSDGTPVFAPVSA	T	38
6361	LOC_Os04g56370	ACLLLLAAACAESARPLPAPAKAAA	S	25			9314	LOC_Os08g41260	SINPAEFHQVYTTGIEAAGGDDRVM	T	260
6364	LOC_Os04g56630	PPQPQPPPPSHASPHWYPGPSPPPY	S	34			9317	LOC_Os08g42520	MHILVPEEQKVLTKEERLPINNDIV	T	136
6367	LOC_Os04g56740	IRGHLVRRQAAESLQCLLAIVKMQG	S	303			9320	LOC_Os08g43060	VSPIRKFEHQEQTNEFKDEKVASGN	T	529
6370	LOC_Os04g57460	RRARTPPPPPRQSPRREKALERAEG	S	37			9323	LOC_Os08g43410	VSSSAVVMDPYPTPGPFGGAHFFHG	T	287
6373	LOC_Os04g58110	FKEMIRSAPLPMSPLESLASSAVRT	S	378			9326	LOC_Os08g44420	FLDEHTSAMEVVTKDAKRKWEMFAE	T	185
6376	LOC_Os04g58280	NLSSLIRQYGLCSRSTNEALLVIEA	S	101			9329	LOC_Os08g44870	AQASARVHKWGGTDETKPLLQGDHS	T	474
6379	LOC_Os04g58750	PTAFYLQAAALLSLGMENEAQEAIK	S	333			9332	LOC_Os09g03610	AMQQLQSPPQAQTHPAMQPVQQIPQ	T	681
6382	LOC_Os04g59040	ADDPEYRHYNDISELSPHRLQEIKR	S	156			9335	LOC_Os09g07300	LSVAGYEDCQVLTLNSRGEVTDRLA	T	1894
6385	LOC_Os04g59320	CPEGQSPMFQRYSYKETMKATNNFS	S	321			9338	LOC_Os09g07750	ERRRNGRRAAHATEGASSSGVSHQH	T	193
6388	LOC_Os04g59460	SFSKRTQPSHLFSGNRASNSLKSPV	S	1278			9341	LOC_Os09g09360	GGTCRVAAVLVFTAWIALTALTRLL	T	17
6391	LOC_Os05g01060	EHENVGTTSPSSSNQIKKDVVAQGT	S	430			9344	LOC_Os09g11020	LHSTPRCGCVLVTTRHQDVARSIKA	T	443
6394	LOC_Os05g01540	TSYSRSPASPSSSPSRSRSPPRPAG	S	76			9347	LOC_Os09g12130	RVQPEGGDERLGTSAPAPPRQRIVT	T	749
6397	LOC_Os05g01610	VLRASISSAPSTSSHGSAQDDCDSL	S	22			9350	LOC_Os09g13630	DNNCSGNHVEGSTLQKLEGKCFWGK	T	172
6400	LOC_Os05g02110	YCTTSPSTSGASSSRQLGPRTDKQI	S	136			9353	LOC_Os09g14960	KAPSDQGTPRSITGPGEQGTDNAEP	T	107
6403	LOC_Os05g02400	NKVAAVNSDMEKSYGGSSTIPSATS	S	496			9356	LOC_Os09g15770	AAGSDEDHSNSPTRSHDGDNVAADE	T	219
6406	LOC_Os05g02650	LQRQLEAAQSARSSVRLSERNVVEL	S	20			9359	LOC_Os09g16458	AGTSTLKVSGTITYNGHSMEEFVPQ	T	194
6409	LOC_Os05g02680	DVLSLDAWNTLLSEEERQRLAALLP	S	131			9362	LOC_Os09g17200	PLVAILEGITRGTQKYLCKRYSMAS	T	683
6412	LOC_Os05g03190	SNPNPPDLTPSASLEVNVSVPPPPP	S	46			9365	LOC_Os09g19400	ILKDAVQVAGNSTILNIKWQDNTGR	T	252
6415	LOC_Os05g03320	GDLECRPYDCNASNSVPKCNYEDSG	S	425			9368	LOC_Os09g19910	VSMNKIETHKNETHGTSDDINENTP	T	86
6418	LOC_Os05g03460	AAVAAICIEDEASVRPYMSDIVVAL	S	351			9371	LOC_Os09g20060	NDRQGMVNYIYDTLWSHRDKEYIMY	T	292
6421	LOC_Os05g03550	KQRSKSHKRTDQSNRSSQSGSQRQR	S	634			9374	LOC_Os09g21110	VFANEINIAVIETEKSYNAFMFRDA	T	809
6424	LOC_Os05g03780	DQMLDKVIGYIKSEYNISHVTIQIE	S	404			9377	LOC_Os09g23790	LTPSDPEANPVLTRPGKEQGEEAPE	T	1225
6427	LOC_Os05g05220	KGDHKAKKRSTASVIPAVEVEAPGC	S	165			9380	LOC_Os09g24440	GYTEIKVQAENDTFALHADNMTIRN	T	58
6430	LOC_Os05g05300	DRSRNYTSRGSRSSIEKPRNSATAS	S	473			9383	LOC_Os09g26540	SLISSVDGKVLFTCSGTMVDHVGSA	T	87
6433	LOC_Os05g05370	PLEKWIFHKTKESVFLVDFVPKKDA	S	182			9386	LOC_Os09g26820	FHDLPLSSSTITTATHDAPFAEAVK	T	274
6436	LOC_Os05g05720	RALSAYKQAMQYSKNIPRQATSNTR	S	73			9389	LOC_Os09g27810	KLPTRLKEVQKLTGCMAALSREKVQ	T	252
6439	LOC_Os05g06350	AVPIFVKLLSSPSEDVREQAVWALG	S	177			9392	LOC_Os09g29630	MDEQQNGDVDNETSNGKSSEEAKVI	T	592
6442	LOC_Os05g07000	SPRRRRSPSYGKSGPPSHWGSHGAD	S	175			9395	LOC_Os09g29810	EAECGRKLRVLRTDNGGEFTAAEFA	T	770
6445	LOC_Os05g07350	KPRRQQGQAPPRSNNGGKDVIRGRV	S	668			9398	LOC_Os09g30412	AEINQLLSLIINTFYSNKEIFLREL	T	24
6448	LOC_Os05g07680	TSSLQKDLDEVKSEKEALEAVVLVN	S	242			9401	LOC_Os09g31486	EGLIAVFDLGGGTFDVSILEISNGV	T	250
6451	LOC_Os05g08300	KKRSHKSGKRKPSGEVLAAEQMDPP	S	97			9404	LOC_Os09g32540	LAEASVETKEKKTTAKERVLSFRRR	T	898
6454	LOC_Os05g08790	KIAEAEAVSKVLSDELASVRDVLQK	S	501			9407	LOC_Os09g33450	RVPVDQGLQQTRTPARGFMSPNMGK	T	324
6457	LOC_Os05g08970	KRKPKGGDAAEGSEKRKPKKKKDPN	S	544			9410	LOC_Os09g33940	NSKEQEYNIKGSTQKKIKRDIKVLG	T	320
6460	LOC_Os05g10520	VRAEITLLLDASSLAPIGQQQNCHV	S	428			9413	LOC_Os09g34060	EQQVHLQDALNDTLKSEVQRLKVAT	T	264
6463	LOC_Os05g10770	LPKGVLRGCAECSDCQKVIAKWNPA	S	132			9416	LOC_Os09g35710	PSGNTESAQPVYTVDSPPSSSQPQG	T	315
6466	LOC_Os05g11730	KHELANSSQELISRLIPEHVRRQAT	S	382			9419	LOC_Os09g36360	DAKLNPAPAPAPTPTPPPPGILESL	T	133
6469	LOC_Os05g12100	IELGEVYLYGFASADVAEAAARGGC	S	87			9422	LOC_Os09g37310	VDGPRLLPASPVTPSSPRVSALSPP	T	541
6472	LOC_Os05g13910	AWRKQFWQKDGRSAAKARLLEQLAK	S	784			9425	LOC_Os09g38390	VSMKLYTEAIEDTITTEEAQEVFQL	T	391
6475	LOC_Os05g14940	PMSMVSSGNSIFSAGTGTRMLDDYR	S	672			9428	LOC_Os09g38970	WLNNCVGRKNYFTFISLMAISLFWL	T	223
6478	LOC_Os05g17940	DDWRTLLNKFLSSDELPEDDAEAEK	S	138			9431	LOC_Os09g39380	STEIVKADLASKTLTSAVGATFTYE	T	117
6481	LOC_Os05g19020	RSDTSLFIYHRGSDMVYLLLYVDDI	S	170			9434	LOC_Os09g40040	NNSHFTLPHPVPTWPGREQGGEAPE	T	1033
6484	LOC_Os05g19370	RGKGHTHHKARPSRRATWKRNQTVS	S	187			9437	LOC_Os10g01570	LPTMVLPKYPLPTHNVVVRGGAPNA	T	84
6487	LOC_Os05g22870	AGDTEKLMETWRSMINEKVPGTRVI	S	575			9440	LOC_Os10g02630	RIGGENAEQKLSTRDQQHAGRPRSE	T	248
6490	LOC_Os05g23320	IASQLKMHCIVGSPGTSFKIPDAEI	S	439			9443	LOC_Os10g05690	IWSFLGLLMTTYTAWYIAVASLIHG	T	222
6493	LOC_Os05g24160	INNTSYGSLPGFSNRKAAEQSAAEV	S	85			9446	LOC_Os10g07270	SSSIGDEPELQRTLREGDDYALVPQ	T	104
6496	LOC_Os05g24430	QYEHTSYRDPNRSLTGLGGPSDGSY	S	16			9449	LOC_Os10g07510	QSELNYLQGHLSTMELPSPPPYVAG	T	136
6499	LOC_Os05g28190	KDGDESETKDGSSEEQKDADNKGQS	S	234			9452	LOC_Os10g12410	RAHPEGSMIEEYTTEEVVECCIDYM	T	581
6502	LOC_Os05g28860	IKLYEDDPKVLDSCFRRIMCSSNIL	S	444			9455	LOC_Os10g14120	VQRGGLRKTGHDTLIHKKDFSVKQQ	T	686

6505	LOC_Os05g29010	KILDLIPDENVASELHDKWQGNRRS	S	88			9458	LOC_Os10g16480	MAGSVTRRGLDMTGFVLELRGMCVA	T	35
6508	LOC_Os05g30340	IQSAQDKVLSLRSSLKKAMAKKSKG	S	260			9461	LOC_Os10g20180	VYYVSEALHDAKTRYPQIQKLLYTV	T	235
6511	LOC_Os05g30810	VVRRAVENWQKSSTLERNLGSGSFA	S	64			9464	LOC_Os10g20540	SFCLDEARNWGGTAGYVDQYWCKGQ	T	55
6514	LOC_Os05g31490	MEELQKCAVHFRSEFLSKLLPSSSS	S	632			9467	LOC_Os10g21310	VYWDLEIFCDERTGKPSLDLPKIFG	T	128
6517	LOC_Os05g32070	RRERQQLAGAASSPPTSSAFPAATT	S	162			9470	LOC_Os10g24120	VEDRVDTPGYTPTPSPGHNETGVES	T	437
6520	LOC_Os05g32580	SPSSSSSSSAAGSSSSSSGASSSSG	S	46			9473	LOC_Os10g25320	SLQTLLEDIPVSTALSRSASSAGDS	T	148
6523	LOC_Os05g32760	WDYFFSMDEGMASIAPDDDEIIQEP	S	248			9476	LOC_Os10g28200	APTMKLKDGLRFTYFWIKEQIEKEK	T	333
6526	LOC_Os05g33100	PEVVKQFEQVLSSKSGIGHFVKIFP	S	191			9479	LOC_Os10g30580	ANFISVKGPELLTMWFGESEANVRE	T	555
6529	LOC_Os05g33440	RFRSNADAEAVVSLLRGHGFSDANI	S	92			9482	LOC_Os10g32920	LPVAATVNCADNTGAKNLYIISVKG	T	32
6532	LOC_Os05g34500	EQRRLWLEQEVRSPLHPSAAAAGGQ	S	46			9485	LOC_Os10g33830	NPIELGFLIKICTVVEDPHVTNIFF	T	55
6535	LOC_Os05g34620	RSGRRDRRRKKRSAHSDDEGHVLAV	S	430			9488	LOC_Os10g34580	KPMEEDVKVIEKTATMRGFKVEGED	T	420
6538	LOC_Os05g35800	DKTVIVVSAVVGSLGLLSAILGFAA	S	14			9491	LOC_Os10g35280	SKNLLTEKKKTKTPKEKSKKRAHNH	T	652
6541	LOC_Os05g36160	YGEAAPPPGAAGSAPAPPPAGEAAG	S	69			9494	LOC_Os10g35870	TAAAAEPEKAPATEEKPREVSSEEV	T	201
6544	LOC_Os05g37330	IAAGREKFASVPSGGGGGIAVAAPT	S	67			9497	LOC_Os10g37280	GATNDANSSRGTTTHDKEDASSTIS	T	566
6547	LOC_Os05g37500	SPATLQLPTCLTSESPPLAHLLHSK	S	1904			9500	LOC_Os10g37640	GSQLIRQVSVEDTDTSDPMADPESS	T	191
6550	LOC_Os05g38530	GEEKQFAAEEISSMVLIKMREIAEA	S	124			9503	LOC_Os10g39140	AAEAEQQHQLLSTAVHDTMPGKYVR	T	14
6553	LOC_Os05g38730	IQPPPPGTTSWKSNSSSSIAPPIQA	S	185			9506	LOC_Os10g40540	TFRIVTDRTVFATPEVHIGFHPDAA	T	177
6556	LOC_Os05g38950	ELEDTSGSRSSTSDVAGENTEVSSE	S	240			9509	LOC_Os10g41290	VGQQLRFPDHPPTSNAGRDLIRGLL	T	538
6559	LOC_Os05g39520	VNADAAAGEGGGSSESPRLDADKGA	S	104			9512	LOC_Os10g42110	AMQAQVCMPEWPTAFYLQALALSKL	T	474
6562	LOC_Os05g39730	TASPSIFIMESPSAPRRSPGPSPLG	S	516			9515	LOC_Os10g42210	DGRQRRGARGDGTVARRRDGDGDGA	T	132
6565	LOC_Os05g40060	PEGDGEESGVKMSKEEPVDSCPGPS	S	265			9518	LOC_Os11g01300	SGGGGSNMLRFYTDEAPGLRLSPTM	T	63
6568	LOC_Os05g40230	VCDNTLDARVEISFKQKLPEIRKKL	S	212			9521	LOC_Os11g02080	EHKLASIGGLWATAVGASVAYGRRK	T	26
6571	LOC_Os05g40960	LQNHGVLKAYDDSTLFCRKQSQDQE	S	277			9524	LOC_Os11g04070	QVLNIPTKINKGTVEIITPVELIKK	T	154
6574	LOC_Os05g41100	LASDDKDLNQYVSMKKIAPYREAEW	S	553			9527	LOC_Os11g07040	HYTERAAAQLIRTIVAVVEGCHSLG	T	154
6577	LOC_Os05g41500	GGSSFSFHARSFSGVETTPKFGSFN	S	95			9530	LOC_Os11g08390	LLDLDLPPASGWTMELVSQLINVWS	T	382
6580	LOC_Os05g41730	HGAAAAAPSAPTSFSFPSSPASAST	S	167			9533	LOC_Os11g08470	AEDMGTGSKNNNTITNHSGRLKKED	T	523
6583	LOC_Os05g41790	GNDPVPPTIDDTSKTGGLHLHGGHV	S	80			9536	LOC_Os11g09329	SKVQILTLYVLETLSKNCGDVVYQQ	T	66
6586	LOC_Os05g42220	SIDSAQLGLRSRSFGNLSSLDGAPY	S	214			9539	LOC_Os11g10060	VVQKYQSAVQNSTNLSTQDMQNNCN	T	495
6589	LOC_Os05g42300	AEEPASFDAGKVSGGSVSESRGVEE	S	61			9542	LOC_Os11g10510	PAKFEQAKKFGCTDFVNPKDHSKPV	T	241
6592	LOC_Os05g43380	QDFIVERSPRLQSPISKESSFQQTP	S	247			9545	LOC_Os11g11710	EDEKLLAIHASLTQLLRGPTGTRRP	T	155
6595	LOC_Os05g43520	SNKAVAEISKIASSLLPFGQGDADE	S	149			9548	LOC_Os11g13640	EYIALVVEGEDTTLWDNETYIPDNW	T	212
6598	LOC_Os05g43570	LSDFGLCKPLDSSNFPNLNEPDYTS	S	267			9551	LOC_Os11g16400	PADQDKQHPKRETKTKITSNSKTKA	T	188
6601	LOC_Os05g43950	SDALALYRLEYKSSDTKRSVKSKLA	S	497			9554	LOC_Os11g16730	SRDEWDKFVAKMTTPEALERRKKMS	T	181
6604	LOC_Os05g44310	FDEIDAICKSRGSTRDGTGVHDSIV	S	339			9557	LOC_Os11g20040	AAIVDGHPSQVPTVLAMGSRHCVEA	T	127
6607	LOC_Os05g44380	SPFFRDLASPIPSHRAASRFASSAA	S	34			9560	LOC_Os11g20290	LPVTFGGSANFRTERVNFDVADLSL	T	574
6610	LOC_Os05g45060	HSARHAVSSASSSGHISITVPRVIF	S	67			9563	LOC_Os11g23750	RRTPASCGSTGFTKVRKDTTYTCKI	T	609
6613	LOC_Os05g45670	EPKPIKSLLIYDSYQKGSENAEVPN	S	385			9566	LOC_Os11g28270	SPDNVSGRSRRITHSDSQQIPSGER	T	425
6616	LOC_Os05g45830	AGASGALGATYGSPYITVDGSYYPR	S	647			9569	LOC_Os11g30970	AWVEGQAFAPGKTVVPKPVDNRTVV	T	194
6619	LOC_Os05g46340	QAASEAKTMTKSSSEARKDTAAEDA	S	322			9572	LOC_Os11g33120	LLRAEYDRDGAMTNPSFPKVLIDGP	T	725
6622	LOC_Os05g46500	GGGAAARVERSRSAGHGGYRAAGRV	S	49			9575	LOC_Os11g34200	ETIHGTESVPPSTHPPAEAPSAAEI	T	194
6625	LOC_Os05g46620	DYVGQLALASIASVEVEENLNSQEA	S	110			9578	LOC_Os11g34600	MVLRPSQPIIGVTPGHTWPLGHINL	T	663
6628	LOC_Os05g47470	LGAEAYRLEGIVSQAREDIATAEHA	S	84			9581	LOC_Os11g35590	PEHLQQIAISIETLLDVNELSLEEV	T	275
6631	LOC_Os05g48220	AYVYKAKTKSNDSTIRCIWGKVTRP	S	66			9584	LOC_Os11g36070	KDKESKRTRKRKTNSWLCCLTPSTT	T	536
6634	LOC_Os05g48820	DDEDNSASQSEESSEENSEPMNEEY	S	1748			9587	LOC_Os11g36390	KYFASKTEKEEDTSAGKGTGRGLPK	T	73
6637	LOC_Os05g49160	DTTGQVRPEVDGSDEKVEIANQNEK	S	20			9590	LOC_Os11g37100	NKAAPGLPSGEFTISLYEVLARVHG	T	64
6640	LOC_Os05g50280	LSNVGAMVGAIASGQIAEYIGRKGS	S	119			9593	LOC_Os11g37950	NKPLSWRQKYGWTAFCGPVGPRGQA	T	73
6643	LOC_Os05g50560	HQVPARMNCVEQSQAMDAYKMHSQS	S	258			9596	LOC_Os11g38540	APSRERPTGKWCTVHNTSLHDLADC	T	470
6646	LOC_Os05g51070	IALAAAAATAERSRLVFVGKGAAYS	S	342			9599	LOC_Os11g40120	SALINHTLRLPPTHPRIVRVSNSPI	T	32
6649	LOC_Os05g51490	ISLLDPKRLASASYQAFRSNLSHGT	S	275			9602	LOC_Os11g41890	RTKKIFVGGLASTVTEADFRKYFEQ	T	116
6652	LOC_Os05g51830	DSDEDDSEDEDLSPEDDDDDSSEDD	S	172			9605	LOC_Os11g43610	QWGGRELEQQIRTSITLRKLLPSPN	T	247
6655	LOC_Os05g51850	GRPRKDKPLQSWSVLSGGAAMTKRG	S	322			9608	LOC_Os11g47030	SDPLGVLASNWTTKVSMCRWVGVSC	T	68
6658	LOC_Os06g01650	NKWLDEITGGYDSSIREFQGGDDQK	S	411			9611	LOC_Os11g47451	EKIKAIENMKSPTRLKEVQKLTGCM	T	184
6661	LOC_Os06g02028	LLEEAMGKSTAESTAKHSSINCIVT	S	180			9614	LOC_Os11g48030	TREGEVRFPEISTPILEKICQYFYW	T	80
6664	LOC_Os06g02370	RAGGSFAAAFLGSFLGIIVLIFGNV	S	531			9617	LOC_Os12g01940	VTEVDDDTTMEGTSSAPYPLLQVDN	T	249
6667	LOC_Os06g03910	LLNPDTGCRMLPSPGGCDEEIGLFL	S	233			9620	LOC_Os12g04000	GRAAAMPPASVMTRLILIMVWRKLI	T	230
6670	LOC_Os06g04690	LSNCSFHPTVTLSGLRSLMRLYLCC	S	215			9623	LOC_Os12g05000	WGEELPVSAFCRTSDGEWDAFRSID	T	93
6673	LOC_Os06g04950	VATVASAVPTLASVRMLPWAKANIN	S	45			9626	LOC_Os12g05420	GPKGTKWIFVMSTTKKLYAGKKERG	T	225
6676	LOC_Os06g05020	SFENAPEHLKNTSFQGAGRPHPAFF	S	101			9629	LOC_Os12g06380	DSEALVGYFKRRTIEQGMFYWDVLV	T	257
6679	LOC_Os06g05190	SAGIEAKHSDVTSKQFSPTKIKQWA	S	190			9632	LOC_Os12g06780	GLLKEIHVLEKSTQKLTDAIDAAPI	T	313
6682	LOC_Os06g06014	CRVLGWDIDRMPSVVIDPRGTLGQA	S	234			9635	LOC_Os12g06980	VSAMLDKESNGLTTEMMPEAHIRRS	T	576
6685	LOC_Os06g06830	VRHSAATPSSTPSTREDLRGGPDAR	S	91			9638	LOC_Os12g07300	FCMGLQDKDIGATKEELGIEREQVL	T	514
6688	LOC_Os06g07820	ASATTAESPRAASSTASSPSPSPAA	S	116			9641	LOC_Os12g08800	NTIMPEKRNRWSTAKSWTPCPIGMI	T	476
6691	LOC_Os06g08280	GHQLNVLQNVPVSNNLGVPAKPHIS	S	761			9644	LOC_Os12g10410	ENPPDHVIFPRLTYLDLSDLPELSD	T	857
6694	LOC_Os06g08480	TTSPKDPATEVPSSASKEATPPVQD	S	1215			9647	LOC_Os12g11750	EVEEERETALIATAVFNEARDDLRL	T	543
6697	LOC_Os06g08850	LDAICERLVSSLSTVGTYIVREGDP	S	493			9650	LOC_Os12g14110	DPFAAIHQHLLSTQPPSARKESNSA	T	128
6700	LOC_Os06g09570	LEQIRQHMARLPSIDPNTRTLLICG	S	163			9653	LOC_Os12g16060	DKLGFPLSQELATDLILQSLPPSFE	T	212
6703	LOC_Os06g10710	GALFESLRGSISSHIDQAKVYYKLK	S	138			9656	LOC_Os12g18630	LPGWLSRGLVGRTLADPSFPHKIAF	T	156
6706	LOC_Os06g11610	GKEHVKVWAEQNSLVIKGEGEKDPE	S	180			9659	LOC_Os12g19040	KKKLKVANTGKHTGCSNEDKAHQDQ	T	70
6709	LOC_Os06g12160	RNEFMAAWDGLRSKENQRILILGAT	S	818			9662	LOC_Os12g21798	KNVLTNFWGMSFTTDKLRSLVKKWQ	T	106
6712	LOC_Os06g12590	ESFLSKYQNVSSSLRASIRSAVMPL	S	294			9665	LOC_Os12g22000	NEEVAEAADDIPTIDPDVEEFLANE	T	482
6715	LOC_Os06g12780	NDKGKCVGIAFQSLKHEDVENIGYV	S	336			9668	LOC_Os12g23790	IAATATETATAATEPAAAATEPTAS	T	57
6718	LOC_Os06g13820	ASLLADDDPSDDSAPRRPSTFLNAV	S	30			9671	LOC_Os12g25710	HIEGFAPEVAWVTKSGKSDLEAPIA	T	118
6721	LOC_Os06g14406	LHSEDNSKSDAMSTGQNIRKGKQPG	S	226			9674	LOC_Os12g27994	AHLPLPGPDSTPTSSASTVAQNQLH	T	76
6724	LOC_Os06g15420	IDGLKDHANEHVSDSMMMNASFVYP	S	474			9677	LOC_Os12g28640	SVLWAVRTTLTTTNKETPFFLVYGS	T	368
6727	LOC_Os06g16280	PVDATAPFVASASCCCRHPVHQQHL	S	117			9680	LOC_Os12g31350	QKNLLYLAAIADTQPQTTISRPQMV	T	69

6730	LOC_Os06g16430	DRTKKKSSSHKSSEQQVETADSLLD	S	434			9683	LOC_Os12g32440	PVSPHVIKIIGYTESLDKLGFPLSR	T	223
6733	LOC_Os06g17100	RRIILEDVWEPASTSLGAKKYYVSF	S	338			9686	LOC_Os12g36510	IVHQALKKYQDYTGQLLSPAKCSLL	T	152
6736	LOC_Os06g18130	WAAWHYDQKGVFSVKSAYKVAIQNK	S	53			9689	LOC_Os12g37560	DMSAPFSHYYIYTGHNSYLTGNQLN	T	125
6739	LOC_Os06g18830	STSSDVSDESACSSISSVTKPHKAN	S	161			9692	LOC_Os12g37720	VCHSKAWDSKIVTSAWWVYPYQVSK	T	658
6742	LOC_Os06g19660	ITPSEIISGVLSSPETIATGSSQNV	S	749			9695	LOC_Os12g37870	APLIATLPLEVRTKIGDFIRGVYSV	T	176
6745	LOC_Os06g19980	DTWRRIKPKNLKSVARFCISIKWKD	S	472			9698	LOC_Os12g39090	SPLGKSPLGHQGTGEFVSAGSKAPL	T	149
6748	LOC_Os06g20490	DEAGNFFQGDPLSPILFNLVADMLA	S	50			9701	LOC_Os12g41140	SAVNVRTPGDIVTITAAAATALRGA	T	265
6751	LOC_Os06g22700	RTHCNNCNKYRYSREVCEPGHSPHR	S	114			9704	LOC_Os12g42260	NAAMSKQLKTVKTPPKKDLPQLTPP	T	191
6754	LOC_Os06g22820	GGDSDSDFDEFGSMDVTKTDKGFSI	S	464			9707	LOC_Os12g43640	SGAVVAVKHITRTRAAAAAARSTAA	T	714
6757	LOC_Os06g24460	MWWSTDNGVLLRSRASSRGEEDDDD	S	13			9710	LOC_Os12g44360	SRHLLRGTRVPYTVALLVLGVALGS	T	37
6760	LOC_Os06g24810	NRIITDNGTQFTSELFGDYCEDMGI	S	1483			9713	LOC_Osm1g00580	IIERKSVHEPMQTGLKAVDSLVPIG	T	150
6763	LOC_Os06g25710	QSLSNVLLVNSLSYNLLSVFQLYKL	S	457			9716	LOC_Osp1g00600	SSPRGWFTFGHATFALLFFFGHIWH	T	457
6766	LOC_Os06g26340	AAIPAKDYKHVASSSTKLAVDGSWN	S	434			9720	LOC_Os01g01800	NKSNLDIVTQTPYPHIQSPMSSPVQ	Y	392
6769	LOC_Os06g27600	FDVHFVPRTAVKSQVLADFVANWTM	S	150			9723	LOC_Os01g06290	RGRSYSRSRSRSYSRSQSPRRDSRN	Y	183
6772	LOC_Os06g28100	NAGPTFQRTTRISLGSQLGRNVEAY	S	945			9726	LOC_Os01g08420	DITLPGLAELRGYWRGSLDASGGGN	Y	278
6775	LOC_Os06g29310	LKEEWNYLNKTGSLEDCHLHESVWK	S	389			9729	LOC_Os01g09620	EEAAAMAAAVDAYACDEFRMYEFKV	Y	72
6778	LOC_Os06g29400	KGAATDPEIESSSVQRPVDLYKAIF	S	717			9732	LOC_Os01g11140	EKVASTAATNNSYSIDTNWYIDSAA	Y	260
6781	LOC_Os06g29430	EASTNNAAIASTSESHIQARIEDSA	S	795			9735	LOC_Os01g12650	ATAIWILFELLGYHLLTFVCHGLIF	Y	125
6784	LOC_Os06g30020	PPQYSERYHSSDSREERDRGPPGAD	S	451			9738	LOC_Os01g16020	ERIRLPDEAMAYYDDLAEMYVGGDV	Y	224
6787	LOC_Os06g30320	EKKKAQLAEIGMSMLEDPESHIRSL	S	210			9741	LOC_Os01g16330	IFGVNFSDKSFIYLAGLQLLLSSGK	Y	158
6790	LOC_Os06g30710	AAQKFVRNAIAKSSRTIDESLRASY	S	457			9744	LOC_Os01g17320	HKLSYKNKIKPLYTTYLMLLDSYSK	Y	520
6793	LOC_Os06g30750	KFLGVIVALWVLSEVGSCCDFLTLI	S	196			9747	LOC_Os01g20110	MKNEVKDIMCHIYTTTKESLKSISP	Y	320
6796	LOC_Os06g31250	SPRQATPPPHGTSDLRDHLNGRREA	S	219			9750	LOC_Os01g21450	TSLYIKKHEILSYFFDKQNKGAETD	Y	281
6799	LOC_Os06g32060	WLSEVKFLGHVISAKGVAVDPETVT	S	970			9753	LOC_Os01g23590	YIRKEGLKQLIIYANESFWDQLMKF	Y	636
6802	LOC_Os06g34420	LQLLIHLKAANISVSEYVFRQELVP	S	396			9756	LOC_Os01g25610	VRSVQFGQKGIPYLNTYDGRTIRYP	Y	138
6805	LOC_Os06g34440	HAIFYEKNKTRNSYEIYPRKGEVWA	S	588			9759	LOC_Os01g27790	KTAFITPIGTYCYTTMPFGLKNAGP	Y	927
6808	LOC_Os06g35030	TVLGYYPVRVLPSKTAILPVNPKFL	S	250			9762	LOC_Os01g31510	NPVSPHVIKMIGYTESLDKLGFPFS	Y	132
6811	LOC_Os06g35814	QTDEGKAFAERESLYFMETSALEST	S	150			9765	LOC_Os01g33000	ESGDLHGRLLFQYLEFDSPFCREPL	Y	218
6814	LOC_Os06g36360	LVVPPDLDISQGSVLYDVDEVTVRS	S	189			9768	LOC_Os01g34290	AAMGTACKDLHLYYMEKSNVRKPSK	Y	1234
6817	LOC_Os06g36700	IEPLVQTCMTTLSSKIVNRCKRTLA	S	170			9771	LOC_Os01g35470	PTSKFFRGILNFYGISLHHLNPNSI	Y	81
6820	LOC_Os06g36730	TRDNENRSRHRVSSRRHDNEERGGR	S	406			9774	LOC_Os01g37790	ATKRKRSNIGQAYENSRKKDRRRPL	Y	293
6823	LOC_Os06g36920	FYTVSPLIARETSEQVEVGGYTLPK	S	423			9777	LOC_Os01g40050	VRNFVQLCLEGYYDGTLFHRVIKSF	Y	49
6826	LOC_Os06g36940	KEKTETKDKNKNSGDEKDEGDEDDE	S	312			9780	LOC_Os01g40750	FAVRDYLDPLRNYVFNRTRDDPVIV	Y	202
6829	LOC_Os06g37770	SGAGRPVGGEEASELRRERRSDSFR	S	31			9783	LOC_Os01g42410	LWYLFFMYFTLLYFTFYGMMAVGLT	Y	1317
6832	LOC_Os06g38960	EVTNEDKENMGDSEMPMDLGIAQEI	S	334			9786	LOC_Os01g43420	QLYSASEYCEKSYLHSEQKQMVLDN	Y	69
6835	LOC_Os06g39480	VTELRWKEGSLESGRQHLLRTIDSY	S	350			9789	LOC_Os01g46750	SGAAPLATHVEEYLRVVTCAHVLQG	Y	402
6838	LOC_Os06g39906	AEAIGDIADADESGEGSSLGSEAYG	S	123			9792	LOC_Os01g47490	RTPHITKDHDRAYFDSADWALGKQG	Y	69
6841	LOC_Os06g39912	MMRRVAADDGDDSEKTTKQQRKKKK	S	75			9795	LOC_Os01g49250	SEYGSTLDDDQRYADAAEVLAAAAA	Y	76
6844	LOC_Os06g40510	LEKPIIPVDLYRSIRETQLVGLSGY	S	86			9798	LOC_Os01g50320	LLDRVPPRPRRSYSAALLSAPSLDG	Y	61
6847	LOC_Os06g40704	VSVVGGRVKQEVSLRLQNVECGEVD	S	299			9801	LOC_Os01g51390	PIALDVLSDILQYPCFPANALQRER	Y	164
6850	LOC_Os06g40840	DDLSTKKLNEFLSELQALQKEKSNR	S	169			9804	LOC_Os01g54590	GQERYRAITSAYYRGAVGALLVYDV	Y	88
6853	LOC_Os06g41860	VIITRDSPDALHSHIIEVAPGADVA	S	79			9807	LOC_Os01g55490	AALQQKINKFLAYKRAGKSFNSEVR	Y	253
6856	LOC_Os06g42490	NQCTEEVDCHTKSTLSNSINLSTPI	S	519			9810	LOC_Os01g57630	ITERNMVPDAKNYNAKLRGLVAQGR	Y	272
6859	LOC_Os06g43170	DLSANYFNGSLPSDFGGSRLRFLNI	S	258			9813	LOC_Os01g59760	AHHHGQQGVQGAYFPNRLVPQPLNV	Y	207
6862	LOC_Os06g43460	FFLVYGAEAVLPSELTLSSPRATMY	S	1833			9816	LOC_Os01g61660	YFQAHRVTVVTSYPFGQILHNREGT	Y	1270
6865	LOC_Os06g43610	YQSRTHPLQIASSLSRESCEALHQS	S	208			9819	LOC_Os01g62310	SFMPVATNNASYYPQQQTPLLYPGM	Y	180
6868	LOC_Os06g44150	EIEAYPLLDELTSKISTLNLERVRR	S	205			9822	LOC_Os01g64250	CPVAWRVRVALLYKAAAPVHFTPSE	Y	83
6871	LOC_Os06g45360	PVVHSYSIVTLHSAFFYVDKRKVSV	S	231			9825	LOC_Os01g65260	GVVGDPDATSLCYLGLQKLQHRGEE	Y	102
6874	LOC_Os06g45910	IRKHEDYHRYNKSGERSSSRYDDRG	S	463			9828	LOC_Os01g67134	YEGNIEATGEDYYVEPADERRPFRA	Y	132
6877	LOC_Os06g46366	GDRAILLVPGTPSEGQQDNTSTLRS	S	112			9831	LOC_Os01g69910	SSIIDSPVARQQYRRMLKMHKQNKD	Y	834
6880	LOC_Os06g47350	FIKKEEDSISRMSAVSYVGSYLSRA	S	367			9834	LOC_Os01g71540	DLDKADEWVKIKYAWMAKSLTNDII	Y	66
6883	LOC_Os06g48330	DLAEALSHLKTPSANLDGGLLAAAL	S	167			9837	LOC_Os01g73950	HCAAGEVEKALQYFTEMVEKNLEAD	Y	406
6886	LOC_Os06g48530	HRGGGGGRGGGSSSPPPYRSGRRHS	S	94			9840	LOC_Os01g74370	KQMKQVVVGVVYYLCRQDGQLDHPH	Y	44
6889	LOC_Os06g48950	GAVGRSIDMSQFSGYDELKHALARM	S	1015			9843	LOC_Os02g01110	PQGHRRNENEVRYPVNDYQRPLQQN	Y	403
6892	LOC_Os06g49500	VIQAPVLIKYPASLSLTAYSYSFAT	S	263			9846	LOC_Os02g01490	SVSTWGQGGTSEYPPNMVFYAEYPG	Y	272
6895	LOC_Os06g49950	RTERVNFDVADLSLPYNAVLGRPAL	S	722			9849	LOC_Os02g02410	WLDENQTAEKEEYEEKLKEVEAVCN	Y	622
6898	LOC_Os06g50539	SHHYELLHSLMLSNSGDEHREEAQG	S	375			9852	LOC_Os02g03080	AKVALRRVELVYYKPQEVYDSMRKL	Y	439
6901	LOC_Os06g50818	ATGGAAGEKTASSLLLGVRGYTSTL	S	15			9855	LOC_Os02g03890	GVAIRSRKETGGYYAFVEFEELSGV	Y	353
6904	LOC_Os06g50890	VNHGNTLYVTGLSSRVTERELKDYF	S	73			9858	LOC_Os02g05330	DMGLQENLLRGIYAYGFEKPSAIQQ	Y	57
6907	LOC_Os06g51250	VPSTPMQKSVFDSVPSTPMQNSLFD	S	988			9861	LOC_Os02g07210	HYEGYVPMEYKVYLKKMKRSGEWGD	Y	141
6910	LOC_Os07g01020	ADIRAQGGVARMSDPGLIRDIKRAV	S	87			9864	LOC_Os02g10650	GQSTYDKASVHYYVQSHIQINEYRD	Y	122
6913	LOC_Os07g01550	DVYERMNFEDNGSIGCSDAADGGQR	S	291			9867	LOC_Os02g12310	NVDPWQLPAMAMYGSDHDRYFFTMA	Y	63
6916	LOC_Os07g01870	KPSHGPIHFRAPSRIFWRTVRGMIP	S	82			9870	LOC_Os02g12850	STAYGYGRGGYGYGGNAGFGSGYGG	Y	311
6919	LOC_Os07g03070	AASFNSSSQMAGSSNGFDSSTLSTA	S	390			9873	LOC_Os02g13330	WPIVRGFANPQRYKHFIKSCELAAG	Y	75
6922	LOC_Os07g03980	EYLSLESTKFKRSFTLPARMSSSTT	S	120			9876	LOC_Os02g17430	RLDFRATNNMAEYEGLLAGLRVAAG	Y	1063
6925	LOC_Os07g04220	NLTSLLLSGNSLSGAVPGSLLSVAG	S	168			9879	LOC_Os02g22210	NTSTHTTEGILDYMYSDLWRPARKT	Y	386
6928	LOC_Os07g05190	GLSFPGSSSKNLSLQVLDISGNHFN	S	508			9882	LOC_Os02g28020	KKKLRPDGTIEKYKARLVAKGYTQK	Y	697
6931	LOC_Os07g05350	VLSMATRACGGGSGAAGLGSLLGQS	S	115			9885	LOC_Os02g30680	MASLNLSKPSVKYSPAITQYMDEKS	Y	701
6934	LOC_Os07g06286	RKNFTVEELVVLSGAHSVGDGHCSS	S	124			9888	LOC_Os02g33540	DFAMTSSSLSSEYKHSDEQMRADAL	Y	172
6937	LOC_Os07g06380	ADILIFAARDASSILSNGRVRFDVP	S	79			9891	LOC_Os02g34680	LKAHMKTHSVDNYHVCKYPECARRF	Y	152
6940	LOC_Os07g07000	SALQNAKKLQKNSRKWMCYAIILLL	S	273			9894	LOC_Os02g35750	MLRRRCVPDVVTYTILLEATCKRSG	Y	196
6943	LOC_Os07g08190	NSSPPVNRGRSRSRSKSRDPSKSRS	S	586			9897	LOC_Os02g38250	ESEDEMDGRIAPYHLMEKRSLEKSI	Y	118
6946	LOC_Os07g08960	GRAGHWARECPYSSGGGGGRTGRYS	S	143			9900	LOC_Os02g40930	QWPKDLIEIRLQYDTDFGEDRTEAD	Y	347
6949	LOC_Os07g09340	LDIIKFLIRYALSGRAWDLVLEQRI	S	854			9903	LOC_Os02g42040	NGKDHEGADSESYSDSGSIDGHEDE	Y	864
6952	LOC_Os07g10400	YDSVVDVQNSLISMYARHGAIGESN	S	360			9906	LOC_Os02g43870	GVGAQGGVGAGAYGRADALVKFPGG	Y	108

6955	LOC_Os07g12220	RRNSMADDILGGSIYDPAYYSSLFA	S	181			9909	LOC_Os02g44810	ARTIEGDLEEALYRAGAVPEADRAA	Y	83
6958	LOC_Os07g12910	ALSDRKHGRDNKSGADNGSKSRHSG	S	148			9912	LOC_Os02g45070	IDNHGTVKTVVQYFQETYGFNIKHT	Y	496
6961	LOC_Os07g14270	YSIMFGPDICGYSTKKVHTIFTKND	S	150			9915	LOC_Os02g46956	ADSDLAVVLNAWYAAGFYTGRYLMQ	Y	325
6964	LOC_Os07g15620	AEAVLPSELTLRSPRATMYCEADQN	S	1743			9918	LOC_Os02g47900	DLHGVPMRGRFAYDENSKVILQEGY	Y	336
6967	LOC_Os07g16970	YDLNKMTTKELISKYGLDDNTIDFI	S	172			9921	LOC_Os02g49590	QRIKFKLKLVYMYAAGFYTYSIFAL	Y	127
6970	LOC_Os07g19320	EGWEPPRQLRRFSIDSVRVTLPRLP	S	743			9924	LOC_Os02g52280	KAGVDAKELFHLYIVLWIEDKRRTL	Y	709
6973	LOC_Os07g22024	KARNSLSVDNTSSRGGSKFSEDNEF	S	367			9927	LOC_Os02g52820	VDSTTLQLHNLLYEKNHYVKAIRAC	Y	116
6976	LOC_Os07g23270	PMRLMHEWYLRASELGLGMITVHVP	S	445			9930	LOC_Os02g54700	EDAEKAISKLNGYGYDNLILRVEWA	Y	272
6979	LOC_Os07g25460	GKERDYRRPAASSECGHRRKEGSKE	S	476			9933	LOC_Os02g55020	SMMEDGGLESDEYTFAILVNGLCKM	Y	339
6982	LOC_Os07g25680	NDSNQPNSSRDGSNLYSMSASTAVP	S	336			9936	LOC_Os02g55420	FNDAKVPWSEYRYYDPKTVGLDFEG	Y	205
6985	LOC_Os07g26430	LSQVRSLLFFGASVMDVSEYRFLRV	S	1062			9939	LOC_Os02g56210	IEVSMWQQRVRLYIITGVKGDTVFA	Y	186
6988	LOC_Os07g27790	PLTKEDLMAYLVSGCKPKENWRIGT	S	74			9942	LOC_Os02g57660	NWLDGMMHGYGIYTWNECGYYVGTW	Y	219
6991	LOC_Os07g29360	ESLKAIGHIYAKSGENDKAIETFKK	S	399			9945	LOC_Os03g01490	PHICISIDGVGTYCLNTASHTWIKV	Y	254
6994	LOC_Os07g30300	PPPGETVQERFISAAATTGVLLRKG	S	73			9948	LOC_Os03g03034	LITDGSPAVYRNYTYDEYYKKFWSR	Y	311
6997	LOC_Os07g31370	LVFLGDQSVGKTSIITRFMYDKFDN	S	24			9951	LOC_Os03g03870	TDRFGDYSGPSKYGKKTTPTISDDE	Y	160
7000	LOC_Os07g31450	TTYEFLVKWVGKSNIHNSWISESEL	S	500			9954	LOC_Os03g04520	GPLAAYRFLFNEYLGGACAFLEYID	Y	622
7003	LOC_Os07g31840	NGSIPDSLGQLASLQILNLNGNYLS	S	504			9957	LOC_Os03g04920	VLKDGHITQAGKYDDLLQAGTDFNA	Y	830
7006	LOC_Os07g32430	EREKRAKQQQQVSMPGLKKGSTYLR	S	1262			9960	LOC_Os03g06200	SSPAAAAADRGVYNFAAGPATLPLS	Y	66
7009	LOC_Os07g33340	TEQLQQISQNIASVLVQYPNLQQLG	S	469			9963	LOC_Os03g06950	SISLWVKNMNMGYTPEDKVRVFPIR	Y	200
7012	LOC_Os07g33760	PTGPSDHCPLVLSDPQCPCRPSAFK	S	235			9966	LOC_Os03g08220	PGPVSGEISAAYYRNHWRFDLEGLP	Y	645
7015	LOC_Os07g36420	SGEEEVGIDGWNSMDNCYAFLYIKE	S	66			9969	LOC_Os03g10340	SLADLQNDEDQAYRKIRLRAEDVQG	Y	81
7018	LOC_Os07g37270	HVAFKFQTTAPKSCFMRPPGAILAP	S	141			9972	LOC_Os03g11200	IEVYERAVLAVTYSVDIWYNYCQFA	Y	205
7021	LOC_Os07g37750	TAASSQTVSVKGSSLQCLVSILKSL	S	458			9975	LOC_Os03g13300	RMPENSIPKEAAYQIINDELMLDGN	Y	43
7024	LOC_Os07g38160	NQKTDNKYFSDASRTQLSQGCAMAV	S	272			9978	LOC_Os03g13890	PIQLAIDEVWAQYVQRGGLRKTGHD	Y	520
7027	LOC_Os07g38640	HRHVVSLRGYCNSNGERLLVYDYLP	S	390			9981	LOC_Os03g15650	LLAQKELQKINMYKAPRDKLACILN	Y	158
7030	LOC_Os07g38970	ARGFAAAAAAAPSPAVFVDKSTRVI	S	34			9984	LOC_Os03g15900	LISPMLKVLDEMYRAQDDLARDIRQ	Y	714
7033	LOC_Os07g39560	PNNPTNMSSFAPSGTGGQMGITSDG	S	335			9987	LOC_Os03g16700	LVSSDSTGEMPLYGNKIRRSTSFPR	Y	235
7036	LOC_Os07g40270	RTSTNDMNALCASEENTTASGLGEP	S	152			9990	LOC_Os03g17170	AFDQQNHGAVPQYHDRSGSEDKNGF	Y	122
7039	LOC_Os07g41280	IRQLVKVRTIGVSESNDCPKFDLIL	S	147			9993	LOC_Os03g19040	DPEMKRRRRVASYKAYSVEGKVKSS	Y	90
7042	LOC_Os07g41800	LGQSLYYLLTTGSGQQTLGEEYCDI	S	89			9996	LOC_Os03g19990	DVEGFVRVTHSRYENSTPAWTVFST	Y	674
7045	LOC_Os07g41820	NAALVPSPVNPGSAVRLSLAKLKEG	S	251			9999	LOC_Os03g21470	EALEAARIACNKYMAKHAGKDAFHL	Y	75
7048	LOC_Os07g42490	LSAHTNELVAVFSRLVNQGKGMLQP	S	36			10002	LOC_Os03g22190	DSTAKQMNGPKEYYSATLQKLNERR	Y	979
7051	LOC_Os07g43350	RAWDGTVETIDLSSNKLEGSYPNDA	S	371			10005	LOC_Os03g22900	MLIFWKRVDKEQYELRKREEKEAAE	Y	423
7054	LOC_Os07g43800	YLSAAADSDEPCSSCGGEDEDVVAA	S	82			10008	LOC_Os03g23990	SVPQYPLYPVYGYPPSTVVRQVGGL	Y	22
7057	LOC_Os07g43870	SKETLGPEFIGKSGHHKVKVIFFSS	S	274			10011	LOC_Os03g24790	NGSQFISADFQDYCIGLGVKICFAS	Y	357
7060	LOC_Os07g44970	ERLLDRRPELLSSVLLRQNPHNVEE	S	386			10014	LOC_Os03g27030	RSRSIERYERERYQEKGYRRRSRSI	Y	167
7063	LOC_Os07g45400	RVFEIALAVEASSAITATFAPPKMQ	S	1326			10017	LOC_Os03g28960	QVQVCRKCGLLGYYNHKLKASYCSM	Y	1106
7066	LOC_Os07g45970	LTTSTNTMARLISMLGWSSPNHTVV	S	462			10020	LOC_Os03g29085	WPTALKDACDMDYCIGLGVKICFAS	Y	802
7069	LOC_Os07g46180	VKGCAASGASSPSNNSLDAIAKVSR	S	534			10023	LOC_Os03g35560	LDVSYALSATSRYQSDLGESHWIAV	Y	1071
7072	LOC_Os07g46590	ALLHFLDPSKFNSKDIFVERYKNLS	S	56			10026	LOC_Os03g40110	PARKEKGWVDEIYEREGKKIGEDAE	Y	377
7075	LOC_Os07g47030	GRVFKSAKLMATSGILTDKPSKLDC	S	530			10029	LOC_Os03g43800	EAETGDYPKYASYLMNNEGSASGKS	Y	396
7078	LOC_Os07g47350	IQMEAEEAASSGSYESSEGRMAVIH	S	661			10032	LOC_Os03g44800	RHLARLRSTLREYRHTCLRLGIPLA	Y	163
7081	LOC_Os07g47920	QGGNLNYQYQPQSPHVQYQQAGSAQ	S	232			10035	LOC_Os03g45140	HDVEKEYGYEISYDKAWRAKQKALE	Y	370
7084	LOC_Os07g48310	YLQHNNFSGDLPSFLNPNLSVVDLS	S	139			10038	LOC_Os03g46590	RYGIMTTNLAEVYNWVMRGVRGLPL	Y	644
7087	LOC_Os07g49040	DGFTVTVASVGDSRCILDTQGGEVQ	S	153			10041	LOC_Os03g47800	YGGGRDRYANDRYPSGGDRYVPDRY	Y	195
7090	LOC_Os07g49460	LWQHVWRRCHSSSGSGSESGIQTQK	S	188			10044	LOC_Os03g48140	LSIQQLYRISTMYWDDKYGTHTVSS	Y	1493
7093	LOC_Os08g01130	AYTVAVEDVVDLSSPSPPLRKLSKS	S	495			10047	LOC_Os03g49400	SGVTKHDFSNKEYEVKLLQSLRFCI	Y	1076
7096	LOC_Os08g01620	KDVQGRVEVLSVSAHAEGGSSVDQY	S	240			10050	LOC_Os03g50390	VFAGDLVGVMEKYADSHPEWKETLE	Y	335
7099	LOC_Os08g01794	PMSPSSPSTVDASSKSPSSPSTEEN	S	37			10053	LOC_Os03g51600	VDVNEFQTNLVPYPRIHFMLSSYAP	Y	262
7102	LOC_Os08g02060	EAAEDDDEAARLSREGKVSVTSNPG	S	71			10056	LOC_Os03g55330	LPKLLKPGGIYSYFNGLCGDNAFFH	Y	263
7105	LOC_Os08g02354	GWQRMWWHYAQRSVLADVRRELRKT	S	336			10059	LOC_Os03g56790	VVVEKTGATTESYDDFLASLPENDC	Y	59
7108	LOC_Os08g02670	DPVAAGCRTVASSRKECPSPLAVQP	S	673			10062	LOC_Os03g58590	LDLDRESQINVKYWEALMVVCDWEL	Y	288
7111	LOC_Os08g03290	DLVSTDFVGDSRSSIFDAKAGIALN	S	294			10065	LOC_Os03g60460	NEDFWLNEGFTTYAERRIVEVVQGE	Y	333
7114	LOC_Os08g03390	QRNWKSDPNYTKSWYDRGAKLFQAN	S	77			10068	LOC_Os03g62600	AYYRGAVGALLVYDVTKATTFENVK	Y	98
7117	LOC_Os08g03650	IVKRCIDSVATRSCTDPDLFGWPVV	S	172			10071	LOC_Os03g63320	EASGAQAAAGPPYSKRRRRPSVRLG	Y	47
7120	LOC_Os08g05180	HPGKANVVADALSRKGYCNATEGRQ	S	945			10074	LOC_Os03g63650	RGRTTSVYRSGYYRPGMVQDDMAVP	Y	42
7123	LOC_Os08g06020	KDKFLVQSVIAPSGVTVKDITGEMF	S	102			10077	LOC_Os03g64340	VTGTVSGDKLAEYIHRRTGKLATVV	Y	205
7126	LOC_Os08g06140	FFAPKDRKYPNGSRSNRATEAGYWK	S	82			10080	LOC_Os04g02150	LPLLSYSAPFAIYHQYLQPLATCMH	Y	436
7129	LOC_Os08g07760	LVYPYMANGSVASRLRERPPSEPPL	S	385			10083	LOC_Os04g02370	CIQGGCPWRVHGYKPQHDTLWVASR	Y	500
7132	LOC_Os08g08220	NDNGKRILPSSFSNGRTTNAMHPVV	S	129			10086	LOC_Os04g02870	YAFRDDTERGDRYDGARGGYGRRDD	Y	174
7135	LOC_Os08g08570	RKAFVRAAGGPISSFAEKRGSFFSG	S	19			10089	LOC_Os04g07250	LLHLGGPDNITAYSLEDNKLWKRHL	Y	97
7138	LOC_Os08g09350	DGGRRGGRGGFQSRQSAGTASTGKK	S	554			10092	LOC_Os04g08480	DRHAQSYDVQLQYDTDFGDDRTEAD	Y	611
7141	LOC_Os08g10580	RSELAGANGVVDSNVGDLCSKTRSG	S	87			10095	LOC_Os04g17440	YAVLMASRKLRHYFQAHRVTVVTSY	Y	1355
7144	LOC_Os08g11570	IDCANRKVTLTSSNGETVSFFVSSP	S	427			10098	LOC_Os04g22060	QLLAYDNALTPRYFLTKFVDGLKDD	Y	258
7147	LOC_Os08g13970	TSPSYFSVRQGWSFGGHQTFRRLAT	S	1451			10101	LOC_Os04g22400	QRFERIRRTPARYALLTTGQHDILL	Y	417
7150	LOC_Os08g14460	LQQRQRDIEFRESTNDLRQRMQSDI	S	128			10104	LOC_Os04g26900	ELKKLVEEGKVKYIGLCEASASTIR	Y	17
7153	LOC_Os08g15720	TQLHSLRLEDLNSAHICLIPTKADA	S	93			10107	LOC_Os04g28770	QKIHACENYCILYRKEFADLNSCPT	Y	191
7156	LOC_Os08g16200	ERTVRARFDGFKSRLQAKDDEISRK	S	181			10110	LOC_Os04g31180	VTLFACGSEHKKYRLAKWSILCKPK	Y	460
7159	LOC_Os08g16930	SPVIDLNEACEPSSEEMEELHGYQE	S	161			10113	LOC_Os04g31500	ALVKFMAAVHYAYLQMKMPGPGWPH	Y	721
7162	LOC_Os08g17750	KYVRSDRGGEYLSLEFGNHLKECGI	S	673			10116	LOC_Os04g35240	KTADVWSCGVTLYVMLVGAYPFEDP	Y	191
7165	LOC_Os08g18060	AAAVVVSPPDPKSHHGGGGDEDVGR	S	25			10119	LOC_Os04g37640	GEGGRDPPPPWMYVHLRHDYTRPGA	Y	278
7168	LOC_Os08g20660	LVEIIKNIFVASSNERLSGSVGFVL	S	807			10122	LOC_Os04g38090	TKVAHFIPVKTTYSGSRLAELYMAR	Y	913
7171	LOC_Os08g23020	DENFEVHFKKTGSKVFDSYGDSVLN	S	641			10125	LOC_Os04g40740	MVYQDQIYLYGGYFKEVVSSDKSAS	Y	254
7174	LOC_Os08g23360	RIEEVEPMIGFCSRKLGGSALQAHE	S	241			10128	LOC_Os04g43140	KKRKKRKRGDDDYALPGDGDLVVEC	Y	120
7177	LOC_Os08g23930	AYCSVLRAFKAQSDAISWEKEGLIT	S	75			10131	LOC_Os04g44530	TSRSPTPFGTAKYMLDKIPKDASSK	Y	353

7180	LOC_Os08g24434	GDEEGGGDAADESTDEVAFDGDGPE	S	443			10134	LOC_Os04g45580	PQNCQRRGSALNYDAESETLSRAGS	Y	677
7183	LOC_Os08g25080	MDESQASDEARYSGKNGGGTDLNLN	S	161			10137	LOC_Os04g47380	CHLVISCQRSPIYIGGRYLKLSRNV	Y	142
7186	LOC_Os08g25570	GEGKHYALNVPLSDGIDDDTFRDLF	S	242			10140	LOC_Os04g49210	VSNWRDFLRLHCYPLESFIDQWPSN	Y	142
7189	LOC_Os08g27040	YDLHHGILGILYSVLITAVITDAIK	S	106			10143	LOC_Os04g51540	PFRRAPGGFEYLYVAIDKFTKWPEA	Y	1714
7192	LOC_Os08g27170	EPASTVPDSALYSWNSGMAADDHFS	S	494			10146	LOC_Os04g52760	CKGYLSAIIIYLYAHDFQQAQKCYN	Y	129
7195	LOC_Os08g27870	SIRLDLGMMEEDSFINNDVGEEVVV	S	51			10149	LOC_Os04g52920	VCTVNNAGVTSEYQTKATDNSSSIE	Y	265
7198	LOC_Os08g28880	MMGPKYDGRYLRSIVQELLGDTRIS	S	166			10152	LOC_Os04g55920	SPPPNPSQLTIFYGGSVCVYDSVPP	Y	78
7201	LOC_Os08g31470	SESSAAYFSWPTSTLMHGSAEGRAN	S	59			10155	LOC_Os04g56630	VRRKESESSSKRYNDDDKSSMYGNI	Y	765
7204	LOC_Os08g33082	QRDSSASLSKDSSSSSQYPSTSSNA	S	366			10158	LOC_Os04g59624	NPSTDNQSVDRAYRIGQMKDVIVYR	Y	355
7207	LOC_Os08g33380	EESSSDEDDEILSELKEKWDAIENK	S	72			10161	LOC_Os05g01480	MAGTRRAEEEYDYLFKVVLIGDSGV	Y	13
7210	LOC_Os08g34690	LDRCLEEEVVSISADFDILQYWKMF	S	476			10164	LOC_Os05g03630	ADDSLFKQVSEAYDVLSDPQKRAIY	Y	53
7213	LOC_Os08g34800	SSAASSELLRTSSAGSSSSSPAGGG	S	113			10167	LOC_Os05g06980	QAVADILQDRTDYKSFKKTVARLEE	Y	46
7216	LOC_Os08g35110	RAPPSAGELEEGSAAAAGRAASFHG	S	37			10170	LOC_Os05g08300	FGGSTSYSLKREYKKVEREVCSTSQ	Y	197
7219	LOC_Os08g36480	DLPGGGGSKYGTSITRQWALDPSRD	S	264			10173	LOC_Os05g10810	VETVLKLSLPNVYLWLCMFYAFFHL	Y	381
7222	LOC_Os08g36994	DVKIQGIIGPCTSLEKKSPLSSDTV	S	424			10176	LOC_Os05g13510	KILSMQTDWGGEYLKLHTFFNEIGI	Y	543
7225	LOC_Os08g37800	IKGGIYTLPSHLSALARDLIPRMLV	S	239			10179	LOC_Os05g14940	RVVDVETMEWEVYAVYQKEKENLMQ	Y	215
7228	LOC_Os08g38050	LAYTRRYYVVDMSNKQIFQVYHGPG	S	212			10182	LOC_Os05g19850	LAPGTTPLYKRPYRMAANELAEVKK	Y	874
7231	LOC_Os08g38320	TCGSGKVSDTADSTESGKSSMCRPS	S	140			10185	LOC_Os05g23840	TLGEELRARHRQYVWELGRSPLEAE	Y	97
7234	LOC_Os08g39050	ARLFDEVLQLFESSETYGVELDDIS	S	206			10188	LOC_Os05g23940	GRRTTHVAAACLYIACRQSKKAYLL	Y	124
7237	LOC_Os08g39490	PCIAAPVVYRPPSLLDVAEKVADTG	S	602			10191	LOC_Os05g27820	MHGWDREGHPVCYNAYGVFKDRDMY	Y	174
7240	LOC_Os08g39810	KNAVATDQDDSLSKDPPAEAENGTS	S	539			10194	LOC_Os05g32610	DKPMLERLRSNPYEGVKTRFVMEVV	Y	1220
7243	LOC_Os08g40510	FPPPRLSLHAQKSSLTPRSSNPASS	S	230			10197	LOC_Os05g33940	GGFCISHPSWLMYHHFYARLACALP	Y	118
7246	LOC_Os08g41010	PAGPGPMGARAGSYSEEGSQKKPAG	S	180			10200	LOC_Os05g37434	RRSHFDPEEFALYQRYQTKVHKEKK	Y	358
7249	LOC_Os08g41400	VMFTNSVDHVSSSTKEPVNLLEDKM	S	369			10203	LOC_Os05g40300	IGGMLCIMGNAQYYIHRAAHGRPKH	Y	28
7252	LOC_Os08g42370	FTVYDLTLLLLTSGRDPGIIPRNTH	S	100			10206	LOC_Os05g43060	FLYKRVALSSLIYWGIQPTETELLK	Y	367
7255	LOC_Os08g42600	PPENSAEPNAQASDYYRFGWLLFLV	S	194			10209	LOC_Os05g44100	IDQSVPSAVRLAYYTVAECVVVTAV	Y	449
7258	LOC_Os08g42710	PSLEMIKKILISSYEGLSYHLKSCF	S	941			10212	LOC_Os05g45420	IDTPADIYVVMEYVKSGELFDYIVE	Y	93
7261	LOC_Os08g43090	LPPRKGGHRRSQSDIPFGFSHLSPP	S	141			10215	LOC_Os05g46490	SGAEYVRKEVPLYVFPTRHLLKLDT	Y	276
7264	LOC_Os08g43130	WYFQHVGIASSKSSRGRTVDIDAAD	S	470			10218	LOC_Os05g49230	QDKFYHLAKEQGYRSRAAFKLLQLD	Y	22
7267	LOC_Os08g44150	NELMSIETQITYSYYSLPFCRPDNL	S	49			10221	LOC_Os05g50810	GRELGRGQFGVTYLATHKPTGRRYA	Y	128
7270	LOC_Os08g44540	KLAQTWVDKMSASDQDEPKAKDFEG	S	45			10224	LOC_Os05g51280	TNLQNDRQGMVNYIYDTLWSRRDKE	Y	724
7273	LOC_Os09g01540	LAGRQMPKIRTSSRYKLLGTILFRH	S	391			10227	LOC_Os05g51754	ATGSRGKTEKKVYSLPGQKFDPPEE	Y	49
7276	LOC_Os09g03600	CSLLALEKGILTSSGATSCNDEIEV	S	486			10230	LOC_Os06g02180	IYGSVTEDVVTGYRMHNRGWKSVYC	Y	878
7279	LOC_Os09g04890	IEYIGELVRPPISDIRERRIYNSLV	S	900			10233	LOC_Os06g04330	DRQILVSRNSRGYFALGYSLPGEPF	Y	41
7282	LOC_Os09g07294	GSGRGDCVYTTVSHFNIIHYQCHQE	S	369			10236	LOC_Os06g04510	VVIGMDVAASEFYSEKDKTYDLNFK	Y	258
7285	LOC_Os09g07500	TQIPKKETQPVSSSGASEMAVGAAL	S	95			10239	LOC_Os06g05520	QELKISLSTQCQYVVACCQCFYVNG	Y	122
7288	LOC_Os09g08420	EKSSLRGSVKDSSASAKGSDRASSL	S	92			10242	LOC_Os06g07350	RMVLQRACAFVTYTTREGAEKAAEE	Y	270
7291	LOC_Os09g09360	NGKGHFVFVSNLSPCSGVRIHLWPE	S	465			10245	LOC_Os06g10170	GGKPCLGGVSTWYNDEMCRDMGYEP	Y	489
7294	LOC_Os09g11020	LERAMISKPMLKSKCFLRHLILSCT	S	840			10248	LOC_Os06g12260	DIASLGARVEELYRPGELAARGSRV	Y	562
7297	LOC_Os09g12130	GVQASGPEGSAPSSLRLIAWIAEIQ	S	1525			10251	LOC_Os06g12780	PNFSLPWQRKRQYSSSSSGFIIGGH	Y	190
7300	LOC_Os09g12270	GGKQLEVGDGKHSNKIRTLEDGLIV	S	305			10254	LOC_Os06g13680	AGVLENHEEGRRYAEHGLRNYVRDK	Y	43
7303	LOC_Os09g14490	KCCGSSVGGCNTSANNHLRYLAVLV	S	992			10257	LOC_Os06g16280	VLESSVSLFVGSYILQLIIHLPSHL	Y	794
7306	LOC_Os09g14720	SDSAYPDSDLHVSHIFGGSTAYSSK	S	667			10260	LOC_Os06g20390	PQSQFSSPAQSSYSSPSYQGTDYPS	Y	257
7309	LOC_Os09g15130	AKWALELMPLDISFKPRTSIKSQAL	S	281			10263	LOC_Os06g20840	RGANGIAIKQAAYARKILTQFGMLH	Y	293
7312	LOC_Os09g15750	GKKKDENKDDDLSEEDQALKEQLEL	S	38			10266	LOC_Os06g23530	LREILLDTDLSSYSVVMLDEAHERT	Y	545
7315	LOC_Os09g17200	VIVRGTNREANDSDDEEPDSPMRVD	S	219			10269	LOC_Os06g27970	AGTNPDPVSVVSYLNNLKSSEESLK	Y	125
7318	LOC_Os09g19590	LHLLEDYGSSSTSTPREVLAIDETG	S	220			10272	LOC_Os06g29430	EGAVDVRRGASRYTDNNQRSDWRTE	Y	349
7321	LOC_Os09g19734	KNKNITVDVKANSDSNVVTTVTVDE	S	72			10275	LOC_Os06g32060	PQTKPYPLDMWKYGSALQQRPELNP	Y	1411
7324	LOC_Os09g19830	RGVKRRAVSAKRSWPPLCGRFPAPP	S	36			10278	LOC_Os06g35030	AAGKDGGEEVREYKSDARKLEELFK	Y	57
7327	LOC_Os09g19952	MNKFRPRARGRSSPIKKNHVSYNNC	S	82			10281	LOC_Os06g37440	VTLADIVMTCNLYYGFVRILIKSFT	Y	168
7330	LOC_Os09g20860	RPHLPLLHLPAASLPDPLRLAPSLR	S	95			10284	LOC_Os06g41660	PEDGLPMTLNFLYLSGCHPLLEEQF	Y	1103
7333	LOC_Os09g22150	YLHQMWAYERYMSVLKGLHEGRQTH	S	456			10287	LOC_Os06g42190	WPPRFRPTIAEKYDGSVNPAEFLQV	Y	245
7336	LOC_Os09g23730	FSKNNYFRGDDPSLPPKRGRGRPPK	S	94			10290	LOC_Os06g45870	SGKRVRTRKQFAYPIFRPTENKLQK	Y	234
7339	LOC_Os09g25370	ALQGVRSIVLKPSESLDEGRFTRIA	S	32			10293	LOC_Os06g46880	ARGMPPPQAQQLYSHLPPHQQPPHF	Y	68
7342	LOC_Os09g26560	SEDCNQMISLKFSVKTRGGISEATT	S	438			10296	LOC_Os06g49740	TVHPDCINASNPYHVCSEYCFKRIA	Y	64
7345	LOC_Os09g26830	LIVSMGYGVVRPSLGGLTSKVVMLG	S	327			10299	LOC_Os06g51220	RWKSLTEADKAPYVAKANKLKAEYN	Y	92
7348	LOC_Os09g27020	LHSLRHGFQQIWSKGLMPSANIKYN	S	136			10302	LOC_Os07g01110	CPILPGSGAGSSYTYRFNVTGQEGT	Y	121
7351	LOC_Os09g27850	YPYLWETVEELSSQHPTGAVFKKAF	S	380			10305	LOC_Os07g02610	QLNILRKEMYRGYYTLDRFRCHVHE	Y	101
7354	LOC_Os09g28310	DELLRSIWTAEESQAMASASGSAAG	S	87			10308	LOC_Os07g06130	NNQFYQPATIHGYQLASYLKQMNAN	Y	830
7357	LOC_Os09g29080	IWISSIVVSALDSVPLVPQVMEVVG	S	131			10311	LOC_Os07g08960	YERDGGRSGGSYYRDEPRGSGGYDR	Y	251
7360	LOC_Os09g29810	RQAPRAWNAKLDSTLKGMGFEQSPY	S	1248			10314	LOC_Os07g10050	DSQLVVNQVCKEYRCSDPQMDAYVR	Y	1471
7363	LOC_Os09g29890	LPSFSKFGLMNASHRNPLSKLDECD	S	501			10317	LOC_Os07g10890	PFPMYHPGAAAAYYAHASMAAGVPY	Y	87
7366	LOC_Os09g30418	FRETRLHSCCRSSGGLAMEATKETQ	S	714			10320	LOC_Os07g14270	YIPDPEDKKPEGYDDIPKEIPDPDA	Y	246
7369	LOC_Os09g31486	EQEITIKSSGGLSESDIEKMVREAE	S	555			10323	LOC_Os07g26690	CGVGLVKAFQSAYFNRYGGGANTLA	Y	155
7372	LOC_Os09g32020	VNQETAASGVSNSTRQKKGKLVFGS	S	277			10326	LOC_Os07g28430	MQLQELEQQSRVYQYMAARVPVPTH	Y	130
7375	LOC_Os09g32330	SNFYAGKSKSFTSLAEATASPAAAA	S	126			10329	LOC_Os07g29520	KARLVAKGFKQRYGIDYEDTFNPVV	Y	536
7378	LOC_Os09g32740	AECGVGAEEIVTSSLPSETQRILPG	S	869			10332	LOC_Os07g30820	SYGAPYPHLGLRYGYGPPVGPPASY	Y	124
7381	LOC_Os09g33450	LQGRFIFGPDVRSLFLTIFLILAPV	S	46			10335	LOC_Os07g31460	ANCFTFYCRSFGYEARLILDFTDHV	Y	340
7384	LOC_Os09g33510	SKHTTFPSLVAHSAPLVPNSAPLAP	S	525			10338	LOC_Os07g32530	LGTVRVPGIGLLYNELARGVPGIFG	Y	622
7387	LOC_Os09g33980	MEESRSKSKDQKSSVDLPKAPKEVD	S	850			10341	LOC_Os07g37650	FIQSMGNEKSNSYWEAELPPNYDRV	Y	95
7390	LOC_Os09g34070	PPTLPPSSAILSSQVGHSLPTQMSQ	S	790			10344	LOC_Os07g38640	AEVLGKGVSGSTYKAVLEDGIVVAV	Y	339
7393	LOC_Os09g35760	CALAGKFLGRPISSISSPGPPSLQA	S	259			10347	LOC_Os07g42950	SKDDDVRKYVNTYRRTFTTKNGKKV	Y	162
7396	LOC_Os09g35890	THTDDQKHSESMSNSAENAIKLYAC	S	534			10350	LOC_Os07g43800	FLAAASSGFFYSYSSFLHSFARSLV	Y	45
7399	LOC_Os09g36090	DAANLGPATIQSSTQGYYGMQNSQA	S	1340			10353	LOC_Os07g44710	TEADMWSIGVIAYILLCGSRPFWAR	Y	336
7402	LOC_Os09g36270	ASTIASSFGKTISDDKELSDYRPED	S	340			10356	LOC_Os07g47180	KQPMTSRVVTLWYRPPELLLGATDY	Y	292

7405	LOC_Os09g36480	PVKLGKDPTVETSFLPDREREAEEQ	S	157			10359	LOC_Os07g48310	KHANLVPLRAYYYSKDEKLIVYDYL	Y	408
7408	LOC_Os09g36540	QQQSRILFKPSSSLERPSVSANLVF	S	24			10362	LOC_Os08g01930	AQQGAQQGGYAQYPQSQPAYGDQAA	Y	585
7411	LOC_Os09g37230	EHKPYDHKADVFSFGILMWELLTGK	S	502			10365	LOC_Os08g03840	WTTGDDQNKIILYDSEVEGRNRVLG	Y	354
7414	LOC_Os09g37310	IPEGVSVLALEASSPAKSTTPLLAA	S	301			10368	LOC_Os08g07480	ADGGGGRKVRGPYCRFCRSAEEVVR	Y	1217
7417	LOC_Os09g37720	LPSAAAPGASGPSSHGGKPGGGGGS	S	50			10371	LOC_Os08g08830	LKVRNESVINALYQDLPRQCKTCGL	Y	823
7420	LOC_Os09g38200	LHYEICWEREPASTEVIGDSWLEVG	S	688			10374	LOC_Os08g12140	PPHQSNSGRDIEYAPGLIPNGGGPS	Y	441
7423	LOC_Os09g38390	GGGGKPALDRSGSKVLDGDETIFTD	S	31			10377	LOC_Os08g16130	EACLIGGSVRNAYHTKYLGYYMKHD	Y	123
7426	LOC_Os09g38580	LIGNMQTYLQSISKRLEEWRLRQRD	S	406			10380	LOC_Os08g20390	EGRRDGERRLLVYGDGSTPQGMLQA	Y	72
7429	LOC_Os09g39210	DLVLQNANIIDGSKIRTGESNAIDC	S	361			10383	LOC_Os08g25570	GYTIRNVARCWCYETAVAVGVEPDN	Y	327
7432	LOC_Os09g39540	VLFDQATYDKLLSEVPKYKQITPSV	S	53			10386	LOC_Os08g28570	ALENLPCLMTVQYLLYQYNKNDHEI	Y	827
7435	LOC_Os09g39900	DIHEGECGAHSASRTLVGKAFRQGF	S	1673			10389	LOC_Os08g29500	VLRALQDLGFREYIEEVQAAYEHHK	Y	87
7438	LOC_Os10g02509	EAPPSMPMNFARSDMWMHPQQQQQH	S	97			10392	LOC_Os08g37280	KKTAKHHVPVRLYGTCVHMYVDPWK	Y	72
7441	LOC_Os10g03830	DRAIKKMKGQSESSLLSAQRSQRGE	S	218			10395	LOC_Os08g40800	RPALVKFMAAVHYAYLQMKMPGPGG	Y	719
7444	LOC_Os10g06130	KRDFGFVHFAERSSALKAVKGSEKY	S	338			10398	LOC_Os08g42740	GSDEVVSRREQRYIVLTEKDINERQ	Y	79
7447	LOC_Os10g07150	RLPPLSPHTAALSLSSLHGRRAALF	S	40			10401	LOC_Os08g43110	AAKLWCDNLGAKYLSANPIFHARTK	Y	1368
7450	LOC_Os10g07480	AESWANALISELSHIKKDNGGKGSL	S	318			10404	LOC_Os09g02480	LLVITPKWSTCHYLNSRIDKNAYDW	Y	86
7453	LOC_Os10g08410	MSDVAAAAATSGSRVPKMRRTFTSV	S	211			10407	LOC_Os09g03610	LQSSYQSSQQAIYQLQQQLQLMQQQ	Y	553
7456	LOC_Os10g08550	VIAPALIGKDPTSQAELDNFMVQQL	S	120			10410	LOC_Os09g08390	LQERTKRKIKVLYGSGRDELLKVMD	Y	222
7459	LOC_Os10g10620	CATTRYPDVCYDSLLPYASTFQTSH	S	60			10413	LOC_Os09g13700	LRAARYQQSLRRYHQRHVRARSLCV	Y	1797
7462	LOC_Os10g10990	MKAATNGPAAFGSDVKKLEPTNGTE	S	210			10416	LOC_Os09g15790	GQERYRAITSAYYRGAVGALVVYDV	Y	82
7465	LOC_Os10g14230	EKKKMYEYFKTLSCRFCTTMDMWTS	S	218			10419	LOC_Os09g19560	YKEDKIEFWNNVYGFDMRCIKKQAM	Y	236
7468	LOC_Os10g16520	GTDHVPKDCQRNSVLALTKKEQGTT	S	313			10422	LOC_Os09g23570	AEVLGKGWLGTTYRATLEGGAAVVA	Y	375
7471	LOC_Os10g17340	KANVVADALSRKSHCNTLNVRSIPP	S	1278			10425	LOC_Os09g32020	SSVALCEKLQLWYALAKISAEKAAW	Y	417
7474	LOC_Os10g18940	LTTSTLEDERKHSTYEHESFSFKIP	S	369			10428	LOC_Os09g36030	QMGPLDLSASMGYLWDPGNRKVRAR	Y	240
7477	LOC_Os10g20240	SFDDLPKVPLVKSPSSSVLMNAFQS	S	250			10431	LOC_Os09g37040	SCFMIYVSAQKAYIEALDGWLSKFI	Y	505
7480	LOC_Os10g21000	IEQYCFRAPYITSLLREGLQIKDNQ	S	493			10434	LOC_Os09g39462	AKVAIKQRPQKHYHNNSSSGAHSSR	Y	181
7483	LOC_Os10g21840	SWLIHLAPYSISSWADLWQQFVANF	S	295			10437	LOC_Os10g08570	SEQKALTKVKTVYLEHFPRSWDERN	Y	345
7486	LOC_Os10g22950	SSATADISATENSVQQNDGSLGAAI	S	348			10440	LOC_Os10g21000	LRIGSISHQLSAYSLSGYGLNDAFD	Y	306
7489	LOC_Os10g25090	ANDVTVLNALFTSLNSPGQLRGWQV	S	43			10443	LOC_Os10g25090	SQNNLGGGQNIQYNLPNKKLERLNL	Y	117
7492	LOC_Os10g26540	EWDMKNGAVPDYSMDFSKIREMRKQ	S	38			10446	LOC_Os10g25320	EGLSVTAESDDEYDSGNDDHPTLSA	Y	109
7495	LOC_Os10g28200	KAPAAFCRKAQTSTDRFEMWGDGLQ	S	230			10449	LOC_Os10g31520	AFVPPKEDTATPYESAKSTSNNDEI	Y	336
7498	LOC_Os10g29400	HRLADLRVESMESCSRKVEEDRARS	S	330			10452	LOC_Os10g33230	GAGYGSGNGGSGYPNAWADPSQGGG	Y	424
7501	LOC_Os10g30100	ARLSGRSRALPSSRDFHFYNNFPSF	S	44			10455	LOC_Os10g37740	PTREEIRCMNPNYSEFKFPQIKAHP	Y	372
7504	LOC_Os10g31770	TRYEWDCSFQYGSLVEEVDGHTAIL	S	273			10458	LOC_Os10g41400	TPNRQGNDVGTQYRSGIYYYTPEQE	Y	188
7507	LOC_Os10g32920	GRGGSAGNKFRMSLGLPVAATVNCA	S	17			10461	LOC_Os11g04070	TIAAAPHMFLNGYKNVLAVAVETEY	Y	248
7510	LOC_Os10g33680	WINDNKITMDTASQIFEILRKPDYN	S	221			10464	LOC_Os11g07280	IMVSILQLGQSSYLPHPIDNDSYDR	Y	608
7513	LOC_Os10g34370	LVQGYCKEGNVGSAMRVFKRMKVMK	S	312			10467	LOC_Os11g10070	CANGMSLKYVKNYKRKAFWIYGCMV	Y	527
7516	LOC_Os10g34400	RISAEHGRYVAKSYDTRQLNSDGPG	S	281			10470	LOC_Os11g11390	EALEAARIACNKYMTKNAGKDAFHL	Y	75
7519	LOC_Os10g34660	GHRISRSERESRSDTYYDRTKRDGT	S	96			10473	LOC_Os11g17890	STWSQMEHEFHDYFKDASLMEQRPI	Y	524
7522	LOC_Os10g35150	RDRMVSLFGAADSWLKQLGVQHHDY	S	298			10476	LOC_Os11g23020	QEETKGKEVKKKYVAPQEFQLGMPL	Y	592
7525	LOC_Os10g35580	SDLELEEFGMASSSRQRLSDISDVQ	S	310			10479	LOC_Os11g27820	SKTRYPQVQKLLYGHSVTVVTSFLL	Y	49
7528	LOC_Os10g35870	APATEEKPREVSSEEVKEKEDAVAA	S	210			10482	LOC_Os11g31000	FRAAGEMLLSLPYLLPGQPGRSGSS	Y	27
7531	LOC_Os10g36710	YIRAQYNLLEPSSTDGRLCIENFRM	S	497			10485	LOC_Os11g32970	KLEKVFQCKDGGYRWGIMTSNGSES	Y	225
7534	LOC_Os10g36880	AGSRRPPTSSSSSAGSYLTGRLMPR	S	32			10488	LOC_Os11g35850	PTNMENMSLEKLYLDCNHIADRIPR	Y	534
7537	LOC_Os10g37640	RSDPFGASTVALSVSKVMGEDAKPE	S	265			10491	LOC_Os11g37700	ELIKELSTPPPGYQDLSFPTKYSQN	Y	1164
7540	LOC_Os10g39520	ISLLLSVTGRYISRICIPVGAADTM	S	18			10494	LOC_Os11g39660	WDSMIDKVKKILYRHEGKELDEQCS	Y	500
7543	LOC_Os10g39820	CGLPGHIAAECSSKDLCWNCKEPGH	S	75			10497	LOC_Os11g47330	IVEPFVPHDQEYYLSIVSERLGSTI	Y	118
7546	LOC_Os10g40540	TLLTDDPSVIDTSLTHYGDLVYPDK	S	253			10500	LOC_Os12g01200	TTVRSFPADNRKYCYTMNVRNRTRY	Y	81
7549	LOC_Os10g40780	EDVRSLVQGVDDSTFDSTHRELCQL	S	22			10503	LOC_Os12g03880	RPFSYGLVITNVYDSGSVFSPEVLD	Y	196
7552	LOC_Os10g41030	PWLVQPQNLIFDSSMKPPVPASANE	S	1120			10506	LOC_Os12g04890	EYKENEVIQGAIYSRAEDMKEAVKH	Y	110
7555	LOC_Os10g41260	SLSMISGLEMASSSISSLELSIAPP	S	242			10509	LOC_Os12g06810	NRFPKENTKCGFYLHPAEGSPAPNI	Y	633
7558	LOC_Os10g41770	FWLYLSSSARPSSVILHQVAEGGGD	S	51			10512	LOC_Os12g12480	GMFLGYPAHSRGYRVLVLKTNKIVE	Y	210
7561	LOC_Os10g41960	DFSPGEKYLVTYSSHEPSNPRDTHR	S	273			10515	LOC_Os12g13770	LYGVEVNLGSLRYFKQDDLTVEDYK	Y	629
7564	LOC_Os10g42090	MKLEAVDSRNAASTYNILNEEGRPV	S	151			10518	LOC_Os12g16430	VAKGFKQRYGIDYEDTFSPVVKAAT	Y	863
7567	LOC_Os10g42320	IEHESPEITIDGSRYQLAYAQVARV	S	345			10521	LOC_Os12g21700	AVAAARMNGEYPYRVGQPECQYYLK	Y	110
7570	LOC_Os10g42439	LWYVLPLLLQYDSTAEENATSEAHG	S	1831			10524	LOC_Os12g21940	SLSRRWSGTLGQYNMLDACTARPPL	Y	418
7573	LOC_Os11g01190	SRLSPPNSSSGRSTLPPAASWGHRD	S	318			10527	LOC_Os12g24580	SSMEKQTYAQNKYGGITPKKPLISK	Y	23
7576	LOC_Os11g01836	MKPDCYAVAGALSACTRLGALDLGR	S	281			10530	LOC_Os12g29660	VINFSCPRDARTYLHRVGRTARAGR	Y	523
7579	LOC_Os11g01880	PSAAEEIESDIISLAQSEDSDVYDI	S	216			10533	LOC_Os12g32710	IAPRVMEMLRLSYEDLTSEVQLCFQ	Y	441
7582	LOC_Os11g02620	LVNFLQRIEEDDSGNLSPCGYESDD	S	362			10536	LOC_Os12g35100	RFPDMNGYVDECYSVQRLRQTYAGI	Y	783
7585	LOC_Os11g03810	KCFNLECSFVQTSKKHAIGAKYDKF	S	137			10539	LOC_Os12g36730	PAGTSLQEIGESYFSELINRSLIQP	Y	381
7588	LOC_Os11g04890	ASADTPMSDATPSAADTPNLPDTPA	S	34			10542	LOC_Os12g38000	RTEKPMLKAGNAYHKYRVKRNCWPK	Y	186
7591	LOC_Os11g04930	PPFQPPPPDTPISDATPSAAETPNL	S	36			10545	LOC_Os12g39370	DEHGLVKPRYGRYYDEGLEKTRQTL	Y	120
7594	LOC_Os11g06040	ERDERLKQHLIVSQELHPALLSSEH	S	253			10548	LOC_Os12g42840	SSREMVDDDDALYPYVCGLSGSTEI	Y	152
7597	LOC_Os11g06410	GPSYGSLDLHGISKQMHPPNSGNQG	S	73			10551	LOC_Osm1g00400	AENRKRAISPFVYKSSLYRNSTYCS	Y	277
7600	LOC_Os11g06650	DWVRLHNSSGDASNSIITDGNSPEK	S	186							
7603	LOC_Os11g06680	SPKHFNQPPDGISFSWYGHYRGPLD	S	221							
7606	LOC_Os11g07500	AKAYPFQLDPFQSVSIACLERNESV	S	89							
7609	LOC_Os11g08440	SLHDGIDFCGKMSRSRFEELNKELF	S	328							
7612	LOC_Os11g08800	SASRVRQCWPEESHQGIQAGGVEES	S	102							
7615	LOC_Os11g09240	MRPPANLGMCNASPEAENALTQLKA	S	1063							
7618	LOC_Os11g09420	RRARRAAAAVRHSAATPSSTPPTRE	S	156							
7621	LOC_Os11g10070	PIASLHSFPRRTSSGGVNPQQSQQQ	S	611							
7624	LOC_Os11g10520	FSEINTAFDLMLSGESLRCIIRMED	S	368							
7627	LOC_Os11g11340	GRSRGGEARLWLSEPAMVGAALRPG	S	90							

7630	LOC_Os11g13600	RPVGVQASGPEGSAPNSLRLIAWIA	S	1573							
7633	LOC_Os11g14220	VVEPYNSVLSTHSLLEHTDVAVLLD	S	193							
7636	LOC_Os11g16470	LKLRELLSSCTISQEIEKLKNQVLE	S	120							
7639	LOC_Os11g16730	EALSRNFALQTISHHWRQWKLDMNT	S	140							
7642	LOC_Os11g18470	PGLEHVKAGAASSSSPLRQKEQEGA	S	86							
7645	LOC_Os11g19130	KSVSDQSLRANHSASKQLLSQNGRP	S	307							
7648	LOC_Os11g19810	NSLSCSEMLSQLSLAVFNLKKKERF	S	796							
7651	LOC_Os11g20570	HAKKSTPESKARSEAYRLLQTKNQH	S	154							
7654	LOC_Os11g25410	FRARAAALLRRVSYRSAADVVQTSG	S	489							
7657	LOC_Os11g28230	RWFYHTIPFEAGSESAKALRCRRRA	S	329							
7660	LOC_Os11g30350	SVGVGGGGGGRSSGGSTAVMDLSSR	S	152							
7663	LOC_Os11g31000	SLPYLLPGQPGRSGSSTVTFYTTME	S	36							
7666	LOC_Os11g31890	MPPHGNTKQTSSSVNKADPRLAAGQ	S	884							
7669	LOC_Os11g32900	DKPSSKLEEFLSSCDTDLTSDVVKR	S	418							
7672	LOC_Os11g33340	PVVHSYSIVTLHSAFFYVDNRKVSV	S	264							
7675	LOC_Os11g34190	EGDKDHSSGSDISEATIKNAIVKRA	S	142							
7678	LOC_Os11g34600	APTHEATRRARWSEVALTFDQTDHL	S	590							
7681	LOC_Os11g35050	PPGFESVVNGKRSRDWNTDGFSEVN	S	488							
7684	LOC_Os11g35450	CSGMSMMSFFRLSNNSFSGNFPSFL	S	640							
7687	LOC_Os11g35890	WNQLSGRIPGNISVMQSLESLDLSK	S	828							
7690	LOC_Os11g36880	WAAAPSGSNSVSSLHLKLKSTAASL	S	170							
7693	LOC_Os11g37890	ARINGVKRFFYASSACIYPEFKQLD	S	137							
7696	LOC_Os11g38620	EKLDIDPNSVRSSAIPQSSTPNGNG	S	698							
7699	LOC_Os11g39030	AQYATAGAAAAASAAGRRVYVTNVA	S	175							
7702	LOC_Os11g40090	LGNADTSVGANESQEPTVRNIPPYD	S	271							
7705	LOC_Os11g40130	PAAAAEEERRRSSKRKGKRCASNGS	S	48							
7708	LOC_Os11g40820	TKTFNILKKHGNSWIEELPAVLWAN	S	1807							
7711	LOC_Os11g41890	YSSNSASSNSFMSSANGGLGGIGNN	S	335							
7714	LOC_Os11g42420	WHEYYSLDATYRSWLKFEMENASIS	S	812							
7717	LOC_Os11g42930	SLAEKDASEVSNSELGATTTDMPKF	S	130							
7720	LOC_Os11g43590	VTGLELAQEESNSLSNTVHADNVRL	S	617							
7723	LOC_Os11g44880	DATQDVVFYQRNSPEPQWSWAGSVA	S	888							
7726	LOC_Os11g44940	STIERYQLGFLISALGFDLSNLLVF	S	122							
7729	LOC_Os11g46960	HVIDGQLVQDSSSSTSSIDGFLMPV	S	117							
7732	LOC_Os11g47240	TDSIPNSISHLTSLEVLDLSYNNLS	S	621							
7735	LOC_Os11g47830	APDSERPWALCRSPRPSPPGQSHSR	S	190							
7738	LOC_Os12g01200	VCYLFTCKRKKKSSDETVVIAAPAK	S	557							
7741	LOC_Os12g01922	LASHIGPVLRVGSDNSATARDGRIS	S	782							
7744	LOC_Os12g02540	PRFCNPISRRIFSDVAGDLTVSVDG	S	34							
7747	LOC_Os12g02690	QALETTGKDLIGSNSPRSCETPSSE	S	181							
7750	LOC_Os12g04040	FMPESATGREIVSLGEGCPAPRYPG	S	42							
7753	LOC_Os12g05250	MAATSFPNPFFPSGGEGMDLGKPAT	S	13							
7756	LOC_Os12g06520	SMDGVNSPFEGESALSGGLPDYAKK	S	136							
7759	LOC_Os12g06570	LKYVANQFKRLHSKRLQHAFRYYSH	S	488							
7762	LOC_Os12g06810	WDLDSSLAPVAKSVDAKEDEAPNGN	S	165							
7765	LOC_Os12g06870	FGQPSAPTFGSTSTGAFGQPSAPAF	S	83							
7768	LOC_Os12g07230	DLESDPWPSISDSAKDLVRNMLIRD	S	279							
7771	LOC_Os12g07530	LRVCSTEMGRTSSLYLDRRSPPLPP	S	239							
7774	LOC_Os12g08800	TDKDVDQSSDIHSEISNNDMRTIIT	S	272							
7777	LOC_Os12g09590	SESNMDDAPTSGSDPEMDNGATHPV	S	141							
7780	LOC_Os12g09880	SSGRLSAAGSTASGGSGGGWERRGS	S	28							
7783	LOC_Os12g10630	ALPPPTRMVMPLSAMHTSESDDAAA	S	172							
7786	LOC_Os12g11240	RSIDWDVVREEESSKMGDGRSRWTD	S	107							
7789	LOC_Os12g12370	RRSAVRVTSEFDSDARLFSHRLSCR	S	59							
7792	LOC_Os12g13170	RKEYDELLSKNSSLKEKLEDKQHKT	S	358							
7795	LOC_Os12g13670	ASSSHSIERDRLSSYSKASATSSGE	S	327							
7798	LOC_Os12g14920	DVATSTSASNCTSGEDVSSQAQNHL	S	197							
7801	LOC_Os12g15730	PVEESNLPFKKHSIFFRYLNYWKDL	S	97							
7804	LOC_Os12g16350	DGVAGGVLQSNESSAKVDEPVATEN	S	172							
7807	LOC_Os12g18110	EMIPVAANDVAFSVHAVALTAFTLF	S	96							
7810	LOC_Os12g18760	NGGWCGWKKSIDSKGRSFFLKDKFY	S	617							
7813	LOC_Os12g18990	AAVSRRSSAIPSSTSPTQEDVRGGP	S	49							
7816	LOC_Os12g21870	RAKVYKIASRDLSRPLRESTRNDAP	S	560							
7819	LOC_Os12g22620	EDALDLGALAGASFLPLQRRSMKRE	S	176							
7822	LOC_Os12g24020	CSKQEETERVMRSVDAEEACALLSS	S	30							
7825	LOC_Os12g24160	NGGGRGGYTNRGSGGAPRGRGRGNG	S	247							
7828	LOC_Os12g25530	QLRADCSKVQVDSVKPEYELFPLKY	S	363							
7831	LOC_Os12g27090	SASSPIFCNRLTSTNAPPFGSPATS	S	886							
7834	LOC_Os12g30020	GDHGSNKTRSNQSGSRVDFGANDGL	S	360							
7837	LOC_Os12g31160	VVGLTKLRKLGVSLFFDATGDQHQH	S	691							
7840	LOC_Os12g31840	SGPGRGGGVASASASPVPDLFAFFS	S	102							
7843	LOC_Os12g32986	KQYVWEGEAESSSYTIREETDPEKL	S	265							
7846	LOC_Os12g33090	PEKMMTTTSAMDSIGILGQNTSRDH	S	581							
7849	LOC_Os12g34330	SSFKNDADAFVLSSAQSAEKALKLQ	S	350							
7852	LOC_Os12g34500	FDMVKGKNDRLRSEVATAVKKHRAS	S	302							

7855	LOC_Os12g34920	AISHDGAINFSRSVVAVEGNGGVLT	S	270							
7858	LOC_Os12g35620	KSELSDIKTALNSEIEQLRSDFQEL	S	97							
7861	LOC_Os12g36490	GFVSMLEKKDFWSLTIITLVQTAGV	S	60							
7864	LOC_Os12g36730	ETLDIRGARKPNSSKSHKSEAGTRS	S	530							
7867	LOC_Os12g37860	HPNGNRIDVNKRSDIRESFNEKNRI	S	484							
7870	LOC_Os12g38730	HIGFEKEGRRNSSEMVESVIGIAPL	S	223							
7873	LOC_Os12g38970	QGRLLRKMRFMPSSLSSKSHRLLTA	S	416							
7876	LOC_Os12g39650	KNAALDMDVLYRSGETHQGVATYCP	S	53							
7879	LOC_Os12g40010	DRLHRASDAAEASGYAFRDDDGCVS	S	698							
7882	LOC_Os12g40520	EESSQQASSSSSSSSNDHGAVSSSN	S	935							
7885	LOC_Os12g40810	EDREGSERVQRISKRYVLVEGTLYR	S	1525							
7888	LOC_Os12g41920	PLATSTSSKRSLSRLDNIIVEAVRS	S	120							
7891	LOC_Os12g42140	TEKRQFDAAQQASQYMTHHAVPGAA	S	752							
7894	LOC_Os12g42370	PTAAELLRAYCASRGHVSHAGLPDE	S	443							
7897	LOC_Os12g42550	KPAEPEAAAAPPSNPTEPSAPKAAA	S	245							
7900	LOC_Os12g42840	DDDALYPYVCGLSGSTEILYRKPES	S	159							
7903	LOC_Os12g44130	LSQPTDGNKEHASAEVRTVQESYAS	S	576							
7906	LOC_Os12g44150	HTFFGKAAHLVDSTNQVGHFQKVLT	S	233							
7909	LOC_Os12g44340	ARRQSIGGADSLSKTNGFLSKRPSF	S	378							
7912	LOC_Osm1g00400	LLLKSWFLQELRSYMQEEDLERTKK	S	206							
7915	LOC_Osm1g00440	LQRKIDLKRSTSSIGIVERIEYDPN	S	53							
7918	LOC_Osp1g00740	LVKYTPRIKKKKSGLRKLARKVPTD	S	18							
7921	LOC_Os01g01270	HQINMAKEDEEKTAFFTPFGGALSG	T	1073							
7924	LOC_Os01g01800	NIPYGRPTEFLITSADGVEYNLKPA	T	713							
7927	LOC_Os01g03650	SYVQQQSFAAAHTKWTVVQQNTSSN	T	282							
7930	LOC_Os01g04190	ASALVGAANVFGTMIASSLMDKQGR	T	408							
7933	LOC_Os01g04650	PQPQQSHAFDPNTGMYYIPMRTNAP	T	525							
7936	LOC_Os01g06630	AELDRRARDDGKTVIKSGTGGKSLD	T	30							
7939	LOC_Os01g07520	DLKITIGNYNGSTDGKLRQLENMLR	T	187							
7942	LOC_Os01g08410	KKAAVPVAPPKDTERQLSKKELKKK	T	177							
7945	LOC_Os01g09280	VASHAQKYFIRQTNSSRRKRRSSLF	T	158							
7948	LOC_Os01g09790	SDDASSAAADPFTAAVATVARAPAK	T	89							
7951	LOC_Os01g10350	NEKTNENFQVGATDPFPSQHVAPSS	T	266							
7954	LOC_Os01g11040	GSTSHSSETNQSTVRTPDTVIGQTE	T	802							
7957	LOC_Os01g11920	LPPGRLSPVSPLTHSSSSKLPTPSS	T	44							
7960	LOC_Os01g12590	RHYFQAHRVTVVTSYPLGQILHNRE	T	1371							
7963	LOC_Os01g12760	VDLVEHLTAYSNTVVSRAVFGDESA	T	201							
7966	LOC_Os01g13470	GFPGPGQPSIVSTITADVMVPKLVL	T	630							
7969	LOC_Os01g14860	SFGIVFQAKCLETGETVAIKKVLQD	T	94							
7972	LOC_Os01g15020	TPPTENGVPNGRTSTSSATSNPAAD	T	1113							
7975	LOC_Os01g15260	YIKKEFDKNHGPTWHCIVGRNFGSY	T	94							
7978	LOC_Os01g16240	LGTVMRSLGQNPTEAELQDMINEVD	T	45							
7981	LOC_Os01g16430	AHDAPSAANSVETDALARFAVDEHN	T	63							
7984	LOC_Os01g18070	SRHRTLMNIFDKTPHVHRDAFVAPS	T	52							
7987	LOC_Os01g19940	ALGKVIQKTKKCTCFSGSKMKIIGF	T	60							
7990	LOC_Os01g21180	IGYTKMSAKDIKTINKGIGVDNMHY	T	157							
7993	LOC_Os01g23640	VILLRYSSLVCETIPKHRDYHTFKL	T	69							
7996	LOC_Os01g25330	CAGKGAFLLLNNTYRDLKIYGGSQG	T	575							
7999	LOC_Os01g26920	STTTAPFSVNLSTAAARAPRLLLLS	T	35							
8002	LOC_Os01g27990	EYTMSNREVLQATELQAEGGIPEWV	T	240							
8005	LOC_Os01g31360	FPFAVAGDATVGTRGAARIGLSFGC	T	94							
8008	LOC_Os01g31629	HVQQQQYVDPYRTMVLSPQPDHLNA	T	17							
8011	LOC_Os01g33240	VEGEDTTLWDNETNIPDDWTTISMS	T	343							
8014	LOC_Os01g34330	ATAKFDVMEKQLTSPRDHQPDKPLA	T	595							
8017	LOC_Os01g36260	DAIWDQLDRLTTTHIEEVRKKPIVA	T	574							
8020	LOC_Os01g38160	RAFKSGVRDRYTTQELATRRITTTQ	T	526							
8023	LOC_Os01g40050	SLDRCDWLDKKNTIFGKVTGDSIFN	T	129							
8026	LOC_Os01g40110	SKKQKIKWKKIITKVLQTNPDGVLK	T	241							
8029	LOC_Os01g40990	IIKMFGSALQSSTPQLVQLIQLILD	T	281							
8032	LOC_Os01g41430	LILNHPAVGGFVTHCGWNSTLEAVS	T	375							
8035	LOC_Os01g41630	NPTRPARSLSTLTRVVRRGAESTGI	T	457							
8038	LOC_Os01g42294	ALSAARGLAHLHTVHSLVHGNVKSS	T	494							
8041	LOC_Os01g42410	SEDALKEKHANLTGEVVEGQKDTKS	T	795							
8044	LOC_Os01g43340	IVTLLRDVVFDETQFPFTKLHPNAG	T	646							
8047	LOC_Os01g43920	SLHTRTLDELFLTKKLLQKYQIAFH	T	144							
8050	LOC_Os01g47600	PLVDRRRPKKPPTAFFYFMEDFRKT	T	54							
8053	LOC_Os01g48640	HTTDPAILEVAATLALYGPSSDHGS	T	112							
8056	LOC_Os01g49200	AMTPSLANKVSATPVSLVYDKPEVT	T	654							
8059	LOC_Os01g49640	EVLVLVLVLAVVTSPGTVCGASRTA	T	19							
8062	LOC_Os01g51020	ASGKETAVVLSTTKTEKQNKPASLY	T	74							
8065	LOC_Os01g51390	SIPLMVIQSILGTWNRSIGVGNCSG	T	325							
8068	LOC_Os01g53000	SIVDPQTSLYACTVGQKPSKAKYYL	T	830							
8071	LOC_Os01g53560	SISVEYAKSGRSTCKGCSESIAAGA	T	137							
8074	LOC_Os01g53680	HRVTSTRNKVKITDRMWARLLSSTN	T	504							
8077	LOC_Os01g54400	VPRTIETTPFPTTFVQADTASFKQV	T	61							

8080	LOC_Os01g55040	LNQQFVIQLGLFTALPMIIENSLEQ	T	1421							
8083	LOC_Os01g55280	NSPATDVFEVVRTESGDKENQSAAG	T	165							
8086	LOC_Os01g56140	ELQKLKDERKEATLERERREQELSE	T	677							
8089	LOC_Os01g56800	GLQYRQFQREIPTNLVSYQLPGPVG	T	1114							
8092	LOC_Os01g57420	PEKFKNQLSNQKTYLKLACTQGWFC	T	265							
8095	LOC_Os01g58620	VLVVSYFAACGVTAVLFRAAVVKGR	T	237							
8098	LOC_Os01g61180	MNYLRAACGSRQTLEEVMEGDLGAV	T	443							
8101	LOC_Os01g61370	TPDQRVGSFDRITSKRFEDIFLGRA	T	151							
8104	LOC_Os01g61910	QEEVWSLQETFSTSAVIEDNEARAL	T	207							
8107	LOC_Os01g62290	RAKRTLSSTAQTTIEIDSLYEGIDF	T	286							
8110	LOC_Os01g62650	AEREVLINEMLGTTDENEPLQAMMK	T	982							
8113	LOC_Os01g63250	GNLEEQGANKSETSEPNSDNIESAS	T	304							
8116	LOC_Os01g63810	ANMAAQDGNHTATAAAASGFAGSNG	T	317							
8119	LOC_Os01g64450	DCYIRVPDPSNATAVAMGFTATRCR	T	224							
8122	LOC_Os01g65260	EGAGIAAAGDDGTIKLERGLGLVGD	T	126							
8125	LOC_Os01g66130	GEGTPQGKKDAATALFNLSIFHEHK	T	657							
8128	LOC_Os01g67170	DEDDLDQLLELGTSNRRGIESHHGC	T	461							
8131	LOC_Os01g67330	YGAIFNFLGLVITALIQGPSSFNIL	T	261							
8134	LOC_Os01g68160	KEENEPVVTPLKTFPHEMAKIEAPI	T	238							
8137	LOC_Os01g68810	LIYRPVMRLFKSTNGGDTYWPLDDN	T	871							
8140	LOC_Os01g69100	RGSTPNYQPRTQTVMSNVFVPDNVQ	T	108							
8143	LOC_Os01g69900	RLSKALTIPDHTTVYEACRRMAARR	T	78							
8146	LOC_Os01g70320	ARESKDGKDGEVTTLKHELDCAKEE	T	298							
8149	LOC_Os01g71050	KSNEYVAFKVKTTSPKRYCVRPNTG	T	44							
8152	LOC_Os01g71990	EAFSSIGAHILETNAQVVDDSDVIV	T	72							
8155	LOC_Os01g72690	EIGSGSISTVSSTVSSVESEKAAYE	T	77							
8158	LOC_Os01g73480	HLIQQLMVLAGWTTRSPWLQNHASM	T	117							
8161	LOC_Os01g74230	EAPEPNGGPGPPTTGVGPLPACQTV	T	1007							
8164	LOC_Os02g01280	TKDSTTIVGDGTTQDEVNKRVTQIK	T	389							
8167	LOC_Os02g02290	RSGHRVLLFSTMTKLLDILEEYLQW	T	1319							
8170	LOC_Os02g02570	AHFSSTLPPGIDTVWFEYKGLPLKW	T	75							
8173	LOC_Os02g04270	YEERKVGPGLDSTKMHAMRYLLIQL	T	766							
8176	LOC_Os02g05260	TVGVVSRFMERPTVMHFKAVKQILR	T	1142							
8179	LOC_Os02g05450	ELPARSVKENLSTELGISFEKIDKW	T	473							
8182	LOC_Os02g06370	ERLSLPISKAEFTVMRTALSFALPH	T	182							
8185	LOC_Os02g06740	PVPAILMNCDNQTTCEKTIEVCQEI	T	1108							
8188	LOC_Os02g07210	IPDVNNATIDHETLLERLGTYGLAE	T	75							
8191	LOC_Os02g07870	DALAESDKITLETAKLLREDYLAQN	T	512							
8194	LOC_Os02g08300	GTQFEIRVQPNDTIMAVKKIIEEIQ	T	22							
8197	LOC_Os02g08500	NMIRGAQMSTILTGPSGNSFPNISN	T	377							
8200	LOC_Os02g09920	GPKGEQLMELPNTKAAVILEQKYGL	T	583							
8203	LOC_Os02g10790	ESKIEEILRNSATSEFGGVYYYSER	T	531							
8206	LOC_Os02g11780	RSAQKETPSKVQTQQLESVRSKFRE	T	157							
8209	LOC_Os02g12530	REVLRTHDANFATRPRLLAGEVVLY	T	107							
8212	LOC_Os02g12550	ASSREMAREVLRTHDANFATRPRLL	T	98							
8215	LOC_Os02g13560	QQFAGINGVLYYTPQILEQAGVAVL	T	548							
8218	LOC_Os02g14770	ESLRAIPWIFAWTQTRFHLPVWLGF	T	780							
8221	LOC_Os02g15220	LDSKSRPLSHTPTYSARTPSMKKPK	T	467							
8224	LOC_Os02g17320	GMHTIIRDAATTTHDFIFYADRLIR	T	286							
8227	LOC_Os02g17870	EEEEESEESEYETDSEDEQTGMAMV	T	167							
8230	LOC_Os02g18330	EKFNMDKSYPSKTPMVVRSLDVEKD	T	536							
8233	LOC_Os02g18660	GSSKEAALAASPTKGKKKGSHKDKL	T	372							
8236	LOC_Os02g21080	RLPSNLALPSPMTVFALILLTYFAV	T	60							
8239	LOC_Os02g21920	AKGGRPELANVATCNPCKKRGGGGW	T	125							
8242	LOC_Os02g23970	HLPNDVPLVKLVTINKALPGDTPDY	T	445							
8245	LOC_Os02g25060	DESGTIEKAPDGTPEGGGLLVVIVH	T	414							
8248	LOC_Os02g27769	MDVKCQGCFNITTVFSHSQTVVVCP	T	47							
8251	LOC_Os02g29410	NFSFDTNAMTLPTPNSNNQPSARRA	T	68							
8254	LOC_Os02g30900	PRVEKLSAGAEKTRVKSNAILREPS	T	83							
8257	LOC_Os02g32350	QKQYRAMIEERDTSGGKSKGQGTGT	T	891							
8260	LOC_Os02g33610	DEFGVQKIIPDTTFIKKWSHKIEAV	T	130							
8263	LOC_Os02g34500	PILLEENNESAATGNIIEEKNDVQL	T	453							
8266	LOC_Os02g35130	AREQEVHDAIGYTEEQISELKKEIQ	T	235							
8269	LOC_Os02g37420	LEDARQHANANMTVMLIGNKCDLSH	T	113							
8272	LOC_Os02g37870	HLKGSRPLITFSTNFDEQPHWQLVK	T	179							
8275	LOC_Os02g38840	SYCMNPSSHSGWTRVIVEKPFGKDL	T	175							
8278	LOC_Os02g39030	KWNEENLNDIESTKPVREKITEPKT	T	30							
8281	LOC_Os02g39520	LVALKLLRQAEKTLLDDGDRGRQAG	T	683							
8284	LOC_Os02g40430	DAFNNQSPDVRKTVVFCLVDIYIML	T	1346							
8287	LOC_Os02g42210	EAIDDASLSASSTRHALSLAARRLS	T	70							
8290	LOC_Os02g43020	HRYSEALADAEKTVELKPDWAKGYS	T	62							
8293	LOC_Os02g44410	GDDRVMANFFPMTLKGQARGWLMNL	T	240							
8296	LOC_Os02g44770	DSTASTPRGASRTWSSWTNSPRSPP	T	89							
8299	LOC_Os02g44930	DKKESTSSKKAKTHDDGEGSDKSKS	T	114							
8302	LOC_Os02g46956	TDSDQVAKAAMMTAEGAMNFMRSTI	T	269							

8305	LOC_Os02g47140	SPKKIGEDIAKETAKDWKGLRVTVK	T	50							
8308	LOC_Os02g47900	SQIDRERVQTTGTTAYVIDATRYGN	T	447							
8311	LOC_Os02g48340	TIQAPFLVAPVPTHPHLVLPAHSPQ	T	272							
8314	LOC_Os02g48730	TLKEGSLYKLKFTFSVSNNIVSGLR	T	286							
8317	LOC_Os02g49080	GPRGNSLKRVEATTDCRVLIRGRGS	T	171							
8320	LOC_Os02g49450	AEEEKMDAKKEVTETSQATTAEHKD	T	219							
8323	LOC_Os02g50320	TSSSLVIKHAKGTSRSFDGGTRSLD	T	429							
8326	LOC_Os02g50790	SPSPFQDGAASQTPSVESADVDASA	T	632							
8329	LOC_Os02g50910	VLQRKTEEAAMATKRLKESLEAKKS	T	26							
8332	LOC_Os02g51490	NQSNTVLESISETDEDTGAVKQRND	T	245							
8335	LOC_Os02g52150	LGRLLSLMDDLATPAGRAGAATLRR	T	108							
8338	LOC_Os02g52780	AAVPPPPPPQQQTPMLFGQSNVFPP	T	189							
8341	LOC_Os02g53550	FKLLKRSGPFTWTEEAERALTQLKA	T	1051							
8344	LOC_Os02g53760	WAVATTLHDPIVTTKPFSTCYVFKV	T	234							
8347	LOC_Os02g54280	SLNVEQCSITSRTVQKMADALHEGS	T	932							
8350	LOC_Os02g55400	KKADIGIAVDDATDAARSASDIVLT	T	544							
8353	LOC_Os02g56530	DEVDPFQIPSDYTWVDANEKKRRMK	T	588							
8356	LOC_Os02g57390	NDRMGGGERNGLTRQQSSEWLGSGA	T	175							
8359	LOC_Os02g57600	GNDWEVVQLTASTYASAPGPRMSEP	T	27							
8362	LOC_Os02g58080	VQFGWALQLSLLTPYIQTLGIDHAM	T	84							
8365	LOC_Os02g58220	SKSRGASSTMPLTVKQISEAQQSGI	T	41							
8368	LOC_Os02g58490	TDRPTIIFGADVTHPHPGEDSSPSI	T	724							
8371	LOC_Os03g01420	PPPFVSDRTCCFTYPKITSYAVVGS	T	217							
8374	LOC_Os03g02150	NNLVACTTFFSETTRAMLCLVRIET	T	959							
8377	LOC_Os03g03430	LPVVKNQHLLLCTLLIGNSLAMEAL	T	75							
8380	LOC_Os03g03560	QISGKHCKIYRDTVLGELNRNEPVP	T	116							
8383	LOC_Os03g04570	TKQIVKVIPLLATMFVPCTLIAQTN	T	350							
8386	LOC_Os03g04960	KPTPQLELFNSMTKKKELFEPLVEG	T	18							
8389	LOC_Os03g05390	VAEKRWRVFVWKTAVYLITLGMLIA	T	361							
8392	LOC_Os03g05790	RPLPLVAILEGITRGTQKYLCKRYS	T	631							
8395	LOC_Os03g06220	SAGGCQVLLFSATFNERVKDFVTRV	T	291							
8398	LOC_Os03g06710	KMLASNTPPNDATFKALKIILVKRG	T	740							
8401	LOC_Os03g08270	SIEERTLSLINKTTALNPNAQEFVP	T	14							
8404	LOC_Os03g08830	DRRMLPPLSTCQTKKEPPPCIKMFC	T	265							
8407	LOC_Os03g10070	GINLNEASCTMDTDRPDMPVKSPFA	T	459							
8410	LOC_Os03g10400	SGGGVGKYLKARTAGFADVVADDSN	T	432							
8413	LOC_Os03g10800	DGVMVVEDLSLSTKTTTKLDGAANS	T	527							
8416	LOC_Os03g11140	PVVTLGGIDLNNTGSVVVKEDRKLL	T	111							
8419	LOC_Os03g11380	EAIREDATKTLVTLHRQGEQITRTH	T	83							
8422	LOC_Os03g11580	IGTNPDIGHQLLTEAAPCTERSLED	T	317							
8425	LOC_Os03g11790	SLVELTCHILQNTTSLECLTLDTTR	T	442							
8428	LOC_Os03g11970	HGIQPDGLMPSDTTPGIARDAFNTF	T	40							
8431	LOC_Os03g12360	PETEAKVAELMATYSGKGRVVMLGV	T	332							
8434	LOC_Os03g13560	RKQVPSESVSRFTTPLTPPPMPWGP	T	446							
8437	LOC_Os03g14860	AAELEKAAEPEKTPPPPPAAGAPSG	T	142							
8440	LOC_Os03g15033	RFQTPSSKVIWQTAITFVLLGVLTL	T	184							
8443	LOC_Os03g15650	GSSKTSDDIKNDTLYSEVDNTGTQQ	T	446							
8446	LOC_Os03g16260	RAKALLTTGGPVTPSDDGQRVRRAL	T	65							
8449	LOC_Os03g17020	HSTPSKGSNQNGTTGGSYWTEGGFR	T	441							
8452	LOC_Os03g17180	QEALDTLVMGNKTTILIAHRAAMMK	T	1352							
8455	LOC_Os03g18180	FDKEGNQWKERGTGTVKLLKHKETG	T	72							
8458	LOC_Os03g19250	VAEVVMVDQEFFTLAEESLKILAEK	T	136							
8461	LOC_Os03g19570	RGKSNSNAFYVETNRKDEHVLSDFC	T	431							
8464	LOC_Os03g20860	RFLYRNTLGRFKTEKDLLNDAEQSM	T	96							
8467	LOC_Os03g21160	AATAIGSEGDFTTGSSCNIGGSAVG	T	632							
8470	LOC_Os03g21530	KSLLIKCLVKHYTKQNLSEVRGPIT	T	111							
8473	LOC_Os03g22350	KDFVNVSGPLGVTHFFILTNPKSSP	T	107							
8476	LOC_Os03g22890	TRIEPDRRWFGNTRVVNQKELEFFR	T	89							
8479	LOC_Os03g25140	TTRTIDGIIVNSTRREHGGKGVPLR	T	76							
8482	LOC_Os03g25430	DIDFPSDPNHATTQAFSSAAAAAAA	T	50							
8485	LOC_Os03g25720	EDLVLQDTFAQETAVTIEDPNMLRY	T	96							
8488	LOC_Os03g26000	EGLTENSDKSLHTLFAVLNTVFSKL	T	198							
8491	LOC_Os03g27800	ALAQIELAKESETRTLERLQQETKE	T	652							
8494	LOC_Os03g27930	KNGRRVSYEAANTLIDDDSRTCTGT	T	118							
8497	LOC_Os03g29085	VEELPSVLWAVRTTPTTSNKETPFF	T	863							
8500	LOC_Os03g30460	IQALEKLQLDIATSTDLLGVETRST	T	304							
8503	LOC_Os03g30990	PQIQKLLYAVIMTSRKLRHYFQAHR	T	1050							
8506	LOC_Os03g33110	GGNSSASPAVASTDVVVVPRSREAT	T	184							
8509	LOC_Os03g37330	EGFSEEFLDAFLTLLRRAHRHSRIA	T	78							
8512	LOC_Os03g39400	KEYLDISCLMLATMSPELQRQYEAL	T	174							
8515	LOC_Os03g40550	STLYHLNVNGVQTRAIKMDPSAFTA	T	296							
8518	LOC_Os03g42810	KTIRIQKACEFTTSSVLCVCIITWN	T	46							
8521	LOC_Os03g43390	LCMLSLILGSKITDAAVASIVRSCA	T	364							
8524	LOC_Os03g43800	AMDTVDTTQGATTQQADLASKGRSR	T	644							
8527	LOC_Os03g44420	LLRGGDEAAVVATRKGSPNGSPLEG	T	72							

8530	LOC_Os03g44500	ETSSSSSASAPSTPGTPTVAPAPAA	T	48								
8533	LOC_Os03g46040	ADSMTMSYDPQATESKQSELDLPLS	T	108								
8536	LOC_Os03g46970	APEPNGGPRPPTTGAGPLPACQTVP	T	1181								
8539	LOC_Os03g48310	IGSHRLSQQGAITKRMTAIEEMAGM	T	315								
8542	LOC_Os03g48674	SDYVWRFNECRNTIPEITDASVIRA	T	460								
8545	LOC_Os03g49500	IRSTLDRHTILKTTLVELGGTLGLE	T	165								
8548	LOC_Os03g49640	KELRIGETFEGFTGTSSLSTTNDDD	T	345								
8551	LOC_Os03g50180	GATKKPQAQIVVTSSDGKTTEKKNN	T	48								
8554	LOC_Os03g50300	AEDVESGSSDVETKYKKMYEDDINP	T	570								
8557	LOC_Os03g50730	LNDKTDDRQKDLTFIPLAPACVVIR	T	519								
8560	LOC_Os03g51080	AHLFNAPLGDSETAVGVGTVGSSEA	T	117								
8563	LOC_Os03g51250	YFNHIKSVADSLTALAWVGFLGKDC	T	143								
8566	LOC_Os03g52310	LLAALDYKQAFSTLETSDDNYNEAP	T	423								
8569	LOC_Os03g53060	RAVLKTHDANFATRPRLLAGEIVGY	T	102								
8572	LOC_Os03g53630	QPLTRRGSIVNKTEDAVSGLDQNIC	T	283								
8575	LOC_Os03g53970	SCFDDEYCSSPRTPVLNEETAFSLE	T	184								
8578	LOC_Os03g54970	ARHPQMELPMTCTSHVLLPPPNLSN	T	158								
8581	LOC_Os03g56070	EAGRENRNGRENTAADENACPEAAS	T	310								
8584	LOC_Os03g56790	KQKQVVVEKTGATTESYDDFLASLP	T	55								
8587	LOC_Os03g56990	SQGGALLGPNDSTQYRSIVGALQYL	T	1059								
8590	LOC_Os03g57160	ECESCGAHLIFSTLTSWSPAEVSNA	T	137								
8593	LOC_Os03g57310	YRETVARRYYTVTGEQPDEATLDNL	T	174								
8596	LOC_Os03g57854	AASPTSSRSVTQTVNGSHRFVIQGY	T	46								
8599	LOC_Os03g58480	CSDAPPTPEAANTTAASSRSHRHSI	T	319								
8602	LOC_Os03g59390	AGRPGLMVLRDPTGRDLGARYELGG	T	56								
8605	LOC_Os03g59500	ANRTTPSRATGETPFFLVYGAEAVL	T	1752								
8608	LOC_Os03g60530	SCLLLRFSDGSFTTSFITTIGIDFK	T	41								
8611	LOC_Os03g61160	EKKVRLMRNRSLTREEVDAFWRRQQ	T	28								
8614	LOC_Os03g61690	NLREAASGPEGVTADAIRDAFLATE	T	124								
8617	LOC_Os03g62340	SSRQSNDSTEPPTEGNGELYCILPP	T	347								
8620	LOC_Os03g62500	RYYRAPELIFGATEYTTAIDLWSTG	T	262								
8623	LOC_Os03g63710	LKNMAGETGKKLTSIASNFISDLDR	T	398								
8626	LOC_Os03g64320	FVSTPEVLERVTTIESEILQIEDAI	T	96								
8629	LOC_Os04g01480	ASSSAVLAAANNTGGAQHPMWKTSL	T	44								
8632	LOC_Os04g03430	WSRFTNLVQSGPTLSIPDYALLQHF	T	89								
8635	LOC_Os04g04680	PFDRSVNLLDLGTIARTRTKPVLSL	T	26								
8638	LOC_Os04g07250	ITKATTSHHWFKTLNDLSTLVGFAD	T	409								
8641	LOC_Os04g09860	STSCVVNIGAQVTQVVCVEDGVALP	T	299								
8644	LOC_Os04g11810	YGNCVTSQVVAATSGEVANGDANDV	T	317								
8647	LOC_Os04g14510	EAEVAELLAELPTHRPRPLYRRWAE	T	108								
8650	LOC_Os04g14640	NFKSSHCKLLKFTSEEGSSAFFLWR	T	91								
8653	LOC_Os04g17064	SIIDNAQSLELATLLAGQIARAATV	T	88								
8656	LOC_Os04g17700	EFDRQELRKLVRTVHMVQDNIENEH	T	234								
8659	LOC_Os04g21240	SRPELVRQSLAWTCFVETPRAEPAP	T	91								
8662	LOC_Os04g23330	PITDSDEKFNCYTSREMVNNIRTIQ	T	172								
8665	LOC_Os04g23990	SRLSPPAVAFALTSASKPLPSGSRT	T	548								
8668	LOC_Os04g26660	APPRSNNGGKDVTRGRVNHVTAEDV	T	679								
8671	LOC_Os04g27520	LLSELTLKSPRATMYCEADQDQLRR	T	1750								
8674	LOC_Os04g28090	RWYEWLDPSIKKTEWTREEDEKLLH	T	60								
8677	LOC_Os04g28420	KLKEYREAEKLCTKVLELESTNVKA	T	484								
8680	LOC_Os04g29950	ACSGAGCCQTTITKGFNAYQVEFQN	T	186								
8683	LOC_Os04g30780	VSQCDFDRLKLSTAPFMERNLEFLI	T	231								
8686	LOC_Os04g31190	KDALAFEEKFFSTLPAYHGLTHCCG	T	277								
8689	LOC_Os04g31900	AEIVQAGVLRCHTPLHSSGKLTICV	T	505								
8692	LOC_Os04g33300	SLEKIGVETRVQTALMMQEVAEPYI	T	159								
8695	LOC_Os04g35240	FEDPDDPKNFRKTIGRIMSIQYKIP	T	212								
8698	LOC_Os04g36790	AAPPGATGGAGGTSRRMASVNDASA	T	343								
8701	LOC_Os04g37950	DLSVDIEQNSSITSCLKNFCSTETL	T	211								
8704	LOC_Os04g38480	ISGTIPNELGNLTNLVSLDLYLNNF	T	122								
8707	LOC_Os04g38870	STLIMQLLRDNLTLWTSDISEDTAE	T	236								
8710	LOC_Os04g39440	VDSRPVKLQIWDTAGQESFRSITRS	T	66								
8713	LOC_Os04g40080	ELVDSSRAIVAATDAEAGGSRRVPE	T	49								
8716	LOC_Os04g40950	DSKTLLFGEKEVTVFGCRNPEEIPW	T	77								
8719	LOC_Os04g42010	SEEEEANAVLKNTLVFAEADLEIKP	T	184								
8722	LOC_Os04g43130	KGFTFSEVNCWRTNNSKVVCCHFSS	T	514								
8725	LOC_Os04g43430	ATALLKKMPTPSTVAWNAVISGHAQ	T	290								
8728	LOC_Os04g44960	LRNVRWPPRFRPTIAEKYDGSVNPA	T	240								
8731	LOC_Os04g45570	GGGAGGEVVERWTVVCEPWPDAAAG	T	107								
8734	LOC_Os04g46892	AVVAVTCDKEIITGGHADGSVKLIS	T	96								
8737	LOC_Os04g47320	CLLVATVIRLVKTLLLKVLASSFHV	T	451								
8740	LOC_Os04g47740	DDGEPNPRRRARTPPPPPRQSPRRE	T	29								
8743	LOC_Os04g48290	CGQDAAIAAVAETYIFASLPDLVLQ	T	203								
8746	LOC_Os04g49194	PSNPAQFKEIMSTYCREVRQLGLRL	T	155								
8749	LOC_Os04g51370	CSYPWKNSELTETEKAERVILVDFL	T	431								
8752	LOC_Os04g52050	HRDPVERQAASSTGHMPAAITDSEP	T	475								

8755	LOC_Os04g52200	TRVFALVVFVEATEARHAFKKLLYT	T	608							
8758	LOC_Os04g52570	GDDRDFDANNNLTARVPSWKDEVSS	T	147							
8761	LOC_Os04g52940	IEVLSGTSVPESTIGELLPGSTEIS	T	739							
8764	LOC_Os04g54564	RLPALLIDATTTTMAAPTATDGGEP	T	100							
8767	LOC_Os04g55040	GQTQRLNVVPTVTMLGVMKARLVGA	T	15							
8770	LOC_Os04g55450	QGGCPWRVHGYKTQHDTLWVASRVE	T	295							
8773	LOC_Os04g56290	PTSNKAYVDLILTHTEEDLKIWPHS	T	139							
8776	LOC_Os04g56720	VVGADDSCISWGTAQNGELGYGPNG	T	368							
8779	LOC_Os04g57520	RASTNLEYESDKTFKEAGLANSMIN	T	443							
8782	LOC_Os04g58280	TNEALLVIEAYRTLRDRGPYPADQS	T	116							
8785	LOC_Os04g59060	FGKSSPHLQGGSTGSFANSHDIPRF	T	92							
8788	LOC_Os04g59290	RMLEKIASKEPQTTTELFQLADRVA	T	359							
8791	LOC_Os04g59400	PQSEVETDNADLTKDEQDLPAWYGI	T	232							
8794	LOC_Os04g59624	LQVFKGALFRTATEHKEQTRYFSKR	T	393							
8797	LOC_Os05g01350	VEALRMLMQMVLTNEEELKLKYFKD	T	521							
8800	LOC_Os05g01910	EAILTRIKAHLNTLKKYTYGKHIVA	T	847							
8803	LOC_Os05g02260	SLPEKHSALADATSGVAAENADNLS	T	42							
8806	LOC_Os05g03060	EEMRRSGESTTSTLESESWVMENLE	T	139							
8809	LOC_Os05g03740	AIRADLDHSFLSTKRNKALWEAASA	T	80							
8812	LOC_Os05g05240	MQELNIQRPPTPTRGRPQSASDRNS	T	875							
8815	LOC_Os05g06280	DNYNAVANEKESTARENNVAKIKVV	T	183							
8818	LOC_Os05g07130	WALSFVVPPDHETLDFKFLLKPKDA	T	105							
8821	LOC_Os05g07980	RESEAVSLREEATNRSDGMHPELPG	T	202							
8824	LOC_Os05g09630	VATSPKSMQLGQTLALEVLDLLKTA	T	39							
8827	LOC_Os05g11730	RYYRAPELIFGATEYTTSIDIWSAG	T	239							
8830	LOC_Os05g11980	TQFLLNLFRSFDTHKQPKSDLADLL	T	476							
8833	LOC_Os05g12570	YLDDLRGRFLLQTDTNCLDQSRYVM	T	487							
8836	LOC_Os05g14750	LLVKEPHKRIAFTRGATEIKQHPFF	T	490							
8839	LOC_Os05g15190	PENSSSGDRDTSTPGSRPVDCSDLS	T	245							
8842	LOC_Os05g17100	GPASLWWDHFQATQPEGQPITWARF	T	424							
8845	LOC_Os05g19020	VLTASFNSLLRWTITALQAKFSMKD	T	195							
8848	LOC_Os05g20100	DLESWLLRSPMSTFPYFMIVAIEAG	T	68							
8851	LOC_Os05g22390	PFPVNMVHTAGQTADRARARGFRVN	T	299							
8854	LOC_Os05g22870	KKGIASNTSMYNTLINAYCKANQIE	T	414							
8857	LOC_Os05g23080	EIGMDFITGLPKTQGGYDSIWVVVD	T	1440							
8860	LOC_Os05g23860	QGFARLSAVYGGTYMLNKPDCKVEF	T	248							
8863	LOC_Os05g26890	PDRVDRVFKIYRTTALDQKLVKKTF	T	317							
8866	LOC_Os05g28280	DNAEIKAWLALGTKTKKKSGAKKKK	T	359							
8869	LOC_Os05g30420	DYKAMFMDIKLNTMQHSVGTLKKLC	T	168							
8872	LOC_Os05g30810	PQLFRQMEQDVETVINVLQPGPIGI	T	24							
8875	LOC_Os05g31056	AFSHSNHSSSFSTSGNDMPSFSFKT	T	203							
8878	LOC_Os05g32580	RCYWGYSFENLATENSIDEDGRGID	T	349							
8881	LOC_Os05g33380	ENRRSLRELLFCTPGALQYLSGVIL	T	62							
8884	LOC_Os05g34090	HTWPLGHIDLPVTFGGSANFRTERV	T	635							
8887	LOC_Os05g34780	VLRRSSAATKRRTRTAATWRPRRRS	T	565							
8890	LOC_Os05g37500	IPKLVLQIVTVETLLRLHKPNNELA	T	1454							
8893	LOC_Os05g37930	DGERDAPPKAEVTVGSPQSTLCGLP	T	71							
8896	LOC_Os05g39390	FRDAGIHGHAFLTAGNLAKLSEGEY	T	451							
8899	LOC_Os05g39580	TIVRCNLQSLARTAEQLALAAPRAA	T	205							
8902	LOC_Os05g39780	YYGAYLRWYRSVTRWRCFPPQGDST	T	772							
8905	LOC_Os05g40200	YLQHNNLSGIIPTSLSSNLTFLDLS	T	136							
8908	LOC_Os05g40820	DIHAEAVKKRRRTTKKPYSRSIVGA	T	76							
8911	LOC_Os05g41172	NLDDSEDDYIFQTVCPGEKTLKFNF	T	554							
8914	LOC_Os05g41480	EATRLVLIQILLTSKGISLNPITSL	T	196							
8917	LOC_Os05g41670	GVSYGALSNAIQTILSEKSGRVTNK	T	374							
8920	LOC_Os05g43280	SAEGTTDAKANGTPNKDEPVTNCVA	T	712							
8923	LOC_Os05g43860	FSDALSVADSCLTVNCSSASGLSDA	T	193							
8926	LOC_Os05g44270	SSSQPALPPSPRTVVPRTIDTTPFP	T	67							
8929	LOC_Os05g45280	VSGGMSMPQILSTLDPQTILGDRTG	T	257							
8932	LOC_Os05g46330	AWQWLPFTSSARTDNLQLYHWVRVV	T	84							
8935	LOC_Os05g46720	ANYGRQIVTISSTDGKIIRYAGPQY	T	359							
8938	LOC_Os05g47490	TIERMPTINSSGTDGAVLENIKGDI	T	380							
8941	LOC_Os05g47980	LGMDELSEDDKLTVARARKIQRFLS	T	476							
8944	LOC_Os05g48310	RQGQRVRLYVRGTILGYKRSKSNQY	T	16							
8947	LOC_Os05g49050	PLISRRFAERAFTVGIGGPVGTGKT	T	84							
8950	LOC_Os05g51280	TRNKIPKERFRITAVDAVGLPTSPR	T	35							
8953	LOC_Os05g51690	QQPPPPDAASCIYTTALPLIADPPDI	T	31							
8956	LOC_Os06g01680	SKDAPVSIRGSATSGPNYEFAETGK	T	685							
8959	LOC_Os06g02160	PRFFLPMESLPLTSSGKIDYMKLSS	T	553							
8962	LOC_Os06g03676	HRATVMQRKSLQTLFDNKVKGLVGE	T	156							
8965	LOC_Os06g04560	LSMTASDAEDPQTLRMVAGAIANLC	T	733							
8968	LOC_Os06g04990	NINPTGQALIICTAAGMAYFVAADK	T	67							
8971	LOC_Os06g05250	SIIPVINKIDQPTADPDNVKAQLKR	T	200							
8974	LOC_Os06g05660	EAKYQKLYEPLYTKRYNIVNGVVEV	T	99							
8977	LOC_Os06g05850	KSQALADFVAEWTPAPEPVSIPEAS	T	1345							

8980	LOC_Os06g06770	EREDVDPCRIGITGESLGGMHAWFA	T	269							
8983	LOC_Os06g08140	AGDCRAVLSRCGTAIEMSVDHRPCS	T	191							
8986	LOC_Os06g08740	EGSDEEFSSAASTVKTTGPAQPPAM	T	496							
8989	LOC_Os06g09450	IIGNYSDGNLVATLLAHKLGVTQCT	T	418							
8992	LOC_Os06g11970	NMLTQLKCESDVTEDLTNTSSKAPV	T	102							
8995	LOC_Os06g12080	DGELDFYSAVALTEKKFLDKFVHSC	T	346							
8998	LOC_Os06g13720	VLVCENNHYGMGTAEWRASKSPAYY	T	240							
9001	LOC_Os06g14160	GRSSATKASGAATTSSASSRRRARR	T	54							
9004	LOC_Os06g14510	WGVKDKIKQFSETFRSGSWVGATGK	T	132							
9007	LOC_Os06g16250	ESEDGAAPEMAATEAEFYRAQVEEF	T	411							
9010	LOC_Os06g17820	VDDLVVKTRNQETLLSDLAETFESL	T	993							
9013	LOC_Os06g19390	VTVVHLIIMDYGTTVIQEDIPGYNG	T	585							
9016	LOC_Os06g20370	YSKMKEIAFKKQTELEDIYAGAHMV	T	318							
9019	LOC_Os06g21440	FGLITAENTFQRTVQGALNNQLGNN	T	774							
9022	LOC_Os06g22100	QPGASPPGSLVATMRGQFEILSLTG	T	166							
9025	LOC_Os06g24440	YKFHQVYMEMSATDREMIGARIRDT	T	583							
9028	LOC_Os06g24810	KAPEPNGGLRPLTTGVGPLPACPTT	T	1243							
9031	LOC_Os06g26190	RNKSIAKTDKGETYHLSRCTEIKRD	T	218							
9034	LOC_Os06g27830	CPNAACGSSQGLTDRCVRHGGGRRC	T	208							
9037	LOC_Os06g28100	VYFVSEALRDANTRYPQAQKMLYAI	T	1289							
9040	LOC_Os06g29410	KSRKVHSSPQRITLDKPEAKGRGRG	T	548							
9043	LOC_Os06g30320	SENERLRAELNHTLSSINLYKEYKK	T	817							
9046	LOC_Os06g30901	IEENLFSMLDEETDSDPLCNKMYLY	T	1070							
9049	LOC_Os06g31250	KFDTAYNAIIGRTALAKFMAVSHYA	T	674							
9052	LOC_Os06g33520	LPKQRQTLLFSATVPDEVRQVCHIA	T	650							
9055	LOC_Os06g34440	NADTTGPGHDGPTNGVNNYNNTEDI	T	420							
9058	LOC_Os06g35730	RCKSRVGIQHRVTKEDAMKWFQVKY	T	157							
9061	LOC_Os06g36360	RLGVHGPGADIDTLCVGPSYVNREE	T	116							
9064	LOC_Os06g36730	KRHAIEDDLHALTQNPGESLRDYVQ	T	540							
9067	LOC_Os06g37440	LDEWKRLYSNTKTNFREIAIKGFWD	T	282							
9070	LOC_Os06g38500	MPSPAAAAAALATVLAGGGGARTRQ	T	13							
9073	LOC_Os06g40440	ALALYAGLVWETTRDADRADAYFTR	T	215							
9076	LOC_Os06g40840	TIDTSAARDRILTVIDSSIFEPSEL	T	351							
9079	LOC_Os06g41120	SPLLDLAGGGGATTTWMATTAWMGR	T	41							
9082	LOC_Os06g41860	LPGRFELLSVTGTVLPPPAPPGASG	T	138							
9085	LOC_Os06g42790	MAASASAAATLPTTLLLRRLLLLSP	T	13							
9088	LOC_Os06g43370	LRAALCALVADSTVRAVVGERCAGL	T	190							
9091	LOC_Os06g43790	KQPKVLGDQQRPTELRSGQEPRKTR	T	1556							
9094	LOC_Os06g44200	FRGGLRPPPPPPPTPFGWEEAARLER	T	63							
9097	LOC_Os06g44970	TITLFSLSTLPNTLVMGIPLLRAMY	T	121							
9100	LOC_Os06g45360	FGDKGYLAFEHITWTPYQTKLIDTT	T	592							
9103	LOC_Os06g45990	AAAAAAEVAAPETKEVTAKAAADEA	T	22							
9106	LOC_Os06g46450	MATAVEHLVTGETMQISTSREQRRS	T	252							
9109	LOC_Os06g47320	DKGGTTISNDGATIMRLLDIIHPAA	T	67							
9112	LOC_Os06g47730	FPFSPFLLSLSHTFLLSRQAASWPL	T	21							
9115	LOC_Os06g48640	ILGKYHYMLYILTLAMQPRMLLTVD	T	814							
9118	LOC_Os06g48780	YTFVCRSSGDEWTAKQLKGELEASA	T	16							
9121	LOC_Os06g49430	NDQERSADSVARTTVSPPMSQDAQQ	T	504							
9124	LOC_Os06g49800	ISDAIDLLPRLATGIDVNVMFRKID	T	136							
9127	LOC_Os06g50030	SAVVKKGEYKGQTVAVKIIAKAKMT	T	200							
9130	LOC_Os06g50580	SSLLLGVRGYTSTLKNASTASCRLS	T	26							
9133	LOC_Os06g50706	KRQLYHVTTKTITLGGAKGTVGWVD	T	209							
9136	LOC_Os07g01720	EIADRDEKAMADTSKGSSIEISHSI	T	207							
9139	LOC_Os07g01930	VPTKNILFSTATTSSDRDDGSQSKE	T	51							
9142	LOC_Os07g03230	MDKQFLKDPSEITWDIVDKKLKEIV	T	254							
9145	LOC_Os07g05050	HANGGKCIVFTQTKREADRLAYAMG	T	337							
9148	LOC_Os07g06130	AILCQYLAACIGTVTGRSLAEICHQ	T	84							
9151	LOC_Os07g07194	SFKDSYGKDKIDTREHRLRFVDSSP	T	238							
9154	LOC_Os07g08930	AISGSSPKMPNTTGINPSRRAGGRS	T	20							
9157	LOC_Os07g09384	YKTPSRGPSPVPTPALAPASPPRHR	T	180							
9160	LOC_Os07g10200	VAVLSFRQLRTETMGHGGSDLGYEG	T	43							
9163	LOC_Os07g10540	TDITRTVHFGEPTPRQKECFTRVLQ	T	540							
9166	LOC_Os07g11290	ASRSQENEPNADTQVEEKWERRVQG	T	181							
9169	LOC_Os07g12250	RYFHNRLKPAKLTWTAMYRKQHKKD	T	52							
9172	LOC_Os07g13340	TAFRTPKGNFYYTVMPFRLKNAGAT	T	2030							
9175	LOC_Os07g19130	FVGGIDTTAVTVTWIMSELMRNPRV	T	41							
9178	LOC_Os07g20580	RAILSQTASVRQTVMFSATWPPAVH	T	275							
9181	LOC_Os07g22610	PQYSWGSAVLAVTYAGLCDACVRNS	T	1008							
9184	LOC_Os07g22750	LLAKDAPFEFDDTCLKSFEILKKAL	T	239							
9187	LOC_Os07g26440	AQDQIAKGGYSITLEPSSGVGAPWF	T	61							
9190	LOC_Os07g29520	VRGARLQGYLTGTFEKPKAEVTVTA	T	52							
9193	LOC_Os07g32400	YITDYYGSYLHQTKLWIVMEYMAGG	T	84							
9196	LOC_Os07g33420	MLALRSEGKHSLTVEDIVDNAILLL	T	196							
9199	LOC_Os07g36420	RFVRRPRPPAGTTHPDLEFFQPGPH	T	277							
9202	LOC_Os07g37750	LCMEGFRAGIHLTRVLGMDTMRYAF	T	819							

9205	LOC_Os07g38120	PSGHRLVVLREPTGRDIAARYELGG	T	67							
9208	LOC_Os07g39230	AAAKRILRYLKHTVKLGLKISKSNS	T	1143							
9211	LOC_Os07g39470	AARPGGPPTVRITGIDDSVSAYARG	T	315							
9214	LOC_Os07g41230	LHPQQLLAKDLNTKDLETNIIVLVH	T	122							
9217	LOC_Os07g42400	DFNYFGDVLCLDTTYKINGYGRPLS	T	350							
9220	LOC_Os07g42490	LVACLLAHKLGVTHCTIAHALEKTK	T	435							
9223	LOC_Os07g43316	EDAMETKHMDSKTIPDYLEGKSEVT	T	229							
9226	LOC_Os07g44640	YDENMEMQQKRDTQVITVVHTKAEE	T	353							
9229	LOC_Os07g45360	RQDAVDNLRAGKTWVLIATEVIARG	T	436							
9232	LOC_Os07g47100	FLGNFFYLFLSSTFLGVFAGLLSAY	T	225							
9235	LOC_Os07g49040	FQRKGEVSGTTATLVVVDGFTVTVA	T	136							
9238	LOC_Os08g01900	LWLDEGFTICPKTRQRLGHSNLIPN	T	273							
9241	LOC_Os08g03360	NVPPSTSGAKDNTQSAVRLDQENDP	T	253							
9244	LOC_Os08g04920	GWDGANKRAKILTAASVVCDFHGEL	T	224							
9247	LOC_Os08g06140	SNRATEAGYWKATGKDRVIRSKGDK	T	96							
9250	LOC_Os08g08080	EEPEDLSPPRRRTRHDSHEPKDKLP	T	301							
9253	LOC_Os08g08550	LQEELGTRLNFSTAYHPQTDGQTER	T	1519							
9256	LOC_Os08g08820	SMEFQVPSPLVPTRESYFVRYCKNN	T	423							
9259	LOC_Os08g09910	SRHLLDGMSRRTTTARWVPRDRPEP	T	25							
9262	LOC_Os08g12760	HIILENNDNKPVTNSRDTQEVKLEH	T	453							
9265	LOC_Os08g14050	GSTRRRRSPSATTSAAQAQVGMAGE	T	121							
9268	LOC_Os08g15660	GPSMTGSENAVVTTLGDSMSKDPPA	T	152							
9271	LOC_Os08g16610	DIDEVNNDPMDVTEESSPFVSKNIT	T	254							
9274	LOC_Os08g19310	TMKSNGIQPTNVTYGSLMYWMCHAG	T	644							
9277	LOC_Os08g23020	SINAVRDGPPETTTLTSTDTERGST	T	933							
9280	LOC_Os08g24760	KNGSYRTVKNPQTVFIHPSSGLAQV	T	980							
9283	LOC_Os08g25670	PFQGKLYMLDQATAYGGPEVLQIDP	T	239							
9286	LOC_Os08g28880	VIPTFDIKLLQPTIFSRFDAQKDAS	T	197							
9289	LOC_Os08g29650	SKSKSPTPVRRSTSRSPPPKKSDSR	T	36							
9292	LOC_Os08g32050	RNLEPTRITSEVTRSWCPIHKTRKH	T	60							
9295	LOC_Os08g33190	VGGGDGVCVAPGTSVVVVEVATVSA	T	254							
9298	LOC_Os08g34090	EIRACKERGIQRTSPTRDVYCPIHK	T	186							
9301	LOC_Os08g36500	RMVKWVKRIIVTTAESDNYYHYKDN	T	319							
9304	LOC_Os08g37280	SNQHEDDSTIDGTLASHAEIRVMVR	T	295							
9307	LOC_Os08g38410	HEFNGKQVEIKRTIPKDSVQSKDFK	T	97							
9310	LOC_Os08g39630	AAAAAAHFPDDPTMATQHHHQQQMM	T	92							
9313	LOC_Os08g40830	SPGRRNDSVRFQTPSRNMTAYSGVQ	T	1426							
9316	LOC_Os08g42140	RQRLDAGGRSSATKTSGTAATGSAS	T	16							
9319	LOC_Os08g42750	EPEIRMLLEAADTDGNGTLDCDEFV	T	451							
9322	LOC_Os08g43410	SRGFDCATHVKSTWVPAAKRRERQN	T	128							
9325	LOC_Os08g44150	LTWVLMGMLAGYTSSRLYKMFKSGS	T	289							
9328	LOC_Os08g44540	AKVAPNVKRAAPTDPVERRLLGKVN	T	78							
9331	LOC_Os09g02480	CYSFAHWILLVITPKWSTCHYLNSR	T	78							
9334	LOC_Os09g04890	WPLDEDWYKGSITGYNEATKKHSVK	T	192							
9337	LOC_Os09g07690	PVYSCLPPVYQDTWNSLPMGEEAAQ	T	188							
9340	LOC_Os09g08420	LVMEFCPGGDLHTLRQKQPGKFFPE	T	266							
9343	LOC_Os09g10710	SRWPEDNSFYEATITDYNPETDLYA	T	214							
9346	LOC_Os09g11250	STTIFQRFGFSSTSPELSDKEENQR	T	76							
9349	LOC_Os09g13120	PETPLAWQGTRTTLRLTPFLLHSLR	T	126							
9352	LOC_Os09g13850	QRWLDDVANLVMTARQHLGAGGRSS	T	115							
9355	LOC_Os09g14990	PAKDAECFFCKETGHWKRNCKKYLE	T	176							
9358	LOC_Os09g16380	YRRNKNLIKELSTPHESSSDLSFPT	T	1094							
9361	LOC_Os09g16980	VLVIELLTRKRPTYRTDQGDSLVLH	T	526							
9364	LOC_Os09g17850	YWTQDGGDADDQTVEQQPEDLKTQE	T	419							
9367	LOC_Os09g19750	EVTPFSPPPNSITASLIYYRSPQFV	T	82							
9370	LOC_Os09g20060	IQWPKDLIEIRVTARPTTAPGGRPL	T	79							
9373	LOC_Os09g21000	AAMIASQAMISATFSCIKQAMALGC	T	470							
9376	LOC_Os09g23730	EGLLPPAHPSLLTAHLARMKQTGEL	T	68							
9379	LOC_Os09g24440	CHSKVAAKRFQKTKKGSKPDGSDEN	T	814							
9382	LOC_Os09g26540	PVIAVARNFKSKTLLVRCGEMTRER	T	190							
9385	LOC_Os09g26800	WDQYGIAYGRPSTNIIIHNVTIRSM	T	338							
9388	LOC_Os09g27020	SPETPLAWQGTRTTLRLAPFLLHSL	T	115							
9391	LOC_Os09g29500	RGPGGSLRRARVTSLVVAVVRTTVT	T	440							
9394	LOC_Os09g29750	LRMASAGAPQFGTMNRHNHVPVPEP	T	994							
9397	LOC_Os09g30150	EPSCIQPSCVAPTTCFGPRFFKSKS	T	382							
9400	LOC_Os09g31470	MQSVRMAYTADGTPIFAPVNSAPAP	T	53							
9403	LOC_Os09g32430	HDEVSRSKDGTDTEDATIDTDRSSE	T	151							
9406	LOC_Os09g32650	KPLIKNKLLEEGTAVLYSEPEKKVM	T	511							
9409	LOC_Os09g33870	VDSSHYRRYCSPTQDDDSTVSTHRR	T	245							
9412	LOC_Os09g33980	EKYHFLEAMSTITPAAHRGSIVHSR	T	625							
9415	LOC_Os09g35710	IDSSLQPNPSIPTAPPLVHVTDYDN	T	193							
9418	LOC_Os09g36320	ESCLLKNAKERPTMSEVVDVLRRAV	T	361							
9421	LOC_Os09g37170	DDGEPNPRRRPRTPPPLPRQSHRRE	T	29							
9424	LOC_Os09g37860	KKRNGPGSGKKETHNEKEEPTSEKK	T	635							
9427	LOC_Os09g38620	LAENRHLIVLLTTSLAVLVGCGVAL	T	51							

9430	LOC_Os09g39330	LDKDLFAAATKVTVGDGNTARFWDA	T	869							
9433	LOC_Os09g39490	EFRNKNAARRSETTQKKAEKRRKID	T	135							
9436	LOC_Os10g01320	VYYVSEALHDAKTRYPQIQKLLYAV	T	317							
9439	LOC_Os10g02584	INGIDLDLSRIPTPICSCTGAPQQC	T	250							
9442	LOC_Os10g03830	ATVDGVKVDGLDTNLAVMKELLSPV	T	497							
9445	LOC_Os10g07180	SRSLGKALLQTMTSYLLTAAPITPS	T	202							
9448	LOC_Os10g07480	EKNMSVRSADVATKQHVEDVKKISK	T	125							
9451	LOC_Os10g12174	CYSMTINKSQGQTLQRVGVYLKKPV	T	1240							
9454	LOC_Os10g14120	TTSGSSRARTPRTGNKIPKEWFRIT	T	16							
9457	LOC_Os10g16480	AQFQGVVDCARRTVTLYRGPEQPVV	T	805							
9460	LOC_Os10g20040	DLGFQEPGALIATLDGGACAHVSRR	T	440							
9463	LOC_Os10g20480	YTIPHPPNDEILTLGAARGTFIQWP	T	427							
9466	LOC_Os10g21310	GELNGVSYSDPATVKKYARHSQLGE	T	416							
9469	LOC_Os10g22070	KLLGSTNPSPFVTRVELALALRGLT	T	29							
9472	LOC_Os10g25280	YTTMPISAGSSMTGNENAVVTTQDD	T	76							
9475	LOC_Os10g26670	RSNFTLLAPVKVTIPASGGDDDDDD	T	266							
9478	LOC_Os10g30350	LGPWTTLAEFMATDDFRYLTKSCPG	T	307							
9481	LOC_Os10g32850	PNESHLNNSVSSTADGLLMEAAHQR	T	517							
9484	LOC_Os10g33680	EALYSQWLALPETSKLVNSLIEDAK	T	48							
9487	LOC_Os10g34400	LERRDRFSFSFYTEQYSKRQDVEKV	T	502							
9490	LOC_Os10g35220	RGGDIVKALRDTTKAKIRVADSIPG	T	75							
9493	LOC_Os10g35820	RAAPASTSTPPTTRATTTTPRRPRT	T	48							
9496	LOC_Os10g37230	YNCGHPKNVAALTPDAVSTQTGAFL	T	163							
9499	LOC_Os10g37630	EKYSLVSNFPRVTYGPEKLSQTLEE	T	439							
9502	LOC_Os10g38850	AVDSRFTRAWVDTDTIFIDGVKDPL	T	1679							
9505	LOC_Os10g39620	TVLYLTIWVDVHTYEIIGSYRIEYG	T	620							
9508	LOC_Os10g41120	LPDWLRLALSSATAASSSSSSSSDE	T	126							
9511	LOC_Os10g41790	AHTNAQMNKLLLTMAEWYRYADNRK	T	539							
9514	LOC_Os10g42196	PLSLSLGLPGVVTSNQALEMKQFLP	T	1525							
9517	LOC_Os10g42724	AEPEAEPAMIPVTVEPENSPRTKDG	T	331							
9520	LOC_Os11g01920	LPSELTLRSPRATMYCEADQDQLRR	T	1891							
9523	LOC_Os11g02720	PSRRCAVVRAAHTNPWRHRVSRCSG	T	14							
9526	LOC_Os11g06750	KKYASVVRVIVHTQIRKMKGLKQKK	T	166							
9529	LOC_Os11g07440	ILLRAYTKYTADTSLAESPECQNCM	T	221							
9532	LOC_Os11g08460	RETAEVYLGKTVTNAVITVPVYFNN	T	155							
9535	LOC_Os11g09300	REYVRRFNECRNTIPEITDASVICA	T	305							
9538	LOC_Os11g10060	IHGRLVCWLDAATSKLEKAEQQQQH	T	675							
9541	LOC_Os11g10510	FITHSVPFSEINTAFDLMLKGESLR	T	360							
9544	LOC_Os11g11710	AWFVARDGAKDCTAAVDECKDKVDQ	T	101							
9547	LOC_Os11g13459	VLHLLKDYGSSSTSTPREVLAIDGT	T	190							
9550	LOC_Os11g16370	NMNGSVSGEDQPTTSGVENGHQEPF	T	42							
9553	LOC_Os11g16510	KRYKLDVSVSMGTGCESIDPRLPAF	T	184							
9556	LOC_Os11g17890	DTSSVTCETISVTPMPKTGIVSNFL	T	549							
9559	LOC_Os11g20290	APNRERPTGKWCTVHNTSLHDLADC	T	363							
9562	LOC_Os11g22180	TVGRRIGQALPLTDVIGPLVDHQAA	T	51							
9565	LOC_Os11g28270	FGPNCKFDHPMGTVMYGLATSPTGD	T	384							
9568	LOC_Os11g29000	IVAESATEDAATTTGSSNAIGFPKL	T	566							
9571	LOC_Os11g32900	SSHNVYVSPLRQTKMDALLSPSSRS	T	888							
9574	LOC_Os11g33340	HQGFPLKAHSSPTTRMLISMSSRWL	T	689							
9577	LOC_Os11g34450	KEEGRGNDAHAATIRSYRGKIEAEL	T	91							
9580	LOC_Os11g35450	IAGLIPRMPRNLTTLDLSNNSLSGP	T	565							
9583	LOC_Os11g36060	QPHKVDLSSPDRTIIVQIAKTICMI	T	339							
9586	LOC_Os11g36340	TKIPSQASATAKTETKPLVTKQKTS	T	358							
9589	LOC_Os11g37090	ICEITRVPGELITVACNMHGTRTVQ	T	501							
9592	LOC_Os11g37890	TDRFEMWGDGLQTRSFTFIDECVEG	T	236							
9595	LOC_Os11g38350	RSSATKTSGAATTGSVSSRRRARRA	T	137							
9598	LOC_Os11g40090	KHSKKKREAIDATMAAASPKKKEKK	T	57							
9601	LOC_Os11g40820	LCVDDLVLRRVQTRAGLSKLSPMWE	T	1915							
9604	LOC_Os11g43590	VKGDIPVARSGHTVIRAGPVLILFG	T	211							
9607	LOC_Os11g46240	QVSQSQQERPGQTVYPQASHLGHQQ	T	101							
9610	LOC_Os11g47330	VAGGGASVIYADTVGDLGYASELGN	T	288							
9613	LOC_Os11g47760	AGGVMTVLIPRNTTIPTKKEQVFST	T	424							
9616	LOC_Os12g01922	NKSYTQYIPLPITDLDSWLKTPSIY	T	230							
9619	LOC_Os12g03899	SVIKNNLGVTIITGCFILQNNAVPQ	T	414							
9622	LOC_Os12g05000	TTTMRDGKLWKTTTIKRRPGTGAPS	T	217							
9625	LOC_Os12g05110	KPAVVTRVVDSMTDNLRPTRAEATD	T	320							
9628	LOC_Os12g05930	RLYGQIPHKLMMTSLQFLDLSSNGD	T	76							
9631	LOC_Os12g06640	GKKGAAWQRMKWTDSMVKLLITAVS	T	110							
9634	LOC_Os12g06980	QKQVAGDVGEEITVSVQDGVSVRST	T	202							
9637	LOC_Os12g07260	EKAEEIFDNMFKTWKTLSSKYYNAM	T	443							
9640	LOC_Os12g08230	SGDPSSEGGVQVTCFTEDLHDVILH	T	40							
9643	LOC_Os12g10070	LDISKRLEGSLETLVVCCGSIRDRF	T	497							
9646	LOC_Os12g11600	LLIKVASGMAIPTDISGTYHCRPIP	T	394							
9649	LOC_Os12g13340	KTNAVAPSIGSGTTVGKVEFNEFIN	T	66							
9652	LOC_Os12g15900	NQSNNQINNETWTMAFNGALNSQGA	T	356							

9655	LOC_Os12g18120	DARAPPKSDPGATPIGSISPSSAAP	T	17							
9658	LOC_Os12g18990	GRSSATKASGAATAGSVSSRRRARR	T	23							
9661	LOC_Os12g21710	PPLLLHFPQRRVTLPQSRVSPTAPL	T	25							
9664	LOC_Os12g21940	ELHEGIIVWHIATDIFIAQRKADDQ	T	546							
9667	LOC_Os12g23520	FFFFRADCTISKTRMKFSWIHMFFM	T	677							
9670	LOC_Os12g25710	GGGGKKKEVKKETKLGMAYKKDDNF	T	25							
9673	LOC_Os12g26240	ATCQARWSEIALTFDQTDHPPCVAR	T	595							
9676	LOC_Os12g28260	QYIPRLVRFYPITSELKRTTGVFAE	T	263							
9679	LOC_Os12g31050	TTSLQQIVQKENTREFVPRQQHDEL	T	352							
9682	LOC_Os12g32410	RAFKSGVRDRYTTQELATRRITTTR	T	435							
9685	LOC_Os12g36180	EIWLTVSEIPLFTQPTSAPPPSRTP	T	378							
9688	LOC_Os12g37430	KSTSYEIDNDFVTSRSKRDGNKRSR	T	324							
9691	LOC_Os12g37570	RHPNILLFMGAVTSPQRLCIVTEFL	T	543							
9694	LOC_Os12g37780	SAQQRYSEAEVVTDAALDETTKWEQ	T	514							
9697	LOC_Os12g38440	NVQSPRCLESMRTPDVKKRSVQKQK	T	163							
9700	LOC_Os12g41140	IPVEKNAIAAATTGGGHHKHNAQKQ	T	307							
9703	LOC_Os12g41290	DSSGTSPGDTLSTTYFPRLDGTDYK	T	614							
9706	LOC_Os12g42876	TYFADIPAESYKTLTSLNSVTAYGF	T	254							
9709	LOC_Os12g44340	FQMRSSVSTTTTTLVNHAKGASKSF	T	402							
9712	LOC_Osm1g00440	EFALPRKILESTTATIFCLFSFSSL	T	104							
9715	LOC_Osp1g00600	MFVIPFMTRLGITNSWGGWSISGGT	T	72							
9718	LOC_Osp1g01070	FYCLLFSGKWLRTSEDRGERQAEIS	T	210							
7923	LOC_Os01g01510	ELMAIGPVTNEVTAGQSKDKGKMQP	T	250							
7926	LOC_Os01g02020	KFNSAKLRKLGPTFKKNGSVTAGNS	T	248							
7929	LOC_Os01g03820	EIGIRQSSSGGSTSGIADNKVSQTF	T	320							
7932	LOC_Os01g04330	GREVAAMMNELDTDRDGFVDLGEFA	T	77							
7935	LOC_Os01g06290	RDSRSPKGSPRDTQSPRGSPRDSRS	T	235							
7938	LOC_Os01g07260	SGSGETSSSSKKTKDDGVKISNSSV	T	191							
7941	LOC_Os01g08260	SITISGYPKNQETFTRISGYCEQND	T	92							
7944	LOC_Os01g08970	TSNTQFLGDENRTAAQVLWETIMGV	T	186							
7947	LOC_Os01g09620	PARYRTQPCKDGTACRRRVCFFAHT	T	161							
7950	LOC_Os01g10140	KHLLYGASEFEETPRDLDDVFSEAC	T	509							
7953	LOC_Os01g10820	GIRRNEKIACYVTVRGEKAMQLLES	T	72							
7956	LOC_Os01g11920	LALQANLMNMAGTEGIDKEHDDSQD	T	1174							
7959	LOC_Os01g12360	PAPEPISIPEASTDPSQLPHTAHWV	T	1425							
7962	LOC_Os01g12650	NRNISAGVLGGATAIWILFELLGYH	T	114							
7965	LOC_Os01g13270	KLSITNDRMDFNTRGNAVPNGKLDR	T	20							
7968	LOC_Os01g14690	GPLEALRPKLQPTRQQQQRSRRLIN	T	95							
7971	LOC_Os01g14950	LIPALRTVGNIVTGDDAQTQCIIDH	T	313							
7974	LOC_Os01g15039	GNQGTCLDGFHGTDSRLLRRVESAV	T	199							
7977	LOC_Os01g16020	GYLRRWDDEFRPTFRFLDLLQRVFY	T	367							
7980	LOC_Os01g16400	HRVKLFEPFEAMTIPPGLSAMESLQ	T	754							
7983	LOC_Os01g17320	VFNKMKDLGFPVTVFSCNQLLLLYK	T	277							
7986	LOC_Os01g19150	SGNSMDIGHTIVTTVGGRNGQPKQT	T	57							
7989	LOC_Os01g20940	LFSAADLSEQVVTAELIGKEDIPLT	T	100							
7992	LOC_Os01g23540	TNLSNAFEEAAATAHPEQPPTGDAN	T	230							
7995	LOC_Os01g25040	LRVMLPKPNLLPTNTYEVKKLICPL	T	387							
7998	LOC_Os01g26920	DCPSVPIGRSPRTDVLLKAENVVLE	T	200							
8001	LOC_Os01g27790	LLRHPPVVPDPETPARRWLDDVADL	T	126							
8004	LOC_Os01g28660	DVDQWHRGDLHQTPGEEVQAKISRR	T	381							
8007	LOC_Os01g31580	RYQHASSMGAVETRKGKTMSGTASL	T	343							
8010	LOC_Os01g32870	VYGDNFFKPASYTIEAMCAKSYEDT	T	279							
8013	LOC_Os01g33800	SFSMNGKEGRGDTSVWTDTPLERAQ	T	478							
8016	LOC_Os01g35050	AVCAQTQQTSNPTVWLTEWAPEPRD	T	342							
8019	LOC_Os01g37920	EVGLGAGASAAETAEMAATARTARM	T	38							
8022	LOC_Os01g39780	TYKRDQEKKNAQTAAAAAAVVALSS	T	58							
8025	LOC_Os01g40110	KQANGVEQTPKETVGAPVTESAQVN	T	142							
8028	LOC_Os01g40390	LLRLPHVVHRVLTERRRCHVVPVPE	T	262							
8031	LOC_Os01g41280	RRLYLGVFRFPDTTGHPRGPDVFPH	T	194							
8034	LOC_Os01g41550	VSGLAQGSGEYFTKIGVGTPVTPAL	T	149							
8037	LOC_Os01g42250	YAKKVNLSVADLTFAFDGDKVDAES	T	204							
8040	LOC_Os01g42370	YAFGQVAIEFPYTLVQSIIYGIIVY	T	1282							
8043	LOC_Os01g43050	NFEPEIQPHVQDTDVEQQPACTSQS	T	720							
8046	LOC_Os01g43774	FELAASYTHAPHTVMTLHPMHGAQM	T	633							
8049	LOC_Os01g46580	MRFQDSIDIVLATSFLQEFVEARRT	T	191							
8052	LOC_Os01g48280	LKDKWSPALQIRTVLLSIQALLSAP	T	105							
8055	LOC_Os01g49190	FVEQATEQQILVTGIKVVDLLAPYQ	T	211							
8058	LOC_Os01g49640	CAAAILWGEAPSTACCAGLRAQKRC	T	58							
8061	LOC_Os01g50310	PNGSVEEDEVTVTAAENPAKSYAAV	T	65							
8064	LOC_Os01g51080	VKEVALGDGPSKTTRIGDMPGVPRE	T	776							
8067	LOC_Os01g52390	RDGAGRGNWGTATDEALAQETEEAL	T	191							
8070	LOC_Os01g53370	GAELGIPTYFFLTTCIASVAFMLYL	T	158							
8073	LOC_Os01g53670	PLKSFDGSESFGTFSSNTASNITEL	T	477							
8076	LOC_Os01g54400	LTGAEQPSKNAATAATAAAGNSSAA	T	89							
8079	LOC_Os01g54920	FKGKPTGRRNFSTPEEIAAGTSGRP	T	18							

8082	LOC_Os01g55050	YADNLLRSLEGITNRLSQLEIYCYK	T	92								
8085	LOC_Os01g56130	RWSFVNKDENLLTRLQNINLCFHFE	T	372								
8088	LOC_Os01g56490	EEELPHTNPGLNTTPLRFTKHSNAY	T	500								
8091	LOC_Os01g56910	TMVLLEPDAVARTCTDWLKGCCDEI	T	322								
8094	LOC_Os01g58580	DECIVPSDMNLITDQDFRELVQKVP	T	116								
8097	LOC_Os01g60190	NAVEAVKTLRAETKASDQYLPPFVI	T	251								
8100	LOC_Os01g61370	RKALSKLSIGLGTDSGSGSGSPRVG	T	353								
8103	LOC_Os01g61720	TGGASVRDDASLTSTPALPSYMQST	T	414								
8106	LOC_Os01g62290	KGEGPAIGIDLGTTYSCVGVWQHDR	T	15								
8109	LOC_Os01g62500	FGTTSGVVVLAGTNRPDILDKALLR	T	466								
8112	LOC_Os01g62950	SKKALAAQESASTNGRSMQGTTINV	T	190								
8115	LOC_Os01g63770	SCASNQVAQVLLTLPYSFSQLGMLS	T	74								
8118	LOC_Os01g63930	DPGCLELSMPVSTLVEAFDTASTLS	T	224								
8121	LOC_Os01g64970	SEEIMKIVQEARTMPKSSRTGYWSD	T	303								
8124	LOC_Os01g65560	GTDAEIYREATGTSSADDTGYGDPV	T	149								
8127	LOC_Os01g67126	TNKDITAQIVYATIAGDIVMAAAYS	T	66								
8130	LOC_Os01g67330	AAGAYLYTLFFVTVPALASVYNEKA	T	218								
8133	LOC_Os01g68160	AKQAKRTPIKNDTPKRSASYVCNSC	T	286								
8136	LOC_Os01g68810	KYDRDPREKADRTERRRGEDIERPT	T	1523								
8139	LOC_Os01g69030	DEMSAGNASVLTTVLKLIDKYDLYG	T	497								
8142	LOC_Os01g69260	RQKLASAACAWLTVFVLINDAKARE	T	107								
8145	LOC_Os01g70100	VKDVVVNGITVKTKYCDTCMLYRPP	T	149								
8148	LOC_Os01g71000	KLGPVGDKTHVSTRVMGTYGYCAPE	T	244								
8151	LOC_Os01g71960	AAYAILITLHRETINGKSHMKKQEL	T	129								
8154	LOC_Os01g72320	LRTVRAACGVWHTAAVVEVMVGNSS	T	475								
8157	LOC_Os01g72790	PEQLSKIFELCGTPDELIWPGVTKM	T	259								
8160	LOC_Os01g73950	ARKFPPFADPFPTHIAKYGTFDDAR	T	495								
8163	LOC_Os02g01110	GARKNASELSTVTADSGDETNDCGN	T	261								
8166	LOC_Os02g01960	PRSRAPPSPSPSTSSRAKPRKVAAQ	T	38								
8169	LOC_Os02g02410	AYLGKKINDAVVTVPAYFNDAQRQA	T	175								
8172	LOC_Os02g03820	SNCSNALEEDLRTVEEWSSKYRAEE	T	541								
8175	LOC_Os02g04560	NKLEKERVVPKITIGEVCPFMRNSE	T	504								
8178	LOC_Os02g05330	EFRSGSSRVLITTDLLARGIDVQQV	T	337								
8181	LOC_Os02g05900	DTLSFEQSILKITSMKLGNLWNNPN	T	83								
8184	LOC_Os02g06584	TMTRSDKYRSDVTDFDKTSKGTEAT	T	311								
8187	LOC_Os02g07070	TEDSLPADPTSSTPLHPKRGSELGP	T	387								
8190	LOC_Os02g07630	FFIRMVCPRIHFTRSLLLMHLGPFY	T	331								
8193	LOC_Os02g08010	GKFIPVPRTELKTYISRCLPGKKDN	T	566								
8196	LOC_Os02g08360	VIESFNDLYRSITGRITAEALKERV	T	553								
8199	LOC_Os02g09750	KPPPPPSPSPRPTRALVCRAAAAGE	T	27								
8202	LOC_Os02g10690	KNKSAGETEETVTPPGRPVRLSLDS	T	290								
8205	LOC_Os02g11750	INRRVVRVNEVRTRGAREFGREGFR	T	86								
8208	LOC_Os02g12490	LCLAITIDHCLTTSLSYLSAIFIEP	T	84								
8211	LOC_Os02g12540	RALENVHRIVDSTLEEVIEERRGAA	T	253								
8214	LOC_Os02g13500	EGNTQGEVIVRGTNREANDSDDEEP	T	241								
8217	LOC_Os02g14059	EHIDLGIKYDPSTGIYGMDFYVVLE	T	122								
8220	LOC_Os02g15060	VECIDMWLHSHRTCPMCRCDLSPPR	T	142								
8223	LOC_Os02g15910	ITPGPCFPMLCLTATRKSLQYLLAS	T	80								
8226	LOC_Os02g17770	GDAGSLPPAPAATGEWVPIGGDVIR	T	34								
8229	LOC_Os02g17970	GTTAVTVLVRGKTIYIANTGDSRAV	T	218								
8232	LOC_Os02g18550	DNYMLRLFCIGFTKRRPNQVKRTCY	T	143								
8235	LOC_Os02g19860	ASVDLGCSDEILTIIAMIQTGNIFY	T	1029								
8238	LOC_Os02g21350	KAVELVDIGRLATGDAARALVERLL	T	106								
8241	LOC_Os02g23900	IKLILKIPRGHETLHNLVDEALLLK	T	17								
8244	LOC_Os02g24080	LEGYAAYKAEETTQRVQGCVRIAEE	T	136								
8247	LOC_Os02g27740	ENKFGSNDSDFGTQSGRSFRHDPSF	T	240								
8250	LOC_Os02g29400	RSSVAAHGREAATMAKKLLRSTGKA	T	35								
8253	LOC_Os02g30050	KSLLPLDLRPKKTRAIRRRLTKHQL	T	88								
8256	LOC_Os02g32350	DNFAREAKEFLRTRLIGKQVTVEME	T	444								
8259	LOC_Os02g33080	FACTDVESENVDTAERLIREAHKKG	T	30								
8262	LOC_Os02g34270	PAPSLVVPPLAGTGGGRRVDVAREE	T	48								
8265	LOC_Os02g34590	CSKEGLKEGERLTDANFNDPHTRTN	T	150								
8268	LOC_Os02g35910	TVTFVIIDGLFITVASVGDSRCVLE	T	153								
8271	LOC_Os02g37870	GPSVKFLVNAVHTMEELKLTGNHLK	T	157								
8274	LOC_Os02g38250	QAEAASNLQNYLTDKVYLEEPEGQY	T	588								
8277	LOC_Os02g38920	WKHHEVKVKDSKTLIFGTKEVAVFG	T	68								
8280	LOC_Os02g39420	SRASLVAWMGRLTHRYELAAGTLHR	T	206								
8283	LOC_Os02g39920	EQAPSESMISAITPAMKALIKKELL	T	55								
8286	LOC_Os02g41800	GSGVIQNSPTDNTSSERLQWFRENS	T	574								
8289	LOC_Os02g42560	ASKTTATRTASATAESLNTKARSRR	T	357								
8292	LOC_Os02g44050	AAVRHSAATPSSTPSTREDLRGGPD	T	163								
8295	LOC_Os02g44654	AHVDLQDLLLRLTFDNICGLAFGKD	T	183								
8298	LOC_Os02g44820	EAELLLERSRAITLNGRDKRGRALV	T	26								
8301	LOC_Os02g46090	GRIDYGEFVAMMTKGNMGVGRRTMR	T	529								
8304	LOC_Os02g47140	MRPRSMAKEMAGTVKEILGTCVSVG	T	129								

8307	LOC_Os02g47890	NGDAQPSQDKDITEEQVKGNKEVGS	T	171								
8310	LOC_Os02g48180	HLSSVDGSDLARTISSNASLAEQVI	T	642								
8313	LOC_Os02g48640	QTEALLTKISGETASATVNSGSLAD	T	271								
8316	LOC_Os02g49070	RDMLTSDPYVVLTLGEQKAQTTVKP	T	204								
8319	LOC_Os02g49310	KESVDVSSAKCPTEVSEEMVSEGIA	T	82								
8322	LOC_Os02g49992	LITPHSLFRPHFTTLTQRSPSILSK	T	770								
8325	LOC_Os02g50620	SDSIITAYRDHCTYLARGGDLVSAF	T	122								
8328	LOC_Os02g50850	KIDAKLNIYPSTTSELRGGDKQSHG	T	889								
8331	LOC_Os02g50970	SFGEVYRGEWHGTEVAVKKFLQQDI	T	860								
8334	LOC_Os02g51860	IGVQHDWCEAFPTYPRTYDMVHADG	T	570								
8337	LOC_Os02g52520	LSLQGTGAGVTLTSPSGDVLRYLVR	T	1379								
8340	LOC_Os02g53550	DEEKTAFITPIGTYCYTTMPFELKN	T	900								
8343	LOC_Os02g53700	EVSGSYNPEVLTTQKRQWSRFQLKS	T	88								
8346	LOC_Os02g54254	ASHPLLQQTTRPTYRDFLVELFNAC	T	873								
8349	LOC_Os02g54910	FKTSVLAVRLSRTRLVVVLQDRTFI	T	108								
8352	LOC_Os02g56480	LPGEDLDALVSVTNDEDLEHLVLEY	T	113								
8355	LOC_Os02g56960	VNPKGEMKGSAITGPIGKECADLWP	T	118								
8358	LOC_Os02g57540	HSYKHSGLANKKTVTIQPSGGKDAA	T	56								
8361	LOC_Os02g57820	GSYLVIEEGGSRTRSGGLCIALCGP	T	287								
8364	LOC_Os02g58220	RNTDVSFTIDDGTGRLDFIRWVNDG	T	95								
8367	LOC_Os02g58440	FDKPKDSAHNGKTAQKQNLTNRAGH	T	192								
8370	LOC_Os03g01420	DDGEIIDRIEVFTGVEVLPVGHDGD	T	355								
8373	LOC_Os03g01850	FMDSCPEFAKNPTLIKSYLYQILRG	T	218								
8376	LOC_Os03g02350	RELLAAAPTHEATCRARWSEVALTF	T	584								
8379	LOC_Os03g03470	NVHSVLPKTGVWTNSYWDEFVSLPA	T	121								
8382	LOC_Os03g04330	GPSYHHILTTLGTKMSSRITSEQPN	T	100								
8385	LOC_Os03g04920	ILYKNVGIAMVSTLVATVLSIAASV	T	452								
8388	LOC_Os03g05260	YGQVPAEMADEGTEVRKLLIQEQVE	T	191								
8391	LOC_Os03g05790	APIQICDSGVDLTVYAFHDTSLNAP	T	26								
8394	LOC_Os03g06220	DESQIQAVTSGGTVYESAAAFEDLK	T	94								
8397	LOC_Os03g06410	SYGEVYRADWNGTEVAVKKFLDQDF	T	757								
8400	LOC_Os03g08270	GVQTGPAWLEADTAANMFLESKDEA	T	357								
8403	LOC_Os03g08550	LGGGQQIQYNLPTNKLERLNLAGNQ	T	126								
8406	LOC_Os03g09870	DDIVNAGIAGCCTGLALSFPGTPQA	T	119								
8409	LOC_Os03g10400	LYKSAPHLNFKHTVFGMVVGGLTTL	T	336								
8412	LOC_Os03g10710	MYHNSLRIKGSNTFIIYEMDQLEAG	T	80								
8415	LOC_Os03g10990	LELQQAEKGKKPTTKNKSNGKSPGS	T	533								
8418	LOC_Os03g11290	KLAEMPQLEESRTMEKVTTVKQSSI	T	197								
8421	LOC_Os03g11550	PPSGLFRTSSLPTVAAAEAAKKQGV	T	86								
8424	LOC_Os03g11790	NGFSSTKSLVELTCHILQNTTSLEC	T	435								
8427	LOC_Os03g11910	GIIKQCLADAKLTPEDINDVILVGG	T	363								
8430	LOC_Os03g12250	SALLHGNRASGRTPLDWETRSAIAL	T	477								
8433	LOC_Os03g12910	NPSPILFYPISSTEVRCLVDVPGQK	T	361								
8436	LOC_Os03g14580	KTNSAVKDSGKTTAQKKRRASSDTD	T	286								
8439	LOC_Os03g15010	HNYEGLRYRSTFTCTHGGKARADAS	T	155								
8442	LOC_Os03g15050	GQFPCNRYTHYMTSSTSVDINLARR	T	299								
8445	LOC_Os03g16000	PKTAATHEHSRSTSEDHIFTNTSEP	T	287								
8448	LOC_Os03g16910	IQRVKVSWKNTLTNGIVKVSCTSVG	T	444								
8451	LOC_Os03g17180	AAGWIEVSCWILTGERQTAVIRSKY	T	163								
8454	LOC_Os03g17310	FGSTEDISSKSFTGKEFMSLSDKKK	T	686								
8457	LOC_Os03g19250	LLAALSIRGFRVTHTYGLSETYGPS	T	345								
8460	LOC_Os03g19290	VAGALTGAAVALTSDRASHERVVQC	T	149								
8463	LOC_Os03g19930	MLSRTHGQPASPTTLGKEMANFAAR	T	253								
8466	LOC_Os03g21080	LEDQWKRRTVLQTEGSFPALVNRLL	T	812								
8469	LOC_Os03g21510	FAGQIPASLQNLTQLSTLNLSKNSL	T	162								
8472	LOC_Os03g22350	DLRDLNDVSDYVTKAGYGSESEVDD	T	257								
8475	LOC_Os03g22890	GYPNVGKSSVINTLRSKSVCKVAPI	T	335								
8478	LOC_Os03g24930	AARAVAHLAVQCTSPQPRDRPRMAA	T	373								
8481	LOC_Os03g25430	AIFEIARGIWRPTSDKHGRDADSDD	T	183								
8484	LOC_Os03g25600	RYTLDSSVLDSETRSGSFRQEVVVV	T	84								
8487	LOC_Os03g25970	GNEESDEGLAQVTVRLLVPSDQIGC	T	139								
8490	LOC_Os03g27460	RGGSSRGRVKKTTARKKVGAGLKRR	T	296								
8493	LOC_Os03g27840	CRVHRDDALYQATEDQQGLIPWNGK	T	60								
8496	LOC_Os03g28980	EFDAVTPAAISETKVAVATIRRKSQ	T	297								
8499	LOC_Os03g30460	ALTQLLLYYTRITECVKKINGGSAL	T	669								
8502	LOC_Os03g30990	KSPTRLKEVHKLTGCMAALSRFVAR	T	935								
8505	LOC_Os03g33012	NIESTEVSHGFQTTALTEDKPADDG	T	190								
8508	LOC_Os03g36610	RLQSGIRKDKIYTDGTVRYSCFTSS	T	30								
8511	LOC_Os03g39170	MYVPRNQDGGAITDALVGEIKAEAK	T	409								
8514	LOC_Os03g39590	GFEYLYVTIDKFTKWPEAYPVVKID	T	1177								
8517	LOC_Os03g42770	LTMDALSKDRLLTVARDRTMHLWKI	T	319								
8520	LOC_Os03g42840	LSDEVIESIIDKTFEEADTKHDGKI	T	170								
8523	LOC_Os03g43760	HRDVKTENMLLDTQRNLKIADFGVA	T	231								
8526	LOC_Os03g44390	IEELPAVLWANRTTLSRATGETPFF	T	1737								
8529	LOC_Os03g44440	DHQKGFLEWRTITRQYINETCEPRD	T	318								

8532	LOC_Os03g45344	SRAVSAKIRAGATYDLQRNKSNLES	T	48							
8535	LOC_Os03g46570	CAAAADAAESGVTFGGGQEAAAPRK	T	97							
8538	LOC_Os03g47760	KRPGEGAGDGFVTSIRGLFNSQRRK	T	976							
8541	LOC_Os03g48350	KSQVLADFVADWTMPDNKSDNQIDN	T	69							
8544	LOC_Os03g49150	GKAYLFNKVVNVTSGVKEDRMMITG	T	51							
8547	LOC_Os03g49580	LRMEKRHNYVRKTAELATQFFINPA	T	204							
8550	LOC_Os03g50150	TWLELSEAFPKSTNGSKILLTTRSK	T	311							
8553	LOC_Os03g50290	EKVAKTTDVGELTVEERNLLSVAYK	T	44							
8556	LOC_Os03g50480	GDGSRLVFRLSGTGSVGATIRVYIE	T	527							
8559	LOC_Os03g51030	DAIHSLQLILRGTLNDDIKPTRAAS	T	589							
8562	LOC_Os03g51230	RLPMDNKLLLTGTPLQNNLAELWSL	T	445							
8565	LOC_Os03g51520	ADGRGWNSGEVMTGRPIQVETGYGE	T	521							
8568	LOC_Os03g52980	RRNKDAHEERRSTHWLTTQKERCMY	T	528							
8571	LOC_Os03g53270	SLKQHYGLSKTATEVTILIEAYRTL	T	102							
8574	LOC_Os03g53960	QKAKESGQLLSNTKVVHKTNRPMKI	T	439							
8577	LOC_Os03g54890	MKYLRFLRKRMNTKPSHGPIHFRSP	T	69							
8580	LOC_Os03g55330	LDAGAPWNALSPTGISAGDLASDSA	T	78							
8583	LOC_Os03g56460	EGKPVLGFNSSTTSLLTRYLAVEPS	T	544							
8586	LOC_Os03g56900	EELFVNIAVAVGTQVNKFLSFLQDV	T	145							
8589	LOC_Os03g57100	PAVYFLYDLSPITVTIKEERRNFLH	T	298							
8592	LOC_Os03g57310	EQLVVARKHQKSTRKWTCIAIILLL	T	281							
8595	LOC_Os03g57480	FGLKNAGPTFQRTTRISLGSQIGRN	T	957							
8598	LOC_Os03g58250	PQLSAAAAAAATTSAVVDPVEYNAM	T	113							
8601	LOC_Os03g59020	EIIIGYTKAINKTVEILEDLVEKGS	T	140							
8604	LOC_Os03g59410	ATCRARWSEVALTFDQTDHPPCVAR	T	595							
8607	LOC_Os03g59680	KIAKVVPEQSIATDHAGEYDESLED	T	249							
8610	LOC_Os03g61010	RDGKSYSTNLAFTPPEYLKTGRVIP	T	216							
8613	LOC_Os03g61220	EKEIKESAPKLSTVCVYGGVSYNVQ	T	209							
8616	LOC_Os03g62340	HLNLALLGVEDDTLSHCSTEQTMDT	T	913							
8619	LOC_Os03g62500	QLVEIIKVLGTPTREEIKCMNPNYT	T	307							
8622	LOC_Os03g63140	RSNREGDLRQHETTVRKPYAESPNH	T	85							
8625	LOC_Os03g63920	LISKEEAPDSDVTEEMARNFCLKPE	T	672							
8628	LOC_Os04g01290	AAAGKAADCIIPTFKNIDSFISDWG	T	162							
8631	LOC_Os04g02500	LETEGLEQASVDTQLTSSNVGFRLL	T	99							
8634	LOC_Os04g04254	KVPIHIFFTMPWTPTSEFPHPLSRV	T	303							
8637	LOC_Os04g07060	GKDVIRGRVNHVTAEDVLTTPDVIV	T	644							
8640	LOC_Os04g09700	RTCEGCQFFARQTHLPAQELQTIPL	T	442							
8643	LOC_Os04g10280	RPPSKLRDVQCVTGCMAALSRFISR	T	1062							
8646	LOC_Os04g13340	SVHSWEDLCQQFTMNFQGTYPRPGE	T	307							
8649	LOC_Os04g14580	PYLGEEDELESRTTSIEEGEDDADI	T	1242							
8652	LOC_Os04g16180	VKKAFRQGFFWPTALKDACDMVQRC	T	224							
8655	LOC_Os04g17640	DESDDEVGLTGQTGRQADQTTDTPP	T	637							
8658	LOC_Os04g21220	LDWLHAVGKKLDTVQCSDEEKVVFA	T	412							
8661	LOC_Os04g23010	SKATRSWCPIHKTRKHTLQACWVFL	T	77							
8664	LOC_Os04g23400	TIYTRRRQTGKHTRLPAQALQTIPL	T	451							
8667	LOC_Os04g25750	KRVRVVEKAAEATTQDSDSAHPDSD	T	245							
8670	LOC_Os04g27520	AGGRSSATKTSGTATTGSASSRRRA	T	126							
8673	LOC_Os04g28090	EMLSEARARLANTRGKKAKRKAREK	T	161							
8676	LOC_Os04g28420	KGHDGDEPFEERTDEEQVIEGLDRT	T	333							
8679	LOC_Os04g29180	VTTPAQHIRTLNTILRETPYDPVLN	T	169							
8682	LOC_Os04g30780	SGGARSFLQAVSTVTEEAPSPLRVV	T	23							
8685	LOC_Os04g31190	GRDFLPRGPDICTRRPLVLQLVRHS	T	79							
8688	LOC_Os04g31500	HEGECGAHSASRTLVGKAFRQGFYW	T	1688							
8691	LOC_Os04g32950	ADDFEPSLIPSKTIPDPDDKKPEDW	T	218							
8694	LOC_Os04g34450	VKENFDCFVSCKTTIDDIESKLRQI	T	282							
8697	LOC_Os04g35930	VERCGGGGAGYLTSDDGGVVVISAP	T	239							
8700	LOC_Os04g36890	VDAKQKKNKNKNTSEAEAGAHQNTD	T	334							
8703	LOC_Os04g38090	HQLRIKEEDIPNTTFTTRYGLFECT	T	415							
8706	LOC_Os04g38870	IMQLLRDNLTLWTSDISEDTAEEIR	T	239							
8709	LOC_Os04g39400	YPLGQILHNREGTGRVVKWAIELSE	T	1376							
8712	LOC_Os04g39980	FKYDDYRRLRLSTGERAGEALARLA	T	287							
8715	LOC_Os04g40940	LYNRGVYPEESFTKVKKYGLTMLLT	T	44							
8718	LOC_Os04g41820	TSENSDLKIRVQTMEQQVRLQDALN	T	337							
8721	LOC_Os04g42600	RNVAQQHNANTPTSQLAALSLGDAI	T	645							
8724	LOC_Os04g43300	EAIVNIHRSLSSTVSSMVTQQEAQA	T	89							
8727	LOC_Os04g44740	AASPDMAYLLNGTPAEAWLGAVQRG	T	249							
8730	LOC_Os04g45190	VVVTEGTTTKDVTGEMFNKGSGNVV	T	106							
8733	LOC_Os04g46500	NFQGTYPRPVRNTIPCIPAHAVIYA	T	221							
8736	LOC_Os04g47250	AANLRRTRGTYQTCIFAVPGLARRG	T	66							
8739	LOC_Os04g47700	KKKGIFGMIMKDTKASKGKQSDANG	T	571							
8742	LOC_Os04g48290	ATVASMGILIQTTSLIYIFPSSLSF	T	352							
8745	LOC_Os04g49060	AEDDDEKVAALLTQLHLENSGAGEV	T	302							
8748	LOC_Os04g51080	SHAAAAGRATARTTGSHARGSNGFA	T	21							
8751	LOC_Os04g51580	AWLPKLVKYSTFTARMTPGRTRVHE	T	299							
8754	LOC_Os04g52180	KSLRSKREKRLRTLRREIAQPFYDK	T	15							

8757	LOC_Os04g52540	KAIKTKDYSPTITVIVAKKRHHTRL	T	874							
8760	LOC_Os04g52820	TRVYDDPEEEEGTTLDEAGNFNDNL	T	42							
8763	LOC_Os04g54564	DATTTTMAAPTATDGGEPKPAALVV	T	107							
8766	LOC_Os04g54820	IYVLVCPKPKCGTNPQSWKVLRVQK	T	87							
8769	LOC_Os04g55220	QLAATIASFSKMTIEDSRKLLPADL	T	46							
8772	LOC_Os04g55500	NMAKPEHAIQPCTTSCTDKVENTKS	T	216							
8775	LOC_Os04g56620	DAALSSDADIILTSGGVSMGDRDLV	T	43							
8778	LOC_Os04g57300	KLDGGTGRFGCQTELFPIDHGLCLP	T	326							
8781	LOC_Os04g58110	LVPLLAEGSAKATDSESTEVILDAA	T	465							
8784	LOC_Os04g58750	RDGKSYSTNLAFTPPEYMRTGRITP	T	85							
8787	LOC_Os04g59100	RSYIQRFCQVRNTIPCIPAHAVIYA	T	234							
8790	LOC_Os04g59400	ETEEMLLNQGQSTSVQVLENYNMNG	T	397							
8793	LOC_Os04g59570	KRGHPPPPPPRRTRTRGPPARPQQS	T	70							
8796	LOC_Os05g01040	EWARPLLTQAIETGNLEELVDPRLE	T	293							
8799	LOC_Os05g01590	KWKNFDEDDCSDTPYGNFGGKRSFT	T	105							
8802	LOC_Os05g02060	VAIDMGNPFLNRTVDGFLKIGAVGA	T	29							
8805	LOC_Os05g02680	AVRKAMATPTEDTLSAKQPDGEESR	T	823							
8808	LOC_Os05g03720	GFEYLYVAVDKFTKWPEAYPVIKID	T	1354							
8811	LOC_Os05g04950	EENFLHLKAENDTQRYKSQIRALEQ	T	738							
8814	LOC_Os05g05950	LVLGKIGVGKSATINSIFGEEKSKT	T	684							
8817	LOC_Os05g07120	EVTVQCYAGGEHTAAARTYVRATVS	T	148							
8820	LOC_Os05g07260	GKILKPLLNECVTPVTCYWSLALLL	T	140							
8823	LOC_Os05g09540	EWLPVQLYLPPATDDDGAVVVLPLC	T	153							
8826	LOC_Os05g11370	TLAINSKYGTTYTVDQVQHHYRRHK	T	75							
8829	LOC_Os05g11780	LSAGLLRQATYTTARLGSFRVLTNK	T	89							
8832	LOC_Os05g12290	SLLSKKLQELGFTPSKADTSLFFFN	T	806							
8835	LOC_Os05g13520	ETLRQLAVDGGRTIVSSVHQPSSEV	T	234							
8838	LOC_Os05g15190	LDSLQVVERSLTTIAPTLEIEPIDP	T	292							
8841	LOC_Os05g16640	KPSGSVEVTTEATTSGQVGAKVEED	T	244							
8844	LOC_Os05g17940	ATGAEQVVADIETTDDWRTLLNKFL	T	124							
8847	LOC_Os05g20100	ARGDLVVCPEGTTCREPYLLRFSPL	T	413							
8850	LOC_Os05g22390	KYSKCWNPIRPPTTLLTSNGHRICI	T	1468							
8853	LOC_Os05g22580	KKWEDWKKDWFYTALPDHPRLRLPT	T	158							
8856	LOC_Os05g22990	TTSETTADVRMLTPLSVKLAALETL	T	532							
8859	LOC_Os05g23610	NKPVRQKITEPKTPYHPMVDDDGSL	T	31							
8862	LOC_Os05g25750	ELTPTDQPFHGITPQSSSKPLGKIT	T	663							
8865	LOC_Os05g28190	EDNSKASDIEDKTAKEGDAEEEDGA	T	193							
8868	LOC_Os05g28430	HWILLVITPKWSTCHYLNSRIDKNA	T	701							
8871	LOC_Os05g30530	ILILRNRLKYALTYREVISILMQRH	T	57							
8874	LOC_Os05g31000	RKAMVKLGMKSITGVSRVTVKKSKN	T	82							
8877	LOC_Os05g32360	LTGLADRPGMRFTVPQIKCYMKQLL	T	137							
8880	LOC_Os05g33030	QEVAGLIEAPIYTIDGVWEKLKVGA	T	351							
8883	LOC_Os05g33940	RVFDDGTVDRTWTGPPEVLPLMQPV	T	50							
8886	LOC_Os05g34780	TGGDSGAHARRRTAALRHERRAPTG	T	503							
8889	LOC_Os05g36350	YAAARRWSDVASTRKAIRSRSMRKT	T	477							
8892	LOC_Os05g37720	WGGGALWRPAETTRVRRCATAADEP	T	83							
8895	LOC_Os05g38830	GNMMDTSGSGVMTERVPSFSRKKFK	T	120							
8898	LOC_Os05g39520	WVMNVVPVGQPDTLPVIFNRGLIGV	T	547							
8901	LOC_Os05g39780	NVLNTPAWGLHITPRAPEQGHTQHT	T	972							
8904	LOC_Os05g40060	ESSSADTKRKPATAQPSTRRRRRAT	T	93							
8907	LOC_Os05g40420	DADQYNCIHVAETPTMDSLKKGAPE	T	46							
8910	LOC_Os05g41172	GILPTFGSTTAFTTTANGVSYTSYT	T	72							
8913	LOC_Os05g41480	AVFLLVGKTSALTMNVAGVVKDWLL	T	269							
8916	LOC_Os05g41670	GFRSFLKETLKETVRDAFESRGVHI	T	190							
8919	LOC_Os05g42320	HQIRMAREDEEKTAFITPIGTYCYT	T	869							
8922	LOC_Os05g43730	GLKNAGPTFQRTTRISLGSQIGRNV	T	882							
8925	LOC_Os05g43970	RSFSSEEQIAAGTSAGRPKNFKKKQ	T	26							
8928	LOC_Os05g44380	GALPRPYDPKANTNVIRDANRRATG	T	321							
8931	LOC_Os05g45740	ERCQELSKQLFYTRLASLPGRYEAF	T	60							
8934	LOC_Os05g46560	DGHQDLKELDVSTNMLQRVGARCFA	T	450							
8937	LOC_Os05g47446	EERLISLLEQINTHTSKQTKVTIQR	T	107							
8940	LOC_Os05g47980	LGIYPAVDPLDSTSRMLSPHVLGED	T	427							
8943	LOC_Os05g48020	LQRLALKKVKGLTKEELATRSDLVA	T	100							
8946	LOC_Os05g48980	SALESMNVENAFTEVLTQIYHVVSK	T	167							
8949	LOC_Os05g50530	TEDGPMFLKAIYTEGEIKRKEYIAE	T	311							
8952	LOC_Os05g51690	QPPPDAASCIYTTALPLIADPPDIL	T	32							
8955	LOC_Os06g01560	ARGGQIAMVVSPTVCNVKLGRVLID	T	572							
8958	LOC_Os06g02130	GKEGEKPDRVVSTNLTFNGKLQPNA	T	342							
8961	LOC_Os06g03310	SSGGLSPWSPPRTSEPFMEKLEVQL	T	1078							
8964	LOC_Os06g03780	HLDNLTEEMEEKTENIVYEDFKFLT	T	374							
8967	LOC_Os06g04660	HIDSHDFVRPFCTVSFLSECNILFG	T	336							
8970	LOC_Os06g05180	SGSVCIWDYQSQTMVKSFEVSELPV	T	48							
8973	LOC_Os06g05530	CFVEFSDAKCAITAMEALQEYRFDE	T	187							
8976	LOC_Os06g05850	DELSRLASSRAQTPLGAFEERLAQP	T	1446							
8979	LOC_Os06g06530	GPKLTTPTTSVATPGDPVVDKYISM	T	156							

8982	LOC_Os06g08023	FFQQHLGEKQKYTNLIDGKHFQLEG	T	106							
8985	LOC_Os06g08740	DPQPRKHTLWQATSDFTGGQASMNR	T	232							
8988	LOC_Os06g08850	WWGLQNLSCYGQTLTVSTYIGETLY	T	358							
8991	LOC_Os06g11610	ATAAAPGTNGLATAAARRGGWWVAK	T	140							
8994	LOC_Os06g12030	SSDPMEPTDSSQTEEDILAEDKSEE	T	88							
8997	LOC_Os06g13060	SQRKEKKVKDQPTGGCRSADEDSNE	T	398							
9000	LOC_Os06g14080	KLQDDNRALDRLTKQKEAALLDAER	T	177							
9003	LOC_Os06g14510	VQGFNSSTASLLTRYLAVEPSTPYN	T	548							
9006	LOC_Os06g16160	GKEAARMLLMDATEAMAQRPDGHPS	T	772							
9009	LOC_Os06g17290	LAVQLDRGDNAHTVKRKLQLALNVP	T	114							
9012	LOC_Os06g19090	FGDHYILFLVYPTDQTVVVLDPADY	T	459							
9015	LOC_Os06g20090	ALRTLKGVGQSGTQGADKSSASVTA	T	1000							
9018	LOC_Os06g21350	CYYKANVDGFSATDFHCHWPHGHRR	T	30							
9021	LOC_Os06g22070	FEIIDNPGDQSITLKREIAGETIKA	T	126							
9024	LOC_Os06g23530	ARTLHTVIHAIPTHADDAPAPAPQN	T	90							
9027	LOC_Os06g24810	APEPNGGLRPLTTGVGPLPACPTTP	T	1244							
9030	LOC_Os06g26190	NLETSTLEDENDTFEHESFSFKFPQ	T	93							
9033	LOC_Os06g27830	CIAHGGGKRCAVTGCPNAACGSSQG	T	194							
9036	LOC_Os06g28100	RSSATKASGAATTGSASSRRRARRA	T	137							
9039	LOC_Os06g29400	APQHTSVAGATETEAKGAATDPEIE	T	702							
9042	LOC_Os06g29430	EDDANLASSSKNTMISKHHNTVDQK	T	538							
9045	LOC_Os06g30750	LTLIYVAVLMLHTVPILYDKYQDKV	T	217							
9048	LOC_Os06g30901	SLSTDILSMVQSTFKGILLESEIST	T	825							
9051	LOC_Os06g33520	DVLAKAKTGTGKTVAFLLPAIEVVS	T	506							
9054	LOC_Os06g34440	EVKYNEDSDGDDTDCHGNGDDGFVS	T	372							
9057	LOC_Os06g35530	HGPTIMGGNDPVTGHIISTTIGGKN	T	50							
9060	LOC_Os06g36360	DQMVEEANAVLFTFGSYRLGVHGPG	T	99							
9063	LOC_Os06g36700	PKPKTKHKVDIDTVEKFQMLREQEQ	T	266							
9066	LOC_Os06g36920	LLSRVPATADWRTARANERLRARVG	T	283							
9069	LOC_Os06g38490	LPRVSYTELSMGTNGFSSSNLIGEG	T	654							
9072	LOC_Os06g39344	STAASASKTLAPTLTFRRFAAMAAA	T	46							
9075	LOC_Os06g40640	NAMNKLEVLKPWTLTFSFGRALQQS	T	321							
9078	LOC_Os06g40950	TSPFGKMTFETATKFIVEAASHGES	T	304							
9081	LOC_Os06g41730	ASGSGRARRESATRAMQGWHSQRLL	T	293							
9084	LOC_Os06g42200	AAHEAACAEEEFTLRLREDALTERE	T	784							
9087	LOC_Os06g43270	RSRKLKNTCSPTTMEVPDGTIVGME	T	367							
9090	LOC_Os06g43470	VQRDGSLQFSLTTDNIKAAIAVSYN	T	301							
9093	LOC_Os06g44130	ISIERRRGVCGTTACSKRSPPRSPR	T	190							
9096	LOC_Os06g44890	NPVTDIDVTKRETYLSAAEFREREG	T	980							
9099	LOC_Os06g45120	SPRPVSSKLAADTPLLTGQRVLDAL	T	224							
9102	LOC_Os06g45870	LSRWLKSSPAGNTNQMYDENNLIEE	T	321							
9105	LOC_Os06g46400	LPNQNSIDNTTTTTAQPAVGQQAQT	T	197							
9108	LOC_Os06g47320	AIMIVRRALKNSTVVPGGGAIDMEI	T	407							
9111	LOC_Os06g47340	WMGAVSVGILAHTGTADQPRLSEED	T	327							
9114	LOC_Os06g48534	TPMMRRGPDGPRTLCNACGLMWANK	T	231							
9117	LOC_Os06g48750	TVSATHGDMDQNTRDIIMREFRSGS	T	318							
9120	LOC_Os06g49090	ELHAHSQGSTSATIKVTSGQAFVNV	T	763							
9123	LOC_Os06g49740	IGKLLDSNGLDMTKSYMLDTQESAE	T	191							
9126	LOC_Os06g49830	SRGFSCATHVKSTWVPAAKRRERQQ	T	167							
9129	LOC_Os06g50539	TTTIPTSTSADPTSYLEWVRRSREP	T	302							
9132	LOC_Os06g50670	TTKTITLGGAKGTVGWVDLWRGILL	T	216							
9135	LOC_Os06g51420	SLPKFEDPISAETTEPKQVENPFGN	T	118							
9138	LOC_Os07g01920	DIILSRTQRQTPTVVHKGYAISRIR	T	32							
9141	LOC_Os07g03180	CMEKGACYKKRLTCPEKCFKSFSFK	T	99							
9144	LOC_Os07g04190	LSRNQLDGEIPATIAAMQSLTAVDF	T	571							
9147	LOC_Os07g05610	SSTSTGLSGWQLTVDALETIQSLAQ	T	896							
9150	LOC_Os07g06286	RQLIDNFARKNFTVEELVVLSGAHS	T	116							
9153	LOC_Os07g08710	LAKVKNSFKLSSTRPAAPAAADAKP	T	146							
9156	LOC_Os07g09060	FGAAGQRCMALSTAVFVGGSEPWED	T	323							
9159	LOC_Os07g10110	FGDESCYASELVTWATKQVMSFALL	T	331							
9162	LOC_Os07g10460	DIDGATAFAASGTPADCASLGISGK	T	85							
9165	LOC_Os07g10890	PPEPPAAAAVAGTAVATAAAAVPTH	T	23							
9168	LOC_Os07g12170	KIRPLWRHYFQNTQGLIFVVDSNDR	T	208							
9171	LOC_Os07g13340	DESFPRPRRIPPTLAQFMPRNWGQL	T	1296							
9174	LOC_Os07g18300	LKKLARVKGMASTSEAYRAETRSNY	T	514							
9177	LOC_Os07g20150	DSDAQELGLFHETEEEFEEQSVRAD	T	441							
9180	LOC_Os07g22610	GRCGADVGPTAITTTTPTFQRGSEW	T	1283							
9183	LOC_Os07g22750	KMFTETIFPRFGTPRMVISDGGSHF	T	501							
9186	LOC_Os07g25550	LWKVTSIGRKSDTSLSKGKFKEMFD	T	238							
9189	LOC_Os07g28800	WHPDRHNGSTKATAEEKFKHCSAAY	T	247							
9192	LOC_Os07g31840	SLLLGWSSEDEKTFAEALVFVQDSS	T	150							
9195	LOC_Os07g32480	TPRPDGSYMYQPTSPFKRYWNVELW	T	286							
9198	LOC_Os07g36200	PRNADKKDYRRDTDRSDVYRPTRRE	T	431							
9201	LOC_Os07g37650	PSVAPPPKVDYATDLFNMLSMDGTT	T	227							
9204	LOC_Os07g38120	EFGVTYLCTERETGDAYACKSISKK	T	97							

9207	LOC_Os07g38970	AMEAELDLVVCITEGIPQHDMVKVK	T	131							
9210	LOC_Os07g39280	SATSFQVLGHMKTVCVLILGWILFD	T	263							
9213	LOC_Os07g41190	LVNAAVSKIGPLTGREEKSLEHFEI	T	340							
9216	LOC_Os07g42300	VTADGQGVKVESTAVPSASTPDVAD	T	82							
9219	LOC_Os07g42410	GVFAYITHTAGSTKSFLRKGNEAHP	T	1049							
9222	LOC_Os07g42950	TAGRVRLLLHRGTPCFRGYGRRDGE	T	81							
9225	LOC_Os07g44200	AIFEIARNIWRPTNKHGRDGDSDDE	T	184							
9228	LOC_Os07g45070	VQDLIQKHLAAVTTPQISLEPSNAN	T	1235							
9231	LOC_Os07g46540	LVSKGEISSNTITALWDYFCFHING	T	658							
9234	LOC_Os07g48602	SMTKPSSPLPDGTISSSLLSIPMPV	T	198							
9237	LOC_Os08g01100	KSGARTRKRVEATDSAVLKRARDGS	T	16							
9240	LOC_Os08g02700	AGVLAGIKVDKGTVELAGTDRETTT	T	108							
9243	LOC_Os08g03520	GGGGGGCYNCGETGHIARECPSKTY	T	185							
9246	LOC_Os08g05660	DIAALLPSKTHGTFLKMFLGPVNLR	T	91							
9249	LOC_Os08g07830	DDVLSAAQTAAETAERAASAARAAA	T	468							
9252	LOC_Os08g08390	KVATQRKHGAPYTRRTDILWKIALF	T	345							
9255	LOC_Os08g08720	IPQVSSEENEMLTKEFSEQEVKHAI	T	20							
9258	LOC_Os08g09910	SLKKLTMVKCSITMDLSICAPNLEL	T	280							
9261	LOC_Os08g12680	VRASDGKGVVYQTRNKKEVALDMIN	T	1013							
9264	LOC_Os08g14050	FIMVVPTQPYPFTFFCSESDLVIQI	T	206							
9267	LOC_Os08g14340	RSTMEAELTALDTATVEAEWLRELL	T	1310							
9270	LOC_Os08g16559	WSALVERHRFLLTTLVVLVFLCTIY	T	47							
9273	LOC_Os08g19310	AVETMKSNGIQPTNVTYGSLMYWMC	T	641							
9276	LOC_Os08g22864	APNEGQGWRRNSTVPQEGYGTASVI	T	585							
9279	LOC_Os08g24760	KLVEFGFTSSAETRSFAADIYAKVP	T	58							
9282	LOC_Os08g25670	ETEVGIDGVSESTACKDGDVACEKS	T	351							
9285	LOC_Os08g27870	LTRNVALIRDLNTNIARVVDLYANL	T	138							
9288	LOC_Os08g29650	SDSRSPPPRRRSTSRSPRPRRHGRS	T	57							
9291	LOC_Os08g31240	GVPYKASAFLIPTSTCLVELIETPS	T	774							
9294	LOC_Os08g33120	PSARTIAKSFSRTKDMTWRPDLFSD	T	67							
9297	LOC_Os08g33370	EEMVEYMEKVAKTVDVEELTVEERN	T	32							
9300	LOC_Os08g34230	ELMRNSKSYNESTDKHLKTLSESLV	T	39							
9303	LOC_Os08g36994	PLSSDTVVGQGSTSAWKMCGLDRKT	T	442							
9306	LOC_Os08g38320	CGSGKVSDTADSTESGKSSMCRPST	T	141							
9309	LOC_Os08g39390	RAPLFADLNCIPTCCGDDNDGGDPM	T	162							
9312	LOC_Os08g40230	SECTELMEAENPTDVDEFSKMENKS	T	240							
9315	LOC_Os08g41340	GLFFMETSALDSTNVRTAFEIVIKE	T	161							
9318	LOC_Os08g42700	LVRQFKANSREGTGKTPQGTPSGVS	T	250							
9321	LOC_Os08g43090	QQLQIQQQQSSQTTQQGQQQQPQKS	T	451							
9324	LOC_Os08g43560	SDKDIVALSGGHTLGRAHPERSGFE	T	161							
9327	LOC_Os08g44430	CLQKLPPNTSKSTYSCDGHTFNFLV	T	49							
9330	LOC_Os09g01540	PSDQPPTGVQSATAETATTQEPPTG	T	488							
9333	LOC_Os09g04110	KTVSPADDILRLTQMNTDLFGGVDA	T	182							
9336	LOC_Os09g07430	GTQDSKEFVRRCTSCQRQGGITTRD	T	590							
9339	LOC_Os09g07900	GALLFTTVGNNATFQPNPNSVYQTE	T	3357							
9342	LOC_Os09g10340	SGFNIPDLFPTWTGILATVTGMKRS	T	224							
9345	LOC_Os09g11250	FSKSLLDVADNLTRASSVVKESFSK	T	178							
9348	LOC_Os09g13120	GIRFQWFRSEVPTVGASCCRNDNRI	T	34							
9351	LOC_Os09g13700	PTTGVGPLPACPTTPGAPDSQDGPE	T	1210							
9354	LOC_Os09g14960	RIKQEEAREKKETTLYKKYTELLLA	T	485							
9357	LOC_Os09g16330	LYGLVTSQFGDVTDTFDNGVRISDF	T	369							
9360	LOC_Os09g16458	HNVSGIIKPHRMTLLLGPPGSGKTS	T	164							
9363	LOC_Os09g17730	RSELAQVQELYKTQVDKFVAEWCNS	T	344							
9366	LOC_Os09g19640	FRGAANSLSQLYTLAMGGQKLSFQA	T	37							
9369	LOC_Os09g19952	PIKKNHVSYNNCTKYSKEIEIIFRS	T	95							
9372	LOC_Os09g20590	DMEPGPSAAHTSTTTTTSKEDDSDF	T	299							
9375	LOC_Os09g22150	PETRTGAQVFELTKNIKVVFRKGKK	T	231							
9378	LOC_Os09g24250	PLFNILKKFDGETVTEVVRPSIARM	T	423							
9381	LOC_Os09g25370	GLGLSGMLASLATTGFQASNLGDAV	T	64							
9384	LOC_Os09g26560	LYYNICLVTVHCTSNLPKKSFNDDT	T	229							
9387	LOC_Os09g26970	NIGAEDWVDEANTQQLSQQQPSSPQ	T	1310							
9390	LOC_Os09g28310	MPPRPVPVVPKTTAFLGNFPGANDA	T	201							
9393	LOC_Os09g29630	QTKRMCTANLIVTNHEAQNFPGCNL	T	254							
9396	LOC_Os09g30070	NENGNSPGQLQNTLDVVKTTAALPS	T	879							
9399	LOC_Os09g30439	APTIPGTAVPVATQRRHQQGDTAAA	T	798							
9402	LOC_Os09g32090	WERQIRIRGEGGTKFADVYYTSPTG	T	164							
9405	LOC_Os09g32540	VSKDQLSGQPNMTNLSGESIVGKEV	T	273							
9408	LOC_Os09g33600	GADSQATDRDGRTALQYAIDSGTID	T	811							
9411	LOC_Os09g33960	PTDETLKKNDSGTRVSSTPVSINNG	T	121							
9414	LOC_Os09g34110	LEKKPENRGTMATIPRRKPPVDEKV	T	328							
9417	LOC_Os09g36270	MIPLARELLRNGTEVVLVANSLPAL	T	757							
9420	LOC_Os09g37006	IVGLHQNTTTGSTDITDMWEPLEEG	T	83							
9423	LOC_Os09g37860	FREHRFHTSLDKTRRFYPHVNNMDG	T	525							
9426	LOC_Os09g38580	PSIKNSDYNIRNTTFALVIMIQYIF	T	209							
9429	LOC_Os09g39330	CDGLDKDLFAAATKVTVGDGNTARF	T	866							

9432	LOC_Os09g39380	VGDTILFGDSDPTSAKPKFGSYWIK	T	388							
9435	LOC_Os09g40050	GERRLLVYGDGSTPQGALQAAGALL	T	222							
9438	LOC_Os10g02509	PGTHTLQMMQQQTEPQLQPPPPPQQ	T	154							
9441	LOC_Os10g03130	HEGICGSHASGRTMVGKAFRQVFFW	T	380							
9444	LOC_Os10g07180	AAQNSGKHIHMITRAQRVVMKRLGI	T	493							
9447	LOC_Os10g07300	ISEVLSESKTRYTQIQKLRHAIFIT	T	145							
9450	LOC_Os10g08022	GVILFDETLYQKTKDGKPFVDVLKE	T	83							
9453	LOC_Os10g12808	EELKEKGYIRPSTSPWGAPVIFVEK	T	936							
9456	LOC_Os10g14290	DLESGEDSSDSVTSDAGSAKAAPDD	T	50							
9459	LOC_Os10g17980	TKALGNKEHRGRTQGVGSSVPWKYG	T	351							
9462	LOC_Os10g20240	QERKEVGGGAVETAAERGGGGWGRA	T	48							
9465	LOC_Os10g20600	NHFVTMPKADTRTPPAFASDPLKRV	T	152							
9468	LOC_Os10g21840	RFNECRNTIPEITDSSVIRTFKSGV	T	349							
9471	LOC_Os10g25110	SSGKMFVRDSAGTPFVRNSGKQKRS	T	797							
9474	LOC_Os10g26600	KGSKVKYELDKKTGMIKVDRVLYSS	T	61							
9477	LOC_Os10g28610	NELQNEIQSLRSTISQLNTEKDATL	T	437							
9480	LOC_Os10g30580	SRGFGSEFRFERTEAGAGAAADPFA	T	783							
9483	LOC_Os10g32960	KTMQRVLLNCMYTSLDYAQTGLIAA	T	279							
9486	LOC_Os10g34380	RESKDITSTSRWTKVKENFRSDARY	T	845							
9489	LOC_Os10g34820	PNRHLISSCQGSTPKQGTLLHSPLM	T	360							
9492	LOC_Os10g35580	ESSKGGKETNRRTSVPSQAPAKSSS	T	145							
9495	LOC_Os10g37230	ACVKHEYAPAEATKMDGAIQTVYPR	T	130							
9498	LOC_Os10g37480	RNVVEIKGRFSVTSENVDLAKVQEV	T	565							
9501	LOC_Os10g38060	PMWPEGNPTGAPTQRILYWQHKTMQ	T	822							
9504	LOC_Os10g39440	TLVPLYISETAPTDIRGLLNTLPQF	T	127							
9507	LOC_Os10g40800	AGTINVLLALTTTQKGPMSISEYIA	T	140							
9510	LOC_Os10g41440	LYGRDPALSTLRTSGIHGGGPGGPL	T	315							
9513	LOC_Os10g42196	LSASHGLSPKRPTHEFGNAQNNLAH	T	290							
9516	LOC_Os10g42439	DRAAPEGNATEFTLTLRTDARGKFK	T	101							
9519	LOC_Os11g01860	NRAYGFFCSLTSTNIVTWNEMIAGY	T	361							
9522	LOC_Os11g02190	LDLDSGFISSDITKETHTKPSPIES	T	604							
9525	LOC_Os11g04190	VEIYSLQSSRNPTPRGSSFNHAEFF	T	259							
9528	LOC_Os11g07280	ASKPAQPVVNSVTVSSRRPVVLADG	T	509							
9531	LOC_Os11g08460	KMRETAEVYLGKTVTNAVITVPVYF	T	153							
9534	LOC_Os11g08550	LEAHRRSPISSSTASREAVLKYRLD	T	747							
9537	LOC_Os11g09420	PFTWTEEAENALTQLKAYLSSPPVM	T	856							
9540	LOC_Os11g10490	AEKGKRPADEAGTSSGSASKKSRSD	T	299							
9543	LOC_Os11g10710	HVWNITTLDVHPTEPYLLSIGSQDQ	T	455							
9546	LOC_Os11g12810	TDVPHIYRLAAKTKGVFINPALVEP	T	631							
9549	LOC_Os11g15750	RGKRSRNLMPKETYYIAALNDDGKP	T	619							
9552	LOC_Os11g16400	QQTVDEFLALEATLARSAAAGHAED	T	324							
9555	LOC_Os11g16750	LKSRTDLVNLDITNELHPKELPNGK	T	598							
9558	LOC_Os11g20040	ADDTTDTGTYRLTPLSCLLVDGDDD	T	101							
9561	LOC_Os11g20420	IGAEHVIADIETTEDWRTPLIKFLS	T	405							
9564	LOC_Os11g28230	VEDRVDTPGYTPTPSPGQNETGVES	T	543							
9567	LOC_Os11g29000	GQGHYKMDNLVHTFAKNVVESESLV	T	156							
9570	LOC_Os11g30970	ESRWFSREMVKTTESARTACQALRL	T	802							
9573	LOC_Os11g33330	FGDKGYLAFEHITWAPYQTKLIDTT	T	642							
9576	LOC_Os11g34200	FADQGEALPGEETVPETIHGTESVP	T	179							
9579	LOC_Os11g35080	KKEAAARGAGKKTTKKPAAAAAAAT	T	94							
9582	LOC_Os11g35590	SSRVDANGRLLFTEEEWLAKFRKAA	T	318							
9585	LOC_Os11g36340	HGVEDATGPDRATTLVASNEVPVQG	T	418							
9588	LOC_Os11g36880	SDFAGWWLAARQTVAKIDRKTFDAG	T	948							
9591	LOC_Os11g37660	AAKGFASDLLVATALIGMYAEAGDM	T	158							
9594	LOC_Os11g37990	IAKIALKRGVQLTTSERMAEDIKKL	T	435							
9597	LOC_Os11g38620	IVRTANLYWNSMTQEQRGEFLSVRF	T	353							
9600	LOC_Os11g40140	GCAMSGLIADARTLVEHARVETQNH	T	87							
9603	LOC_Os11g42040	MMEELGRQVGTSTESTSLRVMTENQ	T	282							
9606	LOC_Os11g46200	AGGDGGPFDELKTRVEDVSKRFSDS	T	109							
9609	LOC_Os11g47240	ELTFLGLGYNQLTGPVPSTFGNIRP	T	368							
9612	LOC_Os11g47760	AEDKTTGQKNKITITNDKGRLSKEE	T	508							
9615	LOC_Os12g01260	VLVMSLCFIGFVTALHVFGKLYRSR	T	88							
9618	LOC_Os12g03880	NGYKNVLAVAVETEYSYPHADKIKE	T	258							
9621	LOC_Os12g04050	GSVNPAEFLQVYTTGIEAAGGDDRV	T	146							
9624	LOC_Os12g05110	FAGLAKAVKPGDTIFVGQYLFTGSE	T	147							
9627	LOC_Os12g05930	KELDLGVNQLTGTLPKWIGQLTSLV	T	149							
9630	LOC_Os12g06560	LRAAGIRGPLSITAFGDVVQLARPS	T	95							
9633	LOC_Os12g06890	GASSTPAFGATTTPAFGTTTPAFGS	T	195							
9636	LOC_Os12g07230	FTFFDKDGSGFITIDELSQACEQFG	T	443							
9639	LOC_Os12g07590	SYHLDETVKTLRTQRVGMVQTEKQY	T	297							
9642	LOC_Os12g09739	FIKLDRYGDCSRTAQGHGLRRTTPE	T	146							
9645	LOC_Os12g10700	EFQHITCTPVYDTTGANNEDEYSLT	T	86							
9648	LOC_Os12g13150	KVRISPQSKHIATLDLNGSVNIFVL	T	303							
9651	LOC_Os12g14984	WSRFTNLVLSGPTLSIPDYVLLQHF	T	464							
9654	LOC_Os12g16430	YAYGIDTNWYVDTGATDHITGQLDK	T	326							

9657	LOC_Os12g18860	PEYLRHLKKFVNTVEKSPSPRNAWR	T	260								
9660	LOC_Os12g19290	MLSSKDTNFLNFTYKNLELSDDPEH	T	486								
9663	LOC_Os12g21890	AAGREAQSSIFSTSGLSSWAKNLKI	T	81								
9666	LOC_Os12g22530	HQNVVLDICQETTFFLHDASLEKEN	T	63								
9669	LOC_Os12g25690	DKAQISIYDPQVTEDQIQRDLAMSK	T	368								
9672	LOC_Os12g26240	ASAPATPRQRMITGDEVPVKEVALG	T	780								
9675	LOC_Os12g27994	IDAHLPLPGPDSTPTSSASTVAQNQ	T	74								
9678	LOC_Os12g29660	AHKDEIYSRPKRTWFATEKEKKLLA	T	626								
9681	LOC_Os12g32374	FINKHMLYHYQDTGHCLALSFSDLS	T	119								
9684	LOC_Os12g35290	NGPPPVANDDSGTSSAPAQPDGQTT	T	593								
9687	LOC_Os12g37060	ALGFDLSNLLVFTPMTIEMMKKRHK	T	138								
9690	LOC_Os12g37560	GVRADCVMKKTRTIEDQWVPMWDEE	T	510								
9693	LOC_Os12g37780	LLSQWNLDDDCCTRIQKRFAVFLLY	T	242								
9696	LOC_Os12g37890	QFLAVNHINCLATLEGFQSCANLLV	T	1298								
9699	LOC_Os12g40790	LIERWLRFGPARTLQTVVGSSGGDY	T	370								
9702	LOC_Os12g41180	YNRTYRSELPSKTTPNKIARPARRA	T	405								
9705	LOC_Os12g42550	VVSVEMPAPEGWTKKFTPQRGGRFE	T	40								
9708	LOC_Os12g44150	RASRTENQDAIDTAIVGMLADPKEA	T	380								
9711	LOC_Osm1g00310	RSQIPIAFLVDSTIPGESHKRITYP	T	231								
9714	LOC_Osm1g00590	PEKRVVSEPIEMTQEFRYFDFASPW	T	172								
9717	LOC_Osp1g01070	FGILLFMVLISATRDFRERTKSKLV	T	34								
9719	LOC_Os01g01689	LQKTGCSHVLDVYCQGLSNQKSCLQ	Y	1849								
9722	LOC_Os01g04650	AAAAAAQQPPAGYVYAQMHAPPPQQ	Y	444								
9725	LOC_Os01g08380	SDWTRLGFAEVDYGWGPPAHVVPLT	Y	382								
9728	LOC_Os01g08960	LLQTCLDELATRYPATKFVKIISTD	Y	147								
9731	LOC_Os01g10840	PTREEIRCMNPNYTEFKFPQIKAHP	Y	297								
9734	LOC_Os01g12390	AYYYSKDEKLLVYDFYSRGSVSNML	Y	411								
9737	LOC_Os01g12770	DVVVDLSDLLIAYSNTVLTRIAFGD	Y	197								
9740	LOC_Os01g16110	INEGLYYYENHAYNQRSSQAGTIDI	Y	615								
9743	LOC_Os01g16850	LDDRSTPGVFIGYAEGSKAYRILDP	Y	1004								
9746	LOC_Os01g19894	STDPPQGEVGMDYWHAAPQVTQPTQ	Y	1896								
9749	LOC_Os01g20950	YPTVPSGLDTPYYRHPDGWYTFLPA	Y	44								
9752	LOC_Os01g23530	NDKDLNLVSLRFYLLRKNGYNMSSG	Y	122								
9755	LOC_Os01g25610	LPLILIIRNRLKYALTYREVISILM	Y	54								
9758	LOC_Os01g27040	IAMQLRKCCNHPYLFQGAEPGPPYT	Y	510								
9761	LOC_Os01g28660	SGQLIGSGFRHNYKVALSVRTGTDE	Y	324								
9764	LOC_Os01g32720	TQAGYGQVKAKEYHRSDSLPVKKQA	Y	654								
9767	LOC_Os01g34030	AAWLAVSELGEEYDTVWDRLRGLRS	Y	69								
9770	LOC_Os01g34780	RDYCAQVKDSSVYLAVASNISGSMP	Y	128								
9773	LOC_Os01g36790	HGHLTEKADVYSYGILVLELVTGQR	Y	527								
9776	LOC_Os01g37832	WELAENLQGVLEYQTRYSRFQGVAN	Y	131								
9779	LOC_Os01g40750	ETTDFCTTKSLCYHREYKTNDTSET	Y	144								
9782	LOC_Os01g41640	SADDEILVRDVLYDALILVDYSFIN	Y	285								
9785	LOC_Os01g43410	PGEHFKDLVGSAYYVAPEVLKRNYG	Y	232								
9788	LOC_Os01g46750	GEYVAVENLENIYGLVSAIDSIWVY	Y	536								
9791	LOC_Os01g46980	KEKQVEVRKKIEYSMQLNASRIKVL	Y	70								
9794	LOC_Os01g49200	TEPNTLSTDFLNYVESEVLRLEQLK	Y	298								
9797	LOC_Os01g49529	LNIAVESAAALTYLHAIEPPIVHRD	Y	497								
9800	LOC_Os01g51230	GNGATRSNHSWSYLEGWMATKPWES	Y	247								
9803	LOC_Os01g52950	AVQRRDDESLRSYIQRFCQVRNTIP	Y	337								
9806	LOC_Os01g55260	VPYDTLVEKTEGYSGSDIRLVCKEA	Y	304								
9809	LOC_Os01g56490	VSDEQFAKWKFAYIAHNRLAGEYFQ	Y	1047								
9812	LOC_Os01g58870	LYTSSVLPIGALYSLSLWFSNSAYI	Y	109								
9815	LOC_Os01g60190	ADRMVMLAKALEYADFDKFDRVRVP	Y	299								
9818	LOC_Os01g62310	REHGHIEGKNVFYWFQNHKARQRQK	Y	91								
9821	LOC_Os01g63170	WRKFVSKVREKGYSLAIVVQKTKCV	Y	102								
9824	LOC_Os01g64670	DKIIAVCVDDPEYRHYNDLSELSPH	Y	149								
9827	LOC_Os01g67126	RRVLTLRGLDQEYEGNVEATGEDYY	Y	117								
9830	LOC_Os01g68120	SPGFVPNEEINEYHVGTTGAKVTAD	Y	519								
9833	LOC_Os01g70810	STVLQTINRLRFYRTQDISSKARNL	Y	340								
9836	LOC_Os01g73300	SAFSLLNTVLEEYHKTFGERWRTAK	Y	117								
9839	LOC_Os01g74260	RTLVGKVFRQGFYWPTALNDAVDLV	Y	1569								
9842	LOC_Os02g01030	KAILLNPLSAIMYGTRASVFIKMKK	Y	166								
9845	LOC_Os02g01390	ALVKFMAVVHYAYLQMKMPGPGGPI	Y	721								
9848	LOC_Os02g02290	RGVHLAFQGDGDYDSQFKSEQAFAD	Y	1805								
9851	LOC_Os02g02870	RGGGGSGGGGRSYGGSWGGGRRSGG	Y	107								
9854	LOC_Os02g03890	ATQVGTYFLRNYYNLLQQSPDVVHQ	Y	21								
9857	LOC_Os02g04660	DPLPEQERFEINYRDFLQSPLQPLM	Y	317								
9860	LOC_Os02g06740	RHGGGEGVILCLYVDDILIFGTNLE	Y	863								
9863	LOC_Os02g10220	EPAPVAAESKSLYEILGVERTASQQ	Y	32								
9866	LOC_Os02g12310	EVPTAVKRRFVFYLGHHQPSGSNNN	Y	119								
9869	LOC_Os02g12610	AGDGDDGGEGVKYLRMERRMGKFMR	Y	108								
9872	LOC_Os02g13250	VVHEGFESYDIDYPTADGVSVLGDA	Y	346								
9875	LOC_Os02g14059	FGIQEHIDLGIKYDPSTGIYGMDFY	Y	118								
9878	LOC_Os02g19420	IVASLLQEIIVVYPALSPPTLSLGA	Y	67								

9881	LOC_Os02g25580	RDLQKMVQALPQYSDQIEKLSLHVE	Y	369							
9884	LOC_Os02g30680	GTHMAILDEVNEYGENVLRRAFWSF	Y	411							
9887	LOC_Os02g32490	ALVPSPKKYQKMYERSINDPAGFWS	Y	75							
9890	LOC_Os02g34460	EICKYLFQEGVLYAKKDYNLAKHPQ	Y	22							
9893	LOC_Os02g35150	TNSDVDSDLETQYQRSSSEGSKLGM	Y	584							
9896	LOC_Os02g36974	RGNEDRCTLIKEYRGKIETELSKIC	Y	92							
9899	LOC_Os02g40880	RFVEIGRVALVNYGKDYGRLVVIVD	Y	17							
9902	LOC_Os02g41990	PMFVLPAVSSVIYSTVVKFTRMLER	Y	123							
9905	LOC_Os02g43370	FFNKDYIPNWLAYAGYALLSIVAVI	Y	394							
9908	LOC_Os02g44740	PSSPPRSPRRSAYYVLSPAASHPDV	Y	28							
9911	LOC_Os02g44970	VFKEIIDKDVVAYTSMITGYSEIVD	Y	98							
9914	LOC_Os02g46090	LEREEHLVAAFSYFDKDGSGYITVD	Y	476							
9917	LOC_Os02g47810	PRCESTHTKFCYYNNYSLSQPRYFC	Y	78							
9920	LOC_Os02g49070	WNEVLKISIPRNYGPLKLEVYDHDT	Y	235							
9923	LOC_Os02g51200	FQLRRGEVAVSLYHPQAFLIKFQHR	Y	72							
9926	LOC_Os02g52390	LYQSGYQLNGSAYVISKHISNTWLW	Y	915							
9929	LOC_Os02g53780	LQPSSSLEERRYYDGRFSDQQMALL	Y	206							
9932	LOC_Os02g54990	LMFFVPNGVVSFYESISKFGSGLFL	Y	141							
9935	LOC_Os02g55330	FWRHPSARLTLLYSHGNAADLGQML	Y	39							
9938	LOC_Os02g55560	RSGDWSDIGGRDYMEDAHVCISDLA	Y	66							
9941	LOC_Os02g57260	SASACAAGDSAAYQRTSAYGDDVVV	Y	39							
9944	LOC_Os03g01040	ALVKFMAAVHYAYLQMKMPGPGGPI	Y	691							
9947	LOC_Os03g02680	EQSWPGVSSLPDYKSAFPKWQAQDL	Y	275							
9950	LOC_Os03g03830	KQVLDSREKLEYYRTKMQDLVLYKS	Y	633							
9953	LOC_Os03g04460	VPDQKSGKLDLQYLHDHFALNSTIG	Y	110							
9956	LOC_Os03g04590	RQRKPWRRKDGVYMYFEDNAGVIVN	Y	95							
9959	LOC_Os03g06120	LLVHSCQDRSYLYLLAMGWNIVYQF	Y	98							
9962	LOC_Os03g06540	KARLVAKGFKQRYGIDYEDTFSPVV	Y	812							
9965	LOC_Os03g07840	RRCGWLDIVALKYCCEINGFSSLNL	Y	380							
9968	LOC_Os03g09260	ATCYANSILQCLYMNTSFRLGIFSL	Y	125							
9971	LOC_Os03g10510	KGGLYTLDVSSVYKYKSTLVDVKVD	Y	61							
9974	LOC_Os03g12010	HWHWHKKRCTGSYHRVQGSSSGCVE	Y	195							
9977	LOC_Os03g13720	SASQLIRNYIIGYHVLPNTMLESSF	Y	470							
9980	LOC_Os03g14450	RAAVPSGASTGVYEALELRDGGSDY	Y	47							
9983	LOC_Os03g15810	DELVQLVDTMVSYSITYRNTKLEPQ	Y	829							
9986	LOC_Os03g16369	QHTPSRVGMHNSYSKDGMNSEDAIK	Y	643							
9989	LOC_Os03g16800	WCKETGVARSSEYPEVQRVTDKLLE	Y	336							
9992	LOC_Os03g18600	FRVVGGEHRLKNYLSVTTVHPSPSA	Y	162							
9995	LOC_Os03g19870	ISVIERRADQLDYVLVDTPGQIEIF	Y	152							
9998	LOC_Os03g20680	QTADATRDKLGEYKDYTADKARETN	Y	115							
10001	LOC_Os03g22120	WGLEKGWGDTAGYVLEMIHLLLDVL	Y	246							
10004	LOC_Os03g22340	TSDGPFSKRYLKYLTKKYLKKHNVR	Y	88							
10007	LOC_Os03g23990	DGSTDLEEFLQVYSTELATRRVMTT	Y	422							
10010	LOC_Os03g24110	EEKTTFITPIGTYCYTTMPFGLKNA	Y	917							
10013	LOC_Os03g27030	DRGYRDDYRDRHYRDDYQDREYRRS	Y	144							
10016	LOC_Os03g28330	QMNRVRNGELYRYICDTKGAFVQPA	Y	663							
10019	LOC_Os03g29075	SVPQYPLYPVYGYPPSTVARQVGDL	Y	22							
10022	LOC_Os03g32630	NDADDNERLAEVYEKLNLRDSDAAR	Y	267							
10025	LOC_Os03g39650	DKLTEDDVAKLSYLQLVIKETLRLH	Y	358							
10028	LOC_Os03g43670	EDLKIRRAQEFSYEELEQATGGFSE	Y	494							
10031	LOC_Os03g44310	PMHPSVYKVGLQYLSGEVSGGNGRC	Y	298							
10034	LOC_Os03g45140	FCNVLKMRGPDQYWRIDPRWVPRLR	Y	858							
10037	LOC_Os03g46570	VRQPAAAEVMRQYSQVCAAAADAAE	Y	81							
10040	LOC_Os03g46710	VKENRNFGSTPPYKVEPPTLDLADE	Y	646							
10043	LOC_Os03g47930	LDDVDIDQVSVDYVLNCAKKGEALD	Y	54							
10046	LOC_Os03g48930	KNPYYVNVLLAGYDSDVGASLYYID	Y	106							
10049	LOC_Os03g50350	NNNLMGQFHNALYLGDAMKRVEILE	Y	740							
10052	LOC_Os03g51360	NRAVPRDVIKSAYNKKKARRPRKKK	Y	463							
10055	LOC_Os03g52970	NRIGNLLVPNDNYCKFENWIMPLFD	Y	184							
10058	LOC_Os03g56430	TDVHSMPKKSQKYREMKSKEGIQNL	Y	583							
10061	LOC_Os03g56900	VIGGWCNFLTVIYIGFVSAHTLPVL	Y	190							
10064	LOC_Os03g58740	RPQEVHHRPTKSYTGNPPQPLEKDA	Y	223							
10067	LOC_Os03g61340	RGYRCVAPDLRGYGGTTAPPEHTSY	Y	69							
10070	LOC_Os03g63140	TGNAPVMTSEADYIKRYQQRKYEST	Y	220							
10073	LOC_Os03g63590	SFEVIKYRLKQIYESVESSTEESDV	Y	550							
10076	LOC_Os03g64340	PPPPPPPPPPPVYYSSYVMLDRPPP	Y	368							
10079	LOC_Os04g02000	ASANWPYALAGRYGMQAAGWPFGGN	Y	153							
10082	LOC_Os04g02320	REEFLLLTKQNGYGKCICEKIPSLK	Y	52							
10085	LOC_Os04g02820	TYEDTLLGDVQVYPEKGTVAFSAGL	Y	203							
10088	LOC_Os04g06770	VGRKVIDKLQQTYRSELSSKDFAYD	Y	108							
10091	LOC_Os04g08440	ISKRYVLVEGTLYRRAANGILLKCI	Y	1566							
10094	LOC_Os04g17440	EEAERALTQLKAYLSSPPVLVAPEP	Y	1111							
10097	LOC_Os04g19990	SHTDPKQPFEHWYTSGQSKTIRIAV	Y	74							
10100	LOC_Os04g22400	PPDGFRQIQGVDYDETFSPVTMLKS	Y	484							
10103	LOC_Os04g23060	VLVEMARTMLDEYKTPRKFWAEAIN	Y	282							

10106	LOC_Os04g28180	AVTRKTRILDVVYNASNNELVRTQT	Y	84							
10109	LOC_Os04g30010	SACYNTSSRDMDYNDWQINFTGTPL	Y	124							
10112	LOC_Os04g31330	SPLGRHLVKLISYLEANLEEKSKLY	Y	501							
10115	LOC_Os04g34400	LRLPLKIKIFLWYLKEGVILTKDNL	Y	496							
10118	LOC_Os04g35950	WPPRFWPTIAEKYDGSVNPAEFLQV	Y	245							
10121	LOC_Os04g37920	VLQAAGVELGSNYPLPIVGLDAANA	Y	478							
10124	LOC_Os04g39980	SGYVPPGTANPLYEAFGLCDAAAPA	Y	86							
10127	LOC_Os04g43140	IALISPADKAKFYSLCKSLSKENLQ	Y	623							
10130	LOC_Os04g43430	SGSPGDVLGAFRYIRCTAGGRPDQF	Y	147							
10133	LOC_Os04g45190	SCSMQLTNRTDDYIAFKVKTTSPKK	Y	39							
10136	LOC_Os04g46320	ADFQPRISDFGLYLLLNPAAAQQML	Y	238							
10139	LOC_Os04g47912	QYPCGFPTILSWYIAAGILFKSGHY	Y	320							
10142	LOC_Os04g51060	LGRKACLEGASLYRQNYSSQLGYMG	Y	563							
10145	LOC_Os04g52200	EAEEALKYFNNTYIDTCKITCEVAR	Y	87							
10148	LOC_Os04g52850	SGGKDGPSIRLSYHRHAYGLGEHYN	Y	306							
10151	LOC_Os04g55150	YVRLELLEGVVAYHNGNTVKARESL	Y	279							
10154	LOC_Os04g56290	DAIVFESELDRIYLGTPSKIAIIDH	Y	237							
10157	LOC_Os04g59494	IMLGCPTGNLIPYPSIRLSFFLSFH	Y	903							
10160	LOC_Os05g01360	DLLEVAIRNIIRYLCASREKDLSSS	Y	472							
10163	LOC_Os05g02310	HRLAEIRRFFEDYKKNENKEVAVND	Y	176							
10166	LOC_Os05g05480	LVPFHVEGGQPSYLIVAGLVFTPLT	Y	429							
10169	LOC_Os05g07120	IPPEANEVTVQCYAGGEHTAAARTY	Y	142							
10172	LOC_Os05g10540	YFQAHRVTVVSSYPLGQILHNREGT	Y	1307							
10175	LOC_Os05g12680	RVHHKHLVSLVGYCISGGKRLLVYE	Y	363							
10178	LOC_Os05g14770	NGLGAVSPPVRVYGKMMWRSDRLKS	Y	224							
10181	LOC_Os05g19560	QGANQRVRYTNPYPGGSSSQQQQQQ	Y	562							
10184	LOC_Os05g23080	FRIDLAPGTTPLYKRPYRMAANELA	Y	852							
10187	LOC_Os05g23940	CGLCGKVLDDQVYDGEPTFQKGADG	Y	35							
10190	LOC_Os05g26890	LRSYTSVIHANVYQTIKILYEGAKE	Y	47							
10193	LOC_Os05g32600	WPDMVYLPDYVEYQFVSAPPLRSLF	Y	253							
10196	LOC_Os05g33380	DAKKVSPEVIAEYTVRTLQRTVPAA	Y	246							
10199	LOC_Os05g37434	KTTQEHCPNLQYYYLGYYIHSCNKM	Y	469							
10202	LOC_Os05g39090	PEIDIWSAGVILYILLCGVPPFWAE	Y	264							
10205	LOC_Os05g42230	ESAFAVKWSVQNYLRPGDAVVLLHV	Y	81							
10208	LOC_Os05g44100	EKVVEVINPEDDYVWVHDYHLMALP	Y	223							
10211	LOC_Os05g44922	YIIGGDGTQKGAYEIFKEIRKRGLK	Y	269							
10214	LOC_Os05g46330	FTSSARTDNLQLYHWVRVVNGAPPT	Y	90							
10217	LOC_Os05g48310	IEGVNTKEEVGWYAGKRIAYVYKAK	Y	48							
10220	LOC_Os05g50280	TTIRFADIKQKRYSVPLMIGIGLLV	Y	294							
10223	LOC_Os05g51180	FGEGDANGFEGGYGGGGGFGDGGLA	Y	108							
10226	LOC_Os05g51500	VFIVALNKVDRLYGWKKCTNAPIGK	Y	745							
10229	LOC_Os06g02130	LQPNASNGNTQVYMNGREITKIELR	Y	362							
10232	LOC_Os06g02510	PLRPIVQCQTLKYNMKSRAGRGFTL	Y	65							
10235	LOC_Os06g04510	TGAPCRSERLAKYNQLLRIEEELGD	Y	418							
10238	LOC_Os06g05250	TPTGVLYTGQVGYVISGMRSTKEAR	Y	320							
10241	LOC_Os06g06090	PQRNSFNDVYIAYELMDTDLHQIIR	Y	148							
10244	LOC_Os06g09880	PDGKSLLSILDAYEKMGKHEKGLSL	Y	613							
10247	LOC_Os06g10710	AEAADERDAEEGYIGGNLHVSVRLP	Y	209							
10250	LOC_Os06g12610	IIWYTLMLFMFEYRGARILITEQFP	Y	154							
10253	LOC_Os06g13390	PQSVVKLFQEQFYKDFSLFLTLRHE	Y	171							
10256	LOC_Os06g16020	TDDKGLSPGPYKYLRSFSSVEDAGS	Y	162							
10259	LOC_Os06g19390	VSQLMVFLWVLYYLITVEGGGATEQ	Y	467							
10262	LOC_Os06g20490	GSLKVHNGTQEGYCGSYLKAWLEVV	Y	205							
10265	LOC_Os06g22700	DGSPPYGRGGRSYGRGSGAPGKEFI	Y	54							
10268	LOC_Os06g26340	RWYRAPELCGSFYSKYTPAIDIWSI	Y	206							
10271	LOC_Os06g28140	PDVSYAHSATSQYQSAPVYGGQEEL	Y	750							
10274	LOC_Os06g30570	VNEPTNVNVKCNYTQMADRDEEQIL	Y	54							
10277	LOC_Os06g34710	MQDLPKRDDGLCYYGAYLQRLIWAQ	Y	237							
10280	LOC_Os06g36730	VWTQVGEIVFPVYTTVPISAGPSTT	Y	78							
10283	LOC_Os06g40640	QGLDSLGARCAKYYEAGARFAKWRA	Y	162							
10286	LOC_Os06g42030	LVGRIGPKEYRIYRCIVRYGYHDVH	Y	626							
10289	LOC_Os06g44030	LGGHSSCVWSLAYRALLFNLFCHCC	Y	582							
10292	LOC_Os06g45980	DHQRPSNQTETEYVSQMLTKIKRFA	Y	597							
10295	LOC_Os06g47220	CRSVPHEHSAAYYGCGGGYDYEDVS	Y	16							
10298	LOC_Os06g51140	ADMAAADERAGRYPCPLCDRHFPTE	Y	56							
10301	LOC_Os06g51270	EMQRFYREYYKKYIQALQNAADKAD	Y	139							
10304	LOC_Os07g02610	LNILRKEMYRGYYTLDRFRCHVHEA	Y	102							
10307	LOC_Os07g05050	FKESAPLDSLCVYGGVPISHQMRAL	Y	190							
10310	LOC_Os07g07270	HSHFDRSLESGPYTLKYRGSMWGYK	Y	139							
10313	LOC_Os07g09384	VRDGAHARLRTRYGNYLRANGGLPP	Y	109							
10316	LOC_Os07g10600	EATCHAPDPVGCYKEIYRVLKPGQC	Y	237							
10319	LOC_Os07g12910	HSETLCGTCGGRYNANEFWIGCDIC	Y	197							
10322	LOC_Os07g18300	FSKIDLKLIERLYLPVLKEHHWFLI	Y	819							
10325	LOC_Os07g27950	QCLFHVLCVTVEYRRRREARGGGFG	Y	140							
10328	LOC_Os07g29450	LKKGEVIYLETHYNRYELCFSGEKA	Y	678							

10331	LOC_Os07g30250	GDATVILRYYCFYVICLAMNGTSEA	Y	139								
10334	LOC_Os07g30980	FLSGDEAFGYAQYVIKWEQIPIDKR	Y	1095								
10337	LOC_Os07g32400	LSQCRCPYITDYYGSYLHQTKLWIV	Y	77								
10340	LOC_Os07g36940	GSGPRRGAAPMDYGPRGSAAALASP	Y	1456								
10343	LOC_Os07g38090	DGVGLWYGRSKAYEARTPLMVAATY	Y	67								
10346	LOC_Os07g41810	EALGPTHVLHSRYATKPDLAAYAAH	Y	113								
10349	LOC_Os07g43240	KVDIKTVRKMAEYMNKHFAITNKEE	Y	147								
10352	LOC_Os07g44710	QFYDAFEDEDNVYIVMELCKGGELL	Y	219								
10355	LOC_Os07g46750	LKALEQHLSGKTYVSGNAISKDDIK	Y	26								
10358	LOC_Os07g47700	GARFLLTSTSEVYGDPLQHPQVETY	Y	243								
10361	LOC_Os08g01420	RQPPPPEDHLVTYKRRRSKETQPLP	Y	25								
10364	LOC_Os08g02690	VKLAASDLKELGYDDFHRYFVKKLV	Y	166								
10367	LOC_Os08g04840	VPMPYYGYVPVFYAPPGAVQAQHEV	Y	300								
10370	LOC_Os08g08550	LITTPGGVMTAKYYSPAVPIEIQGI	Y	735								
10373	LOC_Os08g10350	VSLVTILGGAVGYVMTDSGFSLTAY	Y	141								
10376	LOC_Os08g14570	VDLDEYAMEDEEYEERLKKEKISLF	Y	157								
10379	LOC_Os08g20020	DQPEANGVGKHQYHMDDKENKGLDK	Y	252								
10382	LOC_Os08g23360	FRAERCFYLAKSYSSAGKRAESYAL	Y	466								
10385	LOC_Os08g27674	SWQGYDISGYRFYTMFKDRKSAAQN	Y	825								
10388	LOC_Os08g29490	RAALVKFMVATHYAYLQMKMPGPAG	Y	667								
10391	LOC_Os08g31980	INTDDDHVWVHDYHLMLLPTFLRKR	Y	205								
10394	LOC_Os08g38590	TAAMQGADGTQMYVSQADGLATFGG	Y	167								
10397	LOC_Os08g42600	HLSLLSRYYKRAYQELFLLNDAKPP	Y	171								
10400	LOC_Os08g42980	PRRQHRGGGGGAYHHHQQHYQPHHH	Y	63								
10403	LOC_Os08g44870	YLFGLPVGYLLGYYFNLGVGGVWGG	Y	424								
10406	LOC_Os09g02810	LFWYWEDCLKQRYEKFVIALEDALK	Y	292								
10409	LOC_Os09g08390	GSSNDSSDGVDCYSYDHPFHQELYN	Y	260								
10412	LOC_Os09g12730	GDKGSSSSMDDRYSQHADQRRFTER	Y	133								
10415	LOC_Os09g15770	KALARPASYTTKYFGCELGAQSKFD	Y	58								
10418	LOC_Os09g19560	PKVDVIISEWMGYFLLFENMLNTVL	Y	182								
10421	LOC_Os09g23350	VTAVRDGMNLIPYEYVIARQGNEKL	Y	471								
10424	LOC_Os09g30150	DARSMSFVGTHEYLAPEIIKGEGHG	Y	435								
10427	LOC_Os09g33960	AKVDDEISPETSYQAPNVVETVTDI	Y	188								
10430	LOC_Os09g37006	DGGWEDDHAPPAYAGNGYTRGRGRG	Y	190								
10433	LOC_Os09g38200	SATKDLENLATDYFNDMFTADPTVD	Y	828								
10436	LOC_Os10g04580	FVELWDVSGHERYKECRSLFYSQIN	Y	105								
10439	LOC_Os10g20040	VTLTSPSGDVLRYLVRLDFRATNNM	Y	1311								
10442	LOC_Os10g22950	LEEDFMHIVLVHYLETKGGKSRTRG	Y	135								
10445	LOC_Os10g25220	SSKQTPTEQLLHYLPQFPHPQQHNE	Y	385								
10448	LOC_Os10g29400	VKKQWKILKGIPYNPYKNVQSDIIL	Y	702								
10451	LOC_Os10g32710	DSDDEMEDDGERYNRIPLSNEVVLR	Y	163								
10454	LOC_Os10g35450	RFSKENVIGEGGYGVVYRGRLINGT	Y	200								
10457	LOC_Os10g39200	SFNSKVSPSEVGYVQSREFSSELPE	Y	329								
10460	LOC_Os11g03810	VGGTTYFTHVVIYRDDGPAVWWVSL	Y	164								
10463	LOC_Os11g06320	QKVLSYSKYDLGYFHGLKLTRTSSK	Y	444								
10466	LOC_Os11g10040	SLVYSEKKFVARYIDPYKQAAPTLA	Y	363								
10469	LOC_Os11g10710	GFSEERKLGKGGYGTVYKGEHKNGD	Y	29								
10472	LOC_Os11g15170	QYSWGSAVLAATYAGLCDACVRNSK	Y	1015								
10475	LOC_Os11g23020	LTFTVLATEGLAYKPTPETRVHGAQ	Y	410								
10478	LOC_Os11g23960	GCVLMQDRKVVAYASRQLCPHENNY	Y	1177								
10481	LOC_Os11g29970	LMSGHGNNAVSDYLSVLAIVGMGGL	Y	235								
10484	LOC_Os11g32970	EEVKKEIFQALGYTEDHCAMNIQAR	Y	50								
10487	LOC_Os11g35510	PTLAWMRQLEESYTTSPAVAHAGLW	Y	14								
10490	LOC_Os11g37660	ALAVVAGLASNGYVASLLVSRYFRL	Y	50								
10493	LOC_Os11g39190	CPGVDLIDNQIQYHWSYYRDVVISL	Y	83								
10496	LOC_Os11g43970	AQQSSLSQRGPSYGYRGRVMLAFEE	Y	577								
10499	LOC_Os12g01190	SQQAPTNNSISVYITYAKEEEAIRC	Y	170								
10502	LOC_Os12g02540	LPDYARIVDDGLYRAIDIYLKAHPS	Y	428								
10505	LOC_Os12g04050	EEKTAFITPIGTYCYTTMPFGLKNA	Y	831								
10508	LOC_Os12g06670	DQGGNMTAETGTYRWMAPEVINHQP	Y	419								
10511	LOC_Os12g12370	LYSDNEMNIKYRYKDEELSFIPSLS	Y	227								
10514	LOC_Os12g13770	KPKLTFEMLMAKYNKGSAGQRLDNQ	Y	449								
10517	LOC_Os12g15420	LECCSQERTYLRYYGLLGQRFCMIN	Y	595								
10520	LOC_Os12g19290	DYIAPEVLLKKGYGMECDLWSLGAI	Y	342								
10523	LOC_Os12g21798	IEAHVDVKTTDNYMLRLFCIGFTKR	Y	133								
10526	LOC_Os12g24080	SLLAHDGLSDNAYLLVAEVLKKIVA	Y	370								
10529	LOC_Os12g28640	NGIVLQGIKTRVYDRLMSHDKKWVE	Y	340								
10532	LOC_Os12g32410	VWTQIGEIVFPVYTTVPILAGPSMA	Y	78								
10535	LOC_Os12g35030	RKYKLEGYTTCKYLNVVWDNEANAA	Y	211								
10538	LOC_Os12g36180	FENIFPKSQSARYSDVHVDIGASGS	Y	333								
10541	LOC_Os12g37860	YHRGGGTNRGRGYDVGKPSHVTNAE	Y	1389								
10544	LOC_Os12g39090	IAKKLGAERSEHYFHSLKKFLGGQL	Y	34								
10547	LOC_Os12g41956	TVVGTARAARAEYVEALERRGEGNG	Y	198								
10550	LOC_Os12g44140	ATKLQRVRHLSRYFKQSAKTMEQQF	Y	139								
9721	LOC_Os01g02700	KCSQEMNSSNVMYRPVACRSGNSSF	Y	158								

9724	LOC_Os01g08380	PRGGAGFYGNCYYIMRVSAPAGKVA	Y	310							
9727	LOC_Os01g08760	CHNDLQYGNIMIYEETRQVTLIDYE	Y	217							
9730	LOC_Os01g10820	YDPSTGIYGMDFYVVLERAGYRVAR	Y	130							
9733	LOC_Os01g11960	CIPGALCVMFFGYSNSGLRDYGWVK	Y	208							
9736	LOC_Os01g12660	SICLSDTMVLGRYIEEIVVSAVSYH	Y	388							
9739	LOC_Os01g16030	KLGLHSLRQRHWYIQSTCATTGEGL	Y	154							
9742	LOC_Os01g16850	GSDTVEEVTVFEYYRKNWKIDLKGS	Y	1759							
9745	LOC_Os01g19490	EWMVNTKGMNMSYTNHAIRLDLIYK	Y	124							
9748	LOC_Os01g20950	EVYEQLRQLVSTYPTVPSGLDTPYY	Y	32							
9751	LOC_Os01g21960	RFSKDNILGEGGYGVVYRGQLINGT	Y	204							
9754	LOC_Os01g23640	PTPGLAVSETGRYQNLHVPVKLMEC	Y	340							
9757	LOC_Os01g26970	AMAVSASEGVGQYDLLIVGPGVLGR	Y	94							
9760	LOC_Os01g28560	APAAQRREHERQYEALDAHTIITKL	Y	69							
9763	LOC_Os01g31629	QHHVQQQQYVDPYRTMVLSPQPDHL	Y	15							
9766	LOC_Os01g33440	INWTQTAFIPGRYILDGCVILHEVL	Y	139							
9769	LOC_Os01g34330	VLLTPEDSKEKPYVAILKDITETEG	Y	185							
9772	LOC_Os01g35730	DTHEIVVFSHFFYGGFALPTSKFFR	Y	63							
9775	LOC_Os01g37832	PENFGGDTTKIYYIGLRGEATQNKR	Y	161							
9778	LOC_Os01g40370	TNNLSQALRYGIYSNDDMEENQGTL	Y	95							
9781	LOC_Os01g40990	YGSHNFGSDTWQYCLRLRDKKLAAQ	Y	1344							
9784	LOC_Os01g43340	RERVARKLLQVDYVSTNDQVADGFT	Y	1275							
9787	LOC_Os01g43440	QSLAHLQLLDLSYNQLAGEVPPFKF	Y	219							
9790	LOC_Os01g46840	KMSMVESSSKCGYEKVGADVQKCSP	Y	481							
9793	LOC_Os01g48640	TISLVAYVENDRYVYSEKRPSCPPP	Y	219							
9796	LOC_Os01g49470	TPSCNGLSMAKYYCNICKFFDDERT	Y	1059							
9799	LOC_Os01g51200	TKHPQLLYESKIYRILQGGTGIPNV	Y	56							
9802	LOC_Os01g52304	LEWQSSIPPYYSYIVQYGILARQPL	Y	344							
9805	LOC_Os01g55040	HRLDFFRSLSVFYTTVGFYFNTMMV	Y	1358							
9808	LOC_Os01g56300	SYHMAGLSHGLPYGGSVSFGSPNLP	Y	246							
9811	LOC_Os01g58620	VNLLGLLVQSVFYYACKAFHNQQID	Y	288							
9814	LOC_Os01g59990	KTELCRFSGQKIYPGKGIRFIRADS	Y	16							
9817	LOC_Os01g61930	GFQNQYANLAPTYQPGTTYYSQLPL	Y	350							
9820	LOC_Os01g62890	YSKWTSRLKCNLYYYRTNYFILIMF	Y	52							
9823	LOC_Os01g64280	EESSPEYEKLKNYIFELENHLAEAQ	Y	192							
9826	LOC_Os01g66350	SLVTSFAFLSCGYLWSSYHEVRSVV	Y	315							
9829	LOC_Os01g67170	RRKRDRKRKRRRYDSDEDDLDQLLE	Y	446							
9832	LOC_Os01g70160	RSESTKSLSAKSYNSSFAVVTSEDE	Y	164							
9835	LOC_Os01g73300	NGLCAIAFTDDHYPVRSAFSLLNTV	Y	101							
9838	LOC_Os01g74020	LTRENVASHLQKYRLYVKRMQGLSN	Y	169							
9841	LOC_Os01g74470	YSFTNETLAKYSYATSLQATLRYGI	Y	291							
9844	LOC_Os02g01170	LKEGEENTIVNIYNLEEYVTLVVDA	Y	634							
9847	LOC_Os02g01560	QFGQKGIPYLNTYDGRTIRYPDPII	Y	142							
9850	LOC_Os02g02740	MAAAGHFPDVITYTSLMNGMCVKGD	Y	247							
9853	LOC_Os02g03080	VRVNESSTVPNMYIKALVLLEDFLA	Y	121							
9856	LOC_Os02g04450	GDNGTTIKTEASYSGNSDFGFCNES	Y	205							
9859	LOC_Os02g06430	KEDVAEKSDGKGYHVHRSRMAGGLD	Y	319							
9862	LOC_Os02g09170	EEVITGYDETDLYKTWLRANAMRSP	Y	106							
9865	LOC_Os02g11760	FLECAVWICMTYYVMGFDPNIERFF	Y	626							
9868	LOC_Os02g12550	IVNAWAIGRDPRYWDDAEEFKPKRF	Y	412							
9871	LOC_Os02g13130	SGGQQQAYDYSSYYQTQGQQQGYSQ	Y	461							
9874	LOC_Os02g13990	SDNEIVKLENFPYLNRLGTLLVNNN	Y	62							
9877	LOC_Os02g18330	RKLNTTILLLRLYIHKDLKHEYMLE	Y	82							
9880	LOC_Os02g23827	KLLHTGIIYPISYSEWVSPVQVVPK	Y	526							
9883	LOC_Os02g28020	SISEAYASPDADYWKEAVRSEMDSI	Y	648							
9886	LOC_Os02g32340	GILDDPNEEMFFYANKAVTIDQPAF	Y	261							
9889	LOC_Os02g34210	DRHAQSYDVQLQYDTDFGEDRTEAD	Y	44							
9892	LOC_Os02g34680	SAAPPTPSADRPYVCPYDGCAKAYI	Y	221							
9895	LOC_Os02g35800	NTIPSIPAHAVIYAFRGGVRHNRML	Y	358							
9898	LOC_Os02g38840	FGTEGRGGYFDQYGIIRDIIQNHLL	Y	263							
9901	LOC_Os02g41990	GLARKEDYPHITYYCPHCHALNTSK	Y	320							
9904	LOC_Os02g43370	DIGNPDGYWKAPYALIFRNMAILGV	Y	543							
9907	LOC_Os02g44690	KHWIGLAVTSAAYFLPYKQLANMSE	Y	58							
9910	LOC_Os02g44820	LPAAAKERLRAVYFVHPGFQARLFF	Y	145							
9913	LOC_Os02g45070	QLGEGLESWRGFYQSIRPTQMGLSL	Y	394							
9916	LOC_Os02g46962	KKRIMLKDARVQYLSLVLLETIVKN	Y	102							
9919	LOC_Os02g48180	YKKDGYNDFNTFYMQVRKFISYQIL	Y	196							
9922	LOC_Os02g50910	MRKSIRASHSLHYSKNSFLWSDDMD	Y	258							
9925	LOC_Os02g52390	KGSLHTFLNAFTYPDRTCYPVASTN	Y	196							
9928	LOC_Os02g53270	DNEELLLYLLKAYGERDSTQNLLDK	Y	363							
9931	LOC_Os02g54770	QYRKSPTYGRRSYSPRDRSPRRRSV	Y	139							
9934	LOC_Os02g55330	ELRAHLRVNIMSYDYSGYGASTGKP	Y	68							
9937	LOC_Os02g55420	VQRGLEVFVAQSYSKNLGLYAERIG	Y	301							
9940	LOC_Os02g56740	PTLQLHNNMEDCYIDICSPEVLSLF	Y	236							
9943	LOC_Os02g57700	AVIEAENLRHEAYEETRRRQKVERD	Y	351							
9946	LOC_Os03g01710	QDEVSKEFDECQYKIVVSVGSIKSG	Y	88							

9949	LOC_Os03g03060	FTIMHDVEKEYGYEISYDKAWRAKQ	Y	366							
9952	LOC_Os03g04050	VSESGEKAPNFVYRGRLQRTRRAIA	Y	86							
9955	LOC_Os03g04570	GRYWTFVAGSAIYFLGMCLLTLAVT	Y	122							
9958	LOC_Os03g04960	DKALEIASDRVYYIYQTLYDCEEVL	Y	326							
9961	LOC_Os03g06540	EGDPLGPNDATHYRSVVGALQYLTL	Y	1029							
9964	LOC_Os03g07580	DFDYIQEDRLKIYELLSGNVQGALV	Y	610							
9967	LOC_Os03g08360	DELWRKVWGEATYDLATVLAFLAVL	Y	95							
9970	LOC_Os03g10460	SFLEENYSKAKFYLRLKRRNGRVKH	Y	248							
9973	LOC_Os03g11220	KEGSAGSGGGGKYQDEGGEGKYKKK	Y	114							
9976	LOC_Os03g13614	STGPVVLNPMMPYWPVPPPMAGPAT	Y	241							
9979	LOC_Os03g14260	DMLDAKYWEINMYKSALEGRKDEIG	Y	333							
9982	LOC_Os03g15750	DTMQYFEGWPNQYMTSSLMLFYMCQ	Y	103							
9985	LOC_Os03g16310	SIDDGLARFLGGYKLDKFAFKPARG	Y	57							
9988	LOC_Os03g16800	NGNGYGYGGYDDYRERNGNNNTDDK	Y	209							
9991	LOC_Os03g18454	AAAAGEKVPLALYYETLCPYCSRFI	Y	44							
9994	LOC_Os03g19530	KLGGFSIPRPTSYSFERSQPPQRLY	Y	18							
9997	LOC_Os03g20080	YKKGQVSIFLLIYVDDIIVASSVPD	Y	1024							
10000	LOC_Os03g21540	VVKGSFGYLDPEYFRRQQLTDKSDV	Y	713							
10003	LOC_Os03g22320	EGLLTISDAKENYKIPSQADLIRQA	Y	687							
10006	LOC_Os03g23970	VMNAVGVCGLQLYRYGETISIPFFT	Y	130							
10009	LOC_Os03g24050	PSAAIAGFQRRLYGGHRRPDVPLAG	Y	45							
10012	LOC_Os03g25070	DFEEFSAAAISVYQMEGLETWEQHA	Y	521							
10015	LOC_Os03g27140	ARVGFTSELFGDYCEDMGIKLCFTS	Y	1636							
10018	LOC_Os03g29035	YGVHLRGLRGAAYILGIRIYRERSK	Y	500							
10021	LOC_Os03g29260	VFTSLPSAPAASYVNVTRWYDHISA	Y	51							
10024	LOC_Os03g39380	RHLPSLRRTFSLYDQINLIDSVPED	Y	43							
10027	LOC_Os03g40180	VMRFVQRVRCWEYRQQPAIVRLTRP	Y	30							
10030	LOC_Os03g44020	AGVDWWAYGVFLYELLYGRTPFVGA	Y	348							
10033	LOC_Os03g44900	GRPEISADQREKYLQRLQQVQQQGS	Y	534							
10036	LOC_Os03g45480	PIQLAIDKAWAQYVQRGGLRKTGHD	Y	311							
10039	LOC_Os03g46600	PLAPHGVESLDLYQCPVTKAKGYRD	Y	137							
10042	LOC_Os03g47890	PGPEMMMVDLRDYGSGVGTLAVRMD	Y	49							
10045	LOC_Os03g48674	VWTQVGEIVFPVYTTVPISAGPSMT	Y	37							
10048	LOC_Os03g49800	ILYFGIIDILQDYDITKRLEHAYKS	Y	687							
10051	LOC_Os03g51360	EDRPRRKVQRPVYFVSEALRDAKTR	Y	616							
10054	LOC_Os03g52490	GNNQGGYNQGGGYYDNQGGYGGYDN	Y	201							
10057	LOC_Os03g56241	GHSNVWNSHPKNYGPGSRVCRVCGN	Y	14							
10060	LOC_Os03g56800	APRLFSQGVSYTYDDVIFLPGYIGF	Y	27							
10063	LOC_Os03g58590	NLDSQVYWWHDKYRPRKPKYFNRVH	Y	552							
10066	LOC_Os03g60870	AGQERYRAITSAYYRGAVGALLVYD	Y	81							
10069	LOC_Os03g62700	RVLGTFGYHAPEYAMTGQLTQKSDV	Y	248							
10072	LOC_Os03g63590	ATKAIYRLLLSDYVKVSKVSVEDML	Y	129							
10075	LOC_Os03g63670	QPQPSGYSIDPRYQPSFSGVLPGTS	Y	382							
10078	LOC_Os04g01480	LEGILESPVINGYRNKCEFSVGFSL	Y	315							
10081	LOC_Os04g02320	HAADLVQLRPTAYHETSPMQLVRGN	Y	204							
10084	LOC_Os04g02500	KLAHEFESHLSSYDHNHRKRFKEMK	Y	266							
10087	LOC_Os04g04220	RYNVMTTNLAELYNWVMPDTRALPF	Y	46							
10090	LOC_Os04g08410	VWPLENGNRKLSYFCNPVGKGREGV	Y	373							
10093	LOC_Os04g09860	IDMLMLNKLKESYSQIRSGSFDAVS	Y	362							
10096	LOC_Os04g18010	SCRGIWTVYYKSYRGQMAEDNEYHA	Y	54							
10099	LOC_Os04g22180	YIVVDFKDLFDLYRLRAVDTSLLKC	Y	183							
10102	LOC_Os04g23060	FEARSTDGLFLGYPAHTRGYRVLIL	Y	357							
10105	LOC_Os04g27290	MLVTISPEFQRQYQTLDAHTIITRL	Y	93							
10108	LOC_Os04g28770	WYLRVKDRLKRLYSNRDDAKLMRWH	Y	252							
10111	LOC_Os04g31260	FWEDKWLGYHLSYPVATYLGYPPPT	Y	69							
10114	LOC_Os04g32650	IIDMTGNTIGKRYARTDEIGVPLAI	Y	598							
10117	LOC_Os04g35260	SDFLSGKDRPRWYDGRREVHIHPSS	Y	1311							
10120	LOC_Os04g37640	SNTTSDAPSECRYLVWTPHAGLGNR	Y	184							
10123	LOC_Os04g38570	VLFATSIFENIAYGKDGATEEEVIE	Y	1115							
10126	LOC_Os04g41100	GLSRQYGSPLKPYTQLVVTLWYRAP	Y	521							
10129	LOC_Os04g43300	CMEAKEPVPEEPYEISSDVHGSFDG	Y	503							
10132	LOC_Os04g44740	QNLSNLLRLALLYKYGGVYLDADVV	Y	284							
10135	LOC_Os04g45580	VELRRLSFLKDTYSNGAIASIPNTS	Y	822							
10138	LOC_Os04g47870	VNRAVMFELVTLYRYSHLGGRLPAY	Y	301							
10141	LOC_Os04g50890	EGDSKLFFLATIYALLGIPLSYLIW	Y	166							
10144	LOC_Os04g51700	GADLDVIIIGGYYGSGRRGGEVAQF	Y	451							
10147	LOC_Os04g52850	NQLSLYSRETTQYNYQELRQMTANY	Y	204							
10150	LOC_Os04g52940	RLIGADETMYSGYVDSMQWLDDIKV	Y	181							
10153	LOC_Os04g55960	GDGEPTDNAARFYKWFTEGKEKEVW	Y	161							
10156	LOC_Os04g56640	MATSSAYPPPPPYYRLYKDYEKDPS	Y	13							
10159	LOC_Os05g01040	GYCISNNQRLLVYDFVPNNTLHYHL	Y	141							
10162	LOC_Os05g02240	AEFRQRMGAMVRYEERVVVGNVIEE	Y	737							
10165	LOC_Os05g03760	MYSFKVKPCSRAYSHDWTECPFVHP	Y	227							
10168	LOC_Os05g07000	EERLHSRRDGRGYSRSPRRHDSPSN	Y	201							
10171	LOC_Os05g08370	LLERLAYINTIVYPITSIPLIAYCV	Y	854							

10174	LOC_Os05g11710	MQLLESGLKVKEYELLRRNFSETGC	Y	91								
10177	LOC_Os05g14110	KRKAQAEVLASEYERPPKHPDPQGS	Y	531								
10180	LOC_Os05g16910	ITDTFAPQPRSAYNSMAEFVEHVHV	Y	1342								
10183	LOC_Os05g22990	LIHCCCVMLTSYYPVQVNVPVRALI	Y	333								
10186	LOC_Os05g23860	GRKSDMYVFCCSYTHNVAPKGKFIA	Y	340								
10189	LOC_Os05g24460	ADQDQLRRDDLDYLEERRRRAALRA	Y	1874								
10192	LOC_Os05g30420	YIGRLIEKHGDDYKAMFMDIKLNTM	Y	157								
10195	LOC_Os05g33100	LRKIADKVASSGYFVVVPDFLHGDP	Y	69								
10198	LOC_Os05g34900	NGHKSGANGTFEYPYSAYRDKDAPG	Y	260								
10201	LOC_Os05g38830	CPFLFSFEARWKYFQLTAFGSLTPQ	Y	94								
10204	LOC_Os05g41790	SQHEIYRTLVHAYGEDNRGLTHPVV	Y	197								
10207	LOC_Os05g43570	DYIAPEVLLKKGYGMECDWWSLGAI	Y	337								
10210	LOC_Os05g44922	VIRELVCGLAHMYNVSKIYGIQNGY	Y	193								
10213	LOC_Os05g45660	NEFLKPAEGERYYGGRGRGRGRGDR	Y	331								
10216	LOC_Os05g46620	AAFGEAMLTTIGYIYVRQAARELGK	Y	186								
10219	LOC_Os05g49570	TAGCDLLCFLDAYSGYHQIRMARED	Y	876								
10222	LOC_Os05g51070	FAPLSARPNAGGYRAPELVDARRPT	Y	575								
10225	LOC_Os05g51490	VKRSIDLFFQYPYNNFLHHHVESII	Y	374								
10228	LOC_Os06g01700	QGGTRIFGPSQKYSSRDLAAHTTLK	Y	30								
10231	LOC_Os06g02370	REAYRREPAAPAYVMPEEPPAMVEL	Y	85								
10234	LOC_Os06g04330	VSRNSRGYFALGYSLPGEPFPSLHE	Y	46								
10237	LOC_Os06g05040	LRLIAWIAEIQAYLTDKTLPEDREG	Y	1534								
10240	LOC_Os06g05740	KVATAARKEDIPYIRCQVCERIARE	Y	47								
10243	LOC_Os06g07350	YYHGQYPPYYPPYGGYMPPPRMPYP	Y	425								
10246	LOC_Os06g10170	PLRWLLSKLAETYFKMQIPMEKHGM	Y	302								
10249	LOC_Os06g12580	RKPQQEESYGSEYGSGYGRKPQAES	Y	223								
10252	LOC_Os06g13390	NETNHEGEIVPAYYICGVPGSYYTR	Y	101								
10255	LOC_Os06g15420	VLRNAFDDEEKPYLPKHILYRQKEQ	Y	440								
10258	LOC_Os06g17290	AVSALNRALSSEYPSKSRSEGRASG	Y	76								
10261	LOC_Os06g20390	VTPPAIAPPASQYKYDSSYQPEVEK	Y	434								
10264	LOC_Os06g21350	LAYLISCRELRCYYKANVDGFSATD	Y	19								
10267	LOC_Os06g24810	EGRRDRERRLLVYGDGSTPQGALQA	Y	72								
10270	LOC_Os06g28140	NRVPSKSVDKTPYEIWTGKRPSLSF	Y	407								
10273	LOC_Os06g30570	PWKIGFKEDIHTYRSRKRSKRDTEA	Y	380								
10276	LOC_Os06g33520	GLLTDHISENVDYKVIVFCTTAKVT	Y	714								
10279	LOC_Os06g36710	SLSYGQGSSSSGYGMGLPPGRDYAS	Y	253								
10282	LOC_Os06g38120	FLSPQATGRQAIYIDISMFLAFSSF	Y	130								
10285	LOC_Os06g41730	WVKINVRRTKDCYEVYALVPGLLRE	Y	384								
10288	LOC_Os06g43790	NVCCYESMQAGQYRLKHLGIEKLTQ	Y	987								
10291	LOC_Os06g45910	CKLNPVTQDEDLYTIFSRFGTVTSA	Y	259								
10294	LOC_Os06g46890	SKTKKLIKAHARYVKAQDDLKRSQA	Y	219								
10297	LOC_Os06g50742	GVAKLDPEKNTLYSMRCYLATGISK	Y	455								
10300	LOC_Os06g51250	PWPKITQADVRKYMIVFIKVDRDRD	Y	384								
10303	LOC_Os07g01110	LLEASRSRSSGVYTRDFPDRPPVMF	Y	423								
10306	LOC_Os07g04240	GNMTEDDWRWHMYDTVKGSDWLGDQ	Y	108								
10309	LOC_Os07g06190	MSLISISTASLQYNFYGSSCPNAEQ	Y	24								
10312	LOC_Os07g09060	LDDAIQIVNRNKYGNGASIFTTSGV	Y	444								
10315	LOC_Os07g10350	EARIILSNLSDEYVENPQNDFPVGL	Y	377								
10318	LOC_Os07g12170	GGQDKIRPLWRHYFQNTQGLIFVVD	Y	204								
10321	LOC_Os07g15620	PKANGKLRMCIDYTDLNKACPKDPY	Y	749								
10324	LOC_Os07g27790	FDDSFGFERYVDYALDVPMYFVYRN	Y	305								
10327	LOC_Os07g29360	GPEIDEYYADVKYERIAILNALGAF	Y	80								
10330	LOC_Os07g30040	EHRERVAIQATDYQQALRRYHEKRI	Y	1193								
10333	LOC_Os07g30820	VGPPASYGLFSSYGQPGPMGSPMGG	Y	142								
10336	LOC_Os07g32220	LGTAGYAEDQRVYEAIEYGFPDYVW	Y	182								
10339	LOC_Os07g36600	ESAYAVRWAVANYLRPGDAVILLHV	Y	79								
10342	LOC_Os07g37810	HESGLLSDRSDVYSFGVVMLELLTG	Y	680								
10345	LOC_Os07g41110	VVNEADKCVYYRYGGGEGVILCLYV	Y	1594								
10348	LOC_Os07g43030	DQNIEKLVKEKLYCWLVHKVHDEDK	Y	610								
10351	LOC_Os07g44190	SGHSPLKRPIAEYLRYGVINLDKPS	Y	100								
10354	LOC_Os07g45780	WTNGNQHTTIRLYAAKVMAEFAKSL	Y	318								
10357	LOC_Os07g47350	ADDNGEGGTFALYSLLCRHAKFSLL	Y	129								
10360	LOC_Os07g48410	GADGLGWKPCLYYARGYCKNGSACR	Y	238								
10363	LOC_Os08g02470	YFQAHRVTVVTSYPLGQILHNREGT	Y	1232								
10366	LOC_Os08g03970	AKTRYPQAQKMLYAILMASRKLRHY	Y	1275								
10369	LOC_Os08g07480	IGVEEEEDIRCAYGIRHLFAGDEEK	Y	748								
10372	LOC_Os08g09160	EGRRDGERRLLVYGDGSTPQGALQA	Y	72								
10375	LOC_Os08g14230	SKFYTGDCYIFQYMYPGDDKEECLI	Y	428								
10378	LOC_Os08g16930	KSMSENPSQTFVYRVKNPALPPTLR	Y	101								
10381	LOC_Os08g21760	GGYYIVTYALGIYILNLLIAFLSPQ	Y	89								
10384	LOC_Os08g27420	LQPIRDAETGRVYLPPACHTLSKDE	Y	313								
10387	LOC_Os08g29124	HSGHLRADAVKQYLLSGESLYQRSS	Y	221								
10390	LOC_Os08g31350	RWRQWKSDMNTNYVKKNKIPFEEWG	Y	229								
10393	LOC_Os08g38310	DEDSSTSEASRKYMDVGRLDLPTTT	Y	922								
10396	LOC_Os08g41020	KTEKGLLLTQERYAGDIIKRTGMSN	Y	1172								

10399	LOC_Os08g42980	GGGGAYHHHQQHYQPHHHHQHHQHW	Y	70							
10402	LOC_Os08g43560	DKALLEDPSFRRYVDLYARDEDTFF	Y	217							
10405	LOC_Os09g02700	EMNSKMVGSKPLYVALAQRKEDRKA	Y	393							
10408	LOC_Os09g06560	FLLSLFLKISLPYLRHCGREIRCIF	Y	26							
10411	LOC_Os09g09900	IYDTLWSRRDKEYIMCAYNQYAHWI	Y	334							
10414	LOC_Os09g14670	VEYFRLATPETEYGRMNIGSRPSKR	Y	755							
10417	LOC_Os09g16380	LLDVLAGRKTSGYIEGNITISGYPK	Y	855							
10420	LOC_Os09g20460	SSSGFSLHGGSMYGFQELGSVGTSL	Y	56							
10423	LOC_Os09g24260	MSGTEAAKNKAAYTREGRRQLFAVL	Y	400							
10426	LOC_Os09g33600	LAYAQQSRERSNYEQAALVERMQEF	Y	232							
10429	LOC_Os09g36160	FQLWQQQEQQPFYASNIIRFADDAP	Y	52							
10432	LOC_Os09g37520	RSELLALAIRHRYALADTHRAYAES	Y	32							
10435	LOC_Os09g39900	CDLLCFLDAYSGYHQIRMAREDEEK	Y	906							
10438	LOC_Os10g15170	PTRLKEVQKLTGYMAALSRFVARMG	Y	195							
10441	LOC_Os10g21920	RTTVSFNTLIAAYCRDGVDAGPALQ	Y	260							
10444	LOC_Os10g25220	QLEKLKVYECKAYLRMHKLRLSGNK	Y	143							
10447	LOC_Os10g26140	LQLDRVSMTLNLYRFYRDAVVQQGT	Y	241							
10450	LOC_Os10g31770	RYFVLETRLLSYYKRKPQHKMPKLP	Y	42							
10453	LOC_Os10g35150	MSVAWYPIYHIPYQRNVKDLSACFL	Y	216							
10456	LOC_Os10g39140	EAISESLGLEGGYMRETLGEQEQHM	Y	185							
10459	LOC_Os11g02080	GLWATAVGASVAYGRRKTPQMRLIH	Y	34							
10462	LOC_Os11g04670	HESGIHQDGMLKYKGTYEIISPDDI	Y	404							
10465	LOC_Os11g07500	MSEMIRPERALLYLVPGRLVKVRDG	Y	608							
10468	LOC_Os11g10480	IYHFVGTSTFSEYTVMHVGCVAKIN	Y	152							
10471	LOC_Os11g12810	LGRMPRAEIQGTYKIARRIEAEETG	Y	416							
10474	LOC_Os11g19060	VVSSHFTNGVGNYGSQHYGYNGWSP	Y	387							
10477	LOC_Os11g23750	IFYFHAPMMNGLYIVNLDGCSVYNI	Y	297							
10480	LOC_Os11g29970	FTPKEVRYENLQYQYMQQPKYPIVP	Y	746							
10483	LOC_Os11g32470	VMFLPLPMISGYYAARFFTKKSLSS	Y	438							
10486	LOC_Os11g35274	QVTHKNIVKVIGYCIGKKSLMMVTE	Y	86							
10489	LOC_Os11g36480	DFSDICPKEVSCYNCAQPGHTGLGC	Y	43							
10492	LOC_Os11g38130	PYHPQRLRLFLYYRVTLSCSPSAGS	Y	176							
10495	LOC_Os11g40140	GSEGADSSLQEQYNKELTLQEAETI	Y	183							
10498	LOC_Os11g48090	QISALIDRQEELYERESQLKAMLEV	Y	36							
10501	LOC_Os12g01940	DDSNAEDNPLYDYPEELSEDEDDDS	Y	288							
10504	LOC_Os12g04000	SSLLGVIWSLVSYRWGIEMPAIIAR	Y	261							
10507	LOC_Os12g06360	LRRYRLMLNPEKYTFGVPSGKLLGF	Y	148							
10510	LOC_Os12g07190	LASETGDGKSSIYPPVIGNSFAFKL	Y	402							
10513	LOC_Os12g13340	ASFTDDEEEEAPYPYRSDGNLRAGS	Y	209							
10516	LOC_Os12g14920	GRGLASISTRREYSLSPWQQRGGGD	Y	229							
10519	LOC_Os12g18110	VQSIDQHTLVNFYGNIGKTLLSLEV	Y	216							
10522	LOC_Os12g21710	GVWNGDRRLASRYGEAFEVLKKRTS	Y	302							
10525	LOC_Os12g22000	KKQFPLGKYLLGYLYQTLSTAVTKI	Y	133							
10528	LOC_Os12g25690	ANYWKQVIKINDYQKSRFVNRVVSS	Y	305							
10531	LOC_Os12g31040	LLEQGTSIQVFSYVETTETLRRAIS	Y	413							
10534	LOC_Os12g34510	AGLQFPVGRIARYLKAGKYAERVGA	Y	37							
10537	LOC_Os12g35290	YHRTHLFDVVNQYRAIFNNDKSGSD	Y	282							
10540	LOC_Os12g37840	TVIRDMPNVPLLYIIGAFIPATMIA	Y	293							
10543	LOC_Os12g38430	VPFERFGPVRDVYLPKDYYSGEPRG	Y	67							
10546	LOC_Os12g41290	KTAPGYCEKAGVYVSHLSQTSLRAV	Y	128							
10549	LOC_Os12g42884	GFAFTANGWVQSYGSRCVKPPIIYG	Y	519							
10552	LOC_Osp1g00740	VMILNLMPYRASYPILKLVYSAAAN	Y	80							

Table S3. Filtered negative dataset.

No.	Protein Name	Sequence	Amino acid	Position
4976	LOC_Os01g03520	ASLDDGTASDGASWETVDDNQTDLP	S	667
5029	LOC_Os01g13530	EKSSSKGRGFLKSLLTRRRWRNDES	S	297
5037	LOC_Os01g15039	TVSEAVARAPSHSRRRYVIYVKRGV	S	329
5043	LOC_Os01g16110	KSLMDEHGWVPISKVADFNRLKKMT	S	364
5087	LOC_Os01g34410	PPGGFLSYFQDPSILQNHRPFVPPN	S	102
5104	LOC_Os01g39110	HHHHHHHHQLLFSPPPVMAHHQELA	S	256
5112	LOC_Os01g40480	PYAAYTRSKVYQSPKLWYLRASVIE	S	493
5142	LOC_Os01g43170	FPGYGSPQFPPGSAQAQMHQQHPVQ	S	433
5199	LOC_Os01g53140	GVYLRNSQTKANSYWRRHKQIRRSF	S	56
5219	LOC_Os01g55420	PSRRQPLLRPGKSTAFKREERRKRK	S	28
5242	LOC_Os01g58550	TLSGVQVGADWTSILKRRHVYREAF	S	251
5243	LOC_Os01g58550	VNNKPLSPSYKYSRKIPVKTSKSES	S	333
5273	LOC_Os01g63950	SAYDAPPPHHEDSDGDDTDEAAAED	S	51
5282	LOC_Os01g64790	NALVYPATAASWSESSYHHHPPPPH	S	337
5287	LOC_Os01g65560	EALVKTRVEDGFSLKYIPWHIVNKI	S	91
5309	LOC_Os01g68860	PLRPGEELCKFYSRYGICKFGANCK	S	388
5353	LOC_Os01g74020	VPSPYHPHPHHHSYNNAAYAATVSS	S	217
5373	LOC_Os02g03040	ALRDDDEKRNGYSPERRGRDRSPDR	S	176
5405	LOC_Os02g07370	LLRHPPVVPDPESPVQRWLDDVANL	S	100
5487	LOC_Os02g24330	EEEKGEMQPKSRSSNPFGAARPREV	S	295
5496	LOC_Os02g26349	MMKMMGIPVGFDSTKGKHVPDADVS	S	157
5548	LOC_Os02g35690	RHPQWQEVAEVVSSRDGYSKQPKSD	S	101
5581	LOC_Os02g40690	PLRPRHPAPPLPSPQWSRKNPSHPA	S	38
5582	LOC_Os02g40690	QWSRKNPSHPAASAADDDMDDDAIT	S	52
5628	LOC_Os02g48730	IKDQLEKDKDDESLRRWKEQLLGSV	S	218
5630	LOC_Os02g48910	AWTQKHSSTPIVSHYPPYAVIGSTP	S	174
5719	LOC_Os03g01490	EYVPEFNLWFGFSSDESPHYLAASN	S	290
5721	LOC_Os03g02010	FGGRKKTRFVDGSKKRKRYGSGPSG	S	256
5735	LOC_Os03g02480	AIFFYWVTSNLFSLGYGFVLRKPAV	S	358
5745	LOC_Os03g04330	NRRSHKPHASDASFPLTWIGATPSI	S	124
5761	LOC_Os03g06340	AISSDMDELDSDSRKRASLKSKREF	S	451
5762	LOC_Os03g06340	LVMHAYNPPNVDSFIDHLGLHKALC	S	271
5767	LOC_Os03g07380	KNGALPDYSMDFSKIREMRKQNKKE	S	43
5781	LOC_Os03g10180	LESSIANNKFHFSFYKWGGKGAVLV	S	224
5788	LOC_Os03g10740	TRGVGGKPYRNDSSQKDATRIKAGI	S	21
5829	LOC_Os03g14860	AAAAAASDDGEYSARAPAPQFVTEF	S	39
5851	LOC_Os03g16690	GETYEMVNHQGISFIAEQVSHHPPM	S	168
5856	LOC_Os03g17490	AKKKARLERFGQSTNVDKGEEEKRK	S	130
5874	LOC_Os03g20340	SGKDHSLLLILPSGVYRYRFVVDGE	S	155
5879	LOC_Os03g20600	QACVCAPTTHPGSFRCKHHRQNASN	S	71
5906	LOC_Os03g24890	ELNHQVIGGREISIVFAEENRKTPQ	S	133

5918	LOC_Os03g26460	KLNLKPEGQFIFSAKGPADDTPYEL	S	128
5937	LOC_Os03g30860	APQSVGNNKYYVSFIDDYSKFTWIY	S	536
5940	LOC_Os03g31750	KQLHLSVLAVFNSWDSARAKKYRSI	S	228
5975	LOC_Os03g45300	SKVGPGNSEWLYSVYNCRQHWVPVY	S	446
5991	LOC_Os03g47800	TQRHRGFGFVTFSDPEAVDSAIKEM	S	56
6005	LOC_Os03g50340	NAVLICSDVDGGSYELYIVPKDSAG	S	390
6028	LOC_Os03g52490	VPLEMTRHVSMISITLSPRDLDKNS	S	109
6060	LOC_Os03g57854	DYSKPHSIHVPESDIGYHFGTLLDN	S	202
6108	LOC_Os04g02000	WMCSNCNNHNYASRAFCNRCKTQKE	S	323
6123	LOC_Os04g08440	LTDKTLSEDCEGSERVQRISKRYVL	S	1548
6137	LOC_Os04g14640	CKLLKFTSEEGSSAFFLWRSDCSFL	S	97
6140	LOC_Os04g16360	PVGIHQGRNIQVSYEFYHPMSSARQ	S	153
6146	LOC_Os04g18090	AIAKYMEEKHGASLPANYKKMLSIQ	S	70
6166	LOC_Os04g24700	FLLQIDPKYPDESRYVMHDLIHDMA	S	478
6170	LOC_Os04g26660	TLHQHYPDELQHSPYRYHPRRGGAR	S	183
6183	LOC_Os04g31260	FFRFGSFKIRDSSQVRFWEDKWLGY	S	53
6201	LOC_Os04g35090	LPSEIVPATLKKSARPPGRPFGSGP	S	94
6222	LOC_Os04g38290	EEIRLFDGAISESRDLRRYVIAVME	S	415
6232	LOC_Os04g39090	SARSMDDLLKLDSSREAYSSWSGNL	S	183
6243	LOC_Os04g40430	PLNAFKPHPEHKSKLICNITGDIIN	S	98
6370	LOC_Os04g57460	RRARTPPPPPRQSPRREKALERAEG	S	37
6382	LOC_Os04g59040	ADDPEYRHYNDISELSPHRLQEIKR	S	156
6395	LOC_Os05g01540	VRRGRSSSSYSDSPTPPRRGPRRVL	S	384
6442	LOC_Os05g07000	SPRRRRSPSYGKSGPPSHWGSHGAD	S	175
6457	LOC_Os05g08970	KRKPKGGDAAEGSEKRKPKKKKDPN	S	544
6485	LOC_Os05g20230	EAHESPYSIHPGSTKMYLDLKEKYW	S	1316
6493	LOC_Os05g24160	INNTSYGSLPGFSNRKAAEQSAAEV	S	85
6495	LOC_Os05g24430	HRDGLLHSFDFKSFETCESCLLGKM	S	427
6521	LOC_Os05g32610	LSKLVFSTEDEFSNVRNMLSKAEME	S	1384
6524	LOC_Os05g32960	ILLFRSFEGLKFSSLRFKYHAEMRR	S	249
6533	LOC_Os05g34540	LYAESLARFQGGSPYIYPLYGLGEL	S	222
6571	LOC_Os05g40960	LQNHGVLKAYDDSTLFCRKQSQDQE	S	277
6577	LOC_Os05g41500	GGSSFSFHARSFSGVETTPKFGSFN	S	95
6578	LOC_Os05g41500	SFSGVETTPKFGSFNPADDLLVAFQ	S	105
6582	LOC_Os05g41770	PRRKVQRPVYFVSEALRDAKTRYPQ	S	619
6600	LOC_Os05g43730	VDGGACAHVSRRSLKAMKRELLAAA	S	550
6601	LOC_Os05g43950	SDALALYRLEYKSSDTKRSVKSKLA	S	497
6636	LOC_Os05g49150	VDDSLAAAAEDSSFRLVDAKPPPRH	S	89
6656	LOC_Os06g01320	LDDFVRQRFGFESYERVERGLLVPK	S	851
6659	LOC_Os06g01680	EKAVDDLNYRLQSLVPGDSEHVQVL	S	803
6683	LOC_Os06g06530	GDNPAKVKEFVKSYNALHEMGFTSS	S	195
6701	LOC_Os06g09930	LCYLLFRELRKFSFKLVYFLAVSDM	S	53
6728	LOC_Os06g16390	DESICRQEAHGRSWKVIEQGLLLKG	S	535

6751	LOC_Os06g22700	RTHCNNCNKYRYSREVCEPGHSPHR	S	114
6789	LOC_Os06g30570	ASGKAAKNYVRHSGWVVRDNVPVST	S	133
6807	LOC_Os06g34710	EIRNEVSYDDGFSGKMGSLHGMTST	S	551
6810	LOC_Os06g35800	WQWRVVEVASTGSWRKFVSKVREKG	S	116
6829	LOC_Os06g37770	SGAGRPVGGEEASELRRERRSDSFR	S	31
6871	LOC_Os06g45360	PVVHSYSIVTLHSAFFYVDKRKVSV	S	231
6879	LOC_Os06g46890	HRPRSRDRGSSHSRSPIRKRHRKKL	S	103
6886	LOC_Os06g48530	HRGGGGGRGGGSSSPPPYRSGRRHS	S	94
6903	LOC_Os06g50840	GLDGIRMLDPNTSRTLRIYPLETVT	S	229
6904	LOC_Os06g50890	VNHGNTLYVTGLSSRVTERELKDYF	S	73
6906	LOC_Os06g51220	YNKAIAAYNKGESTAKKAPAKEEEE	S	115
6921	LOC_Os07g03180	LLLVLALSVILVSATAFHHHHHHDG	S	25
6926	LOC_Os07g04530	GDTRKLERWSDDSSKYSVDGRRPPQ	S	134
6961	LOC_Os07g14270	YSIMFGPDICGYSTKKVHTIFTKND	S	150
6984	LOC_Os07g25810	AKGDDNAAGEEKSLWLKKQFGKGLG	S	42
7020	LOC_Os07g37610	ITDQELQHLSGDSNSVITPLFEKML	S	445
7081	LOC_Os07g47920	QGGNLNYQYQPQSPHVQYQQAGSAQ	S	232
7088	LOC_Os07g49270	LNVDLLDVHADKSTFHRFDKFNLKY	S	431
7091	LOC_Os08g01054	DIKDGSCDIRDSSLAAVKRKKPKDL	S	184
7101	LOC_Os08g01930	PPNYPPGPGNYNSYGPSQGPNYGQP	S	433
7105	LOC_Os08g02354	GWQRMWWHYAQRSVLADVRRELRKT	S	336
7113	LOC_Os08g03390	QAAPSQAELLFKSFKIKKEKLKSEN	S	327
7115	LOC_Os08g03520	PDDGGEDLFVHQSSLKSDGYRSLND	S	36
7125	LOC_Os08g06070	NDREKQIKAIEDSFRAAKSRPVHQT	S	223
7145	LOC_Os08g12760	DGQPYLPLHFPFSSPYYQPPASPSM	S	149
7150	LOC_Os08g14460	LQQRQRDIEFRESTNDLRQRMQSDI	S	128
7177	LOC_Os08g23930	AYCSVLRAFKAQSDAISWEKEGLIT	S	75
7183	LOC_Os08g25080	MDESQASDEARYSGKNGGGTDLNLN	S	161
7197	LOC_Os08g28670	LKEVYDPYHVTFSFAPVPGKEGEQC	S	100
7259	LOC_Os08g42740	RKLTCGICFEGYSSDVMSSADCDHF	S	163
7287	LOC_Os09g07690	CKLLRYLNWKNGSKLWRMTKRDSAL	S	587
7336	LOC_Os09g23730	FSKNNYFRGDDPSLPPKRGRGRPPK	S	94
7361	LOC_Os09g29840	IQGLVKNYSQFVSFPIYTWQEKSRT	S	289
7386	LOC_Os09g33970	RKEAFSSDKNEYSHLLVSDFSDTTP	S	549
7426	LOC_Os09g38580	LIGNMQTYLQSISKRLEEWRLRQRD	S	406
7432	LOC_Os09g39540	VLFDQATYDKLLSEVPKYKQITPSV	S	53
7439	LOC_Os10g02584	PAERDTKQLLSGSPFLHHQHQQHVP	S	43
7444	LOC_Os10g06130	KRDFGFVHFAERSSALKAVKGSEKY	S	338
7448	LOC_Os10g07270	HYSAYAKLVEEDSWYHFDDSHVSSV	S	878
7492	LOC_Os10g26540	EWDMKNGAVPDYSMDFSKIREMRKQ	S	38
7509	LOC_Os10g33230	ESDQGKLFIGGISWETTEEKLRDHF	S	14
7557	LOC_Os10g41400	NRQGNDVGTQYRSGIYYYTPEQEKA	S	190
7562	LOC_Os10g41960	FSASGNISVSGVSWPIFRWGGGKDD	S	321

7586	LOC_Os11g03890	NQGGFFDSEYRHSPPPRPKKPAEDK	S	190
7589	LOC_Os11g04890	RILCLHTNQSMHSIRLDKKKRVRRE	S	251
7590	LOC_Os11g04930	AAEQKTPDPVPGSEICYFKNGVCQG	S	328
7596	LOC_Os11g06170	AGHKRSGSMDGESSLFEGESAPPDY	S	131
7644	LOC_Os11g19060	AAQNLHNLHHFSSLGYTHNFFQQSD	S	441
7658	LOC_Os11g29380	RLINEMVLANKCSLEIDYKQFIYIH	S	280
7672	LOC_Os11g33340	PVVHSYSIVTLHSAFFYVDNRKVSV	S	264
7694	LOC_Os11g37990	RNLLSKRKALARSIEIQRREALSHH	S	704
7712	LOC_Os11g42000	EKNGLGSYEVNFSYIEAGEQKQALV	S	150
7749	LOC_Os12g03710	NQGGFFDSEYRHSPPPPRPKKPAED	S	142
7801	LOC_Os12g15730	PVEESNLPFKKHSIFFRYLNYWKDL	S	97
7819	LOC_Os12g22620	EDALDLGALAGASFLPLQRRSMKRE	S	176
7827	LOC_Os12g24580	QAANGSARAAIESLKPKLKVKLECS	S	64
7828	LOC_Os12g25530	QLRADCSKVQVDSVKPEYELFPLKY	S	363
7835	LOC_Os12g31040	RCLHSVKVPTGFSANVRKLVSLKDL	S	841
7850	LOC_Os12g34370	IWILTPHDAVAFSRRFAVVEKRWRV	S	23
7874	LOC_Os12g38970	RPPRRQENEYSESEREESEYETEGE	S	568
7917	LOC_Osm1g00500	NKVGSKNVVAGESLIKKRIEERFFI	S	44
7921	LOC_Os01g01270	HQINMAKEDEEKTAFFTPFGGALSG	T	1073
7922	LOC_Os01g01510	DAHEELMAIGPVTNEVTAGQSKDKG	T	246
7923	LOC_Os01g01510	ELMAIGPVTNEVTAGQSKDKGKMQP	T	250
7924	LOC_Os01g01800	NIPYGRPTEFLITSADGVEYNLKPA	T	713
7925	LOC_Os01g01960	RIDTNGVIIRVKTLFNGYPELILGF	T	20
7926	LOC_Os01g02020	KFNSAKLRKLGPTFKKNGSVTAGNS	T	248
7928	LOC_Os01g03760	TGMFCWAHFLFPTLCAKPSGNPQTR	T	329
7929	LOC_Os01g03820	EIGIRQSSSGGSTSGIADNKVSQTF	T	320
7931	LOC_Os01g04330	FDVYDVDGDGRITAAELGKVLGRIG	T	121
7932	LOC_Os01g04330	GREVAAMMNELDTDRDGFVDLGEFA	T	77
7933	LOC_Os01g04650	PQPQQSHAFDPNTGMYYIPMRTNAP	T	525
7934	LOC_Os01g05010	LPLLRASASAAGTTRGAAALLRPLA	T	27
7935	LOC_Os01g06290	RDSRSPKGSPRDTQSPRGSPRDSRS	T	235
7936	LOC_Os01g06630	AELDRRARDDGKTVIKSGTGGKSLD	T	30
7937	LOC_Os01g07110	NSSVIAVQSMFHTGLVRPDPVSRSS	T	209
7939	LOC_Os01g07520	DLKITIGNYNGSTDGKLRQLENMLR	T	187
7940	LOC_Os01g07880	AQQARERKKAYMTELEAKAKDLELR	T	136
7941	LOC_Os01g08260	SITISGYPKNQETFTRISGYCEQND	T	92
7942	LOC_Os01g08410	KKAAVPVAPPKDTERQLSKKELKKK	T	177
7943	LOC_Os01g08970	LEKGFFFLPKPPTLILHEEIEFVEF	T	376
7944	LOC_Os01g08970	TSNTQFLGDENRTAAQVLWETIMGV	T	186
7945	LOC_Os01g09280	VASHAQKYFIRQTNSSRRKRRSSLF	T	158
7946	LOC_Os01g09550	ICYTHPEKLPGVTRDGLSKHFFHRP	T	153
7947	LOC_Os01g09620	PARYRTQPCKDGTACRRRVCFFAHT	T	161
7948	LOC_Os01g09790	SDDASSAAADPFTAAVATVARAPAK	T	89

7949	LOC_Os01g09850	CDALAEESARAVTAAAAVAGQQQHG	T	211
7950	LOC_Os01g10140	KHLLYGASEFEETPRDLDDVFSEAC	T	509
7951	LOC_Os01g10350	NEKTNENFQVGATDPFPSQHVAPSS	T	266
7952	LOC_Os01g10350	PFPSQHVAPSSFTTRNALAPLPSNS	T	280
7953	LOC_Os01g10820	GIRRNEKIACYVTVRGEKAMQLLES	T	72
7954	LOC_Os01g11040	GSTSHSSETNQSTVRTPDTVIGQTE	T	802
7958	LOC_Os01g12360	LGAPDPQEGPEATVGRPHLSPFDPE	T	1204
7960	LOC_Os01g12590	RHYFQAHRVTVVTSYPLGQILHNRE	T	1371
7963	LOC_Os01g12760	VDLVEHLTAYSNTVVSRAVFGDESA	T	201
7964	LOC_Os01g12780	WIVYDLDAKELRTWYEERLLRDRGM	T	19
7967	LOC_Os01g13680	DRTTFVFWDAYHTSDATNQVIADRL	T	19
7968	LOC_Os01g14690	GPLEALRPKLQPTRQQQQRSRRLIN	T	95
7969	LOC_Os01g14860	SFGIVFQAKCLETGETVAIKKVLQD	T	94
7970	LOC_Os01g14920	SPKRDRLRSPVRTTSDIRPRHTDSR	T	202
7972	LOC_Os01g15020	TPPTENGVPNGRTSTSSATSNPAAD	T	1113
7973	LOC_Os01g15020	TVACTLSQGSSVTSMDFHPTRHTLL	T	343
7974	LOC_Os01g15039	GNQGTCLDGFHGTDSRLLRRVESAV	T	199
7975	LOC_Os01g15260	YIKKEFDKNHGPTWHCIVGRNFGSY	T	94
7976	LOC_Os01g15600	GGVGGDVELVSKTLQFEHKLFYFDL	T	41
7977	LOC_Os01g16020	GYLRRWDDEFRPTFRFLDLLQRVFY	T	367
7978	LOC_Os01g16240	LGTVMRSLGQNPTEAELQDMINEVD	T	45
7979	LOC_Os01g16390	SFQGGAPQLERLTLAVLKKPEDGIF	T	932
7980	LOC_Os01g16400	HRVKLFEPFEAMTIPPGLSAMESLQ	T	754
7981	LOC_Os01g16430	AHDAPSAANSVETDALARFAVDEHN	T	63
7982	LOC_Os01g16500	HLLEDYGSSSSSTPRDMFAIGGTST	T	230
7983	LOC_Os01g17320	VFNKMKDLGFPVTVFSCNQLLLLYK	T	277
7984	LOC_Os01g18070	SRHRTLMNIFDKTPHVHRDAFVAPS	T	52
7985	LOC_Os01g18890	FTSDATLVSQSDTAALVDLYARNRK	T	191
7986	LOC_Os01g19150	SGNSMDIGHTIVTTVGGRNGQPKQT	T	57
7988	LOC_Os01g19940	LQQGASNSTKGTTEPLKPKLQRTAY	T	105
7989	LOC_Os01g20940	LFSAADLSEQVVTAELIGKEDIPLT	T	100
7990	LOC_Os01g21180	IGYTKMSAKDIKTINKGIGVDNMHY	T	157
7992	LOC_Os01g23540	TNLSNAFEEAAATAHPEQPPTGDAN	T	230
7993	LOC_Os01g23640	VILLRYSSLVCETIPKHRDYHTFKL	T	69
7994	LOC_Os01g23930	SYELVIVTTVQPTIQEWEHAADNLA	T	1227
7995	LOC_Os01g25040	LRVMLPKPNLLPTNTYEVKKLICPL	T	387
7997	LOC_Os01g25370	VHESSNIVITRETLQCLNETEWLND	T	303
7999	LOC_Os01g26920	STTTAPFSVNLSTAAARAPRLLLLS	T	35
8000	LOC_Os01g26970	VPYVIFCAPPYRTDDYPGDLRVAAS	T	163
8001	LOC_Os01g27790	LLRHPPVVPDPETPARRWLDDVADL	T	126
8003	LOC_Os01g28560	QKRKLNSDEITSTSNSKTKVQKTGP	T	213
8004	LOC_Os01g28660	DVDQWHRGDLHQTPGEEVQAKISRR	T	381
8006	LOC_Os01g31360	QQQFGQDGSGSDTMVPQAYSPKSKN	T	578

8007	LOC_Os01g31580	RYQHASSMGAVETRKGKTMSGTASL	T	343
8008	LOC_Os01g31629	HVQQQQYVDPYRTMVLSPQPDHLNA	T	17
8009	LOC_Os01g32720	PQPDPCLLKDASTGITKPAKEQGKL	T	208
8010	LOC_Os01g32870	VYGDNFFKPASYTIEAMCAKSYEDT	T	279
8011	LOC_Os01g33240	VEGEDTTLWDNETNIPDDWTTISMS	T	343
8012	LOC_Os01g33370	SSQTYKCEICSRTLLDDFRFCSLGC	T	131
8013	LOC_Os01g33800	SFSMNGKEGRGDTSVWTDTPLERAQ	T	478
8014	LOC_Os01g34330	ATAKFDVMEKQLTSPRDHQPDKPLA	T	595
8015	LOC_Os01g34780	EDSQEYKALDSETYSRELFEECVVH	T	265
8018	LOC_Os01g37080	FMPESATGREIVTLGEGRPAPDYPG	T	42
8019	LOC_Os01g37920	EVGLGAGASAAETAEMAATARTARM	T	38
8020	LOC_Os01g38160	RAFKSGVRDRYTTQELATRRITTTQ	T	526
8021	LOC_Os01g39780	ANIFIGVYVFAKTYKRDQEKKNAQT	T	46
8022	LOC_Os01g39780	TYKRDQEKKNAQTAAAAAAVVALSS	T	58
8023	LOC_Os01g40050	SLDRCDWLDKKNTIFGKVTGDSIFN	T	129
8024	LOC_Os01g40070	RQRVAVCIWRLATGEPLRLVSKRFG	T	273
8025	LOC_Os01g40110	KQANGVEQTPKETVGAPVTESAQVN	T	142
8026	LOC_Os01g40110	SKKQKIKWKKIITKVLQTNPDGVLK	T	241
8027	LOC_Os01g40370	PGNGGYQRTTDATDFAKEAAAHAHG	T	31
8028	LOC_Os01g40390	LLRLPHVVHRVLTERRRCHVVPVPE	T	262
8030	LOC_Os01g41090	KRHAIEDDLHVLTQNPGESLRDYVR	T	527
8031	LOC_Os01g41280	RRLYLGVFRFPDTTGHPRGPDVFPH	T	194
8033	LOC_Os01g41430	MAALFAARGVRCTILTTPVNAAVVR	T	41
8034	LOC_Os01g41550	VSGLAQGSGEYFTKIGVGTPVTPAL	T	149
8035	LOC_Os01g41630	NPTRPARSLSTLTRVVRRGAESTGI	T	457
8036	LOC_Os01g41880	EEEEKEPEDKEMTLEEYEKVLEEKR	T	243
8037	LOC_Os01g42250	YAKKVNLSVADLTFAFDGDKVDAES	T	204
8038	LOC_Os01g42294	ALSAARGLAHLHTVHSLVHGNVKSS	T	494
8039	LOC_Os01g42350	TAVISLLQPAPETYDLFDDIILLSD	T	413
8040	LOC_Os01g42370	YAFGQVAIEFPYTLVQSIIYGIIVY	T	1282
8041	LOC_Os01g42410	SEDALKEKHANLTGEVVEGQKDTKS	T	795
8042	LOC_Os01g42850	GMDFVMQVINEPTYLEDLTGLTDLM	T	1008
8043	LOC_Os01g43050	NFEPEIQPHVQDTDVEQQPACTSQS	T	720
8044	LOC_Os01g43340	IVTLLRDVVFDETQFPFTKLHPNAG	T	646
8046	LOC_Os01g43774	FELAASYTHAPHTVMTLHPMHGAQM	T	633
8047	LOC_Os01g43920	SLHTRTLDELFLTKKLLQKYQIAFH	T	144
8048	LOC_Os01g46070	LVSSLSLYDIAGTPGVAADVSHINA	T	64
8050	LOC_Os01g47600	PLVDRRRPKKPPTAFFYFMEDFRKT	T	54
8052	LOC_Os01g48280	LKDKWSPALQIRTVLLSIQALLSAP	T	105
8053	LOC_Os01g48640	HTTDPAILEVAATLALYGPSSDHGS	T	112
8055	LOC_Os01g49190	FVEQATEQQILVTGIKVVDLLAPYQ	T	211
8060	LOC_Os01g49890	AERLGREHLGGMTDLWVKHCGISHT	T	187
8064	LOC_Os01g51080	VKEVALGDGPSKTTRIGDMPGVPRE	T	776

8066	LOC_Os01g51890	PLDILAQRLPDSTKPFISEKALRSC	T	365
8067	LOC_Os01g52390	RDGAGRGNWGTATDEALAQETEEAL	T	191
8068	LOC_Os01g53000	SIVDPQTSLYACTVGQKPSKAKYYL	T	830
8069	LOC_Os01g53370	EALIFPEGFLRRTKGRGLVVMSWAP	T	354
8070	LOC_Os01g53370	GAELGIPTYFFLTTCIASVAFMLYL	T	158
8074	LOC_Os01g53680	HRVTSTRNKVKITDRMWARLLSSTN	T	504
8075	LOC_Os01g53680	STVKQYFVNADDTVPEKVVVQKDSP	T	129
8076	LOC_Os01g54400	LTGAEQPSKNAATAATAAAGNSSAA	T	89
8077	LOC_Os01g54400	VPRTIETTPFPTTFVQADTASFKQV	T	61
8078	LOC_Os01g54530	GVLEALLWRTNSTFAISEHGARSNI	T	332
8080	LOC_Os01g55040	LNQQFVIQLGLFTALPMIIENSLEQ	T	1421
8081	LOC_Os01g55050	QHPPVQNQMRPQTPPNYPHYQPHQS	T	406
8082	LOC_Os01g55050	YADNLLRSLEGITNRLSQLEIYCYK	T	92
8084	LOC_Os01g55650	ENGILFNVVKRDTLLTPTGGVELQG	T	785
8086	LOC_Os01g56140	ELQKLKDERKEATLERERREQELSE	T	677
8087	LOC_Os01g56140	NLEKALHDMRGETAETKVSYESKLA	T	144
8088	LOC_Os01g56490	EEELPHTNPGLNTTPLRFTKHSNAY	T	500
8089	LOC_Os01g56800	GLQYRQFQREIPTNLVSYQLPGPVG	T	1114
8092	LOC_Os01g57420	PEKFKNQLSNQKTYLKLACTQGWFC	T	265
8094	LOC_Os01g58580	DECIVPSDMNLITDQDFRELVQKVP	T	116
8095	LOC_Os01g58620	VLVVSYFAACGVTAVLFRAAVVKGR	T	237
8096	LOC_Os01g59250	HIDLHHTVFSGHTVVGNHSAVAGAA	T	131
8097	LOC_Os01g60190	NAVEAVKTLRAETKASDQYLPPFVI	T	251
8099	LOC_Os01g61210	GNTWEETRVFVDTIVRCNLQSLART	T	178
8101	LOC_Os01g61370	TPDQRVGSFDRITSKRFEDIFLGRA	T	151
8102	LOC_Os01g61660	KANGKLRMCIDYTDLNKACPKDPYP	T	885
8103	LOC_Os01g61720	TGGASVRDDASLTSTPALPSYMQST	T	414
8104	LOC_Os01g61910	QEEVWSLQETFSTSAVIEDNEARAL	T	207
8105	LOC_Os01g62030	AVVGGTGRYDGATGFAVVRAADAHK	T	133
8106	LOC_Os01g62290	KGEGPAIGIDLGTTYSCVGVWQHDR	T	15
8107	LOC_Os01g62290	RAKRTLSSTAQTTIEIDSLYEGIDF	T	286
8109	LOC_Os01g62500	FGTTSGVVVLAGTNRPDILDKALLR	T	466
8110	LOC_Os01g62650	AEREVLINEMLGTTDENEPLQAMMK	T	982
8113	LOC_Os01g63250	GNLEEQGANKSETSEPNSDNIESAS	T	304
8115	LOC_Os01g63770	SCASNQVAQVLLTLPYSFSQLGMLS	T	74
8117	LOC_Os01g63810	RNRILHVADTSNTLIVCEDWDEAKN	T	209
8119	LOC_Os01g64450	DCYIRVPDPSNATAVAMGFTATRCR	T	224
8120	LOC_Os01g64650	RDLDETKIILHKTIESVLQRGERLD	T	174
8122	LOC_Os01g65260	EGAGIAAAGDDGTIKLERGLGLVGD	T	126
8126	LOC_Os01g66520	GVGKESDIFEVATEDGTVLAMKLHR	T	112
8128	LOC_Os01g67170	DEDDLDQLLELGTSNRRGIESHHGC	T	461
8129	LOC_Os01g67250	NDLGQVTAEKGTTESDGSNKVGSLT	T	522
8130	LOC_Os01g67330	AAGAYLYTLFFVTVPALASVYNEKA	T	218

8132	LOC_Os01g67690	AWRNIEALYSTGTRARAVNTRLALT	T	124
8133	LOC_Os01g68160	AKQAKRTPIKNDTPKRSASYVCNSC	T	286
8134	LOC_Os01g68160	KEENEPVVTPLKTFPHEMAKIEAPI	T	238
8135	LOC_Os01g68750	SSSTEKVATANATPQPAKEQVIPSA	T	822
8138	LOC_Os01g68920	REEVLERVEGSATSTSTGDGERRLL	T	52
8144	LOC_Os01g70020	SSKESDVTVDKTTKKASKSKKDEGK	T	368
8145	LOC_Os01g70100	VKDVVVNGITVKTKYCDTCMLYRPP	T	149
8146	LOC_Os01g70320	ARESKDGKDGEVTTLKHELDCAKEE	T	298
8147	LOC_Os01g70330	DRKIMSSGQKAFTGSIIEHDDNIST	T	366
8148	LOC_Os01g71000	KLGPVGDKTHVSTRVMGTYGYCAPE	T	244
8150	LOC_Os01g71050	VILPRSTCDFTVTMQAQRTAPPDMQ	T	69
8151	LOC_Os01g71960	AAYAILITLHRETINGKSHMKKQEL	T	129
8152	LOC_Os01g71990	EAFSSIGAHILETNAQVVDDSDVIV	T	72
8153	LOC_Os01g72270	KRMHYLHAAITETMRLYPPVPLASR	T	396
8156	LOC_Os01g72690	LLTEDSYYNFIQTWDDDEEKKMLHT	T	313
8158	LOC_Os01g73480	HLIQQLMVLAGWTTRSPWLQNHASM	T	117
8159	LOC_Os01g73620	PGQRLEERAKSATTSKRVRSSLAAD	T	169
8160	LOC_Os01g73950	ARKFPPFADPFPTHIAKYGTFDDAR	T	495
8161	LOC_Os01g74230	EAPEPNGGPGPPTTGVGPLPACQTV	T	1007
8165	LOC_Os02g01380	DEEDVGVGVGRATDDHDDQVDESHD	T	356
8166	LOC_Os02g01960	PRSRAPPSPSPSTSSRAKPRKVAAQ	T	38
8167	LOC_Os02g02290	RSGHRVLLFSTMTKLLDILEEYLQW	T	1319
8169	LOC_Os02g02410	AYLGKKINDAVVTVPAYFNDAQRQA	T	175
8170	LOC_Os02g02570	AHFSSTLPPGIDTVWFEYKGLPLKW	T	75
8171	LOC_Os02g03060	DGAGEHPSTSAMTMPFAVSDPSASV	T	54
8172	LOC_Os02g03820	SNCSNALEEDLRTVEEWSSKYRAEE	T	541
8174	LOC_Os02g04490	NGAGYFNGEPTVTSQMQQQKQFPSN	T	248
8175	LOC_Os02g04560	NKLEKERVVPKITIGEVCPFMRNSE	T	504
8176	LOC_Os02g05260	TVGVVSRFMERPTVMHFKAVKQILR	T	1142
8177	LOC_Os02g05310	LIDGILYAFQEQTSDDANVMLNGFG	T	882
8180	LOC_Os02g05510	SKSKAEEATSVITSSEGSPNWGAVE	T	215
8181	LOC_Os02g05900	DTLSFEQSILKITSMKLGNLWNNPN	T	83
8182	LOC_Os02g06370	ERLSLPISKAEFTVMRTALSFALPH	T	182
8183	LOC_Os02g06584	SVNEDLNERSPNTAATKKRSIPYST	T	270
8184	LOC_Os02g06584	TMTRSDKYRSDVTDFDKTSKGTEAT	T	311
8185	LOC_Os02g06740	PVPAILMNCDNQTTCEKTIEVCQEI	T	1108
8186	LOC_Os02g07070	APTEMIDSDIKPTPVSNSLGTTIMG	T	757
8187	LOC_Os02g07070	TEDSLPADPTSSTPLHPKRGSELGP	T	387
8188	LOC_Os02g07210	IPDVNNATIDHETLLERLGTYGLAE	T	75
8189	LOC_Os02g07260	KKLADLTTTKGATTIIGGGDSVAAV	T	353
8190	LOC_Os02g07630	FFIRMVCPRIHFTRSLLLMHLGPFY	T	331
8191	LOC_Os02g07870	DALAESDKITLETAKLLREDYLAQN	T	512
8192	LOC_Os02g07940	LADRVARKEEAWTWNPSGSGVAASA	T	399

8194	LOC_Os02g08300	GTQFEIRVQPNDTIMAVKKIIEEIQ	T	22
8195	LOC_Os02g08360	DSKEGDTLQRWRTEPFIMITGSGRW	T	404
8196	LOC_Os02g08360	VIESFNDLYRSITGRITAEALKERV	T	553
8198	LOC_Os02g09740	VATMKISPAEKMTPERIYGKTGSMR	T	373
8199	LOC_Os02g09750	KPPPPPSPSPRPTRALVCRAAAAGE	T	27
8202	LOC_Os02g10690	KNKSAGETEETVTPPGRPVRLSLDS	T	290
8203	LOC_Os02g10790	ESKIEEILRNSATSEFGGVYYYSER	T	531
8204	LOC_Os02g11060	PREIDKSPGSVRTVAWLHSDQSILS	T	150
8205	LOC_Os02g11750	INRRVVRVNEVRTRGAREFGREGFR	T	86
8206	LOC_Os02g11780	RSAQKETPSKVQTQQLESVRSKFRE	T	157
8207	LOC_Os02g11840	SSRLNYCVGGEVTTLFHLDRQMYLL	T	258
8211	LOC_Os02g12540	RALENVHRIVDSTLEEVIEERRGAA	T	253
8213	LOC_Os02g13330	ERAAGPGRRPTCTSLVAQRVDAPLA	T	48
8214	LOC_Os02g13500	EGNTQGEVIVRGTNREANDSDDEEP	T	241
8215	LOC_Os02g13560	QQFAGINGVLYYTPQILEQAGVAVL	T	548
8216	LOC_Os02g13990	GPKVVAPTPEQITAIKAAIVNSQTL	T	208
8217	LOC_Os02g14059	EHIDLGIKYDPSTGIYGMDFYVVLE	T	122
8218	LOC_Os02g14770	ESLRAIPWIFAWTQTRFHLPVWLGF	T	780
8219	LOC_Os02g14780	LSVRTSFLKDNLTLAYYNIGPGVVI	T	768
8220	LOC_Os02g15060	VECIDMWLHSHRTCPMCRCDLSPPR	T	142
8221	LOC_Os02g15220	LDSKSRPLSHTPTYSARTPSMKKPK	T	467
8222	LOC_Os02g15310	PVRDVYLPKDYYTGEPRGFAFVEFV	T	74
8225	LOC_Os02g17320	IRGMHTIIRDAATTTHDFIFYADRL	T	284
8227	LOC_Os02g17870	EEEEESEESEYETDSEDEQTGMAMV	T	167
8228	LOC_Os02g17870	NEDTTDWNAPWATNGPLRAKYNAKM	T	398
8229	LOC_Os02g17970	GTTAVTVLVRGKTIYIANTGDSRAV	T	218
8230	LOC_Os02g18330	EKFNMDKSYPSKTPMVVRSLDVEKD	T	536
8231	LOC_Os02g18430	FGVFLYELLHGTTPFKGSGNRATLF	T	369
8232	LOC_Os02g18550	DNYMLRLFCIGFTKRRPNQVKRTCY	T	143
8234	LOC_Os02g19220	DVEQTSESSDDVTEKSASKTLDQEP	T	515
8235	LOC_Os02g19860	ASVDLGCSDEILTIIAMIQTGNIFY	T	1029
8237	LOC_Os02g21150	FRPGAIEKYDGSTDPEEFFQVYSMV	T	486
8239	LOC_Os02g21920	AKGGRPELANVATCNPCKKRGGGGW	T	125
8242	LOC_Os02g23970	HLPNDVPLVKLVTINKALPGDTPDY	T	445
8244	LOC_Os02g24080	LEGYAAYKAEETTQRVQGCVRIAEE	T	136
8246	LOC_Os02g27230	RSYIQRFCQVRNTIPCIPAHVVIYA	T	341
8247	LOC_Os02g27740	ENKFGSNDSDFGTQSGRSFRHDPSF	T	240
8249	LOC_Os02g28980	KLKDYKEAKELCTEVLELDSMNVKA	T	529
8250	LOC_Os02g29400	RSSVAAHGREAATMAKKLLRSTGKA	T	35
8253	LOC_Os02g30050	KSLLPLDLRPKKTRAIRRRLTKHQL	T	88
8256	LOC_Os02g32350	DNFAREAKEFLRTRLIGKQVTVEME	T	444
8257	LOC_Os02g32350	QKQYRAMIEERDTSGGKSKGQGTGT	T	891
8258	LOC_Os02g32490	NPSEVCSENLDVTKGPVQISWFKGG	T	112

8259	LOC_Os02g33080	FACTDVESENVDTAERLIREAHKKG	T	30
8260	LOC_Os02g33610	DEFGVQKIIPDTTFIKKWSHKIEAV	T	130
8262	LOC_Os02g34270	PAPSLVVPPLAGTGGGRRVDVAREE	T	48
8263	LOC_Os02g34500	PILLEENNESAATGNIIEEKNDVQL	T	453
8265	LOC_Os02g34590	CSKEGLKEGERLTDANFNDPHTRTN	T	150
8266	LOC_Os02g35130	AREQEVHDAIGYTEEQISELKKEIQ	T	235
8269	LOC_Os02g37420	LEDARQHANANMTVMLIGNKCDLSH	T	113
8270	LOC_Os02g37420	LTIGVEFGARMITIDNKPIKLQIWD	T	49
8271	LOC_Os02g37870	GPSVKFLVNAVHTMEELKLTGNHLK	T	157
8272	LOC_Os02g37870	HLKGSRPLITFSTNFDEQPHWQLVK	T	179
8274	LOC_Os02g38250	QAEAASNLQNYLTDKVYLEEPEGQY	T	588
8275	LOC_Os02g38840	SYCMNPSSHSGWTRVIVEKPFGKDL	T	175
8276	LOC_Os02g38920	VKVKDSKTLIFGTKEVAVFGCRNPE	T	73
8277	LOC_Os02g38920	WKHHEVKVKDSKTLIFGTKEVAVFG	T	68
8278	LOC_Os02g39030	KWNEENLNDIESTKPVREKITEPKT	T	30
8280	LOC_Os02g39420	SRASLVAWMGRLTHRYELAAGTLHR	T	206
8281	LOC_Os02g39520	LVALKLLRQAEKTLLDDGDRGRQAG	T	683
8282	LOC_Os02g39890	GRSGGQVRDEYRTDYDPGRGGYGKM	T	134
8283	LOC_Os02g39920	EQAPSESMISAITPAMKALIKKELL	T	55
8284	LOC_Os02g40430	DAFNNQSPDVRKTVVFCLVDIYIML	T	1346
8285	LOC_Os02g41800	GEEHEKPASFAKTLTQSDANNGGGF	T	123
8286	LOC_Os02g41800	GSGVIQNSPTDNTSSERLQWFRENS	T	574
8290	LOC_Os02g43020	HRYSEALADAEKTVELKPDWAKGYS	T	62
8291	LOC_Os02g43020	LKDAEKCIELDPTFSKGYTRKGAIQ	T	455
8292	LOC_Os02g44050	AAVRHSAATPSSTPSTREDLRGGPD	T	163
8293	LOC_Os02g44410	GDDRVMANFFPMTLKGQARGWLMNL	T	240
8294	LOC_Os02g44410	PSQPIIGVTPGHTWPLGHIDLPVTF	T	614
8295	LOC_Os02g44654	AHVDLQDLLLRLTFDNICGLAFGKD	T	183
8297	LOC_Os02g44810	AVMASVGSGSELTKCLECSERGRKV	T	195
8298	LOC_Os02g44820	EAELLLERSRAITLNGRDKRGRALV	T	26
8299	LOC_Os02g44930	DKKESTSSKKAKTHDDGEGSDKSKS	T	114
8300	LOC_Os02g46040	QRISKRYVLVEGTLYRRAANGILLK	T	1610
8302	LOC_Os02g46956	TDSDQVAKAAMMTAEGAMNFMRSTI	T	269
8303	LOC_Os02g47130	EEKLPADVTDDKTSEEDDLNEVEKL	T	372
8305	LOC_Os02g47140	SPKKIGEDIAKETAKDWKGLRVTVK	T	50
8306	LOC_Os02g47410	GMPWGGRPGHSFTAGGLPSSFAGKD	T	929
8307	LOC_Os02g47890	NGDAQPSQDKDITEEQVKGNKEVGS	T	171
8308	LOC_Os02g47900	SQIDRERVQTTGTTAYVIDATRYGN	T	447
8313	LOC_Os02g48640	QTEALLTKISGETASATVNSGSLAD	T	271
8314	LOC_Os02g48730	TLKEGSLYKLKFTFSVSNNIVSGLR	T	286
8316	LOC_Os02g49070	RDMLTSDPYVVLTLGEQKAQTTVKP	T	204
8317	LOC_Os02g49080	GPRGNSLKRVEATTDCRVLIRGRGS	T	171
8318	LOC_Os02g49090	PPQLEVTPITKYTSDPGLVLGRQIA	T	208

8320	LOC_Os02g49450	AEEEKMDAKKEVTETSQATTAEHKD	T	219
8321	LOC_Os02g49620	RENKLDRHGEVDTANVDEIQSVDED	T	217
8322	LOC_Os02g49992	LITPHSLFRPHFTTLTQRSPSILSK	T	770
8323	LOC_Os02g50320	TSSSLVIKHAKGTSRSFDGGTRSLD	T	429
8324	LOC_Os02g50550	LPGIDQRVMDDSTISEYAGHNDAIL	T	165
8325	LOC_Os02g50620	SDSIITAYRDHCTYLARGGDLVSAF	T	122
8327	LOC_Os02g50840	LPSRGEQPHPVETAAGSGTQRIITS	T	1476
8328	LOC_Os02g50850	KIDAKLNIYPSTTSELRGGDKQSHG	T	889
8329	LOC_Os02g50910	VLQRKTEEAAMATKRLKESLEAKKS	T	26
8330	LOC_Os02g50960	AGADPSKAMAAPTAMPPTSVMTRLI	T	426
8331	LOC_Os02g50970	SFGEVYRGEWHGTEVAVKKFLQQDI	T	860
8333	LOC_Os02g51520	TFITPIGTYCYTTMPFGLKNAGPTF	T	938
8334	LOC_Os02g51860	IGVQHDWCEAFPTYPRTYDMVHADG	T	570
8335	LOC_Os02g52150	LGRLLSLMDDLATPAGRAGAATLRR	T	108
8336	LOC_Os02g52250	PCISNWKNPKGYTIPLDKRLAADGR	T	276
8338	LOC_Os02g52780	AAVPPPPPPQQQTPMLFGQSNVFPP	T	189
8339	LOC_Os02g52820	ETEAAKAPVDSTTLQLHNLLYEKNH	T	108
8340	LOC_Os02g53550	DEEKTAFITPIGTYCYTTMPFELKN	T	900
8341	LOC_Os02g53550	FKLLKRSGPFTWTEEAERALTQLKA	T	1051
8342	LOC_Os02g53580	EMPDRYRFLDRFTDEQLATMPESLH	T	105
8343	LOC_Os02g53700	EVSGSYNPEVLTTQKRQWSRFQLKS	T	88
8344	LOC_Os02g53760	WAVATTLHDPIVTTKPFSTCYVFKV	T	234
8345	LOC_Os02g54110	TSSGWAYTGHDYTSKKASGDLKKKD	T	1196
8349	LOC_Os02g54910	FKTSVLAVRLSRTRLVVVLQDRTFI	T	108
8351	LOC_Os02g55560	HQNALSSGTTALTAMIFGRSLLVAN	T	160
8352	LOC_Os02g56480	LPGEDLDALVSVTNDEDLEHLVLEY	T	113
8353	LOC_Os02g56530	DEVDPFQIPSDYTWVDANEKKRRMK	T	588
8354	LOC_Os02g56560	RGSLPWQGLKAGTKKQKYDKISEKK	T	220
8355	LOC_Os02g56960	VNPKGEMKGSAITGPIGKECADLWP	T	118
8357	LOC_Os02g57510	NDMDSSRGFLSGTVDKFKMVFETKS	T	87
8358	LOC_Os02g57540	HSYKHSGLANKKTVTIQPSGGKDAA	T	56
8360	LOC_Os02g57640	AGTSERKVTITGTSEAIQAAESMIM	T	322
8362	LOC_Os02g58080	VQFGWALQLSLLTPYIQTLGIDHAM	T	84
8364	LOC_Os02g58220	RNTDVSFTIDDGTGRLDFIRWVNDG	T	95
8365	LOC_Os02g58220	SKSRGASSTMPLTVKQISEAQQSGI	T	41
8366	LOC_Os02g58340	RVEPIHVRPAVRTLLARLEGELTDL	T	103
8367	LOC_Os02g58440	FDKPKDSAHNGKTAQKQNLTNRAGH	T	192
8369	LOC_Os03g01040	TAFITPIGTYCYTTMPFGLKNAGPT	T	918
8370	LOC_Os03g01420	DDGEIIDRIEVFTGVEVLPVGHDGD	T	355
8372	LOC_Os03g01470	APPWPDLAGWRLTAGVETAMVGARG	T	19
8373	LOC_Os03g01850	FMDSCPEFAKNPTLIKSYLYQILRG	T	218
8374	LOC_Os03g02150	NNLVACTTFFSETTRAMLCLVRIET	T	959
8375	LOC_Os03g02160	DSKVKEELYVESTVVIRDDMVVNPA	T	504

8376	LOC_Os03g02350	RELLAAAPTHEATCRARWSEVALTF	T	584
8377	LOC_Os03g03430	LPVVKNQHLLLCTLLIGNSLAMEAL	T	75
8379	LOC_Os03g03470	NVHSVLPKTGVWTNSYWDEFVSLPA	T	121
8380	LOC_Os03g03560	QISGKHCKIYRDTVLGELNRNEPVP	T	116
8382	LOC_Os03g04330	GPSYHHILTTLGTKMSSRITSEQPN	T	100
8383	LOC_Os03g04570	TKQIVKVIPLLATMFVPCTLIAQTN	T	350
8384	LOC_Os03g04590	LPSACVGDMVMATVKKGKPDLRKKV	T	64
8386	LOC_Os03g04960	KPTPQLELFNSMTKKKELFEPLVEG	T	18
8387	LOC_Os03g05200	GSRCDSFGRTSYTYENGHTSPDLLS	T	568
8388	LOC_Os03g05260	YGQVPAEMADEGTEVRKLLIQEQVE	T	191
8389	LOC_Os03g05390	VAEKRWRVFVWKTAVYLITLGMLIA	T	361
8390	LOC_Os03g05460	VERCGRHPSQVFTGVCSTCLMERLS	T	45
8391	LOC_Os03g05790	APIQICDSGVDLTVYAFHDTSLNAP	T	26
8392	LOC_Os03g05790	RPLPLVAILEGITRGTQKYLCKRYS	T	631
8393	LOC_Os03g05812	SSSVVSLNSAPTTPNSSYDNLNESV	T	739
8394	LOC_Os03g06220	DESQIQAVTSGGTVYESAAAFEDLK	T	94
8395	LOC_Os03g06220	SAGGCQVLLFSATFNERVKDFVTRV	T	291
8396	LOC_Os03g06260	ASKPEGGNERLSTSVPAAPRQRMIM	T	702
8397	LOC_Os03g06410	SYGEVYRADWNGTEVAVKKFLDQDF	T	757
8398	LOC_Os03g06710	KMLASNTPPNDATFKALKIILVKRG	T	740
8400	LOC_Os03g08270	GVQTGPAWLEADTAANMFLESKDEA	T	357
8401	LOC_Os03g08270	SIEERTLSLINKTTALNPNAQEFVP	T	14
8402	LOC_Os03g08550	AEFQAAQQSAGCTAPEVDMTGQYTL	T	584
8403	LOC_Os03g08550	LGGGQQIQYNLPTNKLERLNLAGNQ	T	126
8405	LOC_Os03g09100	EQDEIQTTLSNPTKEIIDLSKKISS	T	569
8407	LOC_Os03g10070	GINLNEASCTMDTDRPDMPVKSPFA	T	459
8408	LOC_Os03g10180	DYVLGDKSEESGTKHNTNNAKNNVL	T	324
8409	LOC_Os03g10400	LYKSAPHLNFKHTVFGMVVGGLTTL	T	336
8410	LOC_Os03g10400	SGGGVGKYLKARTAGFADVVADDSN	T	432
8411	LOC_Os03g10420	SMTAYGFEPDLRTYNVLAVGLCQAG	T	315
8412	LOC_Os03g10710	MYHNSLRIKGSNTFIIYEMDQLEAG	T	80
8416	LOC_Os03g11140	PVVTLGGIDLNNTGSVVVKEDRKLL	T	111
8417	LOC_Os03g11200	PPTYPAQAYTQQTYAQPSAMAVAAP	T	749
8418	LOC_Os03g11290	KLAEMPQLEESRTMEKVTTVKQSSI	T	197
8419	LOC_Os03g11380	EAIREDATKTLVTLHRQGEQITRTH	T	83
8420	LOC_Os03g11500	TFISKLQVEGYSTERLLSHSIVWRA	T	445
8422	LOC_Os03g11580	IGTNPDIGHQLLTEAAPCTERSLED	T	317
8423	LOC_Os03g11580	PHYNPFASTFEQTDPTLNIGCDVIP	T	720
8426	LOC_Os03g11890	EAVCEKAAVRDETANAAEEAGQRTS	T	345
8427	LOC_Os03g11910	GIIKQCLADAKLTPEDINDVILVGG	T	363
8428	LOC_Os03g11970	HGIQPDGLMPSDTTPGIARDAFNTF	T	40
8429	LOC_Os03g12010	HKCESCGDLIKSTKIKGPSQDDCIE	T	157
8430	LOC_Os03g12250	SALLHGNRASGRTPLDWETRSAIAL	T	477

8434	LOC_Os03g13560	RKQVPSESVSRFTTPLTPPPMPWGP	T	446
8435	LOC_Os03g14490	NDEVLSLGNACGTFIQWPKDLIEIR	T	362
8436	LOC_Os03g14580	KTNSAVKDSGKTTAQKKRRASSDTD	T	286
8437	LOC_Os03g14860	AAELEKAAEPEKTPPPPPAAGAPSG	T	142
8438	LOC_Os03g15000	ESSPSHYVFAAATPYPPPQYTNPNL	T	59
8439	LOC_Os03g15010	HNYEGLRYRSTFTCTHGGKARADAS	T	155
8441	LOC_Os03g15040	VRARKDITDGGKTTKRPRMITRVGC	T	260
8442	LOC_Os03g15050	GQFPCNRYTHYMTSSTSVDINLARR	T	299
8443	LOC_Os03g15650	GSSKTSDDIKNDTLYSEVDNTGTQQ	T	446
8444	LOC_Os03g15750	DVFLETAKLCKYTEKELGASLFFGL	T	185
8445	LOC_Os03g16000	PKTAATHEHSRSTSEDHIFTNTSEP	T	287
8447	LOC_Os03g16700	FKVPELLIEIPETATVGSLKKTVLE	T	416
8448	LOC_Os03g16910	IQRVKVSWKNTLTNGIVKVSCTSVG	T	444
8449	LOC_Os03g17020	HSTPSKGSNQNGTTGGSYWTEGGFR	T	441
8451	LOC_Os03g17180	AAGWIEVSCWILTGERQTAVIRSKY	T	163
8452	LOC_Os03g17180	QEALDTLVMGNKTTILIAHRAAMMK	T	1352
8454	LOC_Os03g17310	FGSTEDISSKSFTGKEFMSLSDKKK	T	686
8455	LOC_Os03g18180	FDKEGNQWKERGTGTVKLLKHKETG	T	72
8457	LOC_Os03g19250	LLAALSIRGFRVTHTYGLSETYGPS	T	345
8458	LOC_Os03g19250	VAEVVMVDQEFFTLAEESLKILAEK	T	136
8459	LOC_Os03g19290	MSGSSNSSLETRTFLDEVRGLEKNW	T	13
8460	LOC_Os03g19290	VAGALTGAAVALTSDRASHERVVQC	T	149
8461	LOC_Os03g19570	RGKSNSNAFYVETNRKDEHVLSDFC	T	431
8465	LOC_Os03g21040	RIFCKADEGFSVTVRGGSVCLAPTN	T	213
8468	LOC_Os03g21350	RRVKAVPAQSNSTAPAPKARVNHVA	T	304
8470	LOC_Os03g21530	KSLLIKCLVKHYTKQNLSEVRGPIT	T	111
8471	LOC_Os03g22120	RVNVHELSVEQLTVSEYLRFKEELV	T	111
8472	LOC_Os03g22350	DLRDLNDVSDYVTKAGYGSESEVDD	T	257
8473	LOC_Os03g22350	KDFVNVSGPLGVTHFFILTNPKSSP	T	107
8474	LOC_Os03g22880	KNREVSEDAEPKTATEGKKKKKKSK	T	535
8476	LOC_Os03g22890	TRIEPDRRWFGNTRVVNQKELEFFR	T	89
8478	LOC_Os03g24930	AARAVAHLAVQCTSPQPRDRPRMAA	T	373
8479	LOC_Os03g25140	TTRTIDGIIVNSTRREHGGKGVPLR	T	76
8480	LOC_Os03g25260	HYCSVCGSTANYTCVRCGTRFCSCR	T	148
8481	LOC_Os03g25430	AIFEIARGIWRPTSDKHGRDADSDD	T	183
8482	LOC_Os03g25430	DIDFPSDPNHATTQAFSSAAAAAAA	T	50
8483	LOC_Os03g25450	VCVARFHAAVPDTARVARALEALTG	T	184
8484	LOC_Os03g25600	RYTLDSSVLDSETRSGSFRQEVVVV	T	84
8485	LOC_Os03g25720	EDLVLQDTFAQETAVTIEDPNMLRY	T	96
8486	LOC_Os03g25890	ADFAGCVDTKKSTSGYVFTLANGAI	T	765
8487	LOC_Os03g25970	GNEESDEGLAQVTVRLLVPSDQIGC	T	139
8488	LOC_Os03g26000	EGLTENSDKSLHTLFAVLNTVFSKL	T	198
8489	LOC_Os03g26630	TVENEHLHAENNTEPFVEKTQDVGT	T	163

8490	LOC_Os03g27460	RGGSSRGRVKKTTARKKVGAGLKRR	T	296
8491	LOC_Os03g27800	ALAQIELAKESETRTLERLQQETKE	T	652
8493	LOC_Os03g27840	CRVHRDDALYQATEDQQGLIPWNGK	T	60
8494	LOC_Os03g27930	KNGRRVSYEAANTLIDDDSRTCTGT	T	118
8495	LOC_Os03g28960	SVTSSTHEIKSKTVIFMEKEKIYLQ	T	226
8496	LOC_Os03g28980	EFDAVTPAAISETKVAVATIRRKSQ	T	297
8497	LOC_Os03g29085	VEELPSVLWAVRTTPTTSNKETPFF	T	863
8498	LOC_Os03g29750	GNFECDVESKAFTLAPDASTNREFN	T	374
8499	LOC_Os03g30460	ALTQLLLYYTRITECVKKINGGSAL	T	669
8501	LOC_Os03g30790	LPWITQFEPIQVTLQTDQVRNIPCG	T	113
8502	LOC_Os03g30990	KSPTRLKEVHKLTGCMAALSRFVAR	T	935
8503	LOC_Os03g30990	PQIQKLLYAVIMTSRKLRHYFQAHR	T	1050
8505	LOC_Os03g33012	NIESTEVSHGFQTTALTEDKPADDG	T	190
8508	LOC_Os03g36610	RLQSGIRKDKIYTDGTVRYSCFTSS	T	30
8509	LOC_Os03g37330	EGFSEEFLDAFLTLLRRAHRHSRIA	T	78
8510	LOC_Os03g39170	AAPPLEARAKEATAMAKKWIAMVAG	T	557
8512	LOC_Os03g39400	KEYLDISCLMLATMSPELQRQYEAL	T	174
8513	LOC_Os03g39570	VWCMMQYIDAKKTKEPIGFLDPTRI	T	721
8514	LOC_Os03g39590	GFEYLYVTIDKFTKWPEAYPVVKID	T	1177
8515	LOC_Os03g40550	STLYHLNVNGVQTRAIKMDPSAFTA	T	296
8517	LOC_Os03g42770	LTMDALSKDRLLTVARDRTMHLWKI	T	319
8519	LOC_Os03g42840	INKEEFQLALFKTNKKESLFADRVF	T	78
8520	LOC_Os03g42840	LSDEVIESIIDKTFEEADTKHDGKI	T	170
8526	LOC_Os03g44390	IEELPAVLWANRTTLSRATGETPFF	T	1737
8528	LOC_Os03g44440	AVRSDVIKSNDFTSKLLYCLWWGLA	T	355
8529	LOC_Os03g44440	DHQKGFLEWRTITRQYINETCEPRD	T	318
8532	LOC_Os03g45344	SRAVSAKIRAGATYDLQRNKSNLES	T	48
8533	LOC_Os03g46040	ADSMTMSYDPQATESKQSELDLPLS	T	108
8534	LOC_Os03g46046	RSSAVGIPCEKVTRVRILGGQLRST	T	25
8535	LOC_Os03g46570	CAAAADAAESGVTFGGGQEAAAPRK	T	97
8537	LOC_Os03g47410	TPWQWSAAAGEVTSTEFEVEEPVGA	T	746
8538	LOC_Os03g47760	KRPGEGAGDGFVTSIRGLFNSQRRK	T	976
8540	LOC_Os03g48350	IDKFTKWIEAIPTGEIKADNAIKFI	T	347
8541	LOC_Os03g48350	KSQVLADFVADWTMPDNKSDNQIDN	T	69
8542	LOC_Os03g48674	SDYVWRFNECRNTIPEITDASVIRA	T	460
8543	LOC_Os03g48930	VGASLYYIDYIATFHKIEKGAFGYG	T	122
8544	LOC_Os03g49150	GKAYLFNKVVNVTSGVKEDRMMITG	T	51
8545	LOC_Os03g49500	IRSTLDRHTILKTTLVELGGTLGLE	T	165
8546	LOC_Os03g49580	DTLKTLEMGAVETLIVWENLDVNRY	T	320
8547	LOC_Os03g49580	LRMEKRHNYVRKTAELATQFFINPA	T	204
8548	LOC_Os03g49640	KELRIGETFEGFTGTSSLSTTNDDD	T	345
8551	LOC_Os03g50180	GATKKPQAQIVVTSSDGKTTEKKNN	T	48
8552	LOC_Os03g50290	EEMVEFMEKVAKTTDVGELTVEERN	T	37

8553	LOC_Os03g50290	EKVAKTTDVGELTVEERNLLSVAYK	T	44
8554	LOC_Os03g50300	AEDVESGSSDVETKYKKMYEDDINP	T	570
8558	LOC_Os03g50740	LERQQPSLWRRGTPLAMEVSAGREL	T	71
8559	LOC_Os03g51030	DAIHSLQLILRGTLNDDIKPTRAAS	T	589
8561	LOC_Os03g51230	HQKEIQNHLVEQTFDEYLHEKSEIV	T	549
8563	LOC_Os03g51250	YFNHIKSVADSLTALAWVGFLGKDC	T	143
8564	LOC_Os03g51310	IYFVSEALRDAKTRYPQAQKMLYAI	T	1226
8565	LOC_Os03g51520	ADGRGWNSGEVMTGRPIQVETGYGE	T	521
8566	LOC_Os03g52310	LLAALDYKQAFSTLETSDDNYNEAP	T	423
8567	LOC_Os03g52980	RPKHLVVAIGNFTYLMLPQLEPVVP	T	559
8572	LOC_Os03g53630	QPLTRRGSIVNKTEDAVSGLDQNIC	T	283
8574	LOC_Os03g53960	QKAKESGQLLSNTKVVHKTNRPMKI	T	439
8575	LOC_Os03g53970	SCFDDEYCSSPRTPVLNEETAFSLE	T	184
8576	LOC_Os03g54780	GGSSSPRESVSGTFIRRTGSPSSPH	T	168
8579	LOC_Os03g55070	VSKSDKIVVEKSTVPVKTAEAIEKI	T	128
8582	LOC_Os03g56260	STSSRSHAVLEITVKRKQKGQYGSQ	T	229
8584	LOC_Os03g56790	KQKQVVVEKTGATTESYDDFLASLP	T	55
8585	LOC_Os03g56800	LVSDDSEVIDLITVNDVERIRSYPK	T	209
8589	LOC_Os03g57100	PAVYFLYDLSPITVTIKEERRNFLH	T	298
8590	LOC_Os03g57160	ECESCGAHLIFSTLTSWSPAEVSNA	T	137
8594	LOC_Os03g57430	GLKQSSTPDSEQTNTADVKTEARLG	T	528
8595	LOC_Os03g57480	FGLKNAGPTFQRTTRISLGSQIGRN	T	957
8597	LOC_Os03g58170	AATDEIFCLFQGTIENIAVLKQQYG	T	81
8598	LOC_Os03g58250	PQLSAAAAAAATTSAVVDPVEYNAM	T	113
8599	LOC_Os03g58480	CSDAPPTPEAANTTAASSRSHRHSI	T	319
8600	LOC_Os03g58810	FLIYLKAAKISLTELVFNENKVPNL	T	472
8601	LOC_Os03g59020	EIIIGYTKAINKTVEILEDLVEKGS	T	140
8603	LOC_Os03g59390	EDIINSIIRDVDTDKDGKISYDEFA	T	487
8604	LOC_Os03g59410	ATCRARWSEVALTFDQTDHPPCVAR	T	595
8605	LOC_Os03g59500	ANRTTPSRATGETPFFLVYGAEAVL	T	1752
8607	LOC_Os03g59680	KIAKVVPEQSIATDHAGEYDESLED	T	249
8608	LOC_Os03g60530	SCLLLRFSDGSFTTSFITTIGIDFK	T	41
8609	LOC_Os03g60620	VPAYFNDSQRQATKDAGVIAGLNVM	T	163
8610	LOC_Os03g61010	RDGKSYSTNLAFTPPEYLKTGRVIP	T	216
8611	LOC_Os03g61160	EKKVRLMRNRSLTREEVDAFWRRQQ	T	28
8613	LOC_Os03g61220	EKEIKESAPKLSTVCVYGGVSYNVQ	T	209
8614	LOC_Os03g61690	NLREAASGPEGVTADAIRDAFLATE	T	124
8615	LOC_Os03g61890	AWKKALQLEAEGTEDLEFSCNVLVL	T	578
8616	LOC_Os03g62340	HLNLALLGVEDDTLSHCSTEQTMDT	T	913
8618	LOC_Os03g62379	DQTGTFRGNRLSTSQFGDMDDEYNE	T	23
8620	LOC_Os03g62500	RYYRAPELIFGATEYTTAIDLWSTG	T	262
8621	LOC_Os03g62700	VPELSLEDLKQKTDNFGSNALIGEG	T	66
8622	LOC_Os03g63140	RSNREGDLRQHETTVRKPYAESPNH	T	85

8623	LOC_Os03g63710	LKNMAGETGKKLTSIASNFISDLDR	T	398
8624	LOC_Os03g63920	IEQEPNEYNGFVTKLCQKFRPAGDK	T	635
8625	LOC_Os03g63920	LISKEEAPDSDVTEEMARNFCLKPE	T	672
8627	LOC_Os03g64400	ALDKRQLPEVDKTSMYKASGYMDVD	T	538
8629	LOC_Os04g01480	ASSSAVLAAANNTGGAQHPMWKTSL	T	44
8630	LOC_Os04g02150	PQPTDTAVPDEKTQSPKEQSIDIDI	T	340
8631	LOC_Os04g02500	LETEGLEQASVDTQLTSSNVGFRLL	T	99
8632	LOC_Os04g03430	WSRFTNLVQSGPTLSIPDYALLQHF	T	89
8633	LOC_Os04g04254	AEALKVPIHIFFTMPWTPTSEFPHP	T	299
8634	LOC_Os04g04254	KVPIHIFFTMPWTPTSEFPHPLSRV	T	303
8635	LOC_Os04g04680	PFDRSVNLLDLGTIARTRTKPVLSL	T	26
8637	LOC_Os04g07060	GKDVIRGRVNHVTAEDVLTTPDVIV	T	644
8638	LOC_Os04g07250	ITKATTSHHWFKTLNDLSTLVGFAD	T	409
8639	LOC_Os04g08450	SGDEELSVLPRHTKVIVTGNNRTKS	T	32
8640	LOC_Os04g09700	RTCEGCQFFARQTHLPAQELQTIPL	T	442
8641	LOC_Os04g09860	STSCVVNIGAQVTQVVCVEDGVALP	T	299
8645	LOC_Os04g11990	LVDPISCIPPYATLIELPADPFPPD	T	392
8646	LOC_Os04g13340	SVHSWEDLCQQFTMNFQGTYPRPGE	T	307
8647	LOC_Os04g14510	EAEVAELLAELPTHRPRPLYRRWAE	T	108
8649	LOC_Os04g14580	PYLGEEDELESRTTSIEEGEDDADI	T	1242
8650	LOC_Os04g14640	NFKSSHCKLLKFTSEEGSSAFFLWR	T	91
8651	LOC_Os04g15800	EPKAKRRCSLAETVSFDGETPVAGA	T	116
8652	LOC_Os04g16180	VKKAFRQGFFWPTALKDACDMVQRC	T	224
8653	LOC_Os04g17064	SIIDNAQSLELATLLAGQIARAATV	T	88
8654	LOC_Os04g17064	VTIAMGTNRDITTACKRKIVSPSSP	T	224
8655	LOC_Os04g17640	DESDDEVGLTGQTGRQADQTTDTPP	T	637
8657	LOC_Os04g19770	VEKAFRQGFFWPTALKDACDMVQRC	T	373
8658	LOC_Os04g21220	LDWLHAVGKKLDTVQCSDEEKVVFA	T	412
8659	LOC_Os04g21240	SRPELVRQSLAWTCFVETPRAEPAP	T	91
8662	LOC_Os04g23330	PITDSDEKFNCYTSREMVNNIRTIQ	T	172
8663	LOC_Os04g23400	TEAERVVADIETTNDWWTPLIKFIN	T	407
8664	LOC_Os04g23400	TIYTRRRQTGKHTRLPAQALQTIPL	T	451
8666	LOC_Os04g24880	RAQDNRTKSEEDTRARTETMITATT	T	248
8671	LOC_Os04g27520	LLSELTLKSPRATMYCEADQDQLRR	T	1750
8673	LOC_Os04g28090	EMLSEARARLANTRGKKAKRKAREK	T	161
8674	LOC_Os04g28090	RWYEWLDPSIKKTEWTREEDEKLLH	T	60
8675	LOC_Os04g28180	QVDAAPFKQWYLTHYGVDIGRKKKA	T	116
8676	LOC_Os04g28420	KGHDGDEPFEFRTDEEQVIEGLDRT	T	333
8677	LOC_Os04g28420	KLKEYREAEKLCTKVLELESTNVKA	T	484
8679	LOC_Os04g29180	VTTPAQHIRTLNTILRETPYDPVLN	T	169
8681	LOC_Os04g30010	FTLYDREKIEVATNNFAKENIVGKG	T	444
8683	LOC_Os04g30780	VSQCDFDRLKLSTAPFMERNLEFLI	T	231
8684	LOC_Os04g31090	GSQGWLAYMVIYTPLPGTIYVLLCI	T	145

8687	LOC_Os04g31340	GNISIRDVQYLLTAPNIYKDYRTIT	T	318
8688	LOC_Os04g31500	HEGECGAHSASRTLVGKAFRQGFYW	T	1688
8690	LOC_Os04g32650	GQAVSEGTVNNETLGYFIGRVYLFL	T	340
8691	LOC_Os04g32950	ADDFEPSLIPSKTIPDPDDKKPEDW	T	218
8695	LOC_Os04g35240	FEDPDDPKNFRKTIGRIMSIQYKIP	T	212
8696	LOC_Os04g35800	ADPAAAAPAAAATDPAAAGSPSPPL	T	34
8697	LOC_Os04g35930	VERCGGGGAGYLTSDDGGVVVISAP	T	239
8698	LOC_Os04g36790	AAPPGATGGAGGTSRRMASVNDASA	T	343
8700	LOC_Os04g36890	VDAKQKKNKNKNTSEAEAGAHQNTD	T	334
8701	LOC_Os04g37950	DLSVDIEQNSSITSCLKNFCSTETL	T	211
8703	LOC_Os04g38090	HQLRIKEEDIPNTTFTTRYGLFECT	T	415
8704	LOC_Os04g38480	ISGTIPNELGNLTNLVSLDLYLNNF	T	122
8705	LOC_Os04g38620	TLTFSPNPYFEDTKLTKTYSFSDDE	T	134
8706	LOC_Os04g38870	IMQLLRDNLTLWTSDISEDTAEEIR	T	239
8709	LOC_Os04g39400	YPLGQILHNREGTGRVVKWAIELSE	T	1376
8714	LOC_Os04g40720	GCFLHVKMTVVVTDSHCCRDTSMED	T	111
8715	LOC_Os04g40940	LYNRGVYPEESFTKVKKYGLTMLLT	T	44
8716	LOC_Os04g40950	DSKTLLFGEKEVTVFGCRNPEEIPW	T	77
8717	LOC_Os04g40950	GILGYVEEDLVSTDFQGDNRSSIFD	T	286
8718	LOC_Os04g41820	TSENSDLKIRVQTMEQQVRLQDALN	T	337
8719	LOC_Os04g42010	SEEEEANAVLKNTLVFAEADLEIKP	T	184
8720	LOC_Os04g42140	DLLKGQLMESGITTADILKDVISLI	T	236
8721	LOC_Os04g42600	RNVAQQHNANTPTSQLAALSLGDAI	T	645
8723	LOC_Os04g43130	KKAVLWNMETFQTQYTAEEHAVIIT	T	550
8727	LOC_Os04g44740	AASPDMAYLLNGTPAEAWLGAVQRG	T	249
8728	LOC_Os04g44960	LRNVRWPPRFRPTIAEKYDGSVNPA	T	240
8729	LOC_Os04g44960	WIAEIQAYLTDKTLPEDREGSERVL	T	1629
8731	LOC_Os04g45570	GGGAGGEVVERWTVVCEPWPDAAAG	T	107
8733	LOC_Os04g46500	NFQGTYPRPVRNTIPCIPAHAVIYA	T	221
8735	LOC_Os04g47240	TSPISVPRSPGRTENPPSPGSRWKK	T	399
8736	LOC_Os04g47250	AANLRRTRGTYQTCIFAVPGLARRG	T	66
8737	LOC_Os04g47320	CLLVATVIRLVKTLLLKVLASSFHV	T	451
8738	LOC_Os04g47330	EAALKGYVRRRVTPAIGDAEFVVVY	T	61
8739	LOC_Os04g47700	KKKGIFGMIMKDTKASKGKQSDANG	T	571
8740	LOC_Os04g47740	DDGEPNPRRRARTPPPPPRQSPRRE	T	29
8745	LOC_Os04g49060	AEDDDEKVAALLTQLHLENSGAGEV	T	302
8746	LOC_Os04g49194	PSNPAQFKEIMSTYCREVRQLGLRL	T	155
8747	LOC_Os04g50890	IKEGDSKLFFLATIYALLGIPLSYL	T	164
8749	LOC_Os04g51370	CSYPWKNSELTETEKAERVILVDFL	T	431
8750	LOC_Os04g51390	RVALGPGGDLALTSRIRNTNTDGRP	T	159
8751	LOC_Os04g51580	AWLPKLVKYSTFTARMTPGRTRVHE	T	299
8753	LOC_Os04g52050	NEEPTAADLFLLTHTHRNGKPMKKE	T	808
8754	LOC_Os04g52180	KSLRSKREKRLRTLRREIAQPFYDK	T	15

8755	LOC_Os04g52200	TRVFALVVFVEATEARHAFKKLLYT	T	608
8757	LOC_Os04g52540	KAIKTKDYSPTITVIVAKKRHHTRL	T	874
8758	LOC_Os04g52570	GDDRDFDANNNLTARVPSWKDEVSS	T	147
8759	LOC_Os04g52570	TSPTKSLSEVHSTFSSWHGSSSHQY	T	216
8760	LOC_Os04g52820	TRVYDDPEEEEGTTLDEAGNFNDNL	T	42
8765	LOC_Os04g54680	KRKVEDQGPLKKTIRSNKESNDEIN	T	403
8768	LOC_Os04g55150	LLEGVVAYHNGNTVKARESLTSAQA	T	284
8769	LOC_Os04g55220	QLAATIASFSKMTIEDSRKLLPADL	T	46
8770	LOC_Os04g55450	QGGCPWRVHGYKTQHDTLWVASRVE	T	295
8771	LOC_Os04g55450	TQETEAGQGPDVTPQQAARDRHPPD	T	1558
8772	LOC_Os04g55500	NMAKPEHAIQPCTTSCTDKVENTKS	T	216
8777	LOC_Os04g57140	EDMLCFQKDPIPTSLLKISSDLVSR	T	42
8779	LOC_Os04g57520	RASTNLEYESDKTFKEAGLANSMIN	T	443
8780	LOC_Os04g57600	RHPPIDGMFGAPTTGMPAVSSHYAP	T	96
8782	LOC_Os04g58280	TNEALLVIEAYRTLRDRGPYPADQS	T	116
8783	LOC_Os04g58720	GILGAQGIVPMKTRDGKGSSDTYCV	T	615
8784	LOC_Os04g58750	RDGKSYSTNLAFTPPEYMRTGRITP	T	85
8786	LOC_Os04g59100	HPRSNGQVERANTEILKGLKTKTFN	T	1531
8787	LOC_Os04g59100	RSYIQRFCQVRNTIPCIPAHAVIYA	T	234
8788	LOC_Os04g59290	RMLEKIASKEPQTTTELFQLADRVA	T	359
8789	LOC_Os04g59330	SGSMAPVPMILRTTVHSRAYVLGAL	T	270
8790	LOC_Os04g59400	ETEEMLLNQGQSTSVQVLENYNMNG	T	397
8792	LOC_Os04g59550	WIFQKLDFKFPLTVSCVHFICSSIG	T	74
8793	LOC_Os04g59570	KRGHPPPPPPRRTRTRGPPARPQQS	T	70
8794	LOC_Os04g59624	LQVFKGALFRTATEHKEQTRYFSKR	T	393
8796	LOC_Os05g01040	EWARPLLTQAIETGNLEELVDPRLE	T	293
8797	LOC_Os05g01350	VEALRMLMQMVLTNEEELKLKYFKD	T	521
8798	LOC_Os05g01400	WITEIQAYLADKTLPEHREGSERVQ	T	1470
8799	LOC_Os05g01590	KWKNFDEDDCSDTPYGNFGGKRSFT	T	105
8800	LOC_Os05g01910	EAILTRIKAHLNTLKKYTYGKHIVA	T	847
8801	LOC_Os05g01990	EDKEGLFASCSFTDLGLHPTLCAHL	T	26
8802	LOC_Os05g02060	VAIDMGNPFLNRTVDGFLKIGAVGA	T	29
8803	LOC_Os05g02260	SLPEKHSALADATSGVAAENADNLS	T	42
8804	LOC_Os05g02670	PIVNEVLEGYNCTIFAYGQTGTGKT	T	130
8805	LOC_Os05g02680	AVRKAMATPTEDTLSAKQPDGEESR	T	823
8807	LOC_Os05g03430	NTDDRPQQQHNATDVIVLSDSDEEN	T	601
8808	LOC_Os05g03720	GFEYLYVAVDKFTKWPEAYPVIKID	T	1354
8809	LOC_Os05g03740	AIRADLDHSFLSTKRNKALWEAASA	T	80
8810	LOC_Os05g03820	GSLGLDMMFRTCTVQVNLDFSSEQD	T	222
8811	LOC_Os05g04950	EENFLHLKAENDTQRYKSQIRALEQ	T	738
8813	LOC_Os05g05700	ENDFEDFLLKDTTDYYSLKAQSWIL	T	204
8814	LOC_Os05g05950	LVLGKIGVGKSATINSIFGEEKSKT	T	684
8815	LOC_Os05g06280	DNYNAVANEKESTARENNVAKIKVV	T	183

8816	LOC_Os05g06630	SWDGATKRAMIVTLFTYFKKKPHEP	T	197
8817	LOC_Os05g07120	EVTVQCYAGGEHTAAARTYVRATVS	T	148
8818	LOC_Os05g07130	WALSFVVPPDHETLDFKFLLKPKDA	T	105
8819	LOC_Os05g07220	SLAYPDQHVPEETQVTAGISCPDQH	T	632
8822	LOC_Os05g08600	SSDYFKELYRLKTYHEVIDEIYNQV	T	36
8824	LOC_Os05g09630	VATSPKSMQLGQTLALEVLDLLKTA	T	39
8825	LOC_Os05g11370	HDSDDVDVLINNTLVGIKNTLEKPI	T	271
8826	LOC_Os05g11370	TLAINSKYGTTYTVDQVQHHYRRHK	T	75
8827	LOC_Os05g11730	RYYRAPELIFGATEYTTSIDIWSAG	T	239
8829	LOC_Os05g11780	LSAGLLRQATYTTARLGSFRVLTNK	T	89
8831	LOC_Os05g11980	TQLGLRNAMKRRTKVPEAPLSAQEL	T	934
8832	LOC_Os05g12290	SLLSKKLQELGFTPSKADTSLFFFN	T	806
8834	LOC_Os05g13520	ALSGRLARNVLLTGKVLLNGKKRRL	T	100
8836	LOC_Os05g14750	LLVKEPHKRIAFTRGATEIKQHPFF	T	490
8837	LOC_Os05g14770	QAYDRTGEEYILTLKYIKANNSYRL	T	328
8840	LOC_Os05g16640	GGMSKPSGSVEVTTEATTSGQVGAK	T	240
8842	LOC_Os05g17100	GPASLWWDHFQATQPEGQPITWARF	T	424
8843	LOC_Os05g17750	SFWDYALGTAAFTLNRVPSKSVDKT	T	365
8844	LOC_Os05g17940	ATGAEQVVADIETTDDWRTLLNKFL	T	124
8845	LOC_Os05g19020	VLTASFNSLLRWTITALQAKFSMKD	T	195
8846	LOC_Os05g19100	TPQSSSKPLGKITLPMTFGQANNFR	T	673
8847	LOC_Os05g20100	ARGDLVVCPEGTTCREPYLLRFSPL	T	413
8848	LOC_Os05g20100	DLESWLLRSPMSTFPYFMIVAIEAG	T	68
8850	LOC_Os05g22390	KYSKCWNPIRPPTTLLTSNGHRICI	T	1468
8851	LOC_Os05g22390	PFPVNMVHTAGQTADRARARGFRVN	T	299
8853	LOC_Os05g22580	KKWEDWKKDWFYTALPDHPRLRLPT	T	158
8854	LOC_Os05g22870	KKGIASNTSMYNTLINAYCKANQIE	T	414
8855	LOC_Os05g22920	EQLNQLPSKEHGTNVMRIRPWYLDQ	T	429
8858	LOC_Os05g23610	EIESNKPVRQKITEPKTPYHPMVDD	T	27
8859	LOC_Os05g23610	NKPVRQKITEPKTPYHPMVDDDGSL	T	31
8860	LOC_Os05g23860	QGFARLSAVYGGTYMLNKPDCKVEF	T	248
8862	LOC_Os05g25750	ELTPTDQPFHGITPQSSSKPLGKIT	T	663
8863	LOC_Os05g26890	PDRVDRVFKIYRTTALDQKLVKKTF	T	317
8864	LOC_Os05g27820	FSVLFSMISPFLTERTKSKFVIARE	T	281
8865	LOC_Os05g28190	EDNSKASDIEDKTAKEGDAEEEDGA	T	193
8866	LOC_Os05g28280	DNAEIKAWLALGTKTKKKSGAKKKK	T	359
8867	LOC_Os05g28430	EAKIRASIRAELTSEFDEKLESMRA	T	268
8869	LOC_Os05g30420	DYKAMFMDIKLNTMQHSVGTLKKLC	T	168
8871	LOC_Os05g30530	ILILRNRLKYALTYREVISILMQRH	T	57
8873	LOC_Os05g30980	HKLFIGMLPKNVTDAEMTDLFSQYG	T	146
8875	LOC_Os05g31056	AFSHSNHSSSFSTSGNDMPSFSFKT	T	203
8876	LOC_Os05g31056	SPQSSVSTQRRNTRRKVRTKSGQLP	T	476
8877	LOC_Os05g32360	LTGLADRPGMRFTVPQIKCYMKQLL	T	137

8878	LOC_Os05g32580	RCYWGYSFENLATENSIDEDGRGID	T	349
8879	LOC_Os05g32960	SRGHAVPRMFYRTTIATGGYARDLA	T	505
8880	LOC_Os05g33030	QEVAGLIEAPIYTIDGVWEKLKVGA	T	351
8881	LOC_Os05g33380	ENRRSLRELLFCTPGALQYLSGVIL	T	62
8882	LOC_Os05g33570	TEACQQYQAAGKTLPAGLWEEIVEG	T	132
8883	LOC_Os05g33940	RVFDDGTVDRTWTGPPEVLPLMQPV	T	50
8884	LOC_Os05g34090	HTWPLGHIDLPVTFGGSANFRTERV	T	635
8885	LOC_Os05g34540	VAPKGKFIAFVSTEAETDHPESELK	T	356
8886	LOC_Os05g34780	TGGDSGAHARRRTAALRHERRAPTG	T	503
8887	LOC_Os05g34780	VLRRSSAATKRRTRTAATWRPRRRS	T	565
8889	LOC_Os05g36350	YAAARRWSDVASTRKAIRSRSMRKT	T	477
8890	LOC_Os05g37500	IPKLVLQIVTVETLLRLHKPNNELA	T	1454
8893	LOC_Os05g37930	DGERDAPPKAEVTVGSPQSTLCGLP	T	71
8894	LOC_Os05g38160	ISIAFLNDSFAVTFNEKVTRTVRQF	T	97
8897	LOC_Os05g39390	FRSSAGVGIILPTKLFRVEVNYCYI	T	487
8898	LOC_Os05g39520	WVMNVVPVGQPDTLPVIFNRGLIGV	T	547
8901	LOC_Os05g39780	NVLNTPAWGLHITPRAPEQGHTQHT	T	972
8902	LOC_Os05g39780	YYGAYLRWYRSVTRWRCFPPQGDST	T	772
8903	LOC_Os05g39810	AASTKITVGDGNTTRFWDSAWINGR	T	750
8904	LOC_Os05g40060	ESSSADTKRKPATAQPSTRRRRRAT	T	93
8907	LOC_Os05g40420	DADQYNCIHVAETPTMDSLKKGAPE	T	46
8908	LOC_Os05g40820	DIHAEAVKKRRRTTKKPYSRSIVGA	T	76
8910	LOC_Os05g41172	GILPTFGSTTAFTTTANGVSYTSYT	T	72
8911	LOC_Os05g41172	NLDDSEDDYIFQTVCPGEKTLKFNF	T	554
8912	LOC_Os05g41210	FSLFDKDGDGCITTKELGTVMRSLG	T	29
8915	LOC_Os05g41510	GRYLQNPLAMAATLCGPGKEILSWK	T	901
8916	LOC_Os05g41670	GFRSFLKETLKETVRDAFESRGVHI	T	190
8919	LOC_Os05g42320	HQIRMAREDEEKTAFITPIGTYCYT	T	869
8920	LOC_Os05g43280	SAEGTTDAKANGTPNKDEPVTNCVA	T	712
8921	LOC_Os05g43670	AHFRRFLARRSRTLRHLKELAVLRS	T	225
8924	LOC_Os05g43970	ESDNFQKNKHKGTEGLIEIENPNLV	T	76
8925	LOC_Os05g43970	RSFSSEEQIAAGTSAGRPKNFKKKQ	T	26
8926	LOC_Os05g44270	SSSQPALPPSPRTVVPRTIDTTPFP	T	67
8928	LOC_Os05g44380	GALPRPYDPKANTNVIRDANRRATG	T	321
8930	LOC_Os05g45660	KREGSGRGNWGTTTDEILAQETGEA	T	186
8931	LOC_Os05g45740	ERCQELSKQLFYTRLASLPGRYEAF	T	60
8933	LOC_Os05g46340	FTLYSFRSGDLATDDIPADAAAAAT	T	274
8936	LOC_Os05g46720	LDRFARPCFEGFTHNDEKKEIRSDA	T	17
8939	LOC_Os05g47640	LYRYYKSGWTEFTPQVAEPTFASAI	T	383
8941	LOC_Os05g47980	LGMDELSEDDKLTVARARKIQRFLS	T	476
8942	LOC_Os05g48020	EKVDRANTDLKNTNVRLKETVLQLR	T	228
8943	LOC_Os05g48020	LQRLALKKVKGLTKEELATRSDLVA	T	100
8944	LOC_Os05g48310	RQGQRVRLYVRGTILGYKRSKSNQY	T	16

8946	LOC_Os05g48980	SALESMNVENAFTEVLTQIYHVVSK	T	167
8947	LOC_Os05g49050	PLISRRFAERAFTVGIGGPVGTGKT	T	84
8949	LOC_Os05g50530	TEDGPMFLKAIYTEGEIKRKEYIAE	T	311
8950	LOC_Os05g51280	TRNKIPKERFRITAVDAVGLPTSPR	T	35
8951	LOC_Os05g51500	LVVDIMHGLEPQTIESLNLLKSRDA	T	720
8952	LOC_Os05g51690	QPPPDAASCIYTTALPLIADPPDIL	T	32
8953	LOC_Os05g51690	QQPPPDAASCIYTTALPLIADPPDI	T	31
8956	LOC_Os06g01680	SKDAPVSIRGSATSGPNYEFAETGK	T	685
8957	LOC_Os06g02028	AVKEYDDGKDLTTYDFEADCFSSPY	T	100
8958	LOC_Os06g02130	GKEGEKPDRVVSTNLTFNGKLQPNA	T	342
8960	LOC_Os06g02380	GTAAKVVLNKESTTIVGDGSTQEEV	T	385
8961	LOC_Os06g03310	SSGGLSPWSPPRTSEPFMEKLEVQL	T	1078
8963	LOC_Os06g03676	RTIALQEHELEHTASLLGEIGSNED	T	439
8964	LOC_Os06g03780	HLDNLTEEMEEKTENIVYEDFKFLT	T	374
8965	LOC_Os06g04560	LSMTASDAEDPQTLRMVAGAIANLC	T	733
8966	LOC_Os06g04660	EPQRWMRGKGRVTIQFGCCYNYATD	T	255
8967	LOC_Os06g04660	HIDSHDFVRPFCTVSFLSECNILFG	T	336
8970	LOC_Os06g05180	SGSVCIWDYQSQTMVKSFEVSELPV	T	48
8973	LOC_Os06g05530	CFVEFSDAKCAITAMEALQEYRFDE	T	187
8974	LOC_Os06g05660	EAKYQKLYEPLYTKRYNIVNGVVEV	T	99
8975	LOC_Os06g05660	KKGSKNAKPITKTEVCESFFNFFSP	T	246
8976	LOC_Os06g05850	DELSRLASSRAQTPLGAFEERLAQP	T	1446
8977	LOC_Os06g05850	KSQALADFVAEWTPAPEPVSIPEAS	T	1345
8978	LOC_Os06g05870	PRLQKLGVYGVITLNEPFETLVPSS	T	87
8979	LOC_Os06g06530	GPKLTTPTTSVATPGDPVVDKYISM	T	156
8981	LOC_Os06g06830	PFTWTEEAERALTQLKAYLSSPPVL	T	1012
8982	LOC_Os06g08023	FFQQHLGEKQKYTNLIDGKHFQLEG	T	106
8984	LOC_Os06g08550	PALTTYDIQLVLTLVGRFMRRAGVT	T	350
8985	LOC_Os06g08740	DPQPRKHTLWQATSDFTGGQASMNR	T	232
8986	LOC_Os06g08740	EGSDEEFSSAASTVKTTGPAQPPAM	T	496
8987	LOC_Os06g08790	CHQKTNLEKAKATLLLATLPKSLPC	T	426
8988	LOC_Os06g08850	WWGLQNLSCYGQTLTVSTYIGETLY	T	358
8989	LOC_Os06g09450	IIGNYSDGNLVATLLAHKLGVTQCT	T	418
8990	LOC_Os06g09880	APALPKTNSMKLTEGYFMRAGLVPV	T	1354
8991	LOC_Os06g11610	ATAAAPGTNGLATAAARRGGWWVAK	T	140
8992	LOC_Os06g11970	NMLTQLKCESDVTEDLTNTSSKAPV	T	102
8993	LOC_Os06g12030	EQPTGKGRIIIYTKLGCEDCKMVRS	T	249
8994	LOC_Os06g12030	SSDPMEPTDSSQTEEDILAEDKSEE	T	88
8995	LOC_Os06g12080	DGELDFYSAVALTEKKFLDKFVHSC	T	346
8996	LOC_Os06g12970	TELGAISKIRGRTTRLRISMYADDA	T	56
8998	LOC_Os06g13720	VLVCENNHYGMGTAEWRASKSPAYY	T	240
8999	LOC_Os06g13810	TIDGDLKCKEVPTSFGFDTACKIYS	T	239
9000	LOC_Os06g14080	KLQDDNRALDRLTKQKEAALLDAER	T	177

9001	LOC_Os06g14160	GRSSATKASGAATTSSASSRRRARR	T	54
9002	LOC_Os06g14490	IQEGSSPTPPSDTHSEATSEAKSAG	T	407
9003	LOC_Os06g14510	VQGFNSSTASLLTRYLAVEPSTPYN	T	548
9004	LOC_Os06g14510	WGVKDKIKQFSETFRSGSWVGATGK	T	132
9005	LOC_Os06g16160	DPARFLSSMRGKTLAFIGDSLARNH	T	139
9006	LOC_Os06g16160	GKEAARMLLMDATEAMAQRPDGHPS	T	772
9007	LOC_Os06g16250	ESEDGAAPEMAATEAEFYRAQVEEF	T	411
9009	LOC_Os06g17290	LAVQLDRGDNAHTVKRKLQLALNVP	T	114
9010	LOC_Os06g17820	VDDLVVKTRNQETLLSDLAETFESL	T	993
9012	LOC_Os06g19090	FGDHYILFLVYPTDQTVVVLDPADY	T	459
9013	LOC_Os06g19390	VTVVHLIIMDYGTTVIQEDIPGYNG	T	585
9014	LOC_Os06g19650	PGATSDAAVTAETVDASEKSSDPQT	T	285
9016	LOC_Os06g20370	YSKMKEIAFKKQTELEDIYAGAHMV	T	318
9017	LOC_Os06g20840	ILTQFGMLHCNSTSIPIEHRSQLHK	T	309
9018	LOC_Os06g21350	CYYKANVDGFSATDFHCHWPHGHRR	T	30
9020	LOC_Os06g21580	DKVVDQAEAKNLTEGDDGDADYQQG	T	55
9021	LOC_Os06g22070	FEIIDNPGDQSITLKREIAGETIKA	T	126
9022	LOC_Os06g22100	QPGASPPGSLVATMRGQFEILSLTG	T	166
9023	LOC_Os06g22650	QPSPYSNGLTSNTMAPYDQPSDINA	T	431
9026	LOC_Os06g24540	VMQGWDVEEEESTVVVGEVEVEKKD	T	285
9028	LOC_Os06g24810	KAPEPNGGLRPLTTGVGPLPACPTT	T	1243
9030	LOC_Os06g26190	NLETSTLEDENDTFEHESFSFKFPQ	T	93
9031	LOC_Os06g26190	RNKSIAKTDKGETYHLSRCTEIKRD	T	218
9032	LOC_Os06g27600	RRASRRELLEPGTFLDILMKPSVKE	T	240
9034	LOC_Os06g27830	CPNAACGSSQGLTDRCVRHGGGRRC	T	208
9035	LOC_Os06g27970	QFNELYKHMDGLTNDVERSNEMVAI	T	286
9037	LOC_Os06g28100	VYFVSEALRDANTRYPQAQKMLYAI	T	1289
9038	LOC_Os06g28140	EVYVKRLQSDKLTPKSDKCFFVGYP	T	438
9040	LOC_Os06g29410	KSRKVHSSPQRITLDKPEAKGRGRG	T	548
9041	LOC_Os06g29410	LQTRNQHPHQLGTAGYAGKDEEWQR	T	172
9042	LOC_Os06g29430	EDDANLASSSKNTMISKHHNTVDQK	T	538
9043	LOC_Os06g30320	SENERLRAELNHTLSSINLYKEYKK	T	817
9044	LOC_Os06g30570	APQAPPPAHRRATKKAKIDAAKNKD	T	643
9045	LOC_Os06g30750	LTLIYVAVLMLHTVPILYDKYQDKV	T	217
9049	LOC_Os06g31250	KFDTAYNAIIGRTALAKFMAVSHYA	T	674
9050	LOC_Os06g31250	VFCYVKMPFGLITVGNTFQCTVQGA	T	887
9051	LOC_Os06g33520	DVLAKAKTGTGKTVAFLLPAIEVVS	T	506
9052	LOC_Os06g33520	LPKQRQTLLFSATVPDEVRQVCHIA	T	650
9054	LOC_Os06g34440	EVKYNEDSDGDDTDCHGNGDDGFVS	T	372
9055	LOC_Os06g34440	NADTTGPGHDGPTNGVNNYNNTEDI	T	420
9056	LOC_Os06g35530	GNDPVTGHIISTTIGGKNDEPKRTI	T	57
9057	LOC_Os06g35530	HGPTIMGGNDPVTGHIISTTIGGKN	T	50
9058	LOC_Os06g35730	RCKSRVGIQHRVTKEDAMKWFQVKY	T	157

9060	LOC_Os06g36360	DQMVEEANAVLFTFGSYRLGVHGPG	T	99
9061	LOC_Os06g36360	RLGVHGPGADIDTLCVGPSYVNREE	T	116
9062	LOC_Os06g36700	IAAGKAVARILRTSLGPKGMDKMLQ	T	46
9063	LOC_Os06g36700	PKPKTKHKVDIDTVEKFQMLREQEQ	T	266
9064	LOC_Os06g36730	KRHAIEDDLHALTQNPGESLRDYVQ	T	540
9065	LOC_Os06g36920	AGSPLHQKGLFFTSVMRERLRCRDA	T	130
9066	LOC_Os06g36920	LLSRVPATADWRTARANERLRARVG	T	283
9067	LOC_Os06g37440	LDEWKRLYSNTKTNFREIAIKGFWD	T	282
9068	LOC_Os06g38470	VEEAFYTTDRVMTVSFHKFGDYFPG	T	207
9070	LOC_Os06g38500	MPSPAAAAAALATVLAGGGGARTRQ	T	13
9072	LOC_Os06g39344	STAASASKTLAPTLTFRRFAAMAAA	T	46
9073	LOC_Os06g40440	ALALYAGLVWETTRDADRADAYFTR	T	215
9074	LOC_Os06g40480	RLASSRAQTPPGTFEERLAQPSARP	T	1569
9075	LOC_Os06g40640	NAMNKLEVLKPWTLTFSFGRALQQS	T	321
9077	LOC_Os06g40950	MGQFSTSPFGKMTFETATKFIVEAA	T	299
9078	LOC_Os06g40950	TSPFGKMTFETATKFIVEAASHGES	T	304
9079	LOC_Os06g41120	SPLLDLAGGGGATTTWMATTAWMGR	T	41
9082	LOC_Os06g41860	LPGRFELLSVTGTVLPPPAPPGASG	T	138
9083	LOC_Os06g42190	ATRRARWSEVALTFDQTDHPPCVAR	T	595
9084	LOC_Os06g42200	AAHEAACAEEEFTLRLREDALTERE	T	784
9087	LOC_Os06g43270	RSRKLKNTCSPTTMEVPDGTIVGME	T	367
9089	LOC_Os06g43460	QRISKCYVLVEGTLYRRAANGILLK	T	1580
9091	LOC_Os06g43790	KQPKVLGDQQRPTELRSGQEPRKTR	T	1556
9092	LOC_Os06g43840	GSLRTALLKNAKTLDKRKRLIIAMD	T	938
9093	LOC_Os06g44130	ISIERRRGVCGTTACSKRSPPRSPR	T	190
9094	LOC_Os06g44200	FRGGLRPPPPPPTPFGWEEAARLER	T	63
9095	LOC_Os06g44890	ENSSQETPVYVITEGSEPQFFTRFF	T	752
9096	LOC_Os06g44890	NPVTDIDVTKRETYLSAAEFRERFG	T	980
9099	LOC_Os06g45120	SPRPVSSKLAADTPLLTGQRVLDAL	T	224
9100	LOC_Os06g45360	FGDKGYLAFEHITWTPYQTKLIDTT	T	592
9102	LOC_Os06g45870	LSRWLKSSPAGNTNQMYDENNLIEE	T	321
9103	LOC_Os06g45990	AAAAAAEVAAPETKEVTAKAAADEA	T	22
9104	LOC_Os06g46366	VPGTPSEGQQDNTSTLRSFSFRKVI	T	119
9105	LOC_Os06g46400	LPNQNSIDNTTTTTAQPAVGQQAQT	T	197
9108	LOC_Os06g47320	AIMIVRRALKNSTVVPGGGAIDMEI	T	407
9109	LOC_Os06g47320	DKGGTTISNDGATIMRLLDIIHPAA	T	67
9110	LOC_Os06g47330	QLVSLAQAGDEITQDSLKRDDLGSL	T	90
9111	LOC_Os06g47340	WMGAVSVGILAHTGTADQPRLSEED	T	327
9112	LOC_Os06g47730	FPFSPFLLSLSHTFLLSRQAASWPL	T	21
9113	LOC_Os06g47950	KMVGSGFMPRTRTTMLLMKVFCENA	T	375
9114	LOC_Os06g48534	TPMMRRGPDGPRTLCNACGLMWANK	T	231
9115	LOC_Os06g48640	ILGKYHYMLYILTLAMQPRMLLTVD	T	814
9116	LOC_Os06g48640	QESVEATAEVSKTFDEKIRNYCDVT	T	597

9117	LOC_Os06g48750	TVSATHGDMDQNTRDIIMREFRSGS	T	318
9118	LOC_Os06g48780	YTFVCRSSGDEWTAKQLKGELEASA	T	16
9120	LOC_Os06g49090	ELHAHSQGSTSATIKVTSGQAFVNV	T	763
9121	LOC_Os06g49430	NDQERSADSVARTTVSPPMSQDAQQ	T	504
9122	LOC_Os06g49500	NVLAGTWSSNSYTPQWLTSAMLQYG	T	216
9124	LOC_Os06g49800	ISDAIDLLPRLATGIDVNVMFRKID	T	136
9125	LOC_Os06g49800	ISSELTTAASQDTPNHATKELDGED	T	447
9126	LOC_Os06g49830	SRGFSCATHVKSTWVPAAKRRERQQ	T	167
9127	LOC_Os06g50030	SAVVKKGEYKGQTVAVKIIAKAKMT	T	200
9129	LOC_Os06g50539	TTTIPTSTSADPTSYLEWVRRSREP	T	302
9135	LOC_Os06g51420	SLPKFEDPISAETTEPKQVENPFGN	T	118
9137	LOC_Os07g01870	KAKVAYERRKQLTRLRVKAEKAAEE	T	180
9140	LOC_Os07g02230	SEQIPDFDLLYPTELLNSIDTNNFP	T	1249
9141	LOC_Os07g03180	CMEKGACYKKRLTCPEKCFKSFSFK	T	99
9142	LOC_Os07g03230	MDKQFLKDPSEITWDIVDKKLKEIV	T	254
9143	LOC_Os07g03230	PTKCIIFHNVDQTRLQGLLFQMSDK	T	800
9145	LOC_Os07g05050	HANGGKCIVFTQTKREADRLAYAMG	T	337
9147	LOC_Os07g05610	SSTSTGLSGWQLTVDALETIQSLAQ	T	896
9149	LOC_Os07g06175	YEAQAELPDPTFTIRQLIDNFARKN	T	164
9150	LOC_Os07g06286	RQLIDNFARKNFTVEELVVLSGAHS	T	116
9151	LOC_Os07g07194	SFKDSYGKDKIDTREHRLRFVDSSP	T	238
9152	LOC_Os07g08330	QPYAVSRRAGHQTSAESWGTGRAVS	T	66
9156	LOC_Os07g09060	FGAAGQRCMALSTAVFVGGSEPWED	T	323
9157	LOC_Os07g09384	YKTPSRGPSPVPTPALAPASPPRHR	T	180
9158	LOC_Os07g09890	QTSIDSGILIKWTKGFAVSGTAGKD	T	204
9159	LOC_Os07g10110	FGDESCYASELVTWATKQVMSFALL	T	331
9160	LOC_Os07g10200	VAVLSFRQLRTETMGHGGSDLGYEG	T	43
9163	LOC_Os07g10540	TDITRTVHFGEPTPRQKECFTRVLQ	T	540
9165	LOC_Os07g10890	PPEPPAAAAVAGTAVATAAAAVPTH	T	23
9166	LOC_Os07g11290	ASRSQENEPNADTQVEEKWERRVQG	T	181
9167	LOC_Os07g11290	VRHRPNSRFLTATVRGVQQANRVVE	T	60
9168	LOC_Os07g12170	KIRPLWRHYFQNTQGLIFVVDSNDR	T	208
9169	LOC_Os07g12250	RYFHNRLKPAKLTWTAMYRKQHKKD	T	52
9170	LOC_Os07g12250	SALREIKERIKKTKDEKKAKKAEVA	T	122
9171	LOC_Os07g13340	DESFPRPRRIPPTLAQFMPRNWGQL	T	1296
9172	LOC_Os07g13340	TAFRTPKGNFYYTVMPFRLKNAGAT	T	2030
9177	LOC_Os07g20150	DSDAQELGLFHETEEEFEEQSVRAD	T	441
9179	LOC_Os07g20580	TASVRQTVMFSATWPPAVHQLAQEF	T	281
9180	LOC_Os07g22610	GRCGADVGPTAITTTTPTFQRGSEW	T	1283
9181	LOC_Os07g22610	PQYSWGSAVLAVTYAGLCDACVRNS	T	1008
9182	LOC_Os07g22640	PQGRLTVTVVKATSLKNKELIGKSD	T	272
9183	LOC_Os07g22750	KMFTETIFPRFGTPRMVISDGGSHF	T	501
9184	LOC_Os07g22750	LLAKDAPFEFDDTCLKSFEILKKAL	T	239

9185	LOC_Os07g23290	PETRTGRSVFDLTKKKPVKRKKRTD	T	416
9186	LOC_Os07g25550	LWKVTSIGRKSDTSLSKGKFKEMFD	T	238
9187	LOC_Os07g26440	AQDQIAKGGYSITLEPSSGVGAPWF	T	61
9188	LOC_Os07g27520	STDPEEFLQVYSTVLYAAGVDDNVL	T	296
9189	LOC_Os07g28800	WHPDRHNGSTKATAEEKFKHCSAAY	T	247
9190	LOC_Os07g29520	VRGARLQGYLTGTFEKPKAEVTVTA	T	52
9191	LOC_Os07g30250	ERMFLNRNRFKQTLPIHMAIGIMCL	T	255
9192	LOC_Os07g31840	SLLLGWSSEDEKTFAEALVFVQDSS	T	150
9193	LOC_Os07g32400	YITDYYGSYLHQTKLWIVMEYMAGG	T	84
9195	LOC_Os07g32480	TPRPDGSYMYQPTSPFKRYWNVELW	T	286
9197	LOC_Os07g35580	DQTQDVTNRVVGTYGYMAPEYAMRG	T	529
9198	LOC_Os07g36200	PRNADKKDYRRDTDRSDVYRPTRRE	T	431
9199	LOC_Os07g36420	RFVRRPRPPAGTTHPDLEFFQPGPH	T	277
9200	LOC_Os07g37610	PSGKGPKHKQTCTCNVCMTVRRRFR	T	841
9201	LOC_Os07g37650	PSVAPPPKVDYATDLFNMLSMDGTT	T	227
9202	LOC_Os07g37750	LCMEGFRAGIHLTRVLGMDTMRYAF	T	819
9203	LOC_Os07g37860	SPTRDVYCPIHKTKNHDLSSCKAFL	T	199
9204	LOC_Os07g38120	EFGVTYLCTERETGDAYACKSISKK	T	97
9206	LOC_Os07g38340	RIREEDIPKTAFTTRYGLYEFTVMS	T	919
9208	LOC_Os07g39230	AAAKRILRYLKHTVKLGLKISKSNS	T	1143
9211	LOC_Os07g39470	AARPGGPPTVRITGIDDSVSAYARG	T	315
9212	LOC_Os07g41180	LPNHVDDNTHSVTFTSEPETSGNVN	T	173
9214	LOC_Os07g41230	LHPQQLLAKDLNTKDLETNIIVLVH	T	122
9217	LOC_Os07g42400	DFNYFGDVLCLDTTYKINGYGRPLS	T	350
9218	LOC_Os07g42400	LASAMAAAWPNTTQRTCAWHVYQNS	T	428
9219	LOC_Os07g42410	GVFAYITHTAGSTKSFLRKGNEAHP	T	1049
9220	LOC_Os07g42490	LVACLLAHKLGVTHCTIAHALEKTK	T	435
9221	LOC_Os07g42770	VHRDLKPENFLFTSKDENSQLKAID	T	136
9222	LOC_Os07g42950	TAGRVRLLLHRGTPCFRGYGRRDGE	T	81
9223	LOC_Os07g43316	EDAMETKHMDSKTIPDYLEGKSEVT	T	229
9224	LOC_Os07g43870	NTAMVELGDVQLTGHFAEQRFSKQP	T	198
9225	LOC_Os07g44200	AIFEIARNIWRPTNKHGRDGDSDDE	T	184
9226	LOC_Os07g44640	YDENMEMQQKRDTQVITVVHTKAEE	T	353
9228	LOC_Os07g45070	VQDLIQKHLAAVTTPQISLEPSNAN	T	1235
9229	LOC_Os07g45360	RQDAVDNLRAGKTWVLIATEVIARG	T	436
9231	LOC_Os07g46540	LVSKGEISSNTITALWDYFCFHING	T	658
9233	LOC_Os07g47700	ANLEVRIARIFNTYGPRMCIDDGRV	T	298
9236	LOC_Os07g49270	DEVVIFRDGTYLTLKEVFESLDLTG	T	404
9237	LOC_Os08g01100	KSGARTRKRVEATDSAVLKRARDGS	T	16
9239	LOC_Os08g02340	DDGIPITSEKIATLVKAANIKVEAY	T	29
9240	LOC_Os08g02700	AGVLAGIKVDKGTVELAGTDRETTT	T	108
9241	LOC_Os08g03360	NVPPSTSGAKDNTQSAVRLDQENDP	T	253
9242	LOC_Os08g03360	RFADTIFKAREKTLDQVAKKDPEKL	T	321

9243	LOC_Os08g03520	GGGGGGCYNCGETGHIARECPSKTY	T	185
9245	LOC_Os08g05180	EFRSLTQGSRSVTEYLHEFNRLARY	T	167
9246	LOC_Os08g05660	DIAALLPSKTHGTFLKMFLGPVNLR	T	91
9247	LOC_Os08g06140	SNRATEAGYWKATGKDRVIRSKGDK	T	96
9249	LOC_Os08g07830	DDVLSAAQTAAETAERAASAARAAA	T	468
9250	LOC_Os08g08080	EEPEDLSPPRRRTRHDSHEPKDKLP	T	301
9251	LOC_Os08g08240	SIPIVLDPRFKITFIEFRLKRAFGA	T	627
9252	LOC_Os08g08390	KVATQRKHGAPYTRRTDILWKIALF	T	345
9253	LOC_Os08g08550	LQEELGTRLNFSTAYHPQTDGQTER	T	1519
9254	LOC_Os08g08720	DNGLSILQYADDTIIFMEHDLEEAK	T	133
9255	LOC_Os08g08720	IPQVSSEENEMLTKEFSEQEVKHAI	T	20
9256	LOC_Os08g08820	SMEFQVPSPLVPTRESYFVRYCKNN	T	423
9257	LOC_Os08g09160	REEALERVEGSATSTSTGDGEGRRD	T	52
9258	LOC_Os08g09910	SLKKLTMVKCSITMDLSICAPNLEL	T	280
9259	LOC_Os08g09910	SRHLLDGMSRRTTTARWVPRDRPEP	T	25
9260	LOC_Os08g12140	IENIELAKFRSKTIDRWWGEWKLHL	T	392
9261	LOC_Os08g12680	VRASDGKGVVYQTRNKKEVALDMIN	T	1013
9262	LOC_Os08g12760	HIILENNDNKPVTNSRDTQEVKLEH	T	453
9263	LOC_Os08g13970	RSGESSHAVVEETPLEGGQAENVWR	T	166
9264	LOC_Os08g14050	FIMVVPTQPYPFTFFCSESDLVIQI	T	206
9265	LOC_Os08g14050	GSTRRRRSPSATTSAAQAQVGMAGE	T	121
9266	LOC_Os08g14230	RAVQYREIQGNETDKFLSYFRPCIM	T	107
9267	LOC_Os08g14340	RSTMEAELTALDTATVEAEWLRELL	T	1310
9271	LOC_Os08g16610	DIDEVNNDPMDVTEESSPFVSKNIT	T	254
9272	LOC_Os08g17120	AKEAIKLSDYERTLKKASSRKSKPV	T	511
9273	LOC_Os08g19310	AVETMKSNGIQPTNVTYGSLMYWMC	T	641
9277	LOC_Os08g23020	SINAVRDGPPETTTLTSTDTERGST	T	933
9278	LOC_Os08g23440	CKIQVFCIAEEDTDAEELKADVKKF	T	831
9279	LOC_Os08g24760	KLVEFGFTSSAETRSFAADIYAKVP	T	58
9280	LOC_Os08g24760	KNGSYRTVKNPQTVFIHPSSGLAQV	T	980
9281	LOC_Os08g25080	NKANDGEPSSAETEPSNGKTNINDT	T	135
9283	LOC_Os08g25670	PFQGKLYMLDQATAYGGPEVLQIDP	T	239
9284	LOC_Os08g27710	TTTTPTAAATKTTSDRPHRRRHQLL	T	28
9285	LOC_Os08g27870	LTRNVALIRDLNTNIARVVDLYANL	T	138
9286	LOC_Os08g28880	VIPTFDIKLLQPTIFSRFDAQKDAS	T	197
9288	LOC_Os08g29650	SDSRSPPPRRRSTSRSPRPRRHGRS	T	57
9289	LOC_Os08g29650	SKSKSPTPVRRSTSRSPPPKKSDSR	T	36
9291	LOC_Os08g31240	GVPYKASAFIIPTSTCLVELIETPS	T	774
9292	LOC_Os08g32050	RNLEPTRITSEVTRSWCPIHKTRKH	T	60
9294	LOC_Os08g33120	PSARTIAKSFSRTKDMTWRPDLFSD	T	67
9295	LOC_Os08g33190	VGGGDGVCVAPGTSVVVVEVATVSA	T	254
9296	LOC_Os08g33320	NKLQVCQAPFDSTIAGNIPDLSEDK	T	455
9297	LOC_Os08g33370	EEMVEYMEKVAKTVDVEELTVEERN	T	32

9298	LOC_Os08g34090	EIRACKERGIQRTSPTRDVYCPIHK	T	186
9299	LOC_Os08g34230	APSTLYSLPPTVTQEYENVFQDEIP	T	683
9300	LOC_Os08g34230	ELMRNSKSYNESTDKHLKTLSESLV	T	39
9302	LOC_Os08g36760	KKHVTRGSEKNSTNIIEWKKKTVES	T	479
9304	LOC_Os08g37280	SNQHEDDSTIDGTLASHAEIRVMVR	T	295
9307	LOC_Os08g38410	HEFNGKQVEIKRTIPKDSVQSKDFK	T	97
9308	LOC_Os08g39050	YGCPRTAKSLNATMKVLLRARLFDE	T	187
9310	LOC_Os08g39630	AAAAAAHFPDDPTMATQHHHQQQMM	T	92
9311	LOC_Os08g40150	AAAPPQSVRMAYTSDGTPVFAPVSA	T	38
9312	LOC_Os08g40230	SECTELMEAENPTDVDEFSKMENKS	T	240
9313	LOC_Os08g40830	SPGRRNDSVRFQTPSRNMTAYSGVQ	T	1426
9314	LOC_Os08g41260	SINPAEFHQVYTTGIEAAGGDDRVM	T	260
9319	LOC_Os08g42750	EPEIRMLLEAADTDGNGTLDCDEFV	T	451
9320	LOC_Os08g43060	VSPIRKFEHQEQTNEFKDEKVASGN	T	529
9321	LOC_Os08g43090	QQLQIQQQQSSQTTQQGQQQQPQKS	T	451
9322	LOC_Os08g43410	SRGFDCATHVKSTWVPAAKRRERQN	T	128
9323	LOC_Os08g43410	VSSSAVVMDPYPTPGPFGGAHFFHG	T	287
9324	LOC_Os08g43560	SDKDIVALSGGHTLGRAHPERSGFE	T	161
9325	LOC_Os08g44150	LTWVLMGMLAGYTSSRLYKMFKSGS	T	289
9326	LOC_Os08g44420	FLDEHTSAMEVVTKDAKRKWEMFAE	T	185
9328	LOC_Os08g44540	AKVAPNVKRAAPTDPVERRLLGKVN	T	78
9330	LOC_Os09g01540	PSDQPPTGVQSATAETATTQEPPTG	T	488
9331	LOC_Os09g02480	CYSFAHWILLVITPKWSTCHYLNSR	T	78
9332	LOC_Os09g03610	AMQQLQSPPQAQTHPAMQPVQQIPQ	T	681
9335	LOC_Os09g07300	LSVAGYEDCQVLTLNSRGEVTDRLA	T	1894
9336	LOC_Os09g07430	GTQDSKEFVRRCTSCQRQGGITTRD	T	590
9337	LOC_Os09g07690	PVYSCLPPVYQDTWNSLPMGEEAAQ	T	188
9339	LOC_Os09g07900	GALLFTTVGNNATFQPNPNSVYQTE	T	3357
9340	LOC_Os09g08420	LVMEFCPGGDLHTLRQKQPGKFFPE	T	266
9341	LOC_Os09g09360	GGTCRVAAVLVFTAWIALTALTRLL	T	17
9342	LOC_Os09g10340	SGFNIPDLFPTWTGILATVTGMKRS	T	224
9343	LOC_Os09g10710	SRWPEDNSFYEATITDYNPETDLYA	T	214
9346	LOC_Os09g11250	STTIFQRFGFSSTSPELSDKEENQR	T	76
9347	LOC_Os09g12130	RVQPEGGDERLGTSAPAPPRQRIVT	T	749
9349	LOC_Os09g13120	PETPLAWQGTRTTLRLTPFLLHSLR	T	126
9350	LOC_Os09g13630	DNNCSGNHVEGSTLQKLEGKCFWGK	T	172
9354	LOC_Os09g14960	RIKQEEAREKKETTLYKKYTELLLA	T	485
9355	LOC_Os09g14990	PAKDAECFFCKETGHWKRNCKKYLE	T	176
9356	LOC_Os09g15770	AAGSDEDHSNSPTRSHDGDNVAADE	T	219
9357	LOC_Os09g16330	LYGLVTSQFGDVTDTFDNGVRISDF	T	369
9360	LOC_Os09g16458	HNVSGIIKPHRMTLLLGPPGSGKTS	T	164
9361	LOC_Os09g16980	VLVIELLTRKRPTYRTDQGDSLVLH	T	526
9363	LOC_Os09g17730	RSELAQVQELYKTQVDKFVAEWCNS	T	344

9364	LOC_Os09g17850	YWTQDGGDADDQTVEQQPEDLKTQE	T	419
9366	LOC_Os09g19640	FRGAANSLSQLYTLAMGGQKLSFQA	T	37
9367	LOC_Os09g19750	EVTPFSPPPNSITASLIYYRSPQFV	T	82
9368	LOC_Os09g19910	VSMNKIETHKNETHGTSDDINENTP	T	86
9369	LOC_Os09g19952	PIKKNHVSYNNCTKYSKEIEIIFRS	T	95
9370	LOC_Os09g20060	IQWPKDLIEIRVTARPTTAPGGRPL	T	79
9371	LOC_Os09g20060	NDRQGMVNYIYDTLWSHRDKEYIMY	T	292
9372	LOC_Os09g20590	DMEPGPSAAHTSTTTTSKEDDSDF	T	299
9374	LOC_Os09g21110	VFANEINIAVIETEKSYNAFMFRDA	T	809
9375	LOC_Os09g22150	PETRTGAQVFELTKNIKVVFRKGKK	T	231
9376	LOC_Os09g23730	EGLLPPAHPSLLTAHLARMKQTGEL	T	68
9377	LOC_Os09g23790	LTPSDPEANPVLTRPGKEQGEEAPE	T	1225
9378	LOC_Os09g24250	PLFNILKKFDGETVTEVVRPSIARM	T	423
9380	LOC_Os09g24440	GYTEIKVQAENDTFALHADNMTIRN	T	58
9381	LOC_Os09g25370	GLGLSGMLASLATTGFQASNLGDAV	T	64
9382	LOC_Os09g26540	PVIAVARNFKSKTLLVRCGEMTRER	T	190
9384	LOC_Os09g26560	LYYNICLVTVHCTSNLPKKSFNDDT	T	229
9385	LOC_Os09g26800	WDQYGIAYGRPSTNIIIHNVTIRSM	T	338
9387	LOC_Os09g26970	NIGAEDWVDEANTQQLSQQQPSSPQ	T	1310
9388	LOC_Os09g27020	SPETPLAWQGTRTTLRLAPFLLHSL	T	115
9391	LOC_Os09g29500	RGPGGSLRRARVTSLVVAVVRTTVT	T	440
9393	LOC_Os09g29630	QTKRMCTANLIVTNHEAQNFPGCNL	T	254
9394	LOC_Os09g29750	LRMASAGAPQFGTMNRHNHVPVPEP	T	994
9396	LOC_Os09g30070	NENGNSPGQLQNTLDVVKTTAALPS	T	879
9398	LOC_Os09g30412	AEINQLLSLIINTFYSNKEIFLREL	T	24
9399	LOC_Os09g30439	APTIPGTAVPVATQRRHQQGDTAAA	T	798
9400	LOC_Os09g31470	MQSVRMAYTADGTPIFAPVNSAPAP	T	53
9401	LOC_Os09g31486	EGLIAVFDLGGGTFDVSILEISNGV	T	250
9402	LOC_Os09g32090	WERQIRIRGEGGTKFADVYYTSPTG	T	164
9403	LOC_Os09g32430	HDEVSRSKDGTDTEDATIDTDRSSE	T	151
9404	LOC_Os09g32540	LAEASVETKEKKTTAKERVLSFRRR	T	898
9405	LOC_Os09g32540	VSKDQLSGQPNMTNLSGESIVGKEV	T	273
9406	LOC_Os09g32650	KPLIKNKLLEEGTAVLYSEPEKKVM	T	511
9407	LOC_Os09g33450	RVPVDQGLQQTRTPARGFMSPNMGK	T	324
9408	LOC_Os09g33600	GADSQATDRDGRTALQYAIDSGTID	T	811
9409	LOC_Os09g33870	VDSSHYRRYCSPTQDDDSTVSTHRR	T	245
9410	LOC_Os09g33940	NSKEQEYNIKGSTQKKIKRDIKVLG	T	320
9412	LOC_Os09g33980	EKYHFLEAMSTITPAAHRGSIVHSR	T	625
9413	LOC_Os09g34060	EQQVHLQDALNDTLKSEVQRLKVAT	T	264
9414	LOC_Os09g34110	LEKKPENRGTMATIPRRKPPVDEKV	T	328
9415	LOC_Os09g35710	IDSSLQPNPSIPTAPPLVHVTDYDN	T	193
9416	LOC_Os09g35710	PSGNTESAQPVYTVDSPPSSSQPQG	T	315
9417	LOC_Os09g36270	MIPLARELLRNGTEVVLVANSLPAL	T	757

9419	LOC_Os09g36360	DAKLNPAPAPAPTPTPPPPGILESL	T	133
9420	LOC_Os09g37006	IVGLHQNTTTGSTDITDMWEPLEEG	T	83
9421	LOC_Os09g37170	DDGEPNPRRRPRTPPPLPRQSHRRE	T	29
9423	LOC_Os09g37860	FREHRFHTSLDKTRRFYPHVNNMDG	T	525
9425	LOC_Os09g38390	VSMKLYTEAIEDTITTEEAQEVFQL	T	391
9426	LOC_Os09g38580	PSIKNSDYNIRNTTFALVIMIQYIF	T	209
9428	LOC_Os09g38970	WLNNCVGRKNYFTFISLMAISLFWL	T	223
9433	LOC_Os09g39490	EFRNKNAARRSETTQKKAEKRRKID	T	135
9434	LOC_Os09g40040	NNSHFTLPHPVPTWPGREQGGEAPE	T	1033
9436	LOC_Os10g01320	VYYVSEALHDAKTRYPQIQKLLYAV	T	317
9437	LOC_Os10g01570	LPTMVLPKYPLPTHNVVVRGGAPNA	T	84
9438	LOC_Os10g02509	PGTHTLQMMQQQTEPQLQPPPPPQQ	T	154
9439	LOC_Os10g02584	INGIDLDLSRIPTPICSCTGAPQQC	T	250
9440	LOC_Os10g02630	RIGGENAEQKLSTRDQQHAGRPRSE	T	248
9441	LOC_Os10g03130	HEGICGSHASGRTMVGKAFRQVFFW	T	380
9442	LOC_Os10g03830	ATVDGVKVDGLDTNLAVMKELLSPV	T	497
9444	LOC_Os10g07180	AAQNSGKHIHMITRAQRVVMKRLGI	T	493
9445	LOC_Os10g07180	SRSLGKALLQTMTSYLLTAAPITPS	T	202
9446	LOC_Os10g07270	SSSIGDEPELQRTLREGDDYALVPQ	T	104
9447	LOC_Os10g07300	ISEVLSESKTRYTQIQKLRHAIFIT	T	145
9449	LOC_Os10g07510	QSELNYLQGHLSTMELPSPPPYVAG	T	136
9450	LOC_Os10g08022	GVILFDETLYQKTKDGKPFVDVLKE	T	83
9451	LOC_Os10g12174	CYSMTINKSQGQTLQRVGVYLKKPV	T	1240
9452	LOC_Os10g12410	RAHPEGSMIEEYTTEEVVECCIDYM	T	581
9453	LOC_Os10g12808	EELKEKGYIRPSTSPWGAPVIFVEK	T	936
9455	LOC_Os10g14120	VQRGGLRKTGHDTLIHKKDFSVKQQ	T	686
9456	LOC_Os10g14290	DLESGEDSSDSVTSDAGSAKAAPDD	T	50
9457	LOC_Os10g16480	AQFQGVVDCARRTVTLYRGPEQPVV	T	805
9459	LOC_Os10g17980	TKALGNKEHRGRTQGVGSSVPWKYG	T	351
9461	LOC_Os10g20180	VYYVSEALHDAKTRYPQIQKLLYTV	T	235
9463	LOC_Os10g20480	YTIPHPPNDEILTLGAARGTFIQWP	T	427
9465	LOC_Os10g20600	NHFVTMPKADTRTPPAFASDPLKRV	T	152
9466	LOC_Os10g21310	GELNGVSYSDPATVKKYARHSQLGE	T	416
9467	LOC_Os10g21310	VYWDLEIFCDERTGKPSLDLPKIFG	T	128
9468	LOC_Os10g21840	RFNECRNTIPEITDSSVIRTFKSGV	T	349
9469	LOC_Os10g22070	KLLGSTNPSPFVTRVELALALRGLT	T	29
9470	LOC_Os10g24120	VEDRVDTPGYTPTPSPGHNETGVES	T	437
9471	LOC_Os10g25110	SSGKMFVRDSAGTPFVRNSGKQKRS	T	797
9474	LOC_Os10g26600	KGSKVKYELDKKTGMIKVDRVLYSS	T	61
9475	LOC_Os10g26670	RSNFTLLAPVKVTIPASGGDDDDDD	T	266
9476	LOC_Os10g28200	APTMKLKDGLRFTYFWIKEQIEKEK	T	333
9478	LOC_Os10g30350	LGPWTTLAEFMATDDFRYLTKSCPG	T	307
9479	LOC_Os10g30580	ANFISVKGPELLTMWFGESEANVRE	T	555

9482	LOC_Os10g32920	LPVAATVNCADNTGAKNLYIISVKG	T	32
9483	LOC_Os10g32960	KTMQRVLLNCMYTSLDYAQTGLIAA	T	279
9484	LOC_Os10g33680	EALYSQWLALPETSKLVNSLIEDAK	T	48
9486	LOC_Os10g34380	RESKDITSTSRWTKVKENFRSDARY	T	845
9487	LOC_Os10g34400	LERRDRFSFSFYTEQYSKRQDVEKV	T	502
9488	LOC_Os10g34580	KPMEEDVKVIEKTATMRGFKVEGED	T	420
9489	LOC_Os10g34820	PNRHLISSCQGSTPKQGTLLHSPLM	T	360
9490	LOC_Os10g35220	RGGDIVKALRDTTKAKIRVADSIPG	T	75
9491	LOC_Os10g35280	SKNLLTEKKKTKTPKEKSKKRAHNH	T	652
9492	LOC_Os10g35580	ESSKGGKETNRRTSVPSQAPAKSSS	T	145
9494	LOC_Os10g35870	TAAAAEPEKAPATEEKPREVSSEEV	T	201
9495	LOC_Os10g37230	ACVKHEYAPAEATKMDGAIQTVYPR	T	130
9498	LOC_Os10g37480	RNVVEIKGRFSVTSENVDLAKVQEV	T	565
9499	LOC_Os10g37630	EKYSLVSNFPRVTYGPEKLSQTLEE	T	439
9502	LOC_Os10g38850	AVDSRFTRAWVDTDTIFIDGVKDPL	T	1679
9503	LOC_Os10g39140	AAEAEQQHQLLSTAVHDTMPGKYVR	T	14
9504	LOC_Os10g39440	TLVPLYISETAPTDIRGLLNTLPQF	T	127
9505	LOC_Os10g39620	TVLYLTIWVDVHTYEIIGSYRIEYG	T	620
9506	LOC_Os10g40540	TFRIVTDRTVFATPEVHIGFHPDAA	T	177
9510	LOC_Os10g41440	LYGRDPALSTLRTSGIHGGGPGGPL	T	315
9511	LOC_Os10g41790	AHTNAQMNKLLLTMAEWYRYADNRK	T	539
9512	LOC_Os10g42110	AMQAQVCMPEWPTAFYLQALALSKL	T	474
9513	LOC_Os10g42196	LSASHGLSPKRPTHEFGNAQNNLAH	T	290
9514	LOC_Os10g42196	PLSLSLGLPGVVTSNQALEMKQFLP	T	1525
9515	LOC_Os10g42210	DGRQRRGARGDGTVARRRDGDGDGA	T	132
9516	LOC_Os10g42439	DRAAPEGNATEFTLTLRTDARGKFK	T	101
9517	LOC_Os10g42724	AEPEAEPAMIPVTVEPENSPRTKDG	T	331
9518	LOC_Os11g01300	SGGGGSNMLRFYTDEAPGLRLSPTM	T	63
9520	LOC_Os11g01920	LPSELTLRSPRATMYCEADQDQLRR	T	1891
9522	LOC_Os11g02190	LDLDSGFISSDITKETHTKPSPIES	T	604
9523	LOC_Os11g02720	PSRRCAVVRAAHTNPWRHRVSRCSG	T	14
9524	LOC_Os11g04070	QVLNIPTKINKGTVEIITPVELIKK	T	154
9526	LOC_Os11g06750	KKYASVVRVIVHTQIRKMKGLKQKK	T	166
9529	LOC_Os11g07440	ILLRAYTKYTADTSLAESPECQNCM	T	221
9530	LOC_Os11g08390	LLDLDLPPASGWTMELVSQLINVWS	T	382
9531	LOC_Os11g08460	KMRETAEVYLGKTVTNAVITVPVYF	T	153
9532	LOC_Os11g08460	RETAEVYLGKTVTNAVITVPVYFNN	T	155
9533	LOC_Os11g08470	AEDMGTGSKNNNTITNHSGRLKKED	T	523
9536	LOC_Os11g09329	SKVQILTLYVLETLSKNCGDVVYQQ	T	66
9537	LOC_Os11g09420	PFTWTEEAENALTQLKAYLSSPPVM	T	856
9538	LOC_Os11g10060	IHGRLVCWLDAATSKLEKAEQQQQH	T	675
9539	LOC_Os11g10060	VVQKYQSAVQNSTNLSTQDMQNNCN	T	495
9540	LOC_Os11g10490	AEKGKRPADEAGTSSGSASKKSRSD	T	299

9542	LOC_Os11g10510	PAKFEQAKKFGCTDFVNPKDHSKPV	T	241
9543	LOC_Os11g10710	HVWNITTLDVHPTEPYLLSIGSQDQ	T	455
9544	LOC_Os11g11710	AWFVARDGAKDCTAAVDECKDKVDQ	T	101
9545	LOC_Os11g11710	EDEKLLAIHASLTQLLRGPTGTRRP	T	155
9546	LOC_Os11g12810	TDVPHIYRLAAKTKGVFINPALVEP	T	631
9549	LOC_Os11g15750	RGKRSRNLMPKETYYIAALNDDGKP	T	619
9550	LOC_Os11g16370	NMNGSVSGEDQPTTSGVENGHQEPF	T	42
9551	LOC_Os11g16400	PADQDKQHPKRETKTKITSNSKTKA	T	188
9552	LOC_Os11g16400	QQTVDEFLALEATLARSAAAGHAED	T	324
9554	LOC_Os11g16730	SRDEWDKFVAKMTTPEALERRKKMS	T	181
9555	LOC_Os11g16750	LKSRTDLVNLDITNELHPKELPNGK	T	598
9556	LOC_Os11g17890	DTSSVTCETISVTPMPKTGIVSNFL	T	549
9560	LOC_Os11g20290	LPVTFGGSANFRTERVNFDVADLSL	T	574
9562	LOC_Os11g22180	TVGRRIGQALPLTDVIGPLVDHQAA	T	51
9563	LOC_Os11g23750	RRTPASCGSTGFTKVRKDTTYTCKI	T	609
9565	LOC_Os11g28270	FGPNCKFDHPMGTVMYGLATSPTGD	T	384
9567	LOC_Os11g29000	GQGHYKMDNLVHTFAKNVVESESLV	T	156
9569	LOC_Os11g30970	AWVEGQAFAPGKTVVPKPVDNRTVV	T	194
9572	LOC_Os11g33120	LLRAEYDRDGAMTNPSFPKVLIDGP	T	725
9573	LOC_Os11g33330	FGDKGYLAFEHITWAPYQTKLIDTT	T	642
9574	LOC_Os11g33340	HQGFPLKAHSSPTTRMIISMSSRWL	T	689
9575	LOC_Os11g34200	ETIHGTESVPPSTHPPAEAPSAAEI	T	194
9576	LOC_Os11g34200	FADQGEALPGEETVPETIHGTESVP	T	179
9577	LOC_Os11g34450	KEEGRGNDAHAATIRSYRGKIEAEL	T	91
9578	LOC_Os11g34600	MVLRPSQPIIGVTPGHTWPLGHINL	T	663
9579	LOC_Os11g35080	KKEAAARGAGKKTTKKPAAAAAAAT	T	94
9580	LOC_Os11g35450	IAGLIPRMPRNLTTLDLSNNSLSGP	T	565
9581	LOC_Os11g35590	PEHLQQIAISIETLLDVNELSLEEV	T	275
9582	LOC_Os11g35590	SSRVDANGRLLFTEEEWLAKFRKAA	T	318
9583	LOC_Os11g36060	QPHKVDLSSPDRTIIVQIAKTICMI	T	339
9585	LOC_Os11g36340	HGVEDATGPDRATTLVASNEVPVQG	T	418
9586	LOC_Os11g36340	TKIPSQASATAKTETKPLVTKQKTS	T	358
9587	LOC_Os11g36390	KYFASKTEKEEDTSAGKGTGRGLPK	T	73
9588	LOC_Os11g36880	SDFAGWWLAARQTVAKIDRKTFDAG	T	948
9589	LOC_Os11g37090	ICEITRVPGELITVACNMHGTRTVQ	T	501
9590	LOC_Os11g37100	NKAAPGLPSGEFTISLYEVLARVHG	T	64
9591	LOC_Os11g37660	AAKGFASDLLVATALIGMYAEAGDM	T	158
9592	LOC_Os11g37890	TDRFEMWGDGLQTRSFTFIDECVEG	T	236
9593	LOC_Os11g37950	NKPLSWRQKYGWTAFCGPVGPRGQA	T	73
9594	LOC_Os11g37990	IAKIALKRGVQLTTSERMAEDIKKL	T	435
9597	LOC_Os11g38620	IVRTANLYWNSMTQEQRGEFLSVRF	T	353
9598	LOC_Os11g40090	KHSKKKREAIDATMAAASPKKKEKK	T	57
9600	LOC_Os11g40140	GCAMSGLIADARTLVEHARVETQNH	T	87

9602	LOC_Os11g41890	RTKKIFVGGLASTVTEADFRKYFEQ	T	116
9604	LOC_Os11g43590	VKGDIPVARSGHTVIRAGPVLILFG	T	211
9605	LOC_Os11g43610	QWGGRELEQQIRTSITLRKLLPSPN	T	247
9606	LOC_Os11g46200	AGGDGGPFDELKTRVEDVSKRFSDS	T	109
9607	LOC_Os11g46240	QVSQSQQERPGQTVYPQASHLGHQQ	T	101
9609	LOC_Os11g47240	ELTFLGLGYNQLTGPVPSTFGNIRP	T	368
9610	LOC_Os11g47330	VAGGGASVIYADTVGDLGYASELGN	T	288
9612	LOC_Os11g47760	AEDKTTGQKNKITITNDKGRLSKEE	T	508
9613	LOC_Os11g47760	AGGVMTVLIPRNTTIPTKKEQVFST	T	424
9614	LOC_Os11g48030	TREGEVRFPEISTPILEKICQYFYW	T	80
9615	LOC_Os12g01260	VLVMSLCFIGFVTALHVFGKLYRSR	T	88
9616	LOC_Os12g01922	NKSYTQYIPLPITDLDSWLKTPSIY	T	230
9617	LOC_Os12g01940	VTEVDDDTTMEGTSSAPYPLLQVDN	T	249
9618	LOC_Os12g03880	NGYKNVLAVAVETEYSYPHADKIKE	T	258
9620	LOC_Os12g04000	GRAAAMPPASVMTRLILIMVWRKLI	T	230
9621	LOC_Os12g04050	GSVNPAEFLQVYTTGIEAAGGDDRV	T	146
9622	LOC_Os12g05000	TTTMRDGKLWKTTTIKRRPGTGAPS	T	217
9624	LOC_Os12g05110	FAGLAKAVKPGDTIFVGQYLFTGSE	T	147
9625	LOC_Os12g05110	KPAVVTRVVDSMTDNLRPTRAEATD	T	320
9626	LOC_Os12g05420	GPKGTKWIFVMSTTKKLYAGKKERG	T	225
9628	LOC_Os12g05930	RLYGQIPHKLMMTSLQFLDLSSNGD	T	76
9629	LOC_Os12g06380	DSEALVGYFKRRTIEQGMFYWDVLV	T	257
9632	LOC_Os12g06780	GLLKEIHVLEKSTQKLTDAIDAAPI	T	313
9633	LOC_Os12g06890	GASSTPAFGATTTPAFGTTTPAFGS	T	195
9635	LOC_Os12g06980	VSAMLDKESNGLTTEMMPEAHIRRS	T	576
9636	LOC_Os12g07230	FTFFDKDGSGFITIDELSQACEQFG	T	443
9637	LOC_Os12g07260	EKAEEIFDNMFKTWKTLSSKYYNAM	T	443
9638	LOC_Os12g07300	FCMGLQDKDIGATKEELGIEREQVL	T	514
9639	LOC_Os12g07590	SYHLDETVKTLRTQRVGMVQTEKQY	T	297
9644	LOC_Os12g10410	ENPPDHVIFPRLTYLDLSDLPELSD	T	857
9646	LOC_Os12g11600	LLIKVASGMAIPTDISGTYHCRPIP	T	394
9647	LOC_Os12g11750	EVEEERETALIATAVFNEARDDLRL	T	543
9648	LOC_Os12g13150	KVRISPQSKHIATLDLNGSVNIFVL	T	303
9649	LOC_Os12g13340	KTNAVAPSIGSGTTVGKVEFNEFIN	T	66
9651	LOC_Os12g14984	WSRFTNLVLSGPTLSIPDYVLLQHF	T	464
9653	LOC_Os12g16060	DKLGFPLSQELATDLILQSLPPSFE	T	212
9654	LOC_Os12g16430	YAYGIDTNWYVDTGATDHITGQLDK	T	326
9655	LOC_Os12g18120	DARAPPKSDPGATPIGSISPSSAAP	T	17
9656	LOC_Os12g18630	LPGWLSRGLVGRTLADPSFPHKIAF	T	156
9659	LOC_Os12g19040	KKKLKVANTGKHTGCSNEDKAHQDQ	T	70
9660	LOC_Os12g19290	MLSSKDTNFLNFTYKNLELSDDPEH	T	486
9662	LOC_Os12g21798	KNVLTNFWGMSFTTDKLRSLVKKWQ	T	106
9663	LOC_Os12g21890	AAGREAQSSIFSTSGLSSWAKNLKI	T	81

9664	LOC_Os12g21940	ELHEGIIVWHIATDIFIAQRKADDQ	T	546
9665	LOC_Os12g22000	NEEVAEAADDIPTIDPDVEEFLANE	T	482
9666	LOC_Os12g22530	HQNVVLDICQETTFFLHDASLEKEN	T	63
9667	LOC_Os12g23520	FFFFRADCTISKTRMKFSWIHMFFM	T	677
9669	LOC_Os12g25690	DKAQISIYDPQVTEDQIQRDLAMSK	T	368
9670	LOC_Os12g25710	GGGGKKKEVKKETKLGMAYKKDDNF	T	25
9672	LOC_Os12g26240	ASAPATPRQRMITGDEVPVKEVALG	T	780
9673	LOC_Os12g26240	ATCQARWSEIALTFDQTDHPPCVAR	T	595
9676	LOC_Os12g28260	QYIPRLVRFYPITSELKRTTGVFAE	T	263
9678	LOC_Os12g29660	AHKDEIYSRPKRTWFATEKEKKLLA	T	626
9679	LOC_Os12g31050	TTSLQQIVQKENTREFVPRQQHDEL	T	352
9680	LOC_Os12g31350	QKNLLYLAAIADTQPQTTISRPQMV	T	69
9681	LOC_Os12g32374	FINKHMLYHYQDTGHCLALSFSDLS	T	119
9682	LOC_Os12g32410	RAFKSGVRDRYTTQELATRRITTTR	T	435
9683	LOC_Os12g32440	PVSPHVIKIIGYTESLDKLGFPLSR	T	223
9685	LOC_Os12g36180	EIWLTVSEIPLFTQPTSAPPPSRTP	T	378
9686	LOC_Os12g36510	IVHQALKKYQDYTGQLLSPAKCSLL	T	152
9687	LOC_Os12g37060	ALGFDLSNLLVFTPMTIEMMKKRHK	T	138
9688	LOC_Os12g37430	KSTSYEIDNDFVTSRSKRDGNKRSR	T	324
9689	LOC_Os12g37560	DMSAPFSHYYIYTGHNSYLTGNQLN	T	125
9690	LOC_Os12g37560	GVRADCVMKKTRTIEDQWVPMWDEE	T	510
9692	LOC_Os12g37720	VCHSKAWDSKIVTSAWWVYPYQVSK	T	658
9695	LOC_Os12g37870	APLIATLPLEVRTKIGDFIRGVYSV	T	176
9696	LOC_Os12g37890	QFLAVNHINCLATLEGFQSCANLLV	T	1298
9697	LOC_Os12g38440	NVQSPRCLESMRTPDVKKRSVQKQK	T	163
9698	LOC_Os12g39090	SPLGKSPLGHQGTGEFVSAGSKAPL	T	149
9702	LOC_Os12g41180	YNRTYRSELPSKTTPNKIARPARRA	T	405
9703	LOC_Os12g41290	DSSGTSPGDTLSTTYFPRLDGTDYK	T	614
9705	LOC_Os12g42550	VVSVEMPAPEGWTKKFTPQRGGRFE	T	40
9708	LOC_Os12g44150	RASRTENQDAIDTAIVGMLADPKEA	T	380
9710	LOC_Os12g44360	SRHLLRGTRVPYTVALLVLGVALGS	T	37
9713	LOC_Osm1g00580	IIERKSVHEPMQTGLKAVDSLVPIG	T	150
9714	LOC_Osm1g00590	PEKRVVSEPIEMTQEFRYFDFASPW	T	172
9716	LOC_Osp1g00600	SSPRGWFTFGHATFALLFFFGHIWH	T	457
9717	LOC_Osp1g01070	FGILLFMVLISATRDFRERTKSKLV	T	34
9719	LOC_Os01g01689	LQKTGCSHVLDVYCQGLSNQKSCLQ	Y	1849
9720	LOC_Os01g01800	NKSNLDIVTQTPYPHIQSPMSSPVQ	Y	392
9721	LOC_Os01g02700	KCSQEMNSSNVMYRPVACRSGNSSF	Y	158
9722	LOC_Os01g04650	AAAAAAQQPPAGYVYAQMHAPPPQQ	Y	444
9723	LOC_Os01g06290	RGRSYSRSRSRSYSRSQSPRRDSRN	Y	183
9724	LOC_Os01g08380	PRGGAGFYGNCYYIMRVSAPAGKVA	Y	310
9725	LOC_Os01g08380	SDWTRLGFAEVDYGWGPPAHVVPLT	Y	382
9726	LOC_Os01g08420	DITLPGLAELRGYWRGSLDASGGGN	Y	278

9727	LOC_Os01g08760	CHNDLQYGNIMIYEETRQVTLIDYE	Y	217
9728	LOC_Os01g08960	LLQTCLDELATRYPATKFVKIISTD	Y	147
9729	LOC_Os01g09620	EEAAAMAAAVDAYACDEFRMYEFKV	Y	72
9730	LOC_Os01g10820	YDPSTGIYGMDFYVVLERAGYRVAR	Y	130
9731	LOC_Os01g10840	PTREEIRCMNPNYTEFKFPQIKAHP	Y	297
9732	LOC_Os01g11140	EKVASTAATNNSYSIDTNWYIDSAA	Y	260
9733	LOC_Os01g11960	CIPGALCVMFFGYSNSGLRDYGWVK	Y	208
9734	LOC_Os01g12390	AYYYSKDEKLLVYDFYSRGSVSNML	Y	411
9735	LOC_Os01g12650	ATAIWILFELLGYHLLTFVCHGLIF	Y	125
9736	LOC_Os01g12660	SICLSDTMVLGRYIEEIVVSAVSYH	Y	388
9737	LOC_Os01g12770	DVVVDLSDLLIAYSNTVLTRIAFGD	Y	197
9738	LOC_Os01g16020	ERIRLPDEAMAYYDDLAEMYVGGDV	Y	224
9739	LOC_Os01g16030	KLGLHSLRQRHWYIQSTCATTGEGL	Y	154
9740	LOC_Os01g16110	INEGLYYYENHAYNQRSSQAGTIDI	Y	615
9741	LOC_Os01g16330	IFGVNFSDKSFIYLAGLQLLLSSGK	Y	158
9742	LOC_Os01g16850	GSDTVEEVTVFEYYRKNWKIDLKGS	Y	1759
9743	LOC_Os01g16850	LDDRSTPGVFIGYAEGSKAYRILDP	Y	1004
9744	LOC_Os01g17320	HKLSYKNKIKPLYTTYLMLLDSYSK	Y	520
9745	LOC_Os01g19490	EWMVNTKGMNMSYTNHAIRLDLIYK	Y	124
9746	LOC_Os01g19894	STDPPQGEVGMDYWHAAPQVTQPTQ	Y	1896
9748	LOC_Os01g20950	EVYEQLRQLVSTYPTVPSGLDTPYY	Y	32
9749	LOC_Os01g20950	YPTVPSGLDTPYYRHPDGWYTFLPA	Y	44
9750	LOC_Os01g21450	TSLYIKKHEILSYFFDKQNKGAETD	Y	281
9751	LOC_Os01g21960	RFSKDNILGEGGYGVVYRGQLINGT	Y	204
9752	LOC_Os01g23530	NDKDLNLVSLRFYLLRKNGYNMSSG	Y	122
9753	LOC_Os01g23590	YIRKEGLKQLIIYANESFWDQLMKF	Y	636
9754	LOC_Os01g23640	PTPGLAVSETGRYQNLHVPVKLMEC	Y	340
9755	LOC_Os01g25610	LPLILIIRNRLKYALTYREVISILM	Y	54
9756	LOC_Os01g25610	VRSVQFGQKGIPYLNTYDGRTIRYP	Y	138
9757	LOC_Os01g26970	AMAVSASEGVGQYDLLIVGPGVLGR	Y	94
9758	LOC_Os01g27040	IAMQLRKCCNHPYLFQGAEPGPPYT	Y	510
9759	LOC_Os01g27790	KTAFITPIGTYCYTTMPFGLKNAGP	Y	927
9760	LOC_Os01g28560	APAAQRREHERQYEALDAHTIITKL	Y	69
9761	LOC_Os01g28660	SGQLIGSGFRHNYKVALSVRTGTDE	Y	324
9762	LOC_Os01g31510	NPVSPHVIKMIGYTESLDKLGFPFS	Y	132
9763	LOC_Os01g31629	QHHVQQQQYVDPYRTMVLSPQPDHL	Y	15
9764	LOC_Os01g32720	TQAGYGQVKAKEYHRSDSLPVKKQA	Y	654
9765	LOC_Os01g33000	ESGDLHGRLLFQYLEFDSPFCREPL	Y	218
9766	LOC_Os01g33440	INWTQTAFIPGRYILDGCVILHEVL	Y	139
9767	LOC_Os01g34030	AAWLAVSELGEEYDTVWDRLRGLRS	Y	69
9768	LOC_Os01g34290	AAMGTACKDLHLYYMEKSNVRKPSK	Y	1234
9769	LOC_Os01g34330	VLLTPEDSKEKPYVAILKDITETEG	Y	185
9770	LOC_Os01g34780	RDYCAQVKDSSVYLAVASNISGSMP	Y	128

9771	LOC_Os01g35470	PTSKFFRGILNFYGISLHHLNPNSI	Y	81
9772	LOC_Os01g35730	DTHEIVVFSHFFYGGFALPTSKFFR	Y	63
9773	LOC_Os01g36790	HGHLTEKADVYSYGILVLELVTGQR	Y	527
9774	LOC_Os01g37790	ATKRKRSNIGQAYENSRKKDRRRPL	Y	293
9775	LOC_Os01g37832	PENFGGDTTKIYYIGLRGEATQNKR	Y	161
9776	LOC_Os01g37832	WELAENLQGVLEYQTRYSRFQGVAN	Y	131
9777	LOC_Os01g40050	VRNFVQLCLEGYYDGTLFHRVIKSF	Y	49
9778	LOC_Os01g40370	TNNLSQALRYGIYSNDDMEENQGTL	Y	95
9779	LOC_Os01g40750	ETTDFCTTKSLCYHREYKTNDTSET	Y	144
9780	LOC_Os01g40750	FAVRDYLDPLRNYVFNRTRDDPVIV	Y	202
9781	LOC_Os01g40990	YGSHNFGSDTWQYCLRLRDKKLAAQ	Y	1344
9782	LOC_Os01g41640	SADDEILVRDVLYDALILVDYSFIN	Y	285
9783	LOC_Os01g42410	LWYLFFMYFTLLYFTFYGMMAVGLT	Y	1317
9784	LOC_Os01g43340	RERVARKLLQVDYVSTNDQVADGFT	Y	1275
9785	LOC_Os01g43410	PGEHFKDLVGSAYYVAPEVLKRNYG	Y	232
9786	LOC_Os01g43420	QLYSASEYCEKSYLHSEQKQMVLDN	Y	69
9788	LOC_Os01g46750	GEYVAVENLENIYGLVSAIDSIWVY	Y	536
9789	LOC_Os01g46750	SGAAPLATHVEEYLRVVTCAHVLQG	Y	402
9790	LOC_Os01g46840	KMSMVESSSKCGYEKVGADVQKCSP	Y	481
9791	LOC_Os01g46980	KEKQVEVRKKIEYSMQLNASRIKVL	Y	70
9792	LOC_Os01g47490	RTPHITKDHDRAYFDSADWALGKQG	Y	69
9793	LOC_Os01g48640	TISLVAYVENDRYVYSEKRPSCPPP	Y	219
9794	LOC_Os01g49200	TEPNTLSTDFLNYVESEVLRLEQLK	Y	298
9795	LOC_Os01g49250	SEYGSTLDDDQRYADAAEVLAAAAA	Y	76
9796	LOC_Os01g49470	TPSCNGLSMAKYYCNICKFFDDERT	Y	1059
9797	LOC_Os01g49529	LNIAVESAAALTYLHAIEPPIVHRD	Y	497
9798	LOC_Os01g50320	LLDRVPPRPRRSYSAALLSAPSLDG	Y	61
9799	LOC_Os01g51200	TKHPQLLYESKIYRILQGGTGIPNV	Y	56
9800	LOC_Os01g51230	GNGATRSNHSWSYLEGWMATKPWES	Y	247
9801	LOC_Os01g51390	PIALDVLSDILQYPCFPANALQRER	Y	164
9802	LOC_Os01g52304	LEWQSSIPPYYSYIVQYGILARQPL	Y	344
9803	LOC_Os01g52950	AVQRRDDESLRSYIQRFCQVRNTIP	Y	337
9804	LOC_Os01g54590	GQERYRAITSAYYRGAVGALLVYDV	Y	88
9805	LOC_Os01g55040	HRLDFFRSLSVFYTTVGFYFNTMMV	Y	1358
9806	LOC_Os01g55260	VPYDTLVEKTEGYSGSDIRLVCKEA	Y	304
9807	LOC_Os01g55490	AALQQKINKFLAYKRAGKSFNSEVR	Y	253
9808	LOC_Os01g56300	SYHMAGLSHGLPYGGSVSFGSPNLP	Y	246
9809	LOC_Os01g56490	VSDEQFAKWKFAYIAHNRLAGEYFQ	Y	1047
9810	LOC_Os01g57630	ITERNMVPDAKNYNAKLRGLVAQGR	Y	272
9811	LOC_Os01g58620	VNLLGLLVQSVFYYACKAFHNQQID	Y	288
9812	LOC_Os01g58870	LYTSSVLPIGALYSLSLWFSNSAYI	Y	109
9813	LOC_Os01g59760	AHHHGQQGVQGAYFPNRLVPQPLNV	Y	207
9814	LOC_Os01g59990	KTELCRFSGQKIYPGKGIRFIRADS	Y	16

9815	LOC_Os01g60190	ADRMVMLAKALEYADFDKFDRVRVP	Y	299
9816	LOC_Os01g61660	YFQAHRVTVVTSYPFGQILHNREGT	Y	1270
9817	LOC_Os01g61930	GFQNQYANLAPTYQPGTTYYSQLPL	Y	350
9818	LOC_Os01g62310	REHGHIEGKNVFYWFQNHKARQRQK	Y	91
9819	LOC_Os01g62310	SFMPVATNNASYYPQQQTPLLYPGM	Y	180
9820	LOC_Os01g62890	YSKWTSRLKCNLYYYRTNYFILIMF	Y	52
9821	LOC_Os01g63170	WRKFVSKVREKGYSLAIVVQKTKCV	Y	102
9822	LOC_Os01g64250	CPVAWRVRVALLYKAAAPVHFTPSE	Y	83
9823	LOC_Os01g64280	EESSPEYEKLKNYIFELENHLAEAQ	Y	192
9824	LOC_Os01g64670	DKIIAVCVDDPEYRHYNDLSELSPH	Y	149
9825	LOC_Os01g65260	GVVGDPDATSLCYLGLQKLQHRGEE	Y	102
9826	LOC_Os01g66350	SLVTSFAFLSCGYLWSSYHEVRSVV	Y	315
9827	LOC_Os01g67126	RRVLTLRGLDQEYEGNVEATGEDYY	Y	117
9828	LOC_Os01g67134	YEGNIEATGEDYYVEPADERRPFRA	Y	132
9829	LOC_Os01g67170	RRKRDRKRKRRRYDSDEDDLDQLLE	Y	446
9830	LOC_Os01g68120	SPGFVPNEEINEYHVGTTGAKVTAD	Y	519
9831	LOC_Os01g69910	SSIIDSPVARQQYRRMLKMHKQNKD	Y	834
9832	LOC_Os01g70160	RSESTKSLSAKSYNSSFAVVTSEDE	Y	164
9833	LOC_Os01g70810	STVLQTINRLRFYRTQDISSKARNL	Y	340
9834	LOC_Os01g71540	DLDKADEWVKIKYAWMAKSLTNDII	Y	66
9835	LOC_Os01g73300	NGLCAIAFTDDHYPVRSAFSLLNTV	Y	101
9836	LOC_Os01g73300	SAFSLLNTVLEEYHKTFGERWRTAK	Y	117
9837	LOC_Os01g73950	HCAAGEVEKALQYFTEMVEKNLEAD	Y	406
9838	LOC_Os01g74020	LTRENVASHLQKYRLYVKRMQGLSN	Y	169
9839	LOC_Os01g74260	RTLVGKVFRQGFYWPTALNDAVDLV	Y	1569
9840	LOC_Os01g74370	KQMKQVVVGVVYYLCRQDGQLDHPH	Y	44
9841	LOC_Os01g74470	YSFTNETLAKYSYATSLQATLRYGI	Y	291
9843	LOC_Os02g01110	PQGHRRNENEVRYPVNDYQRPLQQN	Y	403
9844	LOC_Os02g01170	LKEGEENTIVNIYNLEEYVTLVVDA	Y	634
9845	LOC_Os02g01390	ALVKFMAVVHYAYLQMKMPGPGGPI	Y	721
9846	LOC_Os02g01490	SVSTWGQGGTSEYPPNMVFYAEYPG	Y	272
9847	LOC_Os02g01560	QFGQKGIPYLNTYDGRTIRYPDPII	Y	142
9848	LOC_Os02g02290	RGVHLAFQGDGDYDSQFKSEQAFAD	Y	1805
9849	LOC_Os02g02410	WLDENQTAEKEEYEEKLKEVEAVCN	Y	622
9851	LOC_Os02g02870	RGGGGSGGGGRSYGGSWGGGRRSGG	Y	107
9852	LOC_Os02g03080	AKVALRRVELVYYKPQEVYDSMRKL	Y	439
9853	LOC_Os02g03080	VRVNESSTVPNMYIKALVLLEDFLA	Y	121
9854	LOC_Os02g03890	ATQVGTYFLRNYYNLLQQSPDVVHQ	Y	21
9855	LOC_Os02g03890	GVAIRSRKETGGYYAFVEFEELSGV	Y	353
9856	LOC_Os02g04450	GDNGTTIKTEASYSGNSDFGFCNES	Y	205
9857	LOC_Os02g04660	DPLPEQERFEINYRDFLQSPLQPLM	Y	317
9858	LOC_Os02g05330	DMGLQENLLRGIYAYGFEKPSAIQQ	Y	57
9859	LOC_Os02g06430	KEDVAEKSDGKGYHVHRSRMAGGLD	Y	319

9861	LOC_Os02g07210	HYEGYVPMEYKVYLKKMKRSGEWGD	Y	141
9862	LOC_Os02g09170	EEVITGYDETDLYKTWLRANAMRSP	Y	106
9864	LOC_Os02g10650	GQSTYDKASVHYYVQSHIQINEYRD	Y	122
9865	LOC_Os02g11760	FLECAVWICMTYYVMGFDPNIERFF	Y	626
9866	LOC_Os02g12310	EVPTAVKRRFVFYLGHHQPSGSNNN	Y	119
9867	LOC_Os02g12310	NVDPWQLPAMAMYGSDHDRYFFTMA	Y	63
9868	LOC_Os02g12550	IVNAWAIGRDPRYWDDAEEFKPKRF	Y	412
9869	LOC_Os02g12610	AGDGDDGGEGVKYLRMERRMGKFMR	Y	108
9870	LOC_Os02g12850	STAYGYGRGGYGYGGNAGFGSGYGG	Y	311
9871	LOC_Os02g13130	SGGQQQAYDYSSYYQTQGQQQGYSQ	Y	461
9872	LOC_Os02g13250	VVHEGFESYDIDYPTADGVSVLGDA	Y	346
9873	LOC_Os02g13330	WPIVRGFANPQRYKHFIKSCELAAG	Y	75
9874	LOC_Os02g13990	SDNEIVKLENFPYLNRLGTLLVNNN	Y	62
9875	LOC_Os02g14059	FGIQEHIDLGIKYDPSTGIYGMDFY	Y	118
9876	LOC_Os02g17430	RLDFRATNNMAEYEGLLAGLRVAAG	Y	1063
9877	LOC_Os02g18330	RKLNTTILLLRLYIHKDLKHEYMLE	Y	82
9878	LOC_Os02g19420	IVASLLQEIIVVYPALSPPTLSLGA	Y	67
9879	LOC_Os02g22210	NTSTHTTEGILDYMYSDLWRPARKT	Y	386
9880	LOC_Os02g23827	KLLHTGIIYPISYSEWVSPVQVVPK	Y	526
9881	LOC_Os02g25580	RDLQKMVQALPQYSDQIEKLSLHVE	Y	369
9882	LOC_Os02g28020	KKKLRPDGTIEKYKARLVAKGYTQK	Y	697
9883	LOC_Os02g28020	SISEAYASPDADYWKEAVRSEMDSI	Y	648
9884	LOC_Os02g30680	GTHMAILDEVNEYGENVLRRAFWSF	Y	411
9886	LOC_Os02g32340	GILDDPNEEMFFYANKAVTIDQPAF	Y	261
9887	LOC_Os02g32490	ALVPSPKKYQKMYERSINDPAGFWS	Y	75
9888	LOC_Os02g33540	DFAMTSSSLSSEYKHSDEQMRADAL	Y	172
9889	LOC_Os02g34210	DRHAQSYDVQLQYDTDFGEDRTEAD	Y	44
9890	LOC_Os02g34460	EICKYLFQEGVLYAKKDYNLAKHPQ	Y	22
9891	LOC_Os02g34680	LKAHMKTHSVDNYHVCKYPECARRF	Y	152
9892	LOC_Os02g34680	SAAPPTPSADRPYVCPYDGCAKAYI	Y	221
9893	LOC_Os02g35150	TNSDVDSDLETQYQRSSSEGSKLGM	Y	584
9895	LOC_Os02g35800	NTIPSIPAHAVIYAFRGGVRHNRML	Y	358
9896	LOC_Os02g36974	RGNEDRCTLIKEYRGKIETELSKIC	Y	92
9897	LOC_Os02g38250	ESEDEMDGRIAPYHLMEKRSLEKSI	Y	118
9898	LOC_Os02g38840	FGTEGRGGYFDQYGIIRDIIQNHLL	Y	263
9899	LOC_Os02g40880	RFVEIGRVALVNYGKDYGRLVVIVD	Y	17
9900	LOC_Os02g40930	QWPKDLIEIRLQYDTDFGEDRTEAD	Y	347
9901	LOC_Os02g41990	GLARKEDYPHITYYCPHCHALNTSK	Y	320
9902	LOC_Os02g41990	PMFVLPAVSSVIYSTVVKFTRMLER	Y	123
9903	LOC_Os02g42040	NGKDHEGADSESYSDSGSIDGHEDE	Y	864
9904	LOC_Os02g43370	DIGNPDGYWKAPYALIFRNMAILGV	Y	543
9905	LOC_Os02g43370	FFNKDYIPNWLAYAGYALLSIVAVI	Y	394
9906	LOC_Os02g43870	GVGAQGGVGAGAYGRADALVKFPGG	Y	108

9907	LOC_Os02g44690	KHWIGLAVTSAAYFLPYKQLANMSE	Y	58
9908	LOC_Os02g44740	PSSPPRSPRRSAYYVLSPAASHPDV	Y	28
9909	LOC_Os02g44810	ARTIEGDLEEALYRAGAVPEADRAA	Y	83
9910	LOC_Os02g44820	LPAAAKERLRAVYFVHPGFQARLFF	Y	145
9912	LOC_Os02g45070	IDNHGTVKTVVQYFQETYGFNIKHT	Y	496
9913	LOC_Os02g45070	QLGEGLESWRGFYQSIRPTQMGLSL	Y	394
9914	LOC_Os02g46090	LEREEHLVAAFSYFDKDGSGYITVD	Y	476
9915	LOC_Os02g46956	ADSDLAVVLNAWYAAGFYTGRYLMQ	Y	325
9916	LOC_Os02g46962	KKRIMLKDARVQYLSLVLLETIVKN	Y	102
9917	LOC_Os02g47810	PRCESTHTKFCYYNNYSLSQPRYFC	Y	78
9918	LOC_Os02g47900	DLHGVPMRGRFAYDENSKVILQEGY	Y	336
9919	LOC_Os02g48180	YKKDGYNDFNTFYMQVRKFISYQIL	Y	196
9920	LOC_Os02g49070	WNEVLKISIPRNYGPLKLEVYDHDT	Y	235
9921	LOC_Os02g49590	QRIKFKLKLVYMYAAGFYTYSIFAL	Y	127
9922	LOC_Os02g50910	MRKSIRASHSLHYSKNSFLWSDDMD	Y	258
9923	LOC_Os02g51200	FQLRRGEVAVSLYHPQAFLIKFQHR	Y	72
9924	LOC_Os02g52280	KAGVDAKELFHLYIVLWIEDKRRTL	Y	709
9925	LOC_Os02g52390	KGSLHTFLNAFTYPDRTCYPVASTN	Y	196
9926	LOC_Os02g52390	LYQSGYQLNGSAYVISKHISNTWLW	Y	915
9927	LOC_Os02g52820	VDSTTLQLHNLLYEKNHYVKAIRAC	Y	116
9928	LOC_Os02g53270	DNEELLLYLLKAYGERDSTQNLLDK	Y	363
9929	LOC_Os02g53780	LQPSSSLEERRYYDGRFSDQQMALL	Y	206
9930	LOC_Os02g54700	EDAEKAISKLNGYGYDNLILRVEWA	Y	272
9931	LOC_Os02g54770	QYRKSPTYGRRSYSPRDRSPRRRSV	Y	139
9933	LOC_Os02g55020	SMMEDGGLESDEYTFAILVNGLCKM	Y	339
9934	LOC_Os02g55330	ELRAHLRVNIMSYDYSGYGASTGKP	Y	68
9935	LOC_Os02g55330	FWRHPSARLTLLYSHGNAADLGQML	Y	39
9936	LOC_Os02g55420	FNDAKVPWSEYRYYDPKTVGLDFEG	Y	205
9937	LOC_Os02g55420	VQRGLEVFVAQSYSKNLGLYAERIG	Y	301
9938	LOC_Os02g55560	RSGDWSDIGGRDYMEDAHVCISDLA	Y	66
9939	LOC_Os02g56210	IEVSMWQQRVRLYIITGVKGDTVFA	Y	186
9940	LOC_Os02g56740	PTLQLHNNMEDCYIDICSPEVLSLF	Y	236
9941	LOC_Os02g57260	SASACAAGDSAAYQRTSAYGDDVVV	Y	39
9942	LOC_Os02g57660	NWLDGMMHGYGIYTWNECGYYVGTW	Y	219
9943	LOC_Os02g57700	AVIEAENLRHEAYEETRRRQKVERD	Y	351
9944	LOC_Os03g01040	ALVKFMAAVHYAYLQMKMPGPGGPI	Y	691
9945	LOC_Os03g01490	PHICISIDGVGTYCLNTASHTWIKV	Y	254
9946	LOC_Os03g01710	QDEVSKEFDECQYKIVVSVGSIKSG	Y	88
9947	LOC_Os03g02680	EQSWPGVSSLPDYKSAFPKWQAQDL	Y	275
9948	LOC_Os03g03034	LITDGSPAVYRNYTYDEYYKKFWSR	Y	311
9949	LOC_Os03g03060	FTIMHDVEKEYGYEISYDKAWRAKQ	Y	366
9950	LOC_Os03g03830	KQVLDSREKLEYYRTKMQDLVLYKS	Y	633
9951	LOC_Os03g03870	TDRFGDYSGPSKYGKKTTPTISDDE	Y	160

9952	LOC_Os03g04050	VSESGEKAPNFVYRGRLQRTRRAIA	Y	86
9953	LOC_Os03g04460	VPDQKSGKLDLQYLHDHFALNSTIG	Y	110
9954	LOC_Os03g04520	GPLAAYRFLFNEYLGGACAFLEYID	Y	622
9956	LOC_Os03g04590	RQRKPWRRKDGVYMYFEDNAGVIVN	Y	95
9957	LOC_Os03g04920	VLKDGHITQAGKYDDLLQAGTDFNA	Y	830
9958	LOC_Os03g04960	DKALEIASDRVYYIYQTLYDCEEVL	Y	326
9959	LOC_Os03g06120	LLVHSCQDRSYLYLLAMGWNIVYQF	Y	98
9960	LOC_Os03g06200	SSPAAAAADRGVYNFAAGPATLPLS	Y	66
9961	LOC_Os03g06540	EGDPLGPNDATHYRSVVGALQYLTL	Y	1029
9962	LOC_Os03g06540	KARLVAKGFKQRYGIDYEDTFSPVV	Y	812
9963	LOC_Os03g06950	SISLWVKNMNMGYTPEDKVRVFPIR	Y	200
9964	LOC_Os03g07580	DFDYIQEDRLKIYELLSGNVQGALV	Y	610
9965	LOC_Os03g07840	RRCGWLDIVALKYCCEINGFSSLNL	Y	380
9966	LOC_Os03g08220	PGPVSGEISAAYYRNHWRFDLEGLP	Y	645
9967	LOC_Os03g08360	DELWRKVWGEATYDLATVLAFLAVL	Y	95
9969	LOC_Os03g10340	SLADLQNDEDQAYRKIRLRAEDVQG	Y	81
9970	LOC_Os03g10460	SFLEENYSKAKFYLRLKRRNGRVKH	Y	248
9971	LOC_Os03g10510	KGGLYTLDVSSVYKYKSTLVDVKVD	Y	61
9972	LOC_Os03g11200	IEVYERAVLAVTYSVDIWYNYCQFA	Y	205
9973	LOC_Os03g11220	KEGSAGSGGGGKYQDEGGEGKYKKK	Y	114
9974	LOC_Os03g12010	HWHWHKKRCTGSYHRVQGSSSGCVE	Y	195
9975	LOC_Os03g13300	RMPENSIPKEAAYQIINDELMLDGN	Y	43
9976	LOC_Os03g13614	STGPVVLNPMMPYWPVPPPMAGPAT	Y	241
9977	LOC_Os03g13720	SASQLIRNYIIGYHVLPNTMLESSF	Y	470
9978	LOC_Os03g13890	PIQLAIDEVWAQYVQRGGLRKTGHD	Y	520
9979	LOC_Os03g14260	DMLDAKYWEINMYKSALEGRKDEIG	Y	333
9980	LOC_Os03g14450	RAAVPSGASTGVYEALELRDGGSDY	Y	47
9981	LOC_Os03g15650	LLAQKELQKINMYKAPRDKLACILN	Y	158
9983	LOC_Os03g15810	DELVQLVDTMVSYSITYRNTKLEPQ	Y	829
9984	LOC_Os03g15900	LISPMLKVLDEMYRAQDDLARDIRQ	Y	714
9985	LOC_Os03g16310	SIDDGLARFLGGYKLDKFAFKPARG	Y	57
9986	LOC_Os03g16369	QHTPSRVGMHNSYSKDGMNSEDAIK	Y	643
9987	LOC_Os03g16700	LVSSDSTGEMPLYGNKIRRSTSFPR	Y	235
9988	LOC_Os03g16800	NGNGYGYGGYDDYRERNGNNNTDDK	Y	209
9989	LOC_Os03g16800	WCKETGVARSSEYPEVQRVTDKLLE	Y	336
9990	LOC_Os03g17170	AFDQQNHGAVPQYHDRSGSEDKNGF	Y	122
9991	LOC_Os03g18454	AAAAGEKVPLALYYETLCPYCSRFI	Y	44
9992	LOC_Os03g18600	FRVVGGEHRLKNYLSVTTVHPSPSA	Y	162
9993	LOC_Os03g19040	DPEMKRRRRVASYKAYSVEGKVKSS	Y	90
9994	LOC_Os03g19530	KLGGFSIPRPTSYSFERSQPPQRLY	Y	18
9995	LOC_Os03g19870	ISVIERRADQLDYVLVDTPGQIEIF	Y	152
9996	LOC_Os03g19990	DVEGFVRVTHSRYENSTPAWTVFST	Y	674
9997	LOC_Os03g20080	YKKGQVSIFLLIYVDDIIVASSVPD	Y	1024

9998	LOC_Os03g20680	QTADATRDKLGEYKDYTADKARETN	Y	115
9999	LOC_Os03g21470	EALEAARIACNKYMAKHAGKDAFHL	Y	75
10000	LOC_Os03g21540	VVKGSFGYLDPEYFRRQQLTDKSDV	Y	713
10001	LOC_Os03g22120	WGLEKGWGDTAGYVLEMIHLLLDVL	Y	246
10002	LOC_Os03g22190	DSTAKQMNGPKEYYSATLQKLNERR	Y	979
10003	LOC_Os03g22320	EGLLTISDAKENYKIPSQADLIRQA	Y	687
10004	LOC_Os03g22340	TSDGPFSKRYLKYLTKKYLKKHNVR	Y	88
10006	LOC_Os03g23970	VMNAVGVCGLQLYRYGETISIPFFT	Y	130
10007	LOC_Os03g23990	DGSTDLEEFLQVYSTELATRRVMTT	Y	422
10008	LOC_Os03g23990	SVPQYPLYPVYGYPPSTVVRQVGGL	Y	22
10009	LOC_Os03g24050	PSAAIAGFQRRLYGGHRRPDVPLAG	Y	45
10010	LOC_Os03g24110	EEKTTFITPIGTYCYTTMPFGLKNA	Y	917
10011	LOC_Os03g24790	NGSQFISADFQDYCIGLGVKICFAS	Y	357
10012	LOC_Os03g25070	DFEEFSAAAISVYQMEGLETWEQHA	Y	521
10013	LOC_Os03g27030	DRGYRDDYRDRHYRDDYQDREYRRS	Y	144
10014	LOC_Os03g27030	RSRSIERYERERYQEKGYRRRSRSI	Y	167
10015	LOC_Os03g27140	ARVGFTSELFGDYCEDMGIKLCFTS	Y	1636
10016	LOC_Os03g28330	QMNRVRNGELYRYICDTKGAFVQPA	Y	663
10017	LOC_Os03g28960	QVQVCRKCGLLGYYNHKLKASYCSM	Y	1106
10018	LOC_Os03g29035	YGVHLRGLRGAAYILGIRIYRERSK	Y	500
10019	LOC_Os03g29075	SVPQYPLYPVYGYPPSTVARQVGDL	Y	22
10020	LOC_Os03g29085	WPTALKDACDMDYCIGLGVKICFAS	Y	802
10021	LOC_Os03g29260	VFTSLPSAPAASYVNVTRWYDHISA	Y	51
10022	LOC_Os03g32630	NDADDNERLAEVVYEKLNLRDSDAAR	Y	267
10023	LOC_Os03g35560	LDVSYALSATSRYQSDLGESHWIAV	Y	1071
10024	LOC_Os03g39380	RHLPSLRRTFSLYDQINLIDSVPED	Y	43
10025	LOC_Os03g39650	DKLTEDDVAKLSYLQLVIKETLRLH	Y	358
10026	LOC_Os03g40110	PARKEKGWVDEIYEREGKKIGEDAE	Y	377
10027	LOC_Os03g40180	VMRFVQRVRCWEYRQQPAIVRLTRP	Y	30
10028	LOC_Os03g43670	EDLKIRRAQEFSYEELEQATGGFSE	Y	494
10029	LOC_Os03g43800	EAETGDYPKYASYLMNNEGSASGKS	Y	396
10030	LOC_Os03g44020	AGVDWWAYGVFLYELLYGRTPFVGA	Y	348
10031	LOC_Os03g44310	PMHPSVYKVGLQYLSGEVSGGNGRC	Y	298
10032	LOC_Os03g44800	RHLARLRSTLREYRHTCLRLGIPLA	Y	163
10033	LOC_Os03g44900	GRPEISADQREKYLQRLQQVQQQGS	Y	534
10034	LOC_Os03g45140	FCNVLKMRGPDQYWRIDPRWVPRLR	Y	858
10035	LOC_Os03g45140	HDVEKEYGYEISYDKAWRAKQKALE	Y	370
10036	LOC_Os03g45480	PIQLAIDKAWAQYVQRGGLRKTGHD	Y	311
10037	LOC_Os03g46570	VRQPAAAEVMRQYSQVCAAAADAAE	Y	81
10038	LOC_Os03g46590	RYGIMTTNLAEVYNWVMRGVRGLPL	Y	644
10039	LOC_Os03g46600	PLAPHGVESLDLYQCPVTKAKGYRD	Y	137
10040	LOC_Os03g46710	VKENRNFGSTPPYKVEPPTLDLADE	Y	646
10041	LOC_Os03g47800	YGGGRDRYANDRYPSGGDRYVPDRY	Y	195

10042	LOC_Os03g47890	PGPEMMMVDLRDYGSGVGTLAVRMD	Y	49
10043	LOC_Os03g47930	LDDVDIDQVSVDYVLNCAKKGEALD	Y	54
10044	LOC_Os03g48140	LSIQQLYRISTMYWDDKYGTHTVSS	Y	1493
10045	LOC_Os03g48674	VWTQVGEIVFPVYTTVPISAGPSMT	Y	37
10046	LOC_Os03g48930	KNPYYVNVLLAGYDSDVGASLYYID	Y	106
10047	LOC_Os03g49400	SGVTKHDFSNKEYEVKLLQSLRFCI	Y	1076
10048	LOC_Os03g49800	ILYFGIIDILQDYDITKRLEHAYKS	Y	687
10049	LOC_Os03g50350	NNNLMGQFHNALYLGDAMKRVEILE	Y	740
10050	LOC_Os03g50390	VFAGDLVGVMEKYADSHPEWKETLE	Y	335
10051	LOC_Os03g51360	EDRPRRKVQRPVYFVSEALRDAKTR	Y	616
10052	LOC_Os03g51360	NRAVPRDVIKSAYNKKKARRPRKKK	Y	463
10053	LOC_Os03g51600	VDVNEFQTNLVPYPRIHFMLSSYAP	Y	262
10054	LOC_Os03g52490	GNNQGGYNQGGGYYDNQGGYGGYDN	Y	201
10055	LOC_Os03g52970	NRIGNLLVPNDNYCKFENWIMPLFD	Y	184
10057	LOC_Os03g56241	GHSNVWNSHPKNYGPGSRVCRVCGN	Y	14
10058	LOC_Os03g56430	TDVHSMPKKSQKYREMKSKEGIQNL	Y	583
10059	LOC_Os03g56790	VVVEKTGATTESYDDFLASLPENDC	Y	59
10060	LOC_Os03g56800	APRLFSQGVSYTYDDVIFLPGYIGF	Y	27
10061	LOC_Os03g56900	VIGGWCNFLTVIYIGFVSAHTLPVL	Y	190
10062	LOC_Os03g58590	LDLDRESQINVKYWEALMVVCDWEL	Y	288
10063	LOC_Os03g58590	NLDSQVYWWHDKYRPRKPKYFNRVH	Y	552
10064	LOC_Os03g58740	RPQEVHHRPTKSYTGNPPQPLEKDA	Y	223
10065	LOC_Os03g60460	NEDFWLNEGFTTYAERRIVEVVQGE	Y	333
10066	LOC_Os03g60870	AGQERYRAITSAYYRGAVGALLVYD	Y	81
10067	LOC_Os03g61340	RGYRCVAPDLRGYGGTTAPPEHTSY	Y	69
10068	LOC_Os03g62600	AYYRGAVGALLVYDVTKATTFENVK	Y	98
10069	LOC_Os03g62700	RVLGTFGYHAPEYAMTGQLTQKSDV	Y	248
10070	LOC_Os03g63140	TGNAPVMTSEADYIKRYQQRKYEST	Y	220
10071	LOC_Os03g63320	EASGAQAAAGPPYSKRRRRPSVRLG	Y	47
10072	LOC_Os03g63590	ATKAIYRLLLSDYVKVSKVSVEDML	Y	129
10073	LOC_Os03g63590	SFEVIKYRLKQIYESVESSTEESDV	Y	550
10074	LOC_Os03g63650	RGRTTSVYRSGYYRPGMVQDDMAVP	Y	42
10075	LOC_Os03g63670	QPQPSGYSIDPRYQPSFSGVLPGTS	Y	382
10076	LOC_Os03g64340	PPPPPPPPPPPVYYSSYVMLDRPPP	Y	368
10077	LOC_Os03g64340	VTGTVSGDKLAEYIHRRTGKLATVV	Y	205
10078	LOC_Os04g01480	LEGILESPVINGYRNKCEFSVGFSL	Y	315
10079	LOC_Os04g02000	ASANWPYALAGRYGMQAAGWPFGGN	Y	153
10080	LOC_Os04g02150	LPLLSYSAPFAIYHQYLQPLATCMH	Y	436
10081	LOC_Os04g02320	HAADLVQLRPTAYHETSPMQLVRGN	Y	204
10082	LOC_Os04g02320	REEFLLLTKQNGYGKCICEKIPSLK	Y	52
10083	LOC_Os04g02370	CIQGGCPWRVHGYKPQHDTLWVASR	Y	500
10084	LOC_Os04g02500	KLAHEFESHLSSYDHNHRKRFKEMK	Y	266
10085	LOC_Os04g02820	TYEDTLLGDVQVYPEKGTVAFSAGL	Y	203

10086	LOC_Os04g02870	YAFRDDTERGDRYDGARGGYGRRDD	Y	174
10087	LOC_Os04g04220	RYNVMTTNLAELYNWVMPDTRALPF	Y	46
10088	LOC_Os04g06770	VGRKVIDKLQQTYRSELSSKDFAYD	Y	108
10089	LOC_Os04g07250	LLHLGGPDNITAYSLEDNKLWKRHL	Y	97
10091	LOC_Os04g08440	ISKRYVLVEGTLYRRAANGILLKCI	Y	1566
10092	LOC_Os04g08480	DRHAQSYDVQLQYDTDFGDDRTEAD	Y	611
10093	LOC_Os04g09860	IDMLMLNKLKESYSQIRSGSFDAVS	Y	362
10094	LOC_Os04g17440	EEAERALTQLKAYLSSPPVLVAPEP	Y	1111
10095	LOC_Os04g17440	YAVLMASRKLRHYFQAHRVTVVTSY	Y	1355
10096	LOC_Os04g18010	SCRGIWTVYYKSYRGQMAEDNEYHA	Y	54
10097	LOC_Os04g19990	SHTDPKQPFEHWYTSGQSKTIRIAV	Y	74
10098	LOC_Os04g22060	QLLAYDNALTPRYFLTKFVDGLKDD	Y	258
10099	LOC_Os04g22180	YIVVDFKDLFDLYRLRAVDTSLLKC	Y	183
10100	LOC_Os04g22400	PPDGFRQIQGVDYDETFSPVTMLKS	Y	484
10101	LOC_Os04g22400	QRFERIRRTPARYALLTTGQHDILL	Y	417
10102	LOC_Os04g23060	FEARSTDGLFLGYPAHTRGYRVLIL	Y	357
10103	LOC_Os04g23060	VLVEMARTMLDEYKTPRKFWAEAIN	Y	282
10104	LOC_Os04g26900	ELKKLVEEGKVKYIGLCEASASTIR	Y	17
10106	LOC_Os04g28180	AVTRKTRILDVVYNASNNELVRTQT	Y	84
10107	LOC_Os04g28770	QKIHACENYCILYRKEFADLNSCPT	Y	191
10108	LOC_Os04g28770	WYLRVKDRLKRLYSNRDDAKLMRWH	Y	252
10109	LOC_Os04g30010	SACYNTSSRDMDYNDWQINFTGTPL	Y	124
10110	LOC_Os04g31180	VTLFACGSEHKKYRLAKWSILCKPK	Y	460
10111	LOC_Os04g31260	FWEDKWLGYHLSYPVATYLGYPPPT	Y	69
10112	LOC_Os04g31330	SPLGRHLVKLISYLEANLEEKSKLY	Y	501
10113	LOC_Os04g31500	ALVKFMAAVHYAYLQMKMPGPGWPH	Y	721
10114	LOC_Os04g32650	IIDMTGNTIGKRYARTDEIGVPLAI	Y	598
10115	LOC_Os04g34400	LRLPLKIKIFLWYLKEGVILTKDNL	Y	496
10116	LOC_Os04g35240	KTADVWSCGVTLYVMLVGAYPFEDP	Y	191
10117	LOC_Os04g35260	SDFLSGKDRPRWYDGRREVHIHPSS	Y	1311
10118	LOC_Os04g35950	WPPRFWPTIAEKYDGSVNPAEFLQV	Y	245
10119	LOC_Os04g37640	GEGGRDPPPPWMYVHLRHDYTRPGA	Y	278
10120	LOC_Os04g37640	SNTTSDAPSECRYLVWTPHAGLGNR	Y	184
10121	LOC_Os04g37920	VLQAAGVELGSNYPLPIVGLDAANA	Y	478
10122	LOC_Os04g38090	TKVAHFIPVKTTYSGSRLAELYMAR	Y	913
10123	LOC_Os04g38570	VLFATSIFENIAYGKDGATEEEVIE	Y	1115
10124	LOC_Os04g39980	SGYVPPGTANPLYEAFGLCDAAAPA	Y	86
10126	LOC_Os04g41100	GLSRQYGSPLKPYTQLVVTLWYRAP	Y	521
10127	LOC_Os04g43140	IALISPADKAKFYSLCKSLSKENLQ	Y	623
10128	LOC_Os04g43140	KKRKKRKRGDDDYALPGDGDLVVEC	Y	120
10129	LOC_Os04g43300	CMEAKEPVPEEPYEISSDVHGSFDG	Y	503
10130	LOC_Os04g43430	SGSPGDVLGAFRYIRCTAGGRPDQF	Y	147
10131	LOC_Os04g44530	TSRSPTPFGTAKYMLDKIPKDASSK	Y	353

10132	LOC_Os04g44740	QNLSNLLRLALLYKYGGVYLDADVV	Y	284
10133	LOC_Os04g45190	SCSMQLTNRTDDYIAFKVKTTSPKK	Y	39
10134	LOC_Os04g45580	PQNCQRRGSALNYDAESETLSRAGS	Y	677
10135	LOC_Os04g45580	VELRRLSFLKDTYSNGAIASIPNTS	Y	822
10136	LOC_Os04g46320	ADFQPRISDFGLYLLLNPAAAQQML	Y	238
10137	LOC_Os04g47380	CHLVISCQRSPIYIGGRYLKLSRNV	Y	142
10138	LOC_Os04g47870	VNRAVMFELVTLYRYSHLGGRLPAY	Y	301
10139	LOC_Os04g47912	QYPCGFPTILSWYIAAGILFKSGHY	Y	320
10140	LOC_Os04g49210	VSNWRDFLRLHCYPLESFIDQWPSN	Y	142
10141	LOC_Os04g50890	EGDSKLFFLATIYALLGIPLSYLIW	Y	166
10142	LOC_Os04g51060	LGRKACLEGASLYRQNYSSQLGYMG	Y	563
10143	LOC_Os04g51540	PFRRAPGGFEYLYVAIDKFTKWPEA	Y	1714
10144	LOC_Os04g51700	GADLDVIIIGGYYGSGRRGGEVAQF	Y	451
10145	LOC_Os04g52200	EAEEALKYFNNTYIDTCKITCEVAR	Y	87
10146	LOC_Os04g52760	CKGYLSAIIIYLYAHDFQQAQKCYN	Y	129
10147	LOC_Os04g52850	NQLSLYSRETTQYNYQELRQMTANY	Y	204
10148	LOC_Os04g52850	SGGKDGPSIRLSYHRHAYGLGEHYN	Y	306
10149	LOC_Os04g52920	VCTVNNAGVTSEYQTKATDNSSSIE	Y	265
10150	LOC_Os04g52940	RLIGADETMYSGYVDSMQWLDDIKV	Y	181
10152	LOC_Os04g55920	SPPPNPSQLTIFYGGSVCVYDSVPP	Y	78
10153	LOC_Os04g55960	GDGEPTDNAARFYKWFTEGKEKEVW	Y	161
10154	LOC_Os04g56290	DAIVFESELDRIYLGTPSKIAIIDH	Y	237
10155	LOC_Os04g56630	VRRKESESSSKRYNDDDKSSMYGNI	Y	765
10156	LOC_Os04g56640	MATSSAYPPPPPYYRLYKDYEKDPS	Y	13
10157	LOC_Os04g59494	IMLGCPTGNLIPYPSIRLSFFLSFH	Y	903
10158	LOC_Os04g59624	NPSTDNQSVDRAYRIGQMKDVIVYR	Y	355
10159	LOC_Os05g01040	GYCISNNQRLLVYDFVPNNTLHYHL	Y	141
10160	LOC_Os05g01360	DLLEVAIRNIIRYLCASREKDLSSS	Y	472
10161	LOC_Os05g01480	MAGTRRAEEEYDYLFKVVLIGDSGV	Y	13
10162	LOC_Os05g02240	AEFRQRMGAMVRYEERVVVGNVIEE	Y	737
10163	LOC_Os05g02310	HRLAEIRRFFEDYKKNENKEVAVND	Y	176
10164	LOC_Os05g03630	ADDSLFKQVSEAYDVLSDPQKRAIY	Y	53
10165	LOC_Os05g03760	MYSFKVKPCSRAYSHDWTECPFVHP	Y	227
10166	LOC_Os05g05480	LVPFHVEGGQPSYLIVAGLVFTPLT	Y	429
10167	LOC_Os05g06980	QAVADILQDRTDYKSFKKTVARLEE	Y	46
10168	LOC_Os05g07000	EERLHSRRDGRGYSRSPRRHDSPSN	Y	201
10169	LOC_Os05g07120	IPPEANEVTVQCYAGGEHTAAARTY	Y	142
10170	LOC_Os05g08300	FGGSTSYSLKREYKKVEREVCSTSQ	Y	197
10171	LOC_Os05g08370	LLERLAYINTIVYPITSIPLIAYCV	Y	854
10172	LOC_Os05g10540	YFQAHRVTVVSSYPLGQILHNREGT	Y	1307
10173	LOC_Os05g10810	VETVLKLSLPNVYLWLCMFYAFFHL	Y	381
10175	LOC_Os05g12680	RVHHKHLVSLVGYCISGGKRLLVYE	Y	363
10176	LOC_Os05g13510	KILSMQTDWGGEYLKLHTFFNEIGI	Y	543

10177	LOC_Os05g14110	KRKAQAEVLASEYERPPKHPDPQGS	Y	531
10178	LOC_Os05g14770	NGLGAVSPPVRVYGKMMWRSDRLKS	Y	224
10179	LOC_Os05g14940	RVVDVETMEWEVYAVYQKEKENLMQ	Y	215
10180	LOC_Os05g16910	ITDTFAPQPRSAYNSMAEFVEHVHV	Y	1342
10181	LOC_Os05g19560	QGANQRVRYTNPYPGGSSSQQQQQQ	Y	562
10182	LOC_Os05g19850	LAPGTTPLYKRPYRMAANELAEVKK	Y	874
10183	LOC_Os05g22990	LIHCCCVMLTSYYPVQVNVPVRALI	Y	333
10184	LOC_Os05g23080	FRIDLAPGTTPLYKRPYRMAANELA	Y	852
10185	LOC_Os05g23840	TLGEELRARHRQYVWELGRSPLEAE	Y	97
10187	LOC_Os05g23940	CGLCGKVLDDQVYDGEPTFQKGADG	Y	35
10188	LOC_Os05g23940	GRRTTHVAAACLYIACRQSKKAYLL	Y	124
10189	LOC_Os05g24460	ADQDQLRRDDLDYLEERRRRAALRA	Y	1874
10190	LOC_Os05g26890	LRSYTSVIHANVYQTIKILYEGAKE	Y	47
10191	LOC_Os05g27820	MHGWDREGHPVCYNAYGVFKDRDMY	Y	174
10192	LOC_Os05g30420	YIGRLIEKHGDDYKAMFMDIKLNTM	Y	157
10193	LOC_Os05g32600	WPDMVYLPDYVEYQFVSAPPLRSLF	Y	253
10194	LOC_Os05g32610	DKPMLERLRSNPYEGVKTRFVMEVV	Y	1220
10195	LOC_Os05g33100	LRKIADKVASSGYFVVVPDFLHGDP	Y	69
10196	LOC_Os05g33380	DAKKVSPEVIAEYTVRTLQRTVPAA	Y	246
10197	LOC_Os05g33940	GGFCISHPSWLMYHHFYARLACALP	Y	118
10198	LOC_Os05g34900	NGHKSGANGTFEYPYSAYRDKDAPG	Y	260
10199	LOC_Os05g37434	KTTQEHCPNLQYYYLGYYIHSCNKM	Y	469
10200	LOC_Os05g37434	RRSHFDPEEFALYQRYQTKVHKEKK	Y	358
10202	LOC_Os05g39090	PEIDIWSAGVILYILLCGVPPFWAE	Y	264
10203	LOC_Os05g40300	IGGMLCIMGNAQYYIHRAAHGRPKH	Y	28
10204	LOC_Os05g41790	SQHEIYRTLVHAYGEDNRGLTHPVV	Y	197
10205	LOC_Os05g42230	ESAFAVKWSVQNYLRPGDAVVLLHV	Y	81
10206	LOC_Os05g43060	FLYKRVALSSLIYWGIQPTETELLK	Y	367
10207	LOC_Os05g43570	DYIAPEVLLKKGYGMECDWWSLGAI	Y	337
10208	LOC_Os05g44100	EKVVEVINPEDDYVWVHDYHLMALP	Y	223
10209	LOC_Os05g44100	IDQSVPSAVRLAYYTVAECVVVTAV	Y	449
10210	LOC_Os05g44922	VIRELVCGLAHMYNVSKIYGIQNGY	Y	193
10211	LOC_Os05g44922	YIIGGDGTQKGAYEIFKEIRKRGLK	Y	269
10212	LOC_Os05g45420	IDTPADIYVVMEYVKSGELFDYIVE	Y	93
10213	LOC_Os05g45660	NEFLKPAEGERYYGGRGRGRGRGDR	Y	331
10214	LOC_Os05g46330	FTSSARTDNLQLYHWVRVVNGAPPT	Y	90
10215	LOC_Os05g46490	SGAEYVRKEVPLYVFPTRHLLKLDT	Y	276
10216	LOC_Os05g46620	AAFGEAMLTTIGYIYVRQAARELGK	Y	186
10217	LOC_Os05g48310	IEGVNTKEEVGWYAGKRIAYVYKAK	Y	48
10218	LOC_Os05g49230	QDKFYHLAKEQGYRSRAAFKLLQLD	Y	22
10219	LOC_Os05g49570	TAGCDLLCFLDAYSGYHQIRMARED	Y	876
10220	LOC_Os05g50280	TTIRFADIKQKRYSVPLMIGIGLLV	Y	294
10221	LOC_Os05g50810	GRELGRGQFGVTYLATHKPTGRRYA	Y	128

10222	LOC_Os05g51070	FAPLSARPNAGGYRAPELVDARRPT	Y	575
10223	LOC_Os05g51180	FGEGDANGFEGGYGGGGGFGDGGLA	Y	108
10224	LOC_Os05g51280	TNLQNDRQGMVNYIYDTLWSRRDKE	Y	724
10225	LOC_Os05g51490	VKRSIDLFFQYPYNNFLHHHVESII	Y	374
10226	LOC_Os05g51500	VFIVALNKVDRLYGWKKCTNAPIGK	Y	745
10227	LOC_Os05g51754	ATGSRGKTEKKVYSLPGQKFDPPEE	Y	49
10228	LOC_Os06g01700	QGGTRIFGPSQKYSSRDLAAHTTLK	Y	30
10229	LOC_Os06g02130	LQPNASNGNTQVYMNGREITKIELR	Y	362
10230	LOC_Os06g02180	IYGSVTEDVVTGYRMHNRGWKSVYC	Y	878
10231	LOC_Os06g02370	REAYRREPAAPAYVMPEEPPAMVEL	Y	85
10232	LOC_Os06g02510	PLRPIVQCQTLKYNMKSRAGRGFTL	Y	65
10233	LOC_Os06g04330	DRQILVSRNSRGYFALGYSLPGEPF	Y	41
10234	LOC_Os06g04330	VSRNSRGYFALGYSLPGEPFPSLHE	Y	46
10235	LOC_Os06g04510	TGAPCRSERLAKYNQLLRIEEELGD	Y	418
10236	LOC_Os06g04510	VVIGMDVAASEFYSEKDKTYDLNFK	Y	258
10237	LOC_Os06g05040	LRLIAWIAEIQAYLTDKTLPEDREG	Y	1534
10240	LOC_Os06g05740	KVATAARKEDIPYIRCQVCERIARE	Y	47
10241	LOC_Os06g06090	PQRNSFNDVYIAYELMDTDLHQIIR	Y	148
10242	LOC_Os06g07350	RMVLQRACAFVTYTTREGAEKAAEE	Y	270
10243	LOC_Os06g07350	YYHGQYPPYYPPYGGYMPPPRMPYP	Y	425
10244	LOC_Os06g09880	PDGKSLLSILDAYEKMGKHEKGLSL	Y	613
10245	LOC_Os06g10170	GGKPCLGGVSTWYNDEMCRDMGYEP	Y	489
10246	LOC_Os06g10170	PLRWLLSKLAETYFKMQIPMEKHGM	Y	302
10248	LOC_Os06g12260	DIASLGARVEELYRPGELAARGSRV	Y	562
10249	LOC_Os06g12580	RKPQQEESYGSEYGSGYGRKPQAES	Y	223
10250	LOC_Os06g12610	IIWYTLMLFMFEYRGARILITEQFP	Y	154
10251	LOC_Os06g12780	PNFSLPWQRKRQYSSSSSGFIIGGH	Y	190
10252	LOC_Os06g13390	NETNHEGEIVPAYYICGVPGSYYTR	Y	101
10253	LOC_Os06g13390	PQSVVKLFQEQFYKDFSLFLTLRHE	Y	171
10254	LOC_Os06g13680	AGVLENHEEGRRYAEHGLRNYVRDK	Y	43
10255	LOC_Os06g15420	VLRNAFDDEEKPYLPKHILYRQKEQ	Y	440
10256	LOC_Os06g16020	TDDKGLSPGPYKYLRSFSSVEDAGS	Y	162
10257	LOC_Os06g16280	VLESSVSLFVGSYILQLIIHLPSHL	Y	794
10258	LOC_Os06g17290	AVSALNRALSSEYPSKSRSEGRASG	Y	76
10259	LOC_Os06g19390	VSQLMVFLWVLYYLITVEGGGATEQ	Y	467
10260	LOC_Os06g20390	PQSQFSSPAQSSYSSPSYQGTDYPS	Y	257
10261	LOC_Os06g20390	VTPPAIAPPASQYKYDSSYQPEVEK	Y	434
10262	LOC_Os06g20490	GSLKVHNGTQEGYCGSYLKAWLEVV	Y	205
10263	LOC_Os06g20840	RGANGIAIKQAAYARKILTQFGMLH	Y	293
10264	LOC_Os06g21350	LAYLISCRELRCYYKANVDGFSATD	Y	19
10265	LOC_Os06g22700	DGSPPYGRGGRSYGRGSGAPGKEFI	Y	54
10266	LOC_Os06g23530	LREILLDTDLSSYSVVMLDEAHERT	Y	545
10267	LOC_Os06g24810	EGRRDRERRLLVYGDGSTPQGALQA	Y	72

10268	LOC_Os06g26340	RWYRAPELCGSFYSKYTPAIDIWSI	Y	206
10269	LOC_Os06g27970	AGTNPDPVSVVSYLNNLKSSEESLK	Y	125
10270	LOC_Os06g28140	NRVPSKSVDKTPYEIWTGKRPSLSF	Y	407
10271	LOC_Os06g28140	PDVSYAHSATSQYQSAPVYGGQEEL	Y	750
10272	LOC_Os06g29430	EGAVDVRRGASRYTDNNQRSDWRTE	Y	349
10273	LOC_Os06g30570	PWKIGFKEDIHTYRSRKRSKRDTEA	Y	380
10274	LOC_Os06g30570	VNEPTNVNVKCNYTQMADRDEEQIL	Y	54
10275	LOC_Os06g32060	PQTKPYPLDMWKYGSALQQRPELNP	Y	1411
10276	LOC_Os06g33520	GLLTDHISENVDYKVIVFCTTAKVT	Y	714
10277	LOC_Os06g34710	MQDLPKRDDGLCYYGAYLQRLIWAQ	Y	237
10278	LOC_Os06g35030	AAGKDGGEEVREYKSDARKLEELFK	Y	57
10279	LOC_Os06g36710	SLSYGQGSSSSGYGMGLPPGRDYAS	Y	253
10280	LOC_Os06g36730	VWTQVGEIVFPVYTTVPISAGPSTT	Y	78
10281	LOC_Os06g37440	VTLADIVMTCNLYYGFVRILIKSFT	Y	168
10282	LOC_Os06g38120	FLSPQATGRQAIYIDISMFLAFSSF	Y	130
10283	LOC_Os06g40640	QGLDSLGARCAKYYEAGARFAKWRA	Y	162
10286	LOC_Os06g42030	LVGRIGPKEYRIYRCIVRYGYHDVH	Y	626
10287	LOC_Os06g42190	WPPRFRPTIAEKYDGSVNPAEFLQV	Y	245
10288	LOC_Os06g43790	NVCCYESMQAGQYRLKHLGIEKLTQ	Y	987
10289	LOC_Os06g44030	LGGHSSCVWSLAYRALLFNLFCHCC	Y	582
10290	LOC_Os06g45870	SGKRVRTRKQFAYPIFRPTENKLQK	Y	234
10291	LOC_Os06g45910	CKLNPVTQDEDLYTIFSRFGTVTSA	Y	259
10292	LOC_Os06g45980	DHQRPSNQTETEYVSQMLTKIKRFA	Y	597
10293	LOC_Os06g46880	ARGMPPPQAQQLYSHLPPHQQPPHF	Y	68
10294	LOC_Os06g46890	SKTKKLIKAHARYVKAQDDLKRSQA	Y	219
10295	LOC_Os06g47220	CRSVPHEHSAAYYGCGGGYDYEDVS	Y	16
10296	LOC_Os06g49740	TVHPDCINASNPYHVCSEYCFKRIA	Y	64
10297	LOC_Os06g50742	GVAKLDPEKNTLYSMRCYLATGISK	Y	455
10298	LOC_Os06g51140	ADMAAADERAGRYPCPLCDRHFPTE	Y	56
10299	LOC_Os06g51220	RWKSLTEADKAPYVAKANKLKAEYN	Y	92
10300	LOC_Os06g51250	PWPKITQADVRKYMIVFIKVDRDRD	Y	384
10301	LOC_Os06g51270	EMQRFYREYYKKYIQALQNAADKAD	Y	139
10302	LOC_Os07g01110	CPILPGSGAGSSYTYRFNVTGQEGT	Y	121
10303	LOC_Os07g01110	LLEASRSRSSGVYTRDFPDRPPVMF	Y	423
10304	LOC_Os07g02610	LNILRKEMYRGYYTLDRFRCHVHEA	Y	102
10305	LOC_Os07g02610	QLNILRKEMYRGYYTLDRFRCHVHE	Y	101
10306	LOC_Os07g04240	GNMTEDDWRWHMYDTVKGSDWLGDQ	Y	108
10307	LOC_Os07g05050	FKESAPLDSLCVYGGVPISHQMRAL	Y	190
10308	LOC_Os07g06130	NNQFYQPATIHGYQLASYLKQMNAN	Y	830
10310	LOC_Os07g07270	HSHFDRSLESGPYTLKYRGSMWGYK	Y	139
10311	LOC_Os07g08960	YERDGGRSGGSYYRDEPRGSGGYDR	Y	251
10312	LOC_Os07g09060	LDDAIQIVNRNKYGNGASIFTTSGV	Y	444
10313	LOC_Os07g09384	VRDGAHARLRTRYGNYLRANGGLPP	Y	109

10314	LOC_Os07g10050	DSQLVVNQVCKEYRCSDPQMDAYVR	Y	1471
10315	LOC_Os07g10350	EARIILSNLSDEYVENPQNDFPVGL	Y	377
10316	LOC_Os07g10600	EATCHAPDPVGCYKEIYRVLKPGQC	Y	237
10317	LOC_Os07g10890	PFPMYHPGAAAAYYAHASMAAGVPY	Y	87
10318	LOC_Os07g12170	GGQDKIRPLWRHYFQNTQGLIFVVD	Y	204
10319	LOC_Os07g12910	HSETLCGTCGGRYNANEFWIGCDIC	Y	197
10320	LOC_Os07g14270	YIPDPEDKKPEGYDDIPKEIPDPDA	Y	246
10321	LOC_Os07g15620	PKANGKLRMCIDYTDLNKACPKDPY	Y	749
10322	LOC_Os07g18300	FSKIDLKLIERLYLPVLKEHHWFLI	Y	819
10323	LOC_Os07g26690	CGVGLVKAFQSAYFNRYGGGANTLA	Y	155
10324	LOC_Os07g27790	FDDSFGFERYVDYALDVPMYFVYRN	Y	305
10325	LOC_Os07g27950	QCLFHVLCVTVEYRRRREARGGGFG	Y	140
10326	LOC_Os07g28430	MQLQELEQQSRVYQYMAARVPVPTH	Y	130
10327	LOC_Os07g29360	GPEIDEYYADVKYERIAILNALGAF	Y	80
10328	LOC_Os07g29450	LKKGEVIYLETHYNRYELCFSGEKA	Y	678
10329	LOC_Os07g29520	KARLVAKGFKQRYGIDYEDTFNPVV	Y	536
10330	LOC_Os07g30040	EHRERVAIQATDYQQALRRYHEKRI	Y	1193
10331	LOC_Os07g30250	GDATVILRYYCFYVICLAMNGTSEA	Y	139
10332	LOC_Os07g30820	SYGAPYPHLGLRYGYGPPVGPPASY	Y	124
10333	LOC_Os07g30820	VGPPASYGLFSSYGQPGPMGSPMGG	Y	142
10334	LOC_Os07g30980	FLSGDEAFGYAQYVIKWEQIPIDKR	Y	1095
10335	LOC_Os07g31460	ANCFTFYCRSFGYEARLILDFTDHV	Y	340
10336	LOC_Os07g32220	LGTAGYAEDQRVYEAIEYGFPDYVW	Y	182
10337	LOC_Os07g32400	LSQCRCPYITDYYGSYLHQTKLWIV	Y	77
10338	LOC_Os07g32530	LGTVRVPGIGLLYNELARGVPGIFG	Y	622
10339	LOC_Os07g36600	ESAYAVRWAVANYLRPGDAVILLHV	Y	79
10340	LOC_Os07g36940	GSGPRRGAAPMDYGPRGSAAALASP	Y	1456
10341	LOC_Os07g37650	FIQSMGNEKSNSYWEAELPPNYDRV	Y	95
10342	LOC_Os07g37810	HESGLLSDRSDVYSFGVVMLELLTG	Y	680
10343	LOC_Os07g38090	DGVGLWYGRSKAYEARTPLMVAATY	Y	67
10344	LOC_Os07g38640	AEVLGKGVSGSTYKAVLEDGIVVAV	Y	339
10345	LOC_Os07g41110	VVNEADKCVYYRYGGGEGVILCLYV	Y	1594
10346	LOC_Os07g41810	EALGPTHVLHSRYATKPDLAAYAAH	Y	113
10347	LOC_Os07g42950	SKDDDVRKYVNTYRRTFTTKNGKKV	Y	162
10348	LOC_Os07g43030	DQNIEKLVKEKLYCWLVHKVHDEDK	Y	610
10349	LOC_Os07g43240	KVDIKTVRKMAEYMNKHFAITNKEE	Y	147
10350	LOC_Os07g43800	FLAAASSGFFYSYSSFLHSFARSLV	Y	45
10351	LOC_Os07g44190	SGHSPLKRPIAEYLRYGVINLDKPS	Y	100
10352	LOC_Os07g44710	QFYDAFEDEDNVYIVMELCKGGELL	Y	219
10353	LOC_Os07g44710	TEADMWSIGVIAYILLCGSRPFWAR	Y	336
10354	LOC_Os07g45780	WTNGNQHTTIRLYAAKVMAEFAKSL	Y	318
10355	LOC_Os07g46750	LKALEQHLSGKTYVSGNAISKDDIK	Y	26
10356	LOC_Os07g47180	KQPMTSRVVTLWYRPPELLLGATDY	Y	292

10357	LOC_Os07g47350	ADDNGEGGTFALYSLLCRHAKFSLL	Y	129
10358	LOC_Os07g47700	GARFLLTSTSEVYGDPLQHPQVETY	Y	243
10359	LOC_Os07g48310	KHANLVPLRAYYYSKDEKLIVYDYL	Y	408
10360	LOC_Os07g48410	GADGLGWKPCLYYARGYCKNGSACR	Y	238
10361	LOC_Os08g01420	RQPPPPEDHLVTYKRRRSKETQPLP	Y	25
10362	LOC_Os08g01930	AQQGAQQGGYAQYPQSQPAYGDQAA	Y	585
10363	LOC_Os08g02470	YFQAHRVTVVTSYPLGQILHNREGT	Y	1232
10364	LOC_Os08g02690	VKLAASDLKELGYDDFHRYFVKKLV	Y	166
10365	LOC_Os08g03840	WTTGDDQNKIILYDSEVEGRNRVLG	Y	354
10366	LOC_Os08g03970	AKTRYPQAQKMLYAILMASRKLRHY	Y	1275
10367	LOC_Os08g04840	VPMPYYGYVPVFYAPPGAVQAQHEV	Y	300
10368	LOC_Os08g07480	ADGGGGRKVRGPYCRFCRSAEEVVR	Y	1217
10369	LOC_Os08g07480	IGVEEEEDIRCAYGIRHLFAGDEEK	Y	748
10371	LOC_Os08g08830	LKVRNESVINALYQDLPRQCKTCGL	Y	823
10372	LOC_Os08g09160	EGRRDGERRLLVYGDGSTPQGALQA	Y	72
10374	LOC_Os08g12140	PPHQSNSGRDIEYAPGLIPNGGGPS	Y	441
10375	LOC_Os08g14230	SKFYTGDCYIFQYMYPGDDKEECLI	Y	428
10376	LOC_Os08g14570	VDLDEYAMEDEEYEERLKKEKISLF	Y	157
10377	LOC_Os08g16130	EACLIGGSVRNAYHTKYLGYYMKHD	Y	123
10378	LOC_Os08g16930	KSMSENPSQTFVYRVKNPALPPTLR	Y	101
10379	LOC_Os08g20020	DQPEANGVGKHQYHMDDKENKGLDK	Y	252
10380	LOC_Os08g20390	EGRRDGERRLLVYGDGSTPQGMLQA	Y	72
10381	LOC_Os08g21760	GGYYIVTYALGIYILNLLIAFLSPQ	Y	89
10382	LOC_Os08g23360	FRAERCFYLAKSYSSAGKRAESYAL	Y	466
10383	LOC_Os08g25570	GYTIRNVARCWCYETAVAVGVEPDN	Y	327
10384	LOC_Os08g27420	LQPIRDAETGRVYLPPACHTLSKDE	Y	313
10385	LOC_Os08g27674	SWQGYDISGYRFYTMFKDRKSAAQN	Y	825
10386	LOC_Os08g28570	ALENLPCLMTVQYLLYQYNKNDHEI	Y	827
10387	LOC_Os08g29124	HSGHLRADAVKQYLLSGESLYQRSS	Y	221
10388	LOC_Os08g29490	RAALVKFMVATHYAYLQMKMPGPAG	Y	667
10389	LOC_Os08g29500	VLRALQDLGFREYIEEVQAAYEHHK	Y	87
10390	LOC_Os08g31350	RWRQWKSDMNTNYVKKNKIPFEEWG	Y	229
10391	LOC_Os08g31980	INTDDDHVWVHDYHLMLLPTFLRKR	Y	205
10392	LOC_Os08g37280	KKTAKHHVPVRLYGTCVHMYVDPWK	Y	72
10393	LOC_Os08g38310	DEDSSTSEASRKYMDVGRLDLPTTT	Y	922
10395	LOC_Os08g40800	RPALVKFMAAVHYAYLQMKMPGPGG	Y	719
10396	LOC_Os08g41020	KTEKGLLLTQERYAGDIIKRTGMSN	Y	1172
10397	LOC_Os08g42600	HLSLLSRYYKRAYQELFLLNDAKPP	Y	171
10398	LOC_Os08g42740	GSDEVVSRREQRYIVLTEKDINERQ	Y	79
10399	LOC_Os08g42980	GGGGAYHHHQQHYQPHHHHQHHQHW	Y	70
10400	LOC_Os08g42980	PRRQHRGGGGGAYHHHQQHYQPHHH	Y	63
10401	LOC_Os08g43110	AAKLWCDNLGAKYLSANPIFHARTK	Y	1368
10402	LOC_Os08g43560	DKALLEDPSFRRYVDLYARDEDTFF	Y	217

10403	LOC_Os08g44870	YLFGLPVGYLLGYYFNLGVGGVWGG	Y	424
10404	LOC_Os09g02480	LLVITPKWSTCHYLNSRIDKNAYDW	Y	86
10405	LOC_Os09g02700	EMNSKMVGSKPLYVALAQRKEDRKA	Y	393
10406	LOC_Os09g02810	LFWYWEDCLKQRYEKFVIALEDALK	Y	292
10407	LOC_Os09g03610	LQSSYQSSQQAIYQLQQQLQLMQQQ	Y	553
10408	LOC_Os09g06560	FLLSLFLKISLPYLRHCGREIRCIF	Y	26
10409	LOC_Os09g08390	GSSNDSSDGVDCYSYDHPFHQELYN	Y	260
10410	LOC_Os09g08390	LQERTKRKIKVLYGSGRDELLKVMD	Y	222
10411	LOC_Os09g09900	IYDTLWSRRDKEYIMCAYNQYAHWI	Y	334
10412	LOC_Os09g12730	GDKGSSSSMDDRYSQHADQRRFTER	Y	133
10413	LOC_Os09g13700	LRAARYQQSLRRYHQRHVRARSLCV	Y	1797
10414	LOC_Os09g14670	VEYFRLATPETEYGRMNIGSRPSKR	Y	755
10415	LOC_Os09g15770	KALARPASYTTKYFGCELGAQSKFD	Y	58
10416	LOC_Os09g15790	GQERYRAITSAYYRGAVGALVVYDV	Y	82
10417	LOC_Os09g16380	LLDVLAGRKTSGYIEGNITISGYPK	Y	855
10418	LOC_Os09g19560	PKVDVIISEWMGYFLLFENMLNTVL	Y	182
10419	LOC_Os09g19560	YKEDKIEFWNNVYGFDMRCIKKQAM	Y	236
10420	LOC_Os09g20460	SSSGFSLHGGSMYGFQELGSVGTSL	Y	56
10421	LOC_Os09g23350	VTAVRDGMNLIPYEYVIARQGNEKL	Y	471
10422	LOC_Os09g23570	AEVLGKGWLGTTYRATLEGGAAVVA	Y	375
10425	LOC_Os09g32020	SSVALCEKLQLWYALAKISAEKAAW	Y	417
10426	LOC_Os09g33600	LAYAQQSRERSNYEQAALVERMQEF	Y	232
10427	LOC_Os09g33960	AKVDDEISPETSYQAPNVVETVTDI	Y	188
10428	LOC_Os09g36030	QMGPLDLSASMGYLWDPGNRKVRAR	Y	240
10429	LOC_Os09g36160	FQLWQQQEQQPFYASNIIRFADDAP	Y	52
10430	LOC_Os09g37006	DGGWEDDHAPPAYAGNGYTRGRGRG	Y	190
10431	LOC_Os09g37040	SCFMIYVSAQKAYIEALDGWLSKFI	Y	505
10432	LOC_Os09g37520	RSELLALAIRHRYALADTHRAYAES	Y	32
10433	LOC_Os09g38200	SATKDLENLATDYFNDMFTADPTVD	Y	828
10434	LOC_Os09g39462	AKVAIKQRPQKHYHNNSSSGAHSSR	Y	181
10435	LOC_Os09g39900	CDLLCFLDAYSGYHQIRMAREDEEK	Y	906
10436	LOC_Os10g04580	FVELWDVSGHERYKECRSLFYSQIN	Y	105
10437	LOC_Os10g08570	SEQKALTKVKTVYLEHFPRSWDERN	Y	345
10438	LOC_Os10g15170	PTRLKEVQKLTGYMAALSRFVARMG	Y	195
10439	LOC_Os10g20040	VTLTSPSGDVLRYLVRLDFRATNNM	Y	1311
10440	LOC_Os10g21000	LRIGSISHQLSAYSLSGYGLNDAFD	Y	306
10441	LOC_Os10g21920	RTTVSFNTLIAAYCRDGVDAGPALQ	Y	260
10442	LOC_Os10g22950	LEEDFMHIVLVHYLETKGGKSRTRG	Y	135
10443	LOC_Os10g25090	SQNNLGGGQNIQYNLPNKKLERLNL	Y	117
10444	LOC_Os10g25220	QLEKLKVYECKAYLRMHKLRLSGNK	Y	143
10445	LOC_Os10g25220	SSKQTPTEQLLHYLPQFPHPQQHNE	Y	385
10446	LOC_Os10g25320	EGLSVTAESDDEYDSGNDDHPTLSA	Y	109
10447	LOC_Os10g26140	LQLDRVSMTLNLYRFYRDAVVQQGT	Y	241

10448	LOC_Os10g29400	VKKQWKILKGIPYNPYKNVQSDIIL	Y	702
10449	LOC_Os10g31520	AFVPPKEDTATPYESAKSTSNNDEI	Y	336
10450	LOC_Os10g31770	RYFVLETRLLSYYKRKPQHKMPKLP	Y	42
10451	LOC_Os10g32710	DSDDEMEDDGERYNRIPLSNEVVLR	Y	163
10452	LOC_Os10g33230	GAGYGSGNGGSGYPNAWADPSQGGG	Y	424
10453	LOC_Os10g35150	MSVAWYPIYHIPYQRNVKDLSACFL	Y	216
10454	LOC_Os10g35450	RFSKENVIGEGGYGVVYRGRLINGT	Y	200
10455	LOC_Os10g37740	PTREEIRCMNPNYSEFKFPQIKAHP	Y	372
10457	LOC_Os10g39200	SFNSKVSPSEVGYVQSREFSSELPE	Y	329
10458	LOC_Os10g41400	TPNRQGNDVGTQYRSGIYYYTPEQE	Y	188
10459	LOC_Os11g02080	GLWATAVGASVAYGRRKTPQMRLIH	Y	34
10460	LOC_Os11g03810	VGGTTYFTHVVIYRDDGPAVWWVSL	Y	164
10461	LOC_Os11g04070	TIAAAPHMFLNGYKNVLAVAVETEY	Y	248
10462	LOC_Os11g04670	HESGIHQDGMLKYKGTYEIISPDDI	Y	404
10463	LOC_Os11g06320	QKVLSYSKYDLGYFHGLKLTRTSSK	Y	444
10464	LOC_Os11g07280	IMVSILQLGQSSYLPHPIDNDSYDR	Y	608
10465	LOC_Os11g07500	MSEMIRPERALLYLVPGRLVKVRDG	Y	608
10466	LOC_Os11g10040	SLVYSEKKFVARYIDPYKQAAPTLA	Y	363
10467	LOC_Os11g10070	CANGMSLKYVKNYKRKAFWIYGCMV	Y	527
10468	LOC_Os11g10480	IYHFVGTSTFSEYTVMHVGCVAKIN	Y	152
10469	LOC_Os11g10710	GFSEERKLGKGGYGTVYKGEHKNGD	Y	29
10470	LOC_Os11g11390	EALEAARIACNKYMTKNAGKDAFHL	Y	75
10471	LOC_Os11g12810	LGRMPRAEIQGTYKIARRIEAEETG	Y	416
10472	LOC_Os11g15170	QYSWGSAVLAATYAGLCDACVRNSK	Y	1015
10473	LOC_Os11g17890	STWSQMEHEFHDYFKDASLMEQRPI	Y	524
10474	LOC_Os11g19060	VVSSHFTNGVGNYGSQHYGYNGWSP	Y	387
10475	LOC_Os11g23020	LTFTVLATEGLAYKPTPETRVHGAQ	Y	410
10476	LOC_Os11g23020	QEETKGKEVKKKYVAPQEFQLGMPL	Y	592
10477	LOC_Os11g23750	IFYFHAPMMNGLYIVNLDGCSVYNI	Y	297
10478	LOC_Os11g23960	GCVLMQDRKVVAYASRQLCPHENNY	Y	1177
10479	LOC_Os11g27820	SKTRYPQVQKLLYGHSVTVVTSFLL	Y	49
10480	LOC_Os11g29970	FTPKEVRYENLQYQYMQQPKYPIVP	Y	746
10482	LOC_Os11g31000	FRAAGEMLLSLPYLLPGQPGRSGSS	Y	27
10483	LOC_Os11g32470	VMFLPLPMISGYYAARFFTKKSLSS	Y	438
10484	LOC_Os11g32970	EEVKKEIFQALGYTEDHCAMNIQAR	Y	50
10485	LOC_Os11g32970	KLEKVFQCKDGGYRWGIMTSNGSES	Y	225
10486	LOC_Os11g35274	QVTHKNIVKVIGYCIGKKSLMMVTE	Y	86
10487	LOC_Os11g35510	PTLAWMRQLEESYTTSPAVAHAGLW	Y	14
10488	LOC_Os11g35850	PTNMENMSLEKLYLDCNHIADRIPR	Y	534
10489	LOC_Os11g36480	DFSDICPKEVSCYNCAQPGHTGLGC	Y	43
10490	LOC_Os11g37660	ALAVVAGLASNGYVASLLVSRYFRL	Y	50
10491	LOC_Os11g37700	ELIKELSTPPPGYQDLSFPTKYSQN	Y	1164
10492	LOC_Os11g38130	PYHPQRLRLFLYYRVTLSCSPSAGS	Y	176

10493	LOC_Os11g39190	CPGVDLIDNQIQYHWSYYRDVVISL	Y	83
10494	LOC_Os11g39660	WDSMIDKVKKIIYRHEGKELDEQCS	Y	500
10495	LOC_Os11g40140	GSEGADSSLQEQYNKELTLQEAETI	Y	183
10496	LOC_Os11g43970	AQQSSLSQRGPSYGYRGRVMLAFEE	Y	577
10497	LOC_Os11g47330	IVEPFVPHDQEYYLSIVSERLGSTI	Y	118
10498	LOC_Os11g48090	QISALIDRQEELYERESQLKAMLEV	Y	36
10499	LOC_Os12g01190	SQQAPTNNSISVYITYAKEEEAIRC	Y	170
10500	LOC_Os12g01200	TTVRSFPADNRKYCYTMNVRNRTRY	Y	81
10501	LOC_Os12g01940	DDSNAEDNPLYDYPEELSEDEDDDS	Y	288
10502	LOC_Os12g02540	LPDYARIVDDGLYRAIDIYLKAHPS	Y	428
10503	LOC_Os12g03880	RPFSYGLVITNVYDSGSVFSPEVLD	Y	196
10504	LOC_Os12g04000	SSLLGVIWSLVSYRWGIEMPAIIAR	Y	261
10505	LOC_Os12g04050	EEKTAFITPIGTYCYTTMPFGLKNA	Y	831
10506	LOC_Os12g04890	EYKENEVIQGAIYSRAEDMKEAVKH	Y	110
10507	LOC_Os12g06360	LRRYRLMLNPEKYTFGVPSGKLLGF	Y	148
10508	LOC_Os12g06670	DQGGNMTAETGTYRWMAPEVINHQP	Y	419
10509	LOC_Os12g06810	NRFPKENTKCGFYLHPAEGSPAPNI	Y	633
10510	LOC_Os12g07190	LASETGDGKSSIYPPVIGNSFAFKL	Y	402
10511	LOC_Os12g12370	LYSDNEMNIKYRYKDEELSFIPSLS	Y	227
10512	LOC_Os12g12480	GMFLGYPAHSRGYRVLVLKTNKIVE	Y	210
10513	LOC_Os12g13340	ASFTDDEEEEAPYPYRSDGNLRAGS	Y	209
10514	LOC_Os12g13770	KPKLTFEMLMAKYNKGSAGQRLDNQ	Y	449
10515	LOC_Os12g13770	LYGVEVNLGSLRYFKQDDLTVEDYK	Y	629
10516	LOC_Os12g14920	GRGLASISTRREYSLSPWQQRGGGD	Y	229
10517	LOC_Os12g15420	LECCSQERTYLRYYGLLGQRFCMIN	Y	595
10518	LOC_Os12g16430	VAKGFKQRYGIDYEDTFSPVVKAAT	Y	863
10519	LOC_Os12g18110	VQSIDQHTLVNFYGNIGKTLLSLEV	Y	216
10520	LOC_Os12g19290	DYIAPEVLLKKGYGMECDLWSLGAI	Y	342
10521	LOC_Os12g21700	AVAAARMNGEYPYRVGQPECQYYLK	Y	110
10522	LOC_Os12g21710	GVWNGDRRLASRYGEAFEVLKKRTS	Y	302
10523	LOC_Os12g21798	IEAHVDVKTTDNYMLRLFCIGFTKR	Y	133
10524	LOC_Os12g21940	SLSRRWSGTLGQYNMLDACTARPPL	Y	418
10525	LOC_Os12g22000	KKQFPLGKYLLGYLYQTLSTAVTKI	Y	133
10526	LOC_Os12g24080	SLLAHDGLSDNAYLLVAEVLKKIVA	Y	370
10527	LOC_Os12g24580	SSMEKQTYAQNKYGGITPKKPLISK	Y	23
10528	LOC_Os12g25690	ANYWKQVIKINDYQKSRFVNRVVSS	Y	305
10529	LOC_Os12g28640	NGIVLQGIKTRVYDRLMSHDKKWVE	Y	340
10530	LOC_Os12g29660	VINFSCPRDARTYLHRVGRTARAGR	Y	523
10531	LOC_Os12g31040	LLEQGTSIQVFSYVETTETLRRAIS	Y	413
10532	LOC_Os12g32410	VWTQIGEIVFPVYTTVPILAGPSMA	Y	78
10533	LOC_Os12g32710	IAPRVMEMLRLSYEDLTSEVQLCFQ	Y	441
10534	LOC_Os12g34510	AGLQFPVGRIARYLKAGKYAERVGA	Y	37
10535	LOC_Os12g35030	RKYKLEGYTTCKYLNVVWDNEANAA	Y	211

10536	LOC_Os12g35100	RFPDMNGYVDECYSVQRLRQTYAGI	Y	783
10537	LOC_Os12g35290	YHRTHLFDVVNQYRAIFNNDKSGSD	Y	282
10538	LOC_Os12g36180	FENIFPKSQSARYSDVHVDIGASGS	Y	333
10539	LOC_Os12g36730	PAGTSLQEIGESYFSELINRSLIQP	Y	381
10540	LOC_Os12g37840	TVIRDMPNVPLLYIIGAFIPATMIA	Y	293
10541	LOC_Os12g37860	YHRGGGTNRGRGYDVGKPSHVTNAE	Y	1389
10542	LOC_Os12g38000	RTEKPMLKAGNAYHKYRVKRNCWPK	Y	186
10543	LOC_Os12g38430	VPFERFGPVRDVYLPKDYYSGEPRG	Y	67
10544	LOC_Os12g39090	IAKKLGAERSEHYFHSLKKFLGGQL	Y	34
10545	LOC_Os12g39370	DEHGLVKPRYGRYYDEGLEKTRQTL	Y	120
10546	LOC_Os12g41290	KTAPGYCEKAGVYVSHLSQTSLRAV	Y	128
10547	LOC_Os12g41956	TVVGTARAARAEYVEALERRGEGNG	Y	198
10548	LOC_Os12g42840	SSREMVDDDDALYPYVCGLSGSTEI	Y	152
10550	LOC_Os12g44140	ATKLQRVRHLSRYFKQSAKTMEQQF	Y	139
10551	LOC_Osm1g00400	AENRKRAISPFVYKSSLYRNSTYCS	Y	277
10552	LOC_Osp1g00740	VMILNLMPYRASYPILKLVYSAAAN	Y	80

Table S4. Selected positive dataset.

No.	Protein Name	Sequence	Amino acid	Position
1	LOC Os01g01150.1	QHKSKKSEEGSKSRKDDCLLDDTLS	S	863
4	LOC Os01g01150.1	PSSPAQASRDAYSDADDDDDDRPHA	S	67
11	LOC Os01g01150.1	SRSPIKYRRSRRSRSYSPPVRHTRG	S	541
14	LOC Os01g01510.1	LSMQDFGLEDGESDEEDRAIKASNH	S	173
17	LOC Os01g01510.1	ARRIQKEEESKLSMQDFGLEDGESD	S	162
19	LOC Os01g01510.1	PTVKPPVMAASASDDDEIDAFHKHR	S	26
24	LOC Os01g01880.1	SSLKRKPTVICLSIERILVFFLFSR	S	139
26	LOC Os01g01960.1	DMDHDDQDAKAESEGEAEGTTETHD	S	987
27	LOC Os01g02700.1	RRSAALQAMAAFSVLALVVPDQVQG	S	18
28	LOC Os01g03500.1	AKENANLIDADDSDELRSICSESDE	S	131
33	LOC Os01g03650.1	AARASSAAVPAVSDDLVLRIAEQLE	S	51
35	LOC Os01g03760.1	ATASAYSDDDDDSDEAQATRPEGEV	S	108
36	LOC Os01g03820.1	KPLIGAARIVINSIHIKGDLLLLPI	S	201
37	LOC Os01g04100.1	NRAGDAIAAARRSPEQLDGVKERLL	S	70
40	LOC Os01g04330.1	FTRFDADGDGRISPSELAAVTRAIA	S	44
42	LOC Os01g04650.1	RLFLFPAKPESSSSLGSLLDDSSKS	S	171
43	LOC Os01g04650.1	ISGSLDGIPRGISTDSASVNCLLGL	S	207
44	LOC Os01g04650.1	DGIPRGISTDSASVNCLLGLEDDSS	S	212
45	LOC Os01g04650.1	SLDGIPRGISTDSASVNCLLGLEDD	S	210
47	LOC Os01g04650.1	SVPDSPMIDKNSSFGSTSSAPSLSN	S	276
50	LOC Os01g04650.1	RGGDSWDEPFPSSAAAAAAAAGGGR	S	43
51	LOC Os01g04650.1	STESSPRSRGGDSWDEPFPSSAAAA	S	35
52	LOC Os01g04650.1	SISPSDASSRVFSDDDKSDHGGGGG	S	360
56	LOC Os01g05010.1	RIIDAEIKFAEESDDHDRVEEIPDN	S	81
59	LOC Os01g05420.1	EDDLGDDWNRGRSPTPVHGDDGAGS	S	47
60	LOC Os01g05420.1	DDTGVDPADRYGSDNDGHSPRHYPQ	S	223
61	LOC Os01g05420.1	PADRYGSDNDGHSPRHYPQAEEGEE	S	229
63	LOC Os01g06270.1	STPKAETVVSPKSPAKPDQPLDLDS	S	488
64	LOC Os01g06270.1	QVPSTPKAETVVSPKSPAKPDQPLD	S	485
67	LOC Os01g07110.1	SPMFQTGSGKMVSLSKGSIQKARAV	S	178
74	LOC Os01g08260.1	GITNATSVQPVISIERFVSYRERAA	S	413
77	LOC Os01g08420.1	NGAVATRLSSNKSISVPAGFDQRTV	S	711
78	LOC Os01g08430.1	RNKDGHLEHSVNSGEVAVIRQPRGV	S	114
81	LOC Os01g08970.1	LERIKNQAGDEESDEEDEDFVADKD	S	480
82	LOC Os01g09280.1	VKQKEPEFARHLSDLQLRKHEESEF	S	221
84	LOC Os01g09550.1	QRRRDSGSGSCSSTRDHEVSATSYS	S	302
87	LOC Os01g09620.1	AAIRRGSWPGVGSPVNDVLASFRQL	S	258
88	LOC Os01g09620.1	GWSYPSSSAVYGSPKAATGLYSLPT	S	294
90	LOC Os01g09620.1	ASFRQLRLNKVKSSPSGGWSYPSSS	S	277
92	LOC Os01g09850.1	RCDCGTLFSRRDSFITHRAFCDALA	S	191
96	LOC Os01g10690.1	AQICAKEEANADSSDDEQAVHYKRG	S	295

100	LOC_Os01g10820.1	RASKVLEQLSGQSPVFSKARYTVRS	S	46
103	LOC_Os01g11040.1	MDKAEHVQSKCISPEQQEIDHREME	S	388
104	LOC_Os01g11040.1	SNTPLHTRLNGTSADDLGDDLKQSS	S	330
106	LOC_Os01g11330.1	AGVTIKRAKQNCSAPKKRTMGAVLG	S	556
108	LOC_Os01g11580.1	TADDNERYVGSSSPVHLEDQKGENA	S	302
109	LOC_Os01g11580.1	EIDNSKVLERSDSTVHAAGMEATPK	S	126
110	LOC_Os01g11920.1	ANYGHGSIVRSRSGGVVVAEEDAVV	S	179
114	LOC_Os01g12390.1	ALAFDLEDLLRASAEVLGKGAFGTA	S	341
116	LOC_Os01g12480.1	YRKPAAEKSDDTSDDDEEPDIDIGK	S	68
117	LOC_Os01g12650.1	ADKLHIGGDGSSSDSDADERKQPKP	S	34
120	LOC_Os01g12650.1	IADKLHIGGDGSSSDSDADERKQPK	S	33
122	LOC_Os01g12660.1	SSFPKLKRNASTSSDMSSLASQGPP	S	218
125	LOC_Os01g12660.1	GSSTDSSFKRSISETTLEKVSGLLG	S	161
126	LOC_Os01g12660.1	GGGSSTDSSFKRSISETTLEKVSGL	S	159
127	LOC_Os01g12780.1	QMAAVDDGKLRASSRSSTQPPPRRR	S	111
128	LOC_Os01g12780.1	MAAVDDGKLRASSRSSTQPPPRRRR	S	112
129	LOC_Os01g12880.1	FNGQSPAGRNMRSPDQQDEAESSAG	S	160
131	LOC_Os01g13270.1	LVMEYCSGGNLHSLRQRQLNKHFNE	S	226
132	LOC_Os01g13470.1	KADDSSVAKDTNSEPEAQLELEKGM	S	187
133	LOC_Os01g13530.1	IISTQTKETRAGSPIPNSNPLARSA	S	242
135	LOC_Os01g14050.1	EKNAGEPGRSLTSILSRKISRKKPE	S	603
136	LOC_Os01g14050.1	AGEPGRSLTSILSRKISRKKPEDKL	S	606
138	LOC_Os01g14514.1	TATRRLPSPRGGSVHARCSPFRNLV	S	113
144	LOC_Os01g15020.1	SPIMNGGDPASRSIDIKPRISEERP	S	723
148	LOC_Os01g15039.1	VAQDGSGRWRTVSEAVARAPSHSRR	S	319
150	LOC_Os01g15460.1	ISTKDQSVNQVTSPVAASEPVGSIL	S	435
151	LOC_Os01g15600.1	DDVGAGFIAGHGSQSASGPEVDVER	S	203
152	LOC_Os01g15600.1	AGFIAGHGSQSASGPEVDVERLVDL	S	207
153	LOC_Os01g15630.1	PTDADSMRKARRSPGYQLYRQVSDS	S	179
156	LOC_Os01g16100.1	QSHSHPAKTLRASPPPPSTAGSAPK	S	50
159	LOC_Os01g16110.1	TNNRSHYGGVSESPPSNSIGYFYGS	S	695
160	LOC_Os01g16110.1	LSSSVTSLNKGLSNISIDNKPKSIS	S	476
162	LOC_Os01g16110.1	YWAFHHFRNQDSSPIIKHPELERLL	S	876
169	LOC_Os01g16330.1	SSRPSRSLVGNMSSRTSRAVQNNQP	S	229
172	LOC_Os01g16390.1	LRYKLEPPAAASSSGEYAPRCYDDL	S	154
173	LOC_Os01g16390.1	RYKLEPPAAASSSGEYAPRCYDDLD	S	155
174	LOC_Os01g16850.1	GREKISCRGFHSSFRPTDSGLSLNV	S	55
176	LOC_Os01g16870.1	SQFIKFDEMSETSSSHGGHTSAGSA	S	873
179	LOC_Os01g16870.1	MSETSSSHGGHTSAGSAPVPELPRL	S	881
184	LOC_Os01g18890.1	AHSFGRSHCSAFSFRLYPQVAPDMD	S	115
188	LOC_Os01g19894.1	AFVQKREFRVKVSNRTTYDVKCIQG	S	845
189	LOC_Os01g20940.1	PICSKNVGLSTSSLKLHLQKAHKRL	S	870
190	LOC_Os01g20940.1	SSAGSRGSSDSVSPLSRESWSNKYF	S	590

193	LOC Os01g21590.1	GHHKRRRGDDVSSAGAGDDDDEDGV	S	380
195	LOC Os01g21590.1	DEDGVKRARGAASAAGGGDDEGPSA	S	400
196	LOC Os01g21960.1	SFRYMDKDLGFQSADEGGSGTFRHN	S	143
197	LOC Os01g21960.1	KDLGFQSADEGGSGTFRHNSAHAIT	S	149
198	LOC Os01g23530.1	TVPTVVPARPPNSEGERKSTNFHPS	S	21
199	LOC Os01g23540.1	GNAKLAVQCDKRSLDMVEHTPSPPA	S	763
203	LOC Os01g23590.1	EDYFNEDSDEEDSVRRTKHAQKQDG	S	705
205	LOC Os01g23640.1	EEELSQLSIKQPSPPPVLAELERRP	S	307
212	LOC Os01g25610.1	DEDAKFKLCKVRSVQFGQKGIPYLN	S	128
213	LOC Os01g26940.1	PGKHETNRRRASSRELSPHGRQNSP	S	344
215	LOC Os01g26940.1	RSPRTHSPKELDSPRPENEVGKLAT	S	307
217	LOC Os01g26940.1	SSHRHHSPRRPWSPPANRKTGLGKP	S	603
219	LOC Os01g27790.1	RSGRRDRRRKKGSARFDDEGHVLAV	S	440
220	LOC Os01g27790.1	DDEGHVLAVEGASRAPRKGRPASDK	S	456
223	LOC Os01g31580.1	LDFSHATLQRSSSTPNIPMPDPKAA	S	115
224	LOC Os01g31629.1	SSKSITVEGQRKSLEETQQLLDQRK	S	120
226	LOC Os01g32130.1	LLGEGRLKLMLNSDGELEEEQETHA	S	195
229	LOC Os01g32870.1	TMVNSVGAGSSSSRYPTESPENGNI	S	368
240	LOC Os01g34330.1	RRGRKPKRPAEESEEDEEEEEEEAK	S	113
242	LOC Os01g34410.1	LHPGAQQPVNIDSGDEEAPNVRTEK	S	139
244	LOC Os01g34780.1	KDEMDIDAVDADSHGSKDKKREKDK	S	350
247	LOC Os01g35050.1	NTPVPSKYNGSESPSLAEIVNDQRL	S	756
250	LOC Os01g35050.1	RRNTPVPSKYNGSESPSLAEIVNDQ	S	754
251	LOC Os01g36790.1	KKKIFRNKKRSKSFIDIYGDGVPVR	S	308
253	LOC Os01g36860.1	SRSPGASRRHERSATGSGSALPDSG	S	656
254	LOC Os01g36860.1	GASRRHERSATGSGSALPDSGHGER	S	660
255	LOC Os01g36860.1	KAAEVSKNPDPSSPPHHGKTREDEE	S	754
260	LOC Os01g36940.1	KKHQRQRQQRPLSPGCCGCARCGCR	S	47
266	LOC Os01g37825.1	DFPVPSHVEPRFSCFVESEAAGSAV	S	294
268	LOC Os01g37825.1	RFSCFVESEAAGSAVVVSCKMPADR	S	304
269	LOC Os01g37832.1	KSISVVGGADGTSPSRMRAFINREG	S	92
273	LOC Os01g40050.1	NEDHQLTKSRKFSMKKKGIGSEASA	S	358
274	LOC Os01g40050.1	AHRKDKELPVHRSDDDNDDDNEDHQ	S	338
278	LOC Os01g40340.1	GRPTSLHDRGRDSDDDDFRDRDYDV	S	404
286	LOC Os01g41430.1	SKDFAARGAAELSPDADGCLRWLDA	S	271
287	LOC Os01g41550.1	PSQISRRFGRSFSYCLVDRTSSSAS	S	295
288	LOC Os01g41610.1	ELSKQLFYTRLASLPGRYEAFWKEF	S	61
294	LOC Os01g42250.1	ARIPTEHIDLDKSPERHEAREKVVV	S	154
296	LOC Os01g42380.1	ASMRLGGSMRGDSGSMWRRGDDVFS	S	23
297	LOC Os01g42380.1	EIQKVASMRLGGSMRGDSGSMWRRG	S	18
304	LOC Os01g42830.1	KSAFFEQGLNKLSFNEKANSKEAPV	S	199
312	LOC Os01g43170.1	RDYTNPYLPVAPSAIDGSGPDGKKQ	S	220
316	LOC Os01g43330.1	PLEDTRNASDAASLSEISEEKKTEE	S	575

319	LOC Os01g43330.1	QVEPSDARVSPCSPVLEDKLVDPLC	S	394
322	LOC Os01g43330.1	VDPLCSQEKDNNSEDLGMANISDVN	S	414
324	LOC Os01g43420.1	DQPGVRHLSSFSSFDNPRGRQIQKA	S	268
328	LOC Os01g44310.1	ETTSGDFVGCGKSWDGGDIAELRME	S	620
329	LOC Os01g45550.1	EAALPDGLTKMGSSSTAELHPKVVD	S	421
330	LOC Os01g45550.1	VALGGGGGGENFSFGGGKTVDGAEA	S	393
331	LOC Os01g46070.1	NGVEEVLGLGQLSDFEKEGLENLKG	S	313
332	LOC Os01g46710.1	IVEPDQGMEEVASPTIKEPDEDIEV	S	1157
335	LOC Os01g46710.1	RSSSHRSHRRGGSVERSESEGDEAG	S	20
336	LOC Os01g46710.1	HRSHRRGGSVERSESEGDEAGGGAG	S	24
337	LOC Os01g46710.1	SHRRGGSVERSESEGDEAGGGAGAR	S	26
341	LOC Os01g46840.1	IATVHPLSIRNLSGPELRNLNKGGG	S	527
342	LOC Os01g46980.1	LVDHDVYLPPSPSSHDSHERFCSGG	S	177
345	LOC Os01g48180.1	MLSNAKAATVEESPRSSGADEWGLE	S	1464
347	LOC Os01g48180.1	EKIRAFQNVISDSEAEKEANDAERD	S	653
348	LOC Os01g48180.1	FERTAPSTYCVKSPYRKDPADSEVV	S	623
351	LOC Os01g48280.1	PRRIIKETQRLLSEPAPGISASPSE	S	19
353	LOC Os01g48320.1	PMMRRRLESPSRSPSPPSPTPAVAL	S	145
354	LOC Os01g48320.1	MRRRLESPSRSPSPPSPTPAVALPL	S	147
360	LOC Os01g48330.1	NGSTQKAMSLPSSPHEYRAQISETI	S	491
362	LOC Os01g48720.1	PYIMEDASNANDSPRSSISGAKRKH	S	226
364	LOC Os01g49190.1	NSEVSALLGRIPSAVGYQPTLATDL	S	355
365	LOC Os01g49200.1	GLDIAGLPIKKLSFNASTLRETETP	S	576
370	LOC Os01g49350.1	VEDTGKYQAAPQSPSSPAGSAVMHA	S	247
372	LOC Os01g49350.1	DTGKYQAAPQSPSSPAGSAVMHAAT	S	249
375	LOC Os01g49529.1	LLKYSGSGGTPRSMGGDMESGSVKD	S	353
376	LOC Os01g49529.1	GGKDKKGPDPPLSPDTVHAQWDSRQ	S	682
378	LOC Os01g49640.1	NPDLRKYINSQNSRKVAAACSVPAP	S	89
383	LOC Os01g50310.1	AKSYAAVVAEKTSPNGSVAEDEVTV	S	83
389	LOC Os01g51010.1	GTTTKTAKSYFDSDDSEDELAQRKE	S	947
390	LOC Os01g51010.1	TKTAKSYFDSDDSEDELAQRKEVQT	S	950
392	LOC Os01g51010.1	REEFNHSRERKSSVSGSNWNIEFKD	S	338
394	LOC Os01g51020.1	FSKEPNNLYNVHSYKHSGLANKKTV	S	45
396	LOC Os01g51200.1	TSEPSRTRATDASPGAFRRTSGPQK	S	416
398	LOC Os01g51300.1	AEHISQFNEEARSPFVKKYKTIIHP	S	98
399	LOC Os01g51390.1	PLTNLPELSWFRSHTYSDDEFSSRT	S	485
402	LOC Os01g51640.1	EEVPTVESEENESPQSKHCTSVEVI	S	335
404	LOC Os01g51890.1	PFISEKALRSCLSFKSAHGDSNAFP	S	379
406	LOC Os01g52390.1	NAERDERAKKSVSINEFLKPAEGER	S	318
409	LOC Os01g53000.1	GTLAELDEERAGSVTSDVPSSLASD	S	65
411	LOC Os01g53000.1	AELDEERAGSVTSDVPSSLASDRLI	S	68
415	LOC Os01g53680.1	DSYPNLRALRNASAMSLPDDDAAYA	S	67
418	LOC Os01g54560.1	PGGGGGGRRRSGSFGLKRMSRVMTV	S	34

423	LOC_Os01g55260.1	KEAAMQPLRRLMSVLEARDELVPEE	S	326
425	LOC_Os01g55280.1	SSPKLADRHSPRSPLHEKKRAGTRV	S	53
426	LOC_Os01g55280.1	VVRTESGDKENQSAAGAEDGEEVSC	S	174
428	LOC_Os01g55350.1	TLKRIRDPGFHVSPRAHLSKDIMDS	S	873
430	LOC_Os01g55420.1	LGAPPRPAAAAASPSRAALPDKPWV	S	70
431	LOC_Os01g55490.1	AIVDYAHDETAMSPEQEDGEINGID	S	139
433	LOC_Os01g56000.1	THDSAGGAATLTSPGQNFGKAKHSY	S	522
435	LOC_Os01g56100.1	QILKAREVLLQSSPTKKRLGPDDGS	S	262
437	LOC_Os01g56140.1	KVNGGEITSNCLSALEEKCSKNEHD	S	876
439	LOC_Os01g56140.1	QVIFKRSPEKSASHDQFVQNGVPKK	S	805
441	LOC_Os01g56300.1	QQGDAKPLSRTSSYPQQPLQHRASE	S	169
442	LOC_Os01g56490.1	EHSDVTPKRSCLSNQNRNSFDKAVK	S	1095
443	LOC_Os01g56800.1	ISMKVDTSEEGASDVSDNLDGSIEI	S	546
445	LOC_Os01g56800.1	RAALEPLPCQPVSPKDDKCSQSRTK	S	390
446	LOC_Os01g56800.1	GNLVDSEVTSISSSGRLNLADHYYD	S	1179
447	LOC_Os01g56910.1	SAIAKTSFRRKQSQLQADSATIEPI	S	871
450	LOC_Os01g57450.1	AQAASAAAIHPTSPRYFFSSLAGTN	S	43
451	LOC_Os01g58550.1	ARPLKKSLQKSLSMPASLDNAAAAT	S	45
452	LOC_Os01g58550.1	LDNAAAATTCAASPEKSRAADFARA	S	62
456	LOC_Os01g59670.1	ASHRDMFFRRHDSFNIGRTDATLER	S	579
457	LOC_Os01g59670.1	ESSEQLDVIDAKSVDDIYAALKEHT	S	1172
460	LOC_Os01g59850.1	AFFDGKLGSFLYSPGRLRDQMHEDR	S	195
463	LOC_Os01g59990.1	YPGKGIRFIRADSQVFLFANSKCKR	S	28
464	LOC_Os01g60040.1	QNIIPPMDERALSGLQARMERLKSG	S	1981
466	LOC_Os01g60040.1	SPKFAPSPVHTKSINNKTDCNEDDA	S	1875
467	LOC_Os01g60190.1	FGHVTFFWNGNRSGYFDETKEEYVE	S	374
468	LOC_Os01g60190.1	AVGLPSEDDMGNSEVGHNALGAGRI	S	81
469	LOC_Os01g61180.1	LERTVRTLDRQISQFVTMDRLIWAD	S	108
470	LOC_Os01g61370.1	FSWESSPGVPKRSSACMHMAQEIMP	S	83
471	LOC_Os01g61370.1	SWESSPGVPKRSSACMHMAQEIMPP	S	84
472	LOC_Os01g61430.1	PTQYPNTLHSAKSLEVEDPSWLVDK	S	99
473	LOC_Os01g61590.1	EVAIMRHLPKSASIVSLREACEDEG	S	137
477	LOC_Os01g61780.1	GTSEMDPDSEPDSARSRHDDFNFSE	S	689
482	LOC_Os01g61860.1	KNFLDSQAEYDESSGEDQDAKVSPS	S	61
486	LOC_Os01g62310.1	FSDPPSPLSPPLSPASAAAAALANA	S	31
489	LOC_Os01g62650.1	SKDGTLGDSNSISQSKFQSEISDHQ	S	371
491	LOC_Os01g62650.1	VRINSNEKKLNCSSSFNTASSKAVE	S	333
493	LOC_Os01g62650.1	INSNEKKLNCSSSFNTASSKAVEPD	S	335
494	LOC_Os01g62650.1	ATPDSHYVPRVPSPGLPPVGVRINS	S	313
499	LOC_Os01g64000.1	MEEAKVVDSGSGSGDAGGSGLCRQG	S	105
501	LOC_Os01g64250.1	TTVSPYALARSPSVSAAEADGDDGV	S	50
502	LOC_Os01g64280.1	MSQERSQSQSPRSPASAAGVPFLSI	S	13
503	LOC_Os01g64450.1	DSAELTRFELGGSDGAKRGQLLRYN	S	97

508	LOC_Os01g64970.1	KSSRTGYWSDAGSDEEEKEEEERPE	S	318
510	LOC_Os01g65230.1	VRSHISRLASIDSFDSRWQDFLEPG	S	389
512	LOC_Os01g65330.1	RSPTQRGHTITLSPNKASSNSSPLL	S	554
514	LOC_Os01g65900.1	WADPMKNVILPNSPKESESSISCAG	S	162
516	LOC_Os01g66130.1	ITDGSASADKSGSPEHCQLVAALHP	S	327
518	LOC_Os01g66140.1	NDRKSVIVIDSDSDEDEDPHPEQHE	S	753
520	LOC_Os01g66520.1	HLSSEKCEEQDGSDIDDDENSRPSF	S	288
521	LOC_Os01g67126.1	NLKKLTYEQRKASLVERLNALNSSA	S	277
522	LOC_Os01g67126.1	KASLVERLNALNSSAGADDDDEEED	S	287
525	LOC_Os01g67170.1	LVQNDGNSIALDSDDEDLIRGSHKR	S	422
526	LOC_Os01g67250.1	SAQAVDDMKGELSDIVHDNVNAFDN	S	857
527	LOC_Os01g67250.1	LMEETVPSRVHESPVLSPQRKASPS	S	316
533	LOC_Os01g67550.1	NPLGSLRLARLRSILDEGKSLQLQF	S	519
537	LOC_Os01g68120.1	VKIASSRESGNLSPGFVPNEEINEY	S	507
542	LOC_Os01g68810.1	DKFRPMDHDKSASEEQILSRPEKSK	S	1493
544	LOC_Os01g68970.1	SNSFIKNFEKSFSSTFRTLHLVNEI	S	140
545	LOC_Os01g69030.1	DTPMKKKFQRNFSELTVSWSDENKE	S	117
553	LOC_Os01g69130.1	AHGLSSSFQDGSSPYSTPKQPRSRK	S	787
561	LOC_Os01g69990.1	NSGKLGLRSSSGSMLDMQRGDFSGI	S	1321
562	LOC_Os01g69990.1	SVEQHEHLERSLSLHERLHRGGQGI	S	1080
563	LOC_Os01g69990.1	VDIPVSTNKEAGSFISPSGTSVDGP	S	1452
565	LOC_Os01g69990.1	VHPQQHRLQEQLSGNLARLDRHWSD	S	1156
567	LOC_Os01g69990.1	HFDLLQSLQRSSSVEQHEHLERSLS	S	1068
571	LOC_Os01g70020.1	SEVKHIIEEVINSMSDDEEGEEDNA	S	475
577	LOC_Os01g70320.1	LRAGSPVYGRQRSGSSTGSSSPGGV	S	16
579	LOC_Os01g70330.1	NPGIQLPLKKAVSFQDDNRHTVGPS	S	315
580	LOC_Os01g71000.1	LDDLDMTGSQMGSPAQTGRKRETPR	S	444
584	LOC_Os01g71050.1	ETSFLREVPAPVSPVKETPILREVP	S	216
586	LOC_Os01g71230.1	EQFKAPDLSNVISKAEPSAAAQDDE	S	144
589	LOC_Os01g71990.1	GKHPGQLKDMVTSPAGTTITGIQEL	S	246
591	LOC_Os01g72320.1	VHKDGSDHYRLRSPFGSPPKNALDK	S	177
592	LOC_Os01g72320.1	GSDHYRLRSPFGSPPKNALDKAFSD	S	181
594	LOC_Os01g72710.1	LAQLGRKLESLPSDLAAAVEGGRVT	S	121
595	LOC_Os01g72890.1	PPPKKASPPRKASPAPESVVLHIDH	S	101
596	LOC_Os01g72890.1	ASPPRKASPAPESVVLHIDHLSRNV	S	106
599	LOC_Os01g72890.1	PRRRSPGPIRRRSPPPPPRRPRSPP	S	330
600	LOC_Os01g72890.1	RRLRGSPSPRRRSPGPIRRRSPPPP	S	322
603	LOC_Os01g73160.1	GEKGGAPAEFQPSFRGSRPGFGRGG	S	155
604	LOC_Os01g73300.1	KIILHKTIDSVLSRGERLDSLVEKS	S	178
605	LOC_Os01g73620.1	FEASAVKSNFGSSPDFKHSNHSSVH	S	825
607	LOC_Os01g73644.1	VLDSVVGMDFLLSDSGGNVGADGKT	S	55
608	LOC_Os01g73644.1	DSVVGMDFLLSDSGGNVGADGKTRY	S	57
611	LOC_Os01g73950.1	ADAVRALEDGSDSDASAELDEKLEG	S	154

614	LOC_Os01g74030.1	IGMPGLEIGRQNSFSGIFQDDSYQH	S	146
615	LOC_Os01g74030.1	ASPDPELVRRVPSPCLPPIGVKLGA	S	236
616	LOC_Os01g74030.1	QYMDQTNGNQRHSYMKRSEQGHVKV	S	324
619	LOC_Os01g74370.1	RHSRSGDGNGWASPERTMVWRTELK	S	18
621	LOC_Os02g01020.1	RALSRDSVYDLDSEDEQWLTQLNHS	S	650
627	LOC_Os02g01380.1	AGGNGGEEAKGSSELLDAVVKYPDV	S	297
629	LOC_Os02g01490.1	SPLMPGKQSHSRSNSNSSVQFNSLT	S	1494
633	LOC_Os02g01510.1	SRSRSRDMKKASSLSELGFDAEGAS	S	45
636	LOC_Os02g01960.1	KQKSLENLFVTNSQRVKKDAGRRWS	S	581
637	LOC_Os02g02050.1	DSQTKHQLETVLSQRGETCGHLGDA	S	1861
639	LOC_Os02g02290.1	PPAPSSSSKKLRSLSALDSRPGALS	S	1707
641	LOC_Os02g02290.1	IYREIDDDDFEESDDDSEERNTSSL	S	1615
646	LOC_Os02g02410.1	IKLIKKKYSKDISKDNRALGKLRRE	S	283
647	LOC_Os02g02570.1	RSCEKLESSASSSPQEANVANKGKI	S	320
651	LOC_Os02g03040.1	GSPDYGRGGDRGSPDYHRGASPQGG	S	229
656	LOC_Os02g03080.1	SNLGKWQENFVSSQGRQGGGRSGYS	S	858
658	LOC_Os02g03080.1	AEEDPEKIAMSESDEGDDDEEDDQD	S	214
659	LOC_Os02g03740.1	DTKAFSALSLSSSSRDTQDGSKNKP	S	236
660	LOC_Os02g03740.1	ESDTKAFSALSLSSSSRDTQDGSKN	S	234
664	LOC_Os02g04050.1	TEVMNLLESAGFSRSNPYYVVQQGK	S	131
667	LOC_Os02g04270.1	AEVTKDGDDSDDSDGMDDDAMFRID	S	995
668	LOC_Os02g04450.1	SFGFLSQIPRNFSFSDLTEDFSQSA	S	267
670	LOC_Os02g04660.1	RSNHNVSQGGVLSGDENHTEDTAVR	S	275
672	LOC_Os02g05330.1	LGVKVHACVGGTSVREDQRILASGV	S	148
675	LOC_Os02g05450.1	YTSRRALQQRTESSSELISVSKRAT	S	23
680	LOC_Os02g05840.1	MPGGSGLDQNNGSPKANSGGQSDPS	S	458
681	LOC_Os02g05900.1	DVGEDPEERQMVSLNRLFEKHRDVS	S	29
682	LOC_Os02g06430.1	EVAPQHPPERTVSLPSDSGNLGVKP	S	625
683	LOC_Os02g06740.1	SRLSSMCLIPKLSIVKGSKCHSCVQ	S	335
684	LOC_Os02g06920.1	GQQVKATTSRSFSFTKVINSLSAKR	S	129
687	LOC_Os02g07070.1	GRNQRDGFYDDNSFGRRREYDWDER	S	218
689	LOC_Os02g07070.1	DSYERDRDYDRYSYDSDYEKSRRDG	S	138
691	LOC_Os02g07190.1	LKRAGLDDSLSESFRRTEIFKDKSA	S	809
694	LOC_Os02g07260.1	NPKKPFAAIVGGSKVSTKIGVIESL	S	201
696	LOC_Os02g07870.1	FGCGKTVISQALSKYSNSQAVVYVG	S	264
697	LOC_Os02g08190.1	AAKSSAAAATPASIDAIDRHLRSLH	S	56
698	LOC_Os02g08190.1	SIWEDATDGFVPSPSRSPMPSRSPI	S	240
699	LOC_Os02g08300.1	LVVMLSKSKASGSSGALSSLTSSTP	S	82
703	LOC_Os02g08360.1	LPASKWSREDDVSDDEDRKGGRGLG	S	774
704	LOC_Os02g08360.1	RRRLQSEYGLSFSNDGANSRRSSER	S	873
707	LOC_Os02g08360.1	DEDRKGGRGLGLSYSSGSDIAGDSG	S	788
712	LOC_Os02g09920.1	SFQDNLEASSTKSIELDADNNEMDT	S	422
713	LOC_Os02g10020.1	ECAEEPVPQVPKSTKPSILKLRQAT	S	361

719	LOC Os02g10510.1	RDEEKLSKLASLSQGAAGESSTARE	S	337
722	LOC Os02g10790.1	CAENLRSANLLQSPGSSNLAMNGNK	S	478
724	LOC Os02g10970.1	SKTERNADIALTSNRSKSRFPIFLH	S	408
727	LOC Os02g10970.1	SKRHHRGRKSKASSQVSGENHTALA	S	345
730	LOC Os02g10970.1	SATPRSLAQEDKSPKENGDTRTNGV	S	381
732	LOC Os02g11750.1	NDRVKMSEKELQSLIDDAMGEVDIG	S	314
735	LOC Os02g11750.1	SRDLSVSSDDLHSDAKRQLNKAIQM	S	226
739	LOC Os02g11820.1	DDRNSRYSYGERSPGYEHNDYKKSP	S	186
740	LOC Os02g11820.1	NRRSDGNWGGSRSPPYNESYSDRRS	S	156
741	LOC Os02g11820.1	SENRRSDGNWGGSRSPPYNESYSDR	S	154
742	LOC Os02g11820.1	PPPQMQRTSTASSIGSSEGTSEQIK	S	327
744	LOC Os02g11820.1	NGSPNYQKETDGSSPVVRPVRDILG	S	246
747	LOC Os02g12360.1	DQDISGAGKKWDSSEEDDLDGYKEL	S	640
750	LOC Os02g13130.1	DHYSSKRKYDDPSPPPRRTGFSSAP	S	16
751	LOC Os02g13130.1	LINEVLAEADAASSGNLSSRKYNAP	S	220
753	LOC Os02g13170.1	KELLLKDVARANSIEVSILKDRLYK	S	419
758	LOC Os02g14530.1	NWWSSHAEHKLGSISLTKQERNSGS	S	31
762	LOC Os02g14530.1	TSNMRRGRRRSISFTPEIGDDIVSA	S	401
763	LOC Os02g14770.1	AAAGKAAMERHQSIDAQLRLLAPGK	S	15
765	LOC Os02g14780.1	DEQPKQVIWDGHSGSIGRTATQALS	S	513
766	LOC Os02g15060.1	SHRTCPMCRCDLSPPREVAAKEATA	S	151
770	LOC Os02g15220.1	KNPKPPPNPSPSSSPLAQTLASIRR	S	64
772	LOC Os02g15220.1	SPSSSPLAQTLASIRRSIRRPEDGP	S	73
774	LOC Os02g15310.1	SYSPAPRRRDDYSASPQRKDTHRAK	S	177
777	LOC Os02g16090.1	QPVFDKISGRVASWRGKNMAVAGRT	S	786
779	LOC Os02g16620.1	AGKAHVELSRQYSVLDAKVLSKIPR	S	209
781	LOC Os02g17980.1	LLDRVERRLTASSMFLPQGGRLTLI	S	222
783	LOC Os02g18660.1	TTAASDDGTLVGSKKGKADNGKLDG	S	447
785	LOC Os02g18660.1	GGDEEGEEVEGDSSESEPEPEPVKK	S	98
786	LOC Os02g18660.1	GDEEGEEVEGDSSESEPEPEPVKKE	S	99
787	LOC Os02g18660.1	EEGEEVEGDSSESEPEPEPVKKESA	S	101
788	LOC Os02g18660.1	PPPQKPSGRGAASSDEEEEEEEDSD	S	59
791	LOC Os02g18660.1	SKYNLAPEPSPSSKSGKALSRWTTD	S	194
794	LOC Os02g19150.1	YKRIHCELLSSRSAGDCSETDSCAS	S	191
796	LOC Os02g19170.1	PTLASMLKKKVTSKDRIAERLLNAR	S	685
799	LOC Os02g19420.1	DLLDPELKRHAPSELRKKREMFQNL	S	33
801	LOC Os02g19860.1	VAPPSRRPLKRMSSPERWEAKQLIA	S	418
803	LOC Os02g19860.1	FLQGQSRFSIDMSPVKIFKNPEGSL	S	483
808	LOC Os02g22090.1	DGQTACSKNQGLSDGEIGNGKSDFV	S	252
809	LOC Os02g22210.1	KSEIAKLKVQLSSEFEMKDLGAAKK	S	835
811	LOC Os02g22370.1	QAQKRNALGRQPSQEILSEEKTLPL	S	531
813	LOC Os02g24080.1	GEIRGPMLTRDDSFIRKGSHLEQRH	S	217
815	LOC Os02g24080.1	KPSRSSNPFDSDSDDGGREQRPARA	S	63

816	LOC Os02g24330.1	PPPERRSFGRRGSPVRGEDNGSRPL	S	386
819	LOC Os02g25060.1	VDVTYKPFKEGDSDVDTSDESGTIE	S	396
824	LOC Os02g26140.1	PPGKVQVAAAAASVSKTRRCTFSPS	S	316
826	LOC Os02g26349.1	PDAGRHKRRRDGSPAAAATDHKDDK	S	103
827	LOC Os02g28810.1	RTLDAHIEEQFGSGRLLACISSRPG	S	176
829	LOC Os02g28980.1	KRKETKLYGNMISKLSKLEDSETEG	S	602
834	LOC Os02g30140.1	LSSSLRSLSLSSSPRGRHHRGATTR	S	39
837	LOC Os02g30230.1	SKNAADDMIRILSGFDNRLSQITSD	S	50
839	LOC Os02g31150.1	MKYWSEYRNFSVSPPLKDSFSPVTS	S	410
840	LOC Os02g31220.1	SGGAEEGLYRLSSVGEEIDNLSNLS	S	75
843	LOC Os02g32350.1	ERLQIWQYGDVESDEEEQAPAARRT	S	970
844	LOC Os02g32469.1	AKCVCAPATHAGSFKCRLHRTNSQG	S	66
845	LOC Os02g32490.1	AGASSDKLRHVESMSELPSGAGRIS	S	22
846	LOC Os02g32550.1	VARSRSVGCGSRSFSGDFLERISNG	S	278
847	LOC Os02g32550.1	RSRSVGCGSRSFSGDFLERISNGFG	S	280
848	LOC Os02g33320.1	QKNHPASHDQGHSPDVAPEQKSSDG	S	119
851	LOC Os02g33320.1	QQDIKDSRLSGESPVIPLRLKIQRN	S	496
852	LOC Os02g33320.1	PEGLRSMAVSNVSPLLVSGSPQHNL	S	436
856	LOC Os02g33360.1	VKVVADVPGTGSSMDGEVKQAPDGS	S	91
857	LOC Os02g33610.1	EDSETKSSGKFGSFSAPRHSSRSSG	S	670
860	LOC Os02g33770.1	AVIDMNKMLSEGSGGPTAEKSPSTP	S	321
863	LOC Os02g34500.1	SHKDSLMQETDLSPSTASHKDSLLE	S	495
864	LOC Os02g34560.1	INVERQRSFDDRSLSDVSYSGGGHG	S	48
869	LOC Os02g34560.1	MELAVGAGGMRRSASHTSLSESDDF	S	13
873	LOC Os02g34570.1	TAGGSDETQEAISPKSLKDAVKPKH	S	373
874	LOC Os02g34590.1	DRVMEGQGAVTNSGDEMGMRAYGDE	S	60
875	LOC Os02g34680.1	HQWPRNVLYEDDSEETEEEGDNVGD	S	312
876	LOC Os02g34840.1	KRRAAGDDGPSESADDDIVVAQISK	S	30
877	LOC Os02g34840.1	AAKRRAAGDDGPSESADDDIVVAQI	S	28
878	LOC Os02g35150.1	GNGERSDTLEVTSDSDTRDMSSDAW	S	639
879	LOC Os02g35150.1	HDTSDDRWVATNSDVDSDLETQYQR	S	574
883	LOC Os02g35190.1	GQSQHRAPEREGSHNYDIESTDGSG	S	15
884	LOC Os02g35690.1	GGGGGGGREDAWSDGATSTLIDAWG	S	65
886	LOC Os02g35910.1	LRCWPGGLCLSRSIGDQDVGQFIVP	S	222
887	LOC Os02g36974.1	LRDNLTLWTSDISEDAAEEIKEAPK	S	243
889	LOC Os02g37030.1	QQKHCSGNGGGRSFSSGSSAWSSSR	S	143
891	LOC Os02g37130.1	SSKENNGVMWISSPDRLEDPRKPFQ	S	134
893	LOC Os02g37870.1	PADDEEAEAEAASEGEEDAAAAAAA	S	49
894	LOC Os02g37880.1	KEALLVMEFCEKSLVSAMESRGTGY	S	113
895	LOC Os02g37920.1	DPGKEMDPAIDGSDEDMCLTEKENV	S	366
897	LOC Os02g38050.1	TSLGSQPLDTQNSKSSGARGLKTLS	S	237
899	LOC Os02g38050.1	GLKTLSVEAPATSGVKSSQGQQASL	S	256
900	LOC Os02g38220.1	SGSGKVTAHLSNSPDESQITESMEQ	S	463

905	LOC_Os02g38480.1	APDDLGGSRSARSLDESIAVIDFPD	S	94
906	LOC_Os02g38840.1	SPSSGRASFSSLSGLKDLELSSESG	S	21
907	LOC_Os02g38920.1	VVDLTVRLEKPASYDQIKAAIKEEA	S	256
909	LOC_Os02g38920.1	EEDLVSTDFQGDSRSSIFDAKAGIA	S	292
910	LOC_Os02g38920.1	GGRAASFNIIPSSTGAAKAVGKVLP	S	212
917	LOC_Os02g38980.1	PVEEDVDICGNASPILIEKDAHNNP	S	439
918	LOC_Os02g39010.1	NEQDRRFKKHMKSPDPLEEQRMKEQ	S	671
920	LOC_Os02g39520.1	EGKEVILVDDNDSEQEDGGSGKVDE	S	65
925	LOC_Os02g39750.1	VRRWSSAARPEASSRASSCAATAST	S	136
926	LOC_Os02g39820.1	HCEEVKEMTRFASDVGIRGEMLREL	S	178
927	LOC_Os02g39840.1	QFNNQDKLSSQFSAKAQVGPAPALV	S	176
928	LOC_Os02g39890.1	SEKNPRFREKGDSDEEDDDYDKRRR	S	230
931	LOC_Os02g39920.1	YDNIIATICEDSSDVKEDMDADPSG	S	253
932	LOC_Os02g39920.1	EGEAAAEVEQGGSAGGASTGGKKRR	S	740
935	LOC_Os02g40880.1	WGKKLIVQKRRASLNDFDRFKVMLA	S	101
937	LOC_Os02g41550.1	TFDIPKVVLRETSPCALPIDQRVPH	S	526
938	LOC_Os02g41990.1	DSAVARSNDVEISPSEGLRNRKQSN	S	218
939	LOC_Os02g42040.1	AASVEKPENEKLSDSESQKVQPHDT	S	824
940	LOC_Os02g42040.1	EKPENEKLSDSESQKVQPHDTAFSA	S	828
941	LOC_Os02g42040.1	VLEGSKDPRAVESESGEETVDEEGG	S	972
942	LOC_Os02g42040.1	EGSKDPRAVESESGEETVDEEGGSA	S	974
943	LOC_Os02g42540.1	PSQPAENDKPKQSPEDSVNKVSSPQ	S	152
946	LOC_Os02g42580.1	APLPAASLVRSASLSEILDVKTETG	S	337
947	LOC_Os02g43020.1	QRPEASESSQPSSSPPSQPQEQPEA	S	208
948	LOC_Os02g43020.1	RPEASESSQPSSSPPSQPQEQPEAK	S	209
949	LOC_Os02g43020.1	ASESSQPSSSPPSQPQEQPEAKARE	S	212
954	LOC_Os02g43370.1	PEIERCDAGDVESDHDGAAAAAERV	S	18
955	LOC_Os02g43740.1	ELVAEPTSARSMSFVGTHEYLAPEI	S	528
961	LOC_Os02g44690.1	SGLLRGAFFGGSSEGEEEDEKTRKK	S	143
962	LOC_Os02g44740.1	KTDSDVTSLAPSSPPRSPRRSAYYV	S	18
963	LOC_Os02g44740.1	DVTSLAPSSPPRSPRRSAYYVLSPA	S	22
966	LOC_Os02g44770.1	GLMTGKSGQIAKSGPLEEEEEDPFM	S	334
967	LOC_Os02g44810.1	SSSDPDQEPTSPSAAGADGAQPRAP	S	32
969	LOC_Os02g44810.1	PPHPKRRKMSSSSDPDQEPTSPSAA	S	22
970	LOC_Os02g45070.1	RARFYMEPDTSDSGSMASGAHTRGG	S	1036
971	LOC_Os02g45070.1	RFYMEPDTSDSGSMASGAHTRGGGP	S	1038
981	LOC_Os02g46090.1	LRSKYFASFRGASQRHDEAGYAPVA	S	21
983	LOC_Os02g46650.1	ADITNLDRPSTPSPKRKRFFSDDNE	S	562
987	LOC_Os02g46962.1	SEVSKKMTAGMSSMSFKMKEIFQGQ	S	33
991	LOC_Os02g47140.1	SSLAPKIGPLGLSPKKIGEDIAKET	S	38
992	LOC_Os02g47150.1	KKQPAKKASTQLSDDEDDEVLALKD	S	1292
993	LOC_Os02g47150.1	PAAQKKQPAKKASTQLSDDEDDEVL	S	1288
994	LOC_Os02g47170.1	SKEKITTAEKIRSDGSDFDESIEKC	S	239

1007	LOC Os02g48010.1	NLEVKKDDGQGDSVTVGGTRKRRFA	S	963
1008	LOC Os02g48010.1	TVEATADDTEGTSKAEEPATGSKGA	S	1059
1010	LOC Os02g48010.1	SLLQKCSRIFKFSPRKKAEQSSEQQ	S	786
1012	LOC Os02g48010.1	SEQDEDSEAHSESVSLGGQRRKRRQ	S	992
1013	LOC Os02g48010.1	KGRAKGGVKRTRSVLAVVEDAKEIL	S	936
1018	LOC Os02g48380.1	QPPMNLLALPDFSKASILARTAKAR	S	48
1021	LOC Os02g48640.1	YPEFAAGLVRQDSFGGGELPRQDSF	S	465
1022	LOC Os02g48640.1	DSFAIGELRRQGSYGVELVRQDSFG	S	487
1024	LOC Os02g48730.1	EAMSSAVDAISCSKGIAAPPTEEAT	S	134
1025	LOC Os02g48740.1	KVKASGRTSQMESFKDKSLSNGRFF	S	549
1031	LOC Os02g49070.1	AVKSVTSSISSASGKHVADDTREFV	S	164
1035	LOC Os02g49070.1	FLKPSLRITSKGSFDATNAVKSVTS	S	146
1037	LOC Os02g49080.1	SEGSPAYSWRGGSQGSSSGLIVKKT	S	126
1038	LOC Os02g49080.1	SPAYSWRGGSQGSSSGLIVKKTMKV	S	129
1039	LOC Os02g49090.1	MPTSFTDSYSVGSPSKSSTVDQQSE	S	618
1044	LOC Os02g49450.1	GLDDDFDKIDGTSGLESDDDDDKEK	S	281
1045	LOC Os02g49620.1	KKQMKLKSSQTDSDDMLELLTSKKD	S	80
1046	LOC Os02g49992.1	ANVSPIKGAEFSSPGQLGLTARGDQ	S	229
1049	LOC Os02g50550.1	GLNTPEGGSMRQSHSDGSLDTMARK	S	724
1054	LOC Os02g50570.1	GGSTSAGLGRSNSPEQIFLLEPRKS	S	475
1056	LOC Os02g50620.1	VLEMDTYRYHGHSMSDPGSTYRTRD	S	293
1058	LOC Os02g50620.1	AVGMEAAITRSDSIITAYRDHCTYL	S	112
1059	LOC Os02g50700.1	TMHETKEAVVGESDDEKEKFKQRVE	S	142
1060	LOC Os02g50790.1	RLIPSPQTRDDASQGETTDANPPLK	S	748
1061	LOC Os02g50799.1	PLVAADPCDLAGSPMKEMAFSDPAN	S	377
1066	LOC Os02g50840.1	VRFPNPGRSPARSASGGSFEDPSFT	S	683
1069	LOC Os02g50840.1	FPNPGRSPARSASGGSFEDPSFTGS	S	685
1071	LOC Os02g50840.1	EISKSELISSSPSPRNSSANEQGCE	S	844
1079	LOC Os02g50880.1	SRRHEAPADADGSRPPSPPRRGEAK	S	63
1085	LOC Os02g50970.1	ERRQSVAAERRRSQEEEWERRRSQE	S	102
1092	LOC Os02g52250.1	SKILALTVDAKGSVAFDAVVKQGEN	S	110
1095	LOC Os02g52250.1	RASGSPPVPVMHSPPRPVTVKDQQD	S	247
1096	LOC Os02g52250.1	KFKHKRVPRASGSPPVPVMHSPPRP	S	239
1097	LOC Os02g52280.1	DSKELVMESDIRSRMQALKDQLVEA	S	925
1100	LOC Os02g52780.1	AAVGAPPVQRQGSLTLPRTLSQKTV	S	98
1101	LOC Os02g52810.1	PSKWEDAEKWILSPVSCDGIGRMSA	S	246
1110	LOC Os02g54010.1	SDAVGMMDEKVDSDGEDIIDHHVDE	S	493
1116	LOC Os02g54500.1	DFENASTDSEKLSPRFLSYFQLRSP	S	937
1117	LOC Os02g54510.1	NTGTDQDSDVHGSGEQQKSSGIEAT	S	779
1119	LOC Os02g54510.1	FDERIPDDFVRHSPKYRHYEVHSPQ	S	252
1121	LOC Os02g54510.1	GEMDSPWSPAYISPGHYGVHDPRDF	S	215
1123	LOC Os02g54770.1	ECRLRIGPGGLGSGKRRSRSRSRSR	S	112
1125	LOC Os02g55060.1	ALGKMSKDDADVSGDLSGLSDKELG	S	69

1127	LOC Os02g55940.1	DQKTKDDIKKTRSMQGLSFLANIGG	S	290
1131	LOC Os02g56270.1	PTQDERPMERKPSNQQIASPPPNYE	S	295
1134	LOC Os02g56480.1	AKNDDRHTTNPVSDHVVVSPVVSPG	S	280
1136	LOC Os02g56480.1	HTTNPVSDHVVVSPVVSPGEFQRQI	S	286
1138	LOC Os02g56480.1	LLRPAPGSGGGSSRGGSTPRLRVFL	S	142
1140	LOC Os02g56530.1	LQPLDEFATPPSSPTQFQDAKGKES	S	535
1143	LOC Os02g56740.1	PTVLDQDDDSDVSAVEDDDYSKFEK	S	557
1147	LOC Os02g57080.1	RIEGARDPRFALSTGGHAQSPALQN	S	697
1150	LOC Os02g57390.1	DSSRAQLQSRSESMNGLRSGSTSPS	S	254
1151	LOC Os02g57390.1	TTPDPQLIRRTPSPCLPPVGVRMGS	S	305
1153	LOC Os02g57390.1	SHNFASAVGSSISRSTTPDPQLIRR	S	290
1155	LOC Os02g57500.1	APAAGGERAPSPSPPPPPRLPALKS	S	30
1158	LOC Os02g57600.1	EHKLKDDLHWIPSFDKGKNLSLVDM	S	120
1159	LOC Os02g57600.1	EHQKELSGQDAVSAEGDDEKFQDTC	S	95
1165	LOC Os02g57660.1	PHSKNVSLERRWSLEVAIEKFIGHD	S	322
1166	LOC Os02g57660.1	GHDATGSSGLERSESINDSDLPMLE	S	344
1167	LOC Os02g57660.1	DATGSSGLERSESINDSDLPMLERE	S	346
1169	LOC Os02g57820.1	RPPGSGKMQQLASLGKWFLGSVGTG	S	197
1171	LOC Os02g57980.1	AKKDMRFARFGASSEEESGYDELEA	S	260
1174	LOC Os02g57980.1	AGRFGRKLRPDSSDEDDEDISTAKK	S	238
1177	LOC Os02g58110.1	TSLRGIVPAGEQSGDESKRKGGRRA	S	525
1179	LOC Os02g58110.1	TQGERKILDKLRSYQGTPFIEGLMG	S	52
1180	LOC Os02g58210.1	AAEEVEAAAIDGSGEEAKRKSGKQR	S	14
1182	LOC Os02g58220.1	SPAKTYSAMGSSSSNGFSEMTTPTS	S	184
1185	LOC Os02g58340.1	LEVFVSFRGREPSPEALLRHNGLLP	S	754
1187	LOC Os02g58410.1	GLQLTKDASSLGSSSPIQEQNQKAY	S	1900
1188	LOC Os02g58410.1	QLTKDASSLGSSSPIQEQNQKAYYV	S	1902
1189	LOC Os02g58410.1	SAVTGRSVSGPLSPLAPEVSIPNPT	S	1443
1190	LOC Os02g58410.1	SRRNDAPLSRSDSLSIINGMRYLKL	S	164
1195	LOC Os02g58490.1	QQQGRGCRARGASPPPPPQQQQQQQ	S	54
1197	LOC Os02g58570.1	MAQAQWGVDGYGSMKGLIRLRTSPH	S	150
1199	LOC Os03g01470.1	LAGRRRGPHGDGSAEPTARAAAASA	S	109
1200	LOC Os03g01470.1	GSAEPTARAAAASARRRLVVAVRRQ	S	120
1201	LOC Os03g01490.1	KECKNPQLVNLGSGRFCITRFFHSR	S	338
1204	LOC Os03g01710.1	SSNGGMHVGRQDSASSYVSASRGDD	S	246
1205	LOC Os03g01710.1	DRSPTNDDIDSKSDGSDSVANRSVR	S	174
1206	LOC Os03g01710.1	PTNDDIDSKSDGSDSVANRSVRSSS	S	177
1207	LOC Os03g01710.1	NDDIDSKSDGSDSVANRSVRSSSGN	S	179
1208	LOC Os03g01710.1	RIAMQAKLVRLESDLSAVEASHVHE	S	1196
1209	LOC Os03g01970.1	SDNAAIDEGRKQSPEEVSGPESGQC	S	605
1210	LOC Os03g01970.1	RKQSPEEVSGPESGQCEAEADGEDN	S	614
1211	LOC Os03g01970.1	IDEGRKQSPEEVSGPESGQCEAEAD	S	610
1212	LOC Os03g02010.1	YDTAGNCDRTPHSDGSGDEDFFQEM	S	167

1213	LOC Os03g02010.1	AGNCDRTPHSDGSGDEDFFQEMSEK	S	170
1214	LOC Os03g02150.1	PASNHLTLVKMPSQNITEESNVAYE	S	657
1215	LOC Os03g02160.1	SSPDNHDIKCEYSSPTPIPESKELS	S	359
1218	LOC Os03g02310.1	MKAGRKNLRRACSEGAVTLGEGESI	S	13
1219	LOC Os03g02320.1	LALPTRASANSLSAPIRSSGGYVGS	S	540
1220	LOC Os03g02480.1	SDRSSSVLSQRFSDLENRAKSRRES	S	422
1221	LOC Os03g03034.1	SDDPAKKIRLSTSFNVRKETVHNWR	S	111
1223	LOC Os03g03430.1	KINHRDAHADGLSPSRVSIAGSRRS	S	348
1225	LOC Os03g03460.1	QKTDELSAVESGSNYSSRRDDDTAM	S	229
1226	LOC Os03g03460.1	SQQKTDELSAVESGSNYSSRRDDDT	S	227
1227	LOC Os03g03460.1	FASPEDSKYKQKSPAESPMEITSET	S	266
1230	LOC Os03g03560.1	VEGANTQEVECNSPEMATERFRSDS	S	623
1232	LOC Os03g03830.1	DLGYDFGEESVRSPSSAGRSASGSP	S	852
1234	LOC Os03g03830.1	SAGRSASGSPFVSSNFAMHDSSPSK	S	867
1235	LOC Os03g03830.1	DDSSSYNLGRFDSFRSQESGFFPQE	S	998
1236	LOC Os03g03830.1	SPKSSTNFGRFDSFDDADPFGSSGP	S	1044
1237	LOC Os03g03830.1	NNSKGENVTGFDSPKSSTNFGRFDS	S	1032
1239	LOC Os03g03870.1	SNQKSGLTSNEDSGEEHLARTEPVA	S	298
1243	LOC Os03g04710.1	ETIQSFTQRGGASTDEEEEEDDDDG	S	117
1245	LOC Os03g04920.1	SSVPIKRLTPSVSNIDNLKNKVSNN	S	878
1247	LOC Os03g05200.1	MESLHSSVKGVGSDEEEELNMKQEI	S	607
1251	LOC Os03g05390.1	QLAIKSLRASSMSHEMVEVSTVTDR	S	296
1252	LOC Os03g05390.1	QQRSVIIEDSPPSPASNGDKEKEDE	S	336
1253	LOC Os03g05390.1	SVIIEDSPPSPASNGDKEKEDEVAE	S	339
1257	LOC Os03g05390.1	EVTSHRFTPARMSHVSSLNPDDMDC	S	240
1260	LOC Os03g05390.1	MDCISEPIIRSNSVRSTSANENLRS	S	262
1261	LOC Os03g05390.1	ISEPIIRSNSVRSTSANENLRSRSV	S	265
1266	LOC Os03g06120.1	SEKLQVELQRSQSNMDMGQCEVIQH	S	208
1267	LOC Os03g06120.1	RMEYIERDQCEYSFDDEDKEVQDAL	S	150
1269	LOC Os03g06220.1	ADGGKPPTPEKKSWADVEEEEEAKA	S	14
1273	LOC Os03g06950.1	TKDKSDTSDSSTSPHNEECESFGAV	S	482
1274	LOC Os03g06950.1	KILEEELADNVGSDRDKDSMSSDIW	S	456
1277	LOC Os03g07580.1	SPNSSAKGLRLRSLSPLHGSSLKVS	S	247
1282	LOC Os03g08220.1	SEEVPRLAASPSSGIKGGGAGERRP	S	61
1284	LOC Os03g08360.1	VSKAEFIDLARKSGKFDEDSLAFQS	S	150
1286	LOC Os03g08360.1	NRGVEPSGPDAGSPTFSVRVRRRLP	S	26
1292	LOC Os03g08820.1	IFERIDIGGSPVSAARDEKVNNDSD	S	392
1293	LOC Os03g08830.1	YTDARHGRLRSLSDLLFRSDASGSS	S	524
1299	LOC Os03g10180.1	LGQEFYKDIFAGSESMSPRRGGAAG	S	80
1300	LOC Os03g10180.1	FYKDIFAGSESMSPRRGGAAGDLDV	S	84
1301	LOC Os03g10180.1	QEFYKDIFAGSESMSPRRGGAAGDL	S	82
1302	LOC Os03g10460.1	AAPKGEIHYFRDSDDEQPDSDEDPD	S	367
1303	LOC Os03g10510.1	VFDVLPSTKLVTSVKLPDYNSGKVE	S	96

1304	LOC Os03g10590.1	KWKHDLYEQTNRSPTPKTEEEQIAK	S	344
1305	LOC Os03g10590.1	RFPESRDARRPRSFFQHDERGSAGQ	S	138
1310	LOC Os03g11050.1	QKKDRANLRRVSSFEALHSATKALP	S	217
1311	LOC Os03g11140.1	RRDLSADETDFCSDDETLIEKLENN	S	546
1315	LOC Os03g11220.1	HRGRPPPQQHRPSSGGGGGGGSGDL	S	23
1316	LOC Os03g11220.1	RGRPPPQQHRPSSGGGGGGGSGDLA	S	24
1317	LOC Os03g11240.1	NGQNRSRFARTRSSPELTDPSVEGY	S	505
1321	LOC Os03g11380.1	LGTPVYRTNPFDSDSDSEVPSRPSR	S	20
1322	LOC Os03g11380.1	TPVYRTNPFDSDSDSEVPSRPSRAQ	S	22
1323	LOC Os03g11500.1	SRTAVLTASPSSSLASRRFGSRWGR	S	126
1325	LOC Os03g11550.1	LEVKRKRIERRNSLTSNISKEAVGQ	S	183
1326	LOC Os03g11550.1	PSAPPSGLFRTSSLPTVAAAEAAKK	S	83
1329	LOC Os03g11580.1	NSPLAKRANLLESKSPDGVGQTFRA	S	415
1330	LOC Os03g11580.1	KRSRGQSPQGRRSCSPLSQSLSSGR	S	388
1332	LOC Os03g11580.1	NLSPEPLGAKDWSSDMPGDIDNDES	S	873
1336	LOC Os03g11580.1	SDELGHPVKEDLSGNDSDRGQQTRR	S	357
1338	LOC Os03g11790.1	MKNLQVIFPNSISFGRAELPFSMPN	S	315
1342	LOC Os03g11970.1	LDNEAIYDICKRSLDIERPTYTNLN	S	216
1344	LOC Os03g12010.1	DQAGQAASSPLNSPTRGIVVDISDG	S	442
1345	LOC Os03g12120.1	KEVGPPPPPCVDSDHEDLPLAERRR	S	17
1346	LOC Os03g12300.1	RVHLKNQLQSQKSASAIVLSQEIQR	S	675
1347	LOC Os03g12300.1	HLKNQLQSQKSASAIVLSQEIQRYE	S	677
1349	LOC Os03g12360.1	TAGLIDDSPLSPSTPSPSPRPRTIV	S	51
1351	LOC Os03g12450.1	PHLLHRRDKEEASPSPPPAPAHTPA	S	21
1354	LOC Os03g12450.1	SHQRSSIHLSPQSKNQTTGALWNLI	S	732
1358	LOC Os03g12910.1	PLEKFHSDVAGRSFHNGRFIQRMRQ	S	245
1360	LOC Os03g13560.1	TSTMGEMANGDVSPKSSPAKQSDAV	S	600
1361	LOC Os03g13560.1	MGEMANGDVSPKSSPAKQSDAVEQK	S	603
1362	LOC Os03g13560.1	GEMANGDVSPKSSPAKQSDAVEQKP	S	604
1367	LOC Os03g13720.1	LLAMEDSNKGIDSPSREDSSSVSHR	S	707
1369	LOC Os03g13790.1	CPRGDVCLSSSSSAVNRTPGNESFS	S	140
1374	LOC Os03g14260.1	SPAAAPPPTPPESDPRLVEAFVPFL	S	15
1379	LOC Os03g14710.1	IDGVRRSSRDWASGEFVPKSGGAVS	S	517
1384	LOC Os03g15040.1	GEPNQDNEPRDSSIELPRVITDMET	S	22
1391	LOC Os03g15580.1	MRNRRGVVLFAESDDDLADAHAAKD	S	777
1393	LOC Os03g15650.1	EQKSQTLKASRDSDVNLSLKDNFQG	S	304
1397	LOC Os03g15810.1	LPAAAGADQPRRSPQETTAAPSKPA	S	53
1399	LOC Os03g15940.1	PASEKLTPELTRSPSKAARMFSGTQ	S	93
1401	LOC Os03g16369.1	PSPHRRSHIRRKSPPFVRQRSPSPH	S	293
1403	LOC Os03g16369.1	KKYPAKIDDDSESEDGSPFRKDKRK	S	676
1405	LOC Os03g16369.1	DSEHRKLTKSLNSPNKPERNSTRDS	S	513
1408	LOC Os03g16369.1	AHDDLSPGEKSPSQHSGKETRRKQN	S	589
1409	LOC Os03g16369.1	DLSPGEKSPSQHSGKETRRKQNNQL	S	592

1410	LOC_Os03g16369.1	SPRRRSSLDRHWSPSPGRRRPRSPS	S	336
1412	LOC_Os03g16369.1	GMHNSYSKDGMNSEDAIKGLRDGMA	S	650
1413	LOC_Os03g16369.1	DSSLKGTDKHLPSQVRTDSSGEEEG	S	536
1415	LOC_Os03g16369.1	DKHLPSQVRTDSSGEEEGSRARENA	S	543
1416	LOC_Os03g16369.1	LKLKHAETVDMASDLEKDRESDSDA	S	789
1417	LOC_Os03g16400.1	LDIFKGTKGGDLSEDEGVVKDCHPF	S	312
1419	LOC_Os03g16800.1	EEFMRDRAKRPKSPPREPEPEPVKE	S	363
1420	LOC_Os03g17020.1	DDDFRPDMRRNQSVGSFGESGAESG	S	173
1421	LOC_Os03g17020.1	FRPDMRRNQSVGSFGESGAESGRQP	S	176
1423	LOC_Os03g17020.1	GAESGRQPPRSKSTQDMYTRQQLEA	S	193
1427	LOC_Os03g17180.1	NSHDSPKDQSPPSEQTIDNGIPLVA	S	725
1428	LOC_Os03g17180.1	IQRQSSKNSEPDSPISPLLTSDPKN	S	777
1432	LOC_Os03g17310.1	QEEGKRRDGSDASGSEPAAAAFPAW	S	18
1434	LOC_Os03g17490.1	KSRAERFGLASSSADEDAKKKARLE	S	113
1435	LOC_Os03g17540.1	WKDKKGLATKLYSFKHDPLCPQVNS	S	395
1437	LOC_Os03g17950.1	TPSEPIVPIRRSSFSYTPSRSLGQS	S	171
1440	LOC_Os03g18070.1	WALRVSAPTRLTSVVEEDNRGEEVV	S	64
1442	LOC_Os03g18310.1	EQRAIFVDRGVGSPEFARPTNETIS	S	194
1443	LOC_Os03g18454.1	TDCYKGERGHQLSLKYGRQTDALQP	S	165
1444	LOC_Os03g19040.1	KPPGTTTTTTTRSPPPPLPVTKVGG	S	53
1447	LOC_Os03g19340.1	ECSTQDREGLALSPTNREAVETKPN	S	946
1449	LOC_Os03g19520.1	EEENKHVWARKNSAYFSKLVFSFTD	S	587
1450	LOC_Os03g19530.1	AKAVDAGMMEYDSDDNPIVVDKKKI	S	151
1452	LOC_Os03g19530.1	ALAADAMHAGYDSDEEVYAAAKAVD	S	131
1455	LOC_Os03g19590.1	HAVFEQSGASGRSFDYTQSLRASPT	S	84
1459	LOC_Os03g19870.1	KGKSEELADSIGSLSIGPERTNFKK	S	39
1461	LOC_Os03g19990.1	TADPMSLPLFLHSVRPYGDIFTVDI	S	640
1462	LOC_Os03g20340.1	SVGVVGGGGGAGSPPGSPGRSLSPR	S	59
1464	LOC_Os03g20530.1	VASEKLASENSVSFSRKQENSDSFS	S	299
1465	LOC_Os03g20600.1	AVGQLGCIRKSWSNDSLSSYGGGGR	S	40
1471	LOC_Os03g21140.1	HPLQGFDGAECRSPGPGEGGGMFPY	S	192
1473	LOC_Os03g21160.1	HQLGARMLQHSNSANELMLRRKLEE	S	479
1474	LOC_Os03g21160.1	DFHQLGARMLQHSNSANELMLRRKL	S	477
1477	LOC_Os03g21160.1	HALQAFDGAECRSPGPGESGGMLPY	S	190
1480	LOC_Os03g21160.1	YRKQQQGDFCCMSPTGLDARDPFDF	S	454
1486	LOC_Os03g21530.1	AKFDAEYDGSDLSGEEVDNDTKKSK	S	726
1487	LOC_Os03g21540.1	SSAKSHTTGSHASSLPSNLCRHFSF	S	518
1488	LOC_Os03g21800.1	SSPPRPKHRHSSSVDGSGFFAAARK	S	92
1489	LOC_Os03g21940.1	KSKAEKAREKTLSDQFEAKRAKSKA	S	156
1491	LOC_Os03g22340.1	TVSRDKTKVTVTSDGPFSKRYLKYL	S	77
1494	LOC_Os03g22350.1	SDYVTKAGYGSESEVDDEAATVSLA	S	265
1495	LOC_Os03g22350.1	NQVPDLRDLNDVSDYVTKAGYGSES	S	253
1496	LOC_Os03g22580.1	SRRNKNSREAKGSPVESVSSSPLKN	S	447

1501	LOC_Os03g22740.1	EKKEKKKKKKSDSQDAEDVAMETEE	S	540
1506	LOC_Os03g23970.1	EELRGGSAYGADSLCIGVARLGSDD	S	218
1507	LOC_Os03g24050.1	TSSLFARRAGDPSAAIAGFQRRLYG	S	34
1508	LOC_Os03g24180.1	DLYDYKVVHADDSDAERSEDAKAAE	S	464
1511	LOC_Os03g24339.1	TPAFKEGSQKQLSPLNRIIRSTSTN	S	857
1512	LOC_Os03g24339.1	ELNHRNLKDKSTSPDSMPMKELIAV	S	804
1515	LOC_Os03g24890.1	RSASPDELERHVSPSNNGHGPPVDG	S	229
1516	LOC_Os03g24890.1	ASPDELERHVSPSNNGHGPPVDGKS	S	231
1518	LOC_Os03g24890.1	YRDHRDDYSPGESLSPHGQDKRHHR	S	201
1520	LOC_Os03g24890.1	RSVSRSPRSRYHSYSPSPSPARRDY	S	177
1523	LOC_Os03g24890.1	GQDKRHHRSNGRSASPDELERHVSP	S	218
1524	LOC_Os03g24890.1	DKRHHRSNGRSASPDELERHVSPSN	S	220
1526	LOC_Os03g24930.1	FRKLSSMTNSPASSVAGAAEGGKDD	S	64
1531	LOC_Os03g25450.1	SAYADGEVKAELSDGEKGGSEKKKK	S	545
1532	LOC_Os03g25464.1	HVSTSQADDCILSSMSRISIPAQTK	S	30
1533	LOC_Os03g25600.1	AAAMAGLQRPSKSFSETTFRTISGA	S	113
1535	LOC_Os03g25620.1	QFDLLALEEYGDSDDDDDDPGVRNG	S	263
1537	LOC_Os03g25720.1	AGASSAASADGASPRGRGGGGGGLA	S	64
1538	LOC_Os03g25890.1	AISNVIIGGIEDSDDVKTYLENIDR	S	18
1539	LOC_Os03g25970.1	GGYGAYGGYSGRSGSSGLSGPSSFS	S	523
1543	LOC_Os03g26460.1	DLEDDDEDDTADSANKDDEDIKAEG	S	236
1544	LOC_Os03g26630.1	EKLNLDRSSGDESMEEDVMEIKQVE	S	380
1547	LOC_Os03g26630.1	GGSPEKLNLDRSSGDESMEEDVMEI	S	376
1549	LOC_Os03g26630.1	NDQVPEVSPDLGSPIKCESISSDDI	S	283
1551	LOC_Os03g26630.1	TAELSSDHVKEVSLPDTVVDNSSVD	S	412
1552	LOC_Os03g27030.1	RYQEKGYRRRSRSISPDYDRRHRKN	S	178
1553	LOC_Os03g27030.1	QEKGYRRRSRSISPDYDRRHRKNGR	S	180
1555	LOC_Os03g27080.1	AAARIFQAFRVESFHRKKVVEYGDD	S	330
1556	LOC_Os03g27460.1	EVRGININEEAQSTDGATRGHVHAR	S	351
1558	LOC_Os03g27460.1	RQPQNGSARGGSSRGRVKKTTARKK	S	288
1560	LOC_Os03g27800.1	HVANPWTAPPIRSPEQKGSYKEDDA	S	721
1562	LOC_Os03g27840.1	TSDLKVAGISPPSSPIRVGIPNRSS	S	363
1565	LOC_Os03g27840.1	EYITEFGGSDDTSDLKVAGISPPSS	S	352
1567	LOC_Os03g27840.1	YRDPYREQRRSPSYDSYSRGRRSRS	S	295
1571	LOC_Os03g27990.1	EKNEPLTLRPIASGKFNQLRTISII	S	371
1572	LOC_Os03g28330.1	FNASFPRPSLSKSIGNGVQFLNRHL	S	157
1576	LOC_Os03g29750.1	SCVMLPKSDSFVSVSEYQHPLTEDM	S	420
1577	LOC_Os03g30460.1	VNDEYMKTLETLSRKIKFIEVDAMV	S	189
1579	LOC_Os03g30790.1	RTPPPPSPSPSLSLSRSQQQTPRGR	S	25
1581	LOC_Os03g30790.1	PPSPSPSLSLSRSQQQTPRGRQPPP	S	29
1584	LOC_Os03g30790.1	PSLHRTPPPPSPSPSLSLSRSQQQT	S	21
1586	LOC_Os03g31730.1	SSCKDVHVTTDVSPGISPKGDIPSD	S	601
1588	LOC_Os03g31750.1	AAGILTARGGMTSHAAVVARGWGKC	S	468

1594	LOC_Os03g35340.1	ESNIPSERKFSVSSPEKVMFPDHAD	S	1719
1606	LOC_Os03g40010.1	DGNKDEAEEEEVSEADKKVEENKEE	S	250
1607	LOC_Os03g40010.1	EKQGEEEAEEKGSADNDNEEGDKNA	S	212
1610	LOC_Os03g40010.1	NKGGSENGTKGPSDGDVEMAEAETA	S	66
1611	LOC_Os03g40010.1	NDNEEGDKNANISDEHLKKDGGKDG	S	227
1614	LOC_Os03g40110.1	DDYSDEEGHMILSKNRIPLQSGDVP	S	189
1617	LOC_Os03g40110.1	ERQKRMLGTAESSDEDDDDDEEGDR	S	329
1618	LOC_Os03g42220.1	KVKIYGARVRVDSMAKVAEIEAAEK	S	253
1621	LOC_Os03g43180.1	PTVAMEGGGDGYSGGAARLERRRRR	S	93
1622	LOC_Os03g43390.1	GGGEGTKLRRALSFEDAATASASAT	S	73
1627	LOC_Os03g43800.1	QVDADFPEGSLGSREAETGDYPKYA	S	382
1628	LOC_Os03g43800.1	SAQIKDENKDNDSDEKPSMPAAVSV	S	522
1629	LOC_Os03g43800.1	QTPGRSGDRMFLSPVKSTDRKNADS	S	347
1634	LOC_Os03g44310.1	PPPRHADHLAPGSPSPAASPSELGD	S	98
1635	LOC_Os03g44310.1	ASPSELGDDESWSRAPSAAELESNN	S	115
1637	LOC_Os03g44310.1	ELGDDESWSRAPSAAELESNNNDLA	S	119
1638	LOC_Os03g44310.1	SWSRAPSAAELESNNNDLAEIRNDN	S	125
1640	LOC_Os03g44484.1	PNRENTMGMRHGSYDKLDDDGLAPP	S	193
1642	LOC_Os03g44500.1	ATAARKLLDRQMSINSVPKKVIASL	S	643
1645	LOC_Os03g44830.1	QDPRFPGRARTLSSAGTEPTAREAS	S	305
1651	LOC_Os03g45300.1	EVSPVAAGSGEDSGRGRRTRAVTRV	S	141
1653	LOC_Os03g46040.1	PQAPLVPILRTPSGEVVYGITDDPV	S	171
1655	LOC_Os03g46340.1	DNSPPLTKHVDASDDDVDKACVSDS	S	413
1656	LOC_Os03g46340.1	EERSESCSGTDNSPPLTKHVDASDD	S	403
1657	LOC_Os03g46340.1	MLKLRQSEEKVISLSKEKDQLLKER	S	293
1658	LOC_Os03g46480.1	GDADEDAEPEQPSSPSVKKAEEKGI	S	241
1660	LOC_Os03g46480.1	DEDAEPEQPSSPSVKKAEEKGIVVV	S	244
1661	LOC_Os03g46590.1	DGEGPSAEVNETSVEEVNAREDRGV	S	134
1662	LOC_Os03g46600.1	DGAVKSTTGRLPSPSLLGRYPSLEE	S	48
1665	LOC_Os03g46710.1	GDGMQTKFSRRESLDPELPGASSSS	S	174
1667	LOC_Os03g46710.1	DLETEQSESKVPSPQGHRSTRTEDI	S	554
1668	LOC_Os03g46920.1	SSSLSSGYGRRKSAKEDESLSLDPV	S	706
1675	LOC_Os03g47930.1	VHSPVSTTNLSKSQSFDSPTEKELT	S	146
1677	LOC_Os03g47930.1	RILGEENNKGMESPDRDQIDRYITS	S	574
1679	LOC_Os03g48600.1	EITEYRQSQPRRSAELQGIAREAMS	S	263
1681	LOC_Os03g48600.1	PVQCRVTSLENMSPESEPQRTLLSR	S	186
1683	LOC_Os03g49170.1	NGEELGKLRRSSSFELRSGGDDPDL	S	678
1691	LOC_Os03g49640.1	RTDDERVDASHMSDTEEEEEFADEG	S	312
1692	LOC_Os03g49770.1	SSFKSSGSDFQRSEAGSDDEDDYPM	S	381
1694	LOC_Os03g49770.1	ENIYFDTRDFLSSSSFKSSGSDFQR	S	368
1695	LOC_Os03g49770.1	IYFDTRDFLSSSSFKSSGSDFQRSE	S	370
1696	LOC_Os03g50120.1	SFDDSFDREDDGSDPDEGERRRAKV	S	427
1701	LOC_Os03g50300.1	PKKYAEDVESGSSDVETKYKKMYED	S	566

1702	LOC_Os03g50300.1	SRGPKKYAEDVESGSSDVETKYKKM	S	563
1705	LOC_Os03g50330.1	LLNHKSAEDPDGSFYTGDDPDRSFY	S	49
1709	LOC_Os03g50390.1	PRKRVPADIKSFSHELNSKGVRPFP	S	277
1711	LOC_Os03g50390.1	DEEDDVVRSLRASPVHPVKDRTSID	S	839
1713	LOC_Os03g50390.1	SYTQKDLPNAVGSPDVAKVSNSSIN	S	637
1715	LOC_Os03g50730.1	ANGGGKKLEKEDSLDWERYMKENGN	S	170
1716	LOC_Os03g50810.1	YKRKQKPFGRVQSPHAMVVHPRHSG	S	538
1718	LOC_Os03g51000.1	RPPAKIPVPVPPSPSGKDDEILTEP	S	392
1719	LOC_Os03g51020.1	PDDPFVEDDQHGSVRSIGVGISSDA	S	550
1725	LOC_Os03g51020.1	VAEPKEDDEQDGSEAGSNAGPRGAA	S	56
1726	LOC_Os03g51020.1	KEDDEQDGSEAGSNAGPRGAASVRS	S	60
1727	LOC_Os03g51030.1	ADSCHRQLNKILSDLDQDSVMNKSS	S	951
1729	LOC_Os03g51140.1	AGTSGKAILEGGSDDEGASTEAHGR	S	399
1731	LOC_Os03g51250.1	AAPEKTSRAGSFSFKSGPPKLELQM	S	327
1735	LOC_Os03g51520.1	ESFVKQAEEDPMSDDENHEDSRRGR	S	1230
1737	LOC_Os03g51520.1	ISRAEQPTGHNSSDQDELRTRNSDN	S	767
1739	LOC_Os03g51600.1	ALEKDYEEVGAESDENEDGDDGDEY	S	439
1740	LOC_Os03g51790.1	KRSSPTASPPADSDDEGSPRGLSLL	S	54
1741	LOC_Os03g52310.1	FAYSSGSKIAGTSHLKRHITLGSCP	S	108
1742	LOC_Os03g52310.1	CCKLCKQTFAYSSGSKIAGTSHLKR	S	100
1745	LOC_Os03g52700.1	PGKGVSRWRSDESSEDEDDKRTSGY	S	885
1746	LOC_Os03g52700.1	GKGVSRWRSDESSEDEDDKRTSGYM	S	886
1748	LOC_Os03g52794.1	ETSAKSMLAEASSLAEKSRTSFELH	S	2695
1749	LOC_Os03g52970.1	EGVRSIVLKPSESLDEGRFTRIAGA	S	28
1752	LOC_Os03g53280.1	DLTLPKFGDDMSSIGSHGDLDDFSA	S	1524
1755	LOC_Os03g53280.1	NMNRTASYASGISPESSLDDLTLPK	S	1505
1756	LOC_Os03g53280.1	SYSDGRQLTASPSMSSTISGIDLDS	S	2111
1757	LOC_Os03g53280.1	TERKDLDAADFASFKDEDDIFKGGS	S	2668
1761	LOC_Os03g53630.1	ANGDELSMGLSRSASGRESIEQEDT	S	63
1762	LOC_Os03g53630.1	GDELSMGLSRSASGRESIEQEDTVM	S	65
1764	LOC_Os03g53630.1	LTDCTNQKKGIDSPVNETKGDSTPD	S	125
1765	LOC_Os03g53670.1	KRAKQQLLQGQGSLDNASHEKEKNA	S	610
1766	LOC_Os03g53670.1	GSASAGDAQNAASPVDRSITPLLQE	S	54
1767	LOC_Os03g53880.1	FLIQKNILKRPRSPDGDHGLAVGNF	S	91
1773	LOC_Os03g54780.1	QQQNSRRKPSVSSVPESVTREDPST	S	247
1774	LOC_Os03g54780.1	HPDMQEAKTMPPSTGTVKKLKVAEG	S	662
1777	LOC_Os03g55300.1	HLAGGNKAKALLSDGDDDGELGQRM	S	172
1779	LOC_Os03g55330.1	GTVARRQAGPADSPAESYLESRVSF	S	119
1781	LOC_Os03g55570.1	HIWPLNSDGPVTSASPERSTPKEKP	S	220
1784	LOC_Os03g55704.1	EAERVSSMGRKVSFSMEDSRLCRNS	S	826
1785	LOC_Os03g55704.1	HVVSFDAAVRSVSQELEHGKDFTEE	S	700
1786	LOC_Os03g56060.1	LAPKELKHQKTESAPNLDAIAKEQS	S	382
1788	LOC_Os03g56460.1	DQWGVELGKSLASQVRKSLHASRVE	S	520

1789	LOC_Os03g56800.1	DGHRVKKYRGMGSLEAMTKGSDARY	S	405
1790	LOC_Os03g56900.1	MDTIAENLPKQKSGKFDVGAASDKM	S	29
1792	LOC_Os03g57160.1	KIYGSTKADSVESGEKIGGHAVGHA	S	776
1793	LOC_Os03g57160.1	LGQAQNHAMRSDSAASLYMDDHVTP	S	914
1795	LOC_Os03g57160.1	LGRDLMIGQPTGSEVGDHATSYESR	S	457
1801	LOC_Os03g57310.1	LDDIETHVGRARSFVDRGREQLVVA	S	262
1802	LOC_Os03g57430.1	LRKDGSSKLLSHSFKDLGNKDIRSD	S	344
1803	LOC_Os03g57430.1	SVKHSLDPELNGSPSSKDKLRKDGS	S	325
1804	LOC_Os03g57430.1	KHSLDPELNGSPSSKDKLRKDGSSK	S	327
1805	LOC_Os03g57430.1	HSLDPELNGSPSSKDKLRKDGSSKL	S	328
1806	LOC_Os03g57430.1	SRTIGSDLTDGGSPIRDSPSSKRLL	S	204
1810	LOC_Os03g57430.1	ISSDNVLAASPQSNKMAEKIFEQLN	S	249
1813	LOC_Os03g58250.1	KIGRTASLQRVASLEHLQKRMCGGP	S	406
1814	LOC_Os03g58480.1	TRAKSPKFTRRRSCSDAPPTPEAAN	S	306
1815	LOC_Os03g58480.1	AKSPKFTRRRSCSDAPPTPEAANTT	S	308
1816	LOC_Os03g58480.1	KLIDQEKSGPPKSPSKPGISGSDRS	S	110
1817	LOC_Os03g58480.1	IDQEKSGPPKSPSKPGISGSDRSKR	S	112
1819	LOC_Os03g58590.1	LRKHLHRLEHPDSIEQDKESEDEID	S	408
1820	LOC_Os03g58740.1	PKPAPSRLESSLSFSAPLHKVEGRA	S	254
1821	LOC_Os03g58810.1	PPKVNIDLESSASKHRRKMRKVDGG	S	553
1822	LOC_Os03g58810.1	FKNPPKVNIDLESSASKHRRKMRKV	S	550
1824	LOC_Os03g59250.1	TEPFVSDNRHSMSLSPEKDRYYSTR	S	92
1825	LOC_Os03g59250.1	PFVSDNRHSMSLSPEKDRYYSTRGS	S	94
1826	LOC_Os03g59340.1	KIAERMLTWRMNSGRNDDIVHSKYD	S	112
1829	LOC_Os03g59390.1	SRQYSRQRFSNLSLKLQKDGSISDD	S	524
1830	LOC_Os03g59480.1	GSKDLDKELLENSQDNLEDKAQSSD	S	370
1831	LOC_Os03g59480.1	DFLDVEAEEEDDSDDDMMRFKDNEE	S	463
1834	LOC_Os03g59680.1	FKDLSCTPSRSISPEDDAGVEGSGH	S	49
1835	LOC_Os03g59760.1	DDDDDDEDDEDDSDHELEDLFRRQR	S	96
1836	LOC_Os03g60110.1	LYSPGFSPARNLSPQIRSNPTDVDS	S	16
1838	LOC_Os03g60240.1	DNSRHDVQLQSSSPCREELETVKAP	S	929
1840	LOC_Os03g60460.1	QVGRMKYLRPLYSSLARCSGEEKML	S	571
1842	LOC_Os03g60530.1	FFSIARDIKQRLSETDSKPEDRTIK	S	182
1844	LOC_Os03g61010.1	GIQHDKENTERISLSAIAKVYARAD	S	335
1847	LOC_Os03g61160.1	GDEKKVRLMRNRSLTREEVDAFWRR	S	26
1849	LOC_Os03g61160.1	PRAAGDISPLAASPGRAQQEMSSSR	S	88
1853	LOC_Os03g61220.1	GGGSRGGRGGWDSDGEDRFRRGGRS	S	670
1859	LOC_Os03g61930.1	GTSSVLEPEVELSHDGKIREIAMDS	S	510
1860	LOC_Os03g61930.1	KSDLQSKTKEHASDGTSSVLEPEVE	S	496
1864	LOC_Os03g62340.1	SFGAKGLIRRSSSFSSDIQKDVSVC	S	426
1867	LOC_Os03g63320.1	AARARVSGSRDASGDESDGVADWGL	S	168
1868	LOC_Os03g63320.1	RVSGSRDASGDESDGVADWGLPNGR	S	172
1869	LOC_Os03g63320.1	EEAEHYYDDADQSDSAAAAAARARV	S	149

1871	LOC Os03g63650.1	ESFYSQVGSTRGSPAATPSPAPLTP	S	175
1872	LOC Os03g63670.1	NPSQNEDKDEDESEEEEEEEEGPVI	S	216
1874	LOC Os03g63670.1	NEHIEGELSTDESDSESSAYLSSRD	S	619
1875	LOC Os03g63670.1	HIEGELSTDESDSESSAYLSSRDEL	S	621
1876	LOC Os03g63940.1	MDVDNDNFQRTVSLSDGIIQEGPQR	S	136
1877	LOC Os03g63940.1	VDNDNFQRTVSLSDGIIQEGPQRIS	S	138
1878	LOC Os03g63940.1	LTRDFDQINTILSPSTPGSRMNMDV	S	114
1879	LOC Os03g64210.1	IQARIAQLKRELSQTDSAYDSEKLA	S	394
1880	LOC Os03g64210.1	PRGRNVVLDEFGSPKVVNDGVTIAR	S	86
1882	LOC Os03g64400.1	DSSPSPDAFAGWSDGDEQDEQDKSP	S	56
1883	LOC Os04g01480.1	GAQHPMWKTSLCSFFRRRAASSADG	S	58
1885	LOC Os04g01490.1	VKFFNARISRTSSLPVTDVSQEQAD	S	156
1886	LOC Os04g01490.1	KLNNGKSFKRMNSLGGVYRVVPSTP	S	206
1890	LOC Os04g02000.1	DTSSTLGTKRLASEELANEWDNKRL	S	231
1891	LOC Os04g02150.1	SDDIEAMKRDGVSGDEIVEALIANS	S	148
1894	LOC Os04g02870.1	GYGRRDDSPYRRSVSPVYRSRPSPD	S	192
1895	LOC Os04g02870.1	GRRDDSPYRRSVSPVYRSRPSPDYG	S	194
1899	LOC Os04g04680.1	GTIARTRTKPVLSLSKRRPQLEHEP	S	37
1901	LOC Os04g06770.1	TFLKFEDMSDASSSQGGHTSVGSVP	S	881
1908	LOC Os04g08740.1	PVAAIRMPMLKASNFKGGTPEVMET	S	291
1909	LOC Os04g08740.1	VMQTSTVNREYLSLVRRAFNLHSLV	S	443
1910	LOC Os04g14510.1	AIHKDKNYVRTLSICGDKVDGVEHS	S	219
1915	LOC Os04g17700.1	RPAGPLPSDVEDSDDSNAGDGAGEA	S	56
1916	LOC Os04g17700.1	GPLPSDVEDSDDSNAGDGAGEALRK	S	59
1919	LOC Os04g18090.1	GASLPANYKKMLSIQLRGFASKGKL	S	80
1920	LOC Os04g21220.1	LLLTFRPGPSQRSLSLFAGSVWGFQ	S	292
1921	LOC Os04g24170.1	LETEVLQSQQVSSPAQSKTGSSVHE	S	458
1923	LOC Os04g24210.1	KFVSQLGVMTSTSRPMDLYCDNSGA	S	622
1925	LOC Os04g25550.1	ASCSKLVKDVAYSFNDEDEVLPVKK	S	446
1927	LOC Os04g28090.1	GSTATRALLSSYSQTPRLGMTPLRT	S	340
1929	LOC Os04g28180.1	RTLDSHIEEQFGSGRLLACISSRPG	S	177
1935	LOC Os04g31090.1	VEDLWEVAEPQLSPSEKLNSCFEDI	S	111
1936	LOC Os04g31190.1	GSSSSKSVARVHSLDNLISIIQLRE	S	607
1937	LOC Os04g31330.1	SKNAADDMIRILSGFDHRFSSITAD	S	59
1939	LOC Os04g31330.1	GLFCSLRRLSLESMDDLDTSSEFDP	S	194
1940	LOC Os04g31340.1	GSPVANLVSRLGSFTFRRTSSGRVE	S	137
1941	LOC Os04g32920.1	ATATASRLKRHDSLFGDAEKVSGGK	S	31
1945	LOC Os04g33110.1	THAGSFKCRLHRSSSHGHPSSSPTA	S	77
1947	LOC Os04g33110.1	CRLHRSSSHGHPSSSPTAAAAPAAA	S	84
1948	LOC Os04g33110.1	HSSGTTPTGAAASPKMRRSWSSAAS	S	33
1952	LOC Os04g34450.1	KSLRLITDASVSSPVSREKAQGLDP	S	205
1958	LOC Os04g35700.1	AGAASAASSPRKSLDLVDLSASPSV	S	34
1961	LOC Os04g35700.1	RTNMSLPVSPCSSPLRQFKQSNWSC	S	642

1962	LOC_Os04g35700.1	ISVVRTNMSLPVSPCSSPLRQFKQS	S	638
1963	LOC_Os04g35800.1	PSPPLPPRKRRLSPTPSPTRRSSRS	S	54
1964	LOC_Os04g35800.1	LPPRKRRLSPTPSPTRRSSRSRSRS	S	58
1969	LOC_Os04g36790.1	QMELSQVKDHSSSPANAAAPPGATG	S	326
1970	LOC_Os04g36790.1	PGGGGGGREDAWSEGATAALIDAWG	S	69
1974	LOC_Os04g37540.1	SNPASPVLGRSYSVEPLRKPIVNQK	S	382
1976	LOC_Os04g37950.1	DILEKESNAAKDSPQSSSPEKVPNG	S	140
1980	LOC_Os04g38570.1	SLSLRSGSLRNLSYSYSTGADGRIE	S	650
1987	LOC_Os04g39040.1	PSGFPGGGGGGGSDSDEPQEYYTGG	S	46
1989	LOC_Os04g39040.1	TIRTLADISRGPSGFPGGGGGGGSD	S	35
1990	LOC_Os04g39090.1	TSALPSRIPSARSMDDLLKLDSSRE	S	174
1995	LOC_Os04g39629.1	YMYDREAAKGTDSASDIDRHDDTGA	S	357
1996	LOC_Os04g39629.1	YDREAAKGTDSASDIDRHDDTGAAR	S	359
1999	LOC_Os04g40080.1	AIVAATDAEAGGSRRVPEGDLEACR	S	56
2000	LOC_Os04g40400.1	PQIVVSIVDRPRSRGGMEPFPKPAE	S	491
2003	LOC_Os04g40430.1	SLPREPKPEIDDSDNSDDPDFWVPP	S	185
2009	LOC_Os04g40720.1	CCRDTSMEDRGKSSEGCPGAEVHVD	S	127
2011	LOC_Os04g40720.1	KDKGTGTIFNDASSDGSDDELGIGS	S	1299
2012	LOC_Os04g40720.1	TGTIFNDASSDGSDDELGIGSEKAA	S	1303
2013	LOC_Os04g40720.1	DKGTGTIFNDASSDGSDDELGIGSE	S	1300
2014	LOC_Os04g40720.1	EPNEKINFLDHFSPKGTNDQYVSAE	S	1015
2016	LOC_Os04g40720.1	KYPAAIHSDAPISSPSHNKARGEKL	S	748
2017	LOC_Os04g40720.1	YPAAIHSDAPISSPSHNKARGEKLE	S	749
2018	LOC_Os04g40720.1	AAIHSDAPISSPSHNKARGEKLEVL	S	751
2023	LOC_Os04g41030.1	IPDSLGRLDRLKSLDLSDNRLDGQI	S	178
2025	LOC_Os04g41030.1	KGGRRRRRGGSESGGGSAEDGSWWA	S	267
2026	LOC_Os04g41100.1	SRDGESVVAASASSPSKKRKFSPII	S	131
2027	LOC_Os04g41100.1	RDGESVVAASASSPSKKRKFSPIIW	S	132
2028	LOC_Os04g41100.1	GESVVAASASSPSKKRKFSPIIWDR	S	134
2032	LOC_Os04g41100.1	QDHIPERLAVEKSPMDVEPAVASES	S	193
2038	LOC_Os04g41100.1	QSGMDSEYEVRRSETPEPVKPPHRC	S	340
2039	LOC_Os04g41100.1	ASASSPSKKRKFSPIIWDRDSPKPM	S	140
2040	LOC_Os04g41160.1	PSPPEPALPRELSLGDLRAVSMLGR	S	20
2041	LOC_Os04g41820.1	SLSSRDVAAMPDSPPRRAARHRRAQ	S	131
2046	LOC_Os04g42140.1	LDNDNWEVPRSKSMPRGDSLRNQGP	S	529
2047	LOC_Os04g42320.1	DHNLKKSHKLDTSTDSEVVDHSKAV	S	393
2048	LOC_Os04g42320.1	LKKSHKLDTSTDSEVVDHSKAVNNN	S	396
2049	LOC_Os04g42320.1	NNNEDILVSRELSPETDDGDNKLPP	S	418
2053	LOC_Os04g42320.1	DTPPDEDTDEDLSLKDIVSPKSSAK	S	539
2054	LOC_Os04g42320.1	DTDEDLSLKDIVSPKSSAKTGKNKG	S	545
2059	LOC_Os04g43130.1	AGTDGCITSPMQSSSPKVRPDQEYL	S	345
2060	LOC_Os04g43140.1	RNAESMGLLLETSDSEEERVQGHKQ	S	688
2061	LOC_Os04g43140.1	AESMGLLLETSDSEEERVQGHKQRK	S	690

2063	LOC_Os04g43300.1	ANDDNRELKRQKSTDQDDRQPAVAG	S	234
2065	LOC_Os04g44640.1	GGGHRRYSGSKRSYDFDRERRGGGG	S	165
2066	LOC_Os04g44740.1	SFGIHLWNRESRSLEMEEGSVIGRL	S	435
2068	LOC_Os04g44800.1	GEAAAAVAARSRSKSIEADEEERSK	S	25
2070	LOC_Os04g45190.1	QPPRPPSPVREGSEEGSSPRASLSE	S	141
2073	LOC_Os04g45190.1	ATRAHKDAEDVTSDSETSALISRLT	S	186
2074	LOC_Os04g45190.1	RAHKDAEDVTSDSETSALISRLTEE	S	188
2079	LOC_Os04g45800.1	SRQKHLVFDNKDSKKAKNNEQKNDV	S	62
2083	LOC_Os04g46320.1	SSPNARDIHLPSSFKDLVLERKISD	S	307
2087	LOC_Os04g47380.1	GSTDDDKQILSESDAFINKNLEGIQ	S	99
2088	LOC_Os04g47700.1	CANFAPVSEKRNSSIKDDENIELDI	S	613
2089	LOC_Os04g47700.1	ANFAPVSEKRNSSIKDDENIELDID	S	614
2090	LOC_Os04g47870.1	AFRARFYMEPETSDSGSMASGAATS	S	1071
2092	LOC_Os04g47912.1	NPCEAYCGRSTSSLDEFEVRSAMAV	S	207
2095	LOC_Os04g49060.1	VLLQDRVAELIGSCSPGNQFNDADS	S	164
2096	LOC_Os04g49060.1	LQDRVAELIGSCSPGNQFNDADSSD	S	166
2098	LOC_Os04g49510.1	DTDDNNNAAAADSPKKPSRPPAAAK	S	37
2101	LOC_Os04g49520.1	IMMNGYAKQGSLSDAEELFDRMPRR	S	88
2103	LOC_Os04g50204.1	GSHKEESNVKGDSEDDEPVMVVAKM	S	674
2105	LOC_Os04g51040.1	LEALRRLMKQHLSLKSEKALRELME	S	695
2112	LOC_Os04g51120.1	VETAAKDPFDFSSSNLGKTPLADPK	S	447
2113	LOC_Os04g51120.1	ETAAKDPFDFSSSNLGKTPLADPKA	S	448
2114	LOC_Os04g51120.1	SSTGITYKSSSASFGSNYSSGERYG	S	178
2117	LOC_Os04g51370.1	HWLAAGLSPQVGSVNRSLPPNNPYP	S	512
2118	LOC_Os04g51700.1	LDADYISKMEEDSSDRDQGAHPTAP	S	960
2119	LOC_Os04g51700.1	QHAKIDDVEPEESDHGETGLDDQIP	S	879
2120	LOC_Os04g51700.1	RGKQHAKIDYGQSEESDPGETGQDD	S	933
2124	LOC_Os04g51710.1	GPLNSSGAQRKVSGPLDSAASKKTR	S	177
2125	LOC_Os04g51710.1	QRKVSGPLDSAASKKTRATSFSHNQ	S	185
2127	LOC_Os04g51710.1	GPLNKHGEPVKRSSGPQSGGVTPMA	S	128
2130	LOC_Os04g51710.1	KMFELHAVKSRKSGPLSNAPSRNAS	S	62
2132	LOC_Os04g52050.1	IDRIINSICPSSSPRHMTPEKVSNE	S	522
2133	LOC_Os04g52050.1	PDIDRIINSICPSSSPRHMTPEKVS	S	520
2134	LOC_Os04g52050.1	DIDRIINSICPSSSPRHMTPEKVSN	S	521
2138	LOC_Os04g52120.1	LIPNQVHSEKRMSSFRLKLPTPELE	S	223
2139	LOC_Os04g52120.1	IPNQVHSEKRMSSFRLKLPTPELER	S	224
2141	LOC_Os04g52180.1	NNKSKSFLKPIGSISKKKVQLHLKI	S	91
2145	LOC_Os04g52540.1	AARSSGTVERIASTEVVRVQPPAPP	S	169
2147	LOC_Os04g52570.1	PKLTPGSTPKFLSGPTSPTKSLSEV	S	201
2150	LOC_Os04g52920.1	WASKEELELVSASPSIADLERMKKE	S	403
2157	LOC_Os04g52960.1	GRDNSSFKKPAQSSGNTIFIKGFDT	S	546
2160	LOC_Os04g52960.1	KKSAVEVAPTSVSVSEGKSGKKGKR	S	18
2163	LOC_Os04g53540.1	SDPLGGDEFESKSGSENVDGVSVDD	S	84

2165	LOC Os04g53670.1	NTFSPRKLSRQNSFGSIGTPRTPNL	S	1127
2169	LOC Os04g54340.1	RSVTAQSNLNSFSDDEDTREMLLGA	S	539
2170	LOC Os04g54440.1	MAPKKRKADPAESPVASSEAGAGTN	S	13
2174	LOC Os04g54940.1	KSLQANRLSSANSADGGPCVGKVNV	S	166
2175	LOC Os04g55040.1	AKESMGEAMRASSFSLAEAKYVAGD	S	67
2176	LOC Os04g55220.1	KEETPNPFRQQFSMTSLERTMTSME	S	406
2182	LOC Os04g56140.1	AEPEIHMESDINSASELRTKDTGPH	S	464
2183	LOC Os04g56140.1	ESRRSRLGSSGSSPDASKVRQYGST	S	334
2186	LOC Os04g56160.1	PSSALLGQNKDASLEALPVDELIEK	S	545
2190	LOC Os04g56440.1	SLLDAIARLDDASDSESEDMAVGAQ	S	373
2193	LOC Os04g56530.1	HVSGQLDYVRHLSGLERHESILPLL	S	916
2194	LOC Os04g56530.1	SVTQNGSTPRRRSGQLDPPMLEISK	S	790
2196	LOC Os04g56530.1	SLLEKEPPSRHVSGQLDYVRHLSGL	S	906
2199	LOC Os04g56630.1	SSADHSDGTGLSSPKGALLGLASYD	S	585
2200	LOC Os04g56630.1	PKGALLGLASYDSDDEDDEGDNEDK	S	598
2201	LOC Os04g56640.1	LEDQGVRQLYPKSPDIDFKKELRTL	S	66
2202	LOC Os04g56720.1	SNKRKKTKDVSESEEDDDEEDDSDD	S	467
2205	LOC Os04g56740.1	ACAGGGSDPSAGSDGDADDHVDENK	S	25
2206	LOC Os04g56740.1	LKFEELTSNLTVSRSNSLDGANKPK	S	565
2207	LOC Os04g56740.1	ELTSNLTVSRSNSLDGANKPKVHTP	S	569
2208	LOC Os04g56740.1	GGKFQDSLICSSSEKLLNNGFALKL	S	338
2209	LOC Os04g56740.1	RAKASASVSPKLSPDVQDNNPRKRH	S	836
2210	LOC Os04g56740.1	QFTESARAKASASVSPKLSPDVQDN	S	830
2212	LOC Os04g56740.1	FAAAQLKFEELTSNLTVSRSNSLDG	S	560
2213	LOC Os04g56740.1	KDNSVLHSEVNTSGGAIEPEKEVQT	S	669
2217	LOC Os04g57300.1	LLLTRNSINRSCSTPCLCPVSKDFE	S	102
2218	LOC Os04g57300.1	SGSNGSVHALNRSADEQLPSSVYFV	S	547
2219	LOC Os04g57600.1	RRRRINESSLAISPERNGEQRRRDE	S	410
2222	LOC Os04g57600.1	EAGQHEEQTQRRSSELALGSRNADG	S	508
2223	LOC Os04g57600.1	SRLQGRIKLPGRSPDRVDTRSEKER	S	460
2228	LOC Os04g58140.1	AIDTLPPPSRDVSKPLRLPICDVFS	S	480
2229	LOC Os04g58280.1	QKQVAHAPAELNSPRSSAAKPKNPD	S	18
2231	LOC Os04g58620.1	DLAVEGIEQLESSREMSDDESTDKL	S	387
2237	LOC Os04g59330.1	AKTDSEVTSLAPSSPPRSPTSRGGR	S	15
2238	LOC Os04g59330.1	KTDSEVTSLAPSSPPRSPTSRGGRP	S	16
2240	LOC Os04g59400.1	PSQSDEEPLHRQSQFSDAEEGMEGP	S	185
2241	LOC Os04g59400.1	SDEEPLHRQSQFSDAEEGMEGPISP	S	188
2245	LOC Os04g59460.1	SKKKSLSFSSSFSKRTQPSHLFSGN	S	1268
2248	LOC Os04g59570.1	VAARPLRSRRALSVFTCAAPPRQRP	S	25
2251	LOC Os05g01060.1	PQQAYSHVQSPMSSPVRARRDWDLL	S	388
2252	LOC Os05g01060.1	QQAYSHVQSPMSSPVRARRDWDLLG	S	389
2254	LOC Os05g01350.1	RPLPPLPRVGPPSGEFASRSSASDP	S	326
2261	LOC Os05g01990.1	SSAHGKNHTSLLSDDEEEILEKHNV	S	267

2262	LOC_Os05g02240.1	RRRLHHRKLHGPSAPSSPSAAATAG	S	453
2266	LOC_Os05g02400.1	SSACPNKVAAVNSDMEKSYGGSSTI	S	491
2269	LOC_Os05g02500.1	ERRGGFSSLKLPSFQKDLVLPPRVP	S	596
2272	LOC_Os05g03060.1	AAEASRLDPAGDSPEMRRVVDPDGA	S	51
2273	LOC_Os05g03060.1	SHTSESFSTRSSSLSPSLRNSNDHA	S	428
2274	LOC_Os05g03060.1	TSESFSTRSSSLSPSLRNSNDHAPT	S	430
2275	LOC_Os05g03060.1	ESFSTRSSSLSPSLRNSNDHAPTRA	S	432
2277	LOC_Os05g03120.1	LKKAHNKDDVDGSFGSPDHGEAVEK	S	301
2279	LOC_Os05g03120.1	AGITPTRTTPERSPKVTERRSPRSP	S	50
2280	LOC_Os05g03120.1	ASSPFSDEPEEESPKRRNSGVLRRM	S	582
2281	LOC_Os05g03120.1	EYTNSMIGGKLASSPFSDEPEEESP	S	571
2283	LOC_Os05g03120.1	SMIGGKLASSPFSDEPEEESPKRRN	S	575
2290	LOC_Os05g03440.1	KDAAAAAEIAAPSPRKSQKASAAAE	S	268
2291	LOC_Os05g03630.1	RLAMKHHPDKNRSPHADDSLFKQVS	S	38
2293	LOC_Os05g03630.1	RAPGAGFPGFGGSPRAGETSATKAP	S	132
2296	LOC_Os05g03760.1	RLLLKSPAVSPSSSPKKSASPPSPP	S	173
2298	LOC_Os05g03760.1	KAIRLLLKSPAVSPSSSPKKSASPP	S	170
2299	LOC_Os05g03760.1	QGDELHKLRKSASFAFRGQSAMPVA	S	551
2301	LOC_Os05g03780.1	PPQIAEVRMDISSSTSVAAGNKVCR	S	20
2303	LOC_Os05g03820.1	KEGKPNGYLSLRSHIWTDTDNNRSG	S	271
2304	LOC_Os05g05220.1	SRRGKKAWRANISTDDIEDFFEKQT	S	20
2305	LOC_Os05g05230.1	RPDGSELPKGSESDDCSQISGLKVE	S	377
2309	LOC_Os05g05300.1	GTTEIKEPHDQQSEDSDQEELDRFI	S	331
2312	LOC_Os05g05300.1	EKPRNSATASGRSVFRESEDDELDT	S	488
2315	LOC_Os05g05590.1	FGGRGFVPFVPGSPVERSIHGSQLG	S	524
2317	LOC_Os05g05720.1	SNTRSSVTATGRSPSPNSSSLAPLN	S	94
2319	LOC_Os05g05950.1	GIRHGRQTNRAFSLDNARKKAMLLE	S	646
2321	LOC_Os05g05950.1	AAKQIMKELGEGSASVSPVSGLSSS	S	467
2325	LOC_Os05g06630.1	SKPDSDIAEHDDSPKSSSGRLTRSR	S	39
2327	LOC_Os05g06980.1	SRAQITEDQDKSSGNISVENIEGLK	S	247
2330	LOC_Os05g07000.1	RSHNLTSDGMNPSPRERDDQNGSHR	S	269
2331	LOC_Os05g07000.1	ALPANGRSHNLTSDGMNPSPRERDD	S	263
2334	LOC_Os05g07130.1	RGVGSTKFGKSSSACSLASGLNFGT	S	330
2335	LOC_Os05g07220.1	KDNWADSDVENGSISRSSDEKESRE	S	235
2337	LOC_Os05g07680.1	RCHCNFGANSELSEHEEKQQVSDVE	S	217
2339	LOC_Os05g07680.1	SELSEHEEKQQVSDVETSSLQKDLD	S	226
2340	LOC_Os05g07980.1	PAQDRWRRKRKVSFPPRHALSPPIF	S	586
2341	LOC_Os05g08370.1	IQRQGEDVDLSSSSRHEQHRIPRLT	S	141
2343	LOC_Os05g08600.1	RRPPSVKASLSVSFGQRAPHRASTR	S	207
2345	LOC_Os05g08840.1	KMDTDDALGDPASGTDENMQESKCS	S	526
2347	LOC_Os05g09540.1	PAASVPWRWLAFSLCRFLDLPADGS	S	86
2351	LOC_Os05g10770.1	RPPIDYSRFEHISDEDSDVEIVEKD	S	98
2354	LOC_Os05g11370.1	PQGEDFDKLESDSDDCKEVAALSAA	S	199

2355	LOC_Os05g11770.1	IEPSLFFPKQYFSDTEEFEDVIYPK	S	280
2356	LOC_Os05g11770.1	EDIVESVMMLGGSKSDTELDAEPER	S	757
2357	LOC_Os05g11770.1	IVESVMMLGGSKSDTELDAEPERTA	S	759
2358	LOC_Os05g11770.1	VSSANKDAIVCLSNHDEKNESPPAD	S	640
2359	LOC_Os05g11770.1	IVCLSNHDEKNESPPADSYNHLDLR	S	648
2361	LOC_Os05g11980.1	ISQKQEALQDTYSGDEIIDSMHRSG	S	1085
2362	LOC_Os05g12100.1	SVQHCRHAQHTTSTPRRPACPEGDE	S	133
2364	LOC_Os05g13520.1	ADFDDVAAAMKGSMKLRAEADFDPL	S	309
2366	LOC_Os05g18770.1	SSVDKITDKKDLSHTIRGESVLGKS	S	67
2372	LOC_Os05g22940.1	TKLSELRARIARSLSELEMFTEESK	S	1200
2374	LOC_Os05g22990.1	KVSEPTGSPAKDSPVGKEKAIEKSQ	S	722
2376	LOC_Os05g23320.1	IEAVHLDKNGQASPDTSVVIRRTPK	S	614
2377	LOC_Os05g23320.1	PTPLRQKYSRMSSLFNNKRTMAARI	S	550
2378	LOC_Os05g23610.1	QDDDPEAEKSRLSFKEHRRAHYDEF	S	106
2381	LOC_Os05g23610.1	SKTDPKDDGWASSDDDADAMEQDDD	S	85
2388	LOC_Os05g27820.1	SSLMEATALLRSSSFKEDSYVASAL	S	45
2400	LOC_Os05g28190.1	DALPVTDKSAGSSEVAETEKDGSDL	S	128
2406	LOC_Os05g30340.1	LRVSVKRESGAHSEDESVAPTVSVK	S	392
2407	LOC_Os05g30340.1	PCKRLRVSVKRESGAHSEDESVAPT	S	388
2408	LOC_Os05g30420.1	ARAPVDEFEPVDSGSDLESDDLKSA	S	110
2411	LOC_Os05g30510.1	GGEQQSSAAAASSGDGVAAAANVDR	S	48
2413	LOC_Os05g31920.1	ETKSNAQLSRLSSLESFKHMDSRTS	S	763
2415	LOC_Os05g32070.1	QQLAGAASSPPTSSAFPAATTASAK	S	166
2416	LOC_Os05g32070.1	RRRERQQLAGAASSPPTSSAFPAAT	S	161
2420	LOC_Os05g32600.1	KLADFGLARIFGSPERNFTHQVFAR	S	168
2422	LOC_Os05g32760.1	IQEPEDEKYVPASPPRPPLSPPTPV	S	269
2426	LOC_Os05g33100.1	VVKQFEQVLSSKSGIGHFVKIFPGV	S	193
2427	LOC_Os05g33310.1	FVRVRPSTPVDLSPLPSPTKAMTLW	S	243
2428	LOC_Os05g33310.1	RPSTPVDLSPLPSPTKAMTLWQAPP	S	247
2431	LOC_Os05g34500.1	HQQQQQPHRRNLSLEELAGEVGLSP	S	71
2435	LOC_Os05g34780.1	ENITPEDESLDSSEDNTDSIMERKS	S	340
2436	LOC_Os05g34780.1	MAIMIEKNVDVDSDGERVDFSDRET	S	143
2437	LOC_Os05g34790.1	HGKSGDMTVDLDSDGSEDHGTRQHE	S	156
2440	LOC_Os05g36090.1	GRSASDVIRRSASGHEAAPPFQTET	S	416
2444	LOC_Os05g37434.1	LDPQVGSPQNKTSPTKHSLAEPMNM	S	131
2447	LOC_Os05g37720.1	GREEEDELGAFLSGMRLLRYLYYRT	S	141
2452	LOC_Os05g38710.1	DVSNEDTELEIASPSFGKYDTFKSC	S	509
2455	LOC_Os05g38810.1	IRRAASQLTGSSSIVKSQNEQVPHK	S	563
2458	LOC_Os05g38810.1	NIAETENGDFDVSSDDAINELSVEK	S	588
2460	LOC_Os05g38810.1	DEFDGLQSKKKRSLDSFEDPETKAA	S	525
2461	LOC_Os05g38810.1	DGLQSKKKRSLDSFEDPETKAAAPS	S	528
2462	LOC_Os05g38810.1	KPQDGLELTEKKSTSNLSTDNAYDH	S	490
2467	LOC_Os05g38810.1	PETKAAAPSFGSSFKIGECIRRAAS	S	544

2468	LOC_Os05g38830.1	TSGSGVMTERVPSFSRKKFKVDRDN	S	125
2469	LOC_Os05g38950.1	LQYQTSQQKALLSPLPSPRTSMIDA	S	699
2470	LOC_Os05g38950.1	TSQQKALLSPLPSPRTSMIDASPGR	S	703
2471	LOC_Os05g38950.1	PLPSPRTSMIDASPGRANHDSSQEF	S	712
2473	LOC_Os05g38950.1	QVETPKLDVLEESMGSPKEDNKSRV	S	786
2474	LOC_Os05g38950.1	VIHPVKVQEGKLSEDSDDEFYDVDK	S	143
2476	LOC_Os05g39390.1	EREKVQAVFKRLSSDPVGIRVHDVI	S	61
2482	LOC_Os05g39730.1	PRRSPGPSPLGGSPTMGDRTMKAPT	S	530
2483	LOC_Os05g39840.1	LGQILSQKYDALSPLRIDHGGASRR	S	503
2485	LOC_Os05g40420.1	AVGLPSDDDMGNSEVGHNALGAGRI	S	81
2486	LOC_Os05g41100.1	KKKDILAGSDNESDGGGGEEDLSKI	S	22
2487	LOC_Os05g41100.1	EEEKKKKDILAGSDNESDGGGGEED	S	18
2489	LOC_Os05g41100.1	RPYLETGSEKKYSPDDIQEVSDEEE	S	277
2492	LOC_Os05g41480.1	QALKAKEAQKKISQADEEAGSLLQE	S	329
2495	LOC_Os05g41670.1	NDDEDEEPHMGSSSHGGDRIKNRSL	S	222
2496	LOC_Os05g41790.1	SQHVDTNVIRGGSLEKPLTFADKLA	S	508
2498	LOC_Os05g41790.1	SNALSSQRFGSSSPGGDMKNKTRKR	S	111
2500	LOC_Os05g42220.1	TENESGKIKKSQSLGDMLEMEKLYD	S	80
2501	LOC_Os05g42230.1	LAGGAAAAIQPNSPRFFFSSLAAAS	S	38
2502	LOC_Os05g42300.1	DSLFEEGELMLASDDDDEEAPKGPI	S	230
2503	LOC_Os05g42300.1	GENDEDQTDPEFSDDEKEAEYKRSL	S	379
2504	LOC_Os05g42300.1	NMKELYAKGYDGSGENDEDQTDPEF	S	366
2505	LOC_Os05g43060.1	WGEACKETEKTGSDTDPWGSKVKEI	S	795
2506	LOC_Os05g43280.1	RDEDRSLSGESLSEWRSCEQVESES	S	30
2510	LOC_Os05g43380.1	HSEDEQDFIVERSPRLQSPISKESS	S	242
2511	LOC_Os05g43520.1	RTSLGQEEDVSFSDLEDDDDDGNKG	S	414
2516	LOC_Os05g43670.1	EEEEDDDEDAPASLPPPPRRARAPP	S	188
2518	LOC_Os05g43860.1	SRDMVPPLPPLPSMKSPSESWLSRA	S	510
2519	LOC_Os05g43950.1	VPSPASSLQNSQSQGNNREASDPPS	S	93
2520	LOC_Os05g43970.1	QESDEEYDRSQESDEEESDNFQKNK	S	60
2522	LOC_Os05g44320.1	DAGALDLIDDEDSDVEEGIDQQTRG	S	681
2525	LOC_Os05g44380.1	ASELLLPVASSPSPRSAAAAASRSP	S	89
2527	LOC_Os05g44922.1	DSYPNLRALRNASSVSLADAAYVKI	S	71
2528	LOC_Os05g44922.1	PNLRALRNASSVSLADAAYVKISEG	S	74
2529	LOC_Os05g45060.1	ITVPRVIFVADDSDSPGSSSRGGAG	S	84
2530	LOC_Os05g45060.1	VPRVIFVADDSDSPGSSSRGGAGGG	S	86
2531	LOC_Os05g45280.1	MGDEKSPLSQMGSRDRDRELLIPVS	S	13
2544	LOC_Os05g46500.1	LQRSSETFRRSGSSGLVWDDRHLSG	S	16
2546	LOC_Os05g46620.1	FQGAKSLYHRENSLQVETSPRQGAA	S	323
2548	LOC_Os05g47470.1	EDEDEAAAPATPSKEAEVGYHGLMA	S	178
2549	LOC_Os05g48020.1	IGEGLETLKNMASDMNEELDRQVPL	S	198
2551	LOC_Os05g48260.1	ERDVEAAVPVPMSPAGRLFRETNFN	S	93
2552	LOC_Os05g48260.1	TPSPSSLRKRVLSIDTSSRDSRGSP	S	39

2553	LOC_Os05g48290.1	QVGDMEKLGISESFKAKQAVLMSAT	S	501
2554	LOC_Os05g48820.1	EKDVDDAERDEDSECDDADDDPDGD	S	910
2555	LOC_Os05g49050.1	DGLAPHSHEPIYSPGDFSKRAPPLI	S	62
2559	LOC_Os05g49180.1	GAFSGLLPVPSRSADDNNVELPVIE	S	427
2560	LOC_Os05g49230.1	DSENEDIQTREYSDEEMDSDEEQQR	S	441
2565	LOC_Os05g50480.1	KTANGDEEVAAVSDNHSIDQSDVRI	S	453
2566	LOC_Os05g50480.1	GDEEVAAVSDNHSIDQSDVRIHEYE	S	457
2567	LOC_Os05g50480.1	VAAVSDNHSIDQSDVRIHEYENKDT	S	461
2570	LOC_Os05g50480.1	TQPGLGHEHRNPSDGNLSSAAAAAQ	S	841
2571	LOC_Os05g50530.1	LPKDIPLPTESVSRGKRRAVSAIES	S	181
2572	LOC_Os05g50530.1	RNLPKDIPLPTESVSRGKRRAVSAI	S	179
2573	LOC_Os05g50530.1	TESVSRGKRRAVSAIESSFNLDARA	S	189
2579	LOC_Os05g51630.1	RRNTPAQSKYEGSDTLSLPETVHER	S	754
2585	LOC_Os05g51850.1	AVQNGGEVRCLLSDGASSMGNRGPG	S	376
2586	LOC_Os05g51850.1	GGEVRCLLSDGASSMGNRGPGSPRK	S	380
2590	LOC_Os06g01400.1	KFFYKMDTQKKLSEFRDQLSSILFH	S	721
2591	LOC_Os06g01650.1	PSSTIALRESSASPPPSVQSIPVQS	S	1070
2596	LOC_Os06g01680.1	NAFVEKKLQPQSSNISLEPKVIDAT	S	305
2597	LOC_Os06g01700.1	DDSDVEPRSDDESDEDDDDDDDTEA	S	123
2599	LOC_Os06g01700.1	DKIVPREIDADDSDVEPRSDDESDE	S	113
2600	LOC_Os06g02028.1	LTTYDFEADCFSSPYDDLNKRKLAY	S	110
2601	LOC_Os06g02130.1	DSRYESKEFDDVSEQYVAVTKKEKR	S	183
2602	LOC_Os06g02160.1	KAYGISRMNLVPSLMEIILPSLEKN	S	299
2607	LOC_Os06g02180.1	GGLPASRMERRLSIMRSQKAMTRSQ	S	229
2609	LOC_Os06g02370.1	EKRLGMEKGEGMSAVADSGEGRGRV	S	573
2611	LOC_Os06g02380.1	QKVGAEIVRRALSYPLKLIAKNAGV	S	505
2612	LOC_Os06g03676.1	ASDSQLLEKFDLSDSEDATSDESNE	S	504
2614	LOC_Os06g03910.1	LYEMAKREGLLPSSPTTSRRRGSSS	S	309
2616	LOC_Os06g04330.1	SRIRRMIEGLDDSDEPEDIFDPVFK	S	403
2624	LOC_Os06g05190.1	QSKPRSDDAQYDSDDTIEMTEEEID	S	322
2626	LOC_Os06g05310.1	RFVTPLPMGVDMSQQRRRTFLLNAG	S	229
2631	LOC_Os06g06014.1	EDVETGKNDGSLSDVDAMKENSNVG	S	292
2632	LOC_Os06g06014.1	QENNVDAVEKTISSEDQVAEKEDVE	S	271
2636	LOC_Os06g06830.1	DRSAPHATEGASSSRVSPQHGRGNQ	S	124
2638	LOC_Os06g07350.1	PAPPGAERTLYPSMDPQRMGALVKS	S	370
2639	LOC_Os06g07820.1	QSPDDRVLIKTRSNQSIDQWVFAKS	S	268
2640	LOC_Os06g07820.1	DDRVLIKTRSNQSIDQWVFAKSKSE	S	271
2642	LOC_Os06g08023.1	DEFLIKCDGVKNSLLPSMAKLLKLN	S	176
2643	LOC_Os06g08140.1	RVSRPGRVARSISAEGLNSLRTLLR	S	346
2650	LOC_Os06g08480.1	ETKPDASEETSSSESGSKKKPVKRY	S	21
2651	LOC_Os06g08480.1	KPDASEETSSSESGSKKKPVKRYLI	S	23
2656	LOC_Os06g08740.1	PSPLGLRLRKSPSLLDLIQMRLSQA	S	89
2657	LOC_Os06g08740.1	TSDERSLMARVDSLCCLIQKDTPPV	S	453

2658	LOC Os06g08740.1	GPAQPPAMSRKDSFGDLLMNLPRIA	S	513
2662	LOC Os06g08840.1	PRRRSVSPARARSYSRSPQYNRGRD	S	159
2663	LOC Os06g08840.1	RRSVSPARARSYSRSPQYNRGRDES	S	161
2665	LOC Os06g08840.1	PRYRRSPSYGRRSYSPAGRSPRRRS	S	139
2666	LOC Os06g08840.1	SYGRRSYSPAGRSPRRRSVSPARAR	S	146
2667	LOC Os06g08850.1	DLSIRESFFSRRSFEDDGSPEHSLV	S	642
2668	LOC Os06g08850.1	SFFSRRSFEDDGSPEHSLVLNAVRK	S	648
2669	LOC Os06g09570.1	RTFTTNRMGRQLSSMGFDPRAALDR	S	551
2671	LOC Os06g09570.1	GRKRERSLSRAASDGDDMDIDGQQS	S	582
2672	LOC Os06g09930.1	FKRSLPTLTRFKSQQENELTSLIVD	S	305
2673	LOC Os06g10710.1	GDRRPAARAPNPSLSPRGGGGAPSR	S	56
2674	LOC Os06g10710.1	RRPAARAPNPSLSPRGGGGAPSRKS	S	58
2678	LOC Os06g11970.1	LMFSPSGRLSHFSGRRRIEDVLTRY	S	58
2679	LOC Os06g12030.1	FSVEDEKNEGSESISSGNIGSDAEP	S	180
2680	LOC Os06g12030.1	VEDEKNEGSESISSGNIGSDAEPKS	S	182
2681	LOC Os06g12030.1	EKNVAVTEGSTNSEEKDQDEDLLRR	S	20
2682	LOC Os06g12080.1	AVAFLSGLGLSRSGIAAAVAADPRL	S	92
2683	LOC Os06g12160.1	VFHNLARLSPLTSSLKRLVGGLKAR	S	512
2684	LOC Os06g12260.1	ASLGDAGGNADASPAAPALRPAAVA	S	78
2685	LOC Os06g12400.1	NGSKQIDASDLPSDDSADNDYDPTL	S	346
2687	LOC Os06g12400.1	KSAEKLIPGLENSDEARRKAVQREL	S	773
2689	LOC Os06g12580.1	PQQEESYGSEYGSGYGRKPQAESYG	S	225
2690	LOC Os06g12610.1	PRKADGVERDDFSFGNRGVAERDAE	S	386
2691	LOC Os06g12780.1	PSDAGAAPHAPPSPPRRGAKKGAAA	S	126
2692	LOC Os06g13810.1	QASNAPAPTRLASVYSEVQTSRLKH	S	28
2694	LOC Os06g13820.1	PNTDGGSMRQSNSDGALDTMARRPA	S	722
2695	LOC Os06g13820.1	NKIRAVIQSKGGSFKGPNTDGGSMR	S	706
2696	LOC Os06g13820.1	QSSLLSKLTRQLSVHDNRAASYAND	S	826
2698	LOC Os06g14080.1	RGPARRRGTVRASLDADEFIALMHG	S	26
2699	LOC Os06g14406.1	MSQKSGRSSGMESPASLQIEDTRSM	S	171
2701	LOC Os06g14412.1	EVEEKLIDDISGSPSSHLPVALKST	S	1987
2706	LOC Os06g14412.1	EVDKEIVHCTISSPIGDQENLQGNI	S	2427
2707	LOC Os06g14412.1	TRSDIETPAMDASESKSPESDVHEL	S	1366
2709	LOC Os06g14412.1	PAMDASESKSPESDVHELSERVEFM	S	1373
2710	LOC Os06g14412.1	RRGRPRRSDASLSPVTAPPNTGKQE	S	158
2712	LOC Os06g14412.1	KNDDSPIERSSPSADKIAQVAYGGE	S	935
2714	LOC Os06g14412.1	TIEKNDDSPIERSSPSADKIAQVAY	S	932
2718	LOC Os06g16160.1	AFRSALRAAATGSHRHATRRTVIVR	S	690
2720	LOC Os06g16390.1	YYDTNCGEALICSDSEDEAVEDEEE	S	178
2723	LOC Os06g16980.1	RHPKCKYIARTLSDDDDETEASDET	S	1008
2724	LOC Os06g17290.1	TQMAVSALNRALSSEYPSKSRSEGR	S	73
2725	LOC Os06g17290.1	SNHSGSQVGNWRSANEQLPTSASFV	S	641
2726	LOC Os06g17840.1	EINLDNQENLGESEKSKPKKKRMDA	S	1197

2730	LOC_Os06g17840.1	KKGAKKPHAEILSSSPKKSDEAGSS	S	1304
2735	LOC_Os06g17840.1	ENKVEGAKKRDDSVDTELVTSPASV	S	1272
2737	LOC_Os06g18130.1	IQNKDARSGKEASSSGGGDCTGVNF	S	74
2738	LOC_Os06g19390.1	MDLPWSEMFRSASLRLPKQEEPTTT	S	32
2739	LOC_Os06g19650.1	VGPAVAPEASVDSDQHIEDAATEDG	S	49
2740	LOC_Os06g19660.1	ADGSVSTLLKHPSGAGDENTVSEAP	S	838
2742	LOC_Os06g19660.1	TDKYSVEDSQSRSADGSVSTLLKHP	S	825
2743	LOC_Os06g19660.1	GASGQGSFGRKDSFGKEEPRGSQGD	S	704
2747	LOC_Os06g20370.1	QKLQLIWDEVGESDEDRDKVLYQLD	S	24
2750	LOC_Os06g20390.1	TNSHLTDMGRSQSFGSGQHGNEASS	S	182
2758	LOC_Os06g22700.1	GWGSDPKGYPARSPPDHAGRYADPV	S	171
2759	LOC_Os06g22700.1	GGGRGVARFRDGSPPYGRGGRSYGR	S	44
2760	LOC_Os06g22820.1	LQVPESKDEDDDSTEEWANFRGGDS	S	443
2763	LOC_Os06g23460.1	RSSKRGYHYDQDSPPPRSKPRFDRR	S	17
2764	LOC_Os06g23530.1	FLRGQGRSTIDVSPVRISTNPDGSL	S	327
2771	LOC_Os06g29400.1	KEEIANASADVPSDNVEELGLKYEK	S	837
2772	LOC_Os06g29400.1	VQRPVDLYKAIFSDDSDDDMAEPLA	S	730
2773	LOC_Os06g29400.1	PVDLYKAIFSDDSDDDMAEPLANQP	S	733
2775	LOC_Os06g29430.1	SNSNRSVKKSSSSNSQNTEGWISSQ	S	1125
2779	LOC_Os06g29430.1	RCRDCHRRKDAESDYYDEDEYSGRQ	S	243
2780	LOC_Os06g30020.1	YSRYHSYDYGPASPSHGRSHPDRGW	S	477
2783	LOC_Os06g30750.1	RSRGFRLLGEDTSVHKALGGGKTAD	S	70
2784	LOC_Os06g30750.1	PADTAAASPEKVSSPPPEPAPAVRS	S	47
2789	LOC_Os06g33520.1	DLKTDSFHLGLSSGDEGEASTRGPA	S	89
2791	LOC_Os06g33520.1	SDLKTDSFHLGLSSGDEGEASTRGP	S	88
2792	LOC_Os06g33520.1	RGYDFDDEPGFQSPKGQRGQGGRYS	S	238
2795	LOC_Os06g34420.1	FSEPPKVNLDLESSASKHRKKRNVN	S	484
2796	LOC_Os06g34440.1	SRQKQEVKYNEDSDGDDTDCHGNGD	S	367
2798	LOC_Os06g34440.1	HGNGDDGFVSSPSLKRLRKGGLFHG	S	387
2802	LOC_Os06g34710.1	EGERGGEVVLEVSDMDEEDGEDDTD	S	271
2808	LOC_Os06g36360.1	YDSEPGRSVKRASISPSISPVHQKT	S	552
2812	LOC_Os06g36940.1	EGQREDEDWIGESEDDKDSLSRGSS	S	270
2816	LOC_Os06g38960.1	PCRPKPSLDVSKSVCGLDLSSVKDN	S	60
2818	LOC_Os06g39480.1	DPQTLSPQDKFESKLQSPKKDAGSI	S	212
2819	LOC_Os06g39600.1	LTTPRPRVQRSISAEGLRELQSFLD	S	351
2827	LOC_Os06g40650.1	PPPMRNAYSRSTSFDQHSGVYSRSS	S	351
2833	LOC_Os06g40950.1	DDAEKEEGFDMDSESEEDTKSKPES	S	63
2837	LOC_Os06g42200.1	GRASGSRDRGPGSSRPADAREKRKQ	S	342
2839	LOC_Os06g42490.1	PFCGSPDINSRRSDSPDLEARRQPE	S	438
2840	LOC_Os06g42490.1	CGSPDINSRRSDSPDLEARRQPEPP	S	440
2843	LOC_Os06g42790.1	GLAKVDKDYAGFSPILIKSAFTDKK	S	332
2844	LOC_Os06g42810.1	VRTLSDLGGGKDSAGSEDSEDDEYK	S	172
2847	LOC_Os06g43170.1	DDDDGDLKKRSMSFIGRSTPQHHSK	S	494

2848	LOC Os06g43560.1	DHDSNPGHARKDSAESIGSDLSSLR	S	301
2852	LOC Os06g43790.1	CGAPEKSLDRNMSISASLVSDKRMM	S	1478
2853	LOC Os06g43790.1	EPRKTRKIVELSSFEKRDREDDNGF	S	1575
2854	LOC Os06g43840.1	KRINDRCFAGKPSEQDKMRNDFWNE	S	877
2856	LOC Os06g43840.1	QNSPNNQLTGEGSLSGNARHREDGL	S	470
2857	LOC Os06g43840.1	SPNNQLTGEGSLSGNARHREDGLTR	S	472
2862	LOC Os06g44970.1	GGGELEIEDGLKSPAAGLAAKFPVS	S	425
2863	LOC Os06g44970.1	DVGASIASFRVDSDVVSLNGREALQ	S	188
2864	LOC Os06g44970.1	SIASFRVDSDVVSLNGREALQADAE	S	192
2866	LOC Os06g45560.1	TKMSNIDVAIVGSLGGSYLRSYNYK	S	794
2869	LOC Os06g45910.1	DPPQLSELIPENSPVGKPQDEIAEE	S	180
2870	LOC Os06g45990.1	AVTLAAVVSKNASFREESNFLDDLK	S	46
2875	LOC Os06g46890.1	AAKEDPYNAYEMSPSDQRQNHETTR	S	268
2876	LOC Os06g46890.1	KEDPYNAYEMSPSDQRQNHETTRKR	S	270
2879	LOC Os06g47320.1	FLVNGVAFKKTFSYAGFEQQPKKFL	S	224
2880	LOC Os06g47330.1	KSSKKIPLNAVTSVIDGLKKLYIEK	S	158
2881	LOC Os06g48350.1	GSDEDHSNSPTRSHDGDNVAADEDD	S	221
2882	LOC Os06g48530.1	GGPPQPAATRKRSRSPPPPPPPPSL	S	51
2883	LOC Os06g48530.1	PPQPAATRKRSRSPPPPPPPPSLPP	S	53
2884	LOC Os06g48530.1	DSDKRRKHGRGSSKETDPLSGAPVA	S	313
2886	LOC Os06g48530.1	DGSRYGRGDRSPSLDGADDQMFDAF	S	601
2887	LOC Os06g48530.1	PRARRPPPPPPDSPEGRSPPLPPPP	S	25
2889	LOC Os06g48640.1	TLARDIEVMEPKSPEDIYKVHLIDG	S	352
2892	LOC Os06g49740.1	GGDDDAEQEDAGSDDGYPQMTEKQK	S	118
2893	LOC Os06g49760.1	AQELGGNMGRAGSPGFKWHLSNVQT	S	135
2894	LOC Os06g49800.1	QASDTEKIHGQASDTEISSELTTAA	S	431
2895	LOC Os06g49800.1	LGVPSPSVSRGISFDENTLTDPVEL	S	238
2898	LOC Os06g50840.1	PYGARGTAPRSNSPLFDDYGRSIGS	S	145
2900	LOC Os06g50890.1	YGGGRARRERSRSLPYSPYRMPERG	S	249
2901	LOC Os06g50890.1	RARRERSRSLPYSPYRMPERGYGRQ	S	253
2903	LOC Os06g50890.1	MAETPERRRYSGSPSPYRGNPKSRS	S	13
2905	LOC Os06g50890.1	PDPRSQARSRSRSREREPDAVNHGN	S	53
2906	LOC Os06g50890.1	RDYYDGRGGRGYSPHRSPPYGGGRA	S	230
2907	LOC Os06g50890.1	DGRGGRGYSPHRSPPYGGGRARRER	S	234
2913	LOC Os06g51250.1	RTDAFARFDSMKSTDYNSRGYSFDE	S	1148
2916	LOC Os06g51250.1	DQGSNDTFGRFDSFRSNADQGGGNS	S	1109
2917	LOC Os06g51250.1	SNDTFGRFDSFRSNADQGGGNSFTR	S	1112
2919	LOC Os06g51250.1	RSLFDSGPSRAESPTASSIYGKEQR	S	1021
2922	LOC Os06g51270.1	MDEQDQLGGLLDSVHGGNRKHEGMT	S	893
2923	LOC Os06g51400.1	LSDSDAYFPSIASDSESEGVRSKTP	S	132
2924	LOC Os06g51400.1	DSDAYFPSIASDSESEGVRSKTPMG	S	134
2925	LOC Os06g51400.1	DAYFPSIASDSESEGVRSKTPMGAN	S	136
2926	LOC Os07g01110.1	LGSGSTRILLIVSLLLCLRQQAVVD	S	17

2927	LOC Os07g01320.1	STSRDRDVDDADSDLEEEIQDVPRG	S	84
2928	LOC Os07g01550.1	GNMLHKDHDHHFSEGAECDIIDCDH	S	126
2933	LOC Os07g01880.1	GRRGVVASESEGSEDDYYAGRGHED	S	177
2934	LOC Os07g01880.1	VASGRRGVVASESEGSEDDYYAGRG	S	174
2935	LOC Os07g01880.1	KYTQPARQRRQLSPGFLEDALDEDE	S	496
2941	LOC Os07g02230.1	RILIENDDGSCGSETKNVVYHEILD	S	1399
2942	LOC Os07g03070.1	EAPRDEQRDPPPSPPNPSEEAGAGE	S	21
2946	LOC Os07g03180.1	RLTCPEKCFKSFSFKDKHGGGGGGG	S	109
2947	LOC Os07g03230.1	DADIEDPEKMVMSESEEEGDDDEEG	S	212
2950	LOC Os07g04240.1	DGKIYQRAFGGQSLDFGKGGQAYRC	S	161
2952	LOC Os07g04530.1	HANVPSWQASGSSPSKAVAPVQMSS	S	1504
2953	LOC Os07g04530.1	TTSDQQLMAVSKSVDGLESVGDSRN	S	1298
2955	LOC Os07g04530.1	ERRSAVAQQQPPSPTPAKVSINQRN	S	1479
2956	LOC Os07g04530.1	DLQDDAEELFWGSPIQVESSSKSAD	S	1185
2957	LOC Os07g04530.1	LGLESSLVLPAKSFSEDYDTNKGEW	S	1212
2958	LOC Os07g04530.1	EFPTLSSQTRSSSIKDQSPMNRQKS	S	1558
2959	LOC Os07g04530.1	TSQQPRQETLVGSDQGGAKHDLQDD	S	1165
2960	LOC Os07g05050.1	SRGFGDFDGFGSSPNRGGRSRDAGS	S	512
2963	LOC Os07g05190.1	GFSPSKGSRFSWSPDSGEAYTQEGL	S	740
2964	LOC Os07g05190.1	ELLTGRCAGDVVSGSEGGVDLTDWV	S	993
2966	LOC Os07g05190.1	SQEEKLSGVGGFSPSKGSRFSWSPD	S	730
2970	LOC Os07g05610.1	AAESNAREIGGSSMNGGEEVLNAET	S	664
2971	LOC Os07g05610.1	QPTGSELGGFAASFESRGPSSRKRN	S	476
2974	LOC Os07g05610.1	GIGHESNLQHSLSRNARVYSGIDLS	S	808
2976	LOC Os07g05610.1	RGFNLFDVNQPSSSGAGPSRNLSFD	S	549
2977	LOC Os07g05610.1	GFNLFDVNQPSSSGAGPSRNLSFDL	S	550
2980	LOC Os07g05610.1	MGRDLMIGQPTGSELGGFAASFESR	S	468
2984	LOC Os07g06130.1	VRDNLQGSAFLGSSRDLMSTKNEMS	S	750
2985	LOC Os07g06130.1	RDNLQGSAFLGSSRDLMSTKNEMSN	S	751
2987	LOC Os07g06740.1	SSTAPERVTIADSDLSSSTPNKGGN	S	65
2989	LOC Os07g07000.1	LTDSFELPRGGSSRDGDIEMGMQAD	S	17
2990	LOC Os07g07040.1	AKIVDKEEVKLDSDDGAKSAVKSKD	S	66
2995	LOC Os07g08190.1	NRYGRNGPYGARSPVRRYRGSPRAS	S	497
2996	LOC Os07g08190.1	DKGKQTATLDNISNEGSKPSNTDGN	S	366
2998	LOC Os07g08190.1	AGIRDDPGARARSSPIRADASLTKV	S	392
3000	LOC Os07g08190.1	QQYAFARRYRTPSPERSPVRSRYND	S	463
3001	LOC Os07g08190.1	FARRYRTPSPERSPVRSRYNDGRND	S	467
3002	LOC Os07g08330.1	KAAGKAWYKTMISDSDYAEFDNFSK	S	387
3003	LOC Os07g08330.1	RKEKLDSKRTKLSPEEAAKVKAAGK	S	367
3008	LOC Os07g09010.1	ITVLEMSKETERSPRPSSPAPAETS	S	328
3009	LOC Os07g09010.1	MSKETERSPRPSSPAPAETSWVKGM	S	333
3010	LOC Os07g09010.1	EMSKETERSPRPSSPAPAETSWVKG	S	332
3011	LOC Os07g09340.1	PAATAAVFRDMTSYNDLNQLAEEAR	S	907

3012	LOC_Os07g09384.1	PPPPEPTLARIESTESFSLPLHKVD	S	232
3013	LOC_Os07g09384.1	PEPTLARIESTESFSLPLHKVDGRA	S	235
3015	LOC_Os07g10350.1	KSYIGPDSTGDTSDDEDDEIVPEEI	S	514
3016	LOC_Os07g10350.1	ASVLPLQVSLDESEGSDQENDNKGQ	S	561
3018	LOC_Os07g10350.1	PESRASVLPLQVSLDESEGSDQEND	S	557
3019	LOC_Os07g10460.1	NDLLFKRVLVRRSYDEEEGDDIDHK	S	260
3020	LOC_Os07g10600.1	GGAPVIDAAMSRSGAMDLASGLGGK	S	53
3026	LOC_Os07g12250.1	YPGKGIRFIRADSQVFLFSNSKCKR	S	28
3027	LOC_Os07g12250.1	IRADSQVFLFSNSKCKRYFHNRLKP	S	36
3028	LOC_Os07g12250.1	RFIRADSQVFLFSNSKCKRYFHNRL	S	34
3030	LOC_Os07g12910.1	AVVDDGYDEEEHSETLCGTCGGRYN	S	186
3031	LOC_Os07g13240.1	DFTMPSETTIKASNVAPESEAKVED	S	47
3032	LOC_Os07g13340.1	VKANNGGAKPKASNEANTTRMVNQN	S	1072
3033	LOC_Os07g15490.1	DAGTCKECYEEASETEEELKREIDD	S	26
3034	LOC_Os07g18300.1	EKCCDAKVRRTFSKIDLKLIERLYL	S	808
3037	LOC_Os07g22024.1	NSKHGEEEDIIGSPEDEEFDHPSLR	S	464
3038	LOC_Os07g24020.1	GKRELQPAYKDLSGDEVHSMVKDIS	S	397
3040	LOC_Os07g24020.1	SGDEVHSMVKDISNEFGKKRKRSKT	S	409
3043	LOC_Os07g25460.1	EKKQLEAALKMDSPDGAGGEGQSDT	S	353
3048	LOC_Os07g25680.1	QEGLKSSLQPSDSAKSLETYTASKA	S	506
3052	LOC_Os07g25680.1	IPREQAGSLNRLSKSDDSLNSQFLI	S	582
3053	LOC_Os07g25810.1	GGGIGKGGGLGGSFGKGGGLGGGFG	S	334
3055	LOC_Os07g26440.1	SDSKESEVLDEGSDGSSSERGSSHG	S	366
3057	LOC_Os07g26440.1	ESEVLDEGSDGSSSERGSSHGHKSH	S	370
3058	LOC_Os07g28430.1	PVQLVVEDDEPDSTSGSKPASGKAT	S	230
3059	LOC_Os07g28800.1	NFRSRGGFEFCTSDEDEPETVFRNA	S	144
3062	LOC_Os07g29360.1	PGHLLAAAGLEDSDAEEDDMGHPQS	S	1024
3063	LOC_Os07g29450.1	RPLDDSSLYSSPSGKIIQPGSSDFH	S	32
3065	LOC_Os07g30300.1	WRQKATNFFSSSSFKLKQAGQSAGD	S	21
3068	LOC_Os07g30820.1	YGYGFRGSPMPVSSPWSGGALVENN	S	178
3071	LOC_Os07g30820.1	NRGACGAPRPSPSLSPRVPPPPAAG	S	70
3072	LOC_Os07g30820.1	GACGAPRPSPSLSPRVPPPPAAGYD	S	72
3073	LOC_Os07g30820.1	VCNRGACGAPRPSPSLSPRVPPPPA	S	68
3074	LOC_Os07g31450.1	LKVKKHLHPMELSPKKYKNKKQHNH	S	198
3075	LOC_Os07g31450.1	SAVDDMDTGSCRSPVRDTPDPDNQK	S	2089
3076	LOC_Os07g31450.1	RETFATIPNEALSEDSDEEDEPKRE	S	1268
3078	LOC_Os07g31840.1	KLAAAREAPYAKSRTQFTRDMQMAK	S	604
3079	LOC_Os07g32350.1	DEDETRMLDHDDSDSEMHTSKRKKS	S	404
3080	LOC_Os07g32350.1	DETRMLDHDDSDSEMHTSKRKKSGE	S	406
3081	LOC_Os07g32400.1	TIKVERATRDVVSPSSQGTVRKAAG	S	322
3082	LOC_Os07g32430.1	MLAAASPDARLVSPWLGGNTPRYAA	S	275
3084	LOC_Os07g32530.1	RSLESDQYDGTDSEEEVASASSRAL	S	729
3086	LOC_Os07g32530.1	AAASGSRGGCSDSDDNYEEAEMLRQ	S	67

3088	LOC_Os07g33630.1	RRGGGPMLGFDSSPGRSSMKYNGFH	S	593
3089	LOC_Os07g34740.1	EERLALLLQGDKSEEEQTDGEDMEI	S	399
3091	LOC_Os07g35580.1	GSFVGTLPSSGRSAPMSPNEVSITE	S	680
3092	LOC_Os07g35580.1	GTLPSSGRSAPMSPNEVSITELEPR	S	684
3093	LOC_Os07g35580.1	GGGDDGGGTGSYSGSFVGTLPSSGR	S	667
3094	LOC_Os07g36200.1	GIDALASDLDKTSPVGRSKSEFAKG	S	381
3095	LOC_Os07g36200.1	GIERSVAPVRPASHEGRVGEARGLE	S	225
3099	LOC_Os07g36600.1	PRPPTPASLQPESPGVFFTAAAAAA	S	37
3102	LOC_Os07g36940.1	SNKNATKDTRNLSQEPQSASSAEDL	S	646
3108	LOC_Os07g36940.1	VGPVAPQMSRSGSDADRWQQKGIFP	S	1142
3109	LOC_Os07g36940.1	NLAGKSYVVDHPSPGRGADRPASRG	S	1065
3112	LOC_Os07g36940.1	DFIGASESLDSSSIADHELPDESSE	S	962
3114	LOC_Os07g36940.1	PEEKVDFIGASESLDSSSIADHELP	S	957
3115	LOC_Os07g37140.1	RRRRRKAGDRGDSPPPLPDHLRCRR	S	282
3117	LOC_Os07g37140.1	VTRDLPNGLMRISPGSSEPAASLPS	S	377
3121	LOC_Os07g37610.1	DLNSQPEREDEQSPKSDATRLLRDN	S	941
3122	LOC_Os07g37650.1	NGAASAAANKSTSPTSSSLPSQSGK	S	449
3125	LOC_Os07g37750.1	QADEKITLGDALSLNRATEASPASV	S	216
3128	LOC_Os07g38090.1	AAHWLQGSRLRSSFNARDAAVDDLG	S	404
3129	LOC_Os07g38090.1	RHVPHNSGAGLLSPRASSSIDMTAA	S	349
3132	LOC_Os07g38160.1	GLVVKVPEFPNDSDDELEEEKETEV	S	562
3134	LOC_Os07g38970.1	KALREAGVTVVESPAKIGSTMFEIF	S	312
3135	LOC_Os07g38970.1	PGRRMGHAGAIVSGGKGTAQDKIKA	S	289
3139	LOC_Os07g39470.1	AGCMRHDSPSSQSFTTRSGSPLSQE	S	90
3143	LOC_Os07g39560.1	GSFYNGSSNRLGSPIGYVGLNDDSG	S	292
3144	LOC_Os07g39620.1	AKMKLPLDLDNGSDKSAAVSGAGAA	S	392
3146	LOC_Os07g39620.1	RTVQSSASPAANSPGAMSESAVDKP	S	334
3149	LOC_Os07g39620.1	AVSSAEQKEKAASDAADAEVESSRT	S	311
3150	LOC_Os07g40270.1	RFVLGSLEGFFPSDHTSSPGNKQII	S	232
3151	LOC_Os07g41180.1	AKVTDNVEKAVSSPVKPTNAADTTS	S	295
3157	LOC_Os07g41190.1	ADLDRKAPLPGSSDNESDEEGRVDD	S	207
3159	LOC_Os07g41190.1	EDDLPVYDEGLSSEDEGVDMVGRER	S	127
3161	LOC_Os07g41694.1	EAALQAAGEGSSSPARSLTLDGAVK	S	22
3162	LOC_Os07g41694.1	SQGAKRKRDDDPSGSGDDDEDDDGV	S	455
3167	LOC_Os07g42400.1	LVVRQIGFLRSISLLPADYKNYLRS	S	273
3168	LOC_Os07g42400.1	ILTELQSGEAELSDDSVMTPTTKAT	S	246
3170	LOC_Os07g42410.1	PRTQPKSFIRSKSIIEESKNISKDQ	S	984
3171	LOC_Os07g42410.1	RSNAEMRAKFSRSSSVPDSTYISRC	S	849
3175	LOC_Os07g42410.1	IHQGTNLSRQSSSAEQELSLHGGKV	S	667
3176	LOC_Os07g43030.1	FKIEPDGLKKFDSFSRWMSSELPEV	S	369
3177	LOC_Os07g43030.1	YTDGIRYPLLKQSSLDLFKIEPDGL	S	352
3182	LOC_Os07g43316.1	QDTDMSLKKKEGSPDSIYPEKLNLD	S	195
3184	LOC_Os07g43316.1	EKLNLDRSSGDESMEEDAMETKHMD	S	214

3185	LOC_Os07g43316.1	DSIYPEKLNLDRSSGDESMEEDAME	S	209
3187	LOC_Os07g43316.1	LEGKSEVTLEHVSSGDESMEEDVME	S	246
3189	LOC_Os07g43316.1	EVTLEHVSSGDESMEEDVMETKHVD	S	251
3193	LOC_Os07g43316.1	QDIVKPSSSNPSSVGDDLQTPDDDK	S	161
3194	LOC_Os07g43316.1	LESKQDIVKPSSSNPSSVGDDLQTP	S	157
3196	LOC_Os07g43316.1	NTKVEHVSSGDESMEEDVMETKHVD	S	277
3197	LOC_Os07g43316.1	HVDSNTKVEHVSSGDESMEEDVMET	S	273
3198	LOC_Os07g43316.1	DMSLKKKEGSPDSIYPEKLNLDRSS	S	198
3199	LOC_Os07g43316.1	EMITESLILKKDSNENDLMYEKDQK	S	71
3200	LOC_Os07g43350.1	RFHEEPVAFEVYSPDRLVGELIFLD	S	754
3201	LOC_Os07g43350.1	AMEPSPRVVLPTSPHFIDSRFHEEP	S	735
3203	LOC_Os07g44190.1	ASVPVKKIKVEESAEEVEGEKSEKK	S	507
3208	LOC_Os07g46180.1	RPTSSMDHNVTKSPGSNIPAVESGR	S	172
3212	LOC_Os07g46590.1	VVEPEAREEGEISESEAETKYRQDK	S	762
3214	LOC_Os07g47030.1	KLLSYFDGEGDISPEIMDNQNRWPV	S	601
3215	LOC_Os07g47100.1	RLLLPASGHPVTSEPSSPKSLHSPL	S	454
3216	LOC_Os07g47100.1	PASGHPVTSEPSSPKSLHSPLLTSM	S	458
3217	LOC_Os07g47100.1	LPASGHPVTSEPSSPKSLHSPLLTS	S	457
3219	LOC_Os07g47350.1	SLTRSSRSETLRSLQSIYEQESGSL	S	707
3220	LOC_Os07g47350.1	RSSRSETLRSLQSIYEQESGSLSRR	S	710
3222	LOC_Os07g47360.1	VAKTSPIESVSSSPPRISNNDKVSH	S	1097
3223	LOC_Os07g47840.1	RATDGGSAADRLSDVPGKVAEDTAD	S	190
3226	LOC_Os07g48410.1	HQMPMFDGGECRSPGGGDGGLFSYN	S	191
3227	LOC_Os07g48602.1	TVQLGKITETVQSPVKAKPEVAFQP	S	119
3229	LOC_Os07g49040.1	IPSDYRLTSPQLSPKRNQSKFKSLL	S	310
3231	LOC_Os07g49270.1	PLHTGREGKPIISPASTKRVGPLVR	S	94
3237	LOC_Os07g49380.1	WDPRKKGATDSDSEVDFDRKRVSKG	S	264
3238	LOC_Os07g49460.1	MPSLELSLKRSRSTGDGANAIQEEQ	S	416
3240	LOC_Os08g01054.1	SDEQWRMTSGNFSPDDQRNTWLPGA	S	1349
3242	LOC_Os08g01100.1	AELKAEYHNGERSDENNVGGNAGEQ	S	159
3247	LOC_Os08g01900.1	SERGFPRIISSSSMDTRSDLSAIEN	S	527
3249	LOC_Os08g01930.1	SAKVDEPVATENSDAAPAEASVDAV	S	100
3253	LOC_Os08g02410.1	KRTPPSWLKTAASDVEEMIMKAAKK	S	31
3254	LOC_Os08g02690.1	QNAETLSLASPRSPTGGSTSALLQQ	S	35
3255	LOC_Os08g02690.1	LSLASPRSPTGGSTSALLQQYEQQR	S	40
3257	LOC_Os08g02690.1	DACLDRNDPNYDSDEEPYELVEAPV	S	118
3260	LOC_Os08g03390.1	EKDGEEGDENVASEEEDEEDGLKID	S	193
3265	LOC_Os08g03840.1	SDEPCAKDDAKQSDVAPLKEEENWE	S	106
3267	LOC_Os08g05540.1	ETRVRLEDLGGESDEDEDDEVDKVM	S	135
3272	LOC_Os08g05840.1	KSKLKRPFVEDKSDDSEDDHKPIGL	S	115
3274	LOC_Os08g05840.1	KKPAVGSGTANDSDDDKPLSLKINS	S	149
3279	LOC_Os08g06020.1	PPSPVPEESEEGSPSRVSESENGDS	S	149
3280	LOC_Os08g06070.1	SSSMHENLKRQRSSVDDDLYDHPKH	S	423

3285	LOC_Os08g06140.1	APEESTAEKDMPSDGDDAEPTGITI	S	514
3287	LOC_Os08g06140.1	LRRRHAPTASSFSDGDDAESTGITI	S	539
3288	LOC_Os08g06344.1	NAGKKWTLDGEESDEEGNQEDGKKS	S	233
3292	LOC_Os08g08080.1	DISPPRRRTRHDSEEPQDLSPPRRK	S	237
3294	LOC_Os08g08080.1	LSRHDSKESQDISPPRRRTRHDSEE	S	227
3296	LOC_Os08g08080.1	RTRHDSEEPEDLSPPRRRTRHDSHE	S	295
3298	LOC_Os08g08220.1	SHRVLPPSFGRNSSANHSEFANGID	S	223
3301	LOC_Os08g08820.1	DAGGLVDEFESKSCSENVDGAGDGL	S	82
3302	LOC_Os08g08820.1	CSENVDGAGDGLSGDDQDPNQRPRK	S	95
3304	LOC_Os08g08830.1	SVLRGIEDELQFSPLENKRSATATD	S	167
3305	LOC_Os08g09350.1	IFVKGFDSSLEESKIRESLEGHFAD	S	425
3307	LOC_Os08g10580.1	FSKTRSGLVRINSTVESNEVSLSKT	S	41
3309	LOC_Os08g12760.1	ALQENRRQQQPASPELQKPAENKAL	S	507
3310	LOC_Os08g13350.1	SGPLGNQPSRNTSFGGAGSNSGPVS	S	74
3312	LOC_Os08g13350.1	HGEPGKKSSGPQSGGVTPMARQNSG	S	133
3313	LOC_Os08g13350.1	KMFDLHVEKSRKSGPLGNQPSRNTS	S	62
3315	LOC_Os08g13350.1	PRKVSGPLDPSVSMKMRATSFAHNP	S	185
3318	LOC_Os08g14340.1	SANMIISEGGGTSGYSKILPTVLSV	S	392
3320	LOC_Os08g14460.1	PAVVGNGRQEAGSPQSPLGGKTDVF	S	320
3329	LOC_Os08g17120.1	DMSAPAKEAIKLSDYERTLKKASSR	S	506
3333	LOC_Os08g20390.1	KASGVATTGSASSRRRARRAAAVTR	S	142
3334	LOC_Os08g20660.1	RRYAAADMSEDLSEGEKGENINESS	S	142
3335	LOC_Os08g20660.1	REKARRYAAADMSEDLSEGEKGENI	S	138
3336	LOC_Os08g20660.1	SPGDSLRDVHDISLNLKLSLDSEKS	S	719
3337	LOC_Os08g20660.1	DESTRGRMPRIGSTDAIEAWASQHK	S	170
3338	LOC_Os08g20660.1	LSRGVSANRKTESVENMEATTGNKW	S	761
3339	LOC_Os08g20660.1	KSDDATEVSEADSPGDSLRDVHDIS	S	707
3343	LOC_Os08g23200.1	SGSGSTMGGHARSILNAARVIPPND	S	61
3345	LOC_Os08g23360.1	VPERLSKGVSTLSLGERKQAFLLDM	S	542
3346	LOC_Os08g23440.1	KSMTGEQIQAPSSPRDGEDVAITIG	S	111
3347	LOC_Os08g23680.1	YNTILNRYGISKSDLSRSKDSSGDA	S	246
3348	LOC_Os08g23930.1	VNNAQRSHDRVPSPTTSARKRQKTS	S	143
3352	LOC_Os08g25080.1	NINDTPMDESQASDEARYSGKNGGG	S	155
3354	LOC_Os08g25080.1	SVVEDERQESGKSDDENKANDGEPS	S	119
3362	LOC_Os08g29124.1	SVDQGAICPRSDSPSVASLPKEETS	S	704
3366	LOC_Os08g30060.1	IEPLQKSLGKFRSENNAMRAQGAGL	S	162
3370	LOC_Os08g30060.1	VEKFMAQWCSSKSFREDYEKRILVS	S	355
3371	LOC_Os08g31470.1	TNVEERQFLIGMSPAREGQEANGDL	S	539
3372	LOC_Os08g32100.1	APAKKLGFGLIGSGKRTSVPSVFAE	S	684
3376	LOC_Os08g33320.1	ENSNNSRTTWRKSPDYYNYGLPGPR	S	1080
3377	LOC_Os08g33320.1	NGKHSRNGGGDVSPAALKCSWSTTC	S	877
3378	LOC_Os08g33380.1	LLQVQAMRSSEESSSDEDDEILSEL	S	62
3379	LOC_Os08g33380.1	LQVQAMRSSEESSSDEDDEILSELK	S	63

3380	LOC_Os08g33380.1	QVQAMRSSEESSSDEDDEILSELKE	S	64
3382	LOC_Os08g34060.1	QQADYDLMTRGVSWNRSQNENELNL	S	167
3384	LOC_Os08g34950.1	HQESSIGVRRVLSDGQFPVNADISD	S	1316
3385	LOC_Os08g35060.1	SVLSHGRNDDDDSADGDDGELFPIS	S	37
3386	LOC_Os08g35110.1	EEGSAAAAGRAASFHGADEVPKGLH	S	46
3391	LOC_Os08g36790.1	VDEVWRDLEREASPGAAAADGGGGG	S	73
3392	LOC_Os08g37104.1	TNVKQTPRRTIPSMNHGGDRPGKEN	S	182
3393	LOC_Os08g37280.1	KGRRTREGARQLSPTDGEDQASGKK	S	192
3394	LOC_Os08g37280.1	SKKVRKQKGLKESPASEHMGEDTKD	S	146
3395	LOC_Os08g37280.1	VRKQKGLKESPASEHMGEDTKDQHS	S	149
3396	LOC_Os08g37280.1	SEHMGEDTKDQHSAEQHQELGYTAT	S	161
3397	LOC_Os08g37280.1	KKELSHVNSPCDSTEEAGNLKAKTS	S	121
3399	LOC_Os08g37280.1	HEAVKKELSHVNSPCDSTEEAGNLK	S	117
3400	LOC_Os08g37444.1	QKKKGWFSSMFKSIAGNNVLEKSDI	S	326
3403	LOC_Os08g39140.1	SLWTEKTTEKEISDDEDEEEKKDAE	S	220
3405	LOC_Os08g39140.1	QALRDSSMAGYMSSKKTMEINPENA	S	602
3407	LOC_Os08g39390.1	PPLREALPLLSLSPTPARRGGVVDA	S	99
3409	LOC_Os08g39630.1	PGGSGGRLKGQLSFSSRQGSLMSQI	S	206
3414	LOC_Os08g40230.1	KPLPEVRDRDFESDFEEMHDTDEEL	S	114
3417	LOC_Os08g40820.1	EVKRRLKELRKNSFMVLIPEEECAE	S	95
3422	LOC_Os08g41400.1	GNVADDDGNAERSSSPPHVSEVQVG	S	140
3423	LOC_Os08g41400.1	VADDDGNAERSSSPPHVSEVQVGLS	S	142
3425	LOC_Os08g41710.1	SRVRKYLSPEDWSPTETKGRPWYGL	S	298
3426	LOC_Os08g42110.1	EVESSYEGEKPGSPISLSSGLRRRP	S	99
3431	LOC_Os08g42600.1	SPSPMSPPRFCASPTGNGYCSSKMA	S	404
3434	LOC_Os08g42600.1	EKCAADAGPYPESPRLSRFPNLPDM	S	890
3437	LOC_Os08g43060.1	EQQKAQDSSSIVSPIRKFEHQEQTN	S	518
3441	LOC_Os08g43090.1	GKSRHCRSLSVDSFIEKLNFDESPK	S	252
3442	LOC_Os08g43090.1	LPSPSGGLSRSGSGSLDGGAASLFG	S	279
3446	LOC_Os08g43410.1	FKGILHDVGPEHSSGGGGGMGGRHA	S	230
3449	LOC_Os08g44420.1	KDYEPTGETPVRSEPEVPSKDAIES	S	357
3450	LOC_Os08g44430.1	VGKTETLQSQADSFHRHGRELRRKM	S	197
3451	LOC_Os08g44480.1	WSKGKQKEKVNNSVLFDQATYDKLL	S	40
3452	LOC_Os08g44540.1	EEKTIAQEVNDDSDDDSGETESRTS	S	111
3455	LOC_Os09g01640.1	RGRSKHRRSTKRSDTEDDLEEERSK	S	237
3457	LOC_Os09g02810.1	CNDILATLEKCNSDAEDACDTIECV	S	663
3458	LOC_Os09g03600.1	IENCEKALVEKDSDGEDSVAPVVTK	S	722
3459	LOC_Os09g03600.1	KALVEKDSDGEDSVAPVVTKLSSGE	S	727
3460	LOC_Os09g03610.1	PSRWSSGGGGGGSGSPPHRFSRGGG	S	43
3462	LOC_Os09g03610.1	SRGGPAFGGPGFSPRSDAALVIRPT	S	313
3463	LOC_Os09g03610.1	GRHMPPDSWHPSSPRSAPHQFNNFG	S	345
3464	LOC_Os09g06560.1	RKSTYHDHSNGDSEDEDLILQMESR	S	499
3466	LOC_Os09g07294.1	TSYLSSSPSTPESPVRLSALSGARG	S	521

3469	LOC_Os09g07300.1	ALKRLSSKSSGNSFGSGSLIPKQLK	S	1524
3471	LOC_Os09g07300.1	KQVTLSELESVASHEEEALFGNLFA	S	548
3472	LOC_Os09g07300.1	GSLIPKQLKNSDSLVLRTNQESNST	S	1540
3474	LOC_Os09g07900.1	ATEVKELAGSSSSPSLKTGGVCNIT	S	3211
3477	LOC_Os09g09360.1	QDGLEQLLPMDDSPTSVKSFTDCQL	S	946
3478	LOC_Os09g10710.1	SLIDAIARLAEASDGESDEHNRVRR	S	366
3480	LOC_Os09g10710.1	TESLIKEVERVFSINNPDPLEVEKA	S	332
3482	LOC_Os09g11240.1	NLNLGLEDPDDDSDADLSAEEQRDS	S	128
3484	LOC_Os09g11250.1	QRFGFSSTSPELSDKEENQRKDQEN	S	81
3485	LOC_Os09g12240.1	SEIFDPTLTDRKSGEAELYQYLKIA	S	1151
3486	LOC_Os09g12270.1	KRRRLKKKHPADSSDDNNDDSSHRP	S	185
3492	LOC_Os09g13630.1	VDSGSPEDAQAGSEDDYVQRENGSS	S	411
3493	LOC_Os09g14490.1	YETCKAGAVRLISIVAMGGMGKTTL	S	666
3496	LOC_Os09g17730.1	ADTEASTESDNRSDGAAEAQAEDDS	S	539
3499	LOC_Os09g19640.1	IQHEIQYGGDNASASPRSQHASQST	S	93
3500	LOC_Os09g19640.1	HEIQYGGDNASASPRSQHASQSTQA	S	95
3501	LOC_Os09g19734.1	TVTVDELTPGLKSILSFAVPDQRSG	S	91
3502	LOC_Os09g19734.1	VDELTPGLKSILSFAVPDQRSGKFE	S	94
3504	LOC_Os09g19910.1	VGAVHDKMEHNSSPHGNNADLHKDS	S	56
3507	LOC_Os09g21000.1	RRRFGKQPRRVDSLDVEAMSVRGAH	S	108
3511	LOC_Os09g23740.1	SYSKAIPPVRIHSFDSIFRLHQRES	S	1185
3514	LOC_Os09g26540.1	SDLKGAINKDGSSDEEDFTEEGLSA	S	50
3519	LOC_Os09g27080.1	SFKRGEIELQIGSPRSPRGDGVGSP	S	64
3521	LOC_Os09g27850.1	AAAAAAAAGGSASEGSDAEASAEAA	S	25
3523	LOC_Os09g28220.1	KSMKRCIWNITPSPPRREGEDEDYG	S	146
3524	LOC_Os09g28220.1	KSRSSRRKRSKQSDSEDQAPSDADL	S	331
3526	LOC_Os09g28220.1	RSKQSDSEDQAPSDADLGVKEIDET	S	339
3529	LOC_Os09g28220.1	PSPYRSRRDRSPSPYRDRRRQWSPY	S	60
3532	LOC_Os09g28220.1	SPSPYRDRRRQWSPYHRDRGRDVER	S	70
3533	LOC_Os09g29070.1	APHSIADLSRSSSSSSEQQPSSTPA	S	48
3536	LOC_Os09g29080.1	RAKDSDDLRVLISEKPAEPAPAKRE	S	53
3538	LOC_Os09g29810.1	FFRDKNSHERSDSPSLTADSAAKNN	S	1641
3539	LOC_Os09g29890.1	TQMAVAVLDRSFSSEYPASSRTEGR	S	23
3541	LOC_Os09g30070.1	EFERFDVPIQFDSPCAETKTSEALC	S	1035
3542	LOC_Os09g30070.1	DSATLKQHLQCGSPDLSPTHSRNKD	S	376
3548	LOC_Os09g31486.1	EITIKSSGGLSESDIEKMVREAELH	S	557
3555	LOC_Os09g32430.1	LDQRKGHDEVSRSKDGTDTEDATID	S	145
3556	LOC_Os09g32540.1	KKQAADTVSRETSALQNIEERENVE	S	760
3557	LOC_Os09g32540.1	PKPGADDNKFWSSPARLPENNAEKK	S	936
3562	LOC_Os09g32820.1	RLSSPSAPSSPSSAKAAAAAAAHSN	S	39
3563	LOC_Os09g32820.1	LEALRLSSPSAPSSPSSAKAAAAAA	S	35
3564	LOC_Os09g32820.1	EALRLSSPSAPSSPSSAKAAAAAAA	S	36
3565	LOC_Os09g33450.1	VEGRAGMHSRRSSWGHRSGTSESMD	S	403

3568	LOC Os09g33450.1	LQQTRTPARGFMSPNMGKPVGDLEL	S	331
3569	LOC Os09g33510.1	SSSHQPSIHVRVSPNTSPPLLSSPK	S	486
3571	LOC Os09g33510.1	QPSIHVRVSPNTSPPLLSSPKIKEL	S	490
3572	LOC Os09g33510.1	NKEGTEAGHASRSPTEELLDRSKAE	S	289
3574	LOC Os09g33510.1	ASDNKKVKRGSFSGPIVPRSRSTEN	S	451
3580	LOC Os09g33600.1	ISPRDSLSLASTSARIDELDDCVEG	S	731
3582	LOC Os09g33600.1	LGKALLLQEQPTSPSDGSSRCFDRS	S	702
3584	LOC Os09g33870.1	PRRKSSDIYREESPPKRRSKDRVDS	S	223
3587	LOC Os09g33940.1	SNMVHQHEKSSGSPGEGVNLSVTDK	S	105
3590	LOC Os09g33970.1	AAEVEREEEEEASPPRVAARNPFDL	S	38
3591	LOC Os09g33980.1	LHPYQESEDDQESSEEQPAQDRREN	S	164
3592	LOC Os09g33980.1	HPYQESEDDQESSEEQPAQDRRENG	S	165
3601	LOC Os09g34060.1	AHGARPKHQHSLSMDESMSIKAEEL	S	138
3602	LOC Os09g34060.1	SMSIKAEELVGASPGTEGMSSAEAK	S	154
3605	LOC Os09g34180.1	EPHAAHTKAVLLSPGNSTALYDGDH	S	190
3607	LOC Os09g35710.1	NTESAQPVYTVDSPPSSSQPQGNLN	S	318
3608	LOC Os09g35760.1	RSLAMMGNGGSGSGGDGDSLGRGRE	S	80
3616	LOC Os09g36090.1	RGSFSKKDNNPVSPGKGEGRSPAQR	S	1122
3618	LOC Os09g36090.1	ADGVPEVNVVAISRDNSAENEAAAA	S	297
3620	LOC Os09g36090.1	RNTWGESSTHIESPLGRRPAFSSHG	S	1156
3621	LOC Os09g36090.1	GGDSGGRSWRSQSGGDGGSWRPKRG	S	1728
3622	LOC Os09g36090.1	QPSKSAVLSSTQSFSGRAGQGNDAA	S	1318
3623	LOC Os09g36090.1	SSSDVDGRPRNDSLLEEGTRAGEQP	S	1295
3626	LOC Os09g36270.1	ISSKQLHRGGVDSPSWRSGAQPDNI	S	166
3628	LOC Os09g36270.1	AATQAPAAMLPRSGSRPQLDLSGAA	S	50
3629	LOC Os09g36270.1	LPRSGSRPQLDLSGAAIHGNLEDRN	S	59
3630	LOC Os09g36270.1	TQAPAAMLPRSGSRPQLDLSGAAIH	S	52
3631	LOC Os09g36270.1	YWGLGRLMTKCKSFDELLELSQRGD	S	292
3633	LOC Os09g36360.1	STLRRRRPRRGWSLKQHEDDTAASP	S	53
3634	LOC Os09g36440.1	ETSEWRTFANESSDNDPVRVGGPTN	S	52
3639	LOC Os09g37230.1	EEVASRLLNRQQSIHPPPAFGSSTN	S	166
3642	LOC Os09g37520.1	TSRGEVTPPAPPSPPRVSTWDFLNP	S	357
3646	LOC Os09g37860.1	APKKKQELLEPSSDDSELEQQQERL	S	47
3647	LOC Os09g37860.1	KKQELLEPSSDDSELEQQQERLQEV	S	50
3648	LOC Os09g37860.1	VPEEAIEKTDPSSDDLQEQPIQSKI	S	571
3651	LOC Os09g38200.1	NLVFRGAGSASSSTSRQKQGEQWNE	S	406
3652	LOC Os09g38200.1	VFRGAGSASSSTSRQKQGEQWNEAV	S	408
3653	LOC Os09g38390.1	NPEHEPYLDDTNSGPLERNVNSDNG	S	322
3655	LOC Os09g38570.1	EGRAAESDVNVSSPRIKEANEDAEE	S	215
3657	LOC Os09g38570.1	GSIQEKLDKVENSGHVEGRAAESDV	S	199
3658	LOC Os09g38620.1	KPEEAMHINKSFSLSNGHAVYDIQH	S	300
3661	LOC Os09g39210.1	MADPASPATGELSPRTPQPFMEYVR	S	858
3667	LOC Os10g02630.1	STRDQQHAGRPRSEEPRRDERRADR	S	259

3669	LOC Os10g04580.1	KVDIAPRDGKRVSSGNLVDVARQWV	S	181
3670	LOC Os10g04580.1	VDIAPRDGKRVSSGNLVDVARQWVE	S	182
3671	LOC Os10g05690.1	QHGGGGEVEEESSEMGEKTAARTRL	S	41
3672	LOC Os10g06130.1	DDDGYRRHRREDSDDPEEEDPDERQ	S	37
3675	LOC Os10g07510.1	GSSSSRGGDDDGSDGGDLQALAREL	S	237
3676	LOC Os10g08550.1	KISGDSLKNVYKSFVSEYPIVSIED	S	325
3682	LOC Os10g10990.1	QKDQPKDEPVDNSPAKPTPSGHARG	S	389
3685	LOC Os10g19200.1	ARPRRHLPSPSPSGLPRAARATPWC	S	21
3688	LOC Os10g20600.1	TRKGANYVERQNSEISYYADDEDAN	S	254
3689	LOC Os10g20600.1	GANYVERQNSEISYYADDEDANRKK	S	257
3692	LOC Os10g21000.1	RTLQRDGAVASFSKEKTPPSSPTNR	S	50
3696	LOC Os10g22950.1	TQSSNLSDILKDSFKKSDSFTRWMS	S	379
3698	LOC Os10g24690.1	DPVLVRDFFRLLSLGSEASVPRDGV	S	33
3700	LOC Os10g25090.1	RNQSFDDDDDDFSNKPVAKKSNSAS	S	387
3703	LOC Os10g25110.1	SKGRGKSCHSDSSIEMNPGHMKNDN	S	82
3708	LOC Os10g26140.1	AEHVDRTLLNTSSDSDDIIDASGAS	S	481
3712	LOC Os10g28610.1	RDLGINKLEVSTSSVEPHEQWSNNV	S	1850
3713	LOC Os10g28710.1	IPKFSLRQNRSISLELPLFERSDEV	S	134
3714	LOC Os10g29400.1	VRSLDLDLIRPISPQKMAEGKIRWS	S	108
3715	LOC Os10g29560.1	MESAIPVQPKVRSPAVSAASKEEPK	S	389
3716	LOC Os10g29560.1	GDYYGEEEEDDESEEEQHFKRRPSS	S	861
3717	LOC Os10g30100.1	GHLVPDDMEMSRSPPEHSSAGAKQR	S	790
3719	LOC Os10g31520.1	DTATPYESAKSTSNNDEIADMAKSL	S	343
3728	LOC Os10g33230.1	KAANPSAGGRHASGGGGGGGGAGGG	S	102
3734	LOC Os10g34400.1	LREKLGMDSINISSSSFGAKHEDHN	S	341
3735	LOC Os10g34580.1	NLVEAKTSINLHSDQEKDEIVKPME	S	399
3737	LOC Os10g34820.1	SIDISGTSPKDRSNDLLEKHNNLLN	S	115
3739	LOC Os10g34820.1	NTQLDLETEGSSSPAATQKMKVKME	S	18
3740	LOC Os10g35150.1	RSFGEDNGMDYWSDDEDNEKMSRSW	S	124
3742	LOC Os10g35220.1	TRLPNRELPSSISPGADYMSRRSYL	S	597
3747	LOC Os10g35450.1	LVRSKSSDADNLSQCSSVYQCDRAG	S	122
3751	LOC Os10g35690.1	PFIYGRSVEVNLSEEEYGYDFVDRR	S	238
3755	LOC Os10g36350.1	ILDNGRSGSELSSGNVSLRSAEMGT	S	165
3757	LOC Os10g36350.1	RILDNGRSGSELSSGNVSLRSAEMG	S	164
3758	LOC Os10g36350.1	SLRSAEMGTELGSPLGRFVRNEDML	S	181
3761	LOC Os10g37480.1	LGKSSSTIENFFSGRTSTTAANSDG	S	403
3762	LOC Os10g37480.1	DSFSRNHLGKSSSTIENFFSGRTST	S	396
3763	LOC Os10g37480.1	EALSMSEYQRGVSAWNFDVEDLKAQ	S	345
3765	LOC Os10g37480.1	LAKVQEVPLSSLSRKSPQASPLKKS	S	583
3767	LOC Os10g37630.1	AWVAGGVLSRSLSILGLAQGGGGGG	S	126
3772	LOC Os10g38850.1	NLVRLECISEIDSRNDKAKNPSVDH	S	1615
3773	LOC Os10g38850.1	KPKASDDVLCHRSLGETIEQDAETR	S	266
3774	LOC Os10g38850.1	YSRIRIPDEEDSSSQKQTCRSSASQ	S	1720

3777	LOC Os10g39440.1	DHGGDDIEDSLQSPLISRQATSVEG	S	370
3778	LOC Os10g39440.1	DAESQREGEDYGSDHGGDDIEDSLQ	S	357
3779	LOC Os10g39440.1	YGSDHGGDDIEDSLQSPLISRQATS	S	367
3782	LOC Os10g39520.1	STPTPTPPERHDSFSFPRLPAHNLQ	S	494
3786	LOC Os10g40110.1	EELNGSSSSDDESKATSAPPARSIS	S	236
3788	LOC Os10g40590.1	YDTRQSFSDTAASPDLKMRCSNSGS	S	310
3789	LOC Os10g40780.1	VVDALSKLAIRPSSPESLQQLIEIA	S	1813
3792	LOC Os10g41030.1	SDRKSNYTLESVSERCIVSGRSSSP	S	213
3793	LOC Os10g41030.1	SSPHLVIPLPTISGEFIQSPKGKQL	S	434
3795	LOC Os10g41030.1	SAKSQGEPDLKNSSPHLVIPLPTIS	S	422
3796	LOC Os10g41030.1	PQKSRKRKKAPASPEQPIIAPLLKT	S	1198
3798	LOC Os10g41030.1	KHDHAIQNLEPSSSGKGLQLDVVHS	S	1513
3799	LOC Os10g41030.1	HDHAIQNLEPSSSGKGLQLDVVHSG	S	1514
3803	LOC Os10g41030.1	LSHGGDRTLVTVSSENKNGLEHGTG	S	733
3804	LOC Os10g41030.1	SHGGDRTLVTVSSENKNGLEHGTGE	S	734
3809	LOC Os10g41440.1	SGAVVTIQESLGSPDDITVEMKGTS	S	372
3812	LOC Os10g41960.1	RPYRMELRDGEASDDEEEYDTKEVE	S	689
3813	LOC Os10g42196.1	AVNSSVTLPRSYSLGNLANVKTPGP	S	704
3820	LOC Os10g42439.1	DIVPGSSVTEDASDSETLPRISWNY	S	1013
3821	LOC Os10g42439.1	APPPQPALVRLPSTAPALPSAPANP	S	2611
3823	LOC Os10g42724.1	VSRKMSAGVSNMSFKMKEFFQGQNM	S	35
3824	LOC Os10g42724.1	RTKDGTVGKRAGSGADELLQDLDDM	S	351
3826	LOC Os10g43040.1	KRKSDGIAAALRSPELKKAKVSLGH	S	239
3828	LOC Os11g01200.1	TVVIAAPAKKLGSFFSEVATESAHR	S	576
3830	LOC Os11g01300.1	RRASTAGGSGGFSGGGGSNMLRFYT	S	51
3833	LOC Os11g01836.1	HEFRVGDKSHPLSDQIYKKLDELGL	S	535
3836	LOC Os11g02620.1	VSGLAHLECDVGSGKLRMHCQDWWV	S	198
3837	LOC Os11g02720.1	LRRPLKAVQLSPSICRKLDFDSVSP	S	368
3838	LOC Os11g03810.1	IVEKMVVLGGNNSFKFAEQAWHRSE	S	87
3839	LOC Os11g03890.1	IESNPYFAVTAGSPLDVSKRARMME	S	39
3840	LOC Os11g04190.1	RKSTSSRSEAACSHGTQSHSQSMQP	S	227
3841	LOC Os11g04190.1	DEEKGAAGGGGHSPQPVVGKRKDLH	S	293
3844	LOC Os11g04190.1	HMFVWSSSASPVSERAAAAAAGAVH	S	317
3848	LOC Os11g04190.1	LQSSRNPTPRGSSFNHAEFFNIVGN	S	264
3850	LOC Os11g04190.1	HSQSMQPRVSNLSGVEIYSLQSSRN	S	245
3851	LOC Os11g04190.1	GDAKGAQAYDEYSFGNKNEKDGPTL	S	351
3853	LOC Os11g04670.1	DIIQIPTVLREYSMTSVTEGIDAIA	S	550
3854	LOC Os11g04890.1	GSAGANGRLPKPSSGDDELVLTPAP	S	147
3855	LOC Os11g04890.1	SAGANGRLPKPSSGDDELVLTPAPR	S	148
3858	LOC Os11g06320.1	HQFSSHNDNLAHSPDHNSNVRKDLV	S	408
3861	LOC Os11g06440.1	SSSHFRSLSWSVSRNWSAARQLQAI	S	239
3862	LOC Os11g06440.1	SSSSSHFRSLSWSVSRNWSAARQLQ	S	237
3864	LOC Os11g06650.1	GDASNSIITDGNSPEKIQGKNQQSS	S	195

3866	LOC_Os11g06680.1	RHHPPTCSDDEFSDDENHEVVQEAG	S	817
3870	LOC_Os11g07440.1	IDKPRLTLERKRSFDEQSWSELSHR	S	37
3871	LOC_Os11g07440.1	LTLERKRSFDEQSWSELSHRQNDGF	S	42
3872	LOC_Os11g07500.1	MSFRDKQRVIYTSPLKALSNQKYRE	S	133
3873	LOC_Os11g07500.1	QRVIYTSPLKALSNQKYRELSQEFS	S	139
3875	LOC_Os11g08550.1	ETINKEKSSIMFSRNIKEVNKQIFM	S	1158
3877	LOC_Os11g09160.1	KSPNPPFLMRFLSGEVSRRGRCVSK	S	250
3878	LOC_Os11g09160.1	AHPSKNVIIKTESDEGGMVTAAITT	S	145
3879	LOC_Os11g09329.1	SESNRPAVNRQESLPDDDFINPTAI	S	452
3881	LOC_Os11g10060.1	NQLQQQQLLRQQSLNMRTPGKSAPY	S	316
3883	LOC_Os11g10710.1	ELPIDHALEKLPSYNNESINLESKL	S	320
3886	LOC_Os11g12810.1	QELGSREAAADLSELSEGEKDGKPD	S	136
3888	LOC_Os11g14220.1	LAALEKDYEEVGSEFDDGDEGDEGD	S	437
3892	LOC_Os11g16400.1	TVAAMDVRRMSSSLNMSSSSLRSSG	S	30
3894	LOC_Os11g17060.1	KPECPWSFGQRLSSIKPLINKVENI	S	79
3897	LOC_Os11g19810.1	EKEIYGKATNTGSCASFSDVDEDIP	S	843
3898	LOC_Os11g19810.1	IYGKATNTGSCASFSDVDEDIPEKA	S	846
3900	LOC_Os11g22180.1	LGTCRKATNSTGSDASPPPQRRQRL	S	110
3902	LOC_Os11g26130.1	NVEKFRPVSTKTSPAESEMMKIQVV	S	517
3904	LOC_Os11g28270.1	GAVTNWPLSRSASFIASPRWPGHSS	S	215
3907	LOC_Os11g28270.1	VPAHSEVSPDNVSGRSRRITHSDSQ	S	418
3911	LOC_Os11g31880.1	PPWDLLYRLNRHSLQRKKSGKSRLQ	S	305
3915	LOC_Os11g32900.1	PSAGPDGNCYPQSPKRLCTESRNSL	S	644
3916	LOC_Os11g32900.1	TKTIKNMLAAPSSPSSPANGGSIKI	S	369
3917	LOC_Os11g32900.1	IKNMLAAPSSPSSPANGGSIKIVQM	S	372
3918	LOC_Os11g32900.1	APSSPSSPANGGSIKIVQMTPVTSA	S	378
3921	LOC_Os11g32900.1	PKSSPFSNLPDMSPKKVSSSHNVYV	S	870
3926	LOC_Os11g34190.1	FIKQCVDKVFSGSDDDNTNNNAPEK	S	74
3930	LOC_Os11g34200.1	ALPRRRKALPNGSDPENEESRSSKM	S	113
3934	LOC_Os11g35050.1	SGKSPSSPVDVFSSDDEHRHDTESS	S	131
3935	LOC_Os11g35050.1	GKSPSSPVDVFSSDDEHRHDTESSA	S	132
3936	LOC_Os11g35050.1	NAILRRCSFGGNSMVSDISKCQLTG	S	594
3940	LOC_Os11g36340.1	KVGKKPALSQSASFPARGAAAKKAA	S	88
3941	LOC_Os11g36340.1	AAASVDGSVSCESPRSITNLAKLTE	S	314
3942	LOC_Os11g36480.1	APHDARKTSKRKSPLFEERRNSSHF	S	128
3944	LOC_Os11g37100.1	SPNGATSRDKTPSPQASERPISIDD	S	447
3945	LOC_Os11g37890.1	KAEGVDLSAYGSSKVVQTQAPVQLG	S	350
3947	LOC_Os11g37990.1	DVELYAKLDSAYSSDEEDVEDEDDE	S	754
3948	LOC_Os11g37990.1	VELYAKLDSAYSSDEEDVEDEDDEA	S	755
3949	LOC_Os11g38620.1	SKHQRAVLPRLRSVLDQELDRSAFE	S	436
3950	LOC_Os11g38870.1	SVSTERERSRAGSGSTPATSSGLTD	S	237
3953	LOC_Os11g39030.1	VAAPAMKPLAPRSPAAAAAAVVPSP	S	368
3957	LOC_Os11g40080.1	LPVQDTQLRKSKSISCDSSTMEANA	S	133

3958	LOC Os11g40080.1	WRIWPFSFKRTRSVNTVQPVSESTE	S	1097
3962	LOC Os11g40090.1	KREAIDATMAAASPKKKEKKHSKKQ	S	62
3963	LOC Os11g40140.1	DGVVLAVEKRVTSPLLEPSSVEKIM	S	56
3968	LOC Os11g41890.1	KRAVPKELSPGPSMRSPVGGFNYAV	S	191
3970	LOC Os11g41890.1	GSIYGDTTWRSGSSELDGTSPFGYG	S	436
3971	LOC Os11g41890.1	SIYGDTTWRSGSSELDGTSPFGYGL	S	437
3974	LOC Os11g41890.1	SLQYGRQLNPYYSGNSGRYNSNVSY	S	285
3977	LOC Os11g42040.1	CRMPIVVHELAVSISQKEEFGAICH	S	551
3981	LOC Os11g42420.1	ESKLFGSGKKRASLDASDAGFSSGR	S	103
3982	LOC Os11g42420.1	FGSGKKRASLDASDAGFSSGRQAVR	S	107
3983	LOC Os11g42790.1	FGGRGFVPFVPGSPTERSVPLLQGN	S	531
3985	LOC Os11g42930.1	LDSADRATVRASSMDSDLPDSATAK	S	166
3986	LOC Os11g42930.1	ATAKLPDVEEALSPRSERKRKIHLY	S	187
3989	LOC Os11g43590.1	SGRADDLAWRCSSDTFDLNGRAFEN	S	69
3990	LOC Os11g43610.1	RAAALREVFGDSSDSEADDLPVGGA	S	25
3991	LOC Os11g43610.1	AALREVFGDSSDSEADDLPVGGAGR	S	27
3992	LOC Os11g43970.1	PPKRPKAEAAPASPTASVPGRIEED	S	39
3995	LOC Os11g43970.1	DQLKDLSAAPPASAGENNQRLVRPM	S	265
3999	LOC Os11g47330.1	NIEFPLPFGRVLSSTEGFIHDLDEK	S	244
4003	LOC Os11g47830.1	ERSSVLGRSPRPSPPGRSHCHSHSR	S	294
4005	LOC Os11g47830.1	APDGERSWALGRSPQPSPPGRSHFH	S	391
4007	LOC Os11g47830.1	PQPSPPGRSHFHSHSRSRSPELRGR	S	404
4008	LOC Os11g47830.1	PSPPGRSHFHSHSRSRSPELRGRAR	S	406
4011	LOC Os12g02690.1	LETTGKDLIGSNSPRSCETPSSEPL	S	183
4015	LOC Os12g05110.1	AMTKIVGTLGPKSRSVDTISSCLKA	S	40
4018	LOC Os12g05420.1	PTYQRTLSGGLKSPRAADVPREAIL	S	376
4019	LOC Os12g06380.1	NESAEDIQCTQTSPESRVKHRSILE	S	513
4020	LOC Os12g06520.1	SGGGRGHHRRAHSETFLRLPDADLL	S	23
4022	LOC Os12g06520.1	GHKRSGSMDGVNSPFEGESALSGGL	S	130
4023	LOC Os12g06560.1	PTRPQHKQYSFVSDSEEDTEKGKLV	S	961
4026	LOC Os12g06560.1	CYRPPTLSDDDFSEDENHADQQPRK	S	884
4027	LOC Os12g06560.1	AIHNDKPPPLPVSTSFSEKNAKTEN	S	366
4029	LOC Os12g06560.1	DKPPPLPVSTSFSEKNAKTENSSQN	S	370
4031	LOC Os12g06640.1	NNLPSGSLMRSNSGQMHAPNPGKQG	S	16
4034	LOC Os12g06640.1	GNSSSSHGCSRRSDSHGISLDINKA	S	328
4035	LOC Os12g06640.1	SSSSHGCSRRSDSHGISLDINKAFP	S	330
4036	LOC Os12g06670.1	FLRIEEVDPTYDSDASEDGADDGDD	S	134
4041	LOC Os12g06810.1	AHEPLKLFQKGSSFLAGNLSFSRAR	S	379
4045	LOC Os12g06850.1	QNAQVSDFKYSPSPQRHNGLSSERS	S	218
4046	LOC Os12g06870.1	FNKTAISPTRSTSIENGIHRDDRAS	S	767
4048	LOC Os12g06890.1	KGPLAISPTRSTSIENGIQRDDRAS	S	745
4051	LOC Os12g06980.1	SLPVPEDISAPKSPEERAIHQDDSV	S	2334
4053	LOC Os12g07190.1	CDSRSEVSLLLASETGDGKSSIYPP	S	392

4055	LOC Os12g07260.1	AHKKDLDLTSDESSDEDTIDAIGLS	S	115
4056	LOC Os12g07260.1	HKKDLDLTSDESSDEDTIDAIGLSE	S	116
4057	LOC Os12g07260.1	PPEAHKKDLDLTSDESSDEDTIDAI	S	112
4059	LOC Os12g07300.1	PRLKEIDTAPLKSLDEDLDEAAREV	S	565
4060	LOC Os12g07530.1	YRDRLDDPYVERSREREIDRLYAGR	S	318
4062	LOC Os12g07530.1	SPGRDHRIRRTPSPYRDRRRIPDYH	S	133
4065	LOC Os12g07530.1	RQKHSNHSPRDASMEYVRRDPVNAY	S	443
4072	LOC Os12g09880.1	FSSSSGRLSAAGSTASGGSGGGWER	S	25
4075	LOC Os12g10700.1	LKMAGLLDSPPNSPERKNTIIEGGC	S	905
4078	LOC Os12g11600.1	RDAEGNEEEDSGSHPSAGQKRARGQ	S	63
4080	LOC Os12g13170.1	GKEKSPIKRSKGSLGSLNMITGKNS	S	153
4083	LOC Os12g13770.1	AGLTGGTRGLTGSAWPVGRSRAARE	S	602
4084	LOC Os12g14110.1	ELKSKQKLEKKLSFYTKVKDAVTSL	S	31
4089	LOC Os12g16350.1	TRTAVEASSDVDSDNEDGVAGGVLQ	S	156
4090	LOC Os12g17310.1	PAPEAQAHGTASSPVQKASAGKANE	S	588
4092	LOC Os12g18120.1	VDSLGFADKGSASNLENFKKYSYEI	S	198
4094	LOC Os12g18860.1	RESLDRIEPSRKSVDCFEPSRRSVD	S	284
4096	LOC Os12g19040.1	IRFESAGGESDKSPVKEIQTTFQWS	S	239
4098	LOC Os12g19040.1	PGHIRFESAGGESDKSPVKEIQTTF	S	236
4099	LOC Os12g19040.1	LPFVRHEADEEESDTSDDIVPVVVR	S	211
4100	LOC Os12g19040.1	VRHEADEEESDTSDDIVPVVVRPGH	S	214
4101	LOC Os12g19040.1	DCPSSNNRDVLPSPSSNQNNSSLPF	S	189
4103	LOC Os12g19040.1	PSSNNRDVLPSPSSNQNNSSLPFVR	S	191
4104	LOC Os12g19040.1	LLKDTDSVQPAVSTPLTETGIKGGS	S	446
4106	LOC Os12g21710.1	RGSSIGGAGEDDSDGEVDGAPRLVG	S	68
4107	LOC Os12g21710.1	ASAVRPARARGSSIGGAGEDDSDGE	S	59
4109	LOC Os12g21890.1	SPTGKNTFSRLTSGLGLRLSPKGPQ	S	114
4112	LOC Os12g21890.1	SARNDKDDVFSDSEAEDGSSKGRRE	S	545
4113	LOC Os12g21890.1	DDVFSDSEAEDGSSKGRREKVSRNV	S	551
4115	LOC Os12g22090.1	AKAIHQKKRKANSDPGESPLARHKP	S	449
4117	LOC Os12g24080.1	RSKIKHQHDHHHSPVRISVRRAYIL	S	636
4121	LOC Os12g24080.1	NMLRERFAHRYHSGSLFGMNSRGRR	S	51
4122	LOC Os12g24080.1	LRERFAHRYHSGSLFGMNSRGRRGE	S	53
4125	LOC Os12g29660.1	RLEAEREMLEDESEDEEEAKESKGG	S	696
4126	LOC Os12g31160.1	VSASMGVMVPLLSKLIESMEQPRFK	S	17
4127	LOC Os12g32986.1	WSANMERLMRAQSMGDMSSLDFMRS	S	691
4128	LOC Os12g32986.1	AEMNLQRDSPVSSEPIEAEVIEPEL	S	792
4129	LOC Os12g33090.1	DDEELGRLKRCSSFELRSGAANGNH	S	541
4130	LOC Os12g34330.1	DEIFLEKRNETKSLGSLGDQIADVT	S	322
4134	LOC Os12g34330.1	PSLEDVSPSHPNSDDEKLGAYVMKN	S	244
4137	LOC Os12g34500.1	LEAMVAQLERNNSALFGEKDELAKQ	S	189
4139	LOC Os12g35030.1	ITDETRDDEFDESPSRLDPLKFDDV	S	130
4140	LOC Os12g35030.1	GYRNRFKDLIPQSGSPPRRRAGSPP	S	93

4143	LOC Os12g35620.1	NDGNKGSGEENASEGVSDTLGNLKL	S	147
4144	LOC Os12g35620.1	LKLDDTPKNHDESSDANDEKNETIA	S	169
4145	LOC Os12g35620.1	KLDDTPKNHDESSDANDEKNETIAE	S	170
4146	LOC Os12g36180.1	LKDRNGSTKDRDSSPVQNFSRKNTT	S	301
4148	LOC Os12g36180.1	RSNQNHNHHRSSSNQAGSSSIDELE	S	426
4151	LOC Os12g37430.1	EHQRREIHLNAPSPDRAARRAAMEG	S	90
4155	LOC Os12g37720.1	LESNAELDGELDSDSETGKEKHDDE	S	749
4156	LOC Os12g37720.1	SNAELDGELDSDSETGKEKHDDESS	S	751
4158	LOC Os12g37720.1	LGRSSLLSSNSASVADDIDDLDTKK	S	848
4160	LOC Os12g37840.1	AQDIDVALARTQSAEILDDIVTRSR	S	627
4163	LOC Os12g37870.1	PLPFGRVLSPSESFIHELDEKTSSS	S	248
4164	LOC Os12g37890.1	AAVARVREEGEVSSGADDDEPLRAR	S	70
4165	LOC Os12g37890.1	AVARVREEGEVSSGADDDEPLRARF	S	71
4166	LOC Os12g38000.1	HTHHRKGPARFRSLDFGERNGYLKG	S	31
4167	LOC Os12g38430.1	SYSPAPRRRDDYSASPPRKDTHPTK	S	175
4169	LOC Os12g38440.1	ESEARNIQAEQQSGIVVELMQPRGV	S	109
4174	LOC Os12g38970.1	RDQEVVRDVFGESDEDEPAPYRDQQ	S	215
4177	LOC Os12g38970.1	DEEPRQTRKASSSPVEEERDQEVVR	S	197
4182	LOC Os12g39370.1	DSLLDENGQDSESDEHGLVKPRYGR	S	107
4183	LOC Os12g39980.1	AVLKRQRVPRSLSFTSDIFERSGVD	S	613
4184	LOC Os12g40010.1	EANIIADRLHRASDAAEASGYAFRD	S	692
4186	LOC Os12g40010.1	SWFAGRKSNKRMSPNLTSRISRSPL	S	551
4193	LOC Os12g41180.1	TTTQSVKGLSVDSSEAGDEVTSKAI	S	349
4195	LOC Os12g41200.1	SEASAERGRRSCSSEVSQLTVVKDL	S	1372
4196	LOC Os12g41920.1	LIKVNRKYRIAPSSSLSEGRSSKVV	S	187
4198	LOC Os12g42140.1	DDALYGVFAEGDSDYDSDDGRRRSR	S	54
4201	LOC Os12g42370.1	VDPAQPDMRHVLSDLESFDLATEGS	S	528
4202	LOC Os12g42550.1	SQPPSAAAPAKESSPVNNGQLPAGA	S	285
4203	LOC Os12g42550.1	QPPSAAAPAKESSPVNNGQLPAGAS	S	286
4204	LOC Os12g42600.1	KDELALTMSRSMSNGSIMGMDLDSS	S	248
4208	LOC Os12g44140.1	HKLKDGISSITSSETDNDDVNKSIK	S	260
4210	LOC Os12g44330.1	SADMFCDDIFGESPAGIRKLGKDDG	S	566
4212	LOC Os12g44330.1	HHRSPDPADPAASPKRRRRHHRRRR	S	28
4213	LOC Os12g44330.1	TGVSGLGEGTPKSERSADMFCDDIF	S	551
4215	LOC Os12g44340.1	LSKTNGFLSKRPSFQMRSSVSTTTT	S	389
4216	LOC Os12g44360.1	RPEIEADLQRSASLISQTLELPRTQ	S	963
4218	LOC Osm1g00400.1	LRNKRRSIKRNLSQRENLNSNIKSQ	S	44
4228	LOC Os01g06290.1	RSPADHERRDMSTAANGRSPSPRDY	T	296
4230	LOC Os01g08560.1	ETDDAPNEAASGTDVNMQEAKAPAD	T	529
4232	LOC Os01g12660.1	TSSFPKLKRNASTSSDMSSLASQGP	T	217
4236	LOC Os01g14440.1	DQERGDSPDAPSTAAAWLPGRAMAP	T	251
4237	LOC Os01g16100.1	MAAPQSHSHPAKTLRASPPPPSTAG	T	46
4243	LOC Os01g16870.1	EMSETSSSHGGHTSAGSAPVPELPR	T	880

4247	LOC Os01g20940.1	VCPICSKNVGLSTSSLKLHLQKAHK	T	868
4248	LOC Os01g23540.1	QCDKRSLDMVEHTPSPPATAELPKK	T	770
4249	LOC Os01g27730.1	PHEKLLSLYRVPTFTSVDDFLQKVA	T	522
4252	LOC Os01g32660.1	ETPVDKFLTASGTFKDGELRLNQRG	T	30
4253	LOC Os01g32720.1	NIDDISRKFSPGTDFEVNRQHFTAP	T	851
4257	LOC Os01g36860.1	GKVGKVDLDRSPTPQDKSGPYSPAY	T	711
4262	LOC Os01g38950.1	LQSSEAEPSRSRTPDASPGTFQRSA	T	412
4263	LOC Os01g40050.1	KSDFRELEHDSETDDYPDDENEEDF	T	284
4273	LOC Os01g49020.1	AFLAGMVTKGSLTLNKAAVIASSQC	T	49
4275	LOC Os01g49250.1	ADSWSVRSEYGSTLDDDQRYADAAE	T	69
4278	LOC Os01g50310.1	PAKSYATVAAEKTVPDGSVAEDEVT	T	144
4280	LOC Os01g53000.1	RPPRARRMQRTMTTPGTLAELDEER	T	50
4283	LOC Os01g53000.1	LAELDEERAGSVTSDVPSSLASDRL	T	67
4287	LOC Os01g55040.1	QRPRRRLERGTSTVSQLFKGQEDGA	T	1065
4288	LOC Os01g56100.1	IPSDSEKANLHITSDDDDKEVEDWL	T	352
4291	LOC Os01g57450.1	AAQAASAAAIHPTSPRYFFSSLAGT	T	42
4294	LOC Os01g62650.1	ELNMFRSGSAPPTIEGSLNAISGLL	T	65
4297	LOC Os01g67160.1	LGRAFTVPMKSYTHEIVTLWYRAPE	T	170
4298	LOC Os01g67250.1	QVNNVEGVTEQLTDNEKETAVVEKA	T	778
4303	LOC Os01g70020.1	RPAKGVSKPSQDTEEDDEPEVELES	T	334
4304	LOC Os01g70810.1	LFAMKDTIGKKETREISASFGVTVT	T	103
4309	LOC Os02g02360.1	RDALKESEQSIETSSAAPSIDDDKI	T	289
4310	LOC Os02g03030.1	NIARFINGINNHTPDGRKKQNLKCV	T	308
4311	LOC Os02g03060.1	LARAFGIPVRTFTHEVVTLWYRAPE	T	244
4314	LOC Os02g04270.1	AHESKDGDGAEVTKDGDDSDDSDGM	T	986
4317	LOC Os02g05310.1	PTPGQIASRGPMTPEQYQLLRWERD	T	356
4318	LOC Os02g05310.1	PERHDPFAAGEATPDPSVRTYADAM	T	139
4319	LOC Os02g05310.1	QPIRTPARKLLATPTPLGTPLYAIP	T	412
4320	LOC Os02g05310.1	ARKLLATPTPLGTPLYAIPEENRGQ	T	418
4323	LOC Os02g05310.1	ATPKLPGGLVTPTPKKQRSRWDETP	T	298
4324	LOC Os02g05310.1	ATPSVRRNRWDETPTPGRMADADAT	T	256
4325	LOC Os02g05310.1	PSVRRNRWDETPTPGRMADADATPA	T	258
4326	LOC Os02g05330.1	YLGVKVHACVGGTSVREDQRILASG	T	147
4327	LOC Os02g08190.1	KPSAAKSSAAAATPASIDAIDRHLR	T	53
4328	LOC Os02g08500.1	SRVHSSSADPFSTLVGESPQFPDLG	T	424
4331	LOC Os02g13840.1	QQVYTGVWLRHYTPVRERVPASQGE	T	468
4333	LOC Os02g15220.1	PNPSPSSSPLAQTLASIRRSIRRPE	T	70
4334	LOC Os02g17320.1	DRYFGTDDYQSSTPFFCDEKNRVRL	T	476
4337	LOC Os02g18660.1	EEEEEEEDSDTDTYAQGFQLRKVGG	T	74
4341	LOC Os02g20970.1	HSSPERGESESPTKRHLRKKAVHMV	T	624
4342	LOC Os02g21150.1	RTTSEVFLRAMRTTLPKVQQSGISL	T	154
4343	LOC Os02g26140.1	VQVAAAAASVSKTRRCTFSPSRLVS	T	320
4344	LOC Os02g28980.1	MISKLSKLEDSETEGGTTQAPSKKH	T	612

4345	LOC_Os02g33360.1	KYKVKVVADVPGTGSSMDGEVKQAP	T	88
4346	LOC_Os02g33770.1	MNKMLSEGSGGPTAEKSPSTPGMRD	T	325
4348	LOC_Os02g34270.1	PPPPSRSVAAATTTLLGGSHGSNGA	T	199
4349	LOC_Os02g34270.1	PPPSRSVAAATTTLLGGSHGSNGAD	T	200
4351	LOC_Os02g34680.1	PRNVLYEDDSEETEEEGDNVGDGAR	T	315
4367	LOC_Os02g45070.1	TSDSGSMASGAHTRGGGPLPGARST	T	1045
4368	LOC_Os02g45070.1	AAFRARFYMEPDTSDSGSMASGAHT	T	1033
4370	LOC_Os02g47130.1	VEIPQADLTGDKTSEEEDLNEVKEG	T	399
4373	LOC_Os02g48010.1	EVKKDDGQGDSVTVGGTRKRRFAGA	T	965
4378	LOC_Os02g49450.1	DGLDDDFDKIDGTSGLESDDDDDKE	T	280
4380	LOC_Os02g50970.1	NKTFLSSRSTAGTAEWMAPEVLRNE	T	999
4381	LOC_Os02g52250.1	PVPVMHSPPRPVTVKDQQDWKIPPC	T	253
4387	LOC_Os02g54110.1	EDGDNMSMTSRATRQSRWNHTQMFS	T	1044
4388	LOC_Os02g54110.1	DTGELPQTPRSATGDDELSLMDGEP	T	17
4390	LOC_Os02g54500.1	LLRSGRDFENASTDSEKLSPRFLSY	T	931
4392	LOC_Os02g57080.1	IEGARDPRFALSTGGHAQSPALQNR	T	698
4395	LOC_Os02g58080.1	DAEMELVSLNGGTPRGGSPKDPDAT	T	34
4398	LOC_Os03g01470.1	RRGPHGDGSAEPTARAAAASARRRL	T	113
4403	LOC_Os03g04710.1	TIQSFTQRGGASTDEEEEEDDDDGD	T	118
4405	LOC_Os03g05390.1	PAGPEVVADDEVTSHRFTPARMSHV	T	230
4406	LOC_Os03g06340.1	PPPQPQQQPRVETPPPPSYGFHNLD	T	95
4408	LOC_Os03g11140.1	SADETDFCSDDETLIEKLENNKTDL	T	550
4409	LOC_Os03g11580.1	LLESKSPDGVGQTFRAQPGVKLGIS	T	424
4414	LOC_Os03g15050.1	ELHSLQRKRSAPTTPIKDGASSPFA	T	53
4417	LOC_Os03g15810.1	AGADQPRRSPQETTAAPSKPADEVS	T	57
4419	LOC_Os03g16369.1	KGTDKHLPSQVRTDSSGEEEGSRAR	T	540
4423	LOC_Os03g17980.1	LSNVMHDGHFLKTSCGSPNYAAPEV	T	216
4428	LOC_Os03g21530.1	GSDLSGEEVDNDTKKSKREETNGGG	T	734
4429	LOC_Os03g22320.1	AIAAQLQQNALLTRKMNSTKDDSSS	T	428
4431	LOC_Os03g22880.1	VAHANGDAEENGTPKKKKKKNREVS	T	516
4433	LOC_Os03g27260.1	KVSKAPKIQRLVTPLTLQRKRARIA	T	185
4435	LOC_Os03g27260.1	KKGDNDLPGLTDTEKPRMRGPKRAS	T	129
4439	LOC_Os03g30460.1	IVDGEVNDEYMKTLETLSRKIKFIE	T	184
4441	LOC_Os03g31750.1	AAAGILTARGGMTSHAAVVARGWGK	T	467
4443	LOC_Os03g33110.1	TDVVVVPRSREATPSGPVGDLAPAR	T	196
4444	LOC_Os03g40010.1	SSSKKFDDDDDITPSKKRNKALEYD	T	479
4445	LOC_Os03g40110.1	ARDWEQSDDDEVTVEEDEMEGLKQK	T	445
4447	LOC_Os03g43800.1	SGDRMFLSPVKSTDRKNADSEMGSS	T	352
4451	LOC_Os03g46046.1	LGGQLRSTVGTGTKLREELRSRRSR	T	42
4452	LOC_Os03g46570.1	QVWRDLAQSNEATANALRGELEHVL	T	217
4456	LOC_Os03g49170.1	AANSMLSPVSSPTAEDARSPSAAVM	T	232
4457	LOC_Os03g50330.1	HKSAEDPDGSFYTGDDPDRSFYDRD	T	52
4458	LOC_Os03g50480.1	IRERVGADGSKATGAFILTASHNPG	T	116

4460	LOC Os03g51020.1	RHHKLDDAQEEDTGTTLDDEEAAAL	T	744
4461	LOC Os03g51230.1	KMETIADVEGVDTPDEEEPVEEKKK	T	210
4462	LOC Os03g51520.1	KKQKLISRAEQPTGHNSSDQDELRT	T	762
4463	LOC Os03g51600.1	TKRTIQFVDWCPTGFKCGINYQPPS	T	349
4464	LOC Os03g52310.1	QEHKLALTPAVGTDNDGEGTVERPS	T	137
4467	LOC Os03g53630.1	PSLKKARDTSSDTELEQPKMSSGKK	T	368
4468	LOC Os03g53920.1	DTQYEDVEMRDATDDEDLEVKHVHD	T	838
4470	LOC Os03g54780.1	PDMQEAKTMPPSTGTVKKLKVAEGT	T	663
4471	LOC Os03g54780.1	MQEAKTMPPSTGTVKKLKVAEGTMP	T	665
4475	LOC Os03g60530.1	SIARDIKQRLSETDSKPEDRTIKIK	T	184
4477	LOC Os03g61930.1	LQSKTKEHASDGTSSVLEPEVELSH	T	499
4481	LOC Os03g63940.1	DFDQINTILSPSTPGSRMNMDVDND	T	117
4482	LOC Os03g64320.1	IRDGDPGGKSPPTLEVTDGKKADVE	T	1000
4486	LOC Os04g06770.1	DMSDASSSQGGHTSVGSVPVPELPR	T	887
4487	LOC Os04g09860.1	DKDADVDPLQRSTPDDTEPNSEENM	T	161
4489	LOC Os04g17064.1	LVEQMLEPGKRVTIAMGTNRDITTA	T	213
4490	LOC Os04g21220.1	LFAGSVWGFQMVTTRRNVNTGEGNQ	T	307
4492	LOC Os04g23060.1	RDNTSAINIAKNTVQHSRTRHIDIR	T	646
4493	LOC Os04g23330.1	IYGSRRRREPSSTRYRETLPPDKAI	T	100
4495	LOC Os04g24210.1	KKFVSQLGVMTSTSRPMDLYCDNSG	T	621
4496	LOC Os04g26660.1	RIDDLFDQLKGTTVFSKIDLRSGYH	T	925
4497	LOC Os04g28090.1	TATRALLSSYSQTPRLGMTPLRTPQ	T	342
4503	LOC Os04g34450.1	AAATRKALTNVQTLPRGVEVLDPLG	T	174
4506	LOC Os04g35800.1	PRKRRLSPTPSPTRRSSRSRSRSPR	T	60
4510	LOC Os04g38570.1	SGSLRNLSYSYSTGADGRIEMVSNA	T	655
4511	LOC Os04g38870.1	NLTLWTSDISEDTAEEIREAPKRDS	T	246
4512	LOC Os04g39240.1	TSTRSFAFPVLQTEWNSSPVKMAKA	T	631
4513	LOC Os04g41100.1	GMDSEYEVRRSETPEPVKPPHRCIN	T	342
4514	LOC Os04g42320.1	LKQQEDTPPDEDTDEDLSLKDIVSP	T	534
4516	LOC Os04g42320.1	HNLKKSHKLDTSTDSEVVDHSKAVN	T	394
4518	LOC Os04g42320.1	EDILVSRELSPETDDGDNKLPPETG	T	421
4524	LOC Os04g52460.1	QVKEAHNDEDDGTGSDGFELIDVKE	T	55
4526	LOC Os04g52820.1	LSVVEKEHPRSLTPMLWAKKIYSNL	T	168
4529	LOC Os04g53670.1	KLSRQNSFGSIGTPRTPNLHSTTDA	T	1133
4530	LOC Os04g54564.1	GHDERRRRLASSTVGFPPRYLRHWP	T	66
4533	LOC Os04g56620.1	PSHLFPSAKSVSTDLSQVPASQNTE	T	242
4536	LOC Os04g56740.1	AQLKFEELTSNLTVSRSNSLDGANK	T	563
4538	LOC Os04g59570.1	RPLRSRRALSVFTCAAPPRQRPPPG	T	28
4540	LOC Os05g02670.1	EEEYLVDEPSCSTPRRRQIDLPSME	T	988
4541	LOC Os05g02680.1	TASDAKKQKTDSTASGEDKDSEDPA	T	1236
4543	LOC Os05g03120.1	NETDSAGITPTRTTPERSPKVTERR	T	45
4546	LOC Os05g03550.1	AADVIPSDPCGKTGSGIDVDIEQRE	T	489
4548	LOC Os05g05300.1	SVFRESEDDELDTEDDELWGPDLKE	T	500

4549	LOC Os05g05370.1	CDHYECSFRKDKTPDKIRYYSRANI	T	281
4551	LOC Os05g08840.1	DTDDALGDPASGTDENMQESKCSAD	T	528
4556	LOC Os05g12100.1	RGSVQHCRHAQHTTSTPRRPACPEG	T	131
4557	LOC Os05g14940.1	QEHKLLLTPAGGTDNDGEGTVERPS	T	137
4558	LOC Os05g22390.1	RYCKWHNSRSHSTNDCKLFRQQIQV	T	253
4559	LOC Os05g22580.1	DTGDAVLPASRITADRHLSLPCKIM	T	16
4561	LOC Os05g23940.1	EARESDVENNITTPPKNIIGDKHST	T	372
4563	LOC Os05g28190.1	KDADNKGQSSSPTPLFSFKNLSSGQ	T	250
4564	LOC Os05g28190.1	TDKSAGSSEVAETEKDGSDLKGSDE	T	133
4566	LOC Os05g32360.1	ARSFSNDHNGNLTNRVITLWYRPPE	T	198
4568	LOC Os05g33310.1	STPVDLSPLPSPTKAMTLWQAPPPQ	T	249
4569	LOC Os05g34790.1	VDLDSDGSEDHGTRQHEAKPKLCSG	T	164
4571	LOC Os05g37480.1	DEGGRVTARGGWTGWGTGAAVSMRQ	T	104
4575	LOC Os05g42230.1	HPASPVAGGEKATPPSTPPPVRLAG	T	16
4576	LOC Os05g42230.1	PVAGGEKATPPSTPPPVRLAGGAAA	T	20
4577	LOC Os05g42300.1	GYDGSGENDEDQTDPEFSDDEKEAE	T	374
4579	LOC Os05g43380.1	QSPISKESSFQQTPPHIRATNRDTH	T	258
4580	LOC Os05g43520.1	KSLAIEPKIHSETDEDEVDEWPDDD	T	377
4581	LOC Os05g44320.1	DGVLDFLNSKKMTDAGALDLIDDED	T	668
4582	LOC Os05g46780.1	PRSSRSAAHGEDTSSVTDAASRSVD	T	301
4584	LOC Os05g48820.1	ERTYTEDPYPNETMRAELSVKLGLT	T	84
4585	LOC Os05g50160.1	ERAKGGPAPALPTATGGGKRLLEET	T	186
4586	LOC Os05g50480.1	ADEHVASSTKSDTENVDVAPVQQPE	T	490
4587	LOC Os05g50480.1	VAPAADEHVASSTKSDTENVDVAPV	T	486
4588	LOC Os05g50530.1	SARNLPKDIPLPTESVSRGKRRAVS	T	177
4591	LOC Os06g01700.1	DESDEDDDDDDDTEALMAELERIKK	T	133
4593	LOC Os06g02510.1	PRRARKVKAGDSTPEELATATQVQG	T	139
4594	LOC Os06g03780.1	EQSREYLALHPQTSLKEPRLIEEHF	T	529
4595	LOC Os06g04660.1	DDPELLNIIPLETAVQETGRSSDEG	T	425
4597	LOC Os06g05190.1	PRSDDAQYDSDDTIEMTEEEIDLAC	T	325
4599	LOC Os06g05660.1	DEDEPILEKAIGTEIEWYPGKNLTQ	T	214
4603	LOC Os06g06830.1	ERRRNDRSAPHATEGASSSRVSPQH	T	119
4607	LOC Os06g11610.1	PLRRYDVVDESGTDSGDEYDATDDG	T	79
4608	LOC Os06g11610.1	ESGTDSGDEYDATDDGRRLTVPFFF	T	88
4609	LOC Os06g13390.1	KKVYLNEENIYITKTTPQSVVKLFQ	T	155
4610	LOC Os06g13680.1	AEHGLRNYVRDKTPEIMPAINKFFT	T	56
4612	LOC Os06g14412.1	SQAPYDASDKEDTAADLIGPKQATL	T	2483
4614	LOC Os06g17840.1	VEGAKKRDDSVDTELVTSPASVKTP	T	1275
4617	LOC Os06g22820.1	QVPESKDEDDDSTEEWANFRGGDSD	T	444
4620	LOC Os06g30570.1	VNENERTNVNEPTNVNENERTNMNE	T	15
4623	LOC Os06g34710.1	EVTTPATEKVEVTTPATEEAKITTA	T	895
4624	LOC Os06g34710.1	VTTPATEKVEVTTPATEEAKITTAA	T	896
4630	LOC Os06g37770.1	PMAVGDAATAVSTLSCRAPRPRRSS	T	72

4634	LOC Os06g49500.1	LDPLLVEDEDPPTPKTQESSFTGSI	T	399
4636	LOC Os06g50890.1	ITVEKSRRGRPRTPTPGSYLGHRYD	T	148
4637	LOC Os06g50890.1	VEKSRRGRPRTPTPGSYLGHRYDRR	T	150
4639	LOC Os06g51250.1	TDAFARFDSMKSTDYNSRGYSFDED	T	1149
4642	LOC Os07g02230.1	LIENDDGSCGSETKNVVYHEILDAT	T	1401
4649	LOC Os07g08190.1	KHSSKKDPDNTKTISLEKDSTLEDA	T	341
4650	LOC Os07g08190.1	FTQQYAFARRYRTPSPERSPVRSRY	T	461
4652	LOC Os07g09384.1	PPPEPTLARIESTESFSLPLHKVDG	T	233
4655	LOC Os07g12910.1	VDDGYDEEEHSETLCGTCGGRYNAN	T	188
4656	LOC Os07g13340.1	GAKPKASNEANTTRMVNQNNVPMLG	T	1078
4657	LOC Os07g18300.1	PLEKCCDAKVRRTFSKIDLKLIERL	T	806
4659	LOC Os07g25460.1	KFEELQPLEEFTTPPSSPDNSKSPV	T	574
4661	LOC Os07g28800.1	GNFRSRGGFEFCTSDEDEPETVFRN	T	143
4667	LOC Os07g36420.1	KDAGAAKNPEVETSSSIHRSENPGL	T	167
4669	LOC Os07g37140.1	NVNASPASPPAATSDVTRDLPNGLM	T	362
4670	LOC Os07g37750.1	PVKAPDADGADKTDDEENPMMETVR	T	1457
4671	LOC Os07g38730.1	SLRFDGAINVDVTEFQTNLVPYPRI	T	253
4672	LOC Os07g38730.1	DGAINVDVTEFQTNLVPYPRIHFML	T	257
4674	LOC Os07g40270.1	LGSLEGFFPSDHTSSPGNKQIILQG	T	235
4675	LOC Os07g41180.1	NVEKAVSSPVKPTNAADTTSPNDKN	T	300
4676	LOC Os07g42300.1	DDDDDVDLFGEETEEEKKAAEERAA	T	115
4677	LOC Os07g42300.1	VKVESTAVPSASTPDVADAKAPAAD	T	89
4678	LOC Os07g42950.1	IVKKGENDLPGLTDTEKPRMRGPKR	T	127
4679	LOC Os07g43316.1	TIPDYLEGKSEVTLEHVSSGDESME	T	241
4684	LOC Os07g48020.1	FASNAAAFSSAFTTAMVKMGNISPL	T	289
4685	LOC Os07g49270.1	APWEKEIITDPSTPKPNPNPFYYEQ	T	256
4688	LOC Os08g01620.1	RTLVQPVGQKRLTNVAVVRLRKHGQ	T	15
4690	LOC Os08g02690.1	AETLSLASPRSPTGGSTSALLQQYE	T	37
4694	LOC Os08g07830.1	DIAKLLEAGQEATARIRVEHIIREE	T	61
4695	LOC Os08g08080.1	KESQDISPPRRRTRHDSEEPQDLSP	T	233
4697	LOC Os08g08220.1	SRQQFKAYAAAGTLKQNYANILLML	T	692
4699	LOC Os08g14340.1	KSANMIISEGGGTSGYSKILPTVLS	T	391
4701	LOC Os08g19320.1	AGDTSAAAAVAPTPARLREALAALS	T	35
4703	LOC Os08g24760.1	TEEVDDEDDGNDTDEEQERIRDQQE	T	171
4705	LOC Os08g25570.1	SRLWSGGAYESDTEDPDNMKTETND	T	431
4706	LOC Os08g25850.1	KIDSETKSNQPPTKKASSKPAPKKN	T	45
4707	LOC Os08g27040.1	ADEGHGAISLRDTSPILDTMESGRR	T	297
4708	LOC Os08g27150.1	RRPTKSAPSNLLTKREALQRSIEVQ	T	686
4712	LOC Os08g29650.1	QSKEVRYTARSITPPADRNGTSKSK	T	15
4713	LOC Os08g33370.1	LRDNLTLWTSDLTEDGGDEVKEASK	T	237
4717	LOC Os08g39140.1	ISYPISLWTEKTTEKEISDDEDEEE	T	215
4720	LOC Os08g40230.1	RDFESDFEEMHDTDEELDNLYNARQ	T	122
4722	LOC Os08g42600.1	VYVSPLRTSKMDTLLSPSSKSYYAC	T	925

4725	LOC_Os08g44420.1	QSSYKDYEPTGETPVRSEPEVPSKD	T	353
4726	LOC_Os09g01640.1	SSSRGRSKHRRSTKRSDTEDDLEEE	T	234
4727	LOC_Os09g01640.1	RSKHRRSTKRSDTEDDLEEERSKDK	T	239
4728	LOC_Os09g07170.1	ASSSKAPKKTKMTFIMLLNKYEKQG	T	260
4729	LOC_Os09g07294.1	KAVTSYLSSSPSTPESPVRLSALSG	T	518
4730	LOC_Os09g07900.1	RDGIGSVWSSSGTPSRDLHTFSFGT	T	2230
4731	LOC_Os09g16980.1	GMCTRMTGQDRPTMREVEMGLENLR	T	584
4734	LOC_Os09g23790.1	TSAPTAPRQRIATCDEVSVKEEDAL	T	780
4735	LOC_Os09g26820.1	DDLRRLAPPSPATVGSPRRTSSASG	T	70
4739	LOC_Os09g33450.1	SIDGGSDMGNGQTPQQLRLPRTKDV	T	130
4742	LOC_Os09g33600.1	TLGKALLLQEQPTSPSDGSSRCFDR	T	701
4746	LOC_Os09g36160.1	RPRARPSATTPTTSSGDQQMVTVAE	T	180
4748	LOC_Os09g36160.1	KRPRARPSATTPTTSSGDQQMVTVA	T	179
4749	LOC_Os09g37520.1	PADIPSTSRGEVTPPAPPSPPRVST	T	351
4751	LOC_Os09g39870.1	LAMRDCMENGFLTDDGVVHTVFDGN	T	616
4754	LOC_Os10g26140.1	NLAEHVDRTLLNTSSDSDDIIDASG	T	479
4757	LOC_Os10g31520.1	EDTATPYESAKSTSNNDEIADMAKS	T	342
4759	LOC_Os10g34370.1	NCRIQWFAKKGETFKGEELLDAMES	T	275
4760	LOC_Os10g39200.1	DSVSGLDSPGDATCSDEDNGRKAPS	T	1016
4762	LOC_Os10g41030.1	SACISYDSHTEETGLRKEWVPLRQE	T	1597
4763	LOC_Os10g42110.1	LMKNSRDGKSYSTNLAYTPPEFLRT	T	234
4765	LOC_Os11g02190.1	PSPIESPLSRSRTFEDNPIRHSTGK	T	623
4766	LOC_Os11g03360.1	SGERKLWLALWKTKVQAKIKIFAWK	T	193
4767	LOC_Os11g06650.1	GGGGNCGAAAATTPSPRGAAVAEGE	T	74
4768	LOC_Os11g07040.1	MRKGNAGGAGRRTMRNSLQLNLGEI	T	495
4770	LOC_Os11g10480.1	HTDVYFWEAKGQTPVFPRIFGHEAG	T	60
4772	LOC_Os11g19250.1	FTEKFDEETIGGTEMADDENLTKEQ	T	447
4778	LOC_Os11g31890.1	KSNFASDDLPSPTPSGDGDKSGDKG	T	459
4779	LOC_Os11g32900.1	KYHPLQATFASPTVSNPVSGNEKCA	T	691
4782	LOC_Os11g34660.1	CSYIRRKRLARKTSIPGSSSKWANH	T	191
4785	LOC_Os11g37100.1	SASPNGATSRDKTPSPQASERPISI	T	445
4787	LOC_Os11g42930.1	LGATTTDMPKFKTLSLNDSPSALDS	T	144
4790	LOC_Os11g47830.1	QAKEHDEKRRSYTPEYNDRRDADNG	T	360
4792	LOC_Os11g47830.1	SDRRDAFIGHDETPPSAEWGSKLGT	T	478
4794	LOC_Os12g05420.1	VPKVQTKPTYQRTLSGGLKSPRAAD	T	369
4798	LOC_Os12g06850.1	HNSGGDGGSSVLTDGDSPDKIQGRS	T	192
4799	LOC_Os12g06870.1	VPFFADDEESPATPKADAFFIPREN	T	701
4805	LOC_Os12g07530.1	TPSPGRDHRIRRTPSPYRDRRRIPD	T	131
4809	LOC_Os12g10410.1	VLGVWGMGGAGKTTLLKLARDPRVQ	T	219
4810	LOC_Os12g10410.1	LGVWGMGGAGKTTLLKLARDPRVQT	T	220
4811	LOC_Os12g13770.1	SDRHCTAGLTGGTRGLTGSAWPVGR	T	596
4812	LOC_Os12g19040.1	FVRHEADEEESDTSDDIVPVVVRPG	T	213
4814	LOC_Os12g21890.1	GPQQDESAEGSTTPTTAQSGVFGSF	T	136

4818	LOC_Os12g36510.1	TDCANIGRAMVNTGDDRSIPPLAGD	T	576
4819	LOC_Os12g37720.1	SASVADDIDDLDTKKTSVRDKPYIS	T	858
4820	LOC_Os12g38730.1	EDGSARKLKKVPTWERILKSREPNR	T	574
4824	LOC_Os12g43500.1	YSTEPPDDGKCVTKEDLESDEAVWA	T	92
4825	LOC_Os12g44330.1	QRTTGVSGLGEGTPKSERSADMFCD	T	548
4826	LOC_Os01g01150.1	RPSSPAQASRDAYSDADDDDDDRPH	Y	66
4827	LOC_Os01g01150.1	PIKYRRSRRSRSYSPPVRHTRGRRS	Y	544
4832	LOC_Os01g10840.1	AKVLVPGEPNISYICSRYYRAPELI	Y	225
4836	LOC_Os01g35050.1	RQSRRNTPVPSKYNGSESPSLAEIV	Y	751
4837	LOC_Os01g43170.1	LNVKFQSHRSRDYTNPYLPVAPSAI	Y	210
4839	LOC_Os01g48610.1	FSLYITTHGGYGYASPVTLGSAMVD	Y	456
4840	LOC_Os01g49640.1	LCRYAKNPDLRKYINSQNSRKVAAA	Y	83
4841	LOC_Os01g50320.1	RKKHHPESDEENYDSEESYKHSRKK	Y	730
4843	LOC_Os01g53680.1	FSATCVRISRDSYPNLRALRNASAM	Y	57
4844	LOC_Os01g57790.1	TVQGALSDQLGNYVEAYVNDIVVKT	Y	69
4847	LOC_Os02g05310.1	LLATPTPLGTPLYAIPEENRGQQFD	Y	421
4848	LOC_Os02g07070.1	DRDYDRYSYDSDYEKSRRDGSWRRR	Y	143
4850	LOC_Os02g08360.1	EDRKGGRGLGLSYSSGSDIAGDSGK	Y	789
4851	LOC_Os02g13250.1	ELENLSSSQKKGYFKPKREKDVLST	Y	251
4852	LOC_Os02g14130.1	AKMLVKGEANISYICSRYYRAPELI	Y	234
4853	LOC_Os02g18660.1	EEEEEEDSDTDTYAQGFQLRKVGGG	Y	75
4854	LOC_Os02g18910.1	FVLEKEDYPVVDYVLVIYAAKSATA	Y	47
4856	LOC_Os02g28520.1	GDEGGITLVQSHYVDKVLSRFGYSD	Y	699
4859	LOC_Os02g33360.1	FMQTRKGTVVQKYKVKVVADVPGTG	Y	77
4860	LOC_Os02g35190.1	QHRAPEREGSHNYDIESTDGSGGLW	Y	18
4861	LOC_Os02g37880.1	SLVSAMESRGTGYYEEKKALLILRD	Y	125
4865	LOC_Os02g46090.1	FRGASQRHDEAGYAPVATSAAAAAA	Y	29
4867	LOC_Os02g47220.1	AREVTSVPPYTEYVSTRWYRAPEVL	Y	158
4875	LOC_Os03g21530.1	KKLALKAKFDAEYDGSDLSGEEVDN	Y	720
4877	LOC_Os03g24890.1	PARRDYRDHRDDYSPGESLSPHGQD	Y	196
4879	LOC_Os03g40110.1	KDDFDEEVPLDDYSDEEGHMILSKN	Y	179
4880	LOC_Os03g50330.1	NHKSAEDPDGSFYTGDDPDRSFYDR	Y	51
4885	LOC_Os04g02870.1	ARGGYGRRDDSPYRRSVSPVYRSRP	Y	189
4886	LOC_Os04g02870.1	DDSPYRRSVSPVYRSRPSPDYGRQR	Y	197
4888	LOC_Os04g08450.1	ELCSSRVLSPPRYESGDEELSVLPR	Y	18
4889	LOC_Os04g13960.1	DGEVFGRDDTMDYRTIIVQRVLSTQ	Y	162
4891	LOC_Os04g35800.1	RSRSRGRSASPRYPDGKRRRHNDLN	Y	93
4893	LOC_Os04g40660.1	LDIPNAQEKFGAYVDLATERGWLLP	Y	647
4895	LOC_Os05g11770.1	WNIEPSLFFPKQYFSDTEEFEDVIY	Y	278
4897	LOC_Os05g27820.1	ALLRSSSFKEDSYVASALPASDLRA	Y	52
4898	LOC_Os05g40060.1	ARTSEDGFLWRKYGQKEIKNSKHPR	Y	135
4899	LOC_Os05g40230.1	KSLLRVTKDATAYRKVLKGLIVQSL	Y	110
4906	LOC_Os06g05190.1	KHQSKPRSDDAQYDSDDTIEMTEEE	Y	320

4910	LOC_Os06g08840.1	RRRSVSPARARSYSRSPQYNRGRDE	Y	160
4913	LOC_Os06g11610.1	RLYNTEGAPLRRYDVVDESGTDSGD	Y	71
4920	LOC_Os06g39600.1	TEKTFENMDAVAYMPVVRSGGWADI	Y	62
4921	LOC_Os06g43610.1	KNGGGKEKPGSDYSEDDGETAEPNK	Y	356
4922	LOC_Os06g45560.1	IDVAIVGSLGGSYLRSYNYKPATRP	Y	799
4923	LOC_Os06g48530.1	REYDRAERDGSRYGRGDRSPSLDGA	Y	593
4924	LOC_Os06g49740.1	DAEQEDAGSDDGYPQMTEKQKKLFE	Y	122
4925	LOC_Os06g50890.1	GRARRERSRSLPYSPYRMPERGYGR	Y	252
4927	LOC_Os06g50890.1	PERRRYSGSPSPYRGNPKSRSRSRS	Y	17
4928	LOC_Os06g51250.1	AFARFDSMKSTDYNSRGYSFDEDDP	Y	1151
4929	LOC_Os07g01880.1	VVASESEGSEDDYYAGRGHEDEEPH	Y	181
4930	LOC_Os07g05190.1	SRFSWSPDSGEAYTQEGLARLDVRS	Y	747
4931	LOC_Os07g05610.1	NLQHSLSRNARVYSGIDLSKDEVTQ	Y	814
4932	LOC_Os07g08190.1	RGFTQQYAFARRYRTPSPERSPVRS	Y	459
4933	LOC_Os07g19130.1	SKVCEEDIQNMKYLKMIIKENFRLH	Y	81
4934	LOC_Os07g25680.1	LQPSDSAKSLETYTASKAMSAAQDS	Y	513
4935	LOC_Os07g31460.1	SSTTRFPRYNDPYKLLETRKGRCGE	Y	314
4938	LOC_Os07g41190.1	RSGGDGEDDLPVYDEGLSSEDEGVD	Y	121
4940	LOC_Os08g02690.1	RNDPNYDSDEEPYELVEAPVSTPVE	Y	123
4943	LOC_Os08g16930.1	EPSSEEMEELHGYQEPVRVGRVMRY	Y	171
4944	LOC_Os08g17120.1	SAPAKEAIKLSDYERTLKKASSRKS	Y	508
4946	LOC_Os09g19952.1	YEETVMILNLMPYRASYPILKLVYS	Y	30
4947	LOC_Os09g28220.1	PYRSRRDRSPSPYRDRRRQWSPYHR	Y	62
4948	LOC_Os09g31230.1	PDLRASMVQRRGYTSDDDLDDLGNP	Y	722
4959	LOC_Os11g44880.1	ESKQVGRSAPLKYGGILKPSMSGKH	Y	184
4960	LOC_Os12g03899.1	KKGSTGTGVDDGYCIEGCPGCVVDR	Y	29
4962	LOC_Os12g06850.1	RSYQNAQVSDFKYSPSPQRHNGLSS	Y	215
4964	LOC_Os12g38970.1	QPDDVVADEDMRYESDENRELKPKE	Y	261
4965	LOC_Os12g41920.1	TASGKLIKVNRKYRIAPSSSLSEGR	Y	182
